# Supplementary material for: SUMO is a pervasive regulator of meiosis
Source: eLife. 2021 Jan 27;10:e57720. doi: 10.7554/eLife.57720 (PMC7924959; doi:10.7554/eLife.57720)
Supplement: Supplementary file 1. [file elife-57720-supp1.docx]

| **Study** | **SUMOylated proteins reported** | **Overlap with this study** |
| --- | --- | --- |
| (Esteras et al., 2017) | 244 | 166 |
| (Lewicki et al., 2015) | 195 | 126 |
| (Albuquerque et al., 2015) | 31 | 26 |
| (Albuquerque et al., 2013) | 176 | 131 |
| (Hannich et al., 2005) | 150 | 93 |
| (Denison et al., 2005) | 251 | 151 |
| (Panse et al., 2004) | 151 | 51 |
| (Wohlschlegel et al., 2004) | 271 | 163 |

**Supplementary File 1: Comparison of SUMOylated proteins identified in this study with data from previous studies**

**References:**

Albuquerque, C.P., Wang, G., Lee, N.S., Kolodner, R.D., Putnam, C.D., and Zhou, H. (2013). Distinct SUMO ligases cooperate with Esc2 and Slx5 to suppress duplication-mediated genome rearrangements. PLoS Genet *9*, e1003670.

Albuquerque, C.P., Yeung, E., Ma, S., Fu, T., Corbett, K.D., and Zhou, H.L. (2015). A Chemical and Enzymatic Approach to Study Site-Specific Sumoylation. Plos One *10*.

Denison, C., Rudner, A.D., Gerber, S.A., Bakalarski, C.E., Moazed, D., and Gygi, S.P. (2005). A proteomic strategy for gaining insights into protein sumoylation in yeast. Mol Cell Proteomics *4*, 246-254.

Esteras, M., Liu, I.C., Snijders, A.P., Jarmuz, A., and Aragon, L. (2017). Identification of SUMO conjugation sites in the budding yeast proteome. Microb Cell *4*, 331-341.

Hannich, J.T., Lewis, A., Kroetz, M.B., Li, S.J., Heide, H., Emili, A., and Hochstrasser, M. (2005). Defining the SUMO-modified proteome by multiple approaches in Saccharomyces cerevisiae. J Biol Chem *280*, 4102-4110.

Lewicki, M.C., Srikumar, T., Johnson, E., and Raught, B. (2015). The S. cereuisiae SUMO stress response is a conjugation-deconjugation cycle that targets the transcription machinery. J Proteomics *118*, 39-48.

Panse, V.G., Hardeland, U., Werner, T., Kuster, B., and Hurt, E. (2004). A proteome-wide approach identifies sumoylated substrate proteins in yeast. J Biol Chem *279*, 41346-41351.

Wohlschlegel, J.A., Johnson, E.S., Reed, S.I., and Yates, J.R., 3rd (2004). Global analysis of protein sumoylation in Saccharomyces cerevisiae. J Biol Chem *279*, 45662-45668.
