# Supplementary material for: SUMO is a pervasive regulator of meiosis
Source: eLife. 2021 Jan 27;10:e57720. doi: 10.7554/eLife.57720 (PMC7924959; doi:10.7554/eLife.57720)
Supplement: Supplementary file 3. [file elife-57720-supp3.docx]

**Supplementary File 3: Comparison of SUMO sites identified in this study with sites predicted by SUMOsp** (Zhao et al., 2014)

|  |  | **Number identified by SUMOsp**  **(prediction threshold)** | | |
| --- | --- | --- | --- | --- |
| **Lysine class** | **Number in dataset** | **(High)** | **(Medium)** | **(Low)** |
| **SUMOylated** | 2745 | 321 | 428 | 512 |
| **non-SUMOylated** | 29847 | 528 | 1105 | 1778 |

**Reference:**

Zhao, Q., Xie, Y., Zheng, Y., Jiang, S., Liu, W., Mu, W., Liu, Z., Zhao, Y., Xue, Y., and Ren, J. (2014). GPS-SUMO: a tool for the prediction of sumoylation sites and SUMO-interaction motifs. Nucleic Acids Res *42*, W325-330.
