# Supplementary material for: SUMO is a pervasive regulator of meiosis
Source: eLife. 2021 Jan 27;10:e57720. doi: 10.7554/eLife.57720 (PMC7924959; doi:10.7554/eLife.57720)
Supplement: Supplementary file 5. — Upper Track: Red line – SUMOylated lysine, Black line – lysine, Predicted SUMO Interaction Motifs (gray boxes): Dark gray – high threshold, Medium gray – medium threshold, Light gray – low threshold; Middle Track: Blue box - PFAM domains; Lower Track - Protein Secondary Structure: Purple – Globular Domains, Orange-red – Disorder score, Red – coiled-coil, Black – Transmembrane; Horizontal Axis: amino acid residue number [file elife-57720-supp5.pdf]

Protein Diagram Legend:

Upper Track

- SUMOylated lysine
- Lysine

Predicted SUMO Interaction Motifs

- High threshold
- Medium threshold
- Low threshold

MiddleTrack

- PFAM domains

Lower Track

Protein Secondary Structure

- Globular Domains
- Disorder Score
- Coiled-coil
- Transmembrane

Horizontal axis

Amino acid residue number

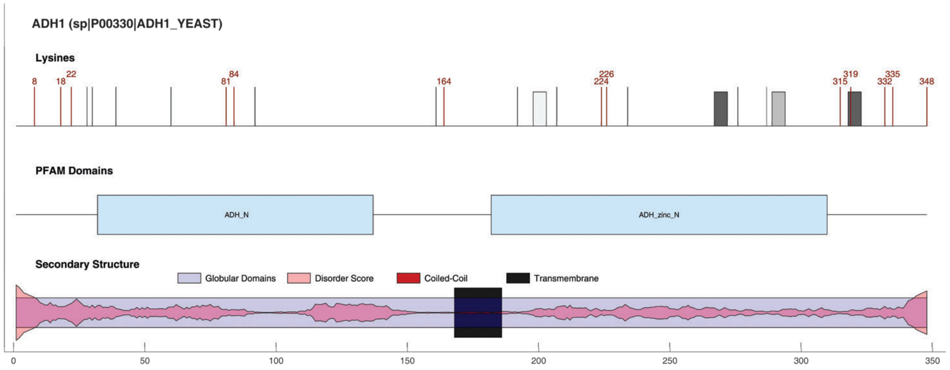

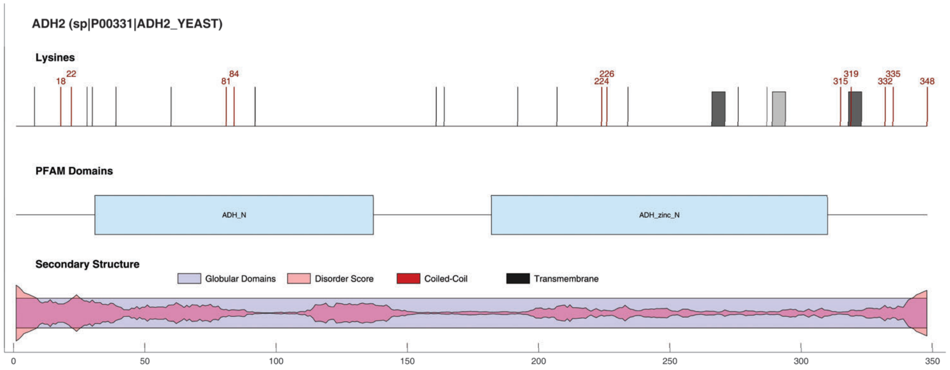

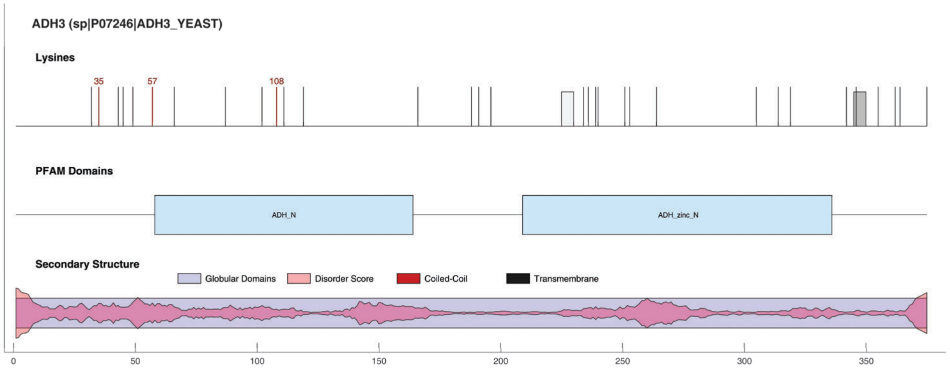

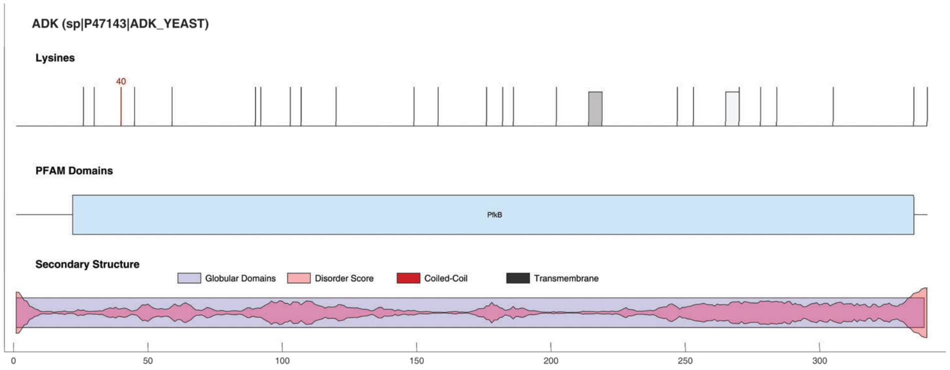
ADK (sp|P47143|ADK_YEAST)
Lysines
40
PFAM Domains
PfkB
Secondary Structure
Globular Domains
Disorder Score
Coiled-Coil
Transmembrane
0
50
100
150
200
250
300

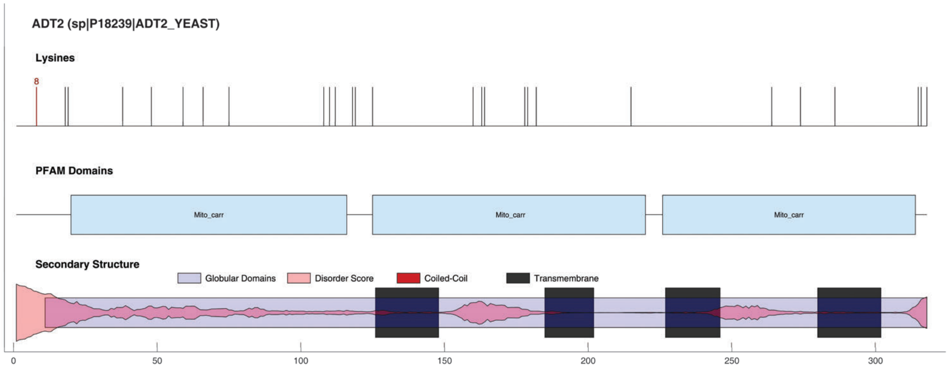
ADT2 (sp|P18239|ADT2_YEAST)
Lysines
8
PFAM Domains
Mito_carr
Mito_carr
Mito_carr
Secondary Structure
Globular Domains
Disorder Score
Coiled-Coil
Transmembrane
0
50
100
150
200
250
300

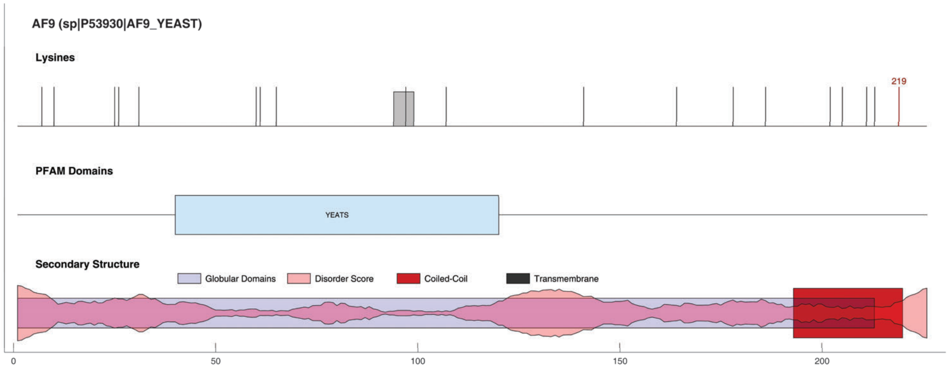
AF9 (sp|P53930|AF9_YEAST)
Lysines
219
PFAM Domains
YEATS
Secondary Structure
Globular Domains
Disorder Score
Coiled-Coil
Transmembrane
0
50
100
150
200

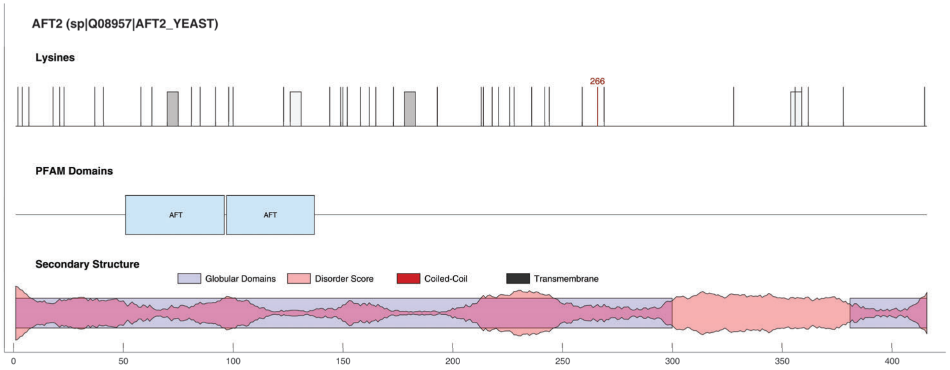
AFT2 (sp|Q08957|AFT2_YEAST)
Lysines
266
PFAM Domains
AFT
AFT
Secondary Structure
Globular Domains
Disorder Score
Coiled-Coil
Transmembrane
0
50
100
150
200
250
300
350
400

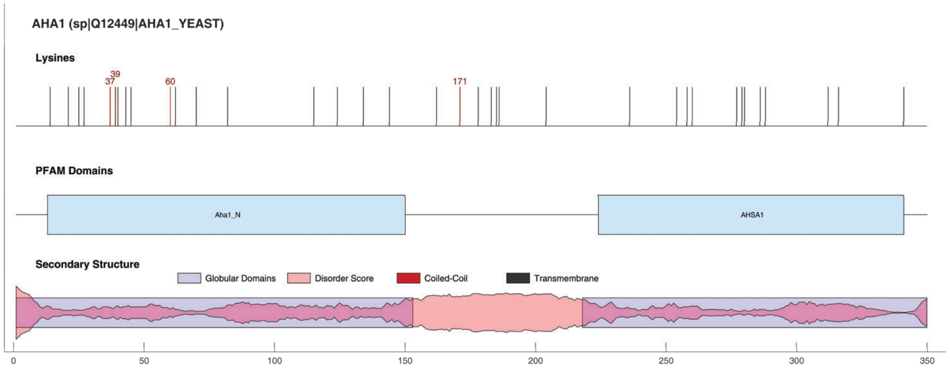
AHA1 (sp|Q12449|AHA1_YEAST)
Lysines
39
37
60
171
PFAM Domains
Aha1_N
AHSA1
Secondary Structure
Globular Domains
Disorder Score
Coiled-Coil
Transmembrane
0
50
100
150
200
250
300
350

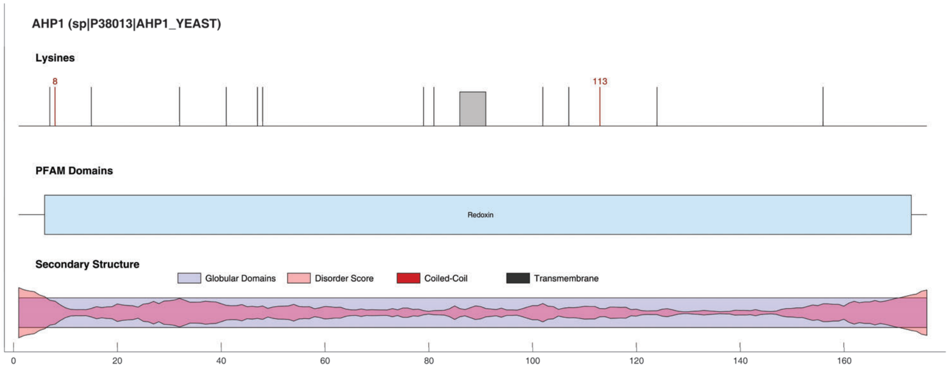
AHP1 (sp|P38013|AHP1_YEAST)
Lysines
8
113
PFAM Domains
Redoxin
Secondary Structure
Globular Domains
Disorder Score
Coiled-Coil
Transmembrane
0
20
40
60
80
100
120
140
160

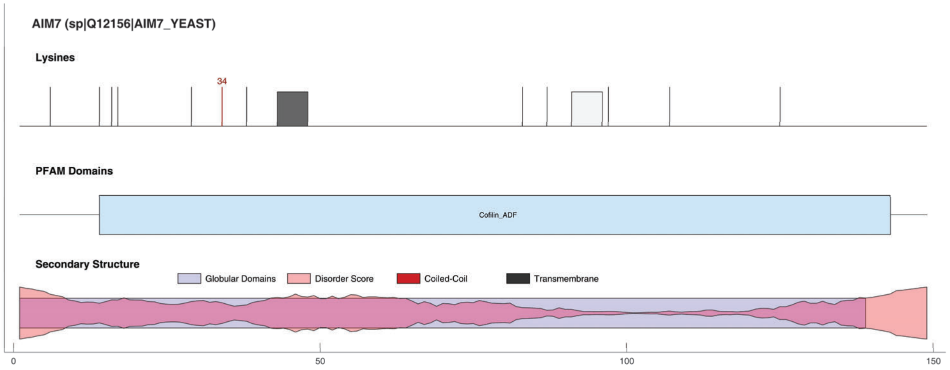
AIM7 (sp|Q12156|AIM7_YEAST)
Lysines
34
PFAM Domains
Cofilin_ADF
Secondary Structure
Globular Domains
Disorder Score
Coiled-Coil
Transmembrane
0
50
100
150

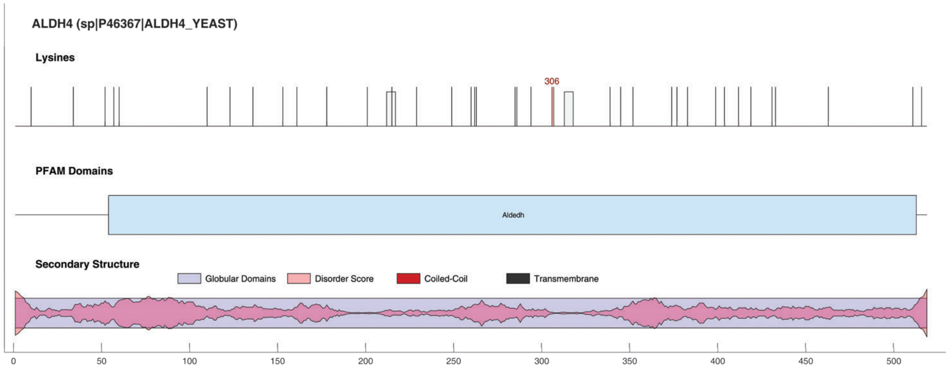
ALDH4 (sp|P46367|ALDH4_YEAST)
Lysines
306
PFAM Domains
Aldedh
Secondary Structure
Globular Domains
Disorder Score
Coiled-Coil
Transmembrane
0
50
100
150
200
250
300
350
400
450
500

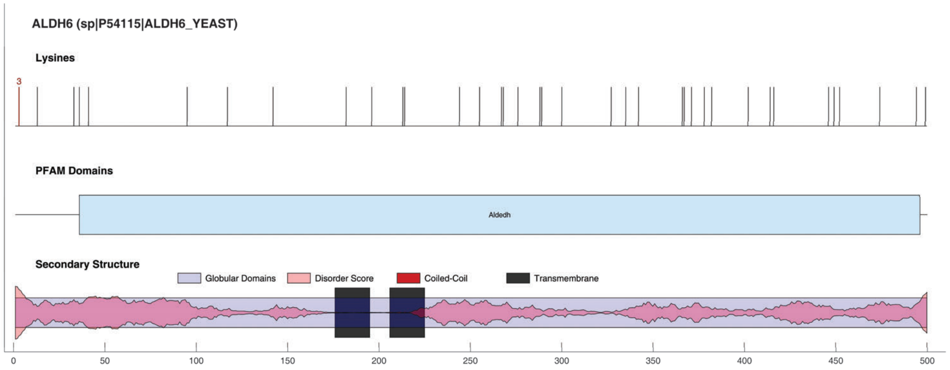
ALDH6 (sp|P54115|ALDH6_YEAST)
Lysines
3
PFAM Domains
Aldedh
Secondary Structure
Globular Domains
Disorder Score
Coiled-Coil
Transmembrane
0
50
100
150
200
250
300
350
400
450
500

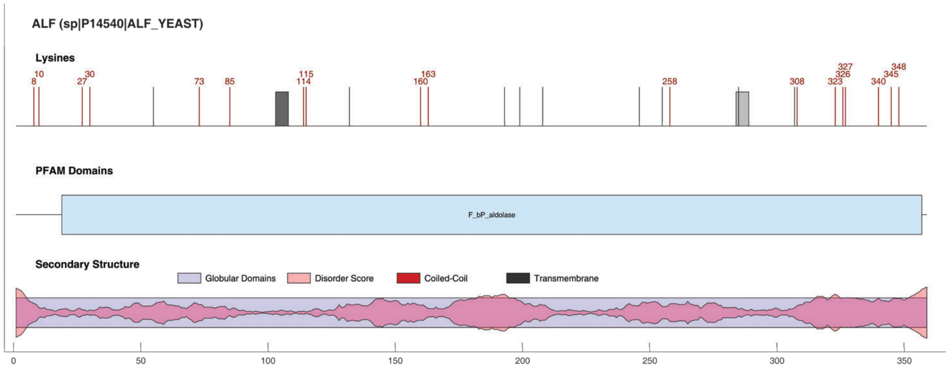
ALF (sp|P14540|ALF_YEAST)
Lysines
8
10
27
30
73
85
114
115
160
163
258
308
323
326
327
340
345
348
PFAM Domains
F_bP_aldolase
Secondary Structure
Globular Domains
Disorder Score
Coiled-Coil
Transmembrane
0
50
100
150
200
250
300
350

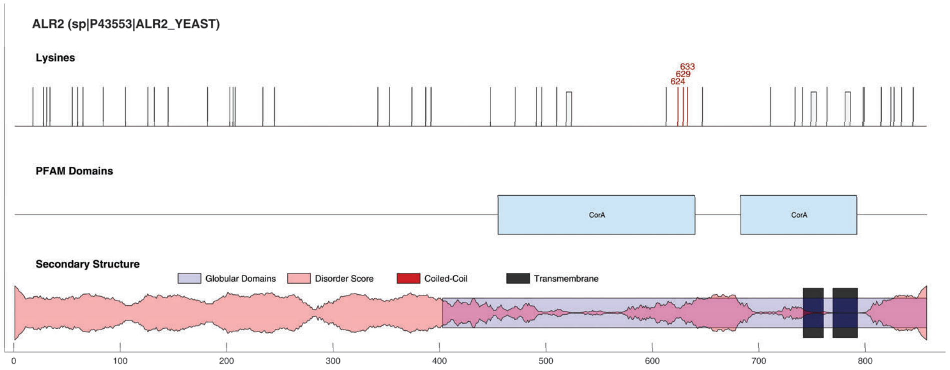
ALR2 (sp|P43553|ALR2_YEAST)
Lysines
624
629
633
PFAM Domains
CorA
CorA
Secondary Structure
Globular Domains
Disorder Score
Coiled-Coil
Transmembrane
0
100
200
300
400
500
600
700
800

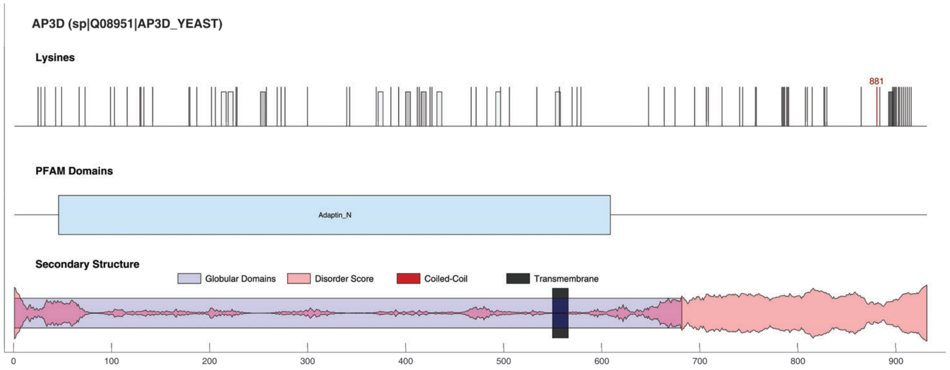
AP3D (sp|Q08951|AP3D_YEAST)
Lysines
881
PFAM Domains
Adaptin_N
Secondary Structure
Globular Domains
Disorder Score
Coiled-Coil
Transmembrane
0
100
200
300
400
500
600
700
800
900

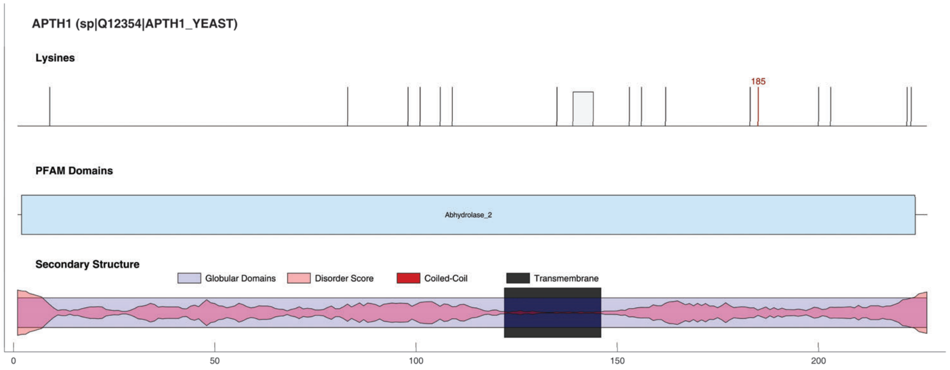
APTH1 (sp|Q12354|APTH1_YEAST)
Lysines
185
PFAM Domains
Abhydrolase_2
Secondary Structure
Globular Domains
Disorder Score
Coiled-Coil
Transmembrane
0
50
100
150
200

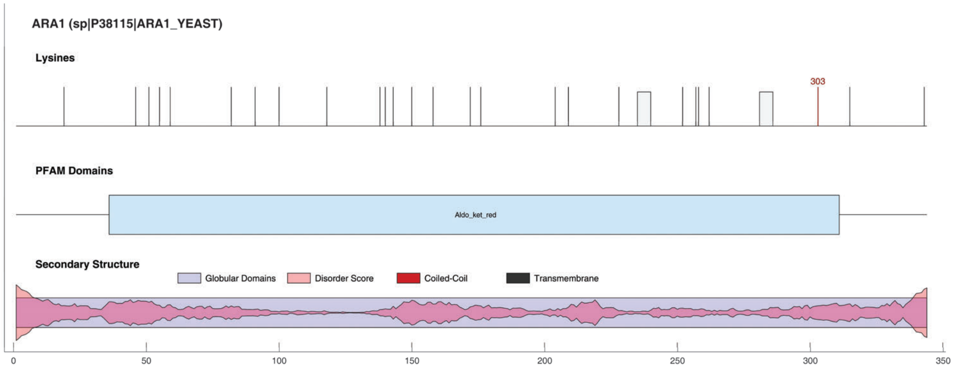
ARA1 (sp|P38115|ARA1_YEAST)
Lysines
303
PFAM Domains
Aldo_ket_red
Secondary Structure
Globular Domains
Disorder Score
Coiled-Coil
Transmembrane
0
50
100
150
200
250
300
350

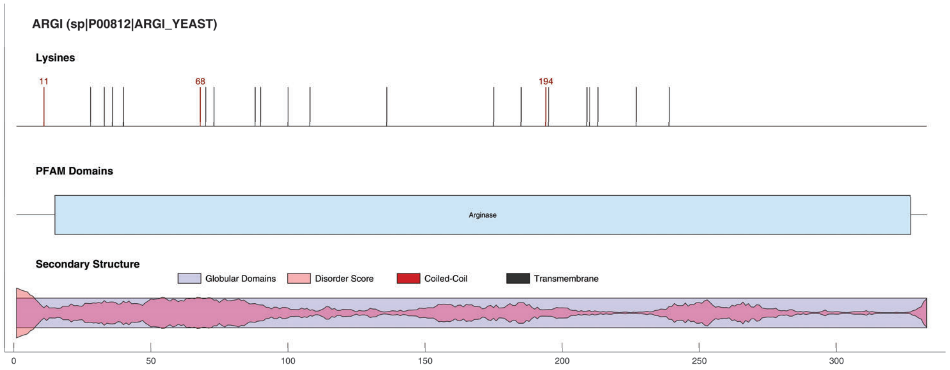
ARGI (sp|P00812|ARGI_YEAST)
Lysines
11
68
194
PFAM Domains
Arginase
Secondary Structure
Globular Domains
Disorder Score
Coiled-Coil
Transmembrane
0
50
100
150
200
250
300

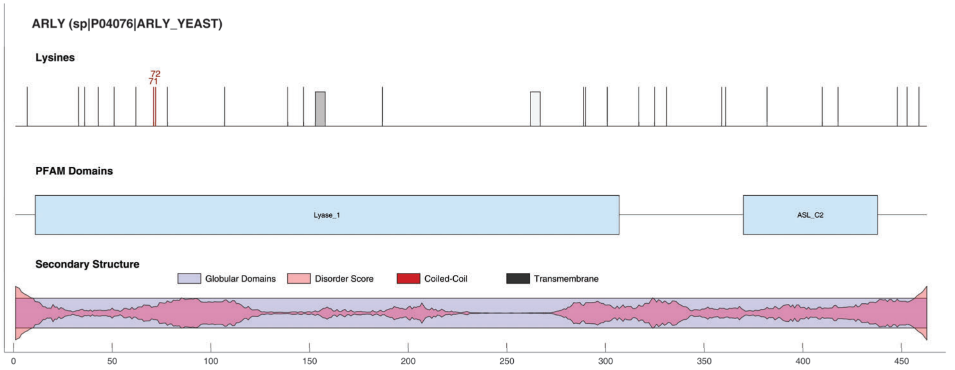
ARLY (sp|P04076|ARLY_YEAST)
Lysines
72
71
PFAM Domains
Lyase_1
ASL_C2
Secondary Structure
Globular Domains
Disorder Score
Coiled-Coil
Transmembrane
0
50
100
150
200
250
300
350
400
450

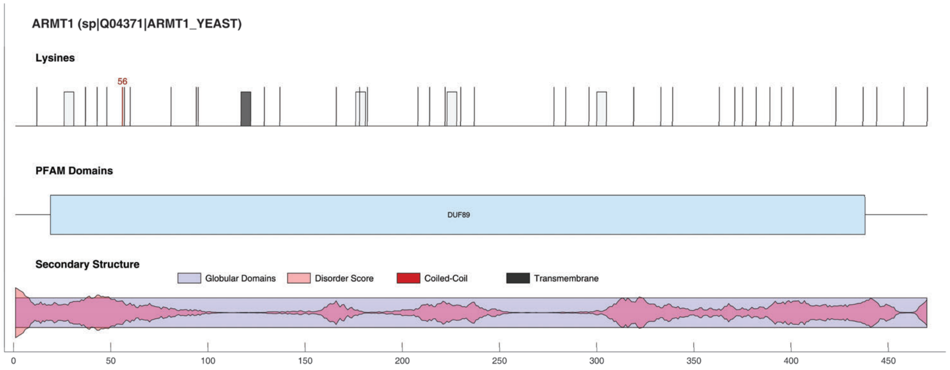

ARMT1 (sp|Q04371|ARMT1_YEAST)
Lysines
56
PFAM Domains
DUF89
Secondary Structure
Globular Domains
Disorder Score
Coiled-Coil
Transmembrane
0
50
100
150
200
250
300
350
400
450

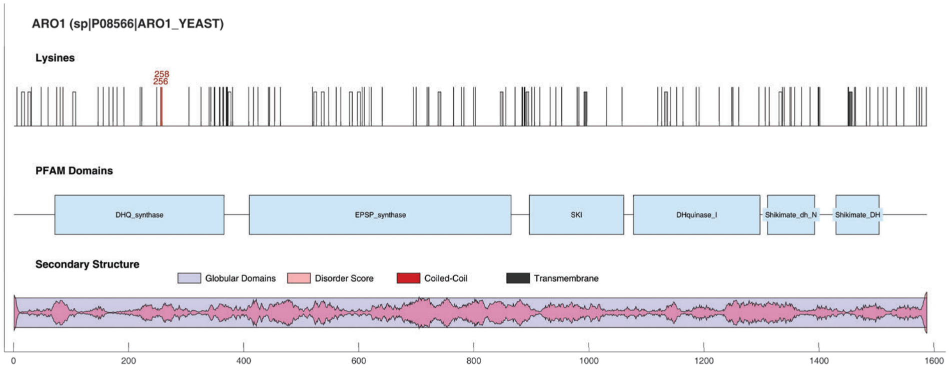

ARO1 (sp|P08566|ARO1_YEAST)
Lysines
258
256
PFAM Domains
DHQ_synthase
EPSP_synthase
SKI
DHquinase_I
Shikimate_dh_N
Shikimate_DH
Secondary Structure
Globular Domains
Disorder Score
Coiled-Coil
Transmembrane
0
200
400
600
800
1000
1200
1400
1600

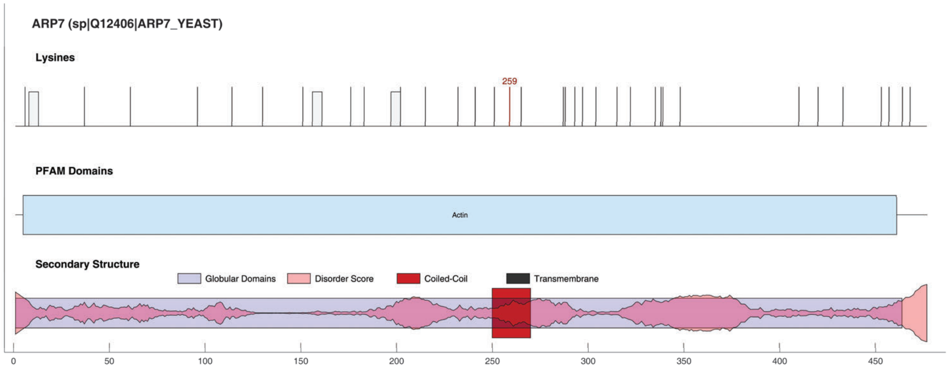

ARP7 (sp|Q12406|ARP7_YEAST)
Lysines
259
PFAM Domains
Actin
Secondary Structure
Globular Domains
Disorder Score
Coiled-Coil
Transmembrane
0
50
100
150
200
250
300
350
400
450

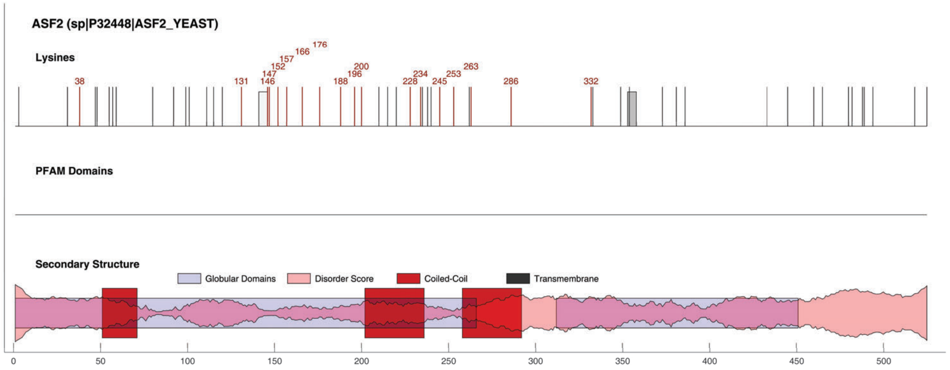

ASF2 (sp|P32448|ASF2_YEAST)
Lysines
38
131
146
147
152
157
166
176
188
196
200
228
234
245
253
263
286
332
PFAM Domains
Secondary Structure
Globular Domains
Disorder Score
Coiled-Coil
Transmembrane
0
50
100
150
200
250
300
350
400
450
500

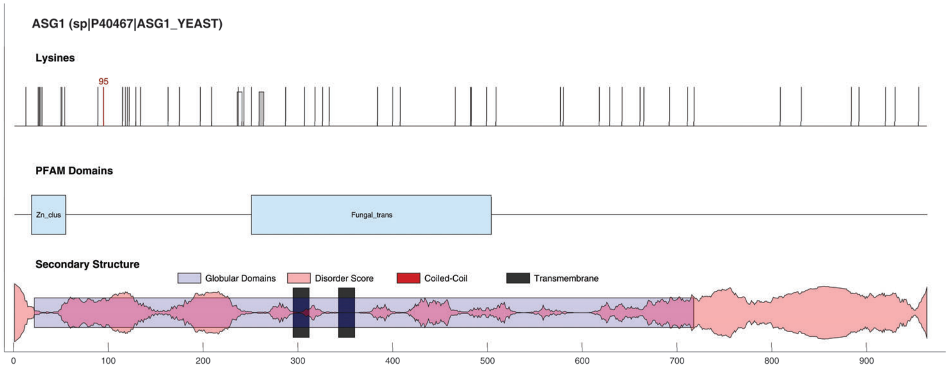
ASG1 (sp|P40467|ASG1_YEAST)
Lysines
95
PFAM Domains
Zn_clus
Fungal_trans
Secondary Structure
Globular Domains
Disorder Score
Coiled-Coil
Transmembrane
0
100
200
300
400
500
600
700
800
900

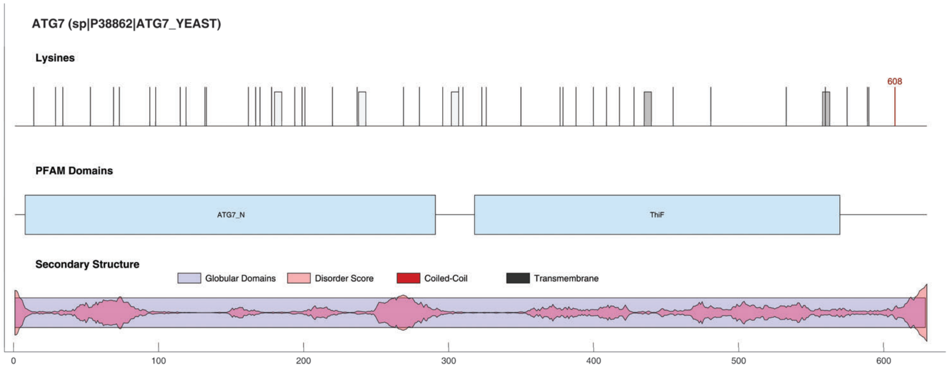
ATG7 (sp|P38862|ATG7_YEAST)
Lysines
608
PFAM Domains
ATG7_N
ThiF
Secondary Structure
Globular Domains
Disorder Score
Coiled-Coil
Transmembrane
0
100
200
300
400
500
600

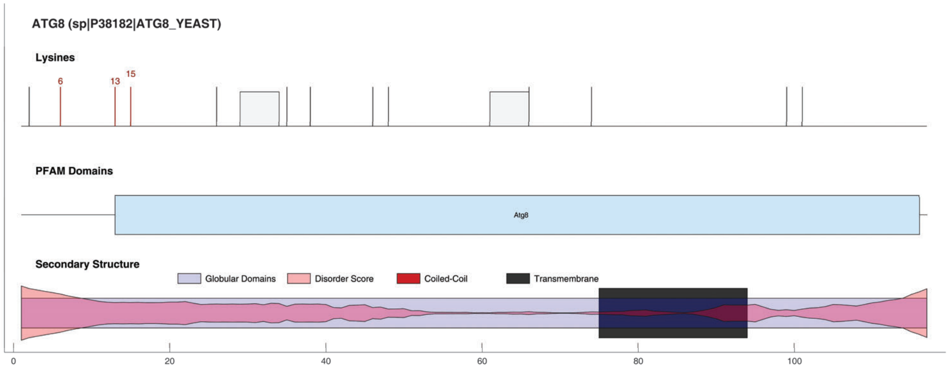
ATG8 (sp|P38182|ATG8_YEAST)
Lysines
6
13
15
PFAM Domains
Atg8
Secondary Structure
Globular Domains
Disorder Score
Coiled-Coil
Transmembrane
0
20
40
60
80
100

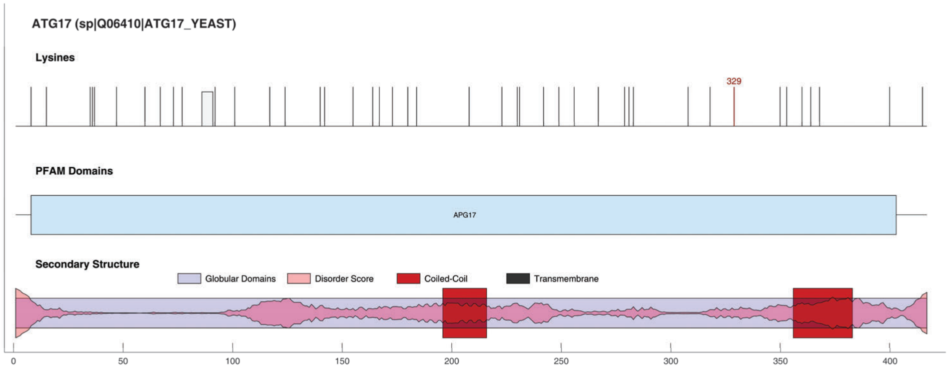
ATG17 (sp|Q06410|ATG17_YEAST)
Lysines
329
PFAM Domains
APG17
Secondary Structure
Globular Domains
Disorder Score
Coiled-Coil
Transmembrane
0
50
100
150
200
250
300
350
400

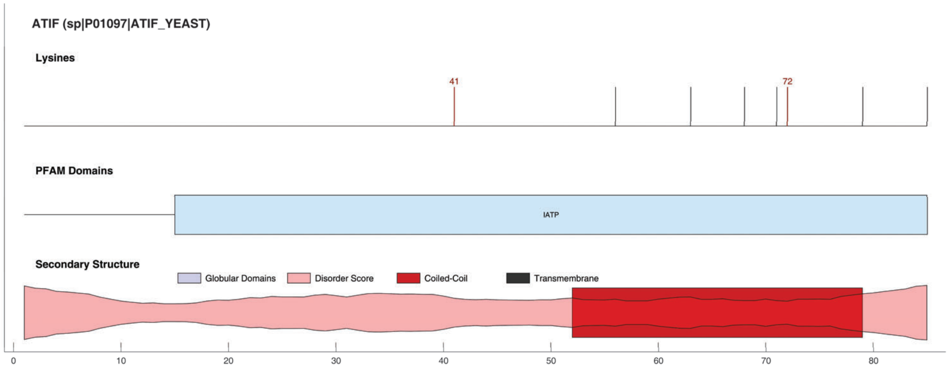
ATIF (sp|P01097|ATIF_YEAST)
Lysines
41
72
PFAM Domains
IATP
Secondary Structure
Globular Domains
Disorder Score
Coiled-Coil
Transmembrane
0
10
20
30
40
50
60
70
80

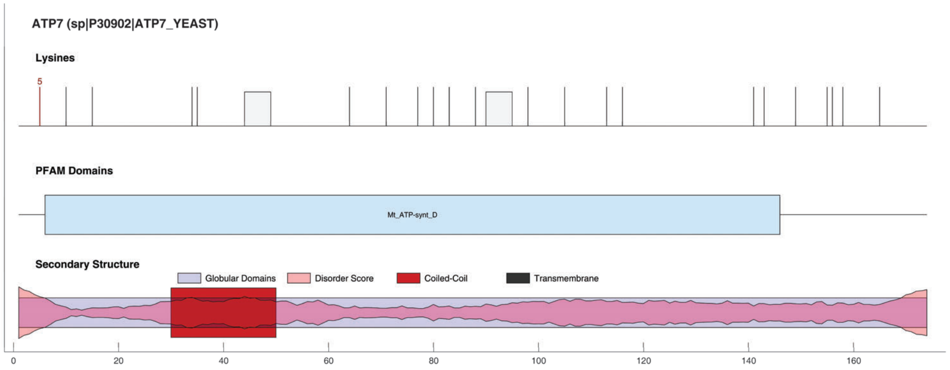
ATP7 (sp|P30902|ATP7_YEAST)
Lysines
5
PFAM Domains
Mt_ATP-synt_D
Secondary Structure
Globular Domains
Disorder Score
Coiled-Coil
Transmembrane
0
20
40
60
80
100
120
140
160

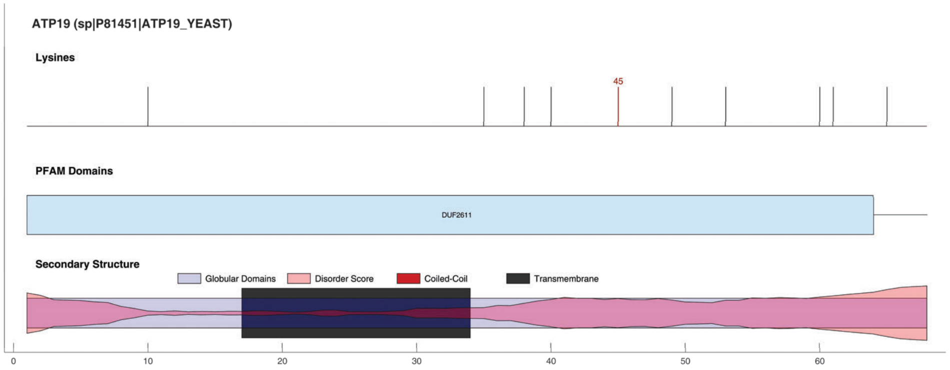
ATP19 (sp|P81451|ATP19_YEAST)
Lysines
45
PFAM Domains
DUF2611
Secondary Structure
Globular Domains
Disorder Score
Coiled-Coil
Transmembrane
0
10
20
30
40
50
60

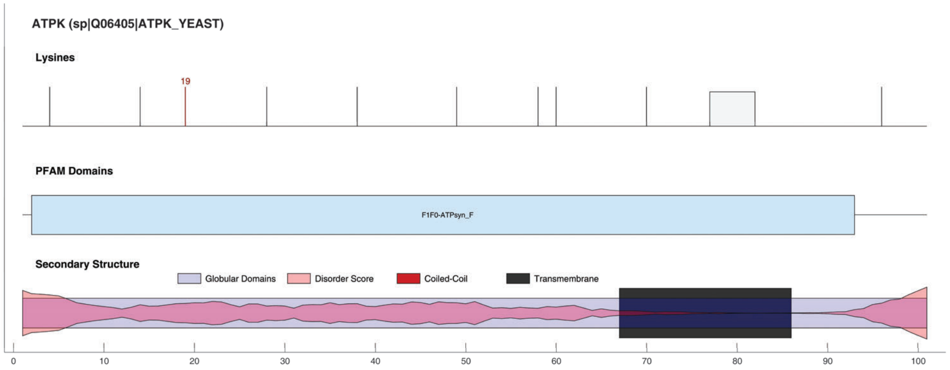
ATPK (sp|Q06405|ATPK_YEAST)
Lysines
19
PFAM Domains
F1F0-ATPsyn_F
Secondary Structure
Globular Domains
Disorder Score
Coiled-Coil
Transmembrane
0
10
20
30
40
50
60
70
80
90
100

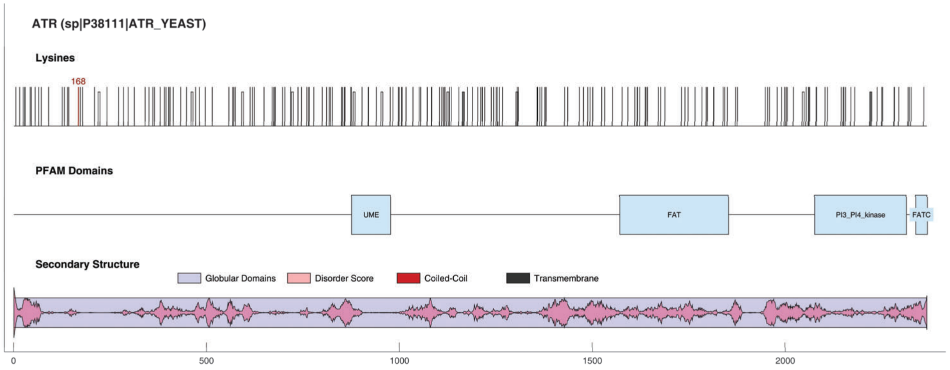
ATR (sp|P38111|ATR_YEAST)
Lysines
168
PFAM Domains
UME
FAT
PI3_PI4_kinase
FATC
Secondary Structure
Globular Domains
Disorder Score
Coiled-Coil
Transmembrane
0
500
1000
1500
2000

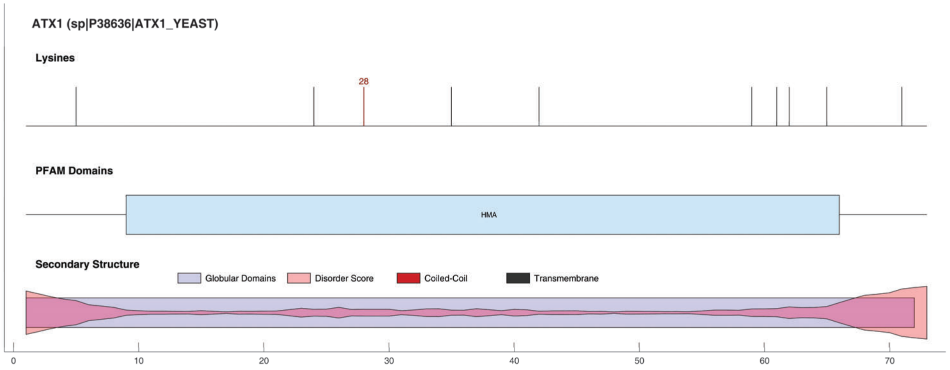
ATX1 (sp|P38636|ATX1_YEAST)
Lysines
28
PFAM Domains
HMA
Secondary Structure
Globular Domains
Disorder Score
Coiled-Coil
Transmembrane
0
10
20
30
40
50
60
70

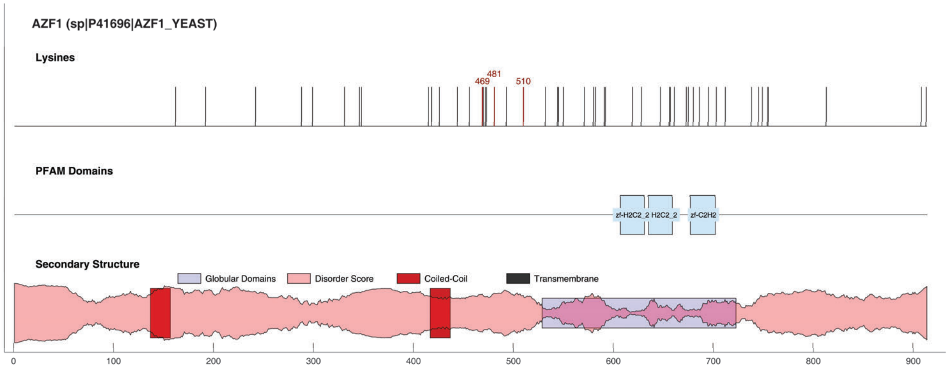
AZF1 (sp|P41696|AZF1_YEAST)
Lysines
469
481
510
PFAM Domains
zf-H2C2_2
H2C2_2
zf-C2H2
Secondary Structure
Globular Domains
Disorder Score
Coiled-Coil
Transmembrane
0
100
200
300
400
500
600
700
800
900

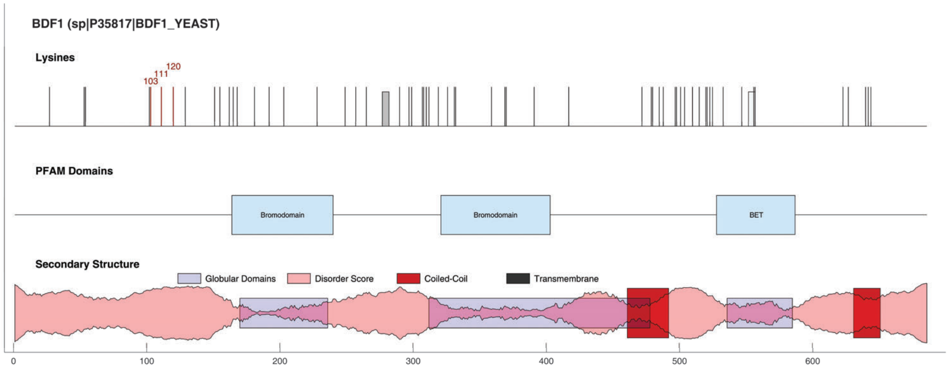
BDF1 (sp|P35817|BDF1_YEAST)
Lysines
103
111
120
PFAM Domains
Bromodomain
Bromodomain
BET
Secondary Structure
Globular Domains
Disorder Score
Coiled-Coil
Transmembrane
0
100
200
300
400
500
600

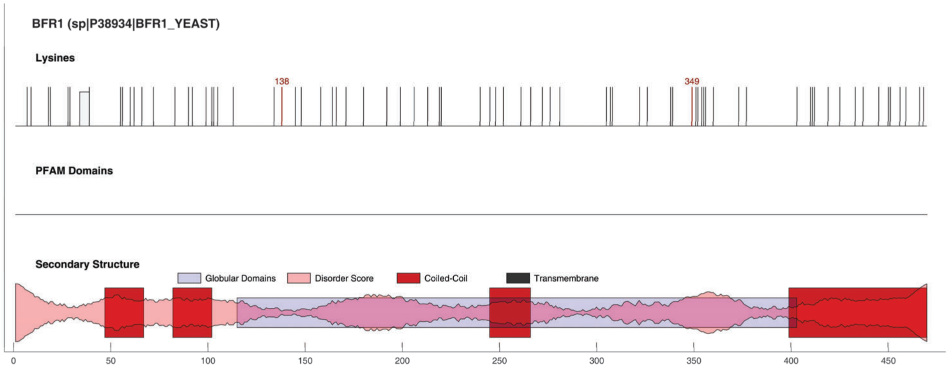
BFR1 (sp|P38934|BFR1_YEAST)
Lysines
138
349
PFAM Domains
Secondary Structure
Globular Domains
Disorder Score
Coiled-Coil
Transmembrane
0
50
100
150
200
250
300
350
400
450

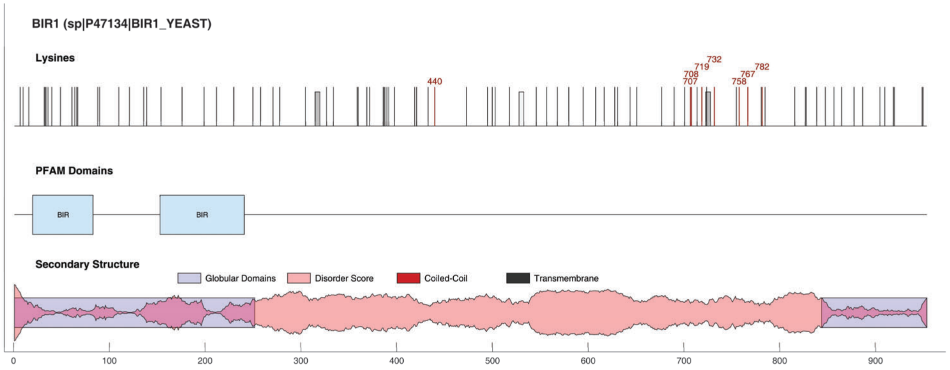
BIR1 (sp|P47134|BIR1_YEAST)
Lysines
440
707
708
719
732
758
767
782
PFAM Domains
BIR
BIR
Secondary Structure
Globular Domains
Disorder Score
Coiled-Coil
Transmembrane
0
100
200
300
400
500
600
700
800
900

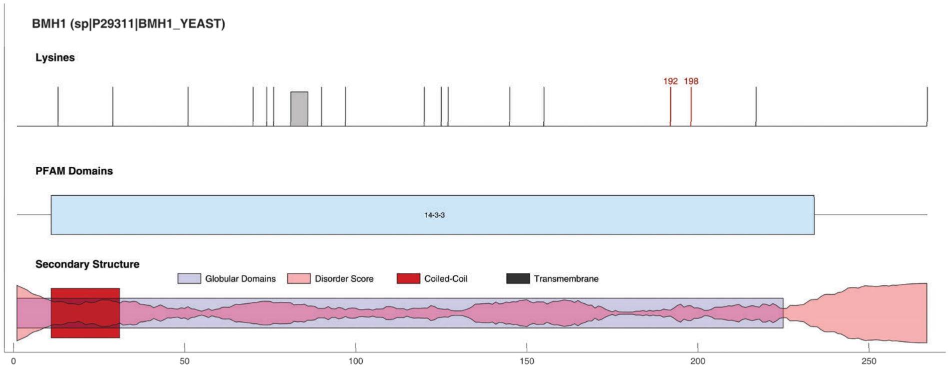
BMH1 (sp|P29311|BMH1_YEAST)
Lysines
192
198
PFAM Domains
14-3-3
Secondary Structure
Globular Domains
Disorder Score
Coiled-Coil
Transmembrane
0
50
100
150
200
250

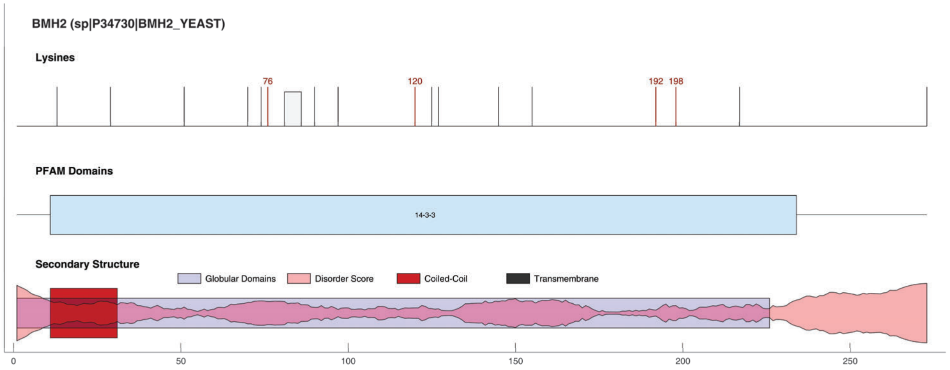
BMH2 (sp|P34730|BMH2_YEAST)
Lysines
76
120
192
198
PFAM Domains
14-3-3
Secondary Structure
Globular Domains
Disorder Score
Coiled-Coil
Transmembrane
0
50
100
150
200
250

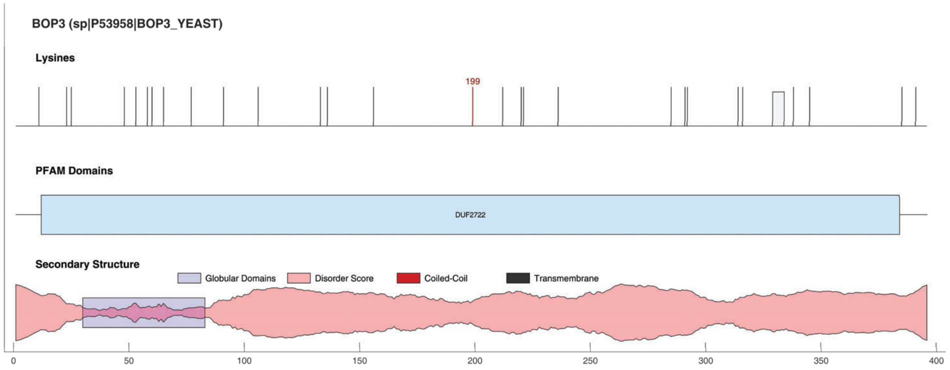
BOP3 (sp|P53958|BOP3_YEAST)
Lysines
199
PFAM Domains
DUF2722
Secondary Structure
Globular Domains
Disorder Score
Coiled-Coil
Transmembrane
0
50
100
150
200
250
300
350
400

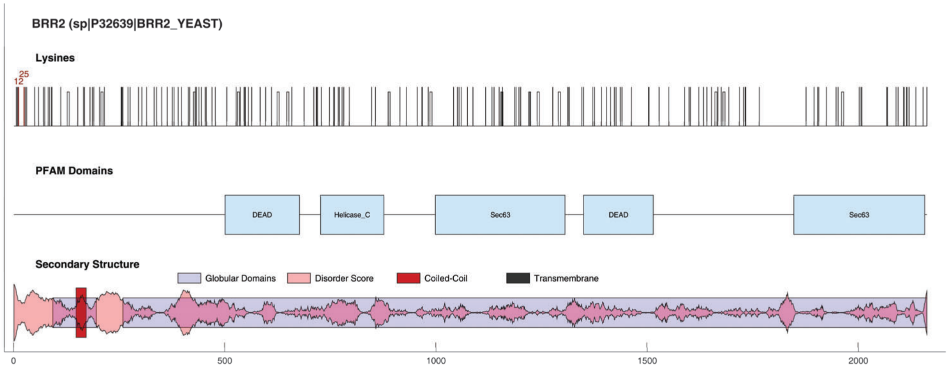
BRR2 (sp|P32639|BRR2_YEAST)
Lysines
25
12
PFAM Domains
DEAD
Helicase_C
Sec63
DEAD
Sec63
Secondary Structure
Globular Domains
Disorder Score
Coiled-Coil
Transmembrane
0
500
1000
1500
2000

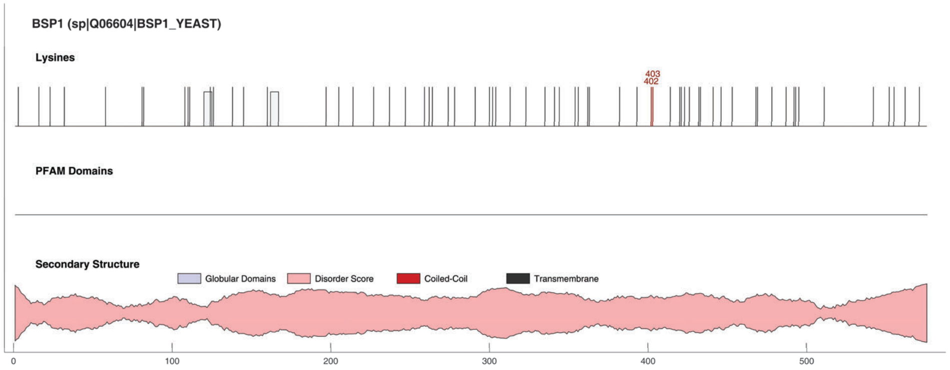
BSP1 (sp|Q06604|BSP1_YEAST)
Lysines
403
402
PFAM Domains
Secondary Structure
Globular Domains
Disorder Score
Coiled-Coil
Transmembrane
0
100
200
300
400
500

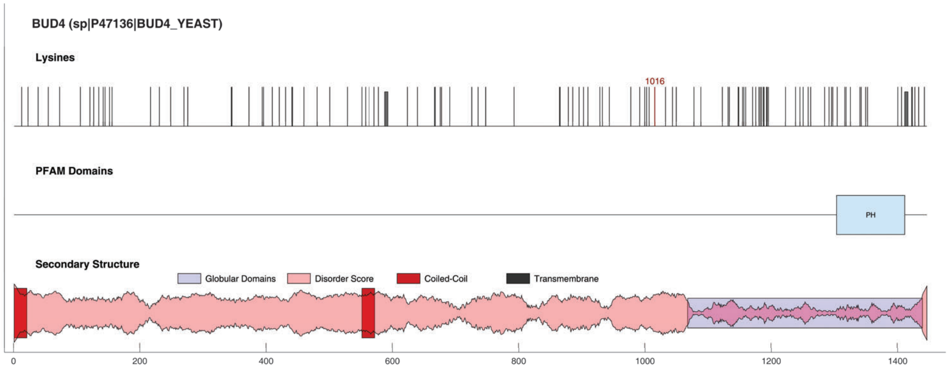
BUD4 (sp|P47136|BUD4_YEAST)
Lysines
1016
PFAM Domains
PH
Secondary Structure
Globular Domains
Disorder Score
Coiled-Coil
Transmembrane
0
200
400
600
800
1000
1200
1400

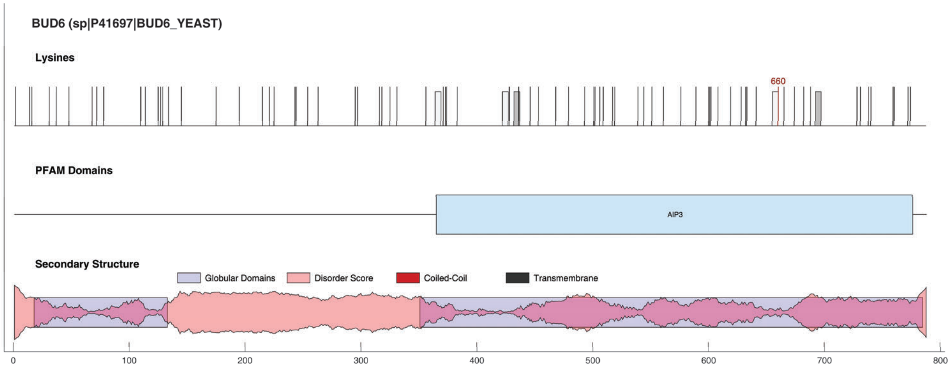

BUD6 (sp|P41697|BUD6_YEAST)
Lysines
660
PFAM Domains
AIP3
Secondary Structure
Globular Domains
Disorder Score
Coiled-Coil
Transmembrane
0
100
200
300
400
500
600
700
800

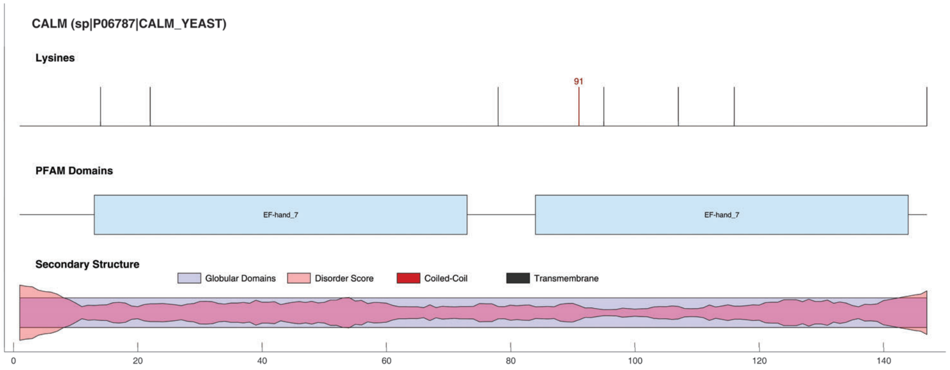

CALM (sp|P06787|CALM_YEAST)
Lysines
91
PFAM Domains
EF-hand_7
EF-hand_7
Secondary Structure
Globular Domains
Disorder Score
Coiled-Coil
Transmembrane
0
20
40
60
80
100
120
140

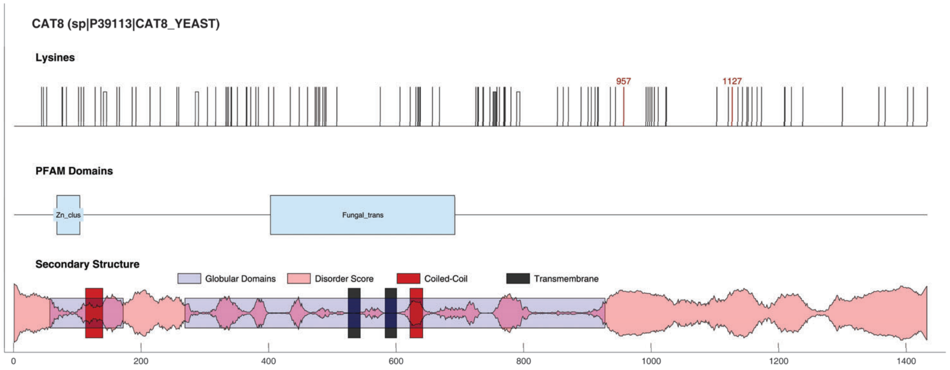

CAT8 (sp|P39113|CAT8_YEAST)
Lysines
957
1127
PFAM Domains
Zn_clus
Fungal_trans
Secondary Structure
Globular Domains
Disorder Score
Coiled-Coil
Transmembrane
0
200
400
600
800
1000
1200
1400

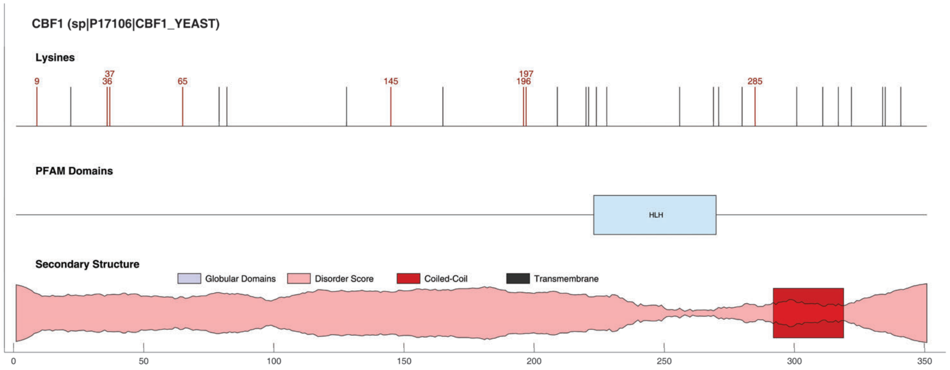

CBF1 (sp|P17106|CBF1_YEAST)
Lysines
9
37
36
65
145
197
196
285
PFAM Domains
HLH
Secondary Structure
Globular Domains
Disorder Score
Coiled-Coil
Transmembrane
0
50
100
150
200
250
300
350

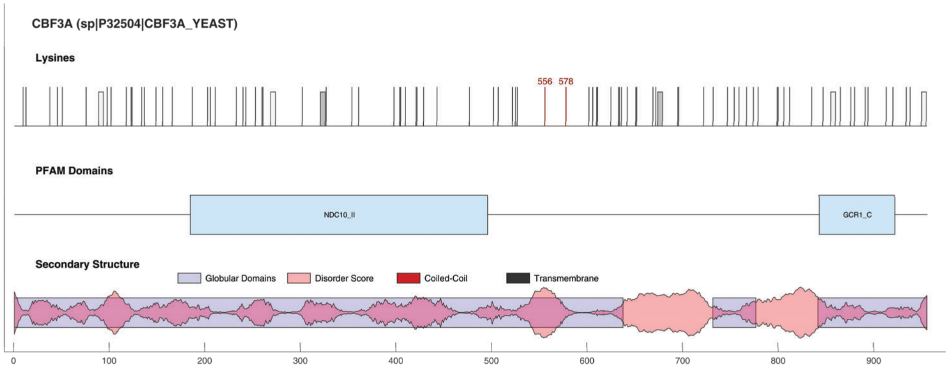
CBF3A (sp|P32504|CBF3A_YEAST)
Lysines
556
578
PFAM Domains
NDC10_II
GCR1_C
Secondary Structure
Globular Domains
Disorder Score
Coiled-Coil
Transmembrane
0
100
200
300
400
500
600
700
800
900

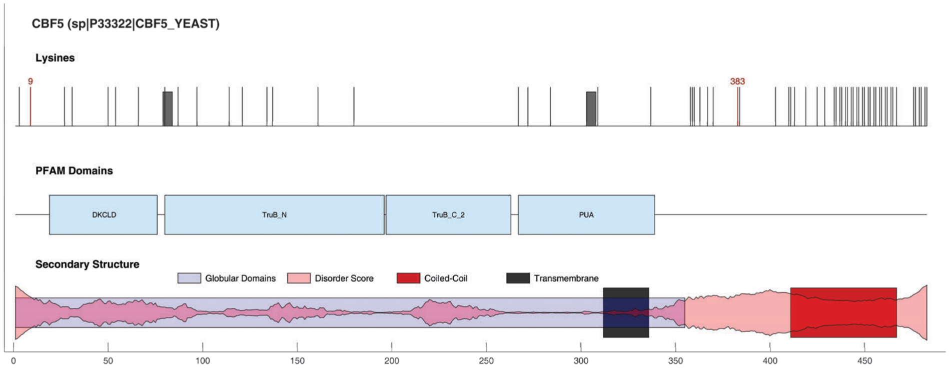
CBF5 (sp|P33322|CBF5_YEAST)
Lysines
9
383
PFAM Domains
DKCLD
TruB_N
TruB_C_2
PUA
Secondary Structure
Globular Domains
Disorder Score
Coiled-Coil
Transmembrane
0
50
100
150
200
250
300
350
400
450

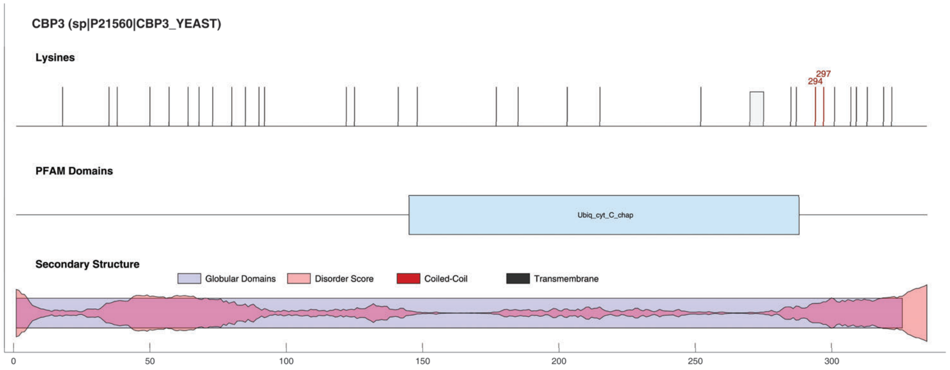
CBP3 (sp|P21560|CBP3_YEAST)
Lysines
294
297
PFAM Domains
Ubiq_cyt_C_chap
Secondary Structure
Globular Domains
Disorder Score
Coiled-Coil
Transmembrane
0
50
100
150
200
250
300

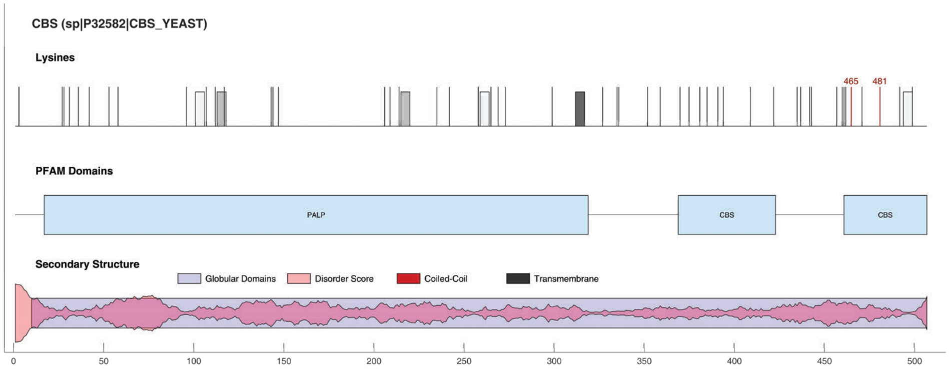
CBS (sp|P32582|CBS_YEAST)
Lysines
465
481
PFAM Domains
PALP
CBS
CBS
Secondary Structure
Globular Domains
Disorder Score
Coiled-Coil
Transmembrane
0
50
100
150
200
250
300
350
400
450
500

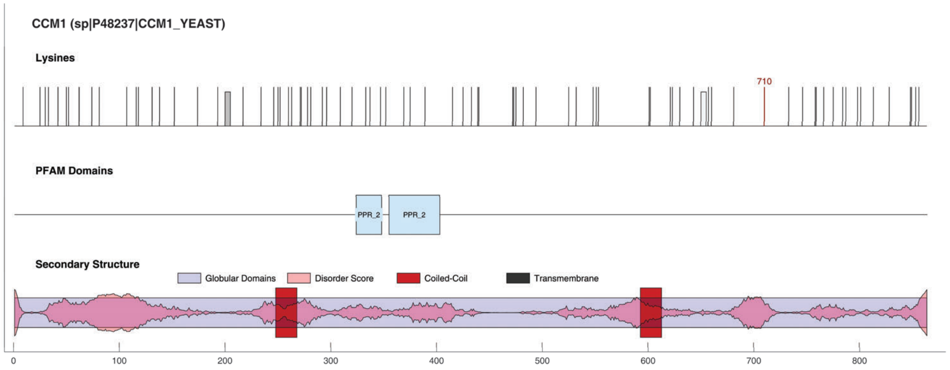
CCM1 (sp|P48237|CCM1_YEAST)
Lysines
710
PFAM Domains
PPR_2
PPR_2
Secondary Structure
Globular Domains
Disorder Score
Coiled-Coil
Transmembrane
0
100
200
300
400
500
600
700
800

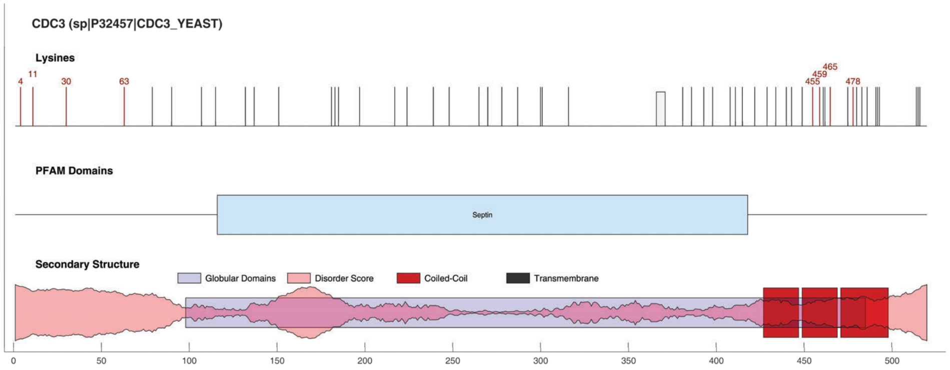
CDC3 (sp|P32457|CDC3_YEAST)
Lysines
4
11
30
63
455
459
465
478
PFAM Domains
Septin
Secondary Structure
Globular Domains
Disorder Score
Coiled-Coil
Transmembrane
0
50
100
150
200
250
300
350
400
450
500

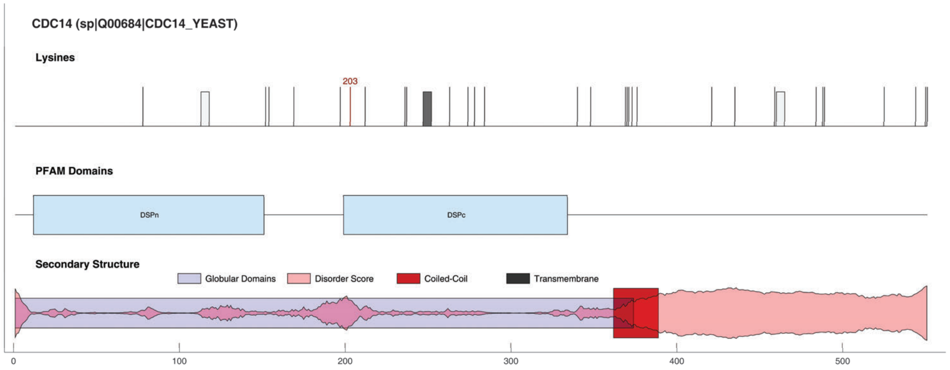
CDC14 (sp|Q00684|CDC14_YEAST)
Lysines
203
PFAM Domains
DSPn
DSPc
Secondary Structure
Globular Domains
Disorder Score
Coiled-Coil
Transmembrane
0
100
200
300
400
500

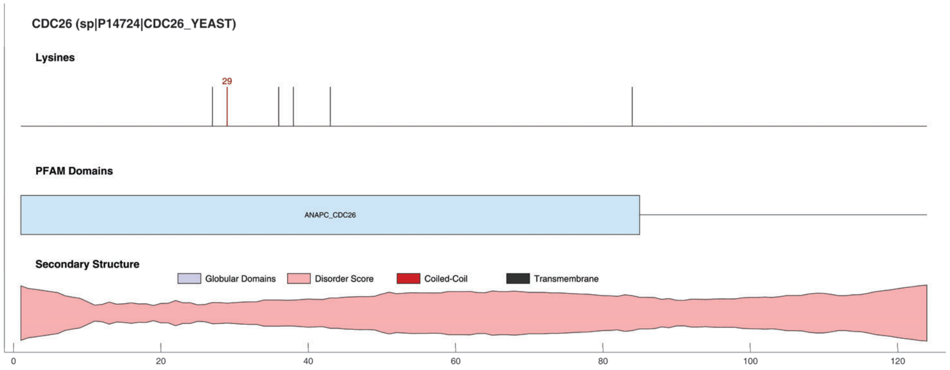
CDC26 (sp|P14724|CDC26_YEAST)
Lysines
29
PFAM Domains
ANAPC_CDC26
Secondary Structure
Globular Domains
Disorder Score
Coiled-Coil
Transmembrane
0
20
40
60
80
100
120

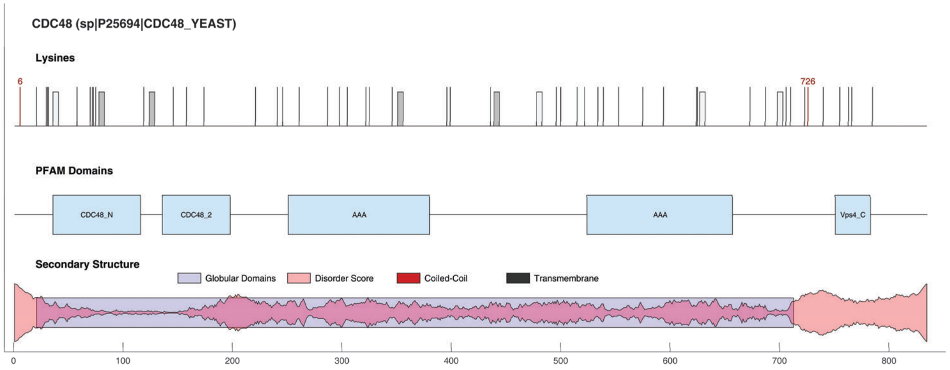
CDC48 (sp|P25694|CDC48_YEAST)
Lysines
6
726
PFAM Domains
CDC48_N
CDC48_2
AAA
AAA
Vps4_C
Secondary Structure
Globular Domains
Disorder Score
Coiled-Coil
Transmembrane
0
100
200
300
400
500
600
700
800

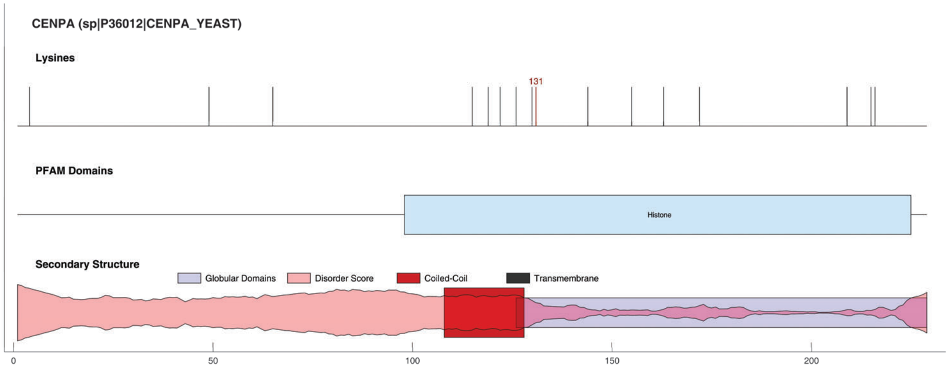
CENPA (sp|P36012|CENPA_YEAST)
Lysines
131
PFAM Domains
Histone
Secondary Structure
Globular Domains
Disorder Score
Coiled-Coil
Transmembrane
0
50
100
150
200

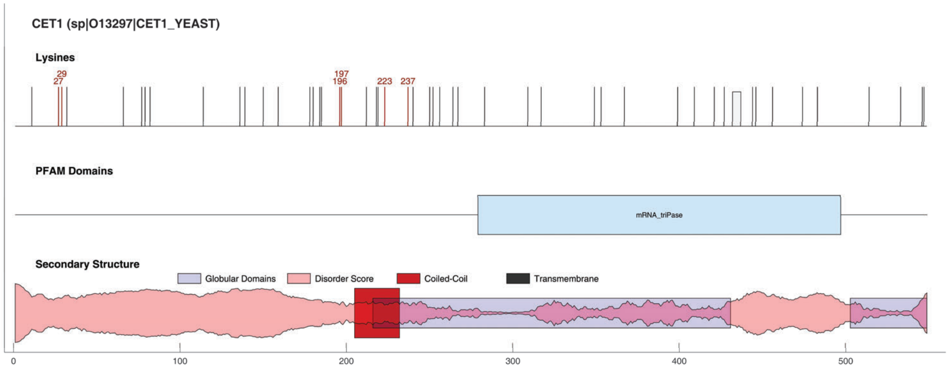
CET1 (sp|O13297|CET1_YEAST)
Lysines
29
27
197
196
223
237
PFAM Domains
mRNA_triPase
Secondary Structure
Globular Domains
Disorder Score
Coiled-Coil
Transmembrane
0
100
200
300
400
500

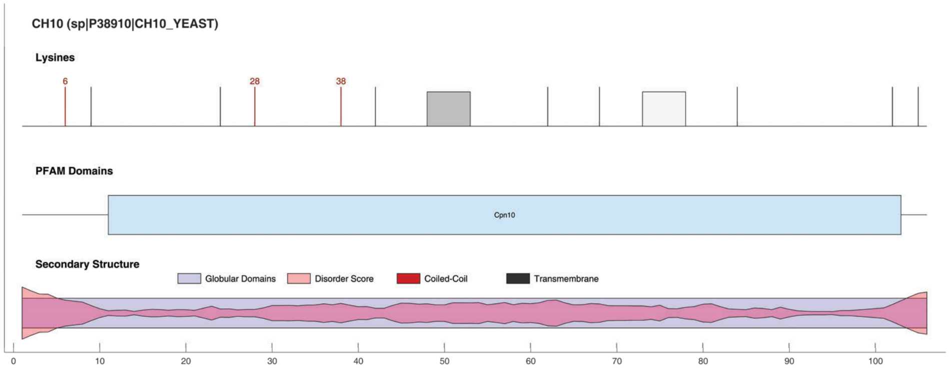
CH10 (sp|P38910|CH10_YEAST)
Lysines
6
28
38
PFAM Domains
Cpn10
Secondary Structure
Globular Domains
Disorder Score
Coiled-Coil
Transmembrane
0
10
20
30
40
50
60
70
80
90
100

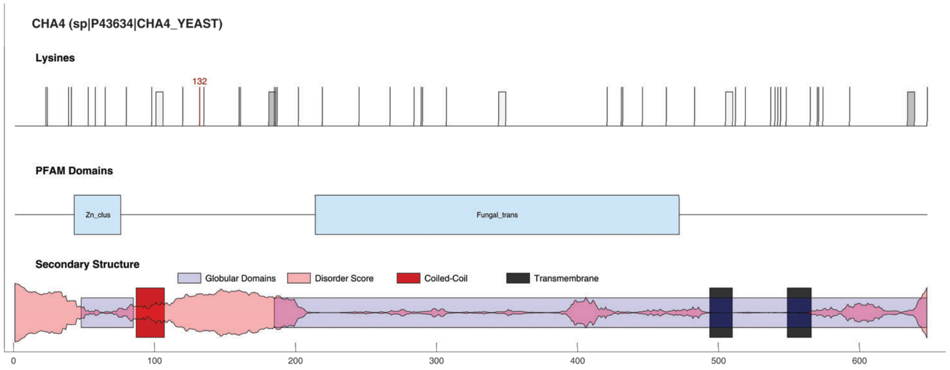
CHA4 (sp|P43634|CHA4_YEAST)
Lysines
132
PFAM Domains
Zn_clus
Fungal_trans
Secondary Structure
Globular Domains
Disorder Score
Coiled-Coil
Transmembrane
0
100
200
300
400
500
600

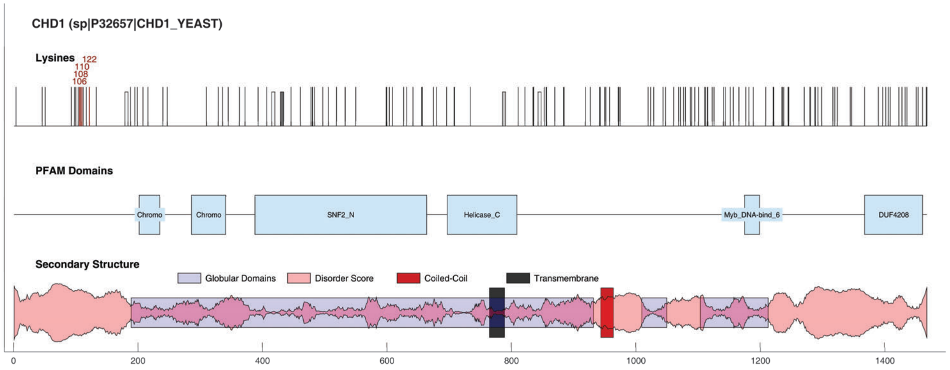
CHD1 (sp|P32657|CHD1_YEAST)
Lysines
122
110
108
106
PFAM Domains
Chromo
Chromo
SNF2_N
Helicase_C
Myb_DNA-bind_6
DUF4208
Secondary Structure
Globular Domains
Disorder Score
Coiled-Coil
Transmembrane
0
200
400
600
800
1000
1200
1400

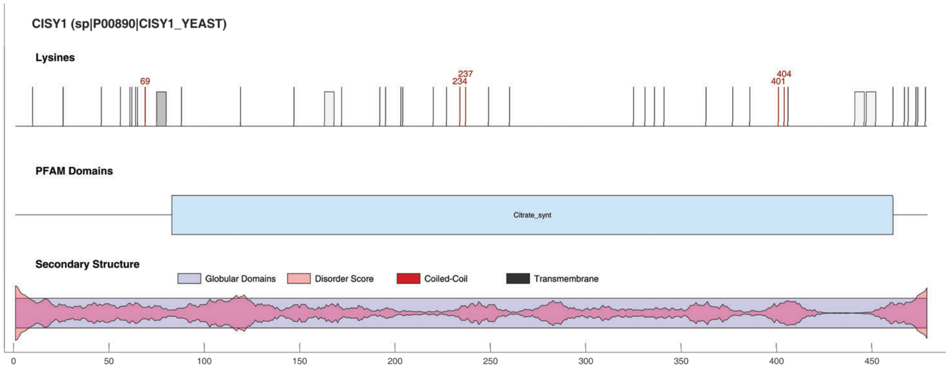
CISY1 (sp|P00890|CISY1_YEAST)
Lysines
69
237
234
404
401
PFAM Domains
Citrate_synt
Secondary Structure
Globular Domains
Disorder Score
Coiled-Coil
Transmembrane
0
50
100
150
200
250
300
350
400
450

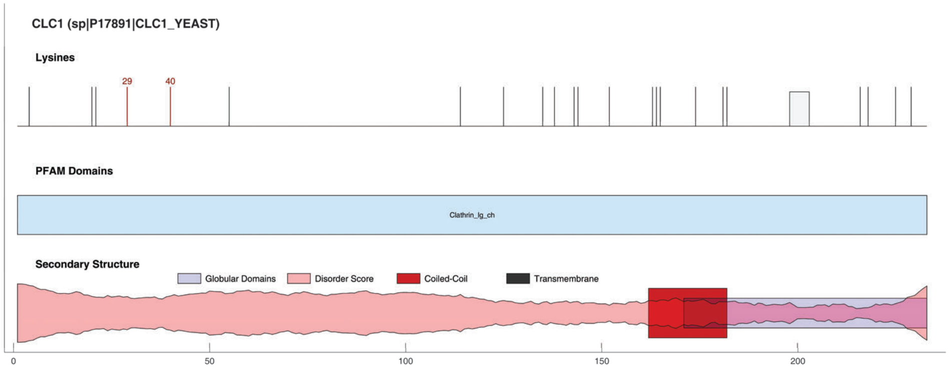
CLC1 (sp|P17891|CLC1_YEAST)
Lysines
29
40
PFAM Domains
Clathrin_lg_ch
Secondary Structure
Globular Domains
Disorder Score
Coiled-Coil
Transmembrane
0
50
100
150
200

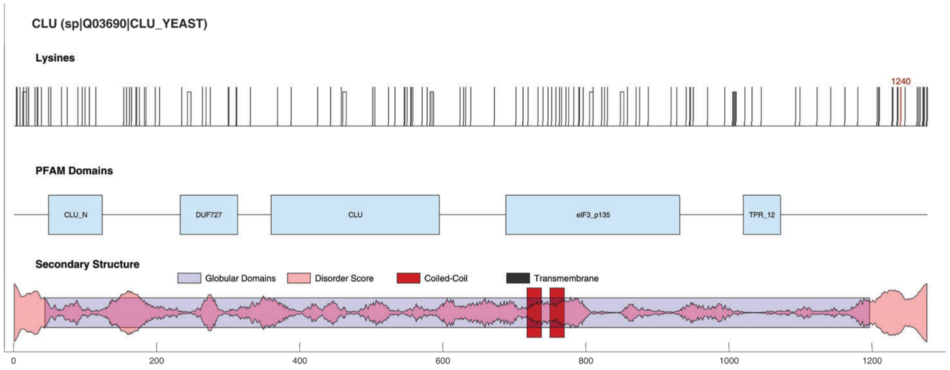
CLU (sp|Q03690|CLU_YEAST)
Lysines
1240
PFAM Domains
CLU_N
DUF727
CLU
eIF3_p135
TPR_12
Secondary Structure
Globular Domains
Disorder Score
Coiled-Coil
Transmembrane
0
200
400
600
800
1000
1200

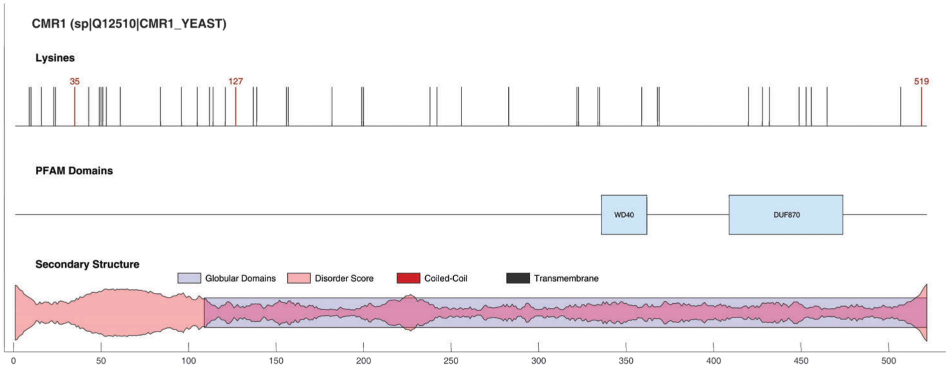
CMR1 (sp|Q12510|CMR1_YEAST)
Lysines
35
127
519
PFAM Domains
WD40
DUF870
Secondary Structure
Globular Domains
Disorder Score
Coiled-Coil
Transmembrane
0
50
100
150
200
250
300
350
400
450
500

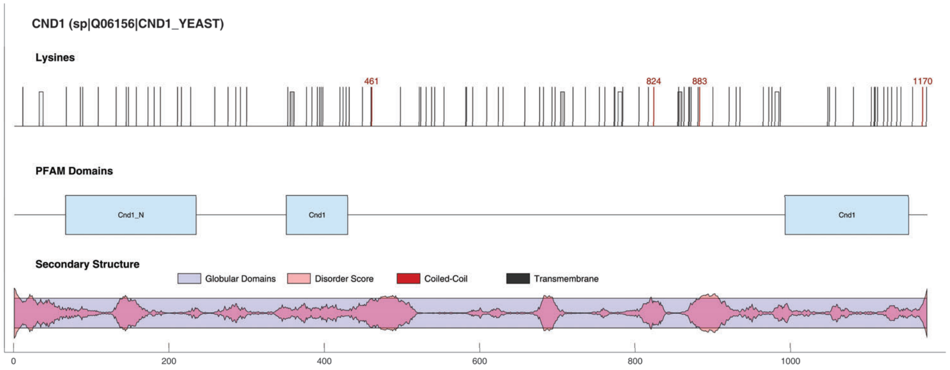
CND1 (sp|Q06156|CND1_YEAST)
Lysines
461
824
883
1170
PFAM Domains
Cnd1_N
Cnd1
Cnd1
Secondary Structure
Globular Domains
Disorder Score
Coiled-Coil
Transmembrane
0
200
400
600
800
1000

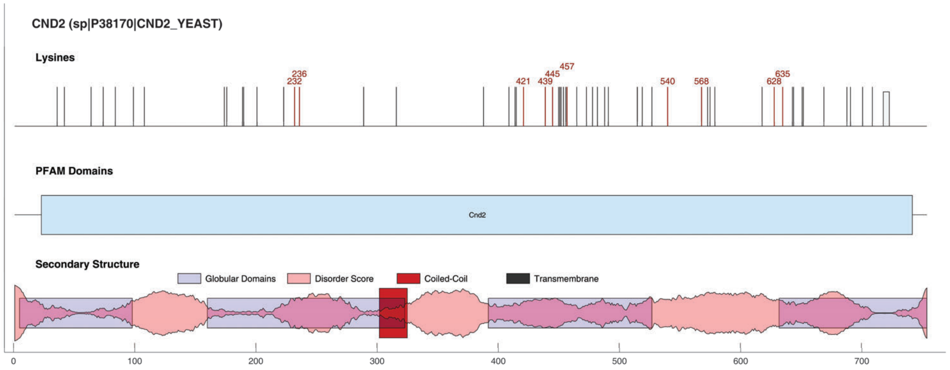
CND2 (sp|P38170|CND2_YEAST)
Lysines
236
232
421
439
445
457
540
568
635
628
PFAM Domains
Cnd2
Secondary Structure
Globular Domains
Disorder Score
Coiled-Coil
Transmembrane
0
100
200
300
400
500
600
700

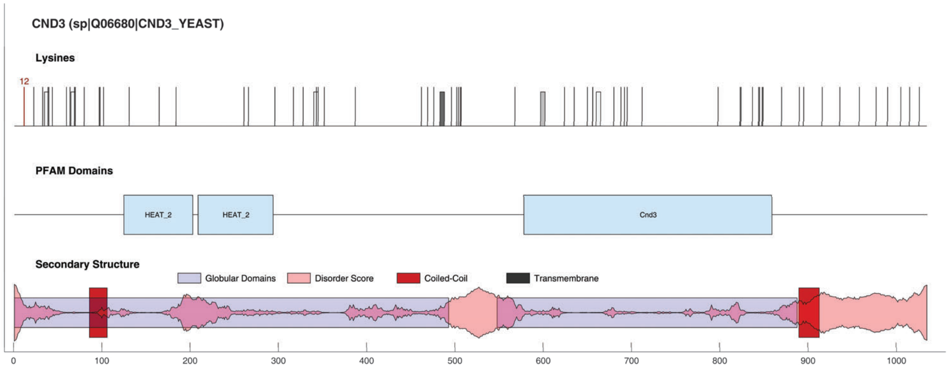

CND3 (sp|Q06680|CND3_YEAST)
Lysines
12
PFAM Domains
HEAT_2
HEAT_2
Cnd3
Secondary Structure
Globular Domains
Disorder Score
Coiled-Coil
Transmembrane
0
100
200
300
400
500
600
700
800
900
1000

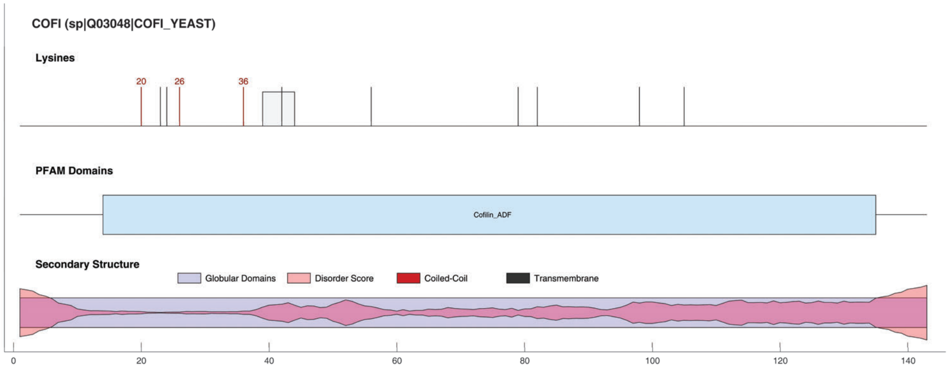

COFI (sp|Q03048|COFI_YEAST)
Lysines
20
26
36
PFAM Domains
Cofilin_ADF
Secondary Structure
Globular Domains
Disorder Score
Coiled-Coil
Transmembrane
0
20
40
60
80
100
120
140

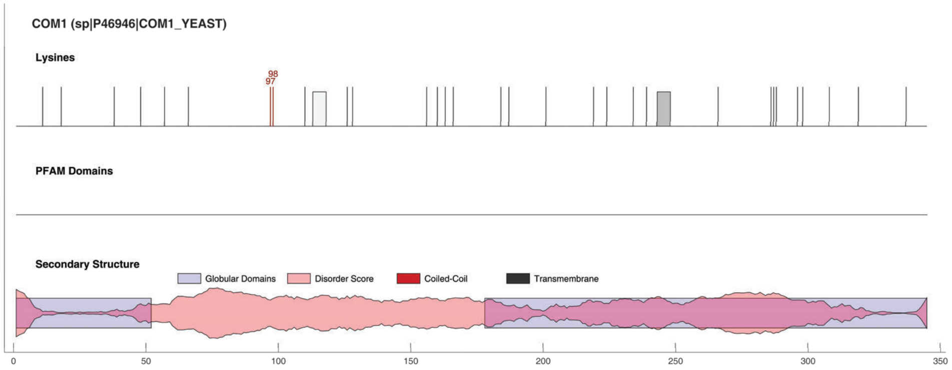

COM1 (sp|P46946|COM1_YEAST)
Lysines
98
97
PFAM Domains
Secondary Structure
Globular Domains
Disorder Score
Coiled-Coil
Transmembrane
0
50
100
150
200
250
300
350

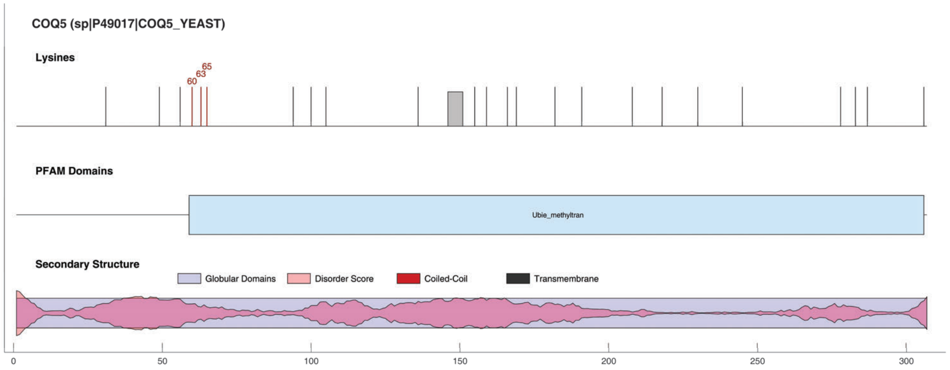

COQ5 (sp|P49017|COQ5_YEAST)
Lysines
65
63
60
PFAM Domains
Ubie_methyltran
Secondary Structure
Globular Domains
Disorder Score
Coiled-Coil
Transmembrane
0
50
100
150
200
250
300

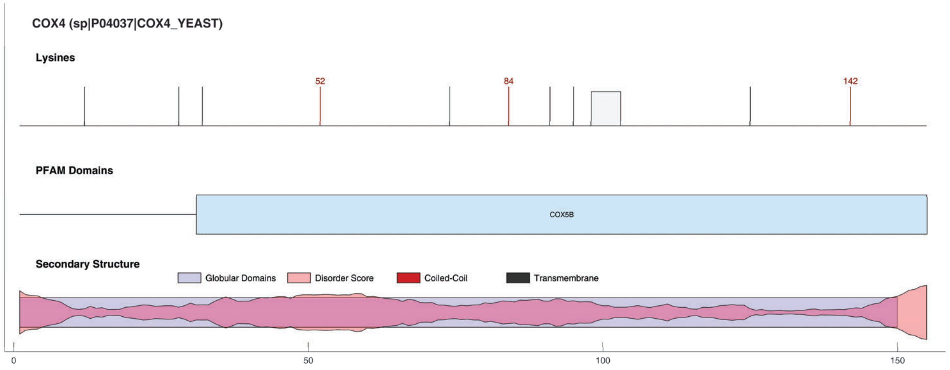
COX4 (sp|P04037|COX4_YEAST)
Lysines
52
84
142
PFAM Domains
COX5B
Secondary Structure
Globular Domains
Disorder Score
Coiled-Coil
Transmembrane
0
50
100
150

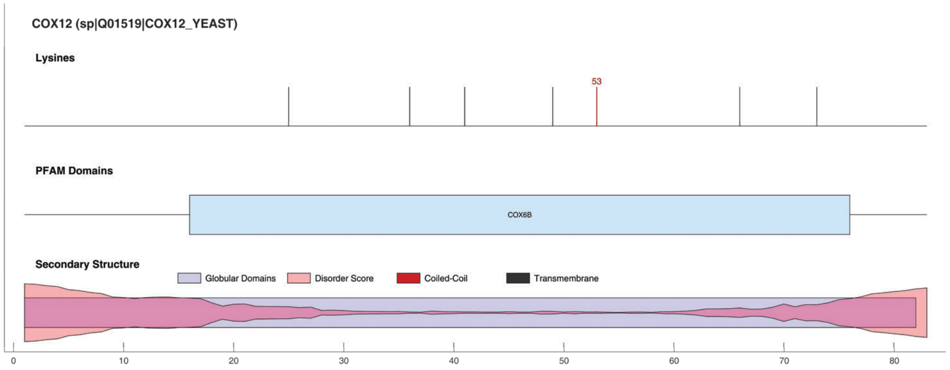
COX12 (sp|Q01519|COX12_YEAST)
Lysines
53
PFAM Domains
COX6B
Secondary Structure
Globular Domains
Disorder Score
Coiled-Coil
Transmembrane
0
10
20
30
40
50
60
70
80

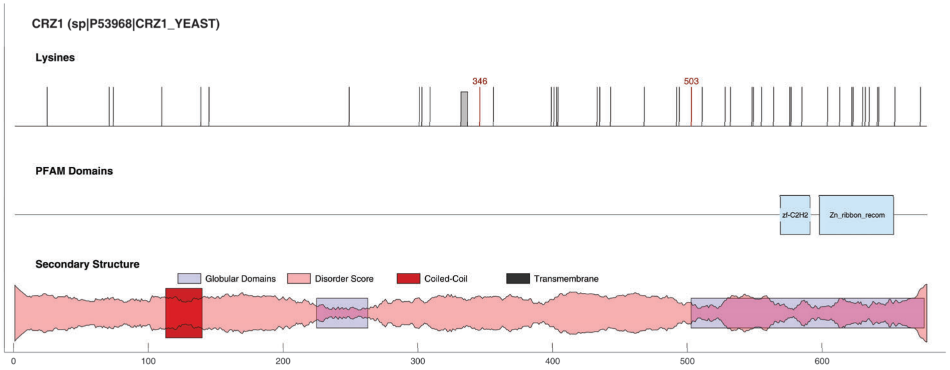
CRZ1 (sp|P53968|CRZ1_YEAST)
Lysines
346
503
PFAM Domains
zf-C2H2
Zn_ribbon_recom
Secondary Structure
Globular Domains
Disorder Score
Coiled-Coil
Transmembrane
0
100
200
300
400
500
600

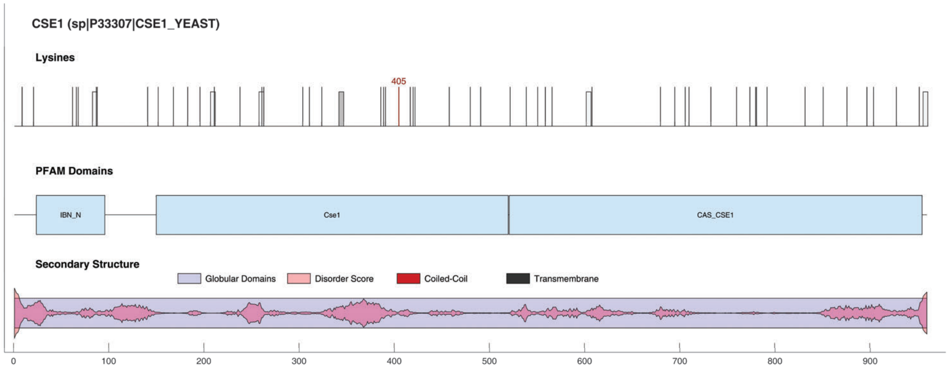
CSE1 (sp|P33307|CSE1_YEAST)
Lysines
405
PFAM Domains
IBN_N
Cse1
CAS_CSE1
Secondary Structure
Globular Domains
Disorder Score
Coiled-Coil
Transmembrane
0
100
200
300
400
500
600
700
800
900

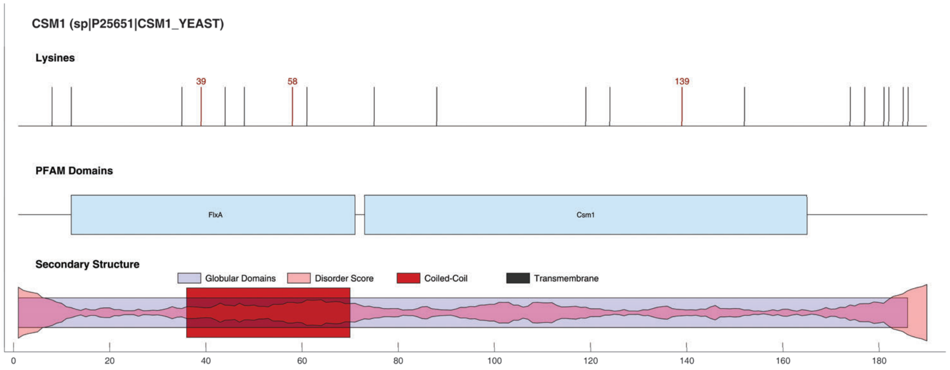
CSM1 (sp|P25651|CSM1_YEAST)
Lysines
39
58
139
PFAM Domains
FlxA
Csm1
Secondary Structure
Globular Domains
Disorder Score
Coiled-Coil
Transmembrane
0
20
40
60
80
100
120
140
160
180

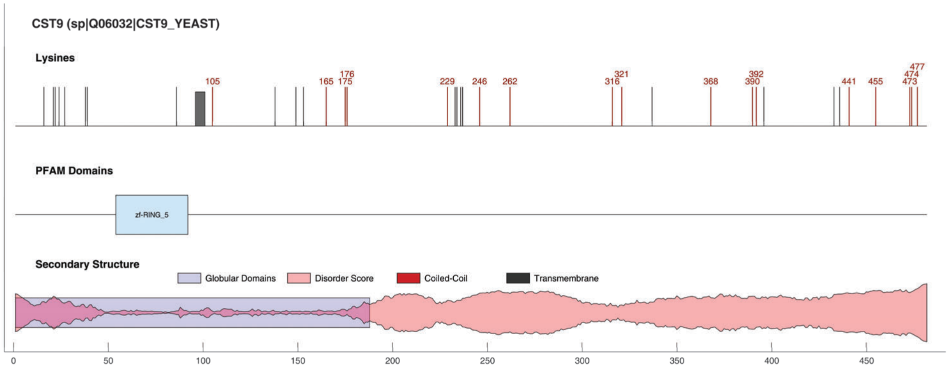
CST9 (sp|Q06032|CST9_YEAST)
Lysines
105
165 175
176
229
246
262
316
321
368
390
392
441
455
473
474
477
PFAM Domains
zf-RING_5
Secondary Structure
Globular Domains
Disorder Score
Coiled-Coil
Transmembrane
0
50
100
150
200
250
300
350
400
450

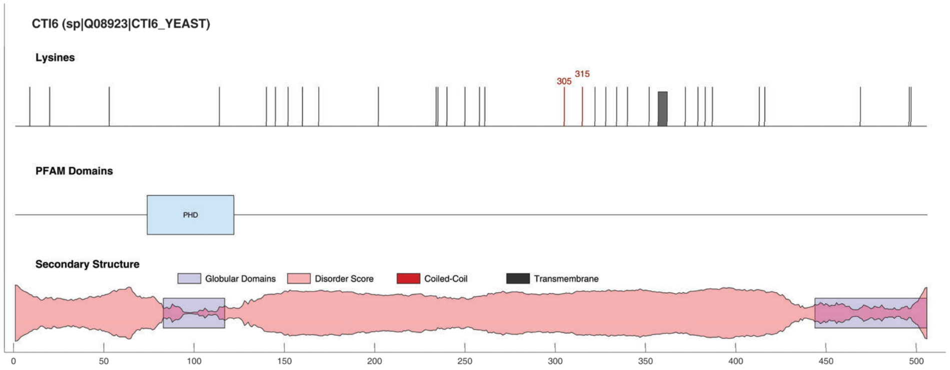
CTI6 (sp|Q08923|CTI6_YEAST)
Lysines
305
315
PFAM Domains
PHD
Secondary Structure
Globular Domains
Disorder Score
Coiled-Coil
Transmembrane
0
50
100
150
200
250
300
350
400
450
500

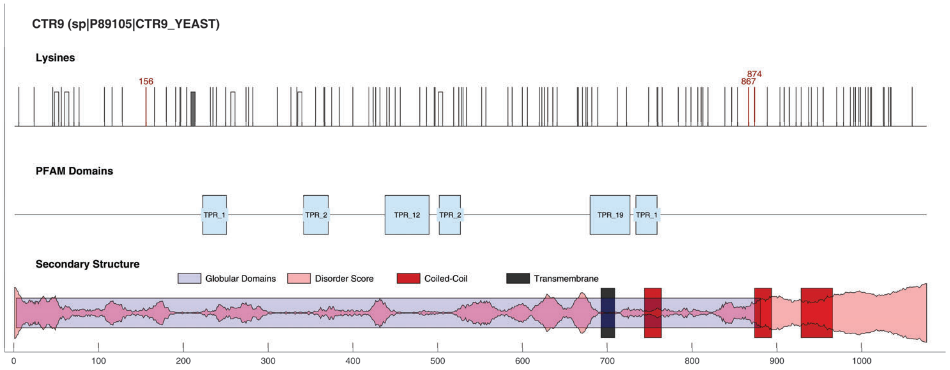
CTR9 (sp|P89105|CTR9_YEAST)
Lysines
156
867
874
PFAM Domains
TPR_1
TPR_2
TPR_12
TPR_2
TPR_19
TPR_1
Secondary Structure
Globular Domains
Disorder Score
Coiled-Coil
Transmembrane
0
100
200
300
400
500
600
700
800
900
1000

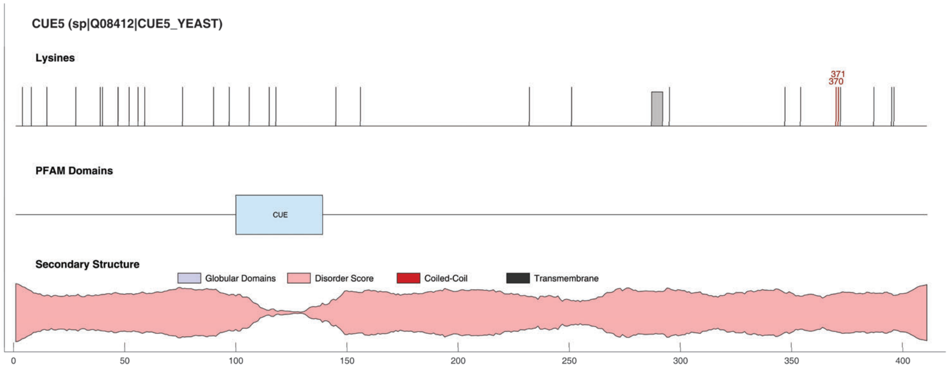
CUE5 (sp|Q08412|CUE5_YEAST)
Lysines
371
370
PFAM Domains
CUE
Secondary Structure
Globular Domains
Disorder Score
Coiled-Coil
Transmembrane
0
50
100
150
200
250
300
350
400

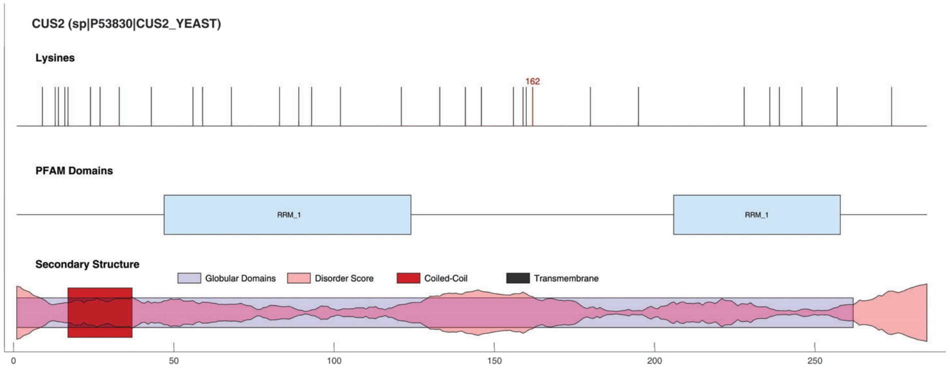
CUS2 (sp|P53830|CUS2_YEAST)
Lysines
162
PFAM Domains
RRM_1
RRM_1
Secondary Structure
Globular Domains
Disorder Score
Coiled-Coil
Transmembrane
0
50
100
150
200
250

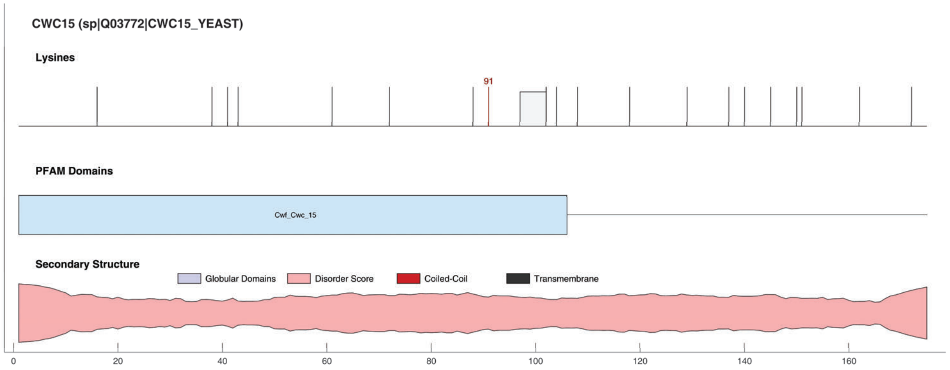
CWC15 (sp|Q03772|CWC15_YEAST)
Lysines
91
PFAM Domains
Cwf_Cwc_15
Secondary Structure
Globular Domains
Disorder Score
Coiled-Coil
Transmembrane
0
20
40
60
80
100
120
140
160

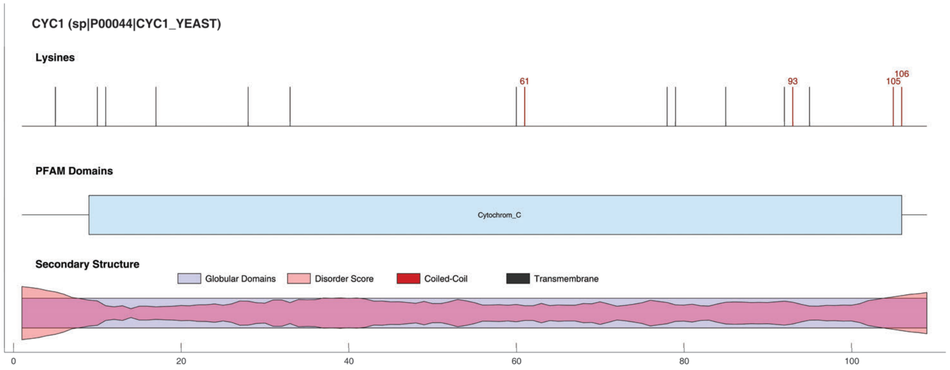
CYC1 (sp|P00044|CYC1_YEAST)
Lysines
61
93
105
106
PFAM Domains
Cytochrom_C
Secondary Structure
Globular Domains
Disorder Score
Coiled-Coil
Transmembrane
0
20
40
60
80
100

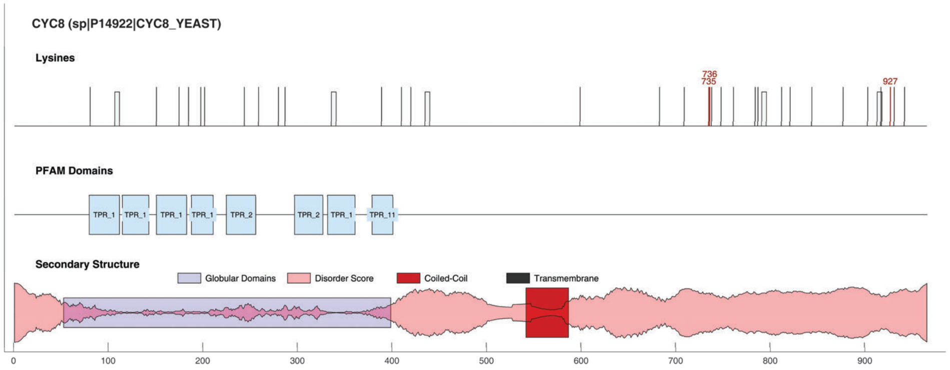

CYC8 (sp|P14922|CYC8_YEAST)
Lysines
736
735
927
PFAM Domains
TPR_1
TPR_1
TPR_1
TPR_1
TPR_2
TPR_2
TPR_1
TPR_11
Secondary Structure
Globular Domains
Disorder Score
Coiled-Coil
Transmembrane
0
100
200
300
400
500
600
700
800
900

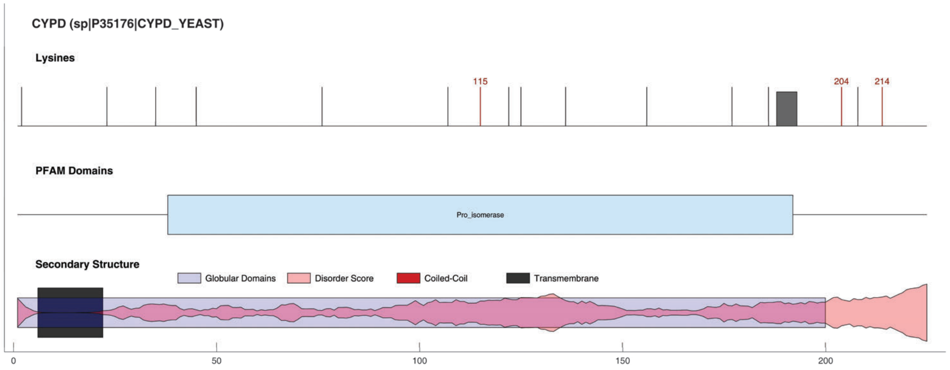

CYPD (sp|P35176|CYPD_YEAST)
Lysines
115
204
214
PFAM Domains
Pro_isomerase
Secondary Structure
Globular Domains
Disorder Score
Coiled-Coil
Transmembrane
0
50
100
150
200

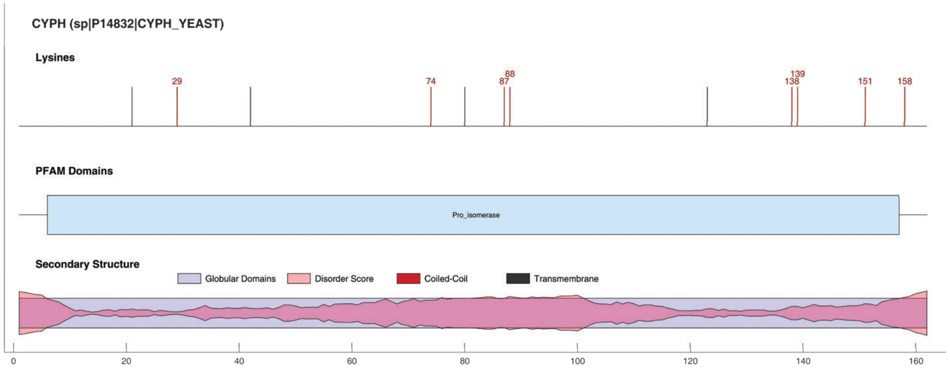

CYPH (sp|P14832|CYPH_YEAST)
Lysines
29
74
88
87
139
138
151
158
PFAM Domains
Pro_isomerase
Secondary Structure
Globular Domains
Disorder Score
Coiled-Coil
Transmembrane
0
20
40
60
80
100
120
140
160

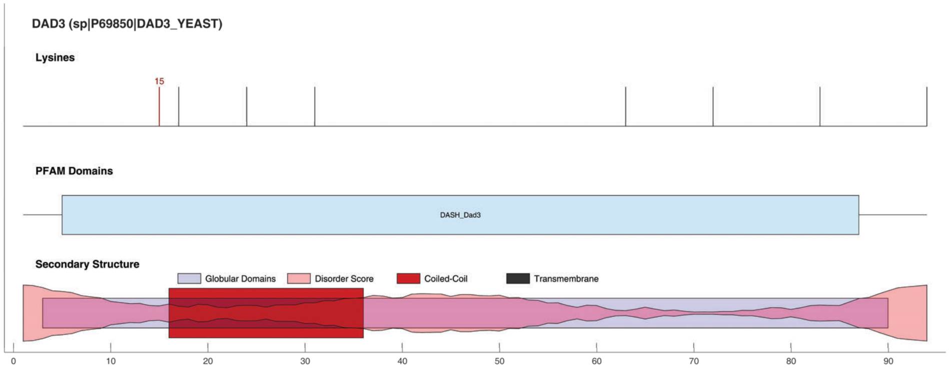

DAD3 (sp|P69850|DAD3_YEAST)
Lysines
15
PFAM Domains
DASH_Dad3
Secondary Structure
Globular Domains
Disorder Score
Coiled-Coil
Transmembrane
0
10
20
30
40
50
60
70
80
90

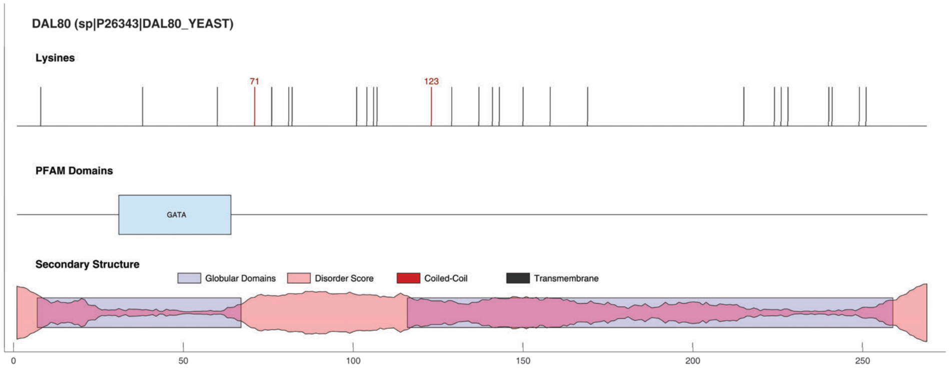
DAL80 (sp|P26343|DAL80_YEAST)
Lysines
71
123
PFAM Domains
GATA
Secondary Structure
Globular Domains
Disorder Score
Coiled-Coil
Transmembrane
0
50
100
150
200
250

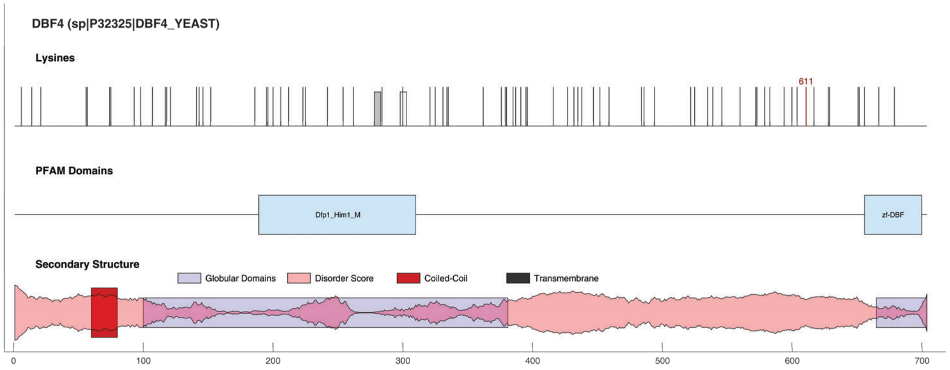
DBF4 (sp|P32325|DBF4_YEAST)
Lysines
611
PFAM Domains
Dfp1_Him1_M
zf-DBF
Secondary Structure
Globular Domains
Disorder Score
Coiled-Coil
Transmembrane
0
100
200
300
400
500
600
700

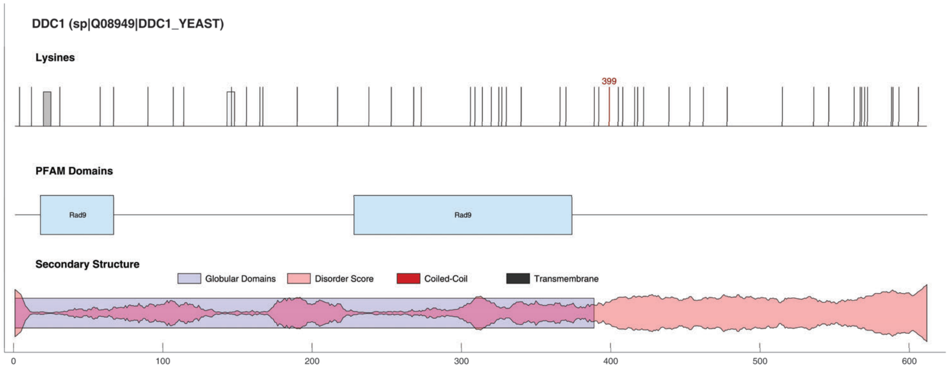
DDC1 (sp|Q08949|DDC1_YEAST)
Lysines
399
PFAM Domains
Rad9
Rad9
Secondary Structure
Globular Domains
Disorder Score
Coiled-Coil
Transmembrane
0
100
200
300
400
500
600

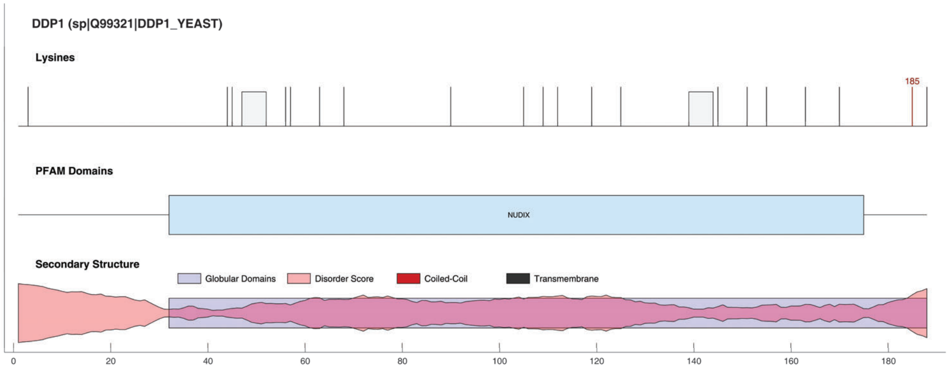
DDP1 (sp|Q99321|DDP1_YEAST)
Lysines
185
PFAM Domains
NUDIX
Secondary Structure
Globular Domains
Disorder Score
Coiled-Coil
Transmembrane
0
20
40
60
80
100
120
140
160
180

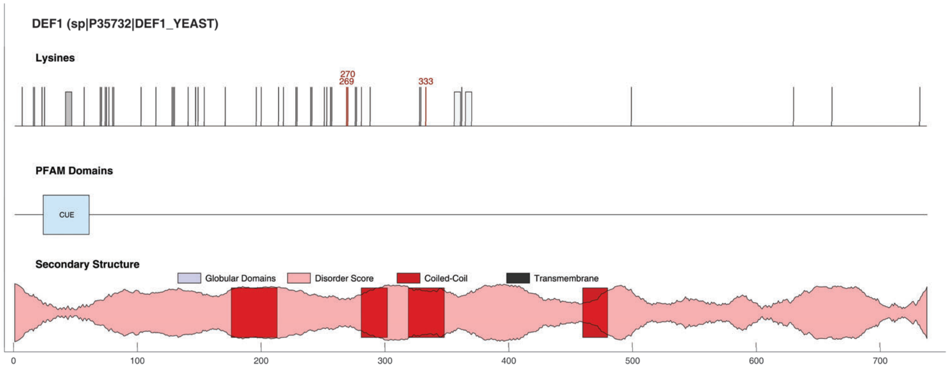
DEF1 (sp|P35732|DEF1_YEAST)
Lysines
270
269
333
PFAM Domains
CUE
Secondary Structure
Globular Domains
Disorder Score
Coiled-Coil
Transmembrane
0
100
200
300
400
500
600
700

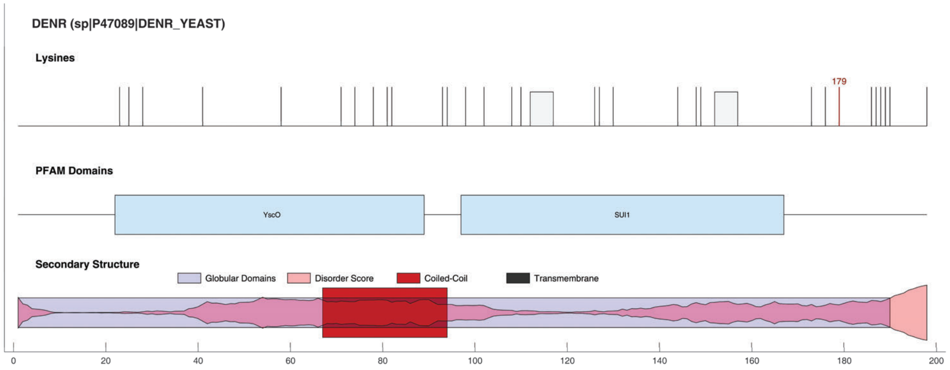
DENR (sp|P47089|DENR_YEAST)
Lysines
179
PFAM Domains
YscO
SUI1
Secondary Structure
Globular Domains
Disorder Score
Coiled-Coil
Transmembrane
0
20
40
60
80
100
120
140
160
180
200

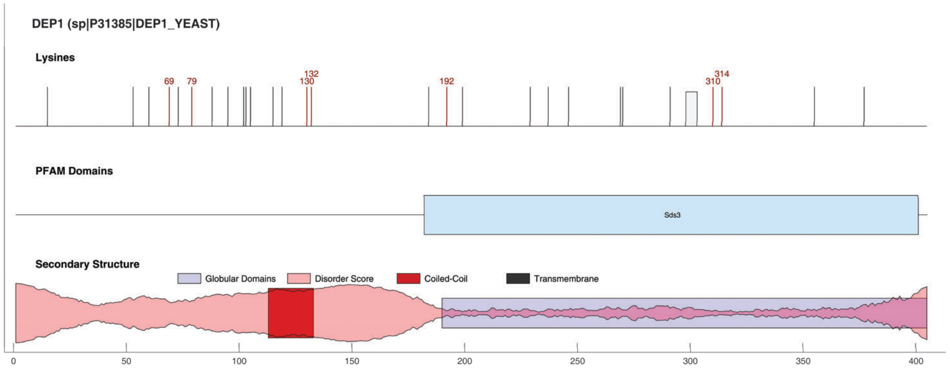
DEP1 (sp|P31385|DEP1_YEAST)
Lysines
69
79
132
130
192
314
310
PFAM Domains
Sds3
Secondary Structure
Globular Domains
Disorder Score
Coiled-Coil
Transmembrane
0
50
100
150
200
250
300
350
400

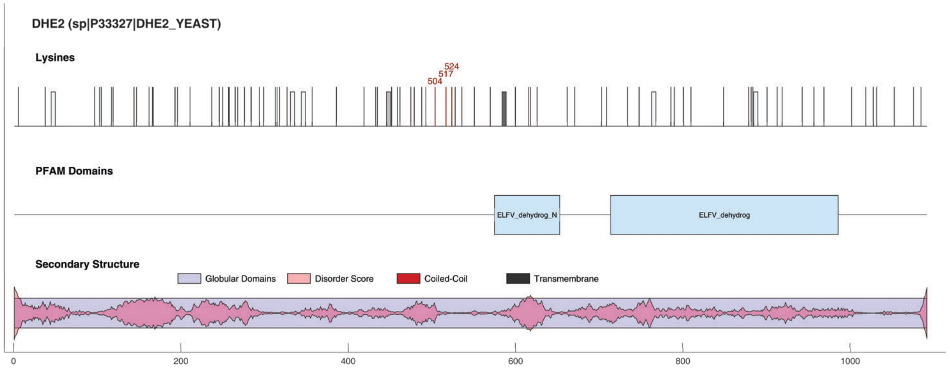
DHE2 (sp|P33327|DHE2_YEAST)
Lysines
524
517
504
PFAM Domains
ELFV_dehydrog_N
ELFV_dehydrog
Secondary Structure
Globular Domains
Disorder Score
Coiled-Coil
Transmembrane
0
200
400
600
800
1000

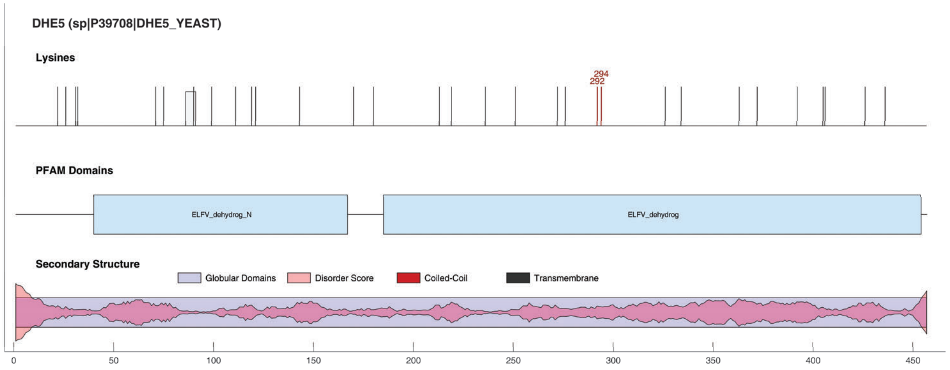
DHE5 (sp|P39708|DHE5_YEAST)
Lysines
294
292
PFAM Domains
ELFV_dehydrog_N
ELFV_dehydrog
Secondary Structure
Globular Domains
Disorder Score
Coiled-Coil
Transmembrane
0
50
100
150
200
250
300
350
400
450

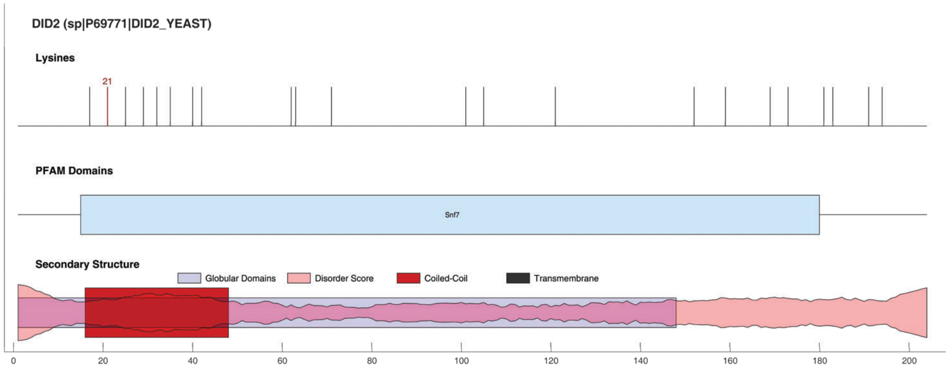
DID2 (sp|P69771|DID2_YEAST)
Lysines
21
PFAM Domains
Snf7
Secondary Structure
Globular Domains
Disorder Score
Coiled-Coil
Transmembrane
0
20
40
60
80
100
120
140
160
180
200

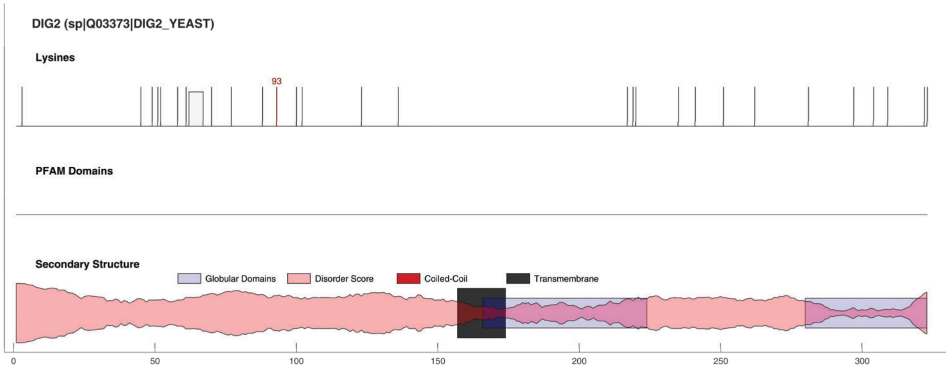
DIG2 (sp|Q03373|DIG2_YEAST)
Lysines
93
PFAM Domains
Secondary Structure
Globular Domains
Disorder Score
Coiled-Coil
Transmembrane
0
50
100
150
200
250
300

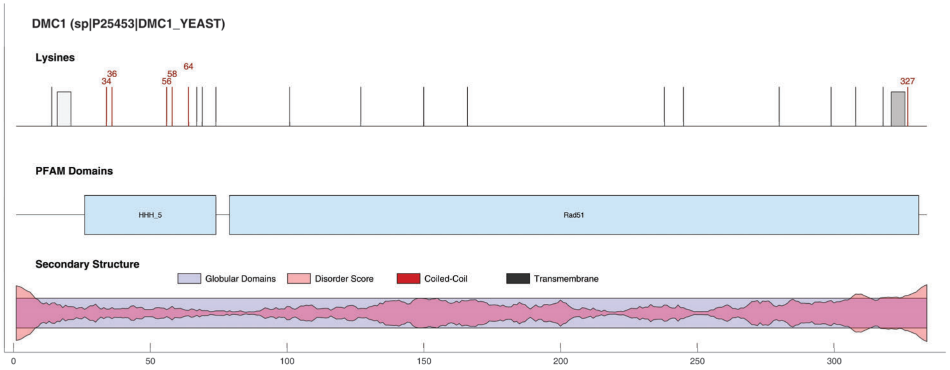
DMC1 (sp|P25453|DMC1_YEAST)
Lysines
36
34
58
56
64
327
PFAM Domains
HHH_5
Rad51
Secondary Structure
Globular Domains
Disorder Score
Coiled-Coil
Transmembrane
0
50
100
150
200
250
300

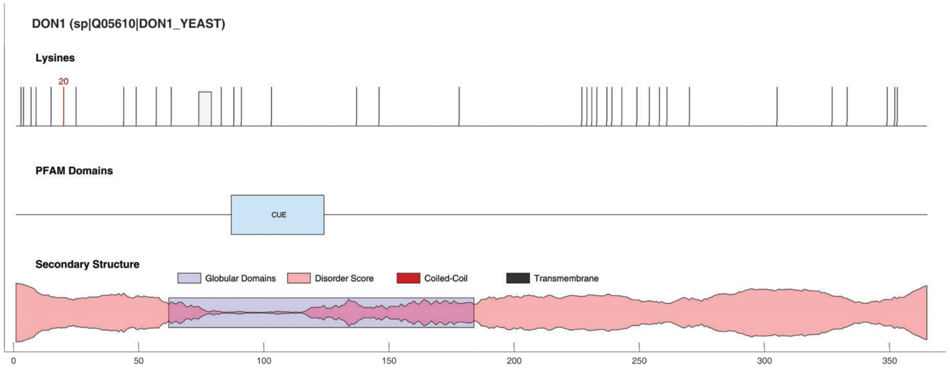
DON1 (sp|Q05610|DON1_YEAST)
Lysines
20
PFAM Domains
CUE
Secondary Structure
Globular Domains
Disorder Score
Coiled-Coil
Transmembrane
0
50
100
150
200
250
300
350

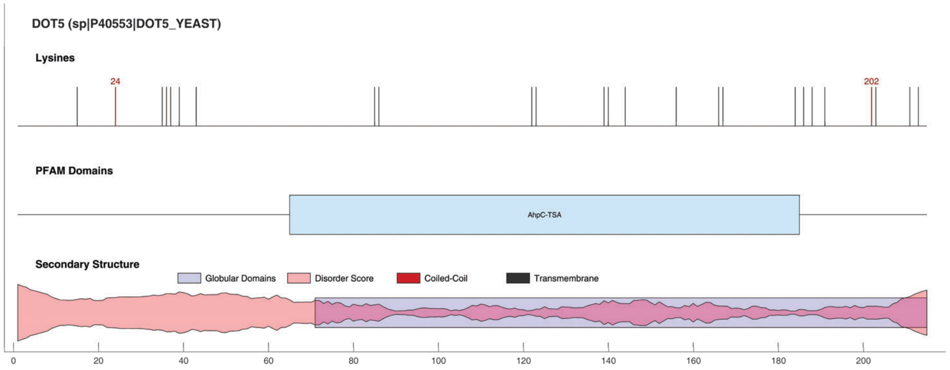
DOT5 (sp|P40553|DOT5_YEAST)
Lysines
24
202
PFAM Domains
AhpC-TSA
Secondary Structure
Globular Domains
Disorder Score
Coiled-Coil
Transmembrane
0
20
40
60
80
100
120
140
160
180
200

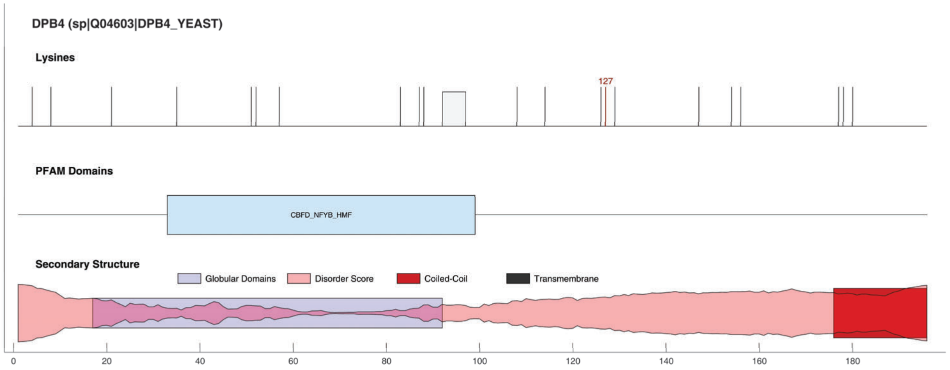
DPB4 (sp|Q04603|DPB4_YEAST)
Lysines
127
PFAM Domains
CBFD_NFYB_HMF
Secondary Structure
Globular Domains
Disorder Score
Coiled-Coil
Transmembrane
0
20
40
60
80
100
120
140
160
180

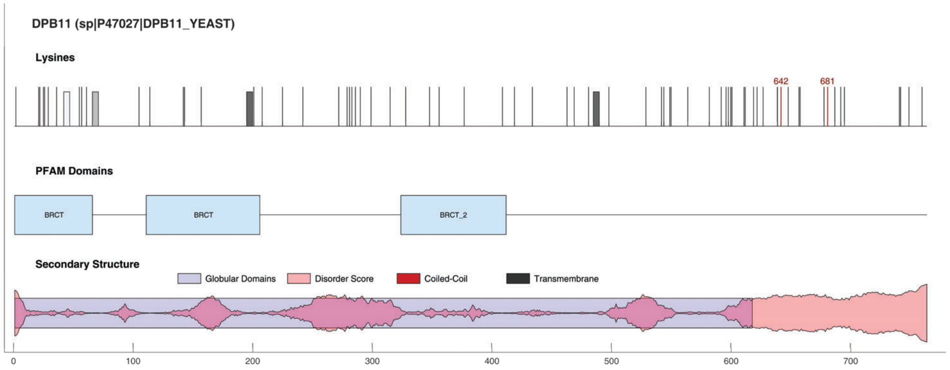
DPB11 (sp|P47027|DPB11_YEAST)
Lysines
642
681
PFAM Domains
BRCT
BRCT
BRCT_2
Secondary Structure
Globular Domains
Disorder Score
Coiled-Coil
Transmembrane
0
100
200
300
400
500
600
700

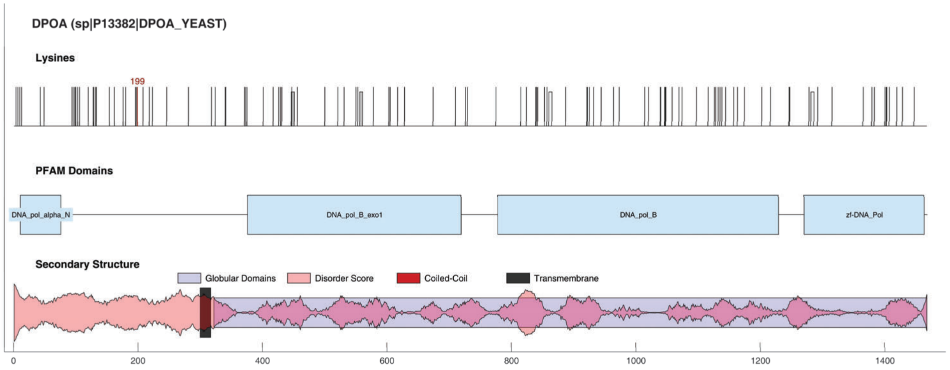
DPOA (sp|P13382|DPOA_YEAST)
Lysines
199
PFAM Domains
DNA_pol_alpha_N
DNA_pol_B_exo1
DNA_pol_B
zf-DNA_Pol
Secondary Structure
Globular Domains
Disorder Score
Coiled-Coil
Transmembrane
0
200
400
600
800
1000
1200
1400

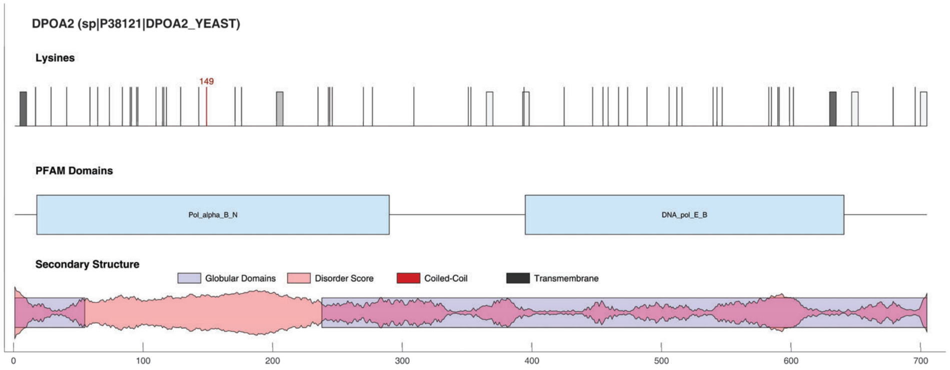
DPOA2 (sp|P38121|DPOA2_YEAST)
Lysines
149
PFAM Domains
Pol_alpha_B_N
DNA_pol_E_B
Secondary Structure
Globular Domains
Disorder Score
Coiled-Coil
Transmembrane
0
100
200
300
400
500
600
700

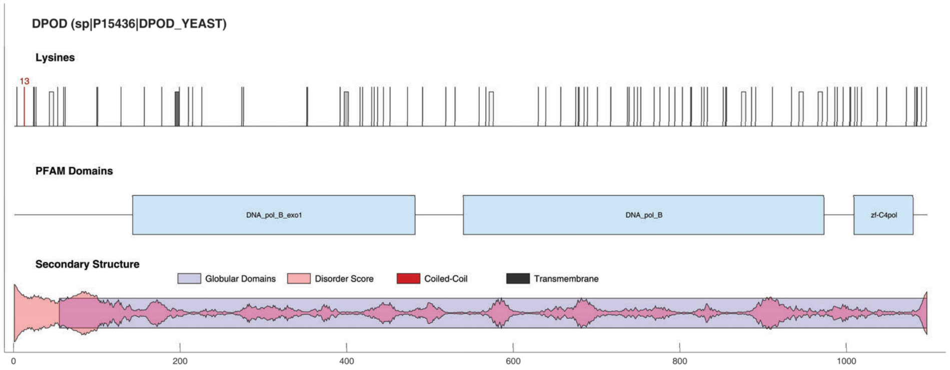
DPOD (sp|P15436|DPOD_YEAST)
Lysines
13
PFAM Domains
DNA_pol_B_exo1
DNA_pol_B
zf-C4pol
Secondary Structure
Globular Domains
Disorder Score
Coiled-Coil
Transmembrane
0
200
400
600
800
1000

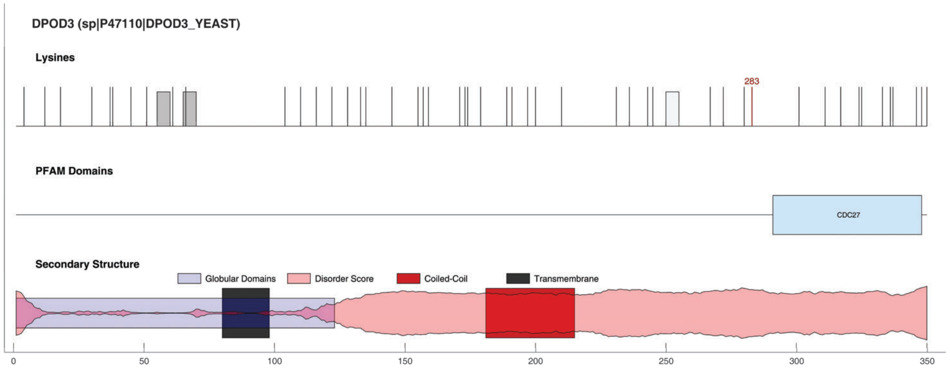
DPOD3 (sp|P47110|DPOD3_YEAST)
Lysines
283
PFAM Domains
CDC27
Secondary Structure
Globular Domains
Disorder Score
Coiled-Coil
Transmembrane
0
50
100
150
200
250
300
350

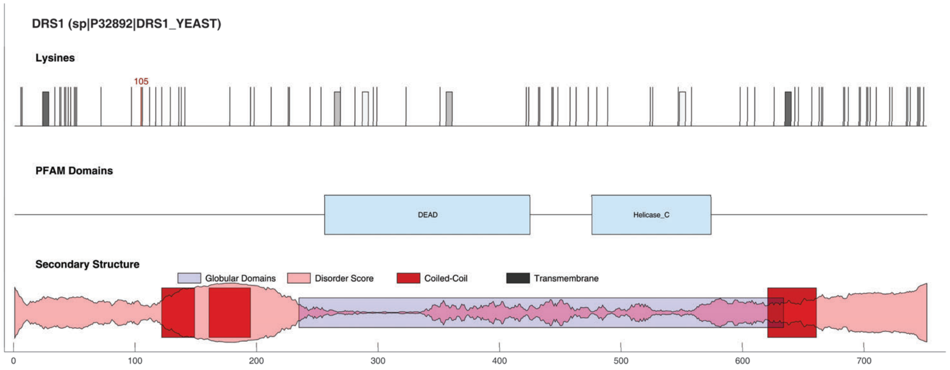
DRS1 (sp|P32892|DRS1_YEAST)
Lysines
105
PFAM Domains
DEAD
Helicase_C
Secondary Structure
Globular Domains
Disorder Score
Coiled-Coil
Transmembrane
0
100
200
300
400
500
600
700

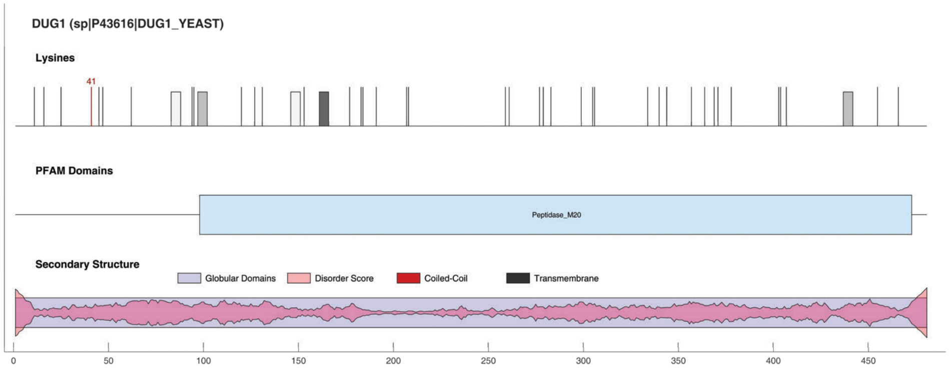
DUG1 (sp|P43616|DUG1_YEAST)
Lysines
41
PFAM Domains
Peptidase_M20
Secondary Structure
Globular Domains
Disorder Score
Coiled-Coil
Transmembrane
0
50
100
150
200
250
300
350
400
450

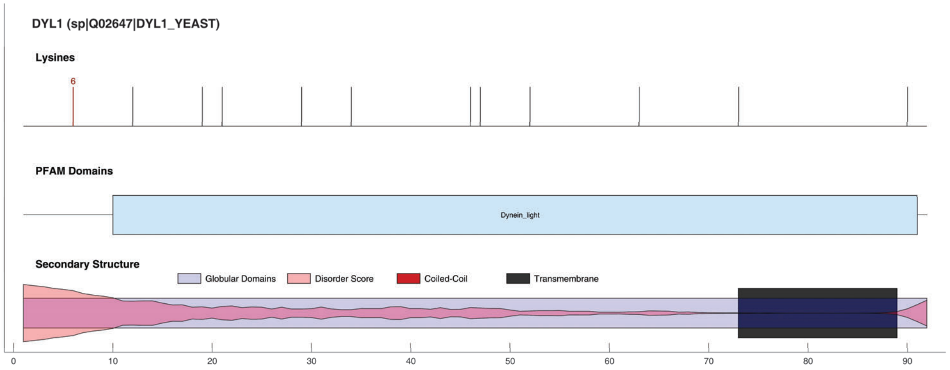
DYL1 (sp|Q02647|DYL1_YEAST)
Lysines
6
PFAM Domains
Dynein_light
Secondary Structure
Globular Domains
Disorder Score
Coiled-Coil
Transmembrane
0
10
20
30
40
50
60
70
80
90

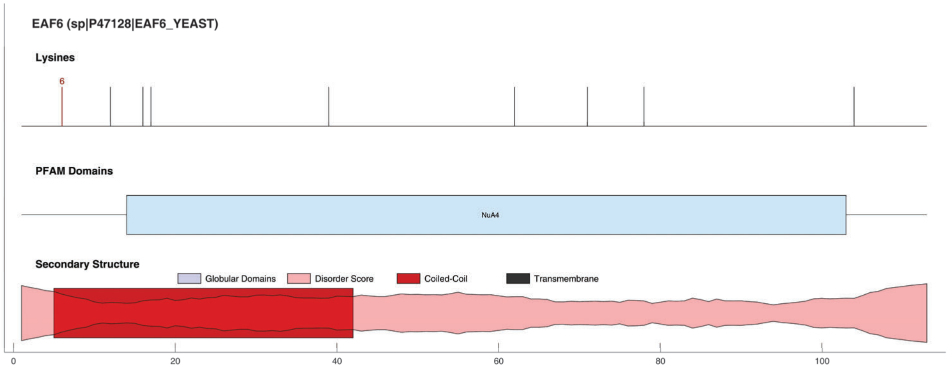
EAF6 (sp|P47128|EAF6_YEAST)
Lysines
6
PFAM Domains
NuA4
Secondary Structure
Globular Domains
Disorder Score
Coiled-Coil
Transmembrane
0
20
40
60
80
100

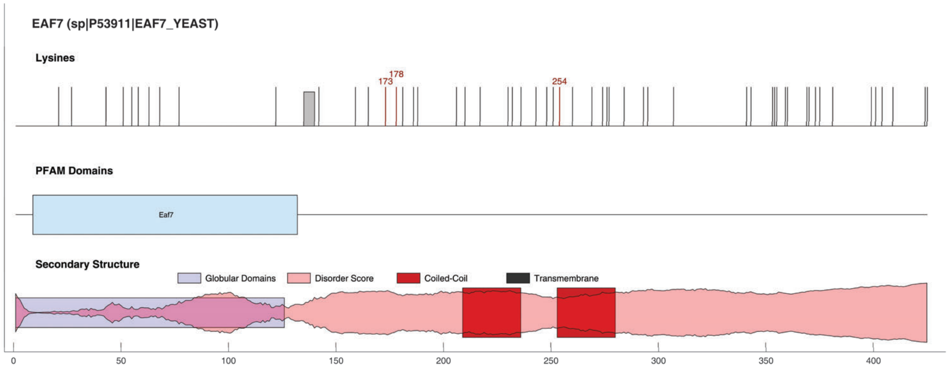
EAF7 (sp|P53911|EAF7_YEAST)
Lysines
173
178
254
PFAM Domains
Eaf7
Secondary Structure
Globular Domains
Disorder Score
Coiled-Coil
Transmembrane
0
50
100
150
200
250
300
350
400

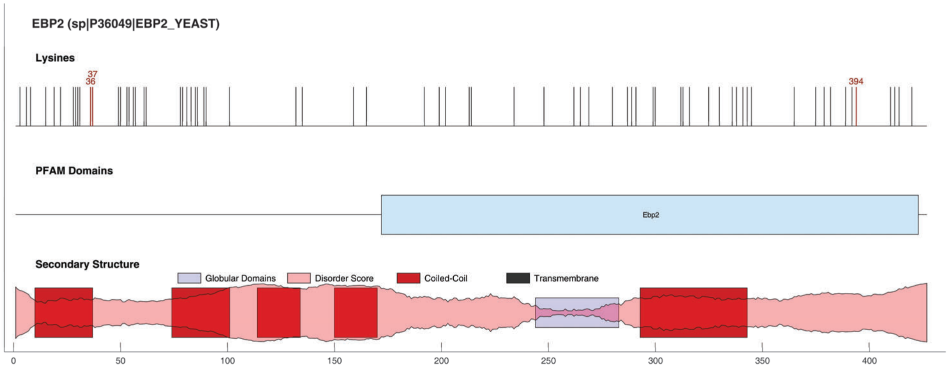
EBP2 (sp|P36049|EBP2_YEAST)
Lysines
36
37
394
PFAM Domains
Ebp2
Secondary Structure
Globular Domains
Disorder Score
Coiled-Coil
Transmembrane
0
50
100
150
200
250
300
350
400

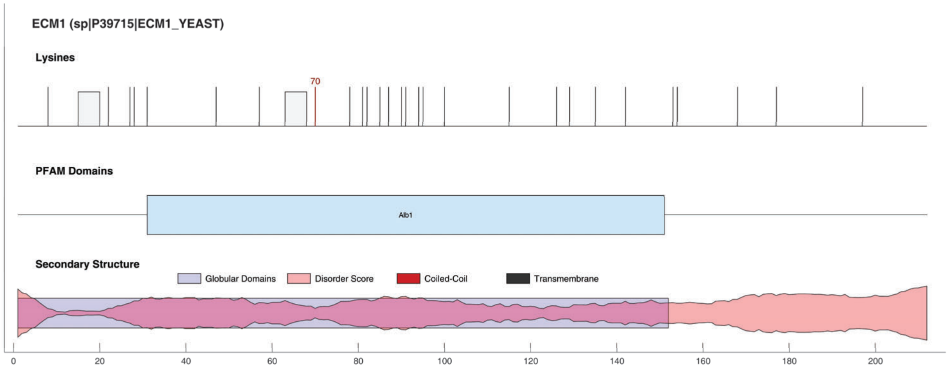
ECM1 (sp|P39715|ECM1_YEAST)
Lysines
70
PFAM Domains
Alb1
Secondary Structure
Globular Domains
Disorder Score
Coiled-Coil
Transmembrane
0
20
40
60
80
100
120
140
160
180
200

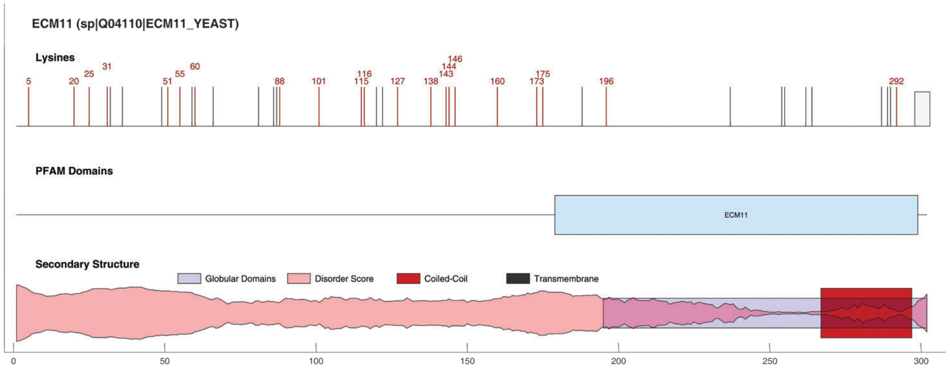
ECM11 (sp|Q04110|ECM11_YEAST)
Lysines
5
20
25
31
51
55
60
88
101
115
116
127
138
143
144
146
160
173
175
196
292
PFAM Domains
ECM11
Secondary Structure
Globular Domains
Disorder Score
Coiled-Coil
Transmembrane
0
50
100
150
200
250
300

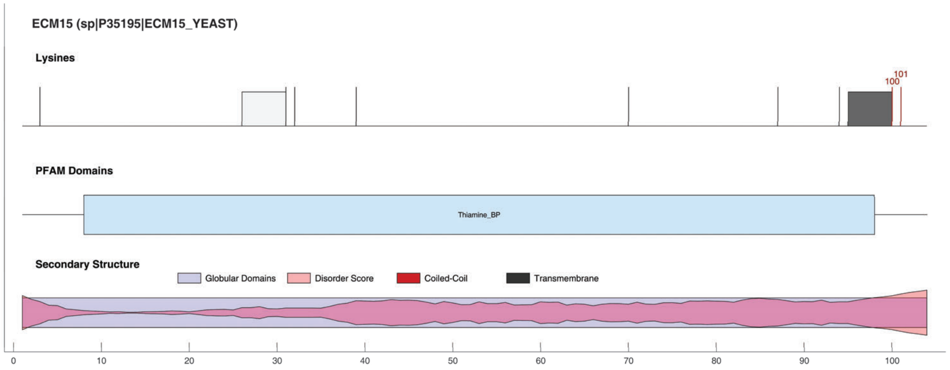
ECM15 (sp|P35195|ECM15_YEAST)
Lysines
100
101
PFAM Domains
Thiamine_BP
Secondary Structure
Globular Domains
Disorder Score
Coiled-Coil
Transmembrane
0
10
20
30
40
50
60
70
80
90
100

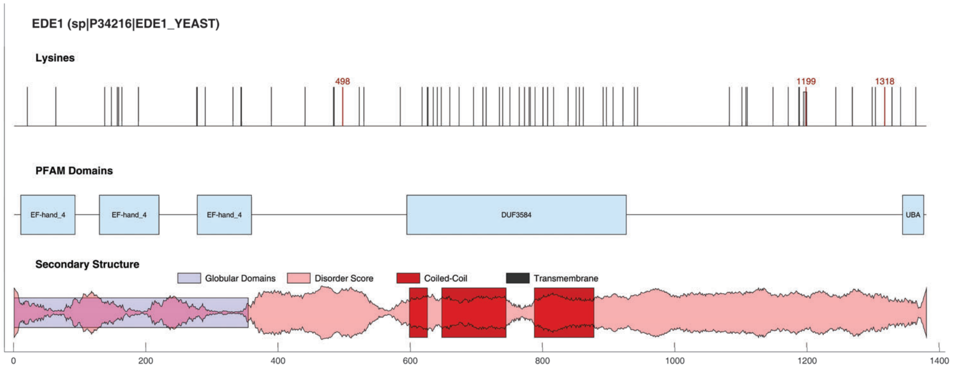
EDE1 (sp|P34216|EDE1_YEAST)
Lysines
498
1199
1318
PFAM Domains
EF-hand_4
EF-hand_4
EF-hand_4
DUF3584
UBA
Secondary Structure
Globular Domains
Disorder Score
Coiled-Coil
Transmembrane
0
200
400
600
800
1000
1200
1400

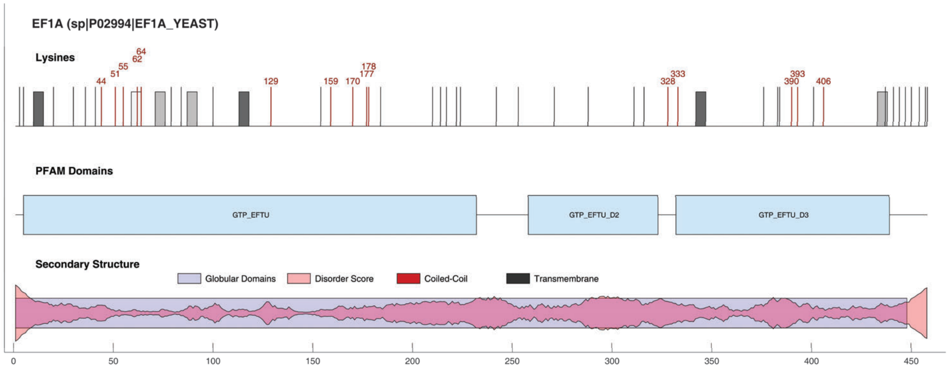
EF1A (sp|P02994|EF1A_YEAST)
Lysines
44
51
55
62
64
129
159
170
177
178
328
333
390
393
406
PFAM Domains
GTP_EFTU
GTP_EFTU_D2
GTP_EFTU_D3
Secondary Structure
Globular Domains
Disorder Score
Coiled-Coil
Transmembrane
0
50
100
150
200
250
300
350
400
450

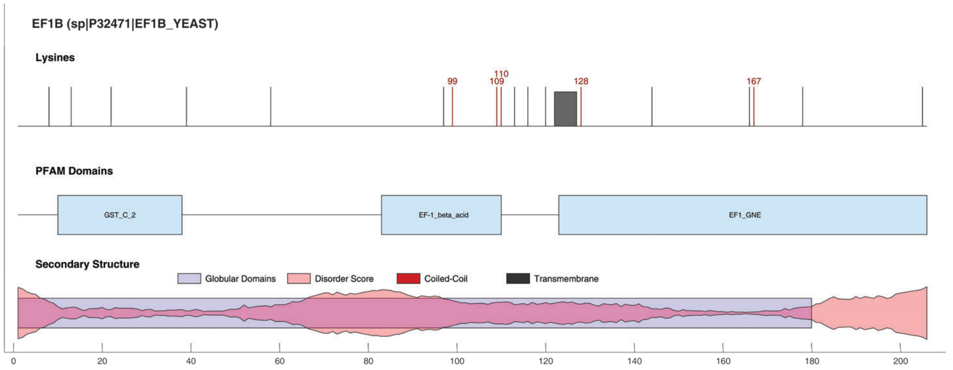
EF1B (sp|P32471|EF1B_YEAST)
Lysines
99
109
110
128
167
PFAM Domains
GST_C_2
EF-1_beta_acid
EF1_GNE
Secondary Structure
Globular Domains
Disorder Score
Coiled-Coil
Transmembrane
0
20
40
60
80
100
120
140
160
180
200

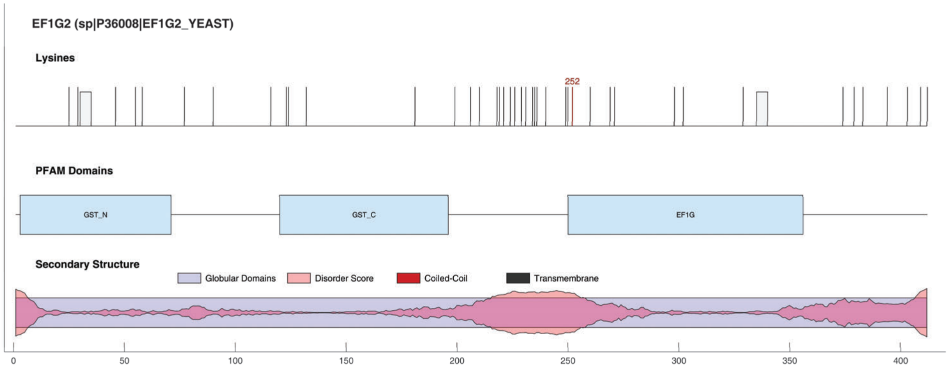
EF1G2 (sp|P36008|EF1G2_YEAST)
Lysines
252
PFAM Domains
GST_N
GST_C
EF1G
Secondary Structure
Globular Domains
Disorder Score
Coiled-Coil
Transmembrane
0
50
100
150
200
250
300
350
400

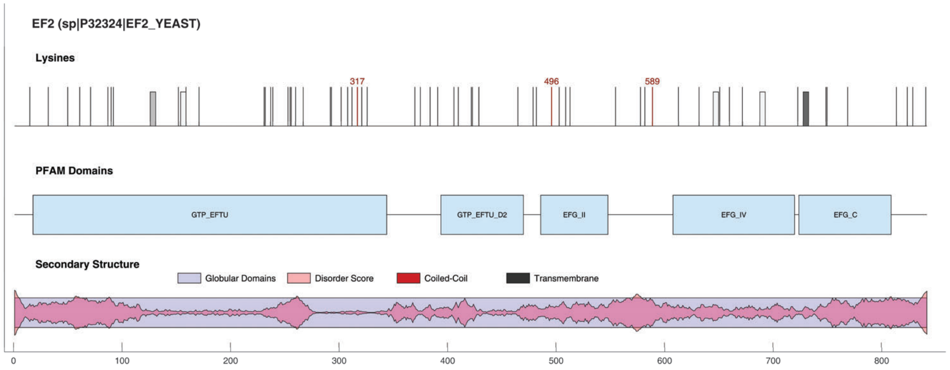
EF2 (sp|P32324|EF2_YEAST)
Lysines
317
496
589
PFAM Domains
GTP_EFTU
GTP_EFTU_D2
EFG_II
EFG_IV
EFG_C
Secondary Structure
Globular Domains
Disorder Score
Coiled-Coil
Transmembrane
0
100
200
300
400
500
600
700
800

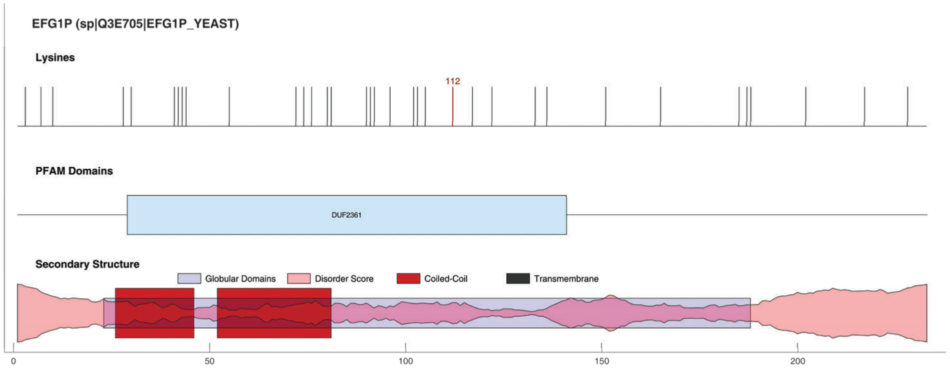
EFG1P (sp|Q3E705|EFG1P_YEAST)
Lysines
112
PFAM Domains
DUF2361
Secondary Structure
Globular Domains
Disorder Score
Coiled-Coil
Transmembrane
0
50
100
150
200

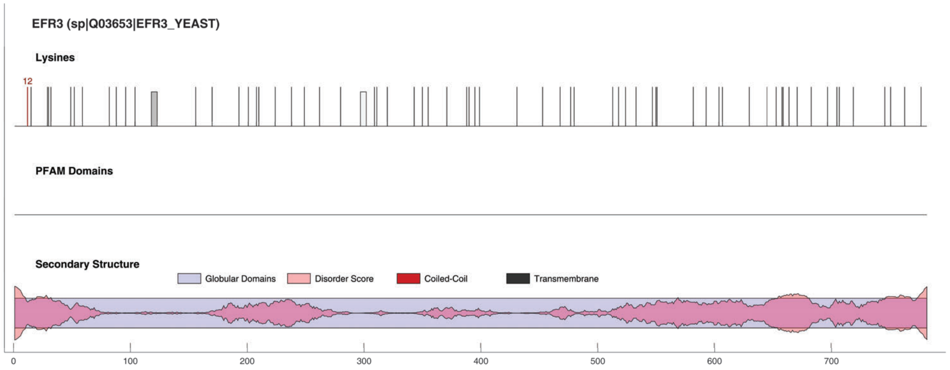
EFR3 (sp|Q03653|EFR3_YEAST)
Lysines
12
PFAM Domains
Secondary Structure
Globular Domains
Disorder Score
Coiled-Coil
Transmembrane
0
100
200
300
400
500
600
700

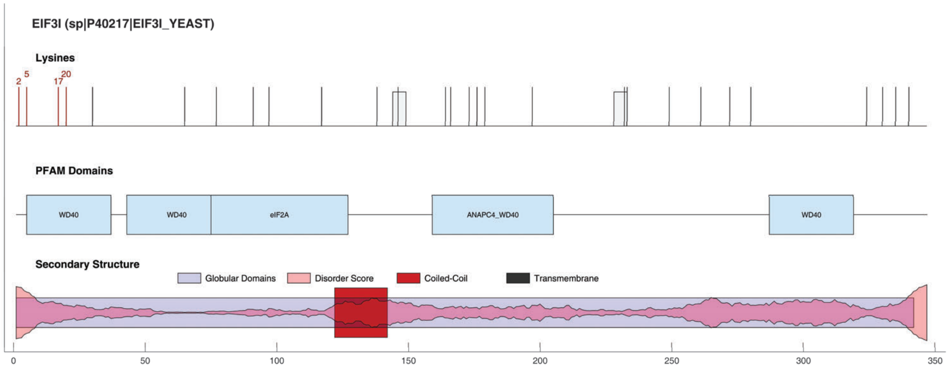
EIF3I (sp|P40217|EIF3I_YEAST)
Lysines
2
5
17
20
PFAM Domains
WD40
WD40
eIF2A
ANAPC4_WD40
WD40
Secondary Structure
Globular Domains
Disorder Score
Coiled-Coil
Transmembrane
0
50
100
150
200
250
300
350

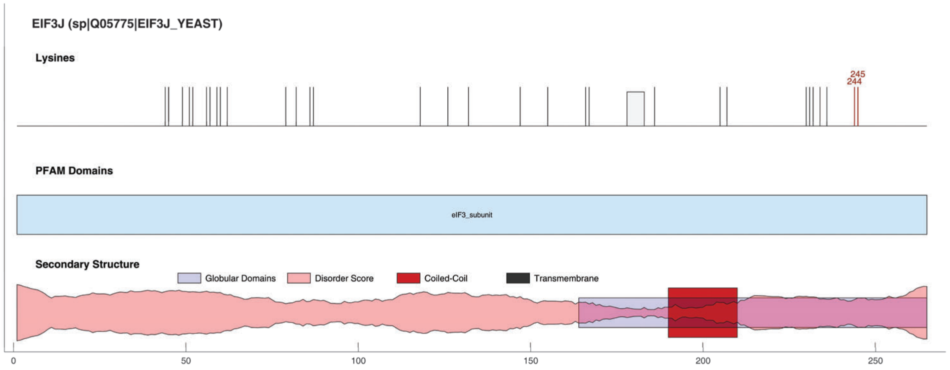
EIF3J (sp|Q05775|EIF3J_YEAST)
Lysines
245
244
PFAM Domains
eIF3_subunit
Secondary Structure
Globular Domains
Disorder Score
Coiled-Coil
Transmembrane
0
50
100
150
200
250

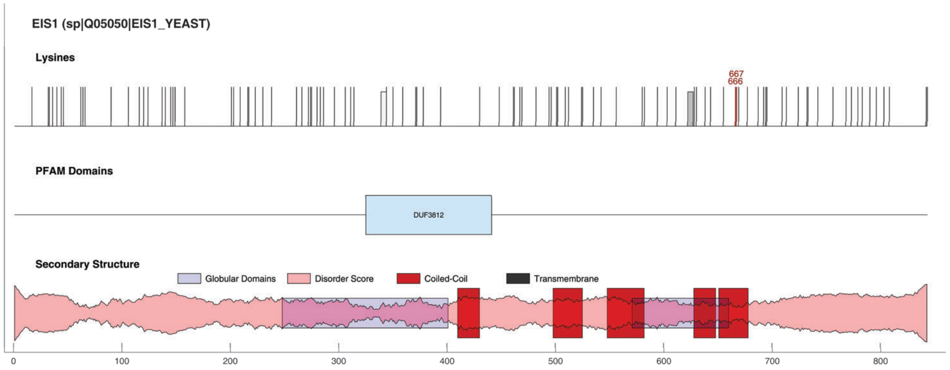
EIS1 (sp|Q05050|EIS1_YEAST)
Lysines
667
666
PFAM Domains
DUF3812
Secondary Structure
Globular Domains
Disorder Score
Coiled-Coil
Transmembrane
0
100
200
300
400
500
600
700
800

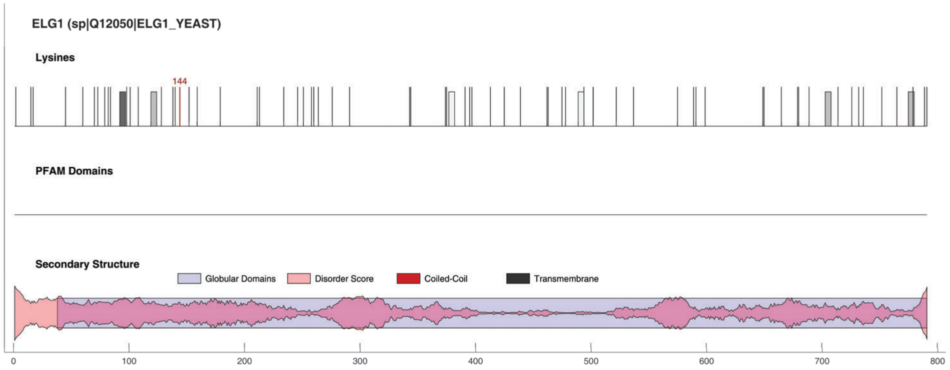
ELG1 (sp|Q12050|ELG1_YEAST)
Lysines
144
PFAM Domains
Secondary Structure
Globular Domains
Disorder Score
Coiled-Coil
Transmembrane
0
100
200
300
400
500
600
700
800

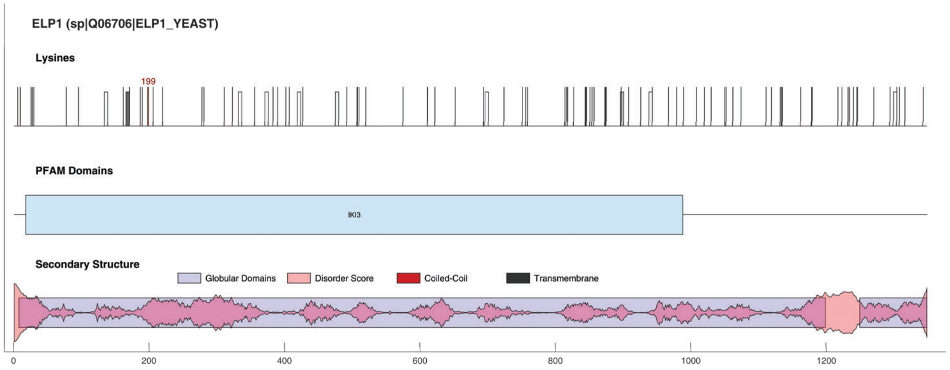
ELP1 (sp|Q06706|ELP1_YEAST)
Lysines
199
PFAM Domains
IKI3
Secondary Structure
Globular Domains
Disorder Score
Coiled-Coil
Transmembrane
0
200
400
600
800
1000
1200

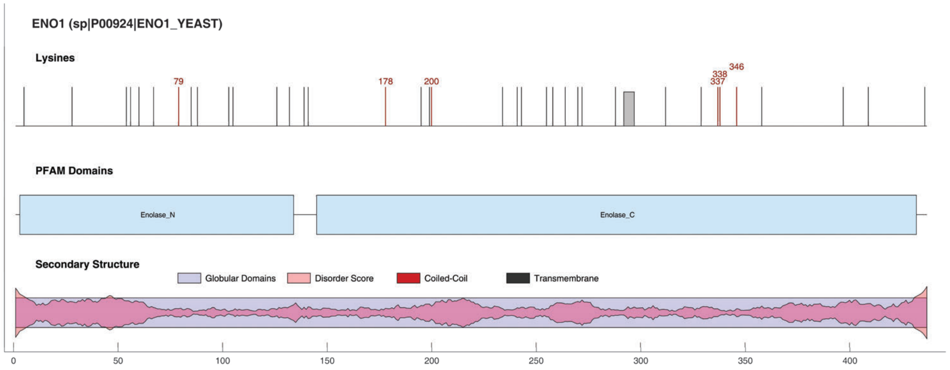
ENO1 (sp|P00924|ENO1_YEAST)
Lysines
79
178
200
338
337
346
PFAM Domains
Enolase_N
Enolase_C
Secondary Structure
Globular Domains
Disorder Score
Coiled-Coil
Transmembrane
0
50
100
150
200
250
300
350
400

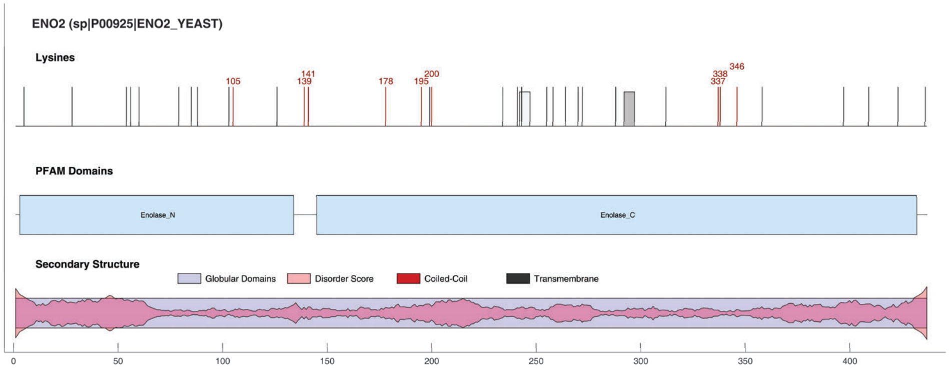
ENO2 (sp|P00925|ENO2_YEAST)
Lysines
105
141
139
178
195
200
338
337
346
PFAM Domains
Enolase_N
Enolase_C
Secondary Structure
Globular Domains
Disorder Score
Coiled-Coil
Transmembrane
0
50
100
150
200
250
300
350
400

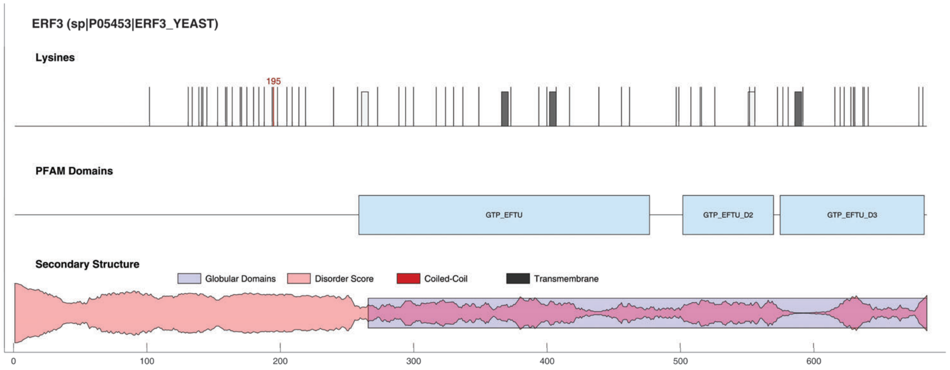
ERF3 (sp|P05453|ERF3_YEAST)
Lysines
195
PFAM Domains
GTP_EFTU
GTP_EFTU_D2
GTP_EFTU_D3
Secondary Structure
Globular Domains
Disorder Score
Coiled-Coil
Transmembrane
0
100
200
300
400
500
600

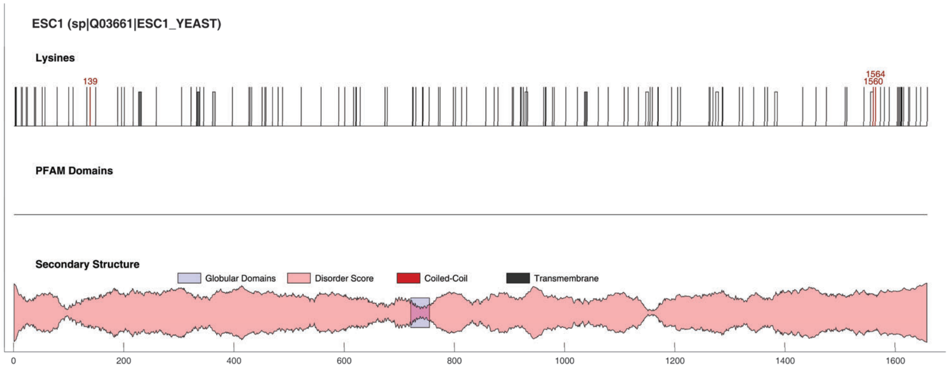
ESC1 (sp|Q03661|ESC1_YEAST)
Lysines
139
1564
1560
PFAM Domains
Secondary Structure
Globular Domains
Disorder Score
Coiled-Coil
Transmembrane
0
200
400
600
800
1000
1200
1400
1600

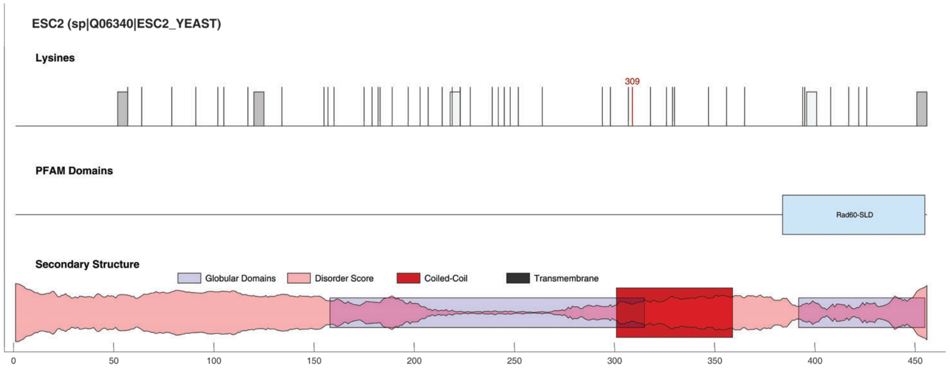
ESC2 (sp|Q06340|ESC2_YEAST)
Lysines
309
PFAM Domains
Rad60-SLD
Secondary Structure
Globular Domains
Disorder Score
Coiled-Coil
Transmembrane
0
50
100
150
200
250
300
350
400
450

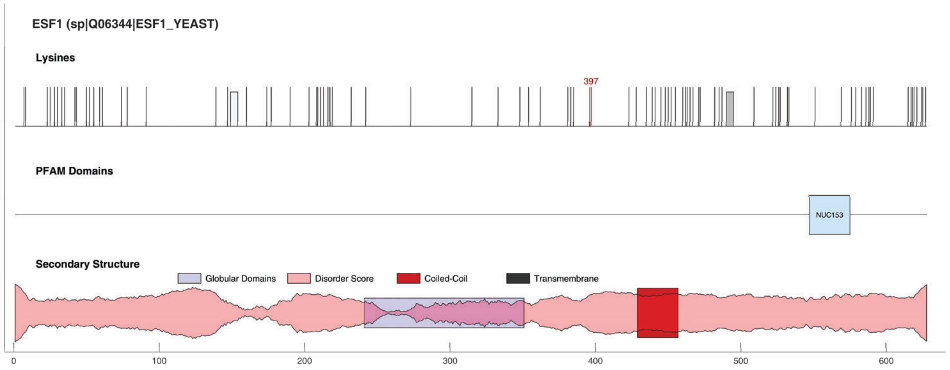
ESF1 (sp|Q06344|ESF1_YEAST)
Lysines
397
PFAM Domains
NUC153
Secondary Structure
Globular Domains
Disorder Score
Coiled-Coil
Transmembrane
0
100
200
300
400
500
600

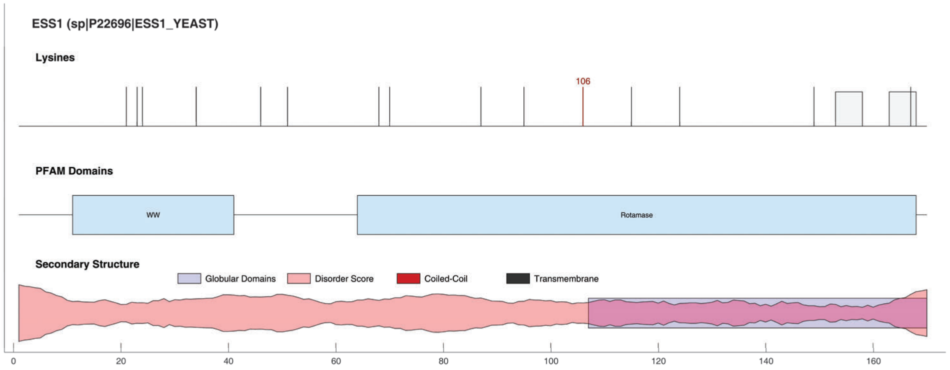
ESS1 (sp|P22696|ESS1_YEAST)
Lysines
106
PFAM Domains
WW
Rotamase
Secondary Structure
Globular Domains
Disorder Score
Coiled-Coil
Transmembrane
0
20
40
60
80
100
120
140
160

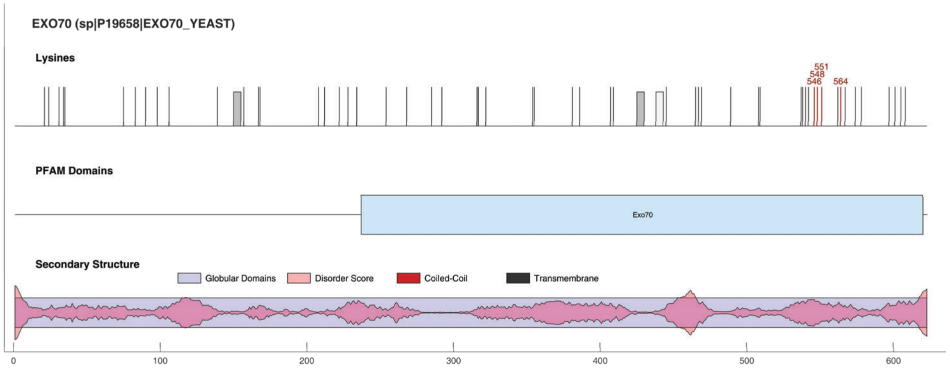
EXO70 (sp|P19658|EXO70_YEAST)
Lysines
551
548
546
564
PFAM Domains
Exo70
Secondary Structure
Globular Domains
Disorder Score
Coiled-Coil
Transmembrane
0
100
200
300
400
500
600

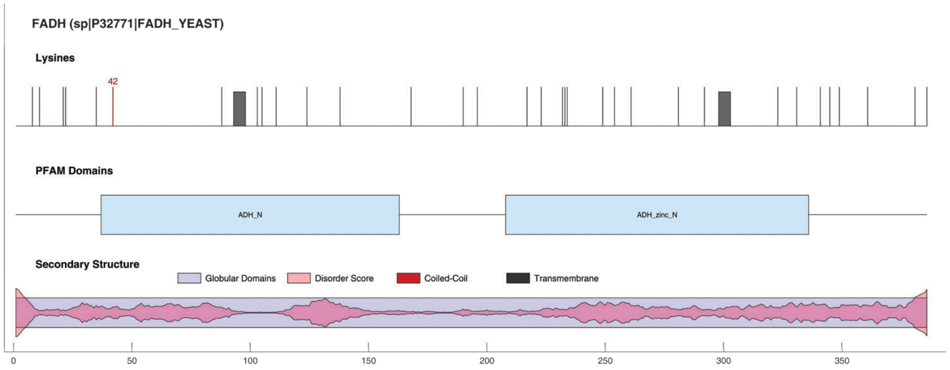
FADH (sp|P32771|FADH_YEAST)
Lysines
42
PFAM Domains
ADH_N
ADH_zinc_N
Secondary Structure
Globular Domains
Disorder Score
Coiled-Coil
Transmembrane
0
50
100
150
200
250
300
350

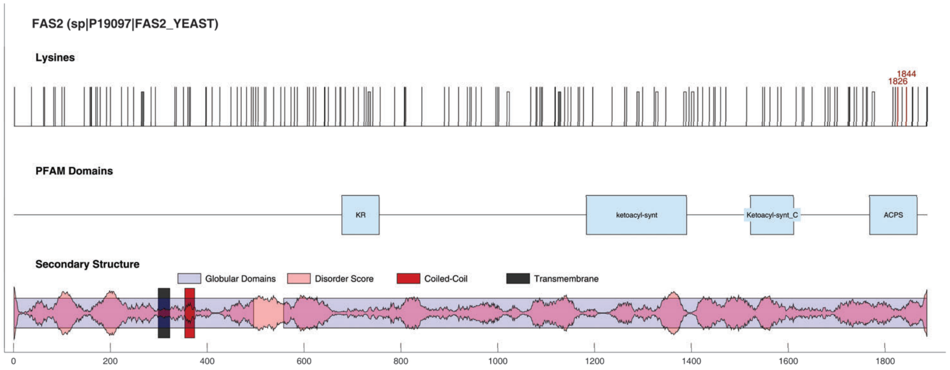
FAS2 (sp|P19097|FAS2_YEAST)
Lysines
1844
1826
PFAM Domains
KR
ketoacyl-synt
Ketoacyl-synt_C
ACPS
Secondary Structure
Globular Domains
Disorder Score
Coiled-Coil
Transmembrane
0
200
400
600
800
1000
1200
1400
1600
1800

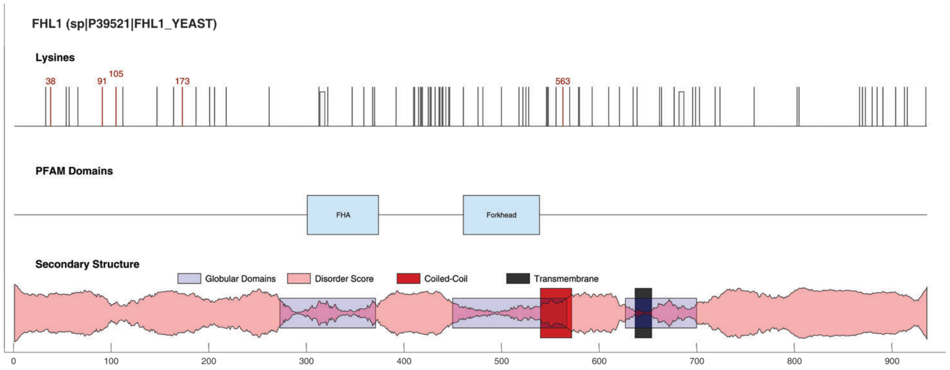
FHL1 (sp|P39521|FHL1_YEAST)
Lysines
38
91
105
173
563
PFAM Domains
FHA
Forkhead
Secondary Structure
Globular Domains
Disorder Score
Coiled-Coil
Transmembrane
0
100
200
300
400
500
600
700
800
900

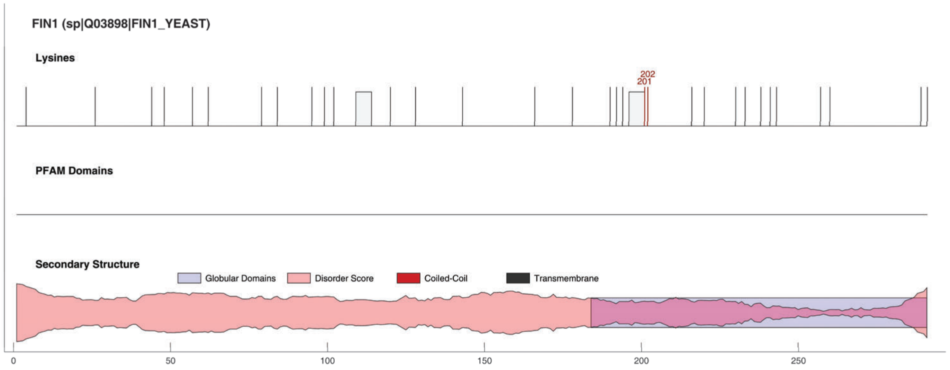
FIN1 (sp|Q03898|FIN1_YEAST)
Lysines
202
201
PFAM Domains
Secondary Structure
Globular Domains
Disorder Score
Coiled-Coil
Transmembrane
0
50
100
150
200
250

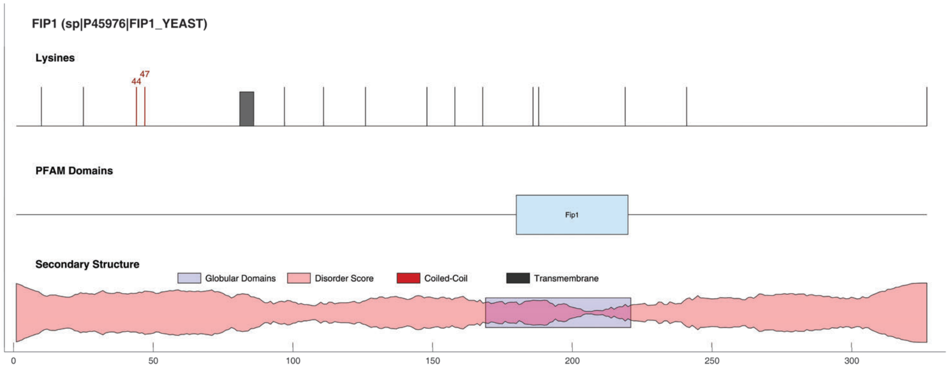
FIP1 (sp|P45976|FIP1_YEAST)
Lysines
44
47
PFAM Domains
Fip1
Secondary Structure
Globular Domains
Disorder Score
Coiled-Coil
Transmembrane
0
50
100
150
200
250
300

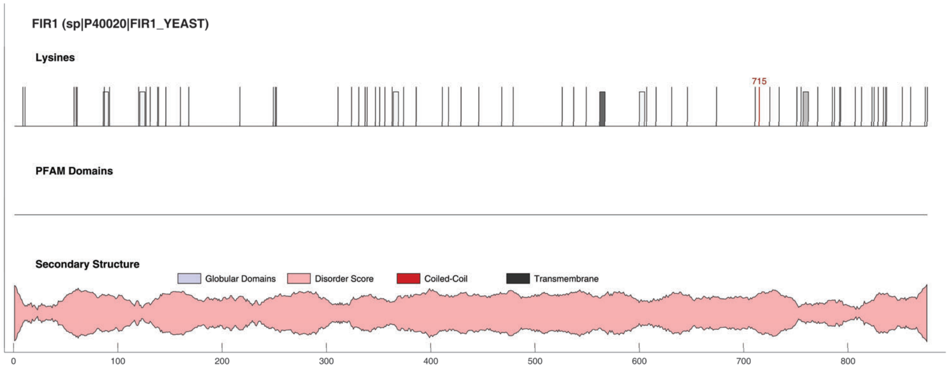
FIR1 (sp|P40020|FIR1_YEAST)
Lysines
715
PFAM Domains
Secondary Structure
Globular Domains
Disorder Score
Coiled-Coil
Transmembrane
0
100
200
300
400
500
600
700
800

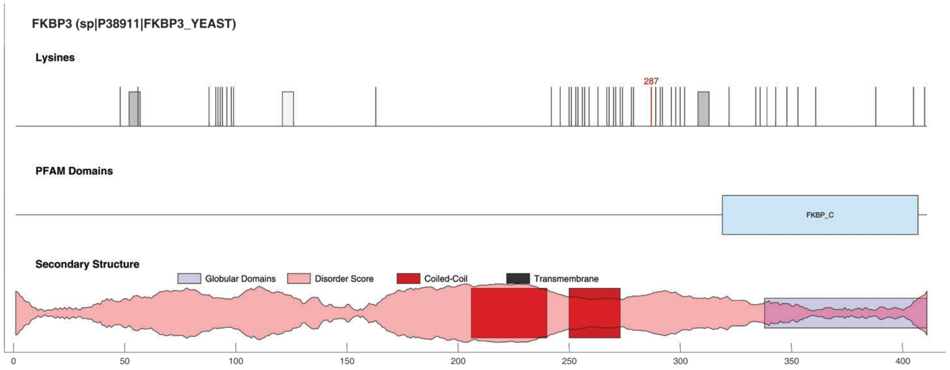
FKBP3 (sp|P38911|FKBP3_YEAST)
Lysines
287
PFAM Domains
FKBP_C
Secondary Structure
Globular Domains
Disorder Score
Coiled-Coil
Transmembrane
0
50
100
150
200
250
300
350
400

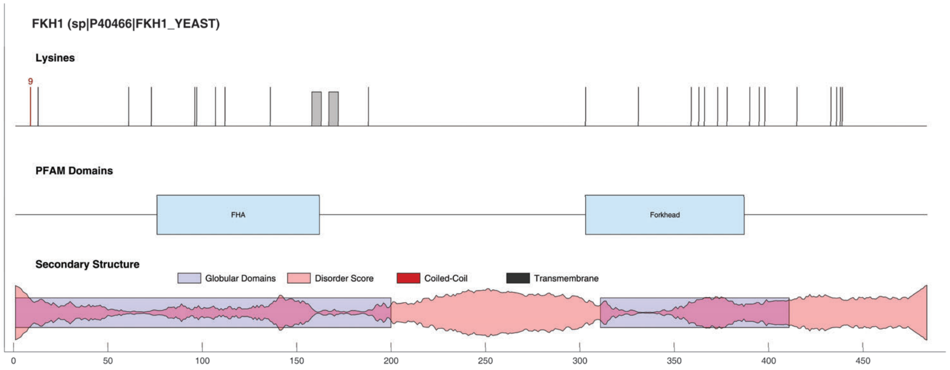

FKH1 (sp|P40466|FKH1_YEAST)
Lysines
9
PFAM Domains
FHA
Forkhead
Secondary Structure
Globular Domains
Disorder Score
Coiled-Coil
Transmembrane
0
50
100
150
200
250
300
350
400
450

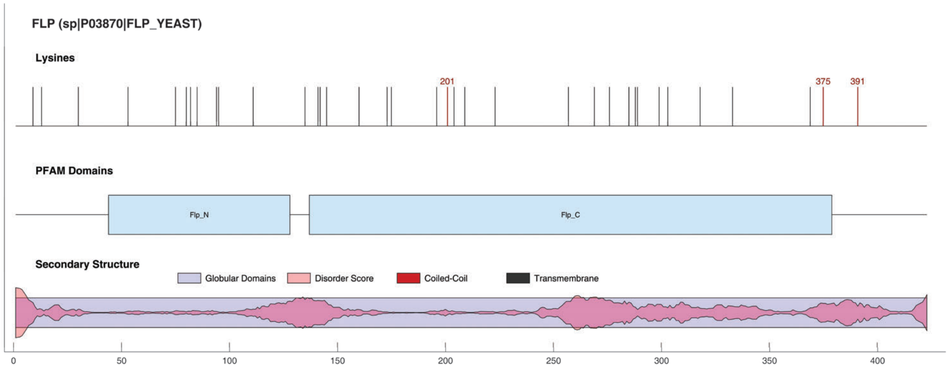

FLP (sp|P03870|FLP_YEAST)
Lysines
201
375
391
PFAM Domains
Flp_N
Flp_C
Secondary Structure
Globular Domains
Disorder Score
Coiled-Coil
Transmembrane
0
50
100
150
200
250
300
350
400

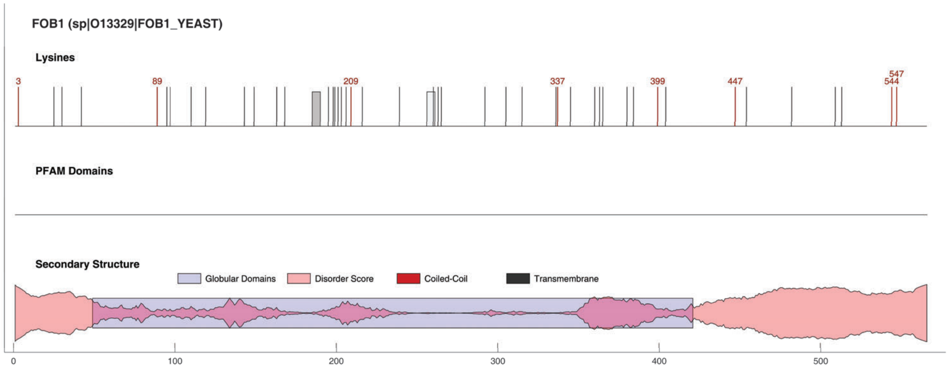

FOB1 (sp|O13329|FOB1_YEAST)
Lysines
3
89
209
337
399
447
544
547
PFAM Domains
Secondary Structure
Globular Domains
Disorder Score
Coiled-Coil
Transmembrane
0
100
200
300
400
500

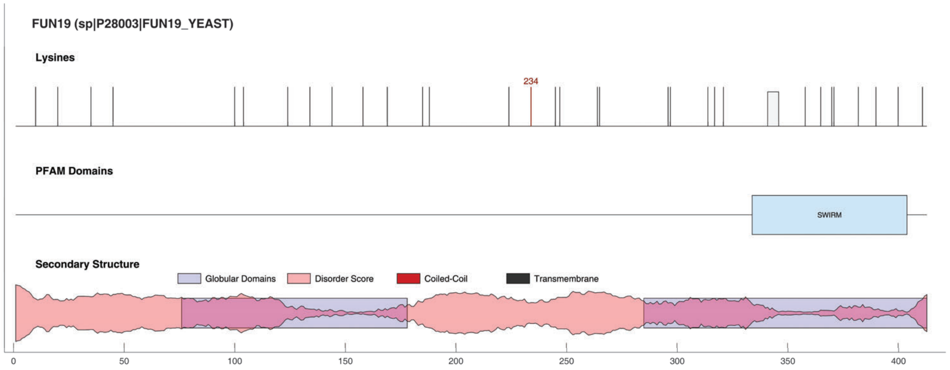

FUN19 (sp|P28003|FUN19_YEAST)
Lysines
234
PFAM Domains
SWIRM
Secondary Structure
Globular Domains
Disorder Score
Coiled-Coil
Transmembrane
0
50
100
150
200
250
300
350
400

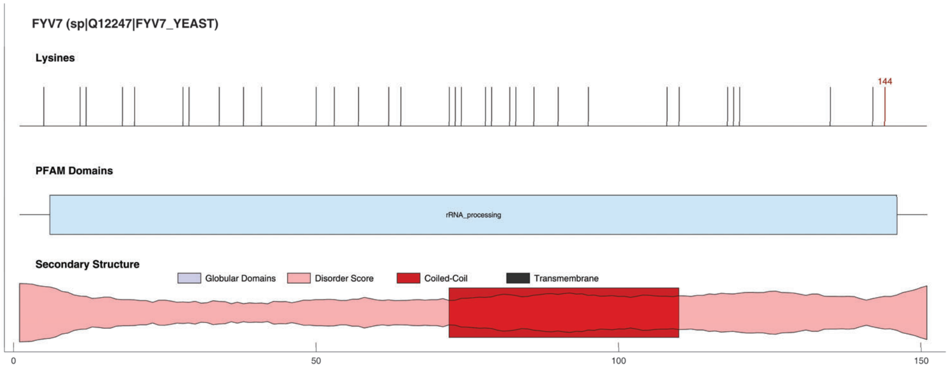
FYV7 (sp|Q12247|FYV7_YEAST)
Lysines
144
PFAM Domains
rRNA_processing
Secondary Structure
Globular Domains
Disorder Score
Coiled-Coil
Transmembrane
0
50
100
150

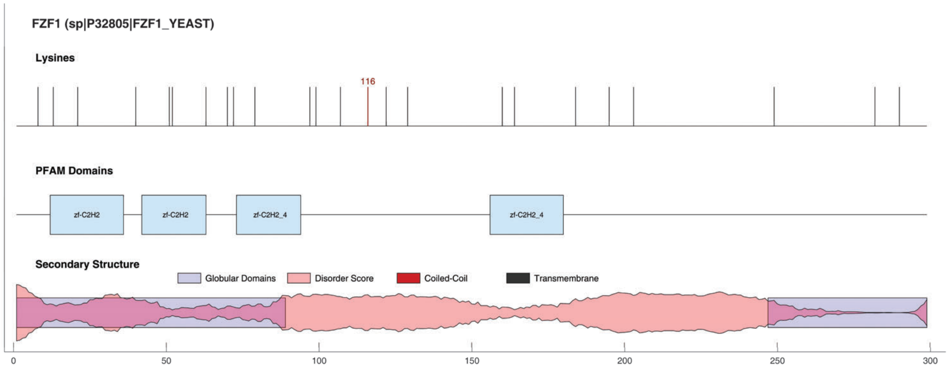
FZF1 (sp|P32805|FZF1_YEAST)
Lysines
116
PFAM Domains
zf-C2H2
zf-C2H2
zf-C2H2_4
zf-C2H2_4
Secondary Structure
Globular Domains
Disorder Score
Coiled-Coil
Transmembrane
0
50
100
150
200
250
300

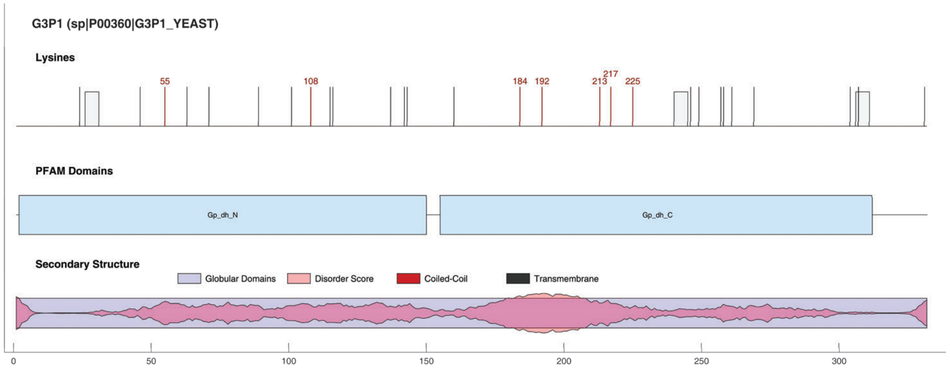
G3P1 (sp|P00360|G3P1_YEAST)
Lysines
55
108
184
192
213
217
225
PFAM Domains
Gp_dh_N
Gp_dh_C
Secondary Structure
Globular Domains
Disorder Score
Coiled-Coil
Transmembrane
0
50
100
150
200
250
300

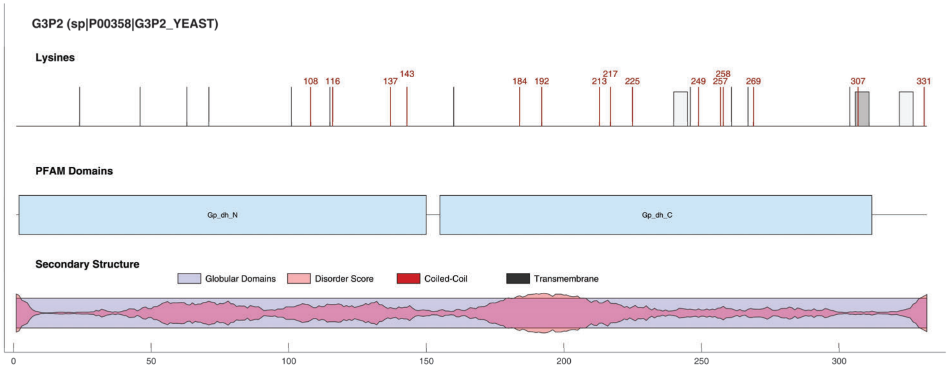
G3P2 (sp|P00358|G3P2_YEAST)
Lysines
108
116
137
143
184
192
213
217
225
249
257
258
269
307
331
PFAM Domains
Gp_dh_N
Gp_dh_C
Secondary Structure
Globular Domains
Disorder Score
Coiled-Coil
Transmembrane
0
50
100
150
200
250
300

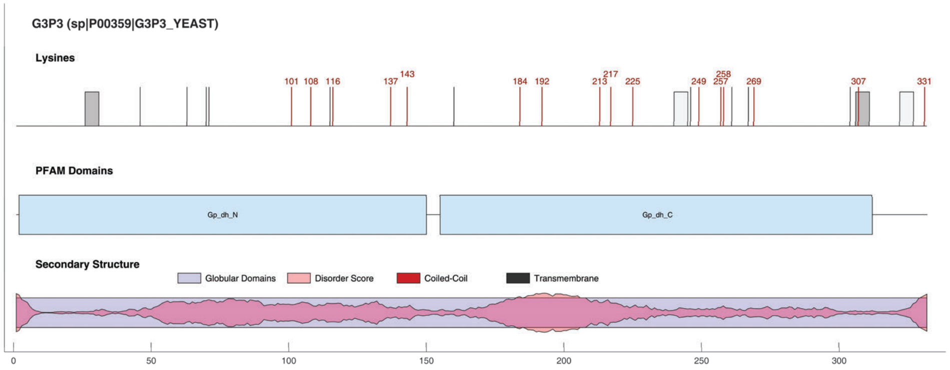
G3P3 (sp|P00359|G3P3_YEAST)
Lysines
101 108 116 137 143 184 192 213 217 225 249 257 258 269 307 331
PFAM Domains
Gp_dh_N
Gp_dh_C
Secondary Structure
Globular Domains
Disorder Score
Coiled-Coil
Transmembrane
0 50 100 150 200 250 300

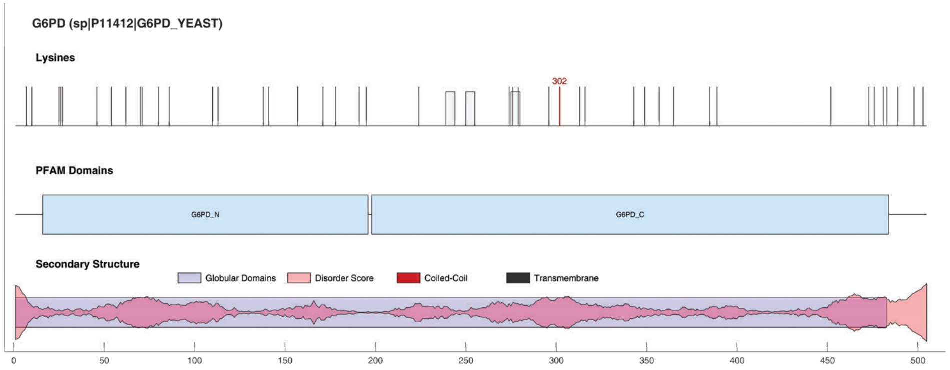
G6PD (sp|P11412|G6PD_YEAST)
Lysines
302
PFAM Domains
G6PD_N
G6PD_C
Secondary Structure
Globular Domains
Disorder Score
Coiled-Coil
Transmembrane
0 50 100 150 200 250 300 350 400 450 500

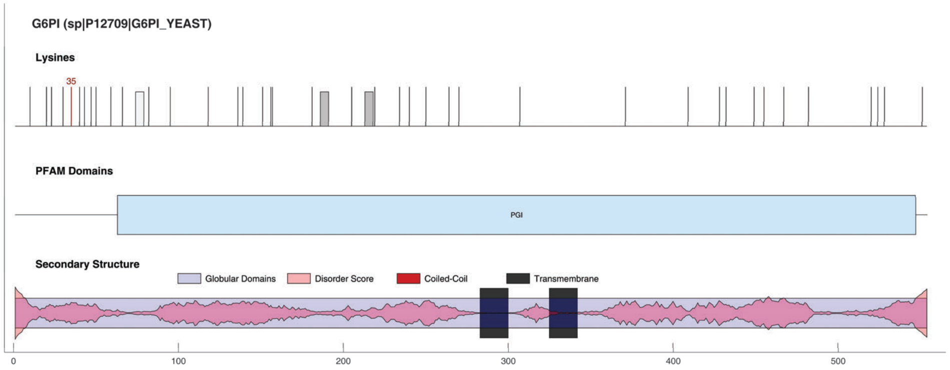
G6PI (sp|P12709|G6PI_YEAST)
Lysines
35
PFAM Domains
PGI
Secondary Structure
Globular Domains
Disorder Score
Coiled-Coil
Transmembrane
0 100 200 300 400 500

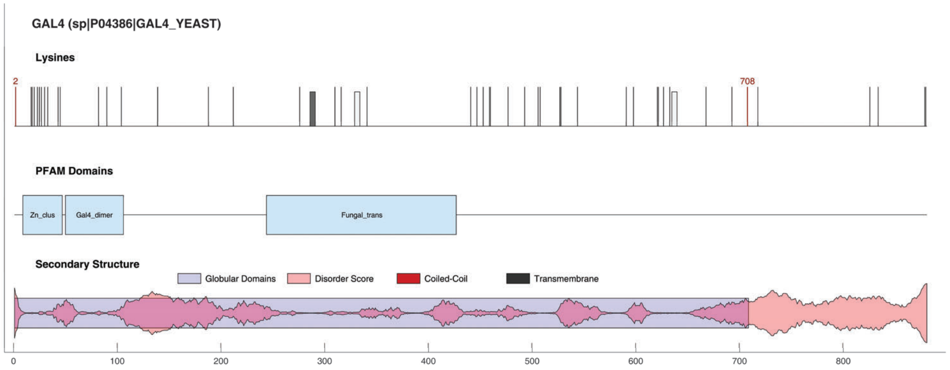
GAL4 (sp|P04386|GAL4_YEAST)
Lysines
2
708
PFAM Domains
Zn_clus
Gal4_dimer
Fungal_trans
Secondary Structure
Globular Domains
Disorder Score
Coiled-Coil
Transmembrane
0 100 200 300 400 500 600 700 800

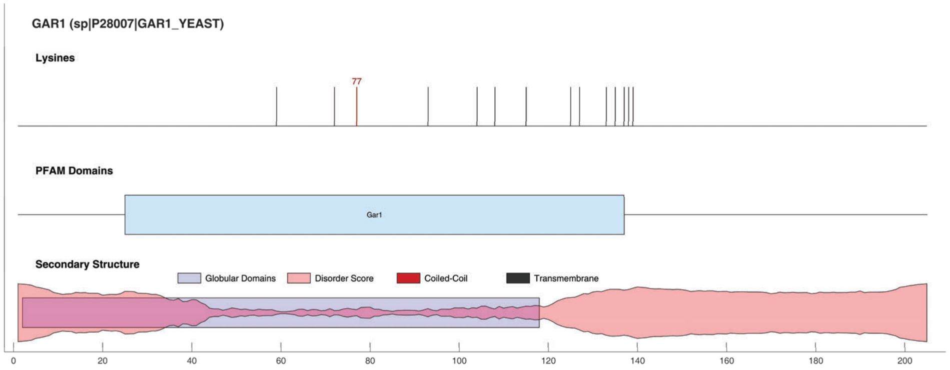
GAR1 (sp|P28007|GAR1_YEAST)
Lysines
77
PFAM Domains
Gar1
Secondary Structure
Globular Domains
Disorder Score
Coiled-Coil
Transmembrane
0 20 40 60 80 100 120 140 160 180 200

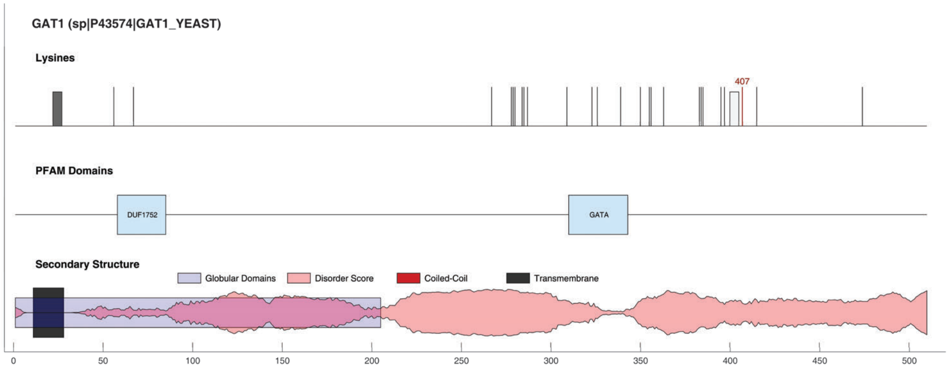
GAT1 (sp|P43574|GAT1_YEAST)
Lysines
407
PFAM Domains
DUF1752
GATA
Secondary Structure
Globular Domains
Disorder Score
Coiled-Coil
Transmembrane
0 50 100 150 200 250 300 350 400 450 500

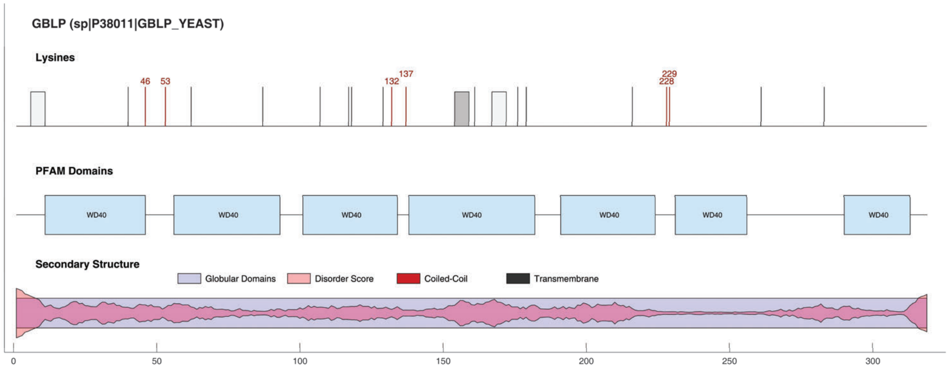
GBLP (sp|P38011|GBLP_YEAST)
Lysines
46 53 132 137 228 229
PFAM Domains
WD40
WD40
WD40
WD40
WD40
WD40
WD40
Secondary Structure
Globular Domains
Disorder Score
Coiled-Coil
Transmembrane
0 50 100 150 200 250 300

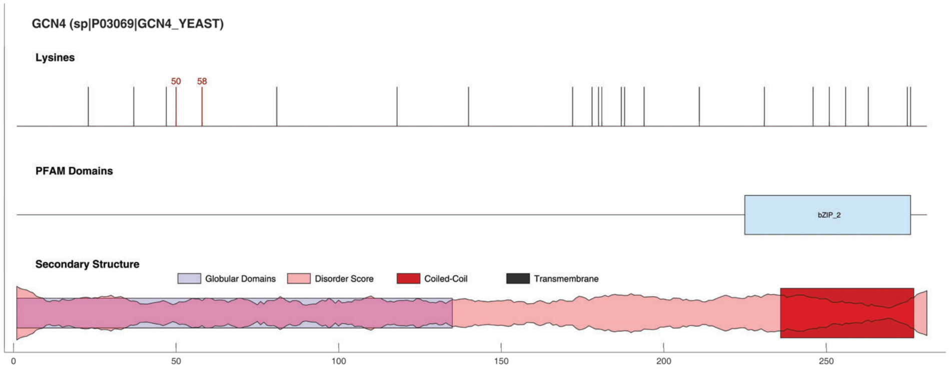
GCN4 (sp|P03069|GCN4_YEAST)
Lysines
50 58
PFAM Domains
bZIP_2
Secondary Structure
Globular Domains
Disorder Score
Coiled-Coil
Transmembrane
0 50 100 150 200 250

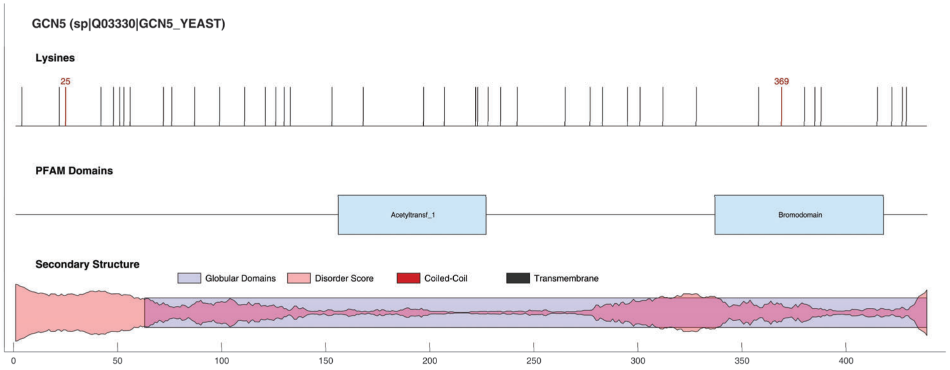

GCN5 (sp|Q03330|GCN5_YEAST)
Lysines
25
369
PFAM Domains
Acetyltransf_1
Bromodomain
Secondary Structure
Globular Domains
Disorder Score
Coiled-Coil
Transmembrane
0 50 100 150 200 250 300 350 400

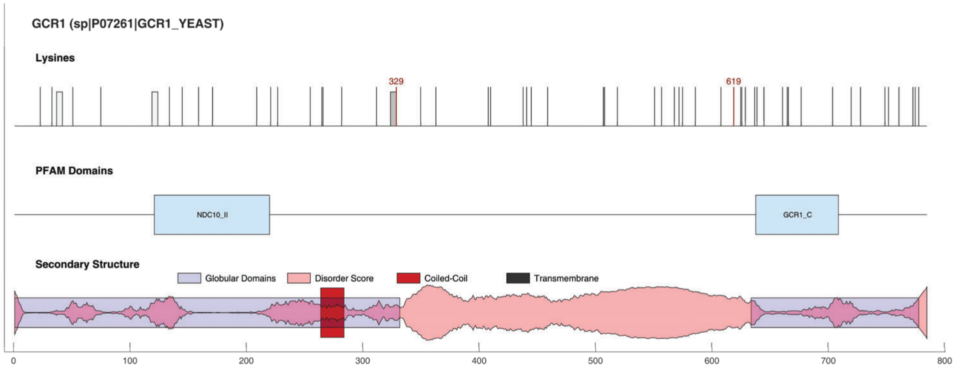

GCR1 (sp|P07261|GCR1_YEAST)
Lysines
329
619
PFAM Domains
NDC10_II
GCR1_C
Secondary Structure
Globular Domains
Disorder Score
Coiled-Coil
Transmembrane
0 100 200 300 400 500 600 700 800

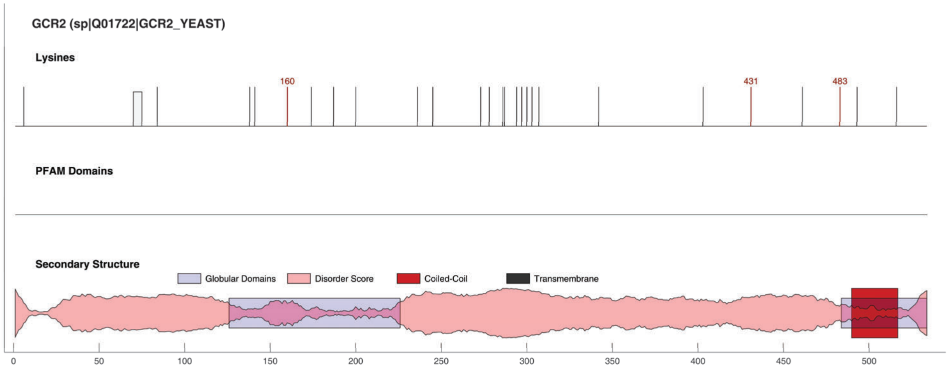

GCR2 (sp|Q01722|GCR2_YEAST)
Lysines
160
431
483
PFAM Domains
Secondary Structure
Globular Domains
Disorder Score
Coiled-Coil
Transmembrane
0 50 100 150 200 250 300 350 400 450 500

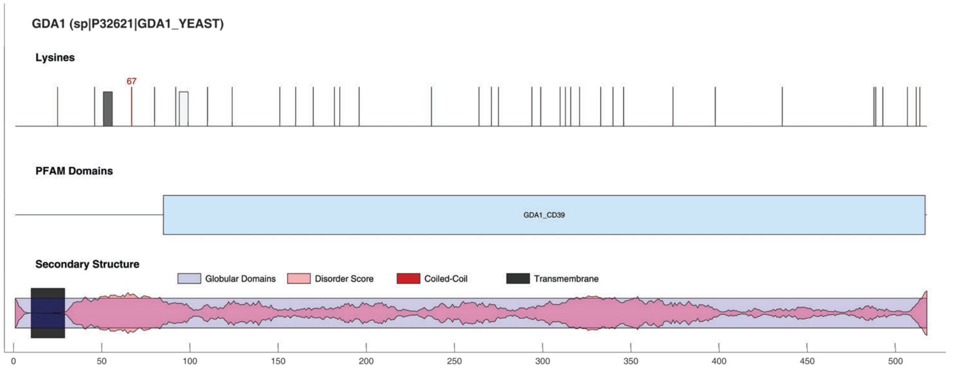

GDA1 (sp|P32621|GDA1_YEAST)
Lysines
67
PFAM Domains
GDA1_CD39
Secondary Structure
Globular Domains
Disorder Score
Coiled-Coil
Transmembrane
0 50 100 150 200 250 300 350 400 450 500

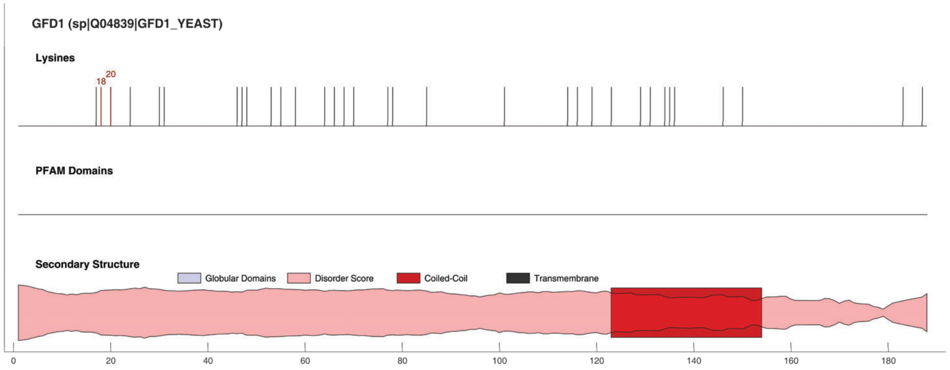
GFD1 (sp|Q04839|GFD1_YEAST)
Lysines
18
20
PFAM Domains
Secondary Structure
Globular Domains
Disorder Score
Coiled-Coil
Transmembrane
0
20
40
60
80
100
120
140
160
180

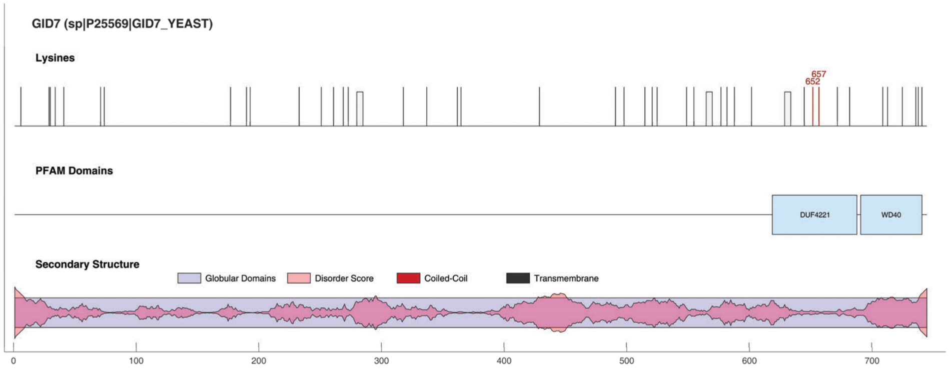
GID7 (sp|P25569|GID7_YEAST)
Lysines
652
657
PFAM Domains
DUF4221
WD40
Secondary Structure
Globular Domains
Disorder Score
Coiled-Coil
Transmembrane
0
100
200
300
400
500
600
700

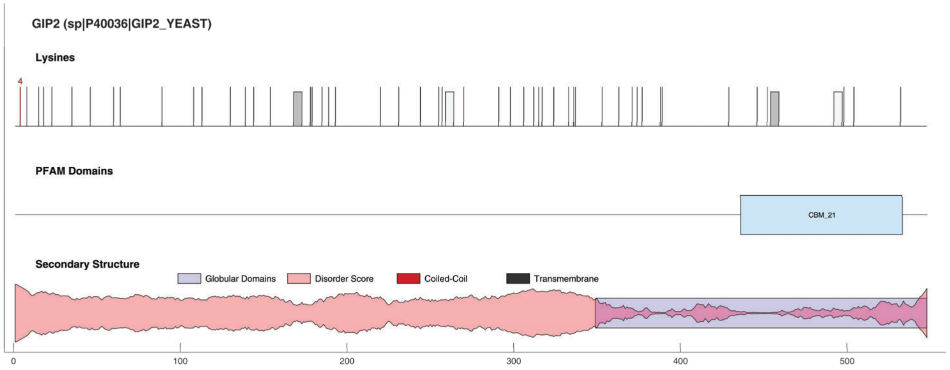
GIP2 (sp|P40036|GIP2_YEAST)
Lysines
4
PFAM Domains
CBM_21
Secondary Structure
Globular Domains
Disorder Score
Coiled-Coil
Transmembrane
0
100
200
300
400
500

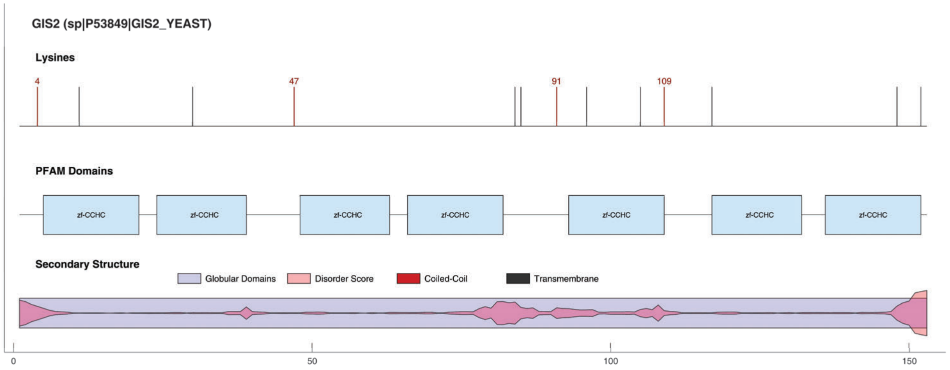
GIS2 (sp|P53849|GIS2_YEAST)
Lysines
4
47
91
109
PFAM Domains
zf-CCHC
zf-CCHC
zf-CCHC
zf-CCHC
zf-CCHC
zf-CCHC
zf-CCHC
Secondary Structure
Globular Domains
Disorder Score
Coiled-Coil
Transmembrane
0
50
100
150

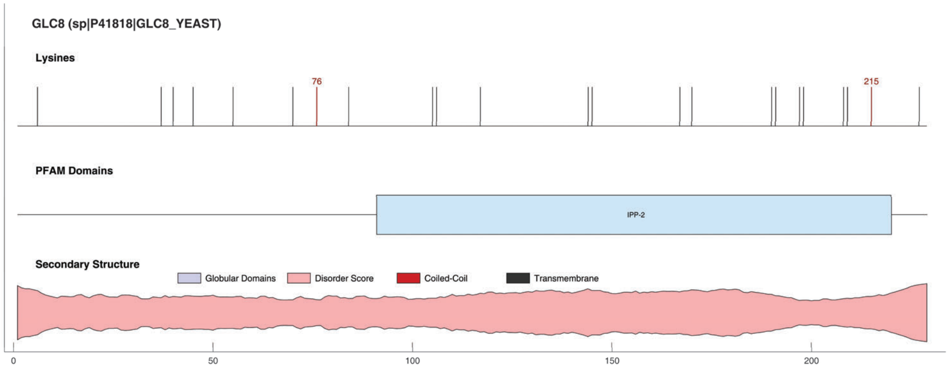

GLC8 (sp|P41818|GLC8_YEAST)
Lysines
76
215
PFAM Domains
IPP-2
Secondary Structure
Globular Domains
Disorder Score
Coiled-Coil
Transmembrane
0
50
100
150
200

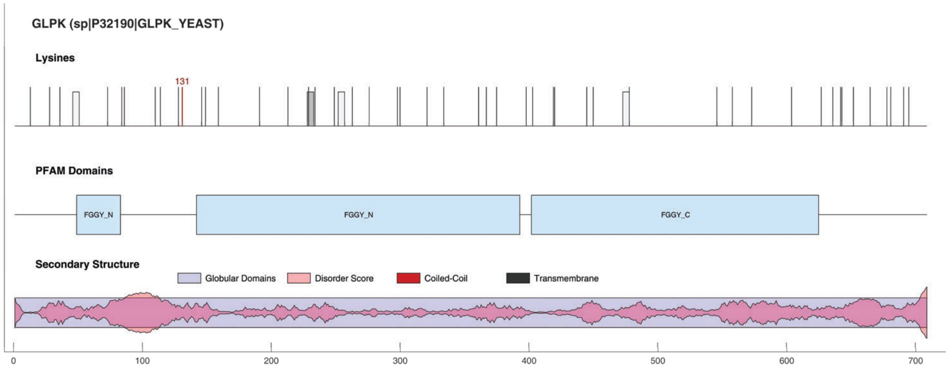

GLPK (sp|P32190|GLPK_YEAST)
Lysines
131
PFAM Domains
FGGY_N
FGGY_N
FGGY_C
Secondary Structure
Globular Domains
Disorder Score
Coiled-Coil
Transmembrane
0
100
200
300
400
500
600
700

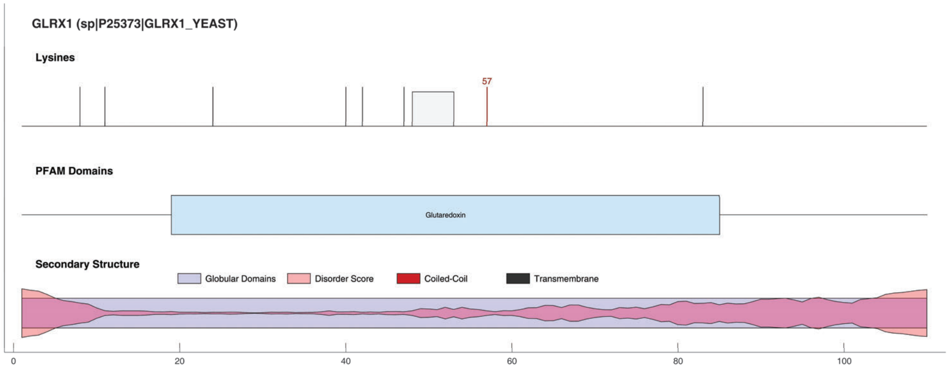

GLRX1 (sp|P25373|GLRX1_YEAST)
Lysines
57
PFAM Domains
Glutaredoxin
Secondary Structure
Globular Domains
Disorder Score
Coiled-Coil
Transmembrane
0
20
40
60
80
100

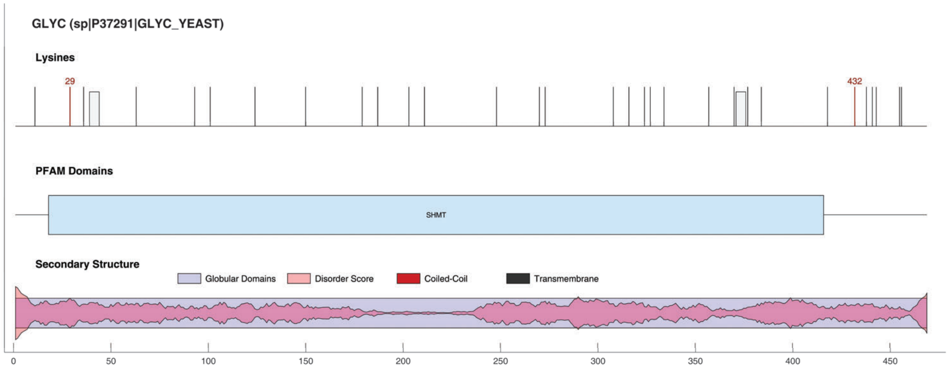

GLYC (sp|P37291|GLYC_YEAST)
Lysines
29
432
PFAM Domains
SHMT
Secondary Structure
Globular Domains
Disorder Score
Coiled-Coil
Transmembrane
0
50
100
150
200
250
300
350
400
450

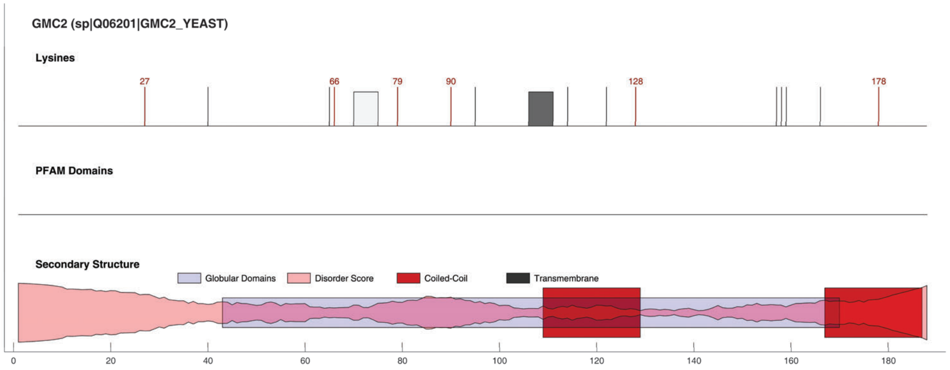
GMC2 (sp|Q06201|GMC2_YEAST)
Lysines
27
66
79
90
128
178
PFAM Domains
Secondary Structure
Globular Domains
Disorder Score
Coiled-Coil
Transmembrane
0
20
40
60
80
100
120
140
160
180

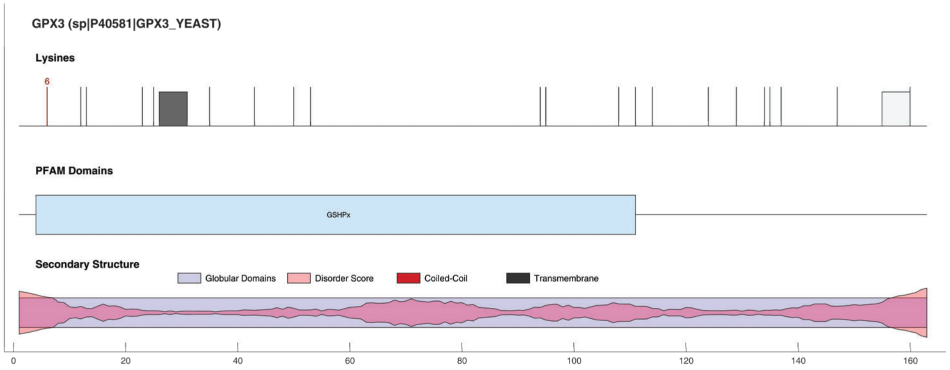
GPX3 (sp|P40581|GPX3_YEAST)
Lysines
6
PFAM Domains
GSHPx
Secondary Structure
Globular Domains
Disorder Score
Coiled-Coil
Transmembrane
0
20
40
60
80
100
120
140
160

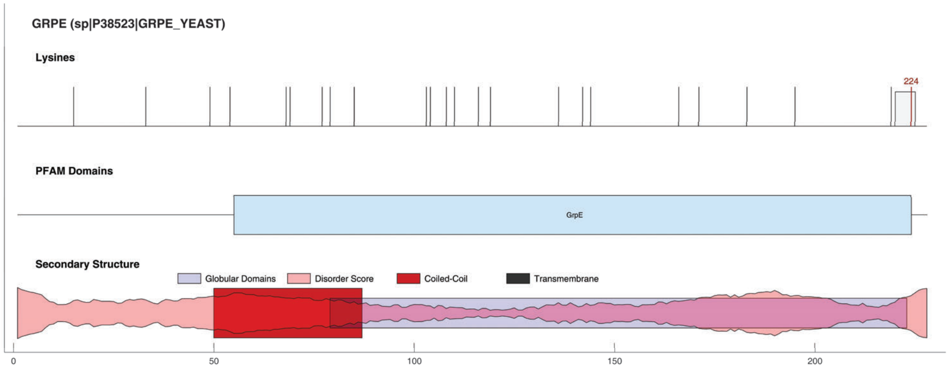
GRPE (sp|P38523|GRPE_YEAST)
Lysines
224
PFAM Domains
GrpE
Secondary Structure
Globular Domains
Disorder Score
Coiled-Coil
Transmembrane
0
50
100
150
200

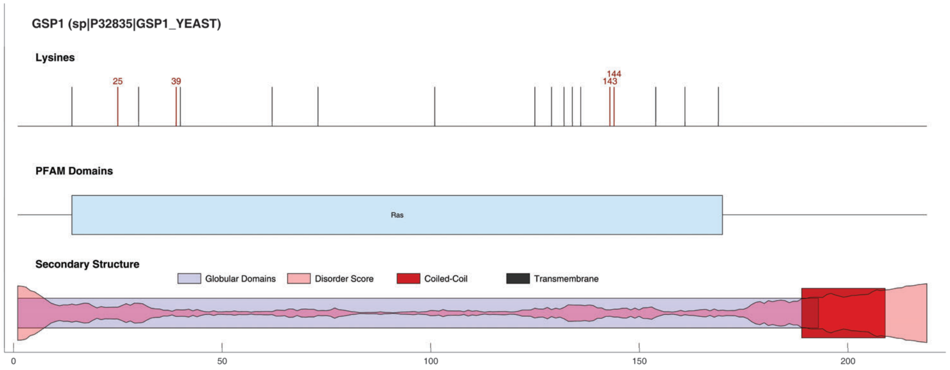
GSP1 (sp|P32835|GSP1_YEAST)
Lysines
25
39
143
144
PFAM Domains
Ras
Secondary Structure
Globular Domains
Disorder Score
Coiled-Coil
Transmembrane
0
50
100
150
200

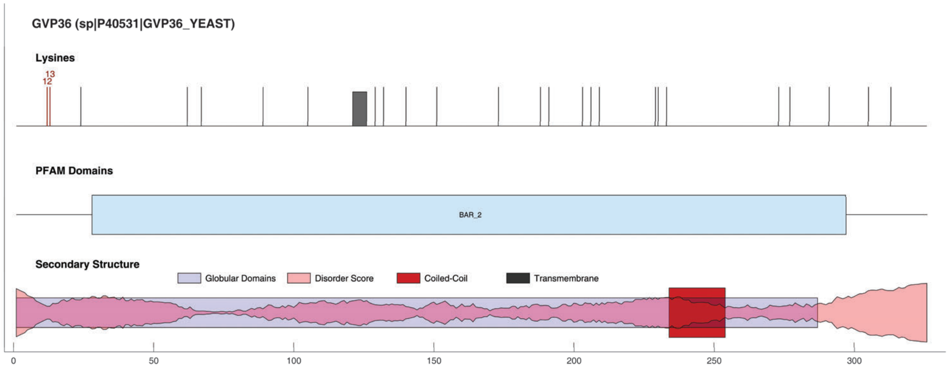
GVP36 (sp|P40531|GVP36_YEAST)
Lysines
12
13
PFAM Domains
BAR_2
Secondary Structure
Globular Domains
Disorder Score
Coiled-Coil
Transmembrane
0
50
100
150
200
250
300

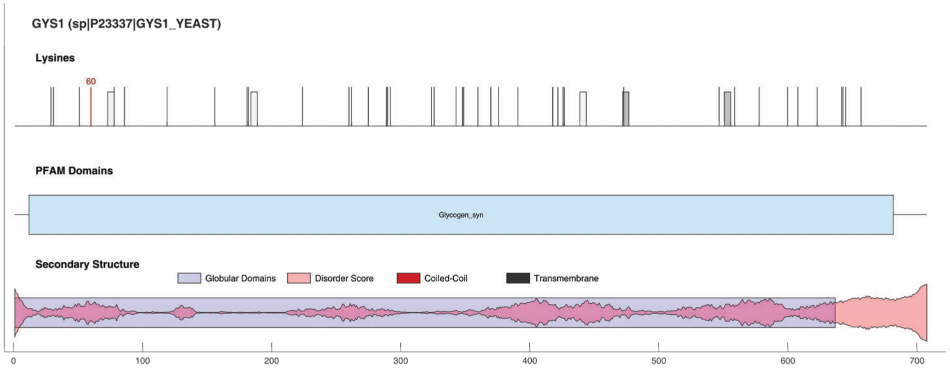
GYS1 (sp|P23337|GYS1_YEAST)
Lysines
60
PFAM Domains
Glycogen_syn
Secondary Structure
Globular Domains
Disorder Score
Coiled-Coil
Transmembrane
0
100
200
300
400
500
600
700

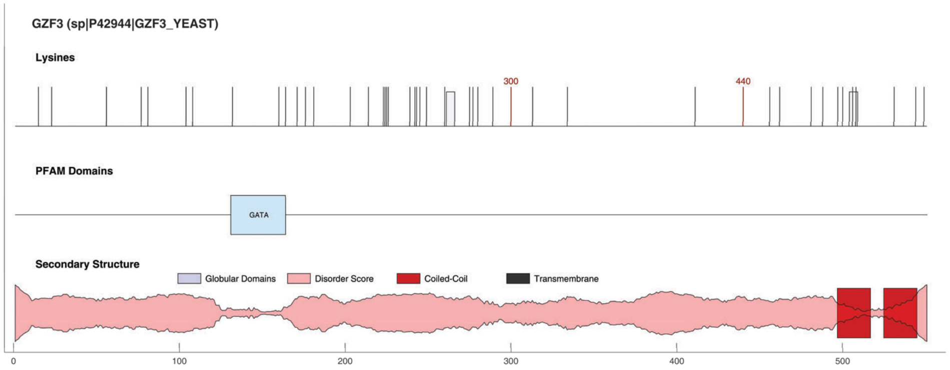
GZF3 (sp|P42944|GZF3_YEAST)
Lysines
300
440
PFAM Domains
GATA
Secondary Structure
Globular Domains
Disorder Score
Coiled-Coil
Transmembrane
0
100
200
300
400
500

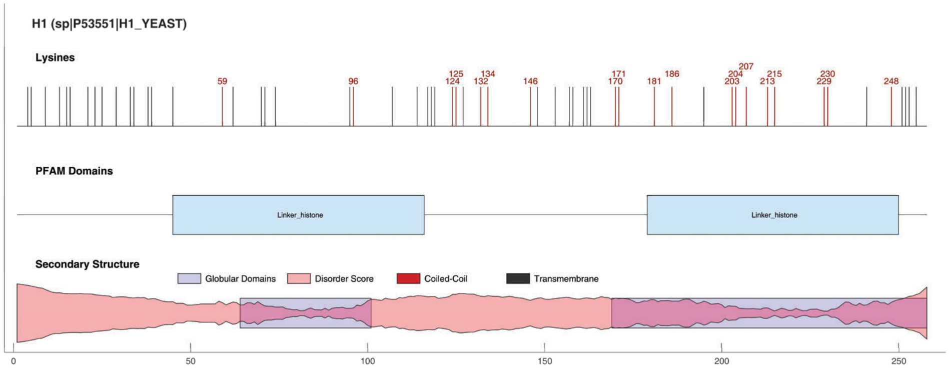
H1 (sp|P53551|H1_YEAST)
Lysines
59
96
124
125
132
134
146
170
171
181
186
203
204
207
213
215
229
230
248
PFAM Domains
Linker_histone
Linker_histone
Secondary Structure
Globular Domains
Disorder Score
Coiled-Coil
Transmembrane
0
50
100
150
200
250

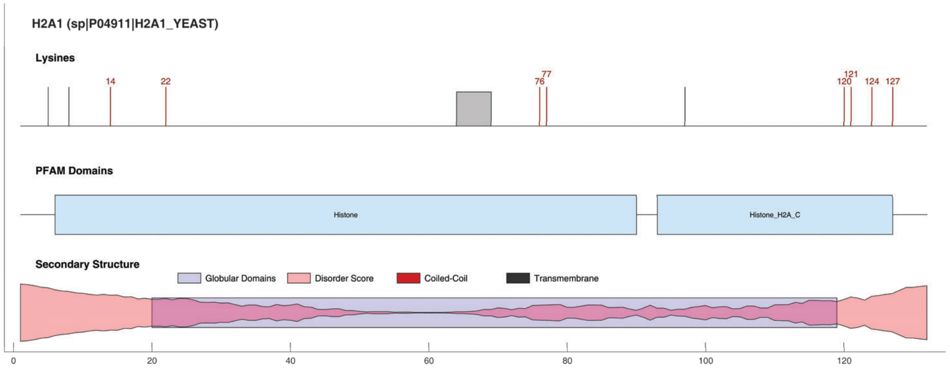
H2A1 (sp|P04911|H2A1_YEAST)
Lysines
14
22
76
77
120
121
124
127
PFAM Domains
Histone
Histone_H2A_C
Secondary Structure
Globular Domains
Disorder Score
Coiled-Coil
Transmembrane
0
20
40
60
80
100
120

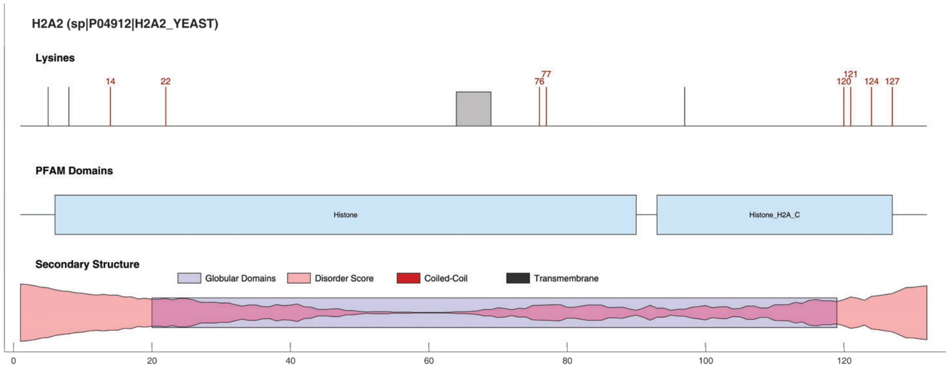
H2A2 (sp|P04912|H2A2_YEAST)
Lysines
14
22
76
77
120
121
124
127
PFAM Domains
Histone
Histone_H2A_C
Secondary Structure
Globular Domains
Disorder Score
Coiled-Coil
Transmembrane
0
20
40
60
80
100
120

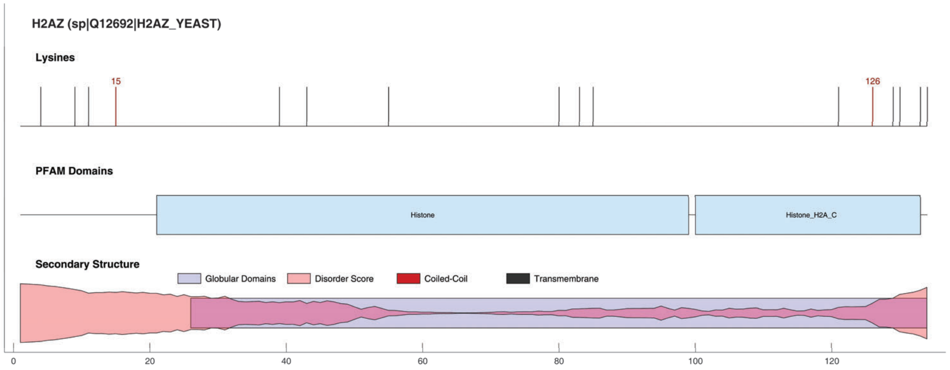
H2AZ (sp|Q12692|H2AZ_YEAST)
Lysines
15
126
PFAM Domains
Histone
Histone_H2A_C
Secondary Structure
Globular Domains
Disorder Score
Coiled-Coil
Transmembrane
0
20
40
60
80
100
120

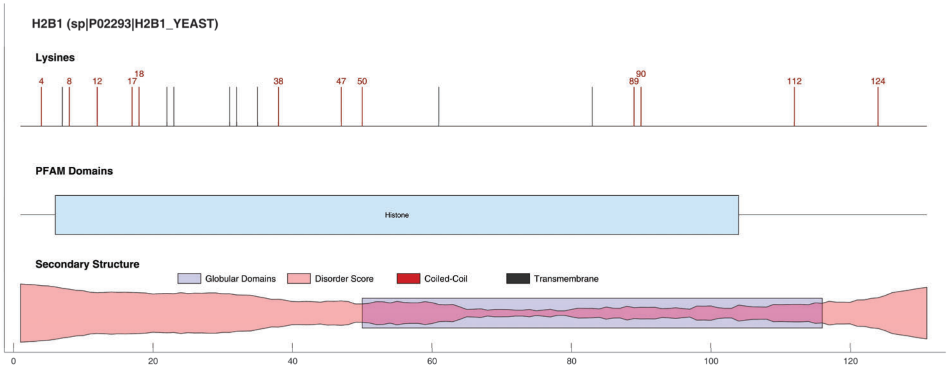
H2B1 (sp|P02293|H2B1_YEAST)
Lysines
4
8
12
17
18
38
47
50
89
90
112
124
PFAM Domains
Histone
Secondary Structure
Globular Domains
Disorder Score
Coiled-Coil
Transmembrane
0
20
40
60
80
100
120

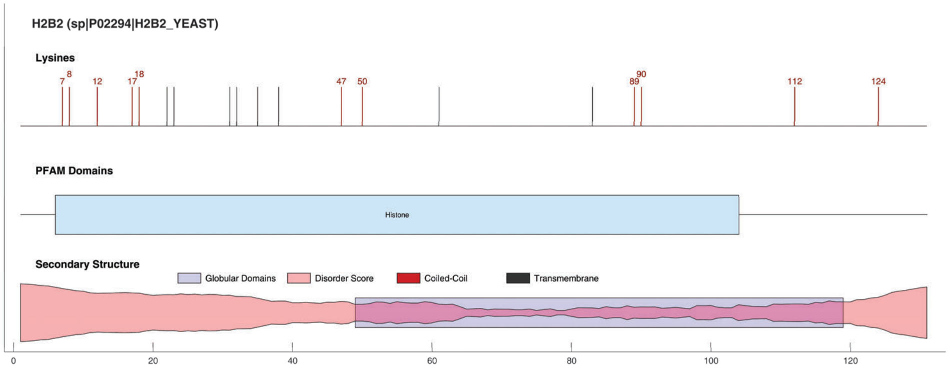
H2B2 (sp|P02294|H2B2_YEAST)
Lysines
7 8 12 17 18 47 50 89 90 112 124
PFAM Domains
Histone
Secondary Structure
Globular Domains
Disorder Score
Coiled-Coil
Transmembrane
0 20 40 60 80 100 120

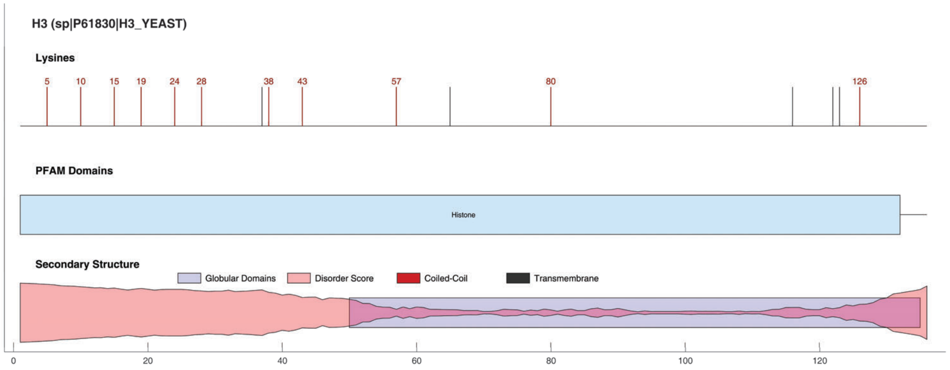
H3 (sp|P61830|H3_YEAST)
Lysines
5 10 15 19 24 28 38 43 57 80 126
PFAM Domains
Histone
Secondary Structure
Globular Domains
Disorder Score
Coiled-Coil
Transmembrane
0 20 40 60 80 100 120

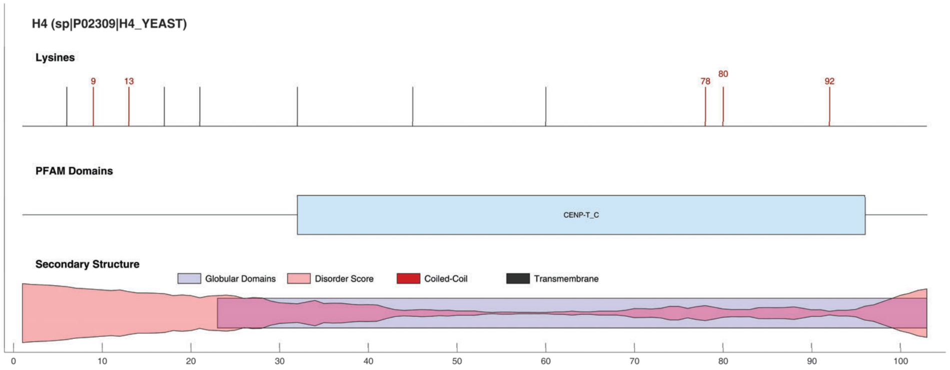
H4 (sp|P02309|H4_YEAST)
Lysines
9 13 78 80 92
PFAM Domains
CENP-T_C
Secondary Structure
Globular Domains
Disorder Score
Coiled-Coil
Transmembrane
0 10 20 30 40 50 60 70 80 90 100

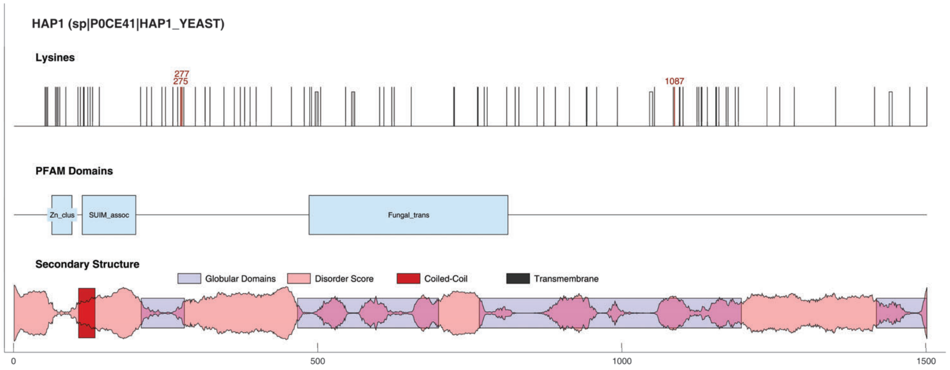
HAP1 (sp|P0CE41|HAP1_YEAST)
Lysines
275 277 1087
PFAM Domains
Zn_clus
SUIM_assoc
Fungal_trans
Secondary Structure
Globular Domains
Disorder Score
Coiled-Coil
Transmembrane
0 500 1000 1500

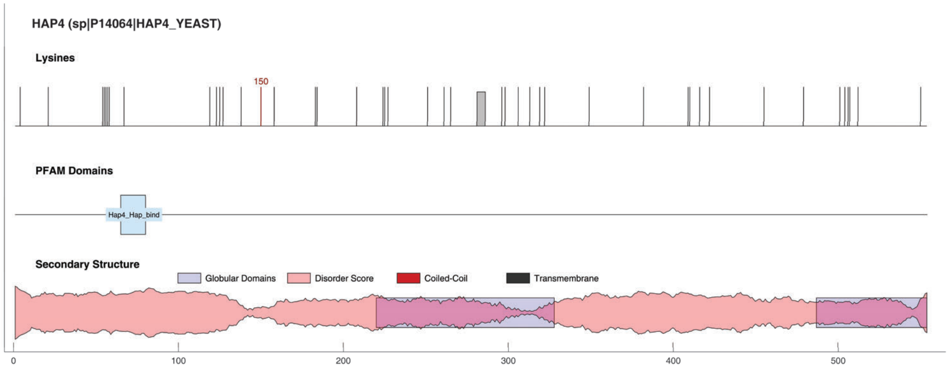
HAP4 (sp|P14064|HAP4_YEAST)
Lysines
150
PFAM Domains
Hap4_Hap_bind
Secondary Structure
Globular Domains
Disorder Score
Coiled-Coil
Transmembrane
0
100
200
300
400
500

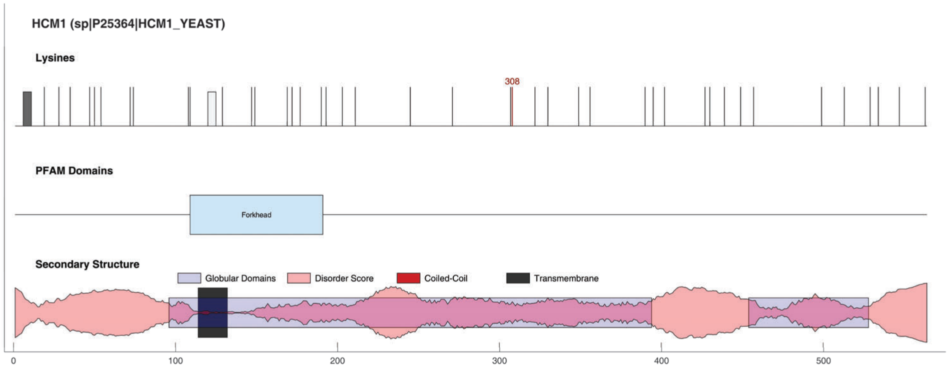
HCM1 (sp|P25364|HCM1_YEAST)
Lysines
308
PFAM Domains
Forkhead
Secondary Structure
Globular Domains
Disorder Score
Coiled-Coil
Transmembrane
0
100
200
300
400
500

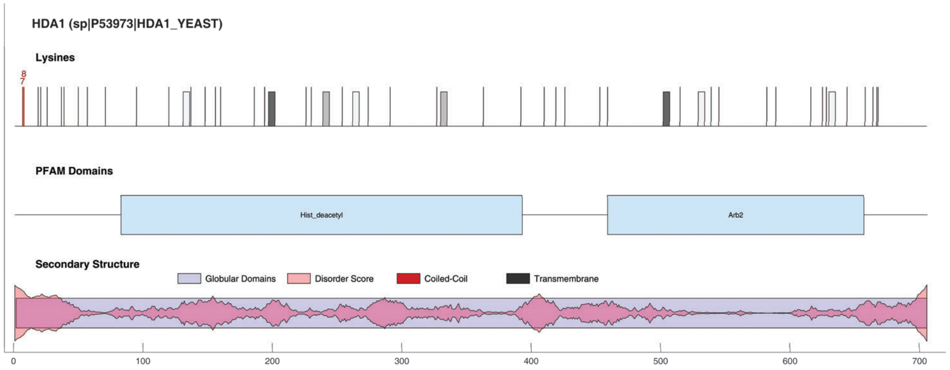
HDA1 (sp|P53973|HDA1_YEAST)
Lysines
8
7
PFAM Domains
Hist_deacetyl
Arb2
Secondary Structure
Globular Domains
Disorder Score
Coiled-Coil
Transmembrane
0
100
200
300
400
500
600
700

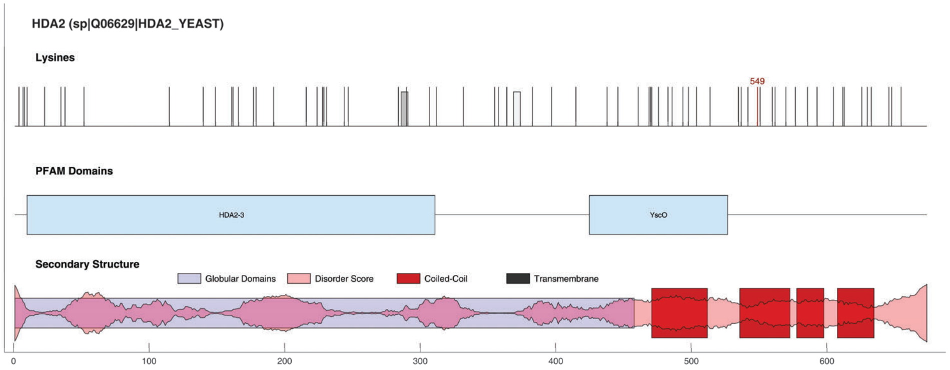
HDA2 (sp|Q06629|HDA2_YEAST)
Lysines
549
PFAM Domains
HDA2-3
YscO
Secondary Structure
Globular Domains
Disorder Score
Coiled-Coil
Transmembrane
0
100
200
300
400
500
600

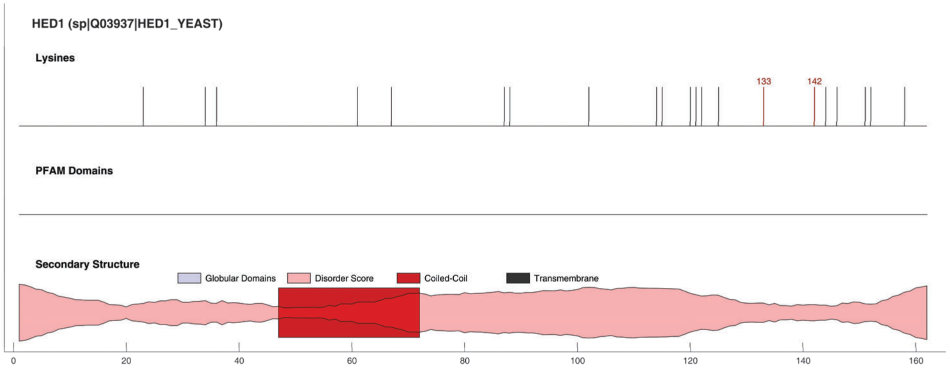
HED1 (sp|Q03937|HED1_YEAST)
Lysines
133
142
PFAM Domains
Secondary Structure
Globular Domains
Disorder Score
Coiled-Coil
Transmembrane
0
20
40
60
80
100
120
140
160

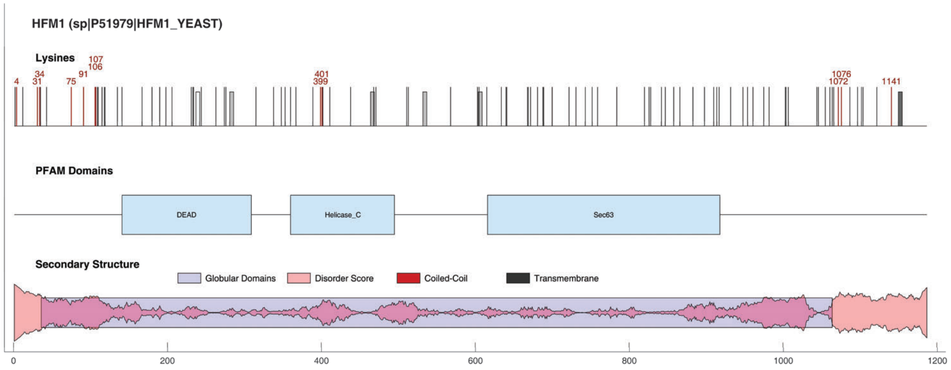
HFM1 (sp|P51979|HFM1_YEAST)
Lysines
4
34
31
75
91
107
106
401
399
1076
1072
1141
PFAM Domains
DEAD
Helicase_C
Sec63
Secondary Structure
Globular Domains
Disorder Score
Coiled-Coil
Transmembrane
0
200
400
600
800
1000
1200

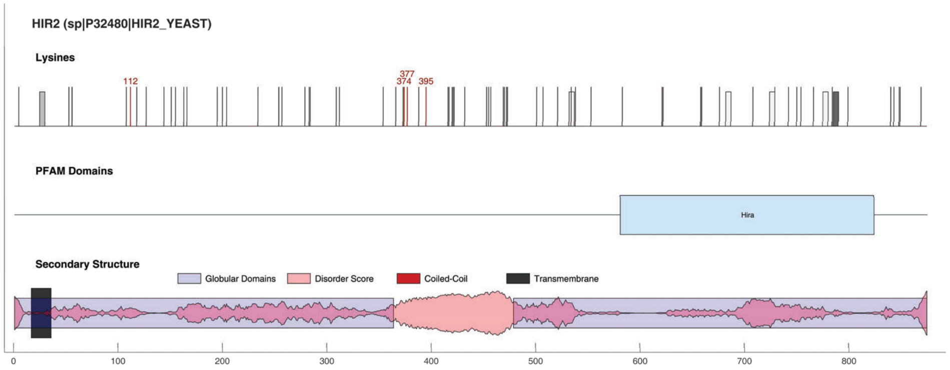
HIR2 (sp|P32480|HIR2_YEAST)
Lysines
112
377
374
395
PFAM Domains
Hira
Secondary Structure
Globular Domains
Disorder Score
Coiled-Coil
Transmembrane
0
100
200
300
400
500
600
700
800

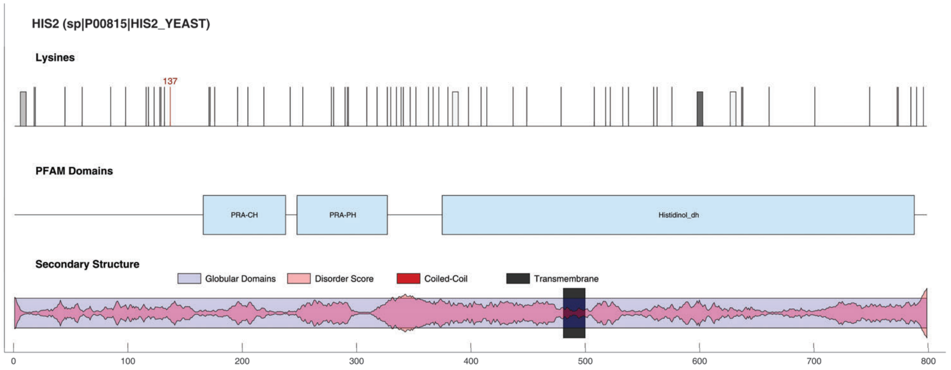
HIS2 (sp|P00815|HIS2_YEAST)
Lysines
137
PFAM Domains
PRA-CH
PRA-PH
Histidinol_dh
Secondary Structure
Globular Domains
Disorder Score
Coiled-Coil
Transmembrane
0
100
200
300
400
500
600
700
800

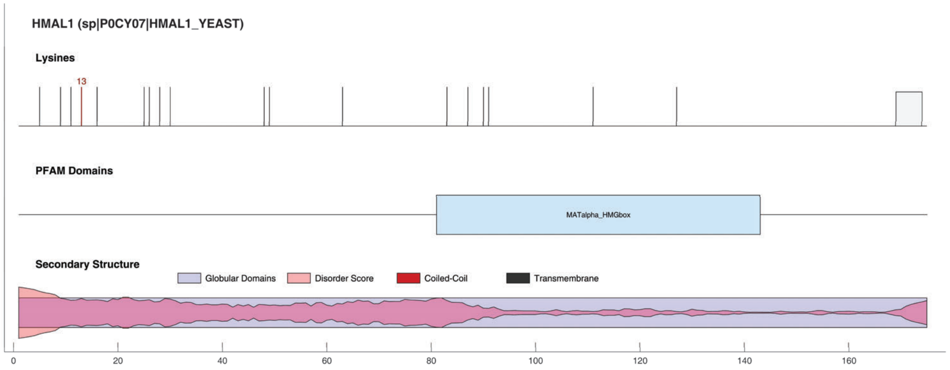
HMAL1 (sp|P0CY07|HMAL1_YEAST)
Lysines
13
PFAM Domains
MATalpha_HMGbox
Secondary Structure
Globular Domains
Disorder Score
Coiled-Coil
Transmembrane
0
20
40
60
80
100
120
140
160

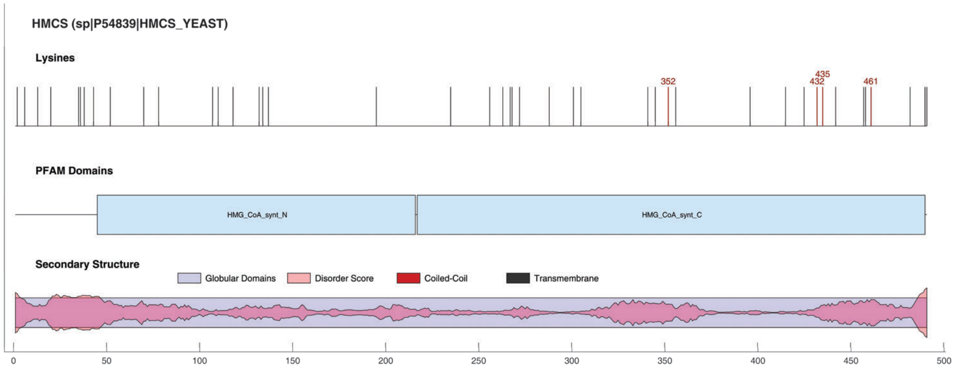
HMCS (sp|P54839|HMCS_YEAST)
Lysines
352
432
435
461
PFAM Domains
HMG_CoA_synt_N
HMG_CoA_synt_C
Secondary Structure
Globular Domains
Disorder Score
Coiled-Coil
Transmembrane
0
50
100
150
200
250
300
350
400
450
500

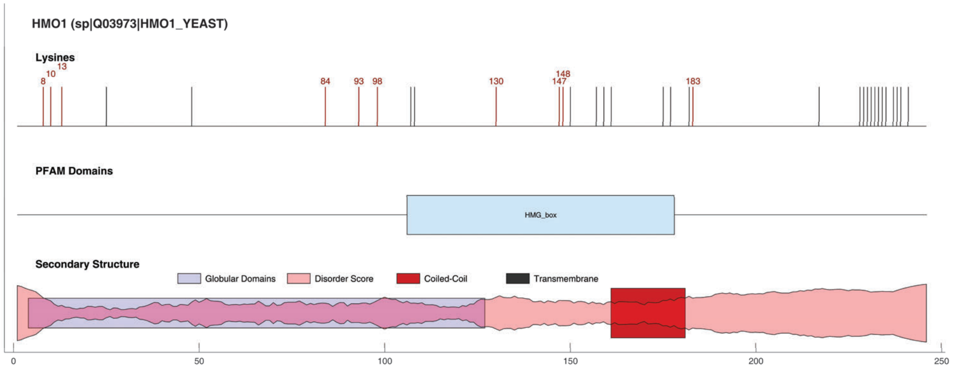
HMO1 (sp|Q03973|HMO1_YEAST)
Lysines
8
10
13
84
93
98
130
147
148
183
PFAM Domains
HMG_box
Secondary Structure
Globular Domains
Disorder Score
Coiled-Coil
Transmembrane
0
50
100
150
200
250

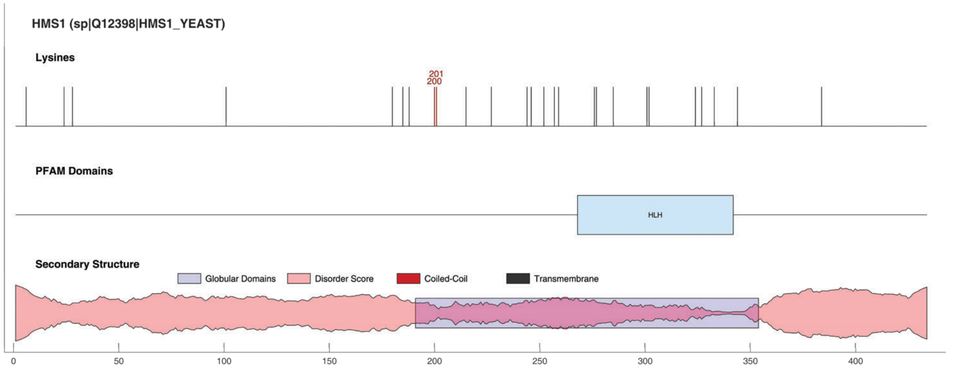
HMS1 (sp|Q12398|HMS1_YEAST)
Lysines
200
201
PFAM Domains
HLH
Secondary Structure
Globular Domains
Disorder Score
Coiled-Coil
Transmembrane
0
50
100
150
200
250
300
350
400

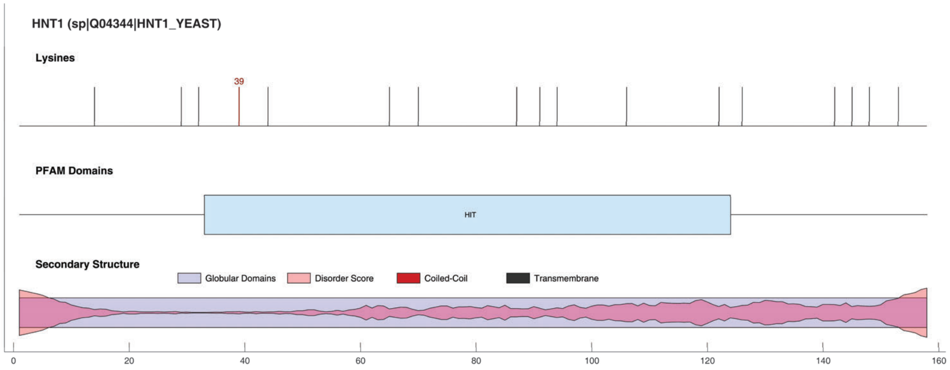
HNT1 (sp|Q04344|HNT1_YEAST)
Lysines
39
PFAM Domains
HIT
Secondary Structure
Globular Domains
Disorder Score
Coiled-Coil
Transmembrane
0 20 40 60 80 100 120 140 160

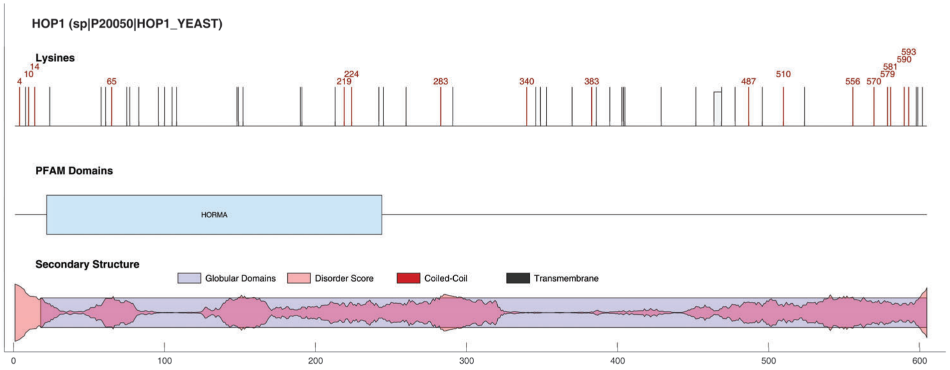
HOP1 (sp|P20050|HOP1_YEAST)
Lysines
4 10 14 65 219 224 283 340 383 487 510 556 570 579 581 590 593
PFAM Domains
HORMA
Secondary Structure
Globular Domains
Disorder Score
Coiled-Coil
Transmembrane
0 100 200 300 400 500 600

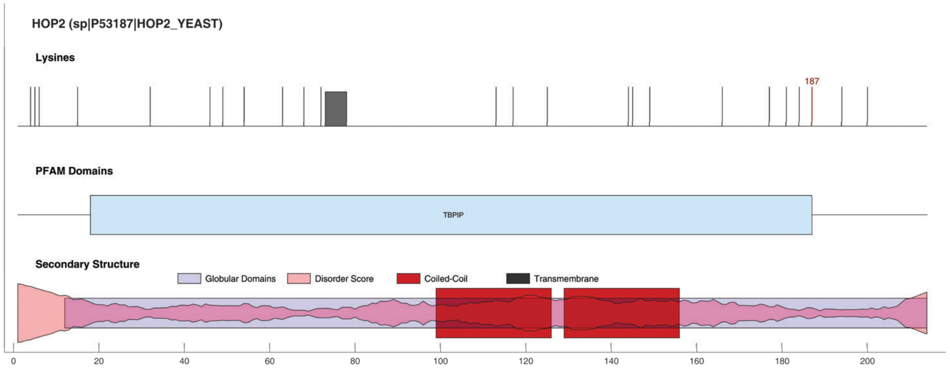
HOP2 (sp|P53187|HOP2_YEAST)
Lysines
187
PFAM Domains
TBPIP
Secondary Structure
Globular Domains
Disorder Score
Coiled-Coil
Transmembrane
0 20 40 60 80 100 120 140 160 180 200

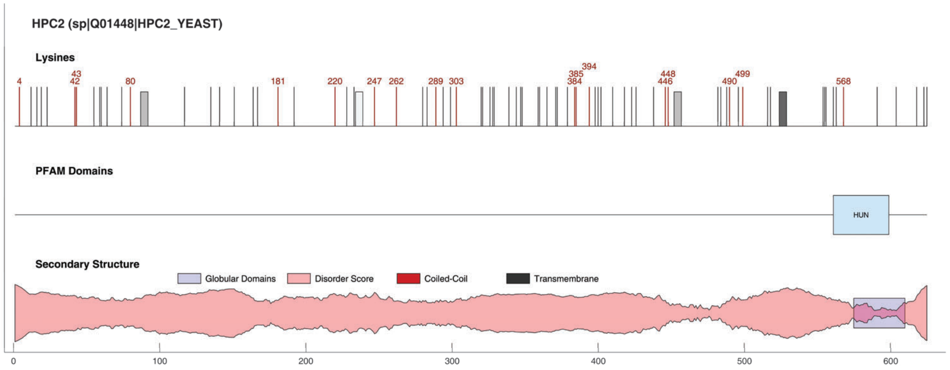
HPC2 (sp|Q01448|HPC2_YEAST)
Lysines
4 42 43 80 181 220 247 262 289 303 384 385 394 446 448 490 499 568
PFAM Domains
HUN
Secondary Structure
Globular Domains
Disorder Score
Coiled-Coil
Transmembrane
0 100 200 300 400 500 600

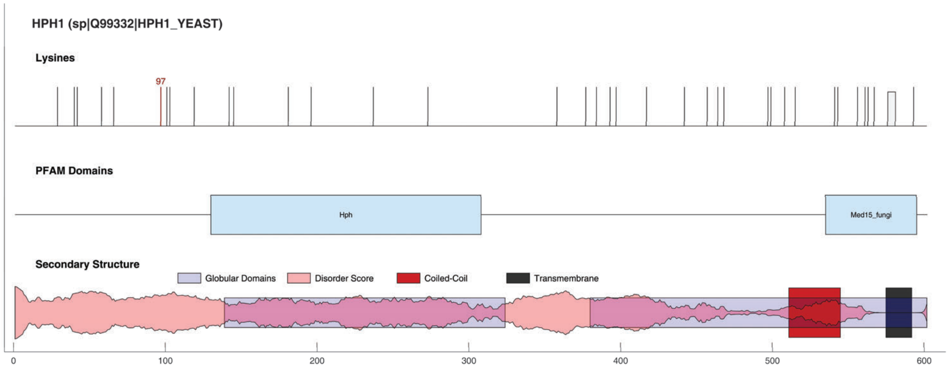
HPH1 (sp|Q99332|HPH1_YEAST)
Lysines
97
PFAM Domains
Hph
Med15_fungi
Secondary Structure
Globular Domains
Disorder Score
Coiled-Coil
Transmembrane
0
100
200
300
400
500
600

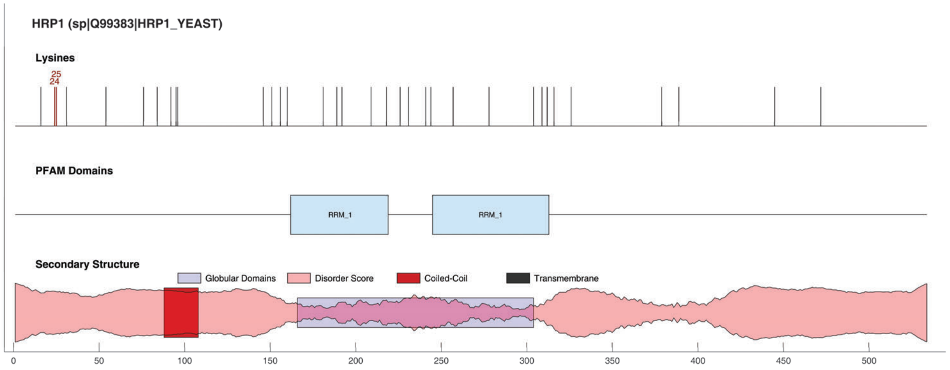
HRP1 (sp|Q99383|HRP1_YEAST)
Lysines
25
24
PFAM Domains
RRM_1
RRM_1
Secondary Structure
Globular Domains
Disorder Score
Coiled-Coil
Transmembrane
0
50
100
150
200
250
300
350
400
450
500

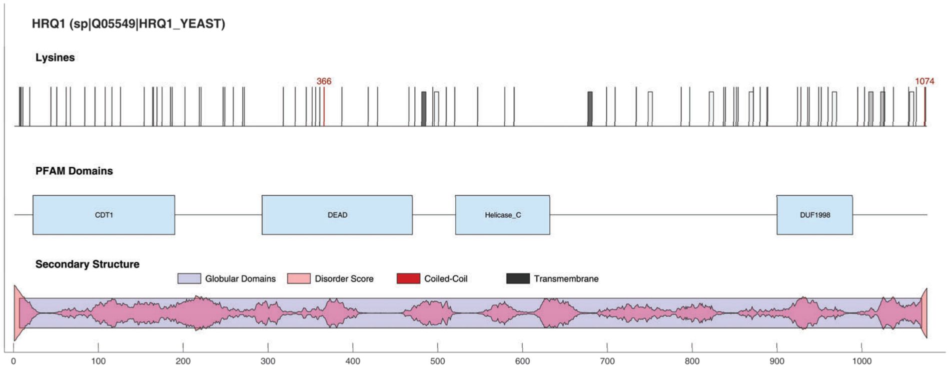
HRQ1 (sp|Q05549|HRQ1_YEAST)
Lysines
366
1074
PFAM Domains
CDT1
DEAD
Helicase_C
DUF1998
Secondary Structure
Globular Domains
Disorder Score
Coiled-Coil
Transmembrane
0
100
200
300
400
500
600
700
800
900
1000

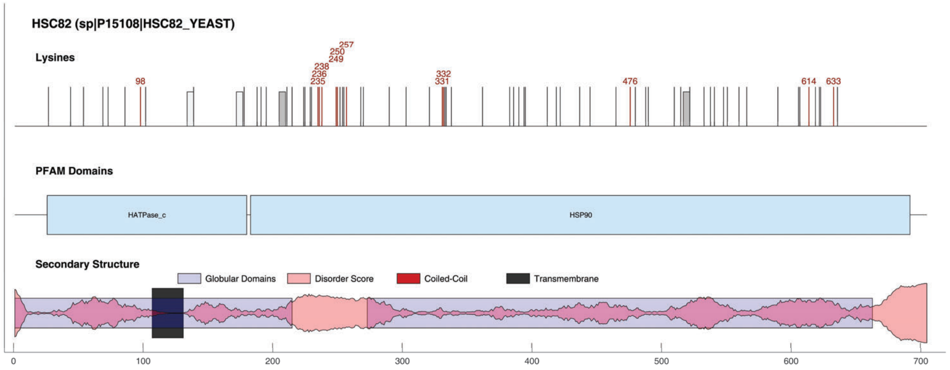
HSC82 (sp|P15108|HSC82_YEAST)
Lysines
98
257
250
249
238
236
235
332
331
476
614
633
PFAM Domains
HATPase_c
HSP90
Secondary Structure
Globular Domains
Disorder Score
Coiled-Coil
Transmembrane
0
100
200
300
400
500
600
700

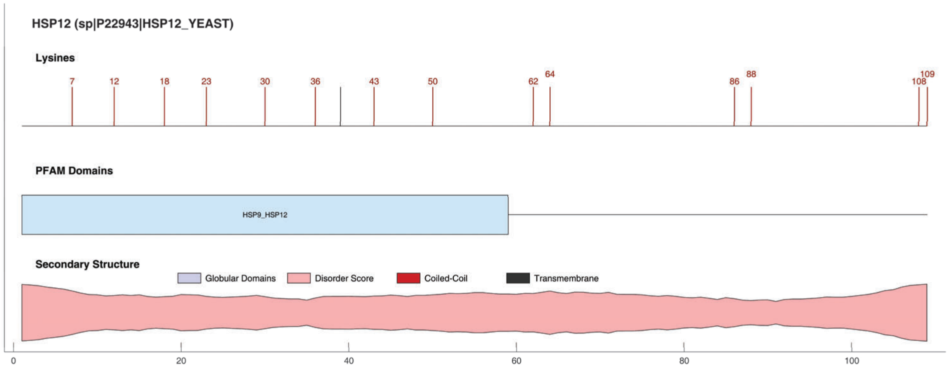
HSP12 (sp|P22943|HSP12_YEAST)
Lysines
7
12
18
23
30
36
43
50
62
64
86
88
108
109
PFAM Domains
HSP9_HSP12
Secondary Structure
Globular Domains
Disorder Score
Coiled-Coil
Transmembrane
0
20
40
60
80
100

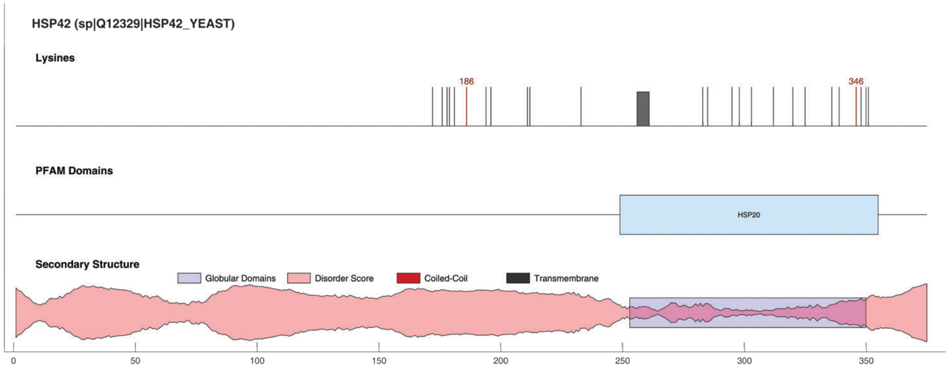
HSP42 (sp|Q12329|HSP42_YEAST)
Lysines
186
346
PFAM Domains
HSP20
Secondary Structure
Globular Domains
Disorder Score
Coiled-Coil
Transmembrane
0
50
100
150
200
250
300
350

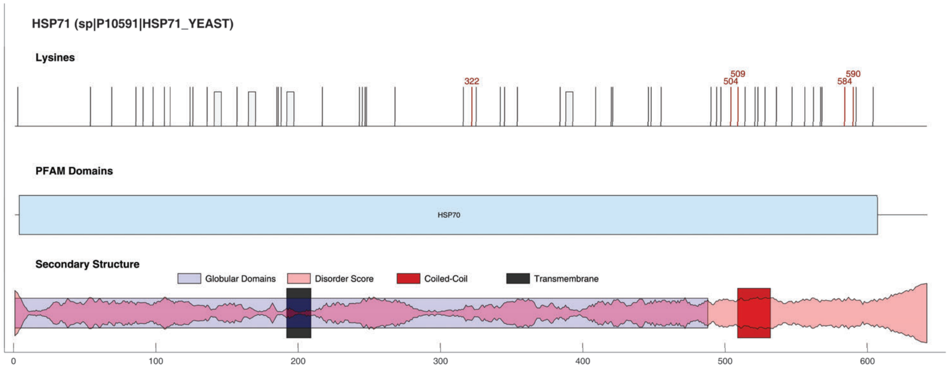
HSP71 (sp|P10591|HSP71_YEAST)
Lysines
322
504
509
584
590
PFAM Domains
HSP70
Secondary Structure
Globular Domains
Disorder Score
Coiled-Coil
Transmembrane
0
100
200
300
400
500
600

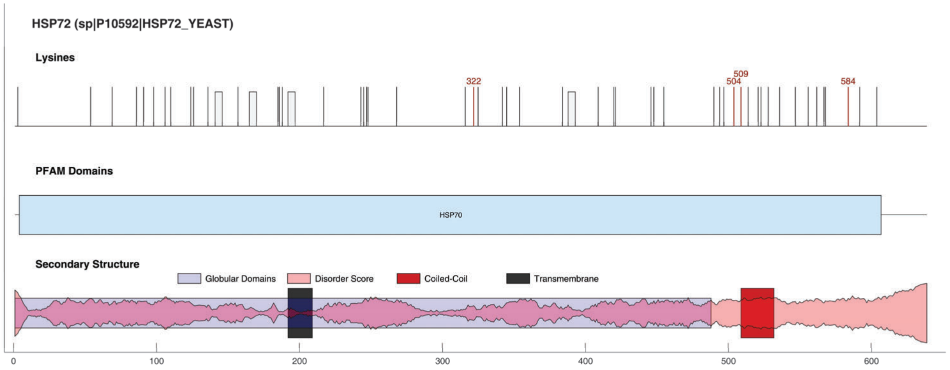
HSP72 (sp|P10592|HSP72_YEAST)
Lysines
322
504
509
584
PFAM Domains
HSP70
Secondary Structure
Globular Domains
Disorder Score
Coiled-Coil
Transmembrane
0
100
200
300
400
500
600

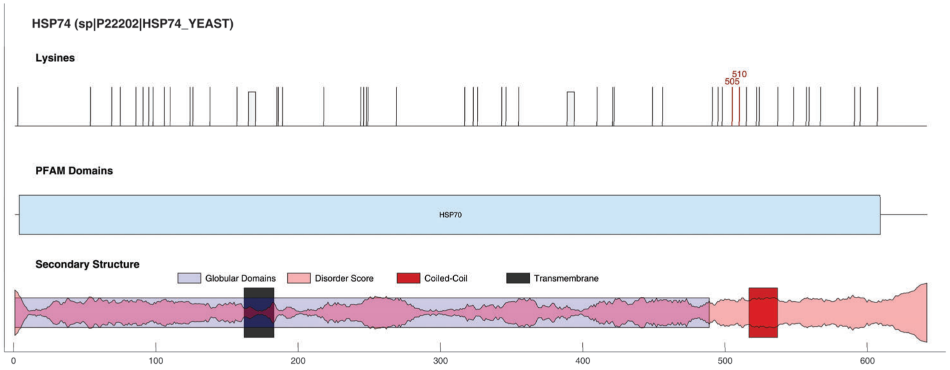
HSP74 (sp|P22202|HSP74_YEAST)
Lysines
510
505
PFAM Domains
HSP70
Secondary Structure
Globular Domains
Disorder Score
Coiled-Coil
Transmembrane
0
100
200
300
400
500
600

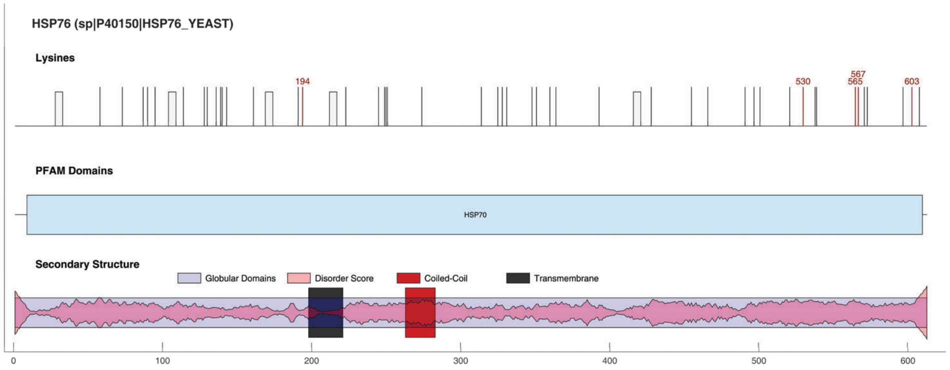
HSP76 (sp|P40150|HSP76_YEAST)
Lysines
194
530
567
565
603
PFAM Domains
HSP70
Secondary Structure
Globular Domains
Disorder Score
Coiled-Coil
Transmembrane
0
100
200
300
400
500
600

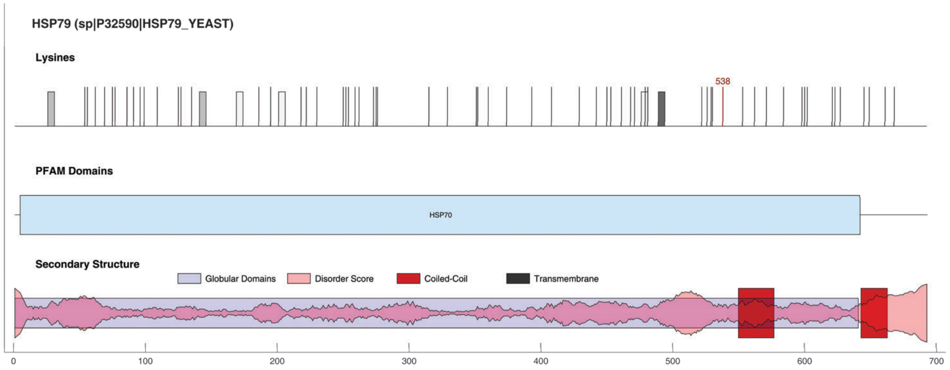
HSP79 (sp|P32590|HSP79_YEAST)
Lysines
538
PFAM Domains
HSP70
Secondary Structure
Globular Domains
Disorder Score
Coiled-Coil
Transmembrane
0
100
200
300
400
500
600
700

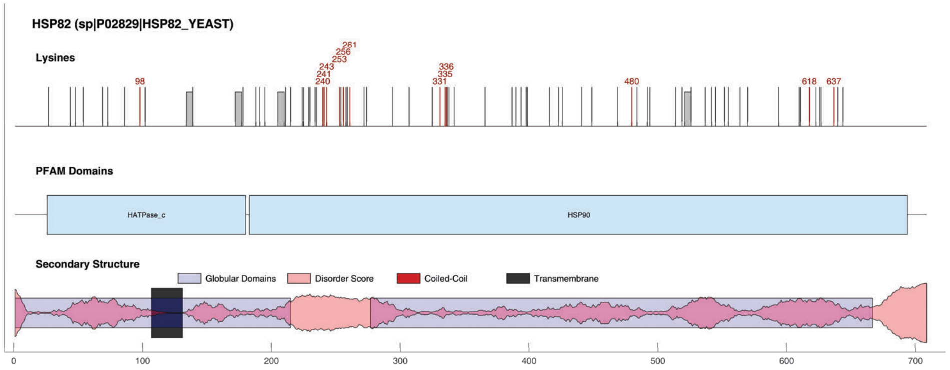
HSP82 (sp|P02829|HSP82_YEAST)
Lysines
98
261
256
253
243
241
240
336
335
331
480
618
637
PFAM Domains
HATPase_c
HSP90
Secondary Structure
Globular Domains
Disorder Score
Coiled-Coil
Transmembrane
0
100
200
300
400
500
600
700

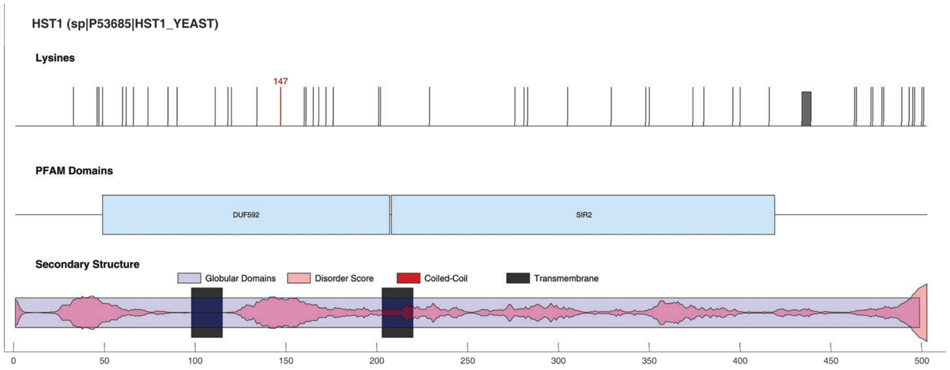
HST1 (sp|P53685|HST1_YEAST)
Lysines
147
PFAM Domains
DUF592
SIR2
Secondary Structure
Globular Domains
Disorder Score
Coiled-Coil
Transmembrane
0
50
100
150
200
250
300
350
400
450
500

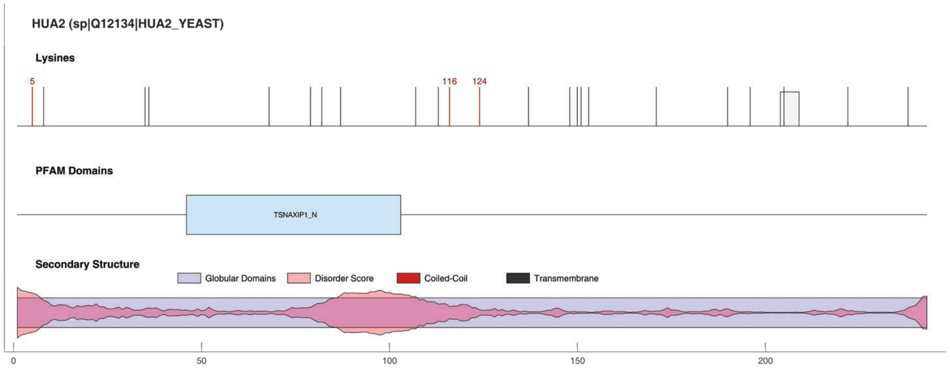
HUA2 (sp|Q12134|HUA2_YEAST)
Lysines
5
116
124
PFAM Domains
TSNAXIP1_N
Secondary Structure
Globular Domains
Disorder Score
Coiled-Coil
Transmembrane
0
50
100
150
200

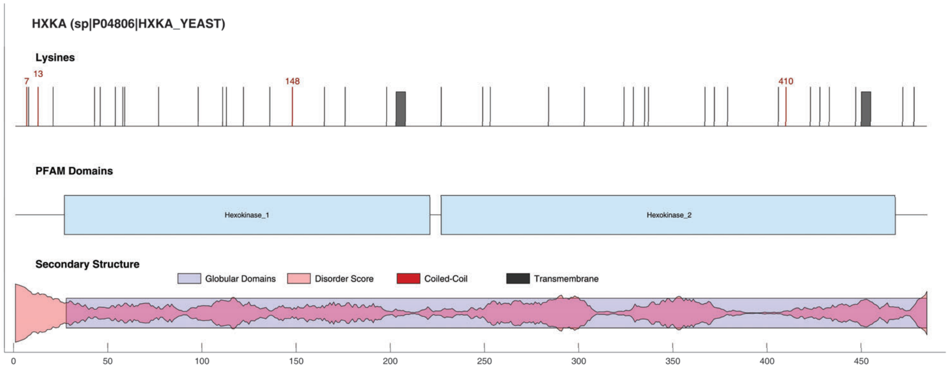
HXKA (sp|P04806|HXKA_YEAST)
Lysines
7
13
148
410
PFAM Domains
Hexokinase_1
Hexokinase_2
Secondary Structure
Globular Domains
Disorder Score
Coiled-Coil
Transmembrane
0
50
100
150
200
250
300
350
400
450

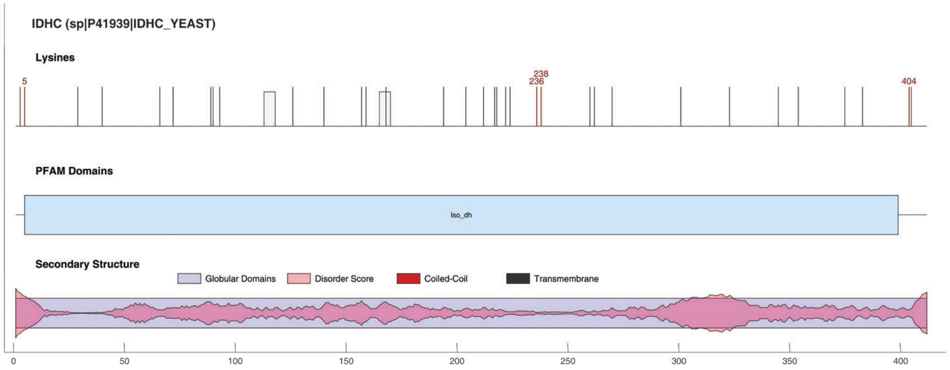
IDHC (sp|P41939|IDHC_YEAST)
Lysines
5
238
236
404
PFAM Domains
Iso_dh
Secondary Structure
Globular Domains
Disorder Score
Coiled-Coil
Transmembrane
0
50
100
150
200
250
300
350
400

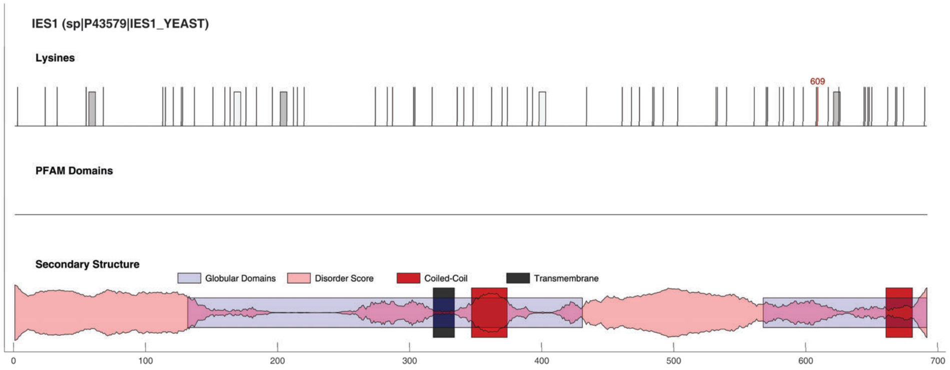

IES1 (sp|P43579|IES1_YEAST)
Lysines
609
PFAM Domains
Secondary Structure
Globular Domains
Disorder Score
Coiled-Coil
Transmembrane
0
100
200
300
400
500
600
700

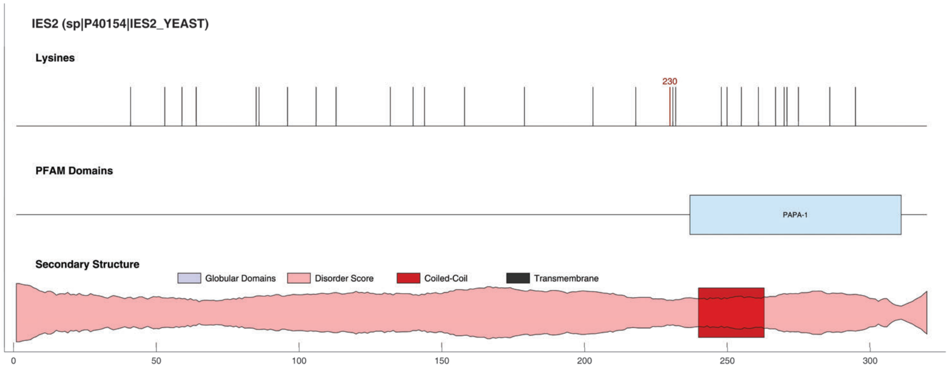

IES2 (sp|P40154|IES2_YEAST)
Lysines
230
PFAM Domains
PAPA-1
Secondary Structure
Globular Domains
Disorder Score
Coiled-Coil
Transmembrane
0
50
100
150
200
250
300

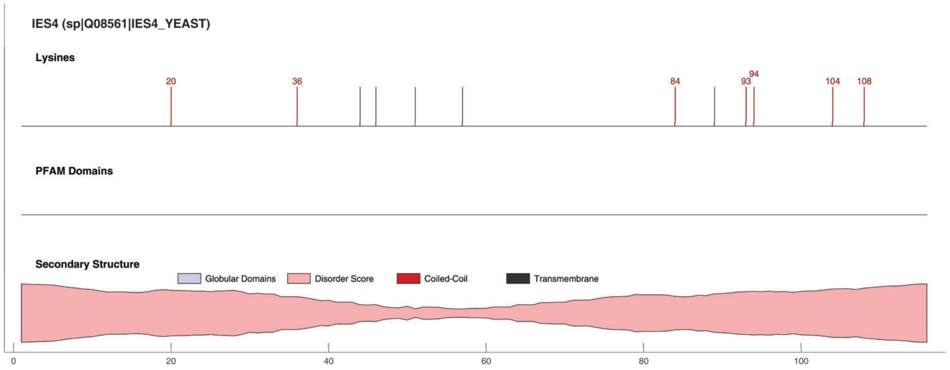

IES4 (sp|Q08561|IES4_YEAST)
Lysines
20
36
84
93
94
104
108
PFAM Domains
Secondary Structure
Globular Domains
Disorder Score
Coiled-Coil
Transmembrane
0
20
40
60
80
100

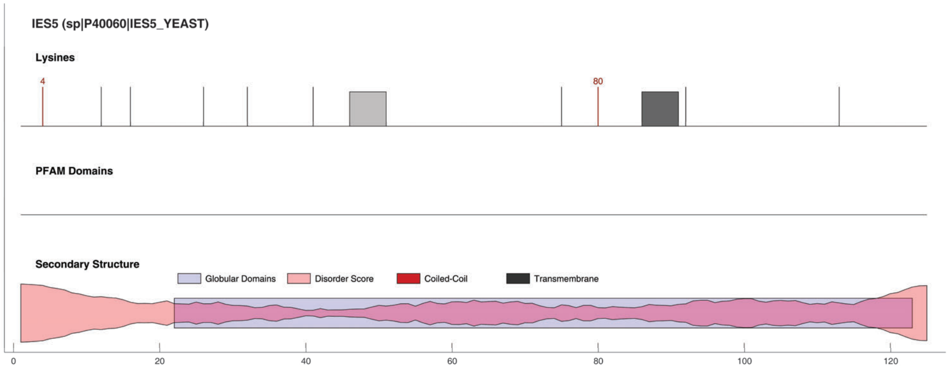

IES5 (sp|P40060|IES5_YEAST)
Lysines
4
80
PFAM Domains
Secondary Structure
Globular Domains
Disorder Score
Coiled-Coil
Transmembrane
0
20
40
60
80
100
120

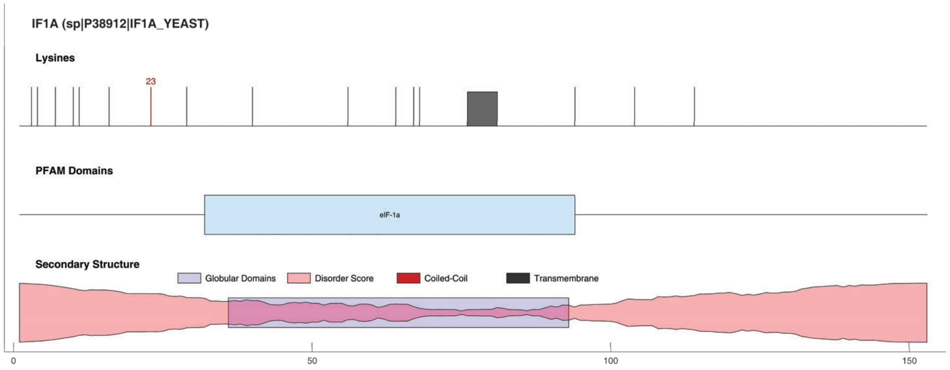
IF1A (sp|P38912|IF1A_YEAST)
Lysines
23
PFAM Domains
eIF-1a
Secondary Structure
Globular Domains
Disorder Score
Coiled-Coil
Transmembrane
0
50
100
150

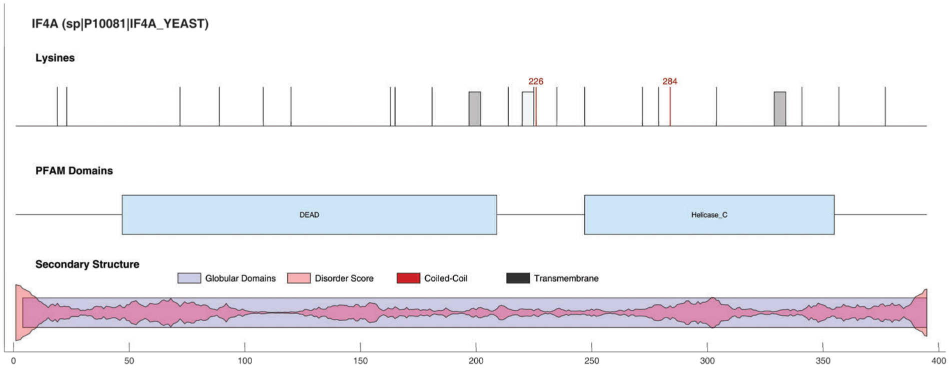
IF4A (sp|P10081|IF4A_YEAST)
Lysines
226
284
PFAM Domains
DEAD
Helicase_C
Secondary Structure
Globular Domains
Disorder Score
Coiled-Coil
Transmembrane
0
50
100
150
200
250
300
350
400

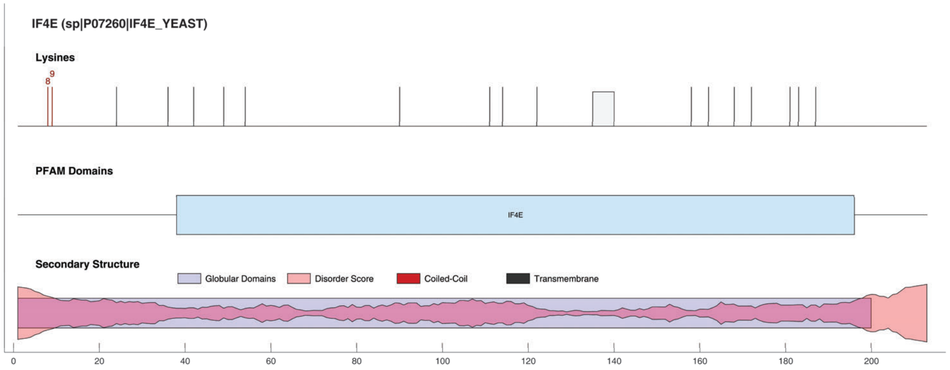
IF4E (sp|P07260|IF4E_YEAST)
Lysines
9
8
PFAM Domains
IF4E
Secondary Structure
Globular Domains
Disorder Score
Coiled-Coil
Transmembrane
0
20
40
60
80
100
120
140
160
180
200

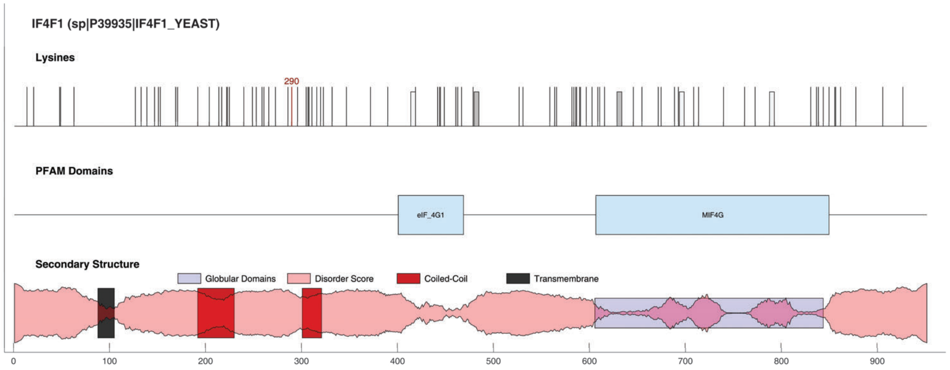
IF4F1 (sp|P39935|IF4F1_YEAST)
Lysines
290
PFAM Domains
eIF_4G1
MIF4G
Secondary Structure
Globular Domains
Disorder Score
Coiled-Coil
Transmembrane
0
100
200
300
400
500
600
700
800
900

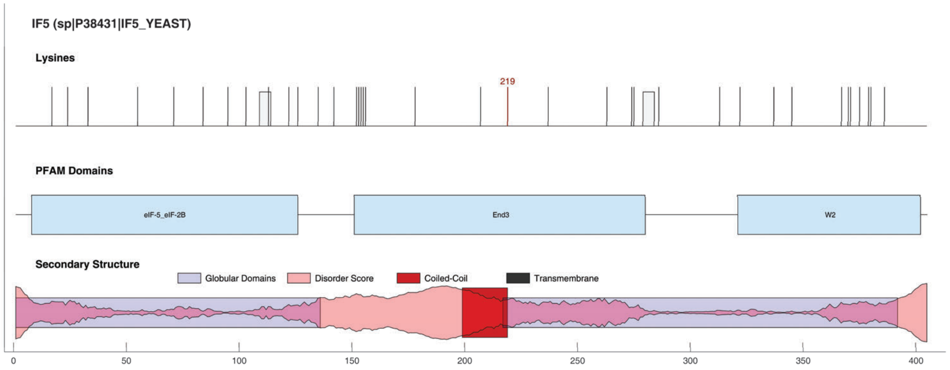

IF5 (sp|P38431|IF5_YEAST)
Lysines
219
PFAM Domains
eIF-5_eIF-2B
End3
W2
Secondary Structure
Globular Domains
Disorder Score
Coiled-Coil
Transmembrane
0
50
100
150
200
250
300
350
400

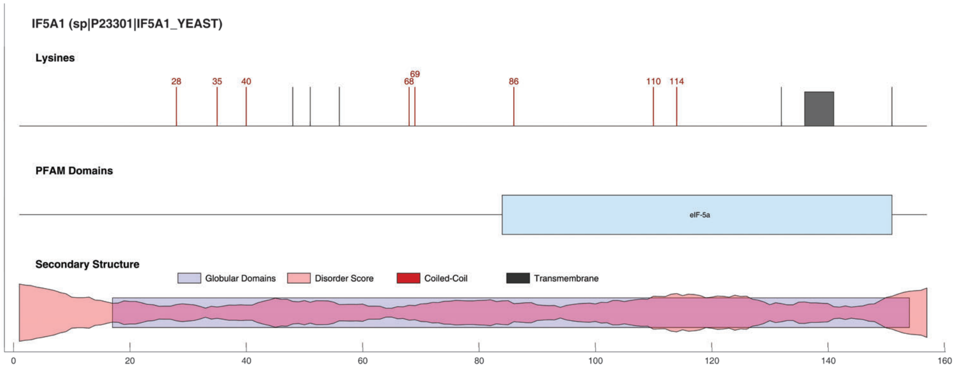

IF5A1 (sp|P23301|IF5A1_YEAST)
Lysines
28
35
40
68
69
86
110
114
PFAM Domains
eIF-5a
Secondary Structure
Globular Domains
Disorder Score
Coiled-Coil
Transmembrane
0
20
40
60
80
100
120
140
160

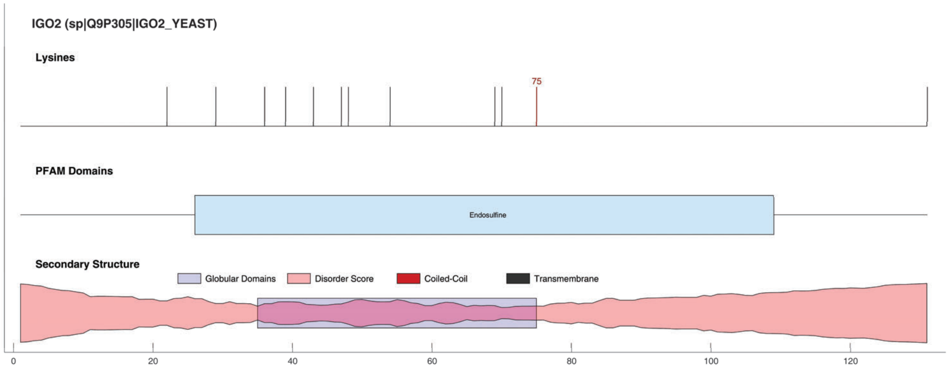

IGO2 (sp|Q9P305|IGO2_YEAST)
Lysines
75
PFAM Domains
Endosulfine
Secondary Structure
Globular Domains
Disorder Score
Coiled-Coil
Transmembrane
0
20
40
60
80
100
120

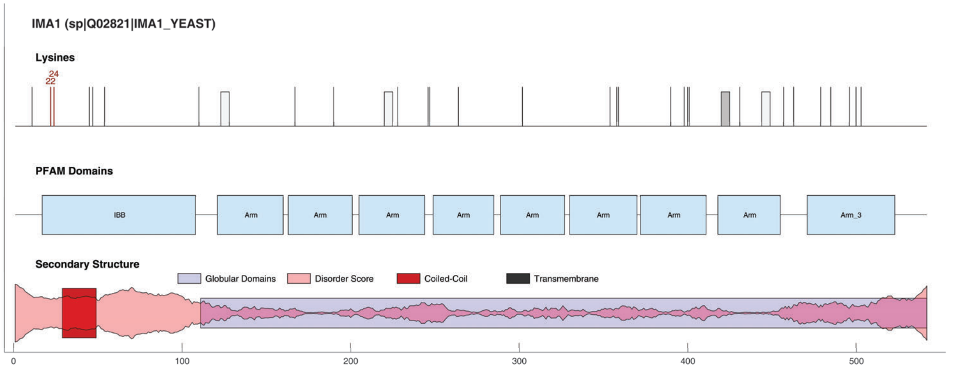

IMA1 (sp|Q02821|IMA1_YEAST)
Lysines
22
24
PFAM Domains
IBB
Arm
Arm
Arm
Arm
Arm
Arm
Arm
Arm
Arm_3
Secondary Structure
Globular Domains
Disorder Score
Coiled-Coil
Transmembrane
0
100
200
300
400
500

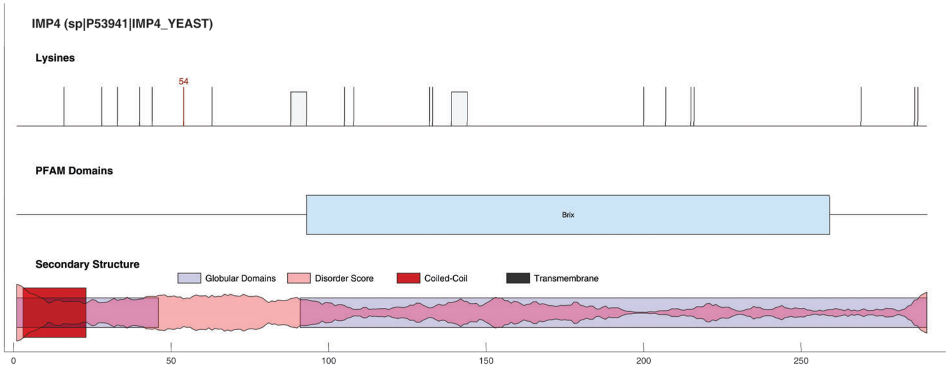
IMP4 (sp|P53941|IMP4_YEAST)
Lysines
54
PFAM Domains
Brix
Secondary Structure
Globular Domains
Disorder Score
Coiled-Coil
Transmembrane
0
50
100
150
200
250

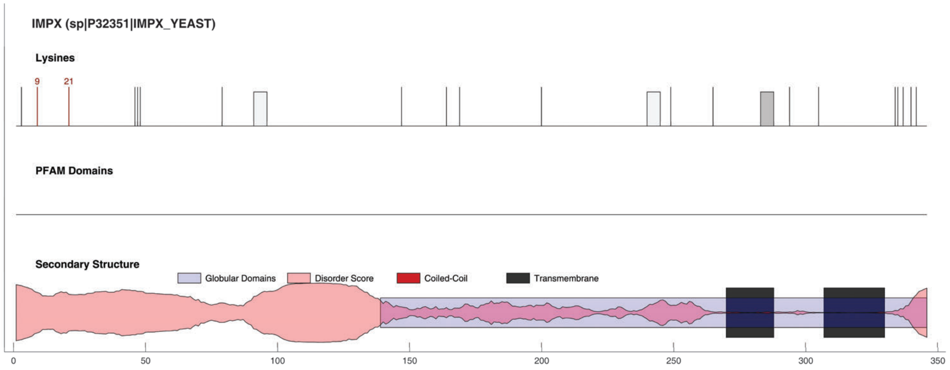
IMPX (sp|P32351|IMPX_YEAST)
Lysines
9
21
PFAM Domains
Secondary Structure
Globular Domains
Disorder Score
Coiled-Coil
Transmembrane
0
50
100
150
200
250
300
350

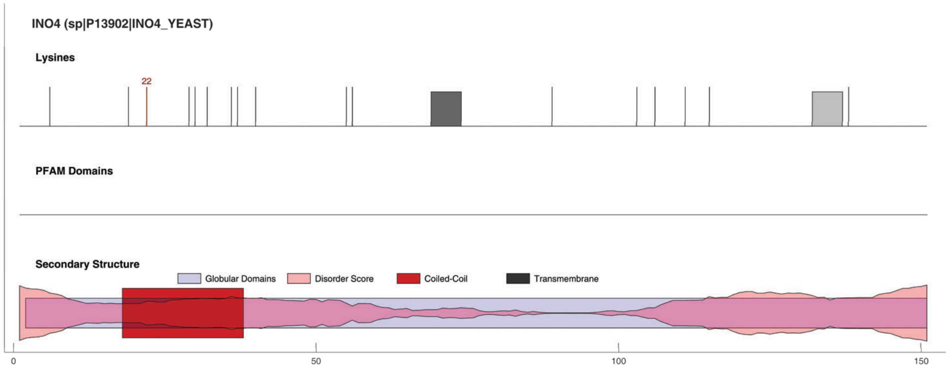
INO4 (sp|P13902|INO4_YEAST)
Lysines
22
PFAM Domains
Secondary Structure
Globular Domains
Disorder Score
Coiled-Coil
Transmembrane
0
50
100
150

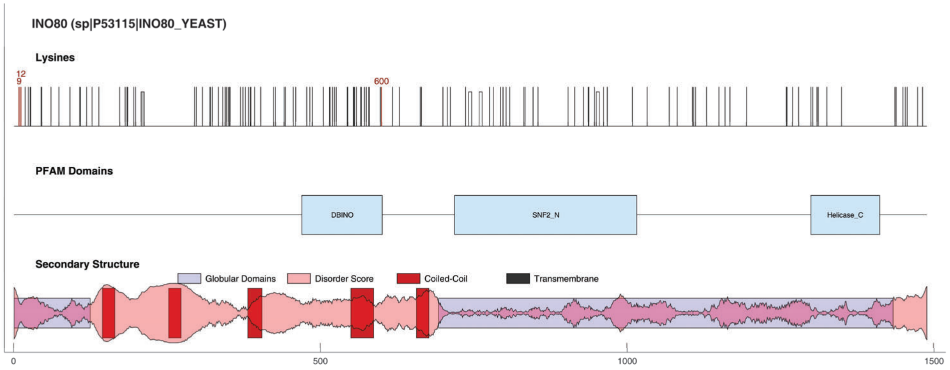
INO80 (sp|P53115|INO80_YEAST)
Lysines
12
9
600
PFAM Domains
DBINO
SNF2_N
Helicase_C
Secondary Structure
Globular Domains
Disorder Score
Coiled-Coil
Transmembrane
0
500
1000
1500

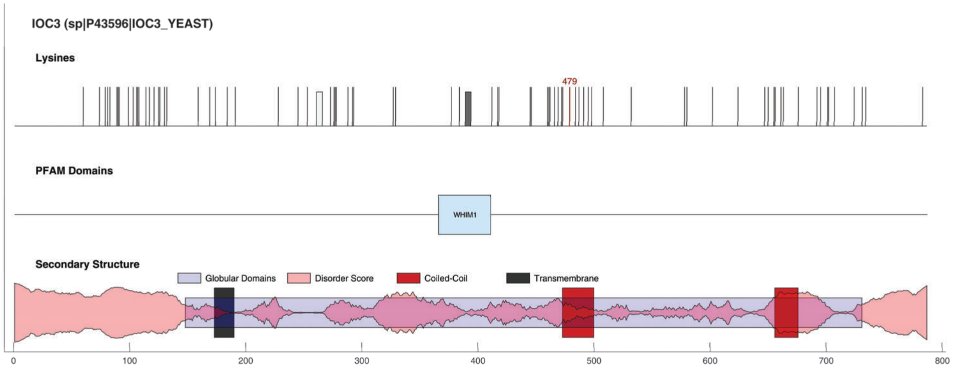
IOC3 (sp|P43596|IOC3_YEAST)
Lysines
479
PFAM Domains
WHIM1
Secondary Structure
Globular Domains
Disorder Score
Coiled-Coil
Transmembrane
0
100
200
300
400
500
600
700
800

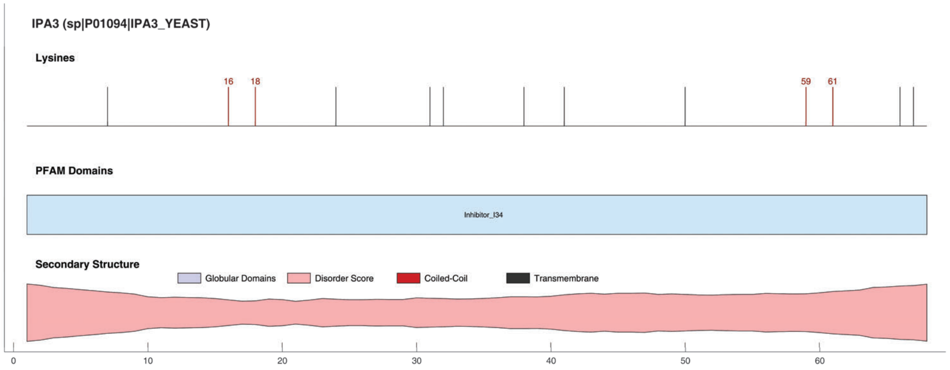
IPA3 (sp|P01094|IPA3_YEAST)
Lysines
16
18
59
61
PFAM Domains
Inhibitor_I34
Secondary Structure
Globular Domains
Disorder Score
Coiled-Coil
Transmembrane
0
10
20
30
40
50
60

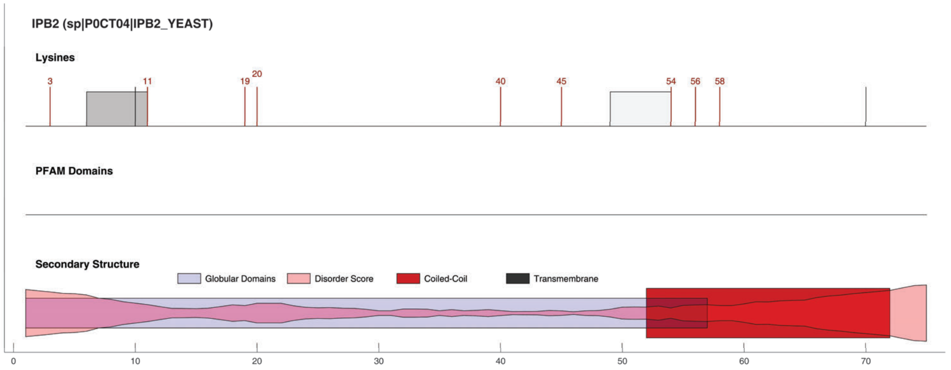
IPB2 (sp|P0CT04|IPB2_YEAST)
Lysines
3
11
19
20
40
45
54
56
58
PFAM Domains
Secondary Structure
Globular Domains
Disorder Score
Coiled-Coil
Transmembrane
0
10
20
30
40
50
60
70

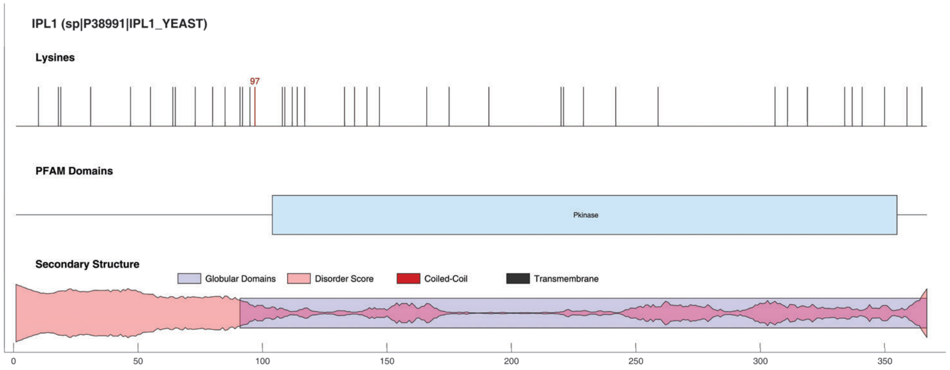
IPL1 (sp|P38991|IPL1_YEAST)
Lysines
97
PFAM Domains
Pkinase
Secondary Structure
Globular Domains
Disorder Score
Coiled-Coil
Transmembrane
0
50
100
150
200
250
300
350

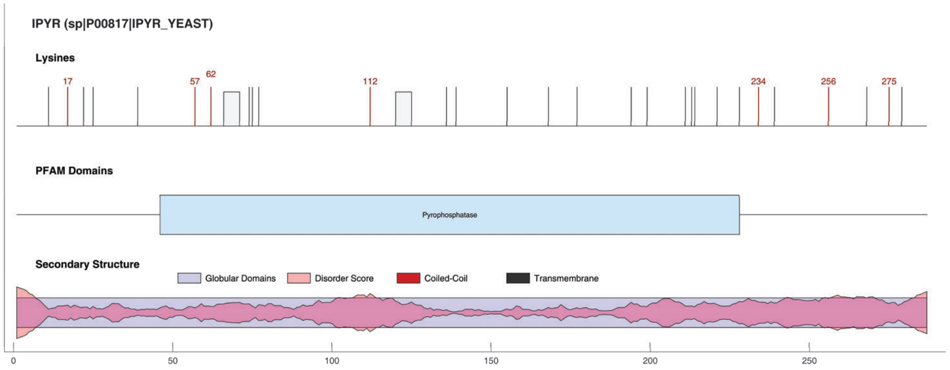
IPYR (sp|P00817|IPYR_YEAST)
Lysines
17
57
62
112
234
256
275
PFAM Domains
Pyrophosphatase
Secondary Structure
Globular Domains
Disorder Score
Coiled-Coil
Transmembrane
0
50
100
150
200
250

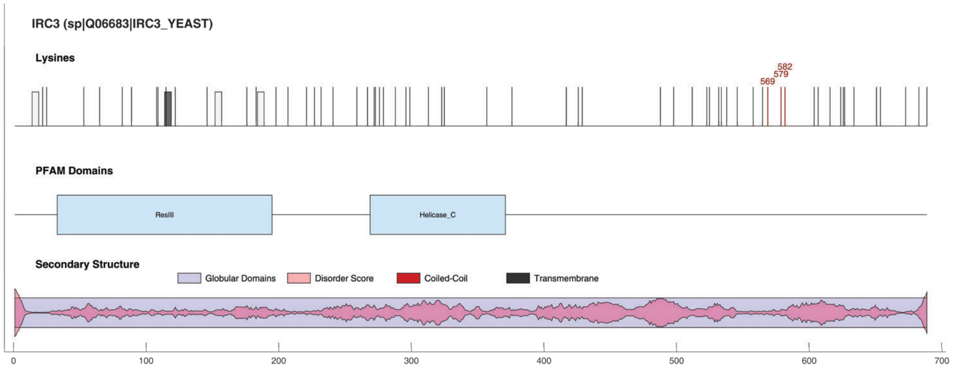
IRC3 (sp|Q06683|IRC3_YEAST)
Lysines
569
579
582
PFAM Domains
ResIII
Helicase_C
Secondary Structure
Globular Domains
Disorder Score
Coiled-Coil
Transmembrane
0
100
200
300
400
500
600
700

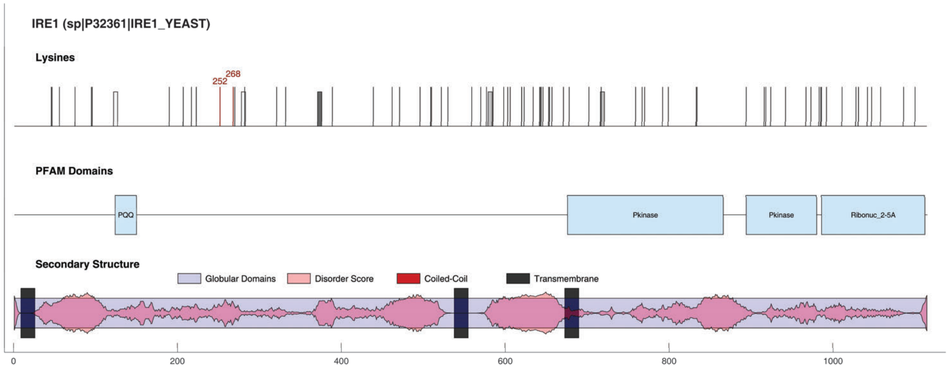
IRE1 (sp|P32361|IRE1_YEAST)
Lysines
252
268
PFAM Domains
PQQ
Pkinase
Pkinase
Ribonuc_2-5A
Secondary Structure
Globular Domains
Disorder Score
Coiled-Coil
Transmembrane
0
200
400
600
800
1000

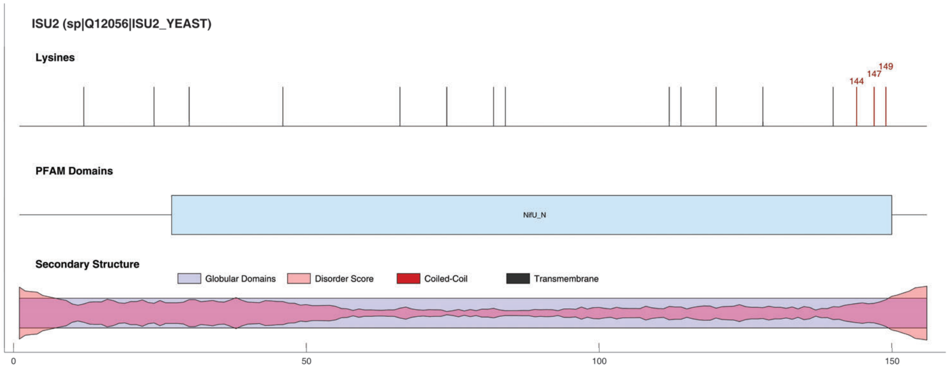
ISU2 (sp|Q12056|ISU2_YEAST)
Lysines
144
147
149
PFAM Domains
NifU_N
Secondary Structure
Globular Domains
Disorder Score
Coiled-Coil
Transmembrane
0
50
100
150

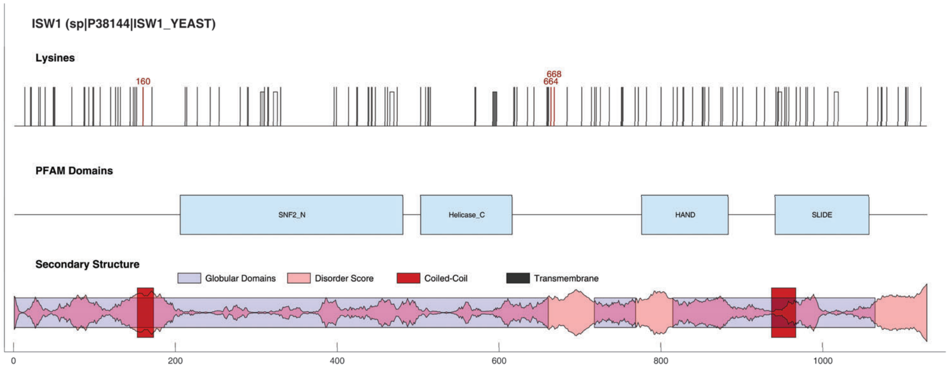

ISW1 (sp|P38144|ISW1_YEAST)
Lysines
160
668
664
PFAM Domains
SNF2_N
Helicase_C
HAND
SLIDE
Secondary Structure
Globular Domains
Disorder Score
Coiled-Coil
Transmembrane
0
200
400
600
800
1000

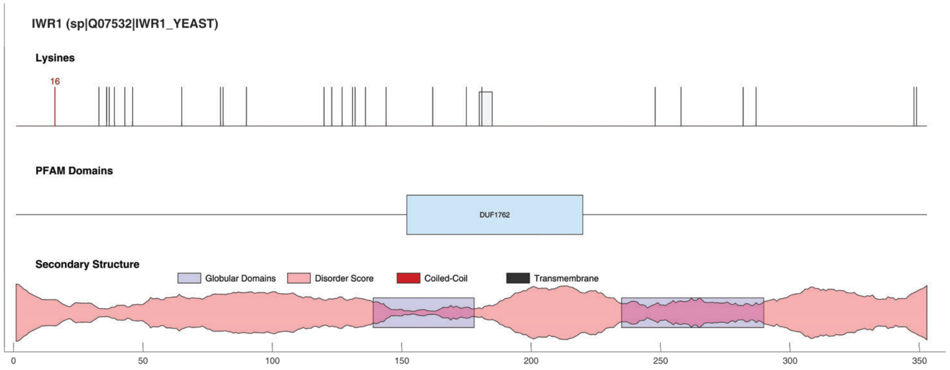

IWR1 (sp|Q07532|IWR1_YEAST)
Lysines
16
PFAM Domains
DUF1762
Secondary Structure
Globular Domains
Disorder Score
Coiled-Coil
Transmembrane
0
50
100
150
200
250
300
350

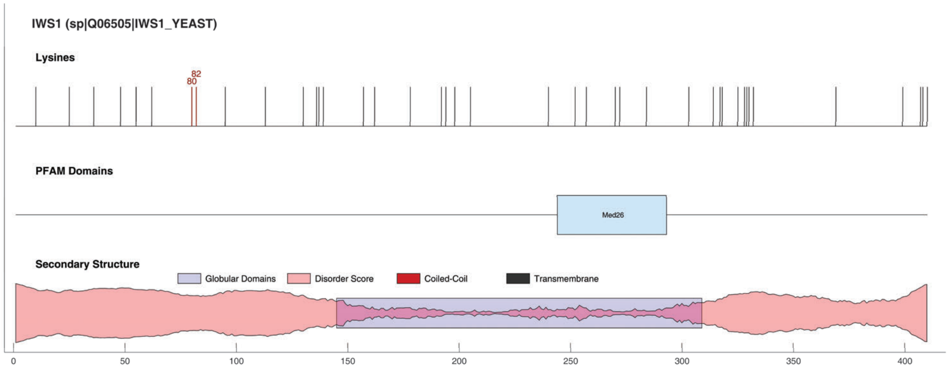

IWS1 (sp|Q06505|IWS1_YEAST)
Lysines
82
80
PFAM Domains
Med26
Secondary Structure
Globular Domains
Disorder Score
Coiled-Coil
Transmembrane
0
50
100
150
200
250
300
350
400

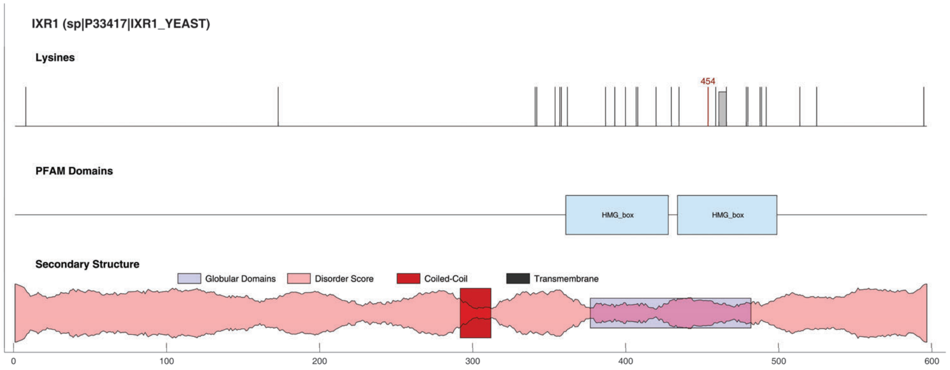

IXR1 (sp|P33417|IXR1_YEAST)
Lysines
454
PFAM Domains
HMG_box
HMG_box
Secondary Structure
Globular Domains
Disorder Score
Coiled-Coil
Transmembrane
0
100
200
300
400
500
600

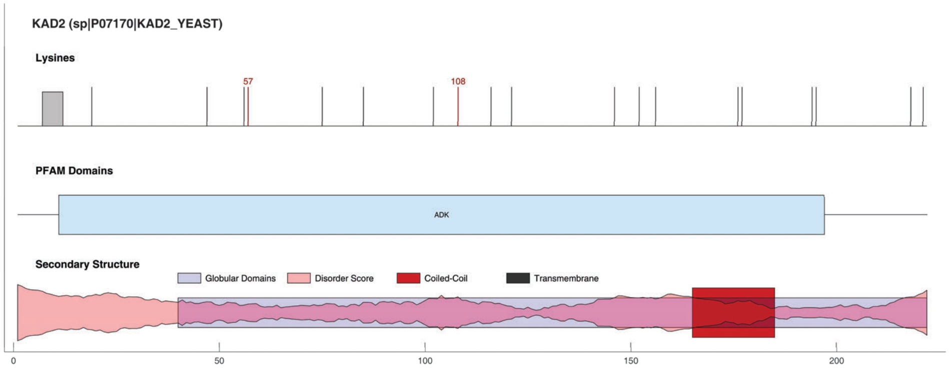
KAD2 (sp|P07170|KAD2_YEAST)
Lysines
57
108
PFAM Domains
ADK
Secondary Structure
Globular Domains
Disorder Score
Coiled-Coil
Transmembrane
0
50
100
150
200

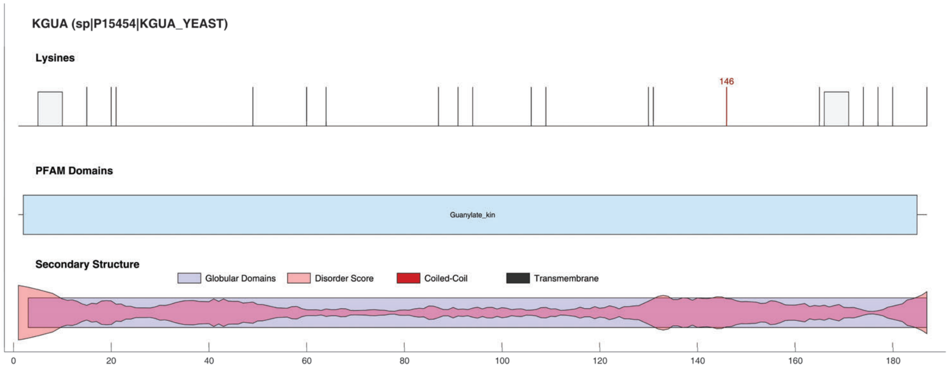
KGUA (sp|P15454|KGUA_YEAST)
Lysines
146
PFAM Domains
Guanylate_kin
Secondary Structure
Globular Domains
Disorder Score
Coiled-Coil
Transmembrane
0
20
40
60
80
100
120
140
160
180

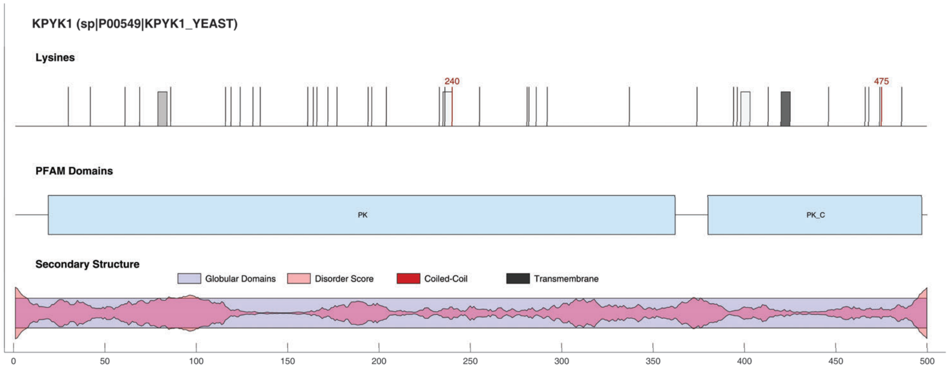
KPYK1 (sp|P00549|KPYK1_YEAST)
Lysines
240
475
PFAM Domains
PK
PK_C
Secondary Structure
Globular Domains
Disorder Score
Coiled-Coil
Transmembrane
0
50
100
150
200
250
300
350
400
450
500

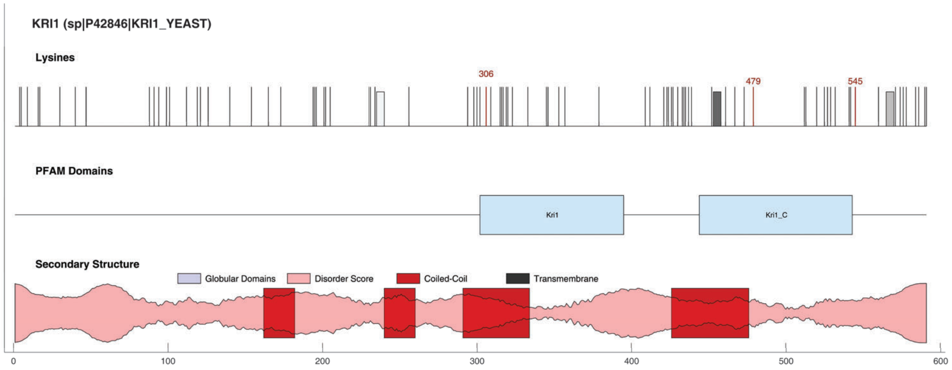
KRI1 (sp|P42846|KRI1_YEAST)
Lysines
306
479
545
PFAM Domains
Kri1
Kri1_C
Secondary Structure
Globular Domains
Disorder Score
Coiled-Coil
Transmembrane
0
100
200
300
400
500
600

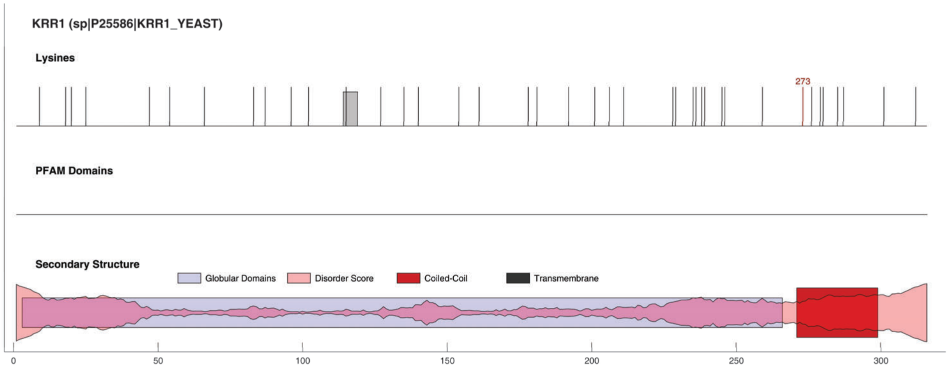
KRR1 (sp|P25586|KRR1_YEAST)
Lysines
273
PFAM Domains
Secondary Structure
Globular Domains
Disorder Score
Coiled-Coil
Transmembrane
0
50
100
150
200
250
300

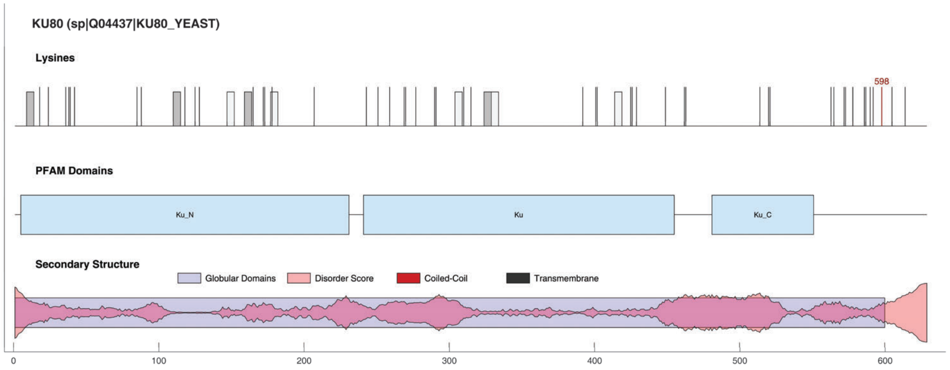
KU80 (sp|Q04437|KU80_YEAST)
Lysines
598
PFAM Domains
Ku_N
Ku
Ku_C
Secondary Structure
Globular Domains
Disorder Score
Coiled-Coil
Transmembrane
0
100
200
300
400
500
600

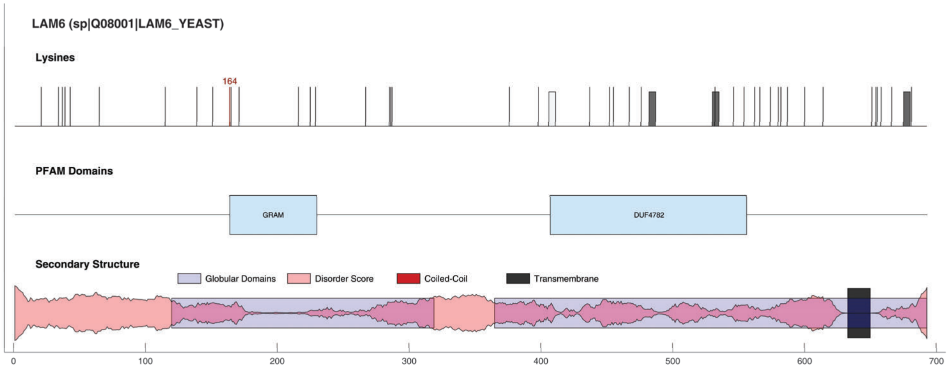
LAM6 (sp|Q08001|LAM6_YEAST)
Lysines
164
PFAM Domains
GRAM
DUF4782
Secondary Structure
Globular Domains
Disorder Score
Coiled-Coil
Transmembrane
0
100
200
300
400
500
600
700

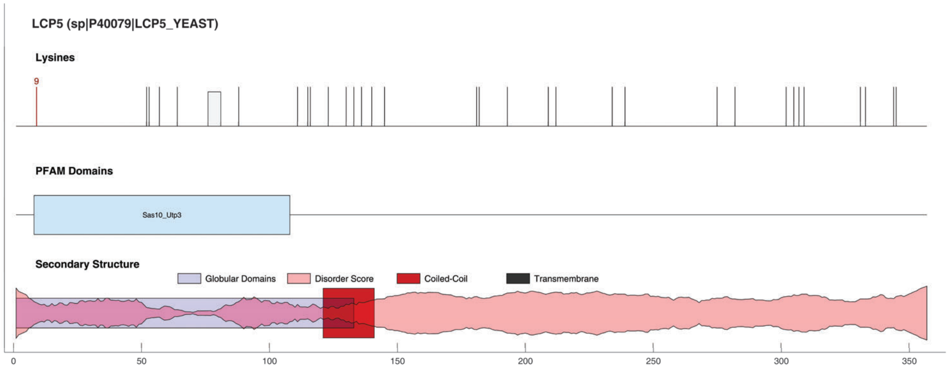
LCP5 (sp|P40079|LCP5_YEAST)
Lysines
9
PFAM Domains
Sas10_Utp3
Secondary Structure
Globular Domains
Disorder Score
Coiled-Coil
Transmembrane
0
50
100
150
200
250
300
350

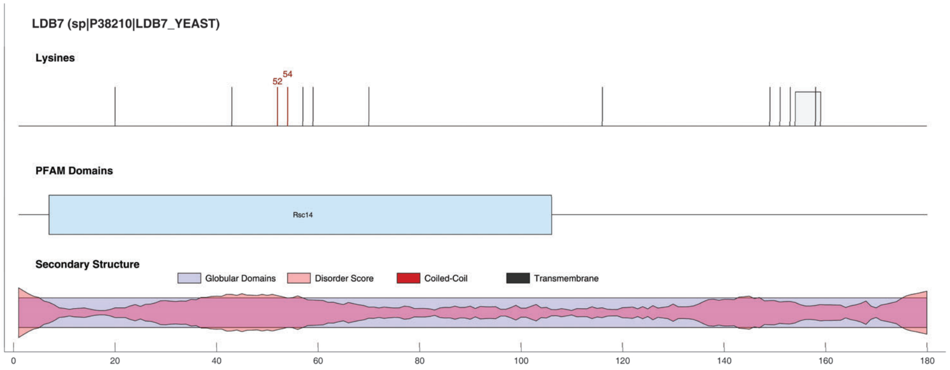

LDB7 (sp|P38210|LDB7_YEAST)
Lysines
52
54
PFAM Domains
Rsc14
Secondary Structure
Globular Domains
Disorder Score
Coiled-Coil
Transmembrane
0
20
40
60
80
100
120
140
160
180

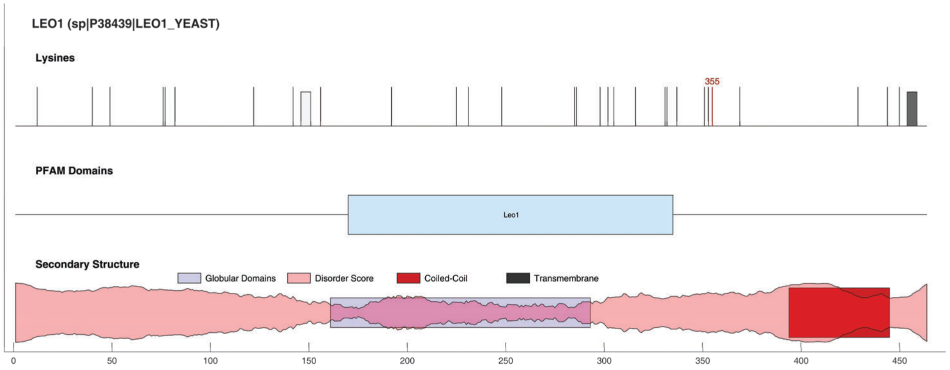

LEO1 (sp|P38439|LEO1_YEAST)
Lysines
355
PFAM Domains
Leo1
Secondary Structure
Globular Domains
Disorder Score
Coiled-Coil
Transmembrane
0
50
100
150
200
250
300
350
400
450

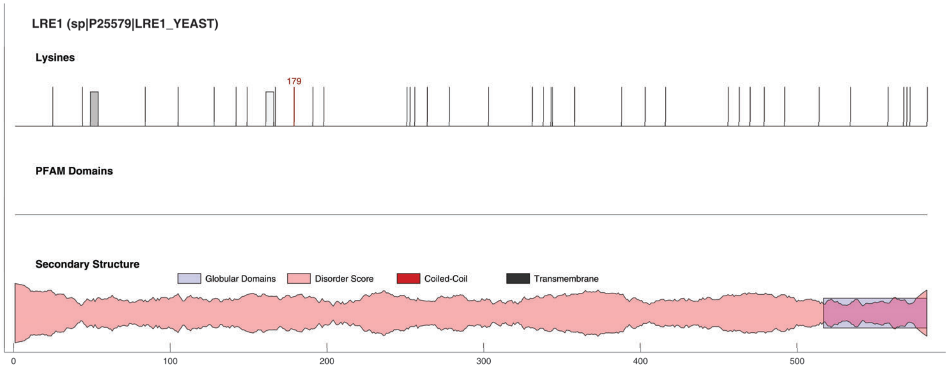

LRE1 (sp|P25579|LRE1_YEAST)
Lysines
179
PFAM Domains
Secondary Structure
Globular Domains
Disorder Score
Coiled-Coil
Transmembrane
0
100
200
300
400
500

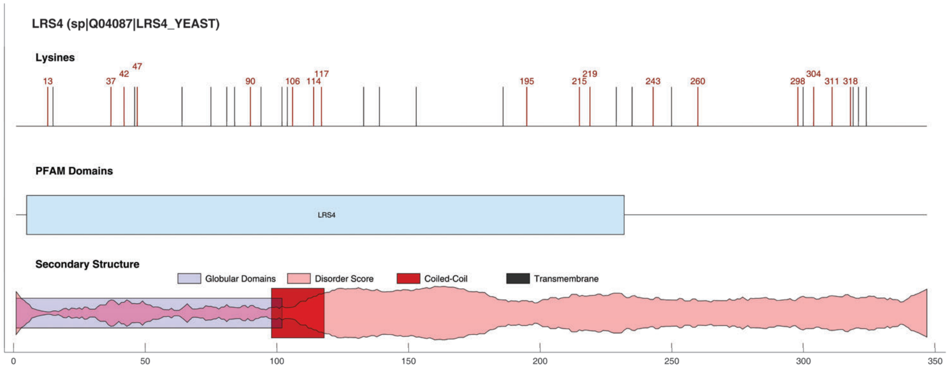

LRS4 (sp|Q04087|LRS4_YEAST)
Lysines
13
37
42
47
90
106
114
117
195
215
219
243
260
298
304
311
318
PFAM Domains
LRS4
Secondary Structure
Globular Domains
Disorder Score
Coiled-Coil
Transmembrane
0
50
100
150
200
250
300
350

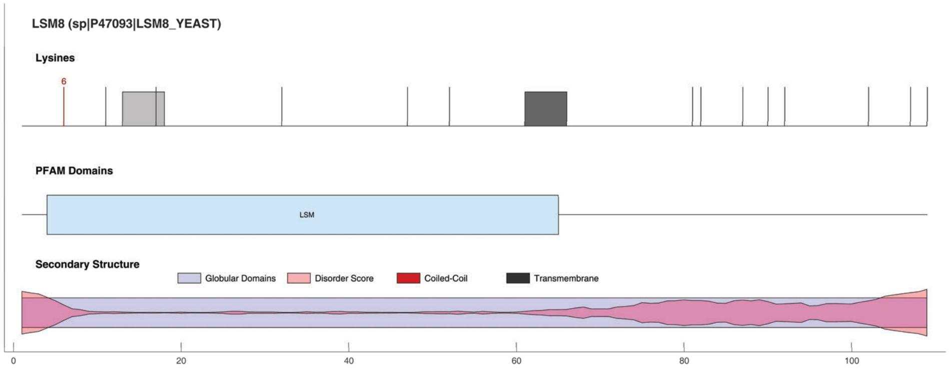
LSM8 (sp|P47093|LSM8_YEAST)
Lysines
6
PFAM Domains
LSM
Secondary Structure
Globular Domains
Disorder Score
Coiled-Coil
Transmembrane
0
20
40
60
80
100

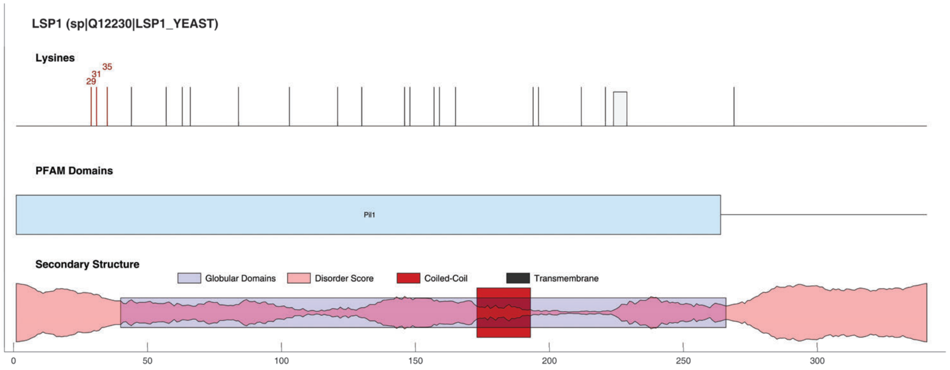
LSP1 (sp|Q12230|LSP1_YEAST)
Lysines
29
31
35
PFAM Domains
Pil1
Secondary Structure
Globular Domains
Disorder Score
Coiled-Coil
Transmembrane
0
50
100
150
200
250
300

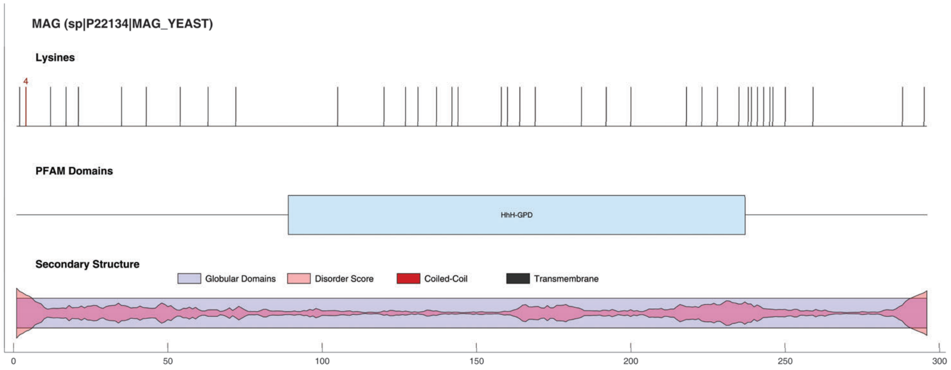
MAG (sp|P22134|MAG_YEAST)
Lysines
4
PFAM Domains
HhH-GPD
Secondary Structure
Globular Domains
Disorder Score
Coiled-Coil
Transmembrane
0
50
100
150
200
250
300

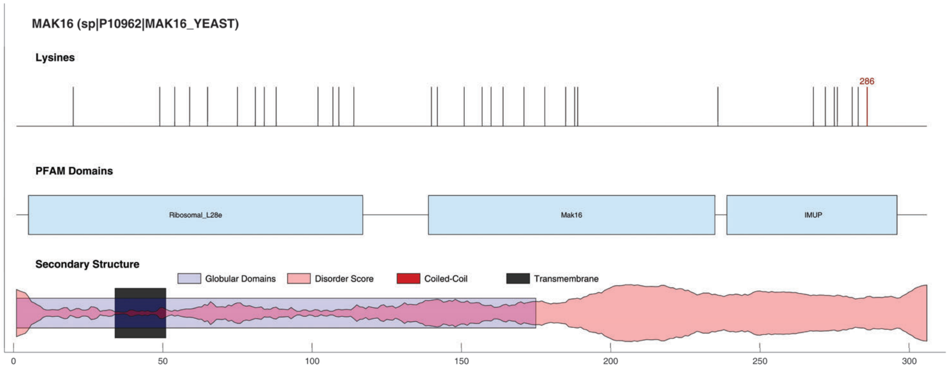
MAK16 (sp|P10962|MAK16_YEAST)
Lysines
286
PFAM Domains
Ribosomal_L28e
Mak16
IMUP
Secondary Structure
Globular Domains
Disorder Score
Coiled-Coil
Transmembrane
0
50
100
150
200
250
300

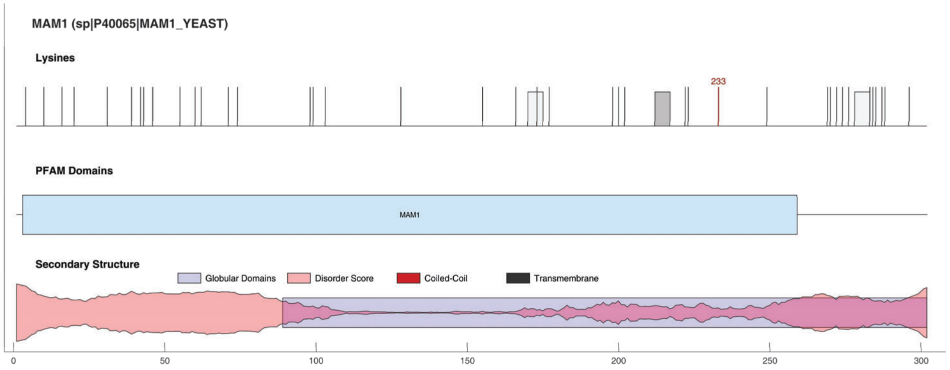
MAM1 (sp|P40065|MAM1_YEAST)
Lysines
233
PFAM Domains
MAM1
Secondary Structure
Globular Domains
Disorder Score
Coiled-Coil
Transmembrane
0
50
100
150
200
250
300

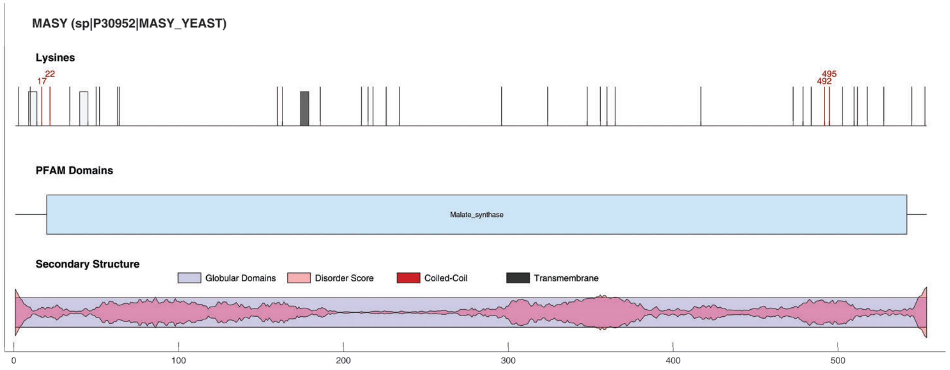
MASY (sp|P30952|MASY_YEAST)
Lysines
17
22
492
495
PFAM Domains
Malate_synthase
Secondary Structure
Globular Domains
Disorder Score
Coiled-Coil
Transmembrane
0
100
200
300
400
500

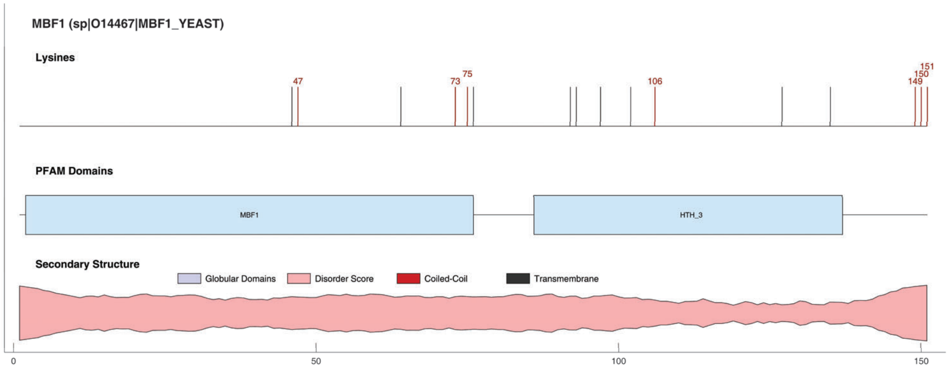
MBF1 (sp|O14467|MBF1_YEAST)
Lysines
47
73
75
106
149
150
151
PFAM Domains
MBF1
HTH_3
Secondary Structure
Globular Domains
Disorder Score
Coiled-Coil
Transmembrane
0
50
100
150

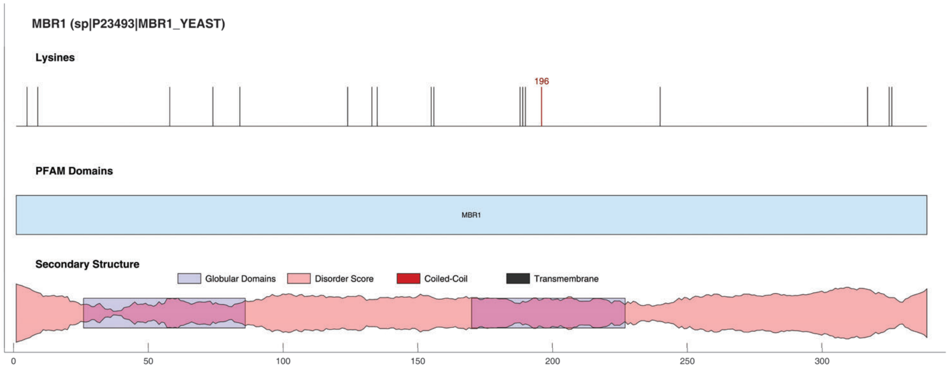
MBR1 (sp|P23493|MBR1_YEAST)
Lysines
196
PFAM Domains
MBR1
Secondary Structure
Globular Domains
Disorder Score
Coiled-Coil
Transmembrane
0
50
100
150
200
250
300

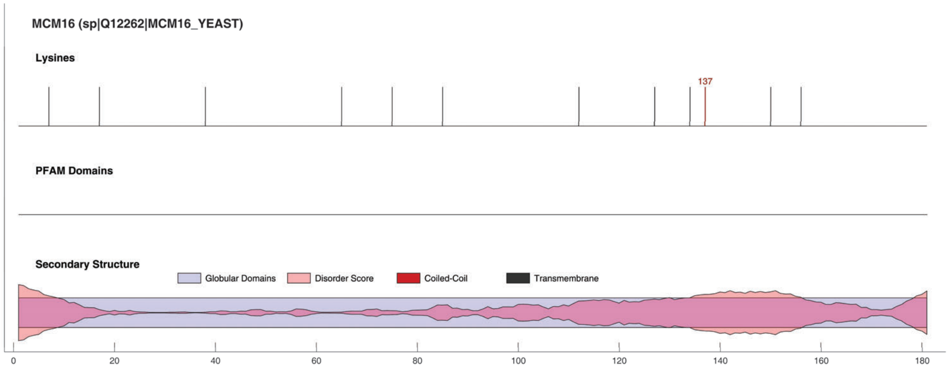
MCM16 (sp|Q12262|MCM16_YEAST)
Lysines
137
PFAM Domains
Secondary Structure
Globular Domains
Disorder Score
Coiled-Coil
Transmembrane
0
20
40
60
80
100
120
140
160
180

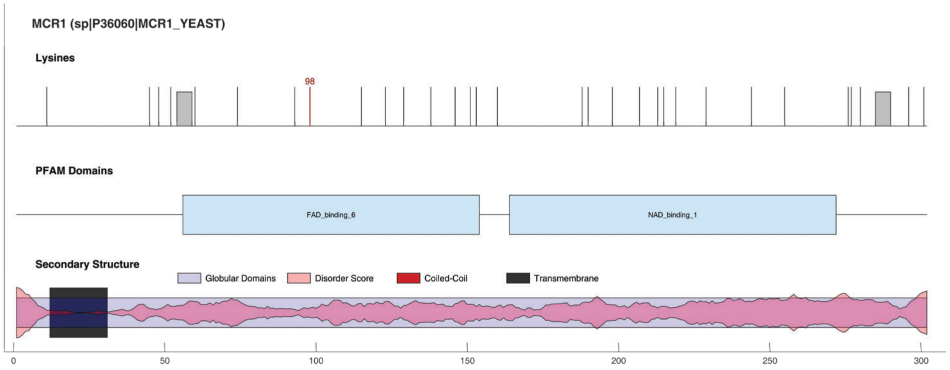
MCR1 (sp|P36060|MCR1_YEAST)
Lysines
98
PFAM Domains
FAD_binding_6
NAD_binding_1
Secondary Structure
Globular Domains
Disorder Score
Coiled-Coil
Transmembrane
0
50
100
150
200
250
300

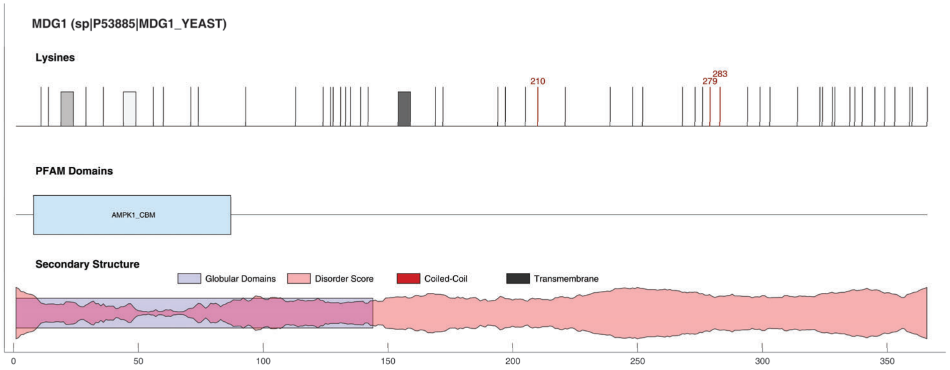
MDG1 (sp|P53885|MDG1_YEAST)
Lysines
210
279
283
PFAM Domains
AMPK1_CBM
Secondary Structure
Globular Domains
Disorder Score
Coiled-Coil
Transmembrane
0
50
100
150
200
250
300
350

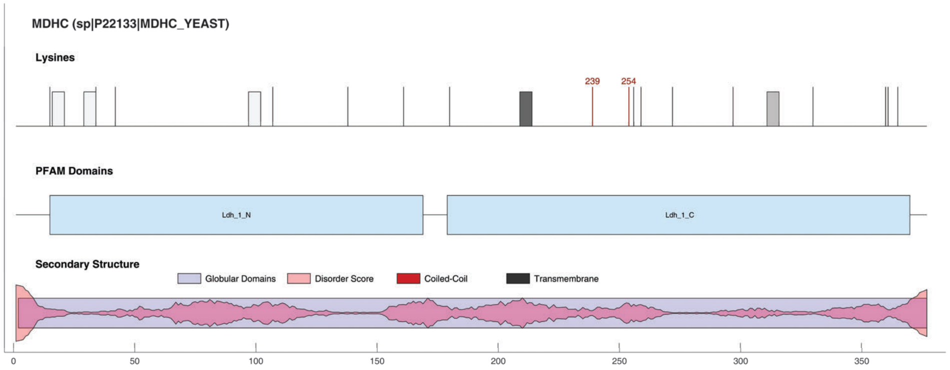
MDHC (sp|P22133|MDHC_YEAST)
Lysines
239
254
PFAM Domains
Ldh_1_N
Ldh_1_C
Secondary Structure
Globular Domains
Disorder Score
Coiled-Coil
Transmembrane
0
50
100
150
200
250
300
350

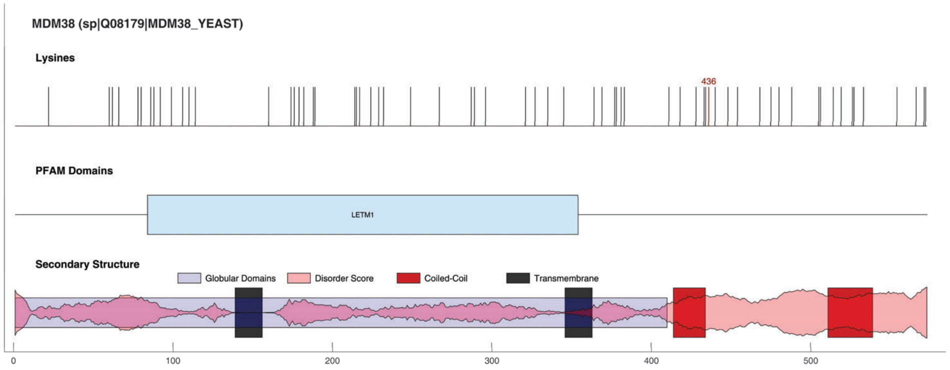
MDM38 (sp|Q08179|MDM38_YEAST)
Lysines
436
PFAM Domains
LETM1
Secondary Structure
Globular Domains
Disorder Score
Coiled-Coil
Transmembrane
0
100
200
300
400
500

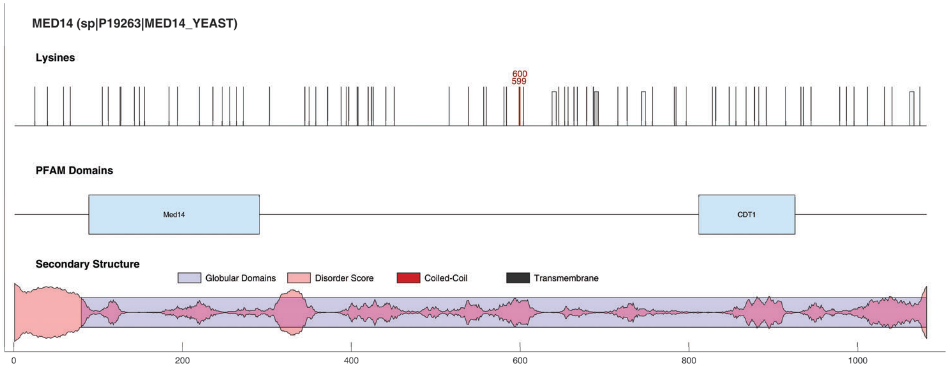
MED14 (sp|P19263|MED14_YEAST)
Lysines
600
599
PFAM Domains
Med14
CDT1
Secondary Structure
Globular Domains
Disorder Score
Coiled-Coil
Transmembrane
0
200
400
600
800
1000

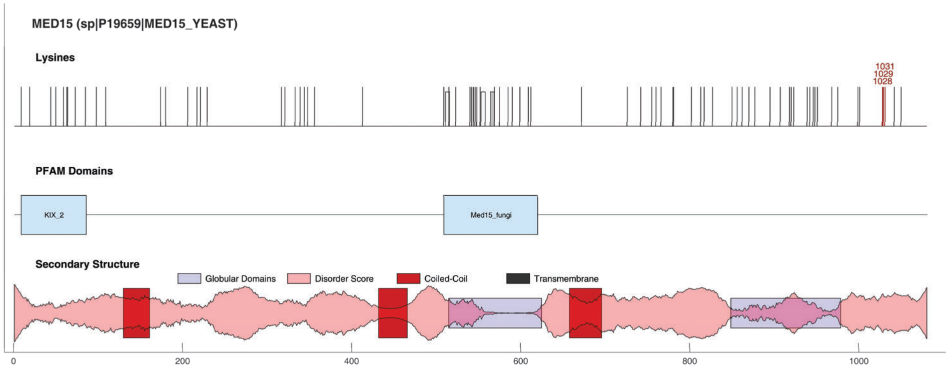
MED15 (sp|P19659|MED15_YEAST)
Lysines
1031
1029
1028
PFAM Domains
KIX_2
Med15_fungi
Secondary Structure
Globular Domains
Disorder Score
Coiled-Coil
Transmembrane
0
200
400
600
800
1000

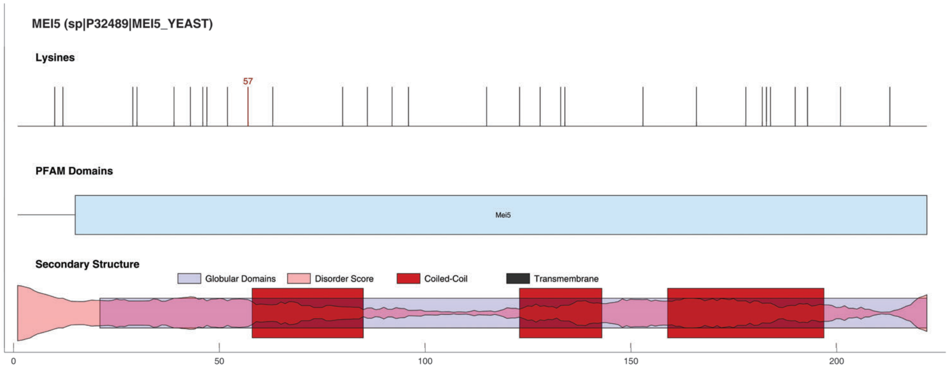
MEI5 (sp|P32489|MEI5_YEAST)
Lysines
57
PFAM Domains
Mei5
Secondary Structure
Globular Domains
Disorder Score
Coiled-Coil
Transmembrane
0
50
100
150
200

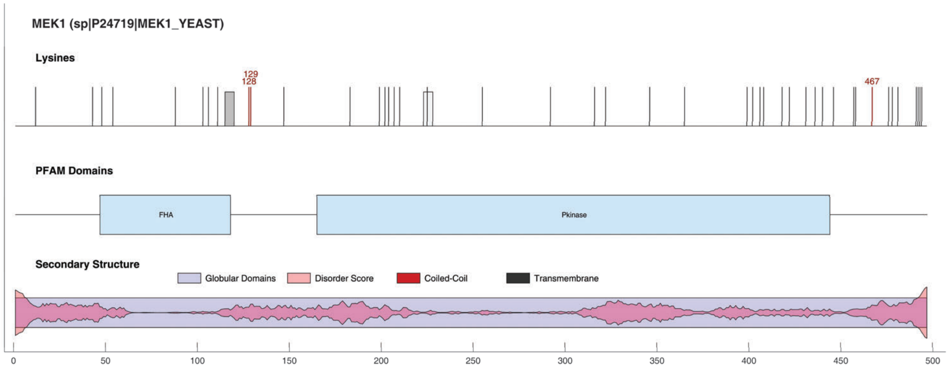

MEK1 (sp|P24719|MEK1_YEAST)
Lysines
129
128
467
PFAM Domains
FHA
Pkinase
Secondary Structure
Globular Domains
Disorder Score
Coiled-Coil
Transmembrane
0
50
100
150
200
250
300
350
400
450
500

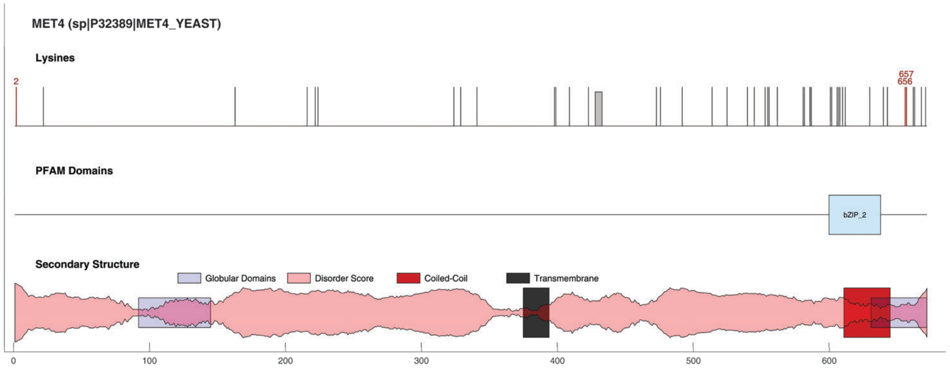

MET4 (sp|P32389|MET4_YEAST)
Lysines
2
657
656
PFAM Domains
bZIP_2
Secondary Structure
Globular Domains
Disorder Score
Coiled-Coil
Transmembrane
0
100
200
300
400
500
600

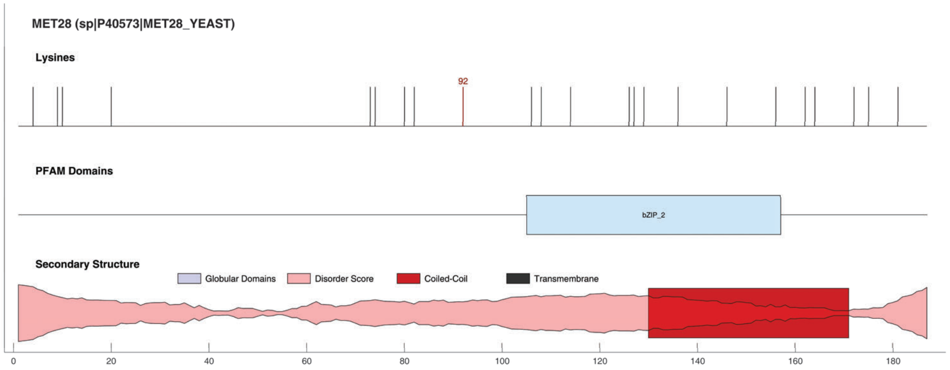

MET28 (sp|P40573|MET28_YEAST)
Lysines
92
PFAM Domains
bZIP_2
Secondary Structure
Globular Domains
Disorder Score
Coiled-Coil
Transmembrane
0
20
40
60
80
100
120
140
160
180

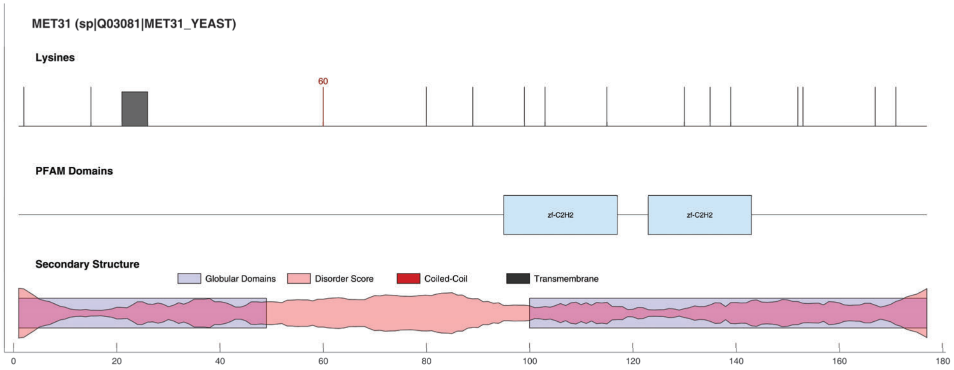

MET31 (sp|Q03081|MET31_YEAST)
Lysines
60
PFAM Domains
zf-C2H2
zf-C2H2
Secondary Structure
Globular Domains
Disorder Score
Coiled-Coil
Transmembrane
0
20
40
60
80
100
120
140
160
180

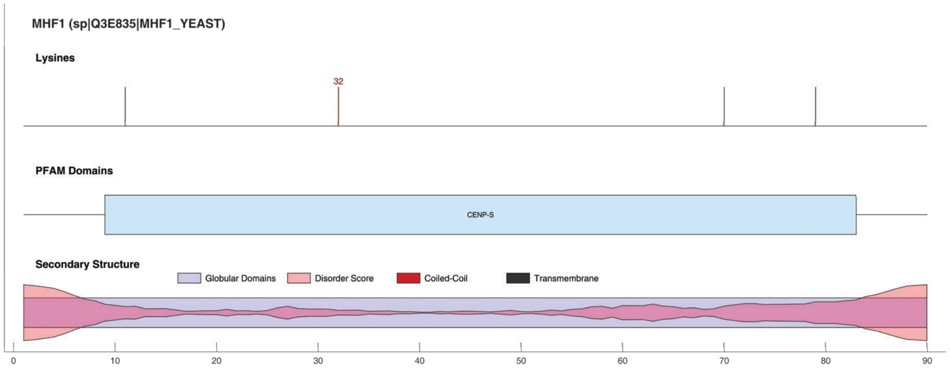
MHF1 (sp|Q3E835|MHF1_YEAST)
Lysines
32
PFAM Domains
CENP-S
Secondary Structure
Globular Domains
Disorder Score
Coiled-Coil
Transmembrane
0 10 20 30 40 50 60 70 80 90

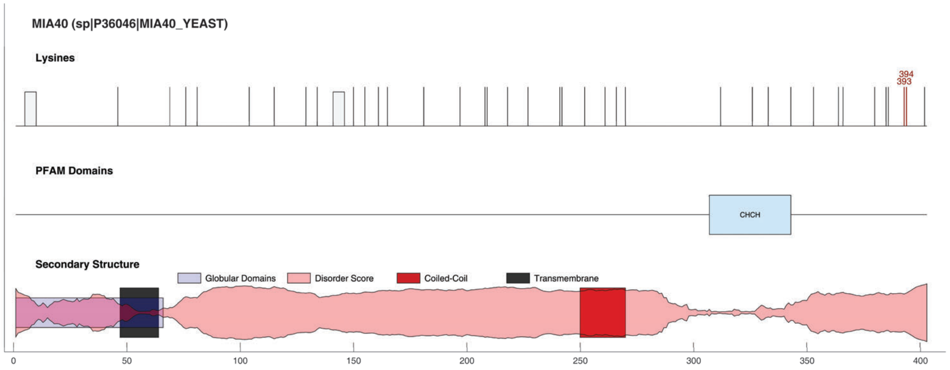
MIA40 (sp|P36046|MIA40_YEAST)
Lysines
394
393
PFAM Domains
CHCH
Secondary Structure
Globular Domains
Disorder Score
Coiled-Coil
Transmembrane
0 50 100 150 200 250 300 350 400

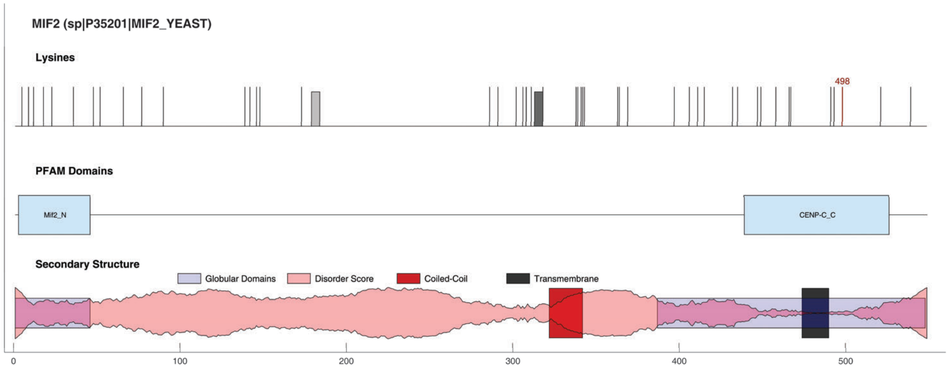
MIF2 (sp|P35201|MIF2_YEAST)
Lysines
498
PFAM Domains
Mif2_N
CENP-C_C
Secondary Structure
Globular Domains
Disorder Score
Coiled-Coil
Transmembrane
0 100 200 300 400 500

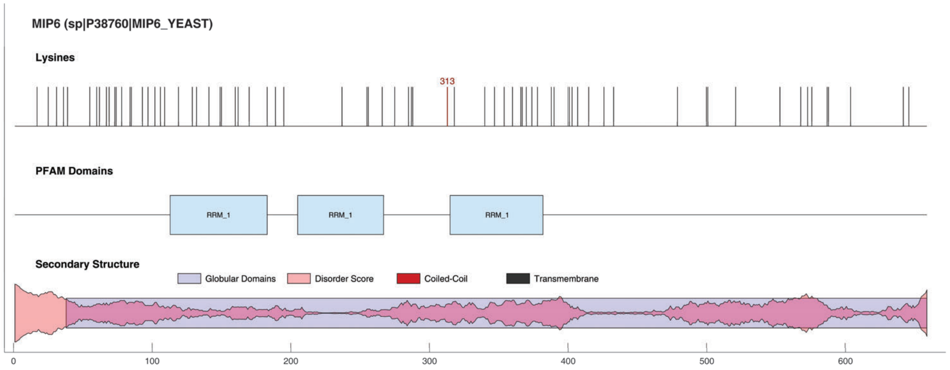
MIP6 (sp|P38760|MIP6_YEAST)
Lysines
313
PFAM Domains
RRM_1
RRM_1
RRM_1
Secondary Structure
Globular Domains
Disorder Score
Coiled-Coil
Transmembrane
0 100 200 300 400 500 600

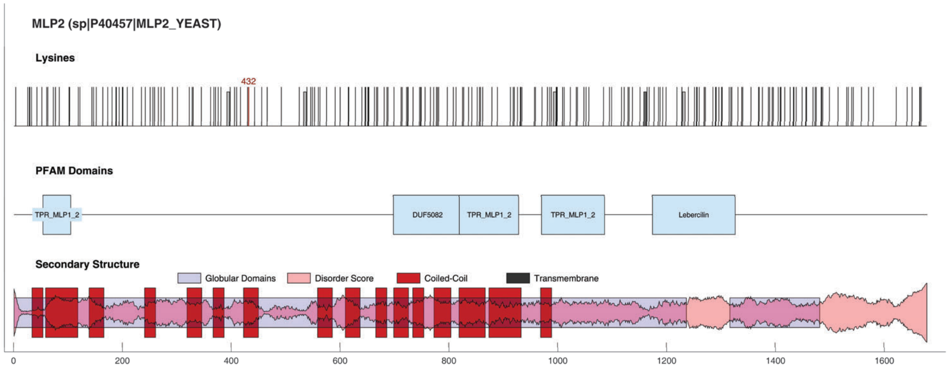
MLP2 (sp|P40457|MLP2_YEAST)
Lysines
432
PFAM Domains
TPR_MLP1_2
DUF5082
TPR_MLP1_2
TPR_MLP1_2
Leberclin
Secondary Structure
Globular Domains
Disorder Score
Coiled-Coil
Transmembrane
0
200
400
600
800
1000
1200
1400
1600

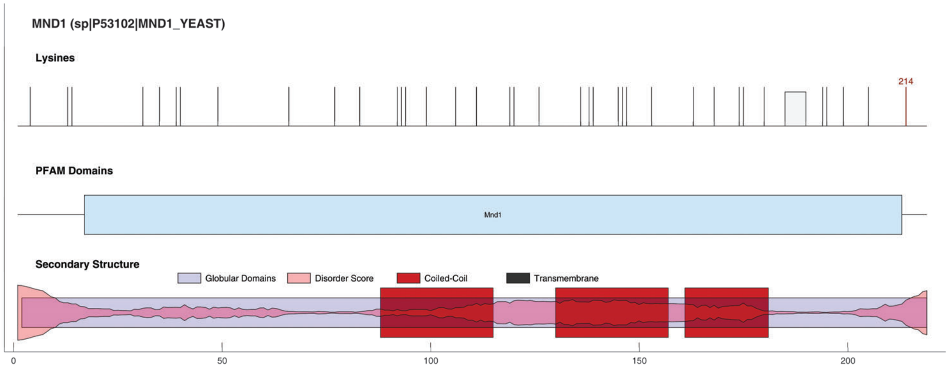
MND1 (sp|P53102|MND1_YEAST)
Lysines
214
PFAM Domains
Mnd1
Secondary Structure
Globular Domains
Disorder Score
Coiled-Coil
Transmembrane
0
50
100
150
200

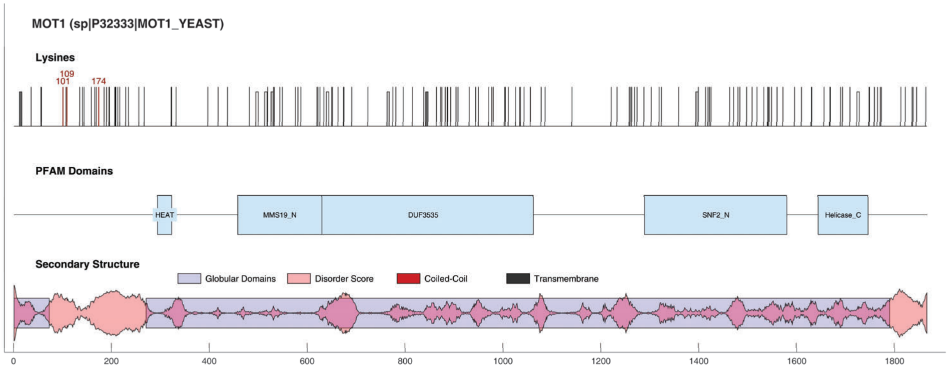
MOT1 (sp|P32333|MOT1_YEAST)
Lysines
109
101
174
PFAM Domains
HEAT
MMS19_N
DUF3535
SNF2_N
Helicase_C
Secondary Structure
Globular Domains
Disorder Score
Coiled-Coil
Transmembrane
0
200
400
600
800
1000
1200
1400
1600
1800

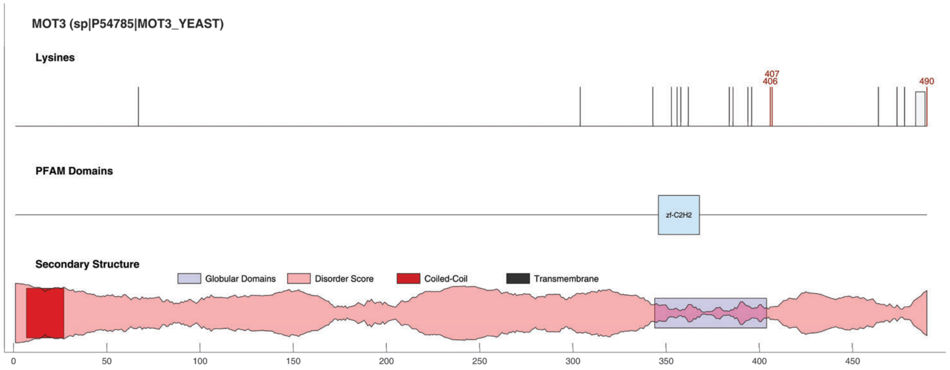
MOT3 (sp|P54785|MOT3_YEAST)
Lysines
407
406
490
PFAM Domains
zf-C2H2
Secondary Structure
Globular Domains
Disorder Score
Coiled-Coil
Transmembrane
0
50
100
150
200
250
300
350
400
450

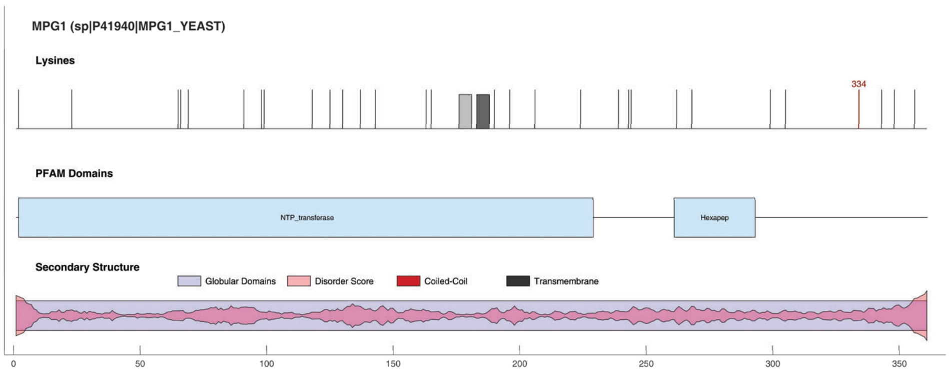
MPG1 (sp|P41940|MPG1_YEAST)
Lysines
334
PFAM Domains
NTP_transferase
Hexapep
Secondary Structure
Globular Domains
Disorder Score
Coiled-Coil
Transmembrane
0
50
100
150
200
250
300
350

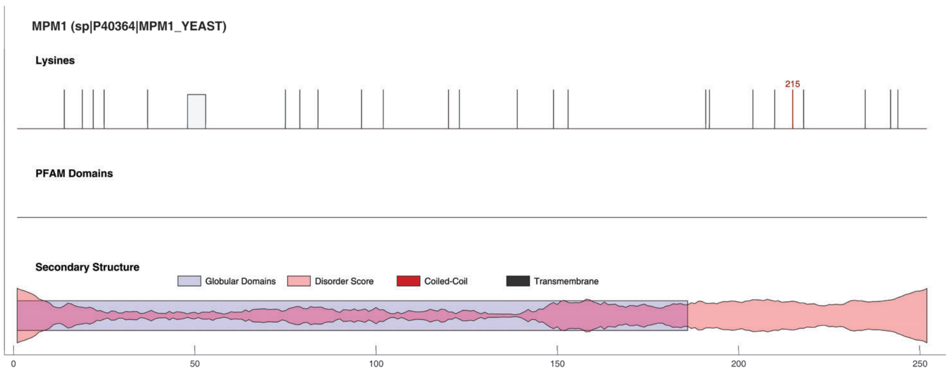
MPM1 (sp|P40364|MPM1_YEAST)
Lysines
215
PFAM Domains
Secondary Structure
Globular Domains
Disorder Score
Coiled-Coil
Transmembrane
0
50
100
150
200
250

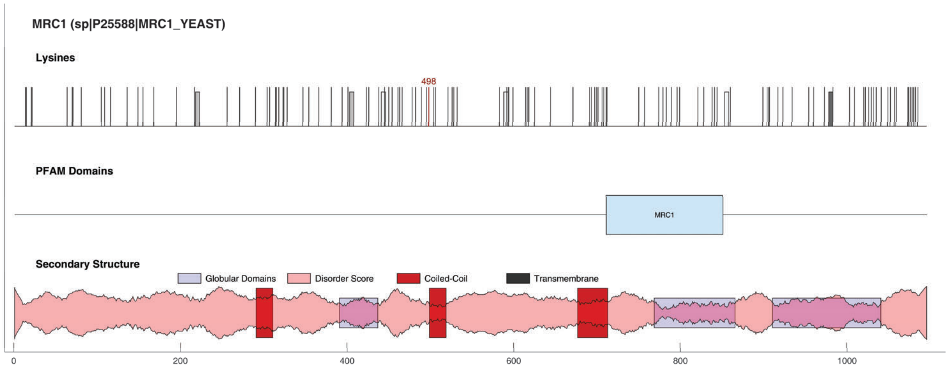
MRC1 (sp|P25588|MRC1_YEAST)
Lysines
498
PFAM Domains
MRC1
Secondary Structure
Globular Domains
Disorder Score
Coiled-Coil
Transmembrane
0
200
400
600
800
1000

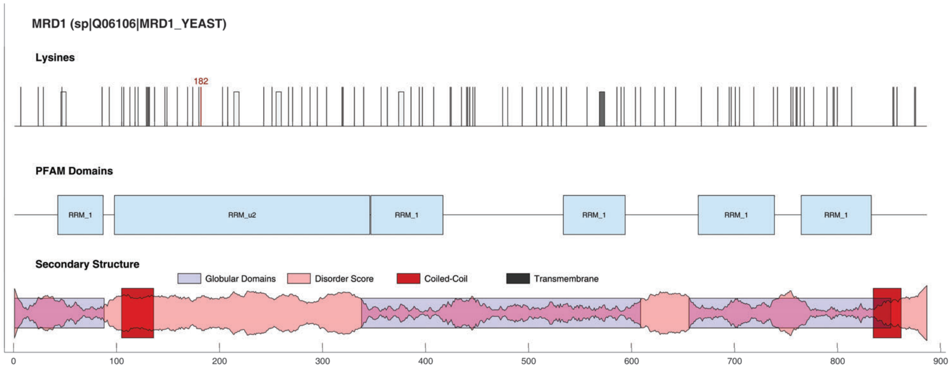
MRD1 (sp|Q06106|MRD1_YEAST)
Lysines
182
PFAM Domains
RRM_1
RRM_u2
RRM_1
RRM_1
RRM_1
RRM_1
Secondary Structure
Globular Domains
Disorder Score
Coiled-Coil
Transmembrane
0
100
200
300
400
500
600
700
800
900

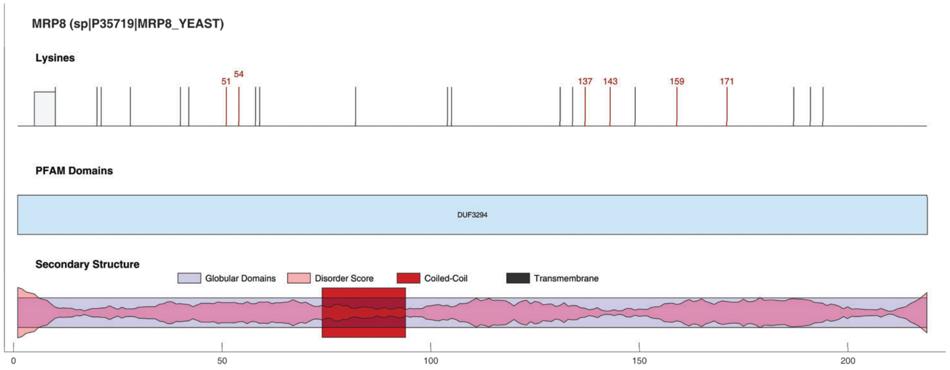
MRP8 (sp|P35719|MRP8_YEAST)
Lysines
51
54
137
143
159
171
PFAM Domains
DUF3294
Secondary Structure
Globular Domains
Disorder Score
Coiled-Coil
Transmembrane
0
50
100
150
200

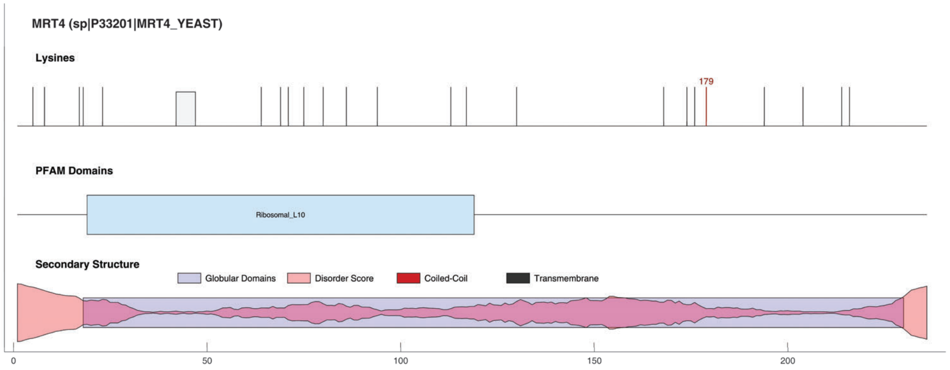
MRT4 (sp|P33201|MRT4_YEAST)
Lysines
179
PFAM Domains
Ribosomal_L10
Secondary Structure
Globular Domains
Disorder Score
Coiled-Coil
Transmembrane
0
50
100
150
200

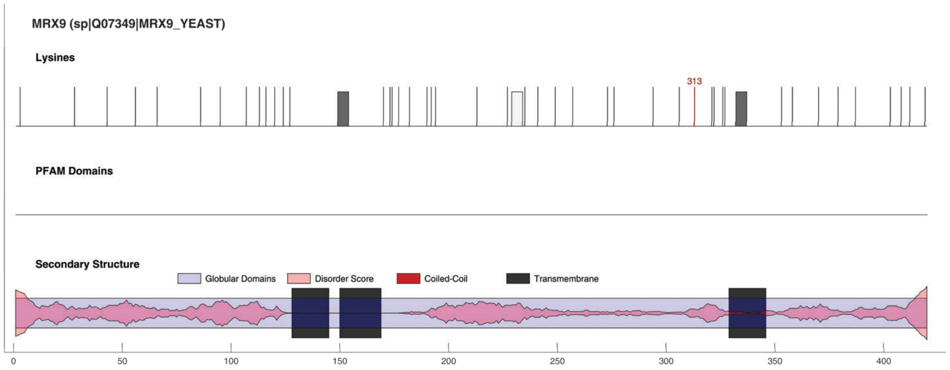
MRX9 (sp|Q07349|MRX9_YEAST)
Lysines
313
PFAM Domains
Secondary Structure
Globular Domains
Disorder Score
Coiled-Coil
Transmembrane
0
50
100
150
200
250
300
350
400

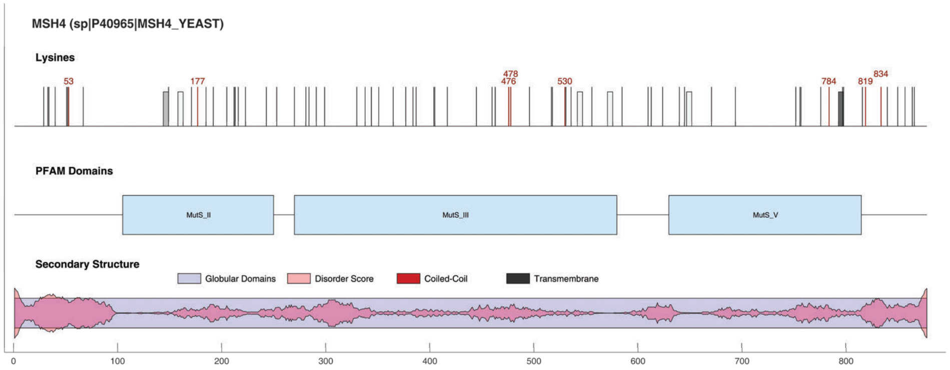
MSH4 (sp|P40965|MSH4_YEAST)
Lysines
53
177
478
476
530
784
819
834
PFAM Domains
MutS_II
MutS_III
MutS_V
Secondary Structure
Globular Domains
Disorder Score
Coiled-Coil
Transmembrane
0
100
200
300
400
500
600
700
800

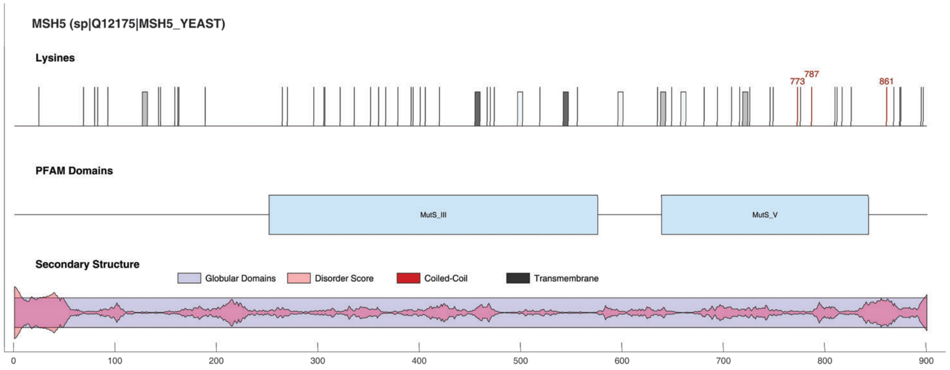
MSH5 (sp|Q12175|MSH5_YEAST)
Lysines
773
787
861
PFAM Domains
MutS_III
MutS_V
Secondary Structure
Globular Domains
Disorder Score
Coiled-Coil
Transmembrane
0
100
200
300
400
500
600
700
800
900

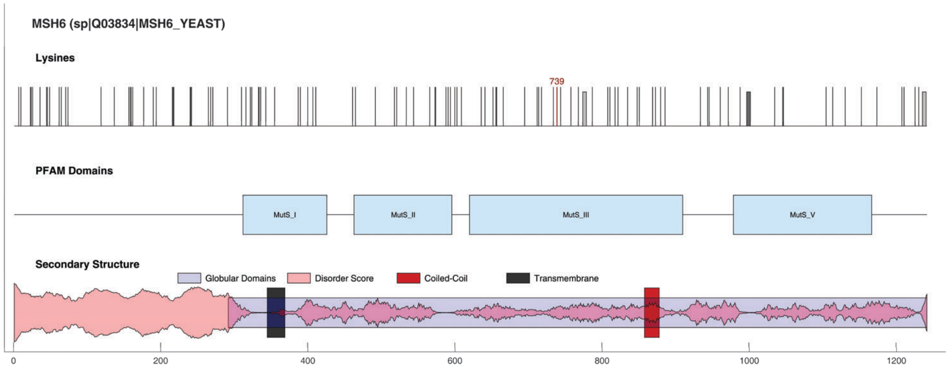
MSH6 (sp|Q03834|MSH6_YEAST)
Lysines
739
PFAM Domains
MutS_I
MutS_II
MutS_III
MutS_V
Secondary Structure
Globular Domains
Disorder Score
Coiled-Coil
Transmembrane
0
200
400
600
800
1000
1200

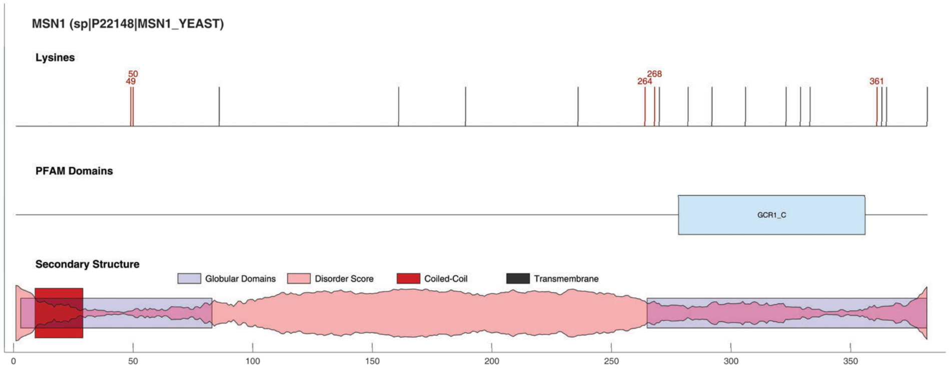
MSN1 (sp|P22148|MSN1_YEAST)
Lysines
49
50
264
268
361
PFAM Domains
GCR1_C
Secondary Structure
Globular Domains
Disorder Score
Coiled-Coil
Transmembrane
0
50
100
150
200
250
300
350

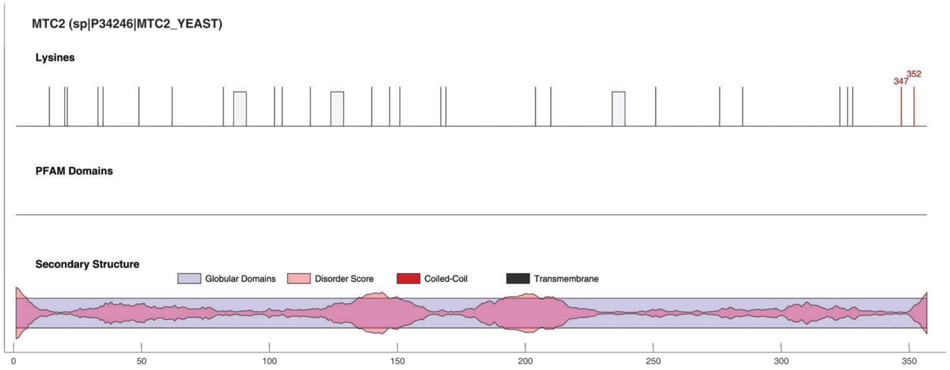
MTC2 (sp|P34246|MTC2_YEAST)
Lysines
347
352
PFAM Domains
Secondary Structure
Globular Domains
Disorder Score
Coiled-Coil
Transmembrane
0
50
100
150
200
250
300
350

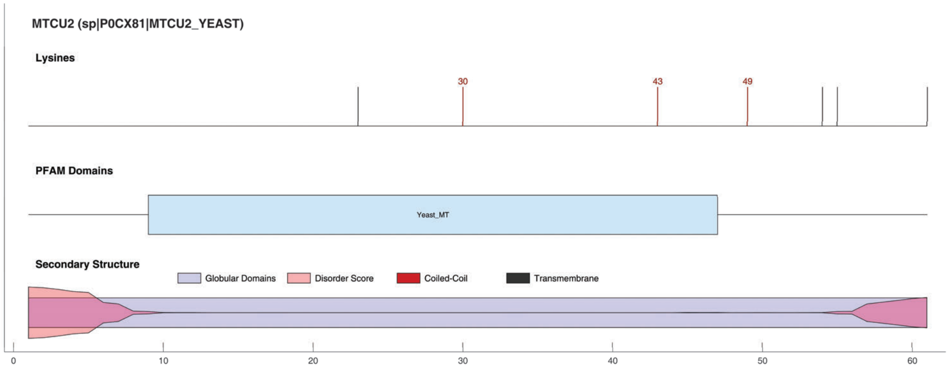
MTCU2 (sp|P0CX81|MTCU2_YEAST)
Lysines
30
43
49
PFAM Domains
Yeast_MT
Secondary Structure
Globular Domains
Disorder Score
Coiled-Coil
Transmembrane
0
10
20
30
40
50
60

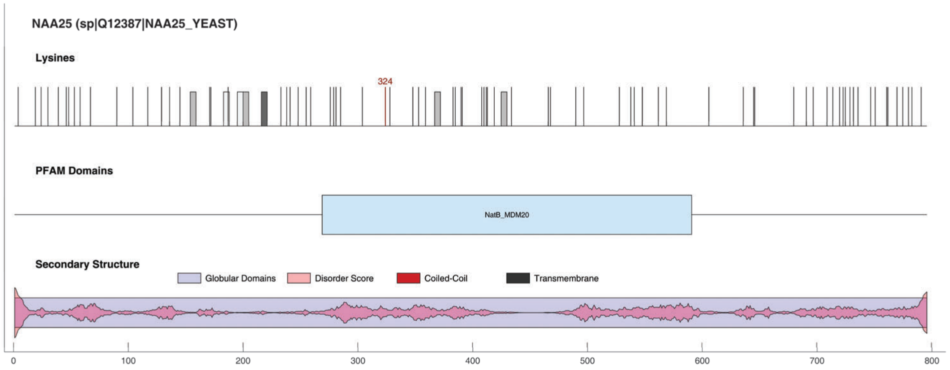
NAA25 (sp|Q12387|NAA25_YEAST)
Lysines
324
PFAM Domains
NatB_MDM20
Secondary Structure
Globular Domains
Disorder Score
Coiled-Coil
Transmembrane
0
100
200
300
400
500
600
700
800

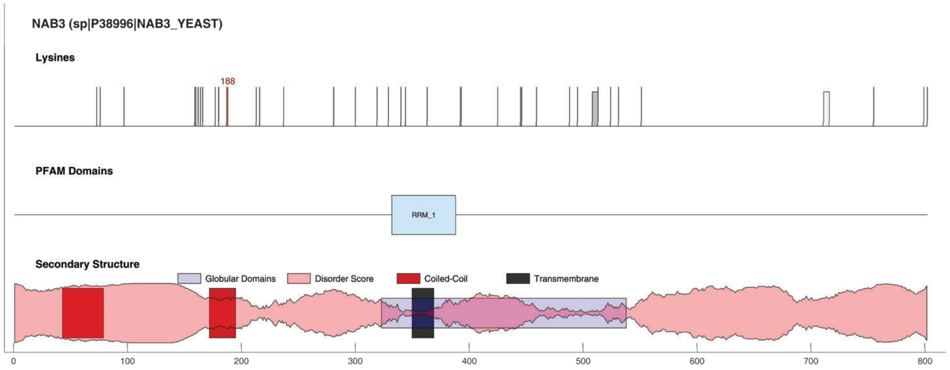
NAB3 (sp|P38996|NAB3_YEAST)
Lysines
188
PFAM Domains
RRM_1
Secondary Structure
Globular Domains
Disorder Score
Coiled-Coil
Transmembrane
0
100
200
300
400
500
600
700
800

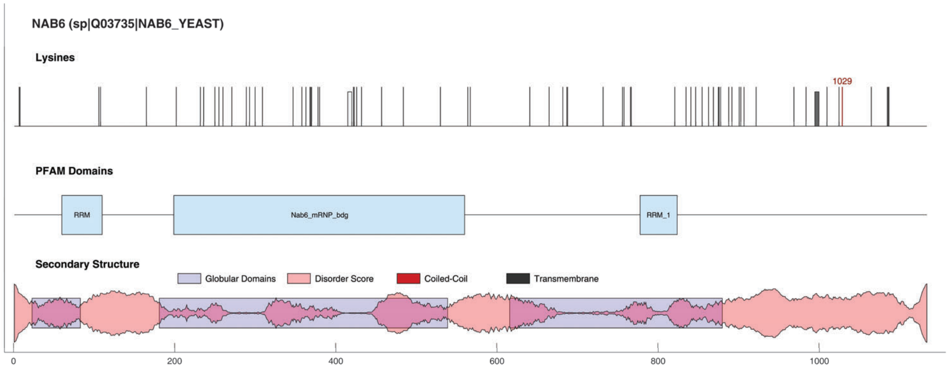
NAB6 (sp|Q03735|NAB6_YEAST)
Lysines
1029
PFAM Domains
RRM
Nab6_mRNP_bdg
RRM_1
Secondary Structure
Globular Domains
Disorder Score
Coiled-Coil
Transmembrane
0
200
400
600
800
1000

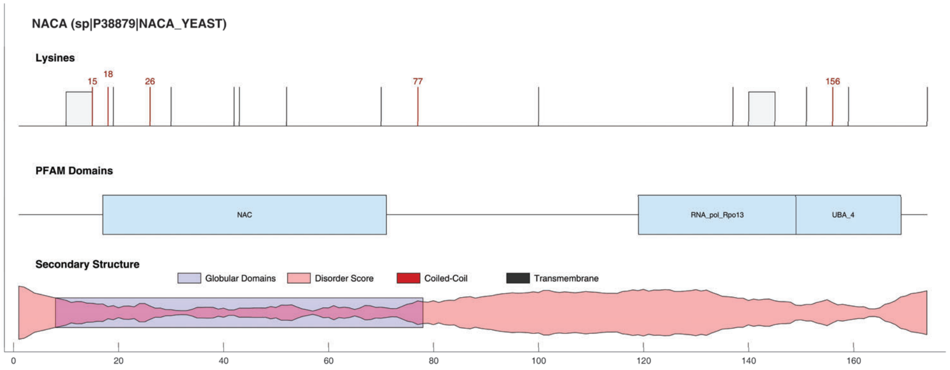
NACA (sp|P38879|NACA_YEAST)
Lysines
15
18
26
77
156
PFAM Domains
NAC
RNA_pol_Rpo13
UBA_4
Secondary Structure
Globular Domains
Disorder Score
Coiled-Coil
Transmembrane
0
20
40
60
80
100
120
140
160

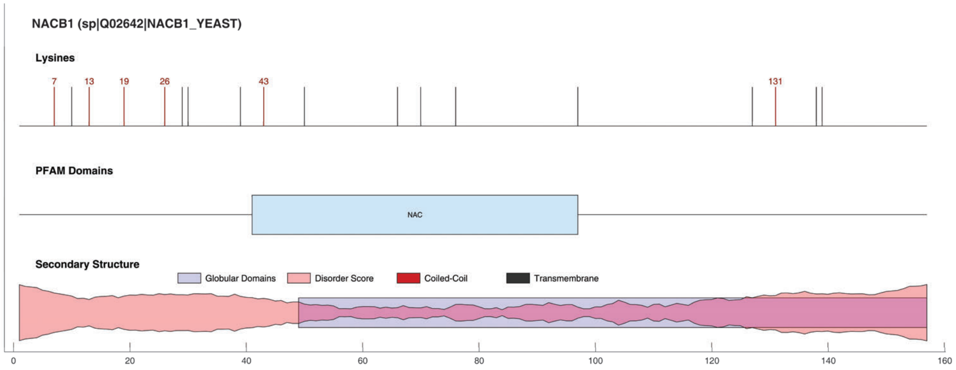
NACB1 (sp|Q02642|NACB1_YEAST)
Lysines
7
13
19
26
43
131
PFAM Domains
NAC
Secondary Structure
Globular Domains
Disorder Score
Coiled-Coil
Transmembrane
0
20
40
60
80
100
120
140
160

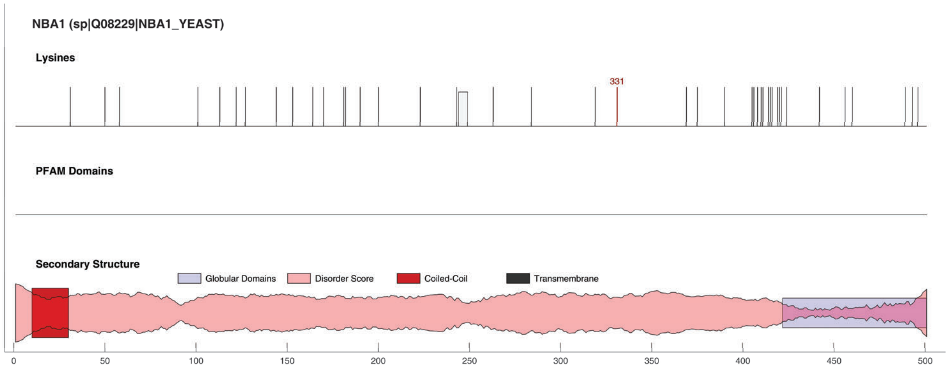
NBA1 (sp|Q08229|NBA1_YEAST)
Lysines
331
PFAM Domains
Secondary Structure
Globular Domains
Disorder Score
Coiled-Coil
Transmembrane
0
50
100
150
200
250
300
350
400
450
500

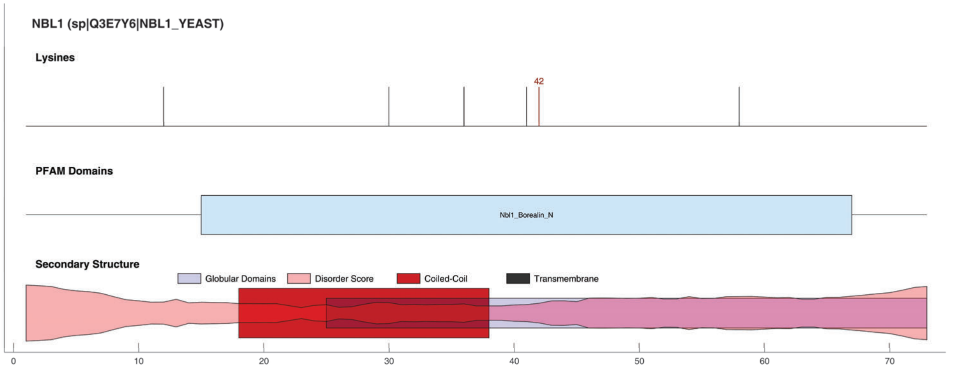
NBL1 (sp|Q3E7Y6|NBL1_YEAST)
Lysines
42
PFAM Domains
Nbl1_Borealin_N
Secondary Structure
Globular Domains
Disorder Score
Coiled-Coil
Transmembrane
0
10
20
30
40
50
60
70

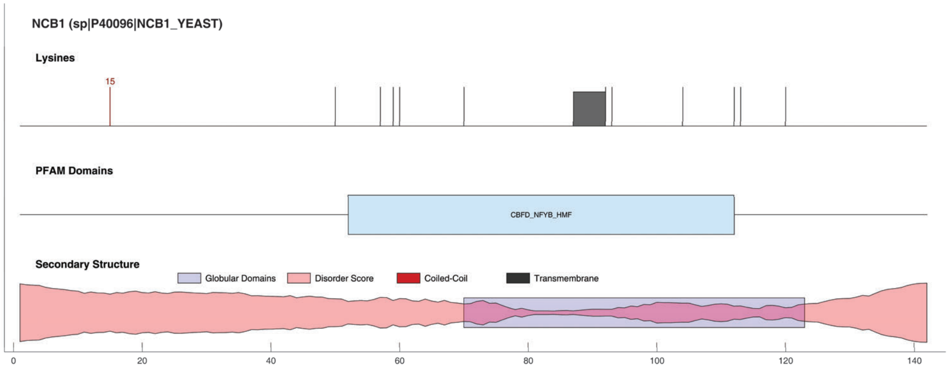

NCB1 (sp|P40096|NCB1_YEAST)
Lysines
15
PFAM Domains
CBFD_NFYB_HMF
Secondary Structure
Globular Domains
Disorder Score
Coiled-Coil
Transmembrane
0
20
40
60
80
100
120
140

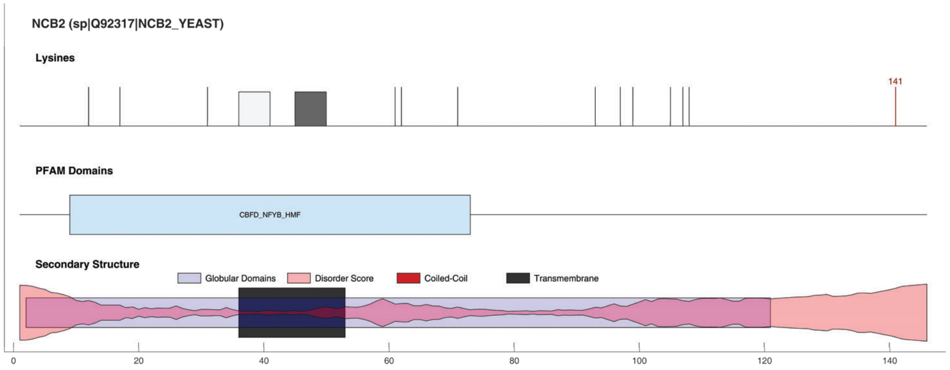

NCB2 (sp|Q92317|NCB2_YEAST)
Lysines
141
PFAM Domains
CBFD_NFYB_HMF
Secondary Structure
Globular Domains
Disorder Score
Coiled-Coil
Transmembrane
0
20
40
60
80
100
120
140

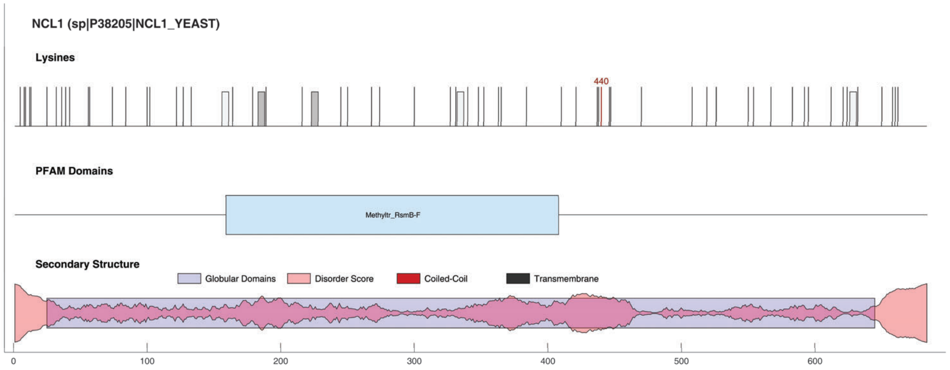

NCL1 (sp|P38205|NCL1_YEAST)
Lysines
440
PFAM Domains
Methyltr_RsmB-F
Secondary Structure
Globular Domains
Disorder Score
Coiled-Coil
Transmembrane
0
100
200
300
400
500
600

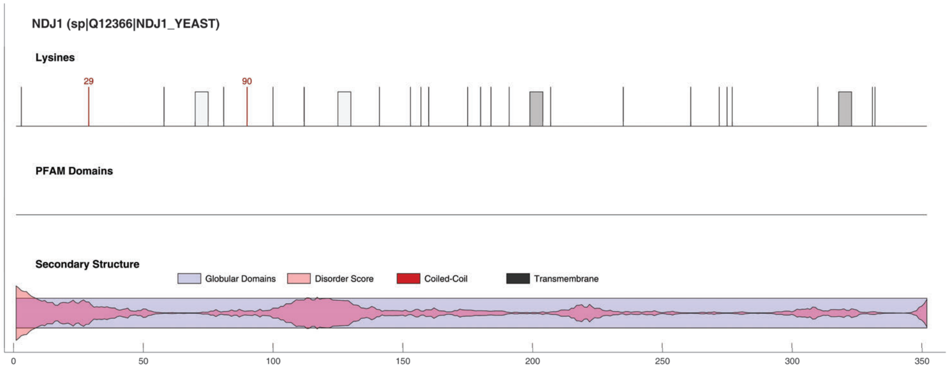

NDJ1 (sp|Q12366|NDJ1_YEAST)
Lysines
29
90
PFAM Domains
Secondary Structure
Globular Domains
Disorder Score
Coiled-Coil
Transmembrane
0
50
100
150
200
250
300
350

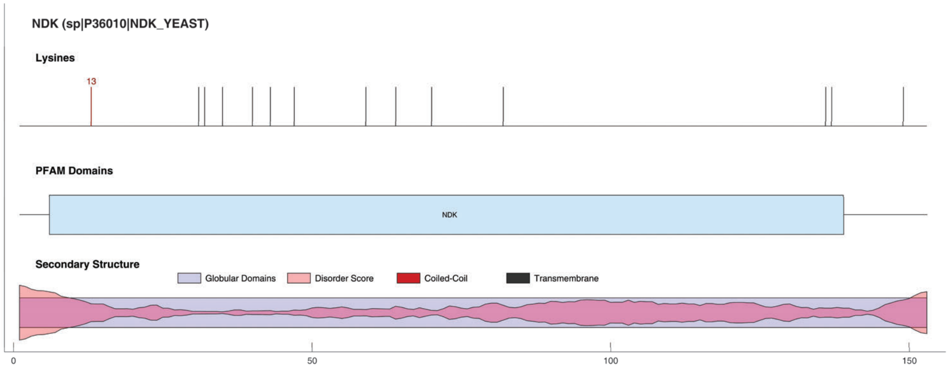
NDK (sp|P36010|NDK_YEAST)
Lysines
13
PFAM Domains
NDK
Secondary Structure
Globular Domains
Disorder Score
Coiled-Coil
Transmembrane
0
50
100
150

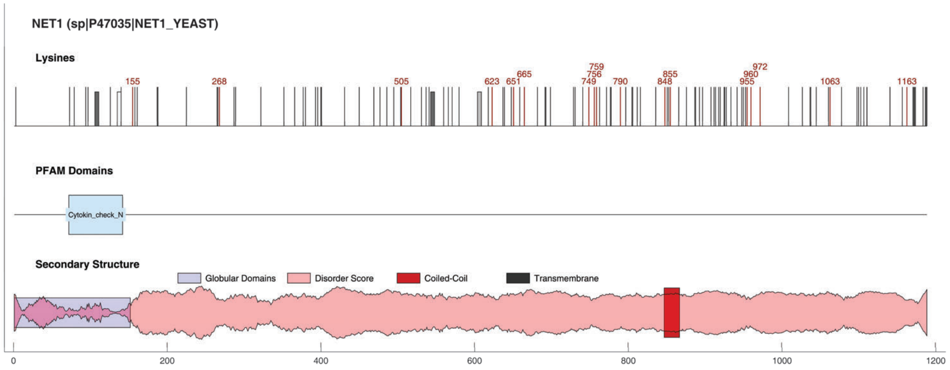
NET1 (sp|P47035|NET1_YEAST)
Lysines
155
268
505
623
651
665
749
756
759
790
848
855
955
960
972
1063
1163
PFAM Domains
Cytokin_check_N
Secondary Structure
Globular Domains
Disorder Score
Coiled-Coil
Transmembrane
0
200
400
600
800
1000
1200

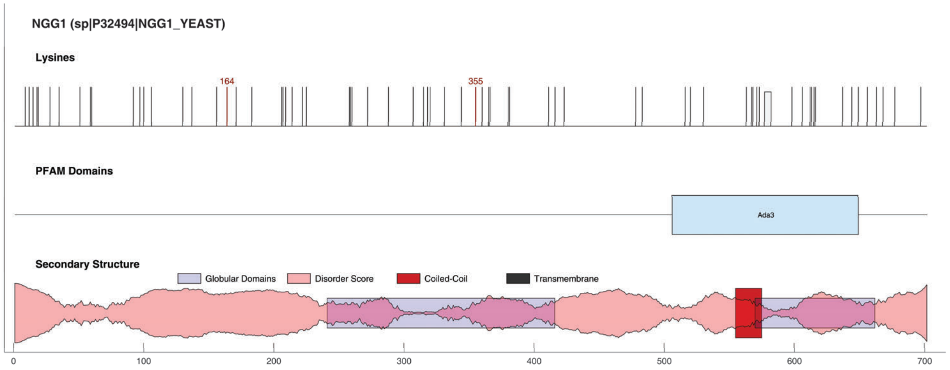
NGG1 (sp|P32494|NGG1_YEAST)
Lysines
164
355
PFAM Domains
Ada3
Secondary Structure
Globular Domains
Disorder Score
Coiled-Coil
Transmembrane
0
100
200
300
400
500
600
700

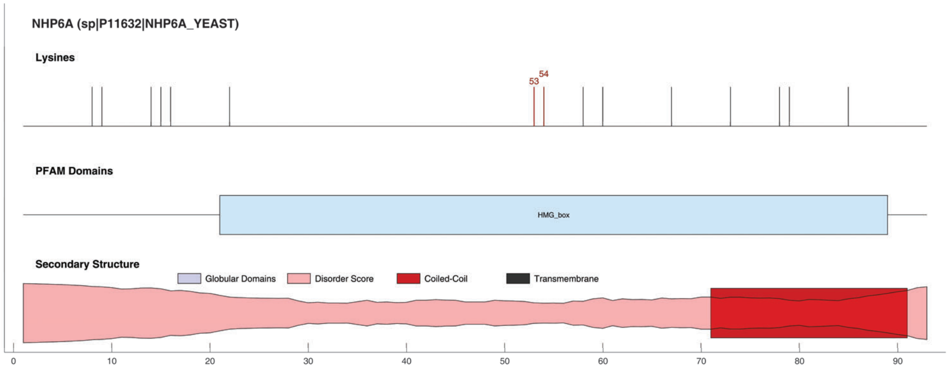
NHP6A (sp|P11632|NHP6A_YEAST)
Lysines
53
54
PFAM Domains
HMG_box
Secondary Structure
Globular Domains
Disorder Score
Coiled-Coil
Transmembrane
0
10
20
30
40
50
60
70
80
90

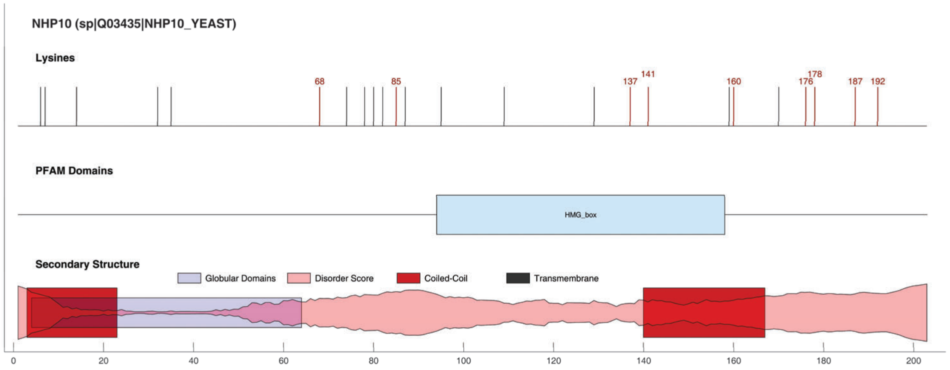

NHP10 (sp|Q03435|NHP10_YEAST)
Lysines
68
85
137
141
160
176
178
187
192
PFAM Domains
HMG_box
Secondary Structure
Globular Domains
Disorder Score
Coiled-Coil
Transmembrane
0
20
40
60
80
100
120
140
160
180
200

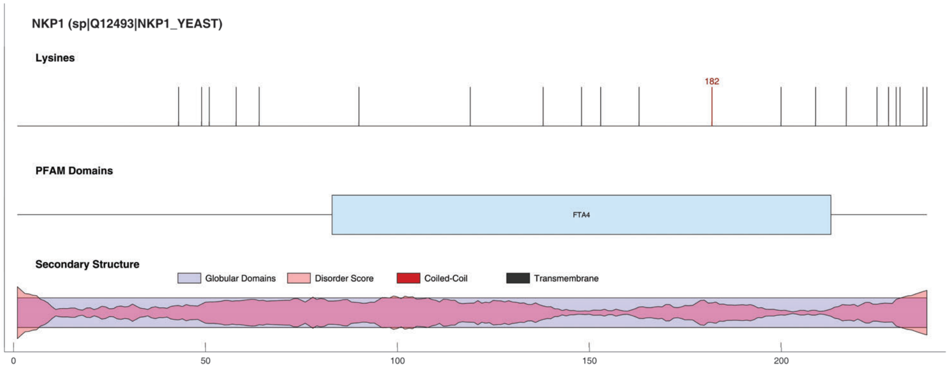

NKP1 (sp|Q12493|NKP1_YEAST)
Lysines
182
PFAM Domains
FTA4
Secondary Structure
Globular Domains
Disorder Score
Coiled-Coil
Transmembrane
0
50
100
150
200

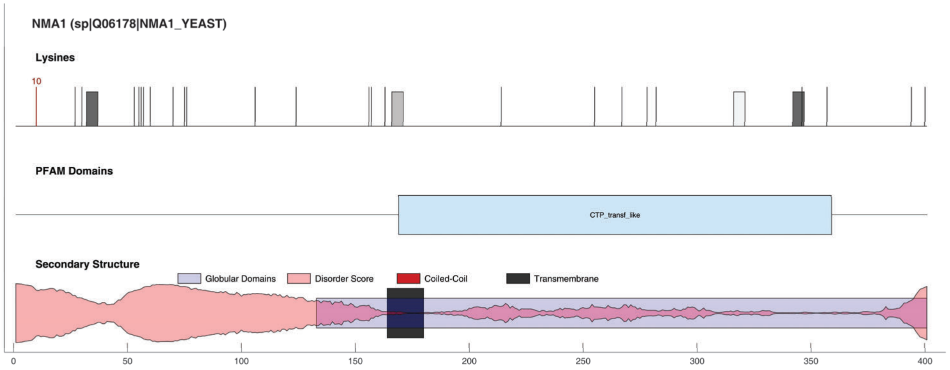

NMA1 (sp|Q06178|NMA1_YEAST)
Lysines
10
PFAM Domains
CTP_transf_like
Secondary Structure
Globular Domains
Disorder Score
Coiled-Coil
Transmembrane
0
50
100
150
200
250
300
350
400

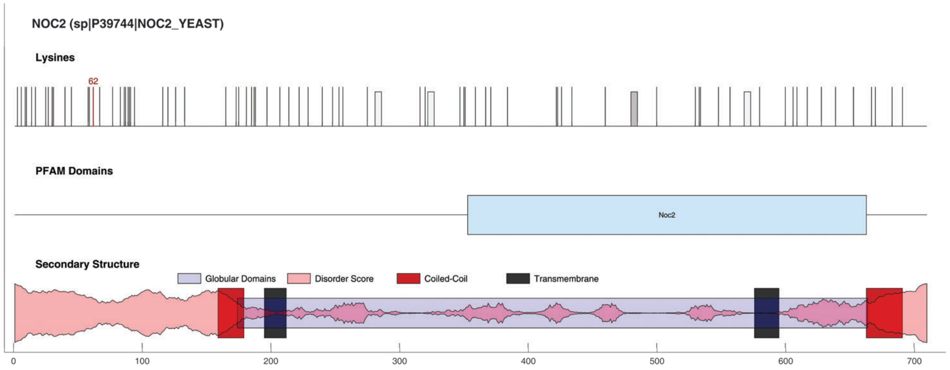

NOC2 (sp|P39744|NOC2_YEAST)
Lysines
62
PFAM Domains
Noc2
Secondary Structure
Globular Domains
Disorder Score
Coiled-Coil
Transmembrane
0
100
200
300
400
500
600
700

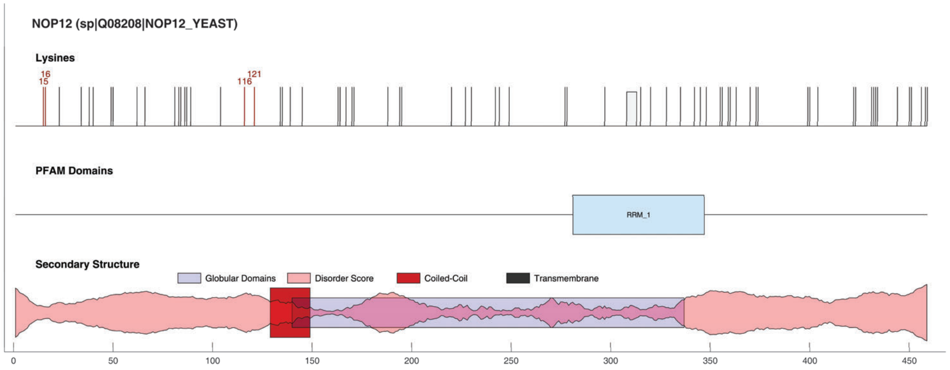
NOP12 (sp|Q08208|NOP12_YEAST)
Lysines
15
16
116
121
PFAM Domains
RRM_1
Secondary Structure
Globular Domains
Disorder Score
Coiled-Coil
Transmembrane
0
50
100
150
200
250
300
350
400
450

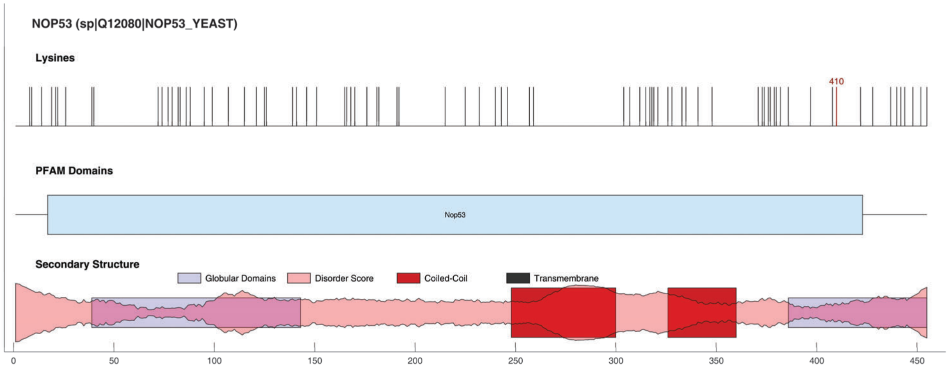
NOP53 (sp|Q12080|NOP53_YEAST)
Lysines
410
PFAM Domains
Nop53
Secondary Structure
Globular Domains
Disorder Score
Coiled-Coil
Transmembrane
0
50
100
150
200
250
300
350
400
450

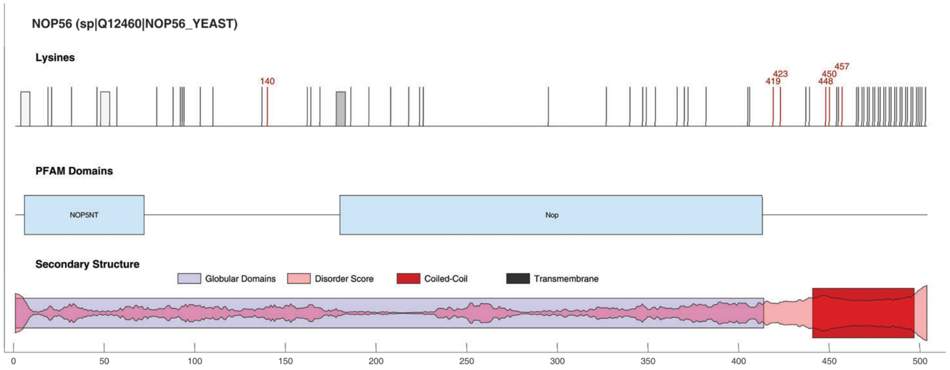
NOP56 (sp|Q12460|NOP56_YEAST)
Lysines
140
419
423
448
450
457
PFAM Domains
NOP5NT
Nop
Secondary Structure
Globular Domains
Disorder Score
Coiled-Coil
Transmembrane
0
50
100
150
200
250
300
350
400
450
500

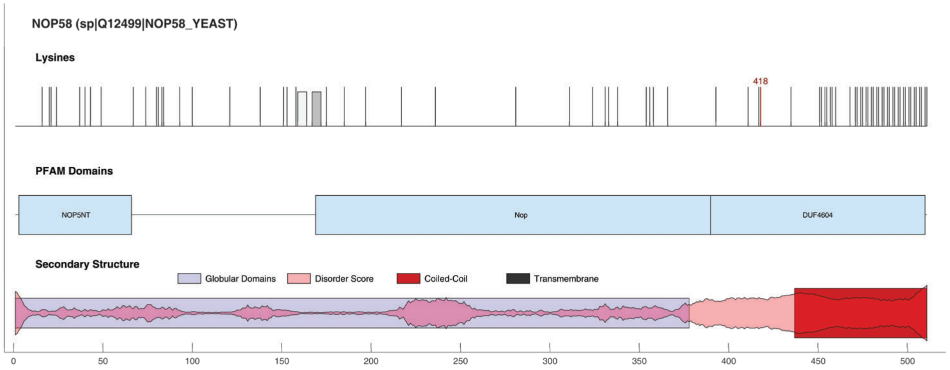
NOP58 (sp|Q12499|NOP58_YEAST)
Lysines
418
PFAM Domains
NOP5NT
Nop
DUF4604
Secondary Structure
Globular Domains
Disorder Score
Coiled-Coil
Transmembrane
0
50
100
150
200
250
300
350
400
450
500

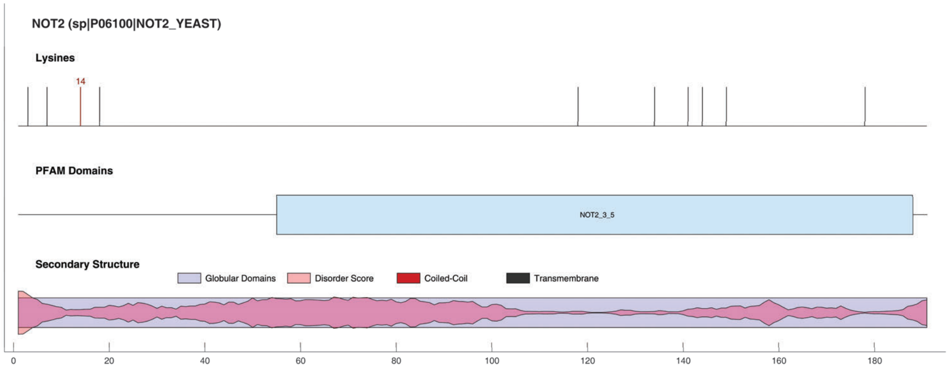
NOT2 (sp|P06100|NOT2_YEAST)
Lysines
14
PFAM Domains
NOT2_3_5
Secondary Structure
Globular Domains
Disorder Score
Coiled-Coil
Transmembrane
0
20
40
60
80
100
120
140
160
180

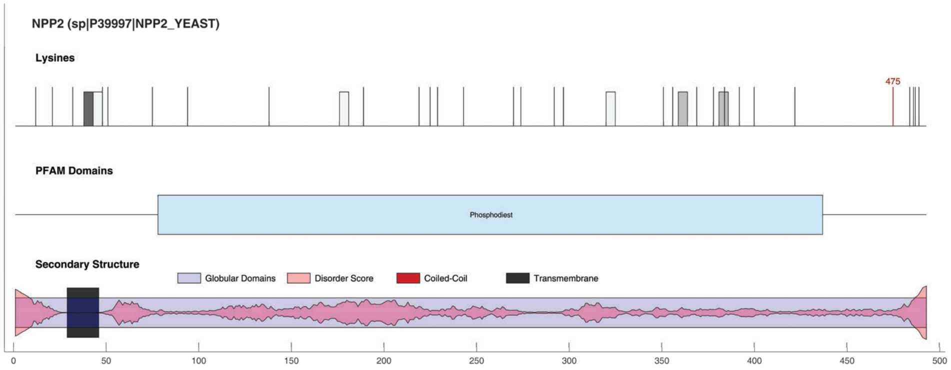
NPP2 (sp|P39997|NPP2_YEAST)
Lysines
475
PFAM Domains
Phosphodiest
Secondary Structure
Globular Domains
Disorder Score
Coiled-Coil
Transmembrane
0
50
100
150
200
250
300
350
400
450
500

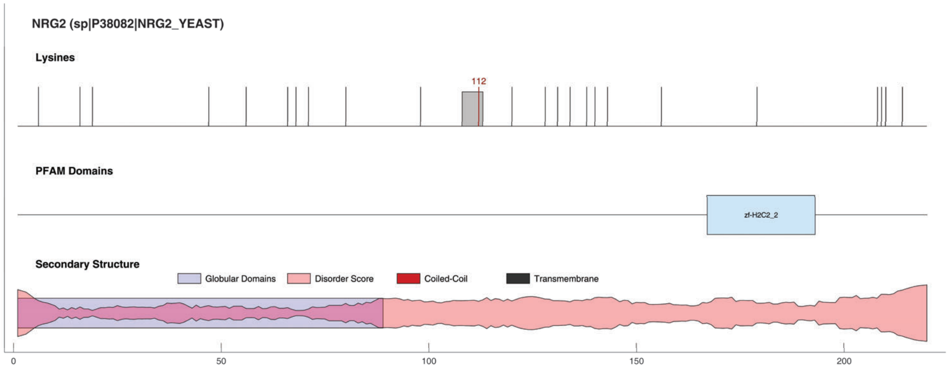
NRG2 (sp|P38082|NRG2_YEAST)
Lysines
112
PFAM Domains
zf-H2C2_2
Secondary Structure
Globular Domains
Disorder Score
Coiled-Coil
Transmembrane
0
50
100
150
200

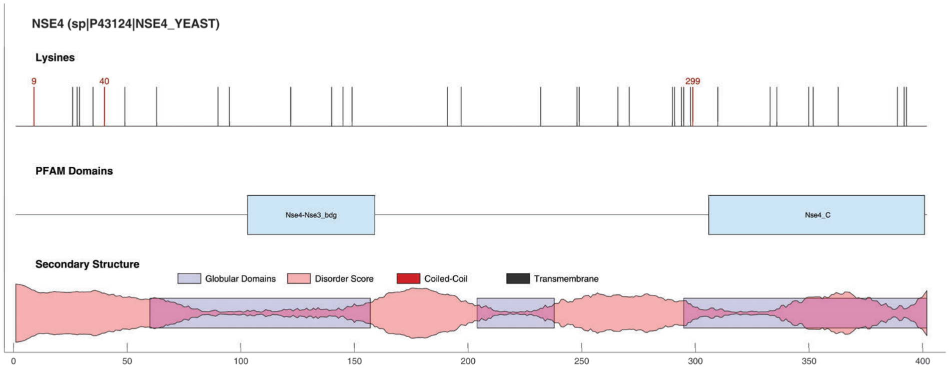
NSE4 (sp|P43124|NSE4_YEAST)
Lysines
9
40
299
PFAM Domains
Nse4-Nse3_bdg
Nse4_C
Secondary Structure
Globular Domains
Disorder Score
Coiled-Coil
Transmembrane
0
50
100
150
200
250
300
350
400

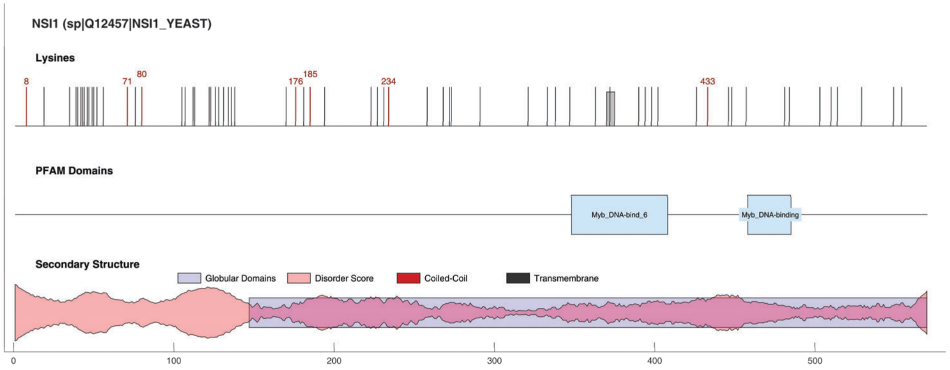
NSI1 (sp|Q12457|NSI1_YEAST)
Lysines
8
71
80
176
185
234
433
PFAM Domains
Myb_DNA-bind_6
Myb_DNA-binding
Secondary Structure
Globular Domains
Disorder Score
Coiled-Coil
Transmembrane
0
100
200
300
400
500

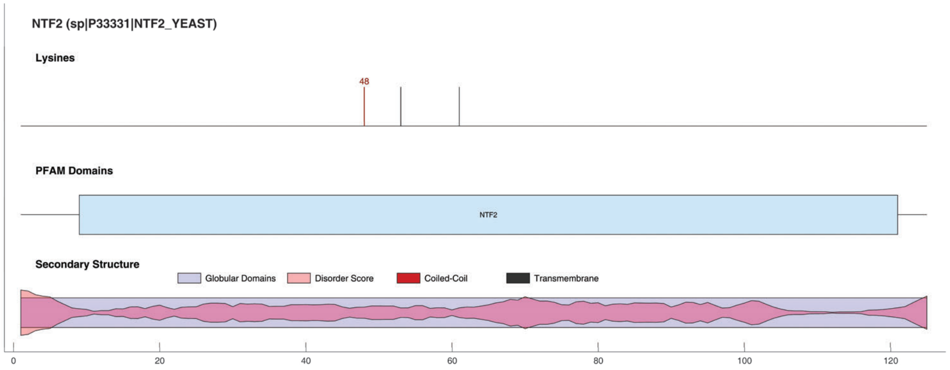
NTF2 (sp|P33331|NTF2_YEAST)
Lysines
48
PFAM Domains
NTF2
Secondary Structure
Globular Domains
Disorder Score
Coiled-Coil
Transmembrane
0
20
40
60
80
100
120

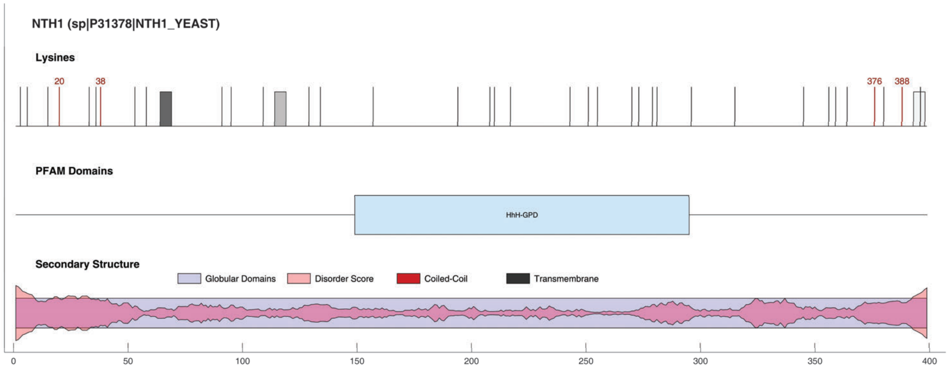
NTH1 (sp|P31378|NTH1_YEAST)
Lysines
20
38
376
388
PFAM Domains
HhH-GPD
Secondary Structure
Globular Domains
Disorder Score
Coiled-Coil
Transmembrane
0
50
100
150
200
250
300
350
400

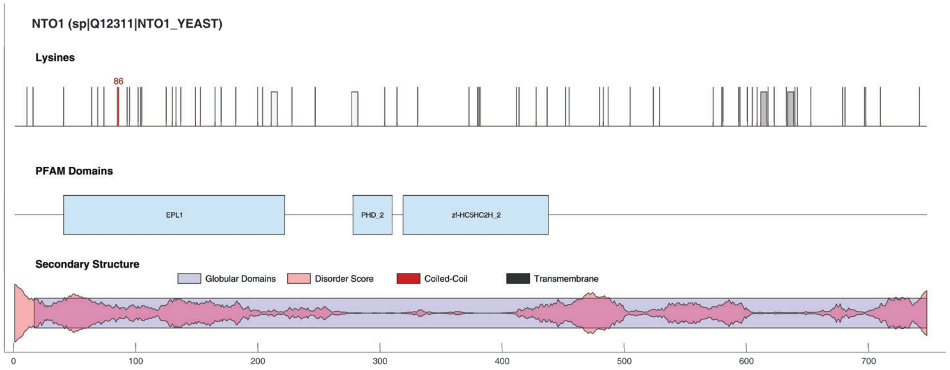
NTO1 (sp|Q12311|NTO1_YEAST)
Lysines
86
PFAM Domains
EPL1
PHD_2
zf-HC5HC2H_2
Secondary Structure
Globular Domains
Disorder Score
Coiled-Coil
Transmembrane
0
100
200
300
400
500
600
700

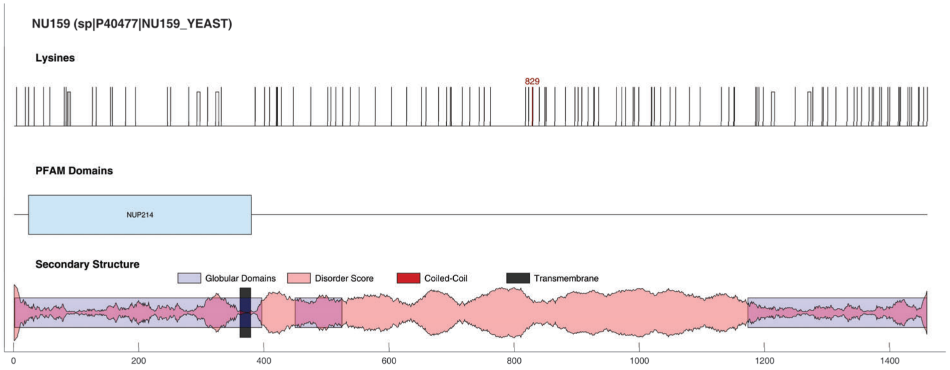
NU159 (sp|P40477|NU159_YEAST)
Lysines
829
PFAM Domains
NUP214
Secondary Structure
Globular Domains
Disorder Score
Coiled-Coil
Transmembrane
0
200
400
600
800
1000
1200
1400

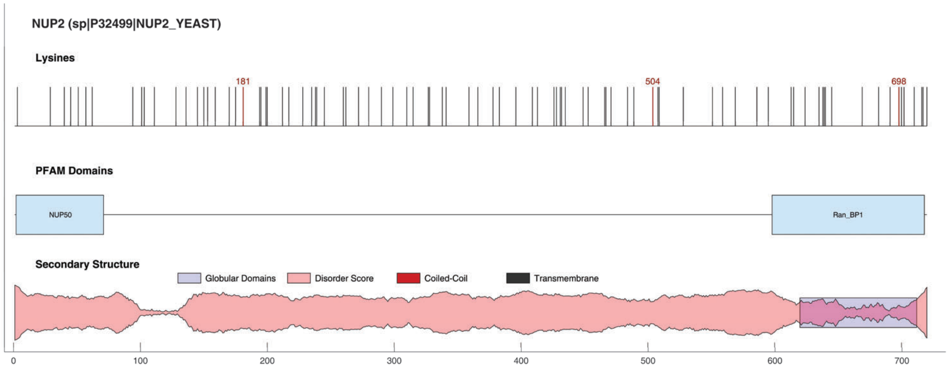
NUP2 (sp|P32499|NUP2_YEAST)
Lysines
181
504
698
PFAM Domains
NUP50
Ran_BP1
Secondary Structure
Globular Domains
Disorder Score
Coiled-Coil
Transmembrane
0
100
200
300
400
500
600
700

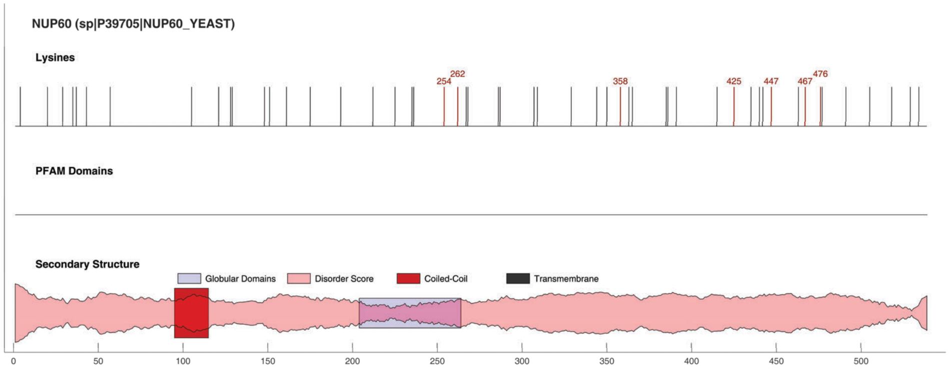
NUP60 (sp|P39705|NUP60_YEAST)
Lysines
254
262
358
425
447
467
476
PFAM Domains
Secondary Structure
Globular Domains
Disorder Score
Coiled-Coil
Transmembrane
0
50
100
150
200
250
300
350
400
450
500

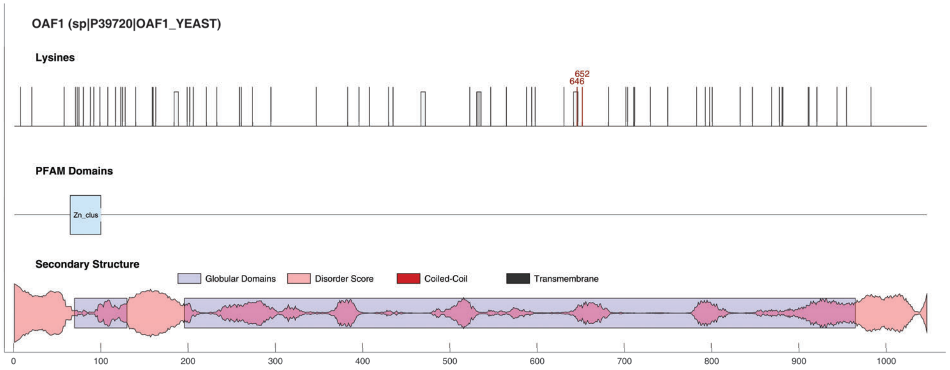
OAF1 (sp|P39720|OAF1_YEAST)
Lysines
646
652
PFAM Domains
Zn_clus
Secondary Structure
Globular Domains
Disorder Score
Coiled-Coil
Transmembrane
0
100
200
300
400
500
600
700
800
900
1000

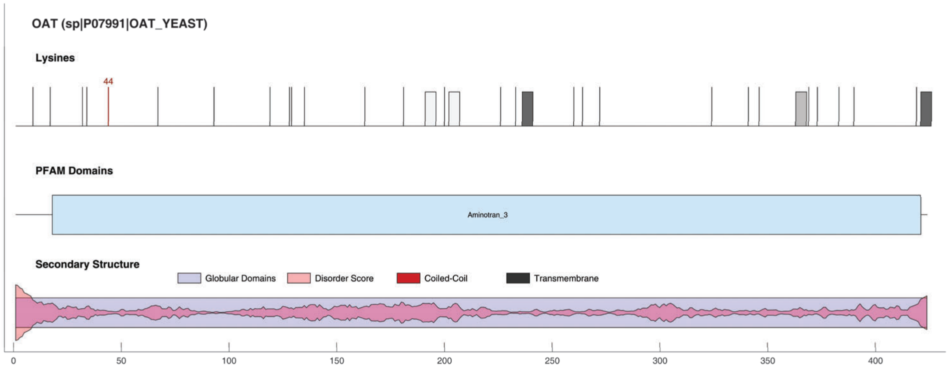
OAT (sp|P07991|OAT_YEAST)
Lysines
44
PFAM Domains
Aminotran_3
Secondary Structure
Globular Domains
Disorder Score
Coiled-Coil
Transmembrane
0
50
100
150
200
250
300
350
400

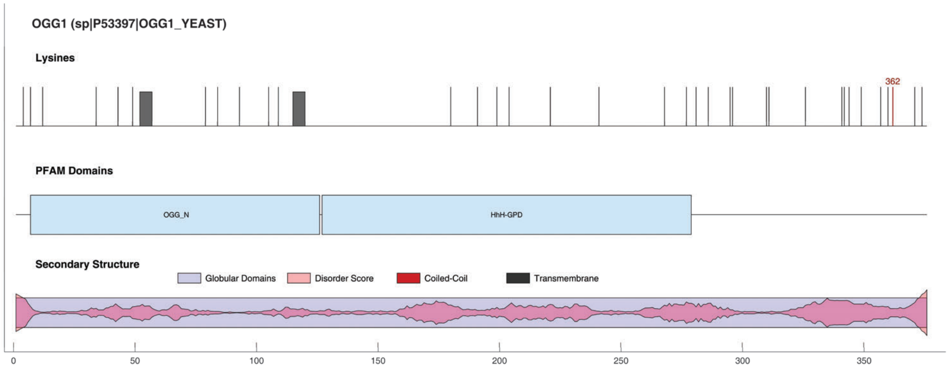
OGG1 (sp|P53397|OGG1_YEAST)
Lysines
362
PFAM Domains
OGG_N
HhH-GPD
Secondary Structure
Globular Domains
Disorder Score
Coiled-Coil
Transmembrane
0
50
100
150
200
250
300
350

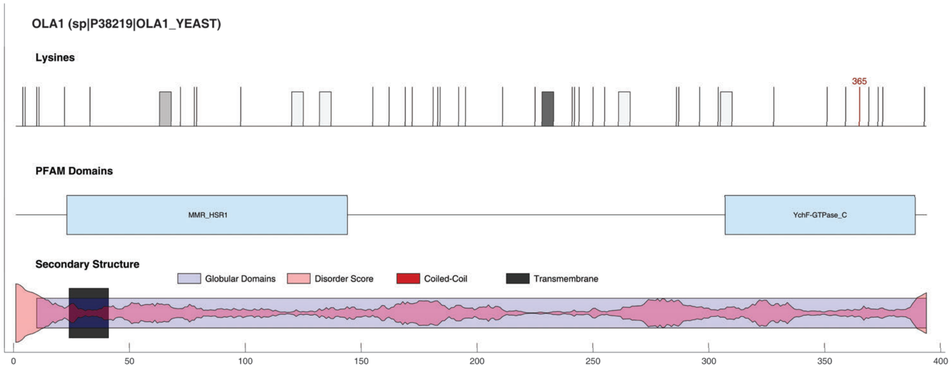
OLA1 (sp|P38219|OLA1_YEAST)
Lysines
365
PFAM Domains
MMR_HSR1
YchF-GTPase_C
Secondary Structure
Globular Domains
Disorder Score
Coiled-Coil
Transmembrane
0
50
100
150
200
250
300
350
400

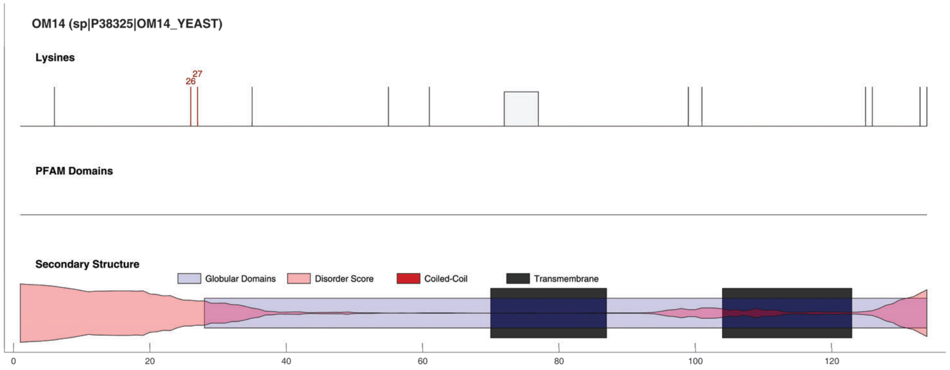
OM14 (sp|P38325|OM14_YEAST)
Lysines
26
27
PFAM Domains
Secondary Structure
Globular Domains
Disorder Score
Coiled-Coil
Transmembrane
0
20
40
60
80
100
120

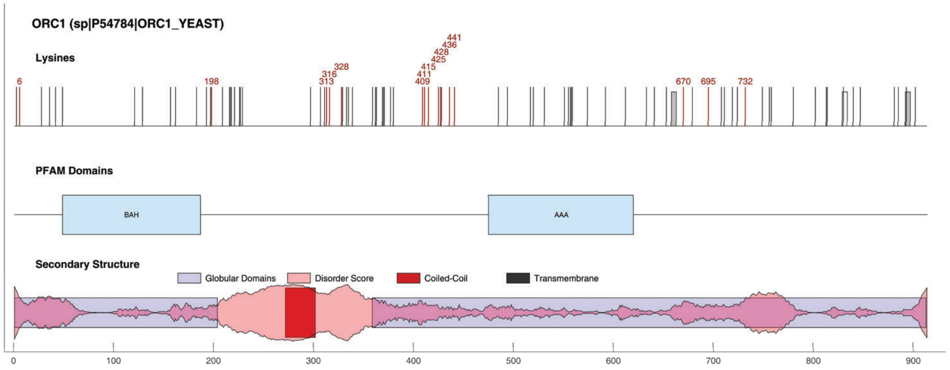

ORC1 (sp|P54784|ORC1_YEAST)
Lysines
6
198
313
316
328
409
411
415
425
428
436
441
670
695
732
PFAM Domains
BAH
AAA
Secondary Structure
Globular Domains
Disorder Score
Coiled-Coil
Transmembrane
0
100
200
300
400
500
600
700
800
900

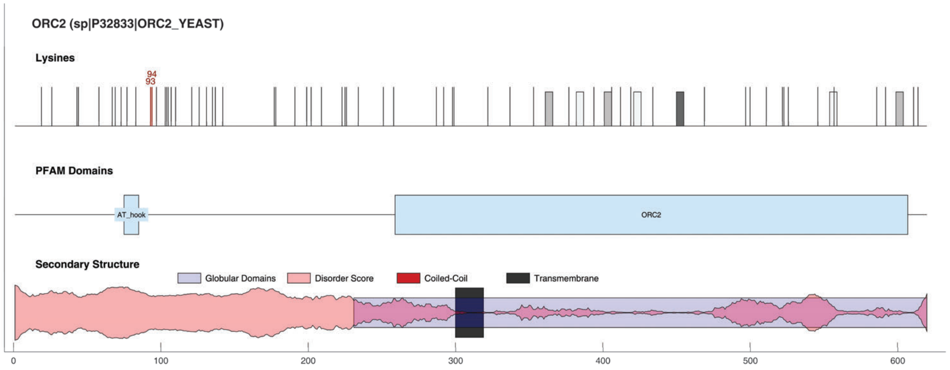

ORC2 (sp|P32833|ORC2_YEAST)
Lysines
93
94
PFAM Domains
AT_hook
ORC2
Secondary Structure
Globular Domains
Disorder Score
Coiled-Coil
Transmembrane
0
100
200
300
400
500
600

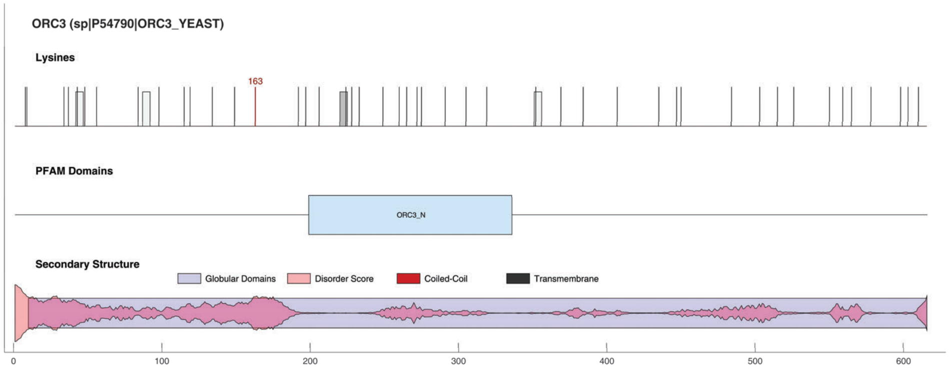

ORC3 (sp|P54790|ORC3_YEAST)
Lysines
163
PFAM Domains
ORC3_N
Secondary Structure
Globular Domains
Disorder Score
Coiled-Coil
Transmembrane
0
100
200
300
400
500
600

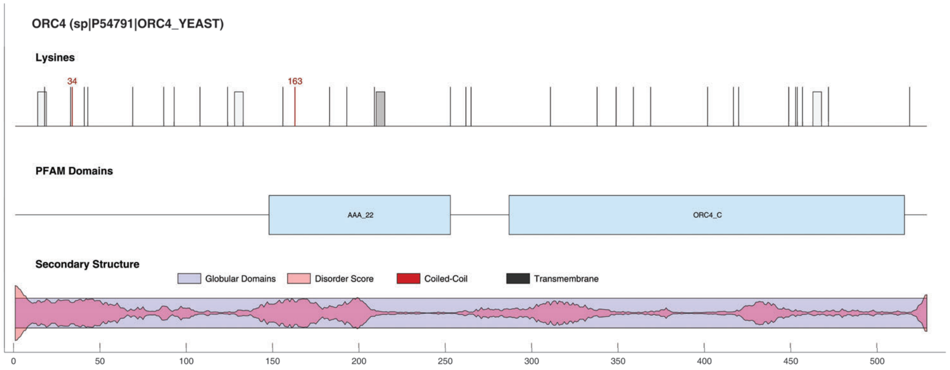

ORC4 (sp|P54791|ORC4_YEAST)
Lysines
34
163
PFAM Domains
AAA_22
ORC4_C
Secondary Structure
Globular Domains
Disorder Score
Coiled-Coil
Transmembrane
0
50
100
150
200
250
300
350
400
450
500

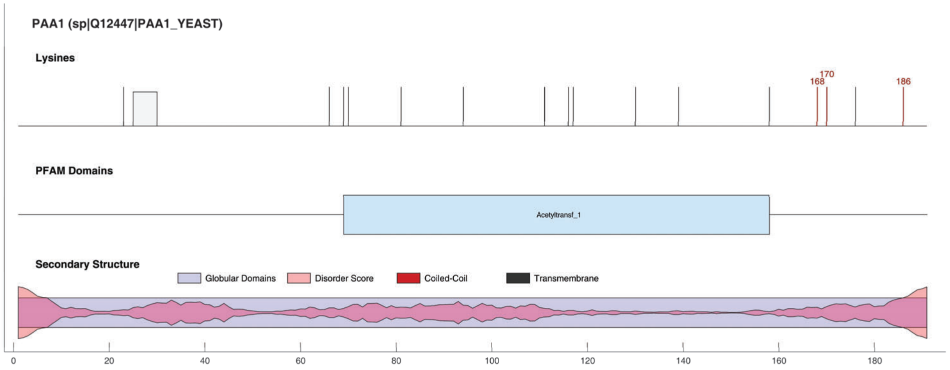
PAA1 (sp|Q12447|PAA1_YEAST)
Lysines
168
170
186
PFAM Domains
Acetyltransf_1
Secondary Structure
Globular Domains
Disorder Score
Coiled-Coil
Transmembrane
0
20
40
60
80
100
120
140
160
180

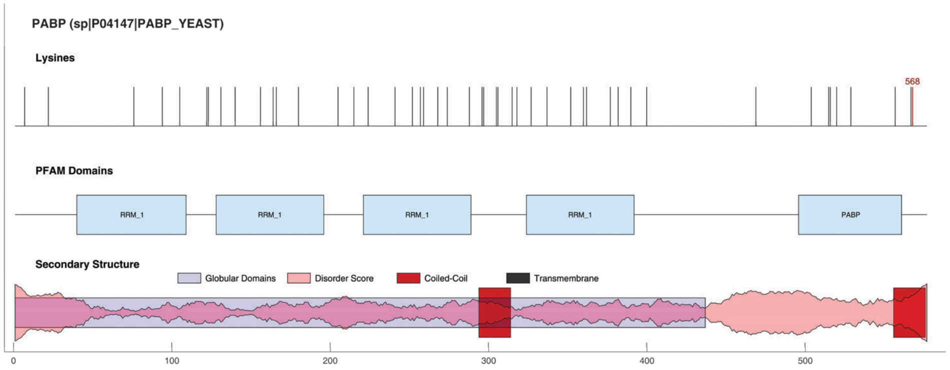
PABP (sp|P04147|PABP_YEAST)
Lysines
568
PFAM Domains
RRM_1
RRM_1
RRM_1
RRM_1
PABP
Secondary Structure
Globular Domains
Disorder Score
Coiled-Coil
Transmembrane
0
100
200
300
400
500

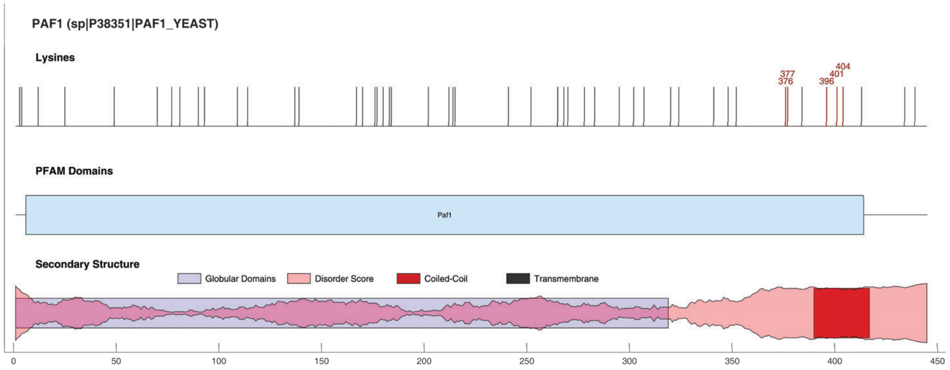
PAF1 (sp|P38351|PAF1_YEAST)
Lysines
377
376
396
401
404
PFAM Domains
Paf1
Secondary Structure
Globular Domains
Disorder Score
Coiled-Coil
Transmembrane
0
50
100
150
200
250
300
350
400
450

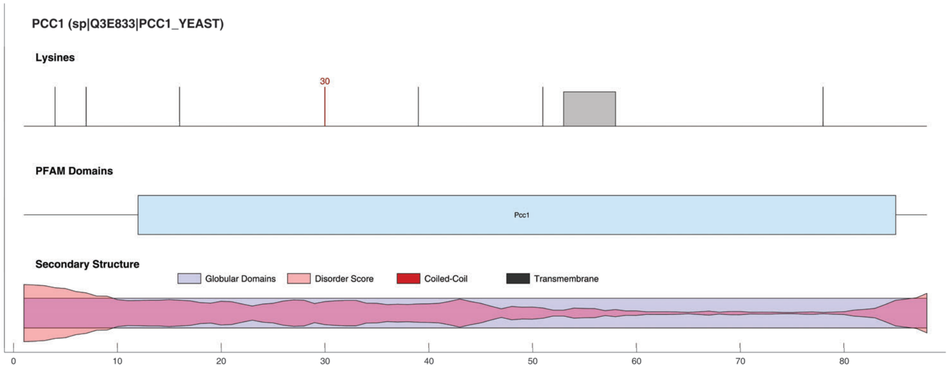
PCC1 (sp|Q3E833|PCC1_YEAST)
Lysines
30
PFAM Domains
Pcc1
Secondary Structure
Globular Domains
Disorder Score
Coiled-Coil
Transmembrane
0
10
20
30
40
50
60
70
80

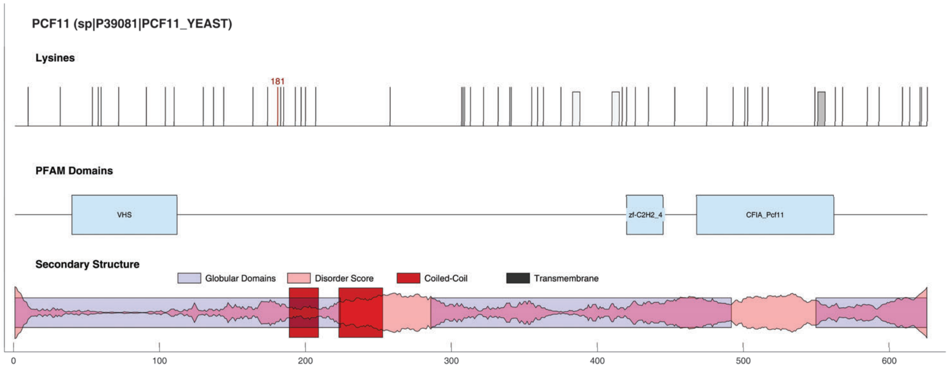
PCF11 (sp|P39081|PCF11_YEAST)
Lysines
181
PFAM Domains
VHS
zf-C2H2_4
CFIA_Pcf11
Secondary Structure
Globular Domains
Disorder Score
Coiled-Coil
Transmembrane
0
100
200
300
400
500
600

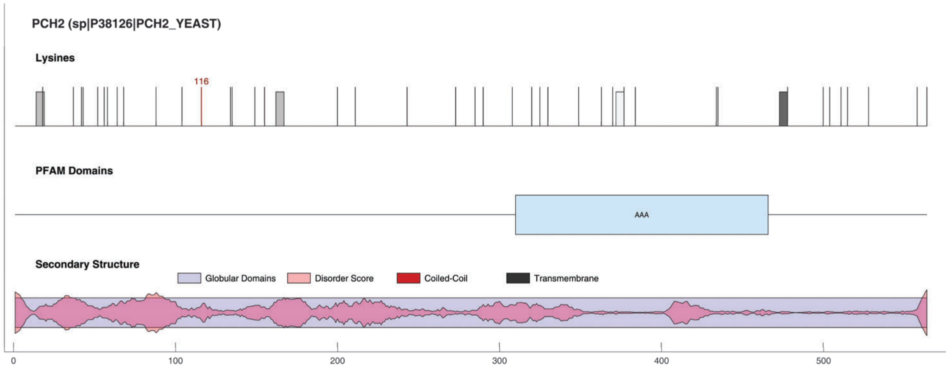
PCH2 (sp|P38126|PCH2_YEAST)
Lysines
116
PFAM Domains
AAA
Secondary Structure
Globular Domains
Disorder Score
Coiled-Coil
Transmembrane
0
100
200
300
400
500

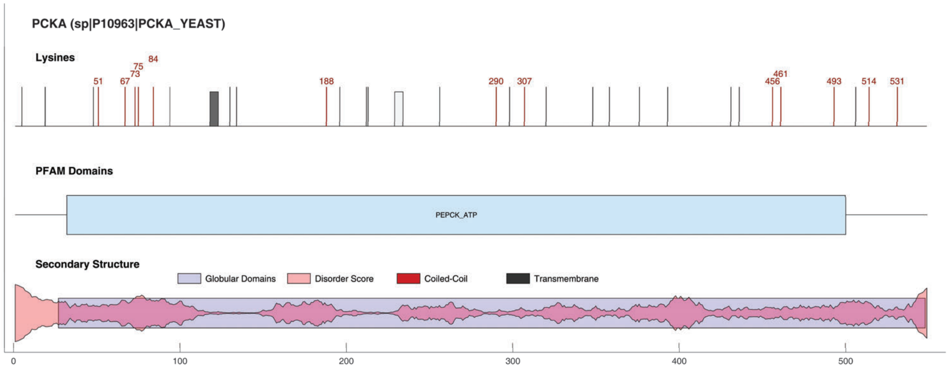
PCKA (sp|P10963|PCKA_YEAST)
Lysines
51
67
73
75
84
188
290
307
456
461
493
514
531
PFAM Domains
PEPCK_ATP
Secondary Structure
Globular Domains
Disorder Score
Coiled-Coil
Transmembrane
0
100
200
300
400
500

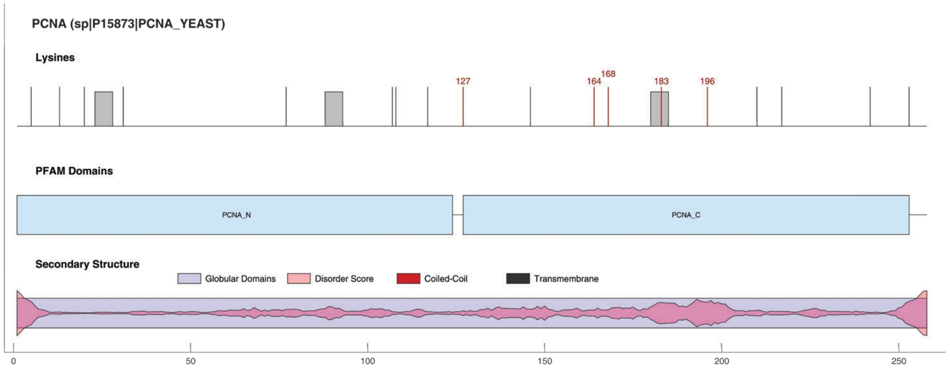
PCNA (sp|P15873|PCNA_YEAST)
Lysines
127
164
168
183
196
PFAM Domains
PCNA_N
PCNA_C
Secondary Structure
Globular Domains
Disorder Score
Coiled-Coil
Transmembrane
0
50
100
150
200
250

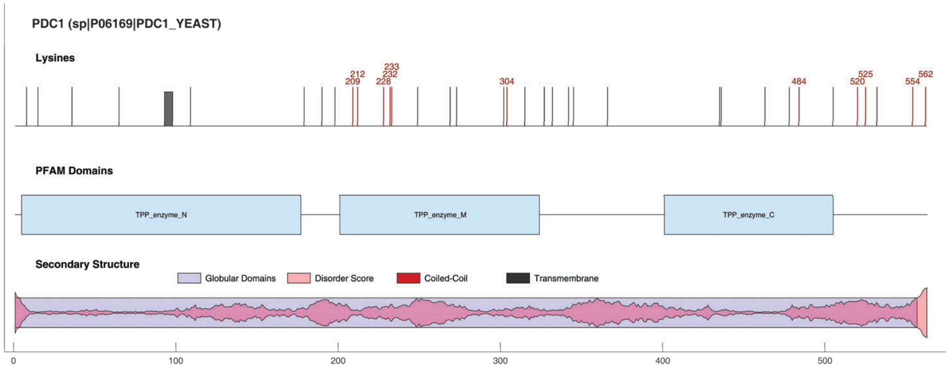

PDC1 (sp|P06169|PDC1_YEAST)
Lysines
209
212
228
232
233
304
484
520
525
554
562
PFAM Domains
TPP_enzyme_N
TPP_enzyme_M
TPP_enzyme_C
Secondary Structure
Globular Domains
Disorder Score
Coiled-Coil
Transmembrane
0
100
200
300
400
500

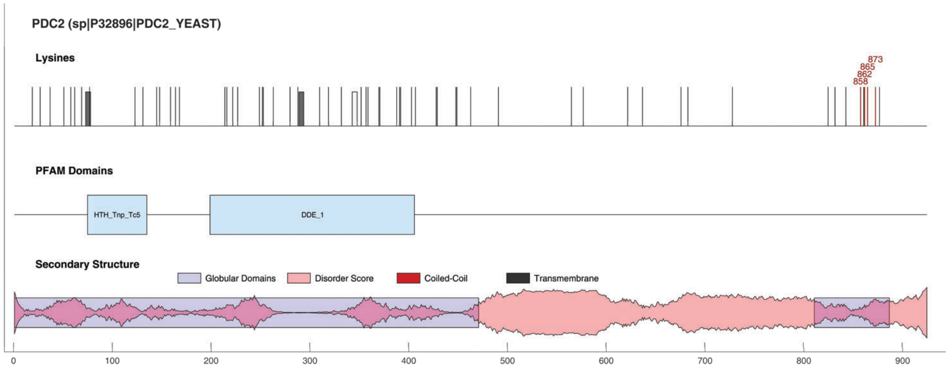

PDC2 (sp|P32896|PDC2_YEAST)
Lysines
858
862
865
873
PFAM Domains
HTH_Tnp_Tc5
DDE_1
Secondary Structure
Globular Domains
Disorder Score
Coiled-Coil
Transmembrane
0
100
200
300
400
500
600
700
800
900

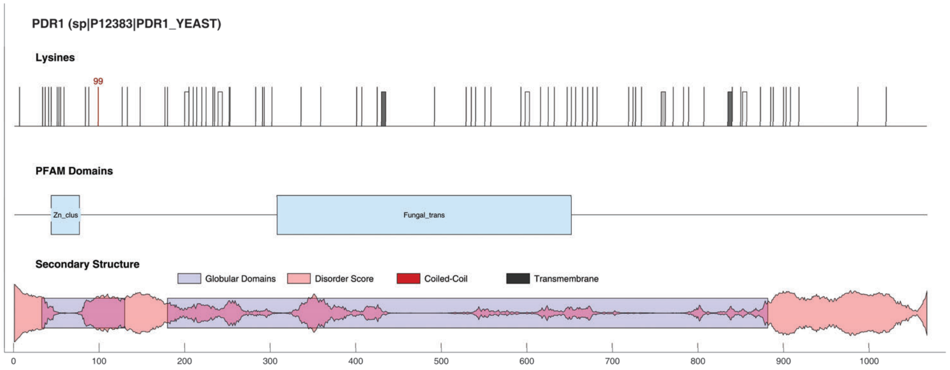

PDR1 (sp|P12383|PDR1_YEAST)
Lysines
99
PFAM Domains
Zn_clus
Fungal_trans
Secondary Structure
Globular Domains
Disorder Score
Coiled-Coil
Transmembrane
0
100
200
300
400
500
600
700
800
900
1000

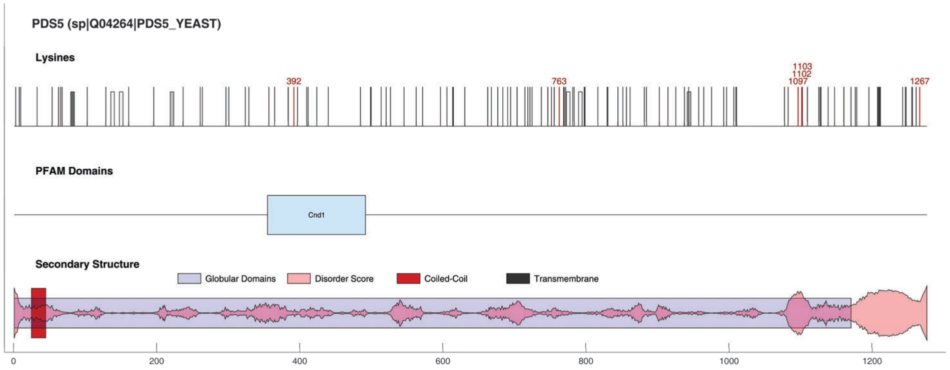

PDS5 (sp|Q04264|PDS5_YEAST)
Lysines
392
763
1097
1102
1103
1267
PFAM Domains
Cnd1
Secondary Structure
Globular Domains
Disorder Score
Coiled-Coil
Transmembrane
0
200
400
600
800
1000
1200

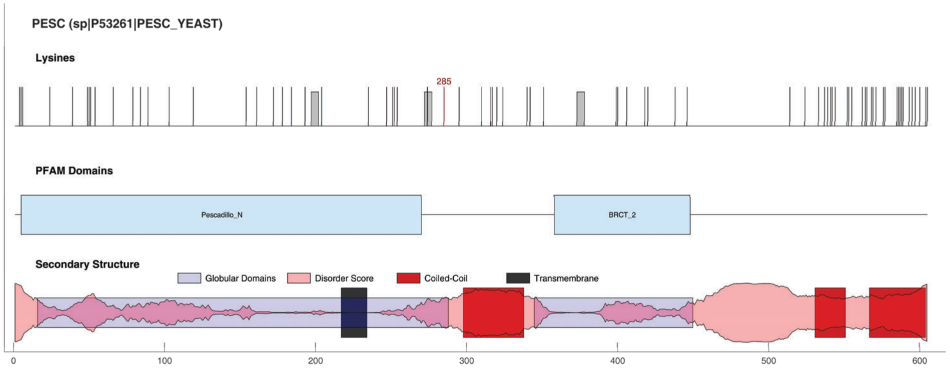
PESC (sp|P53261|PESC_YEAST)
Lysines
285
PFAM Domains
Pescadillo_N
BRCT_2
Secondary Structure
Globular Domains
Disorder Score
Coiled-Coil
Transmembrane
0
100
200
300
400
500
600

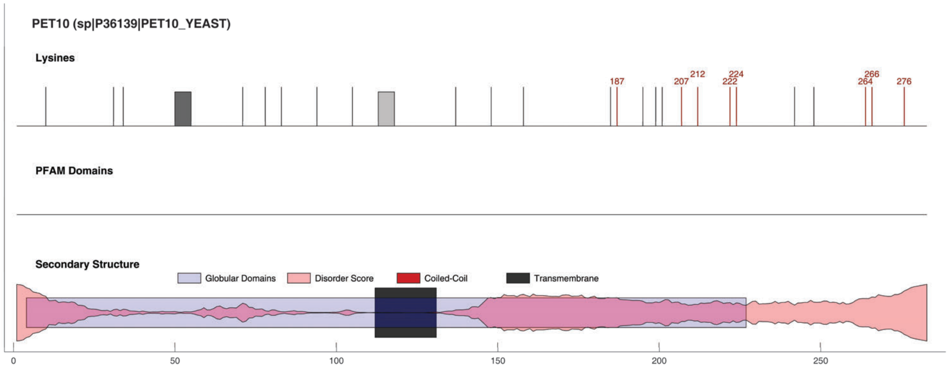
PET10 (sp|P36139|PET10_YEAST)
Lysines
187
207
212
222
224
264
266
276
PFAM Domains
Secondary Structure
Globular Domains
Disorder Score
Coiled-Coil
Transmembrane
0
50
100
150
200
250

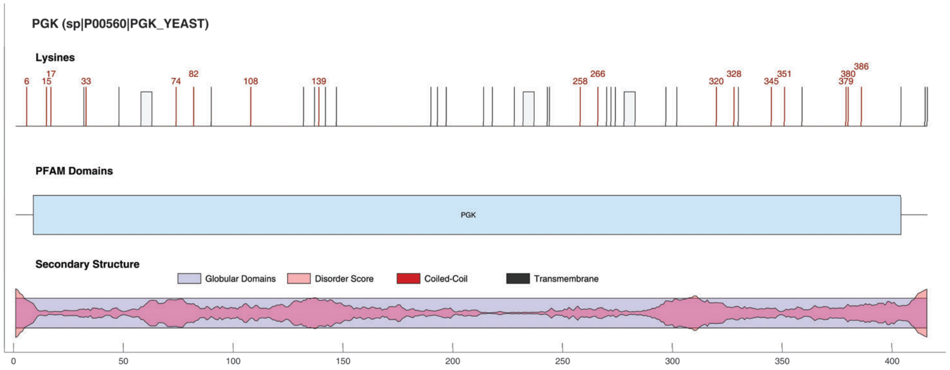
PGK (sp|P00560|PGK_YEAST)
Lysines
6
15
17
33
74
82
108
139
258
266
320
328
345
351
379
380
386
PFAM Domains
PGK
Secondary Structure
Globular Domains
Disorder Score
Coiled-Coil
Transmembrane
0
50
100
150
200
250
300
350
400

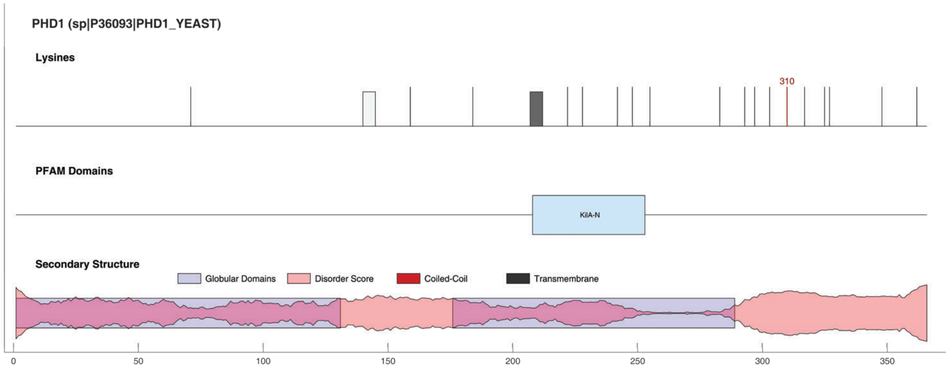
PHD1 (sp|P36093|PHD1_YEAST)
Lysines
310
PFAM Domains
KilA-N
Secondary Structure
Globular Domains
Disorder Score
Coiled-Coil
Transmembrane
0
50
100
150
200
250
300
350

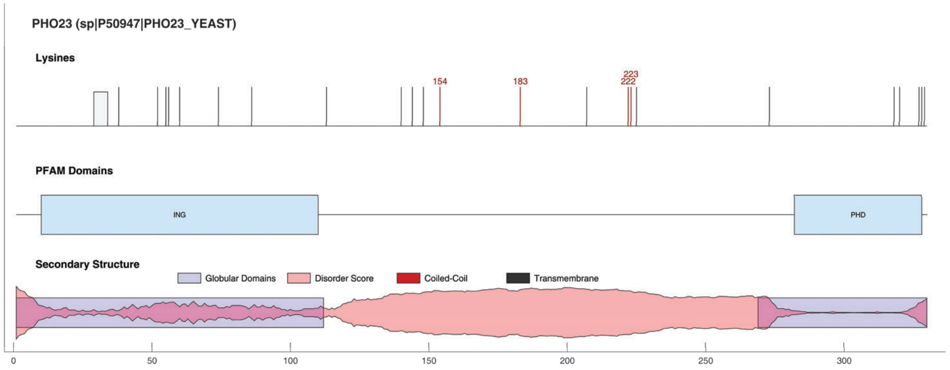
PHO23 (sp|P50947|PHO23_YEAST)
Lysines
154
183
223
222
PFAM Domains
ING
PHD
Secondary Structure
Globular Domains
Disorder Score
Coiled-Coil
Transmembrane
0
50
100
150
200
250
300

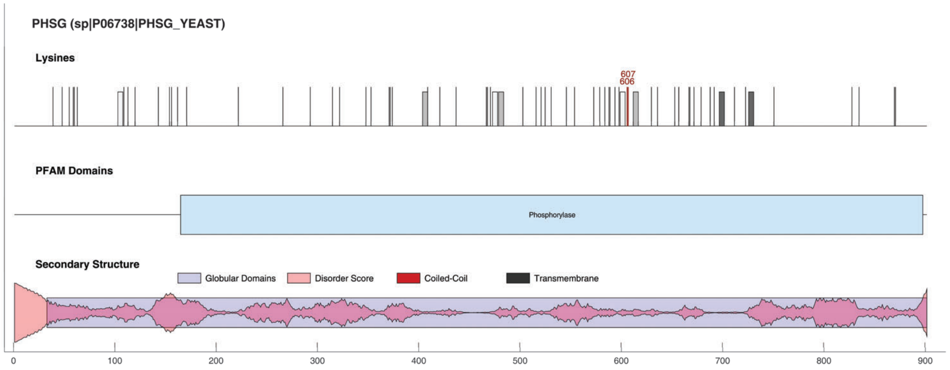
PHSG (sp|P06738|PHSG_YEAST)
Lysines
607
606
PFAM Domains
Phosphorylase
Secondary Structure
Globular Domains
Disorder Score
Coiled-Coil
Transmembrane
0
100
200
300
400
500
600
700
800
900

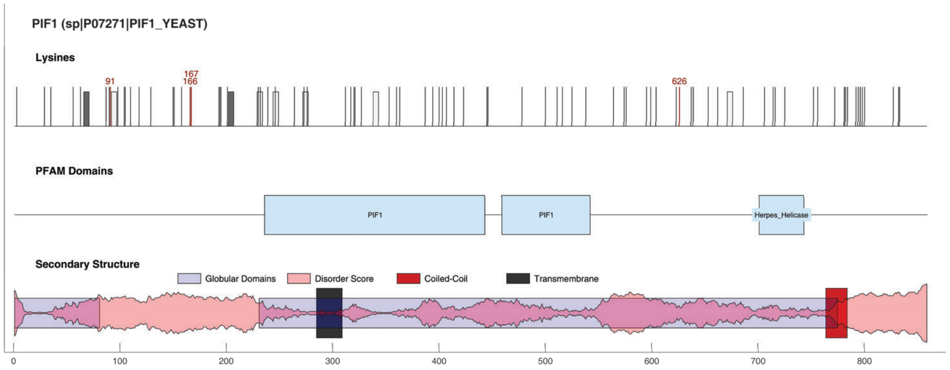
PIF1 (sp|P07271|PIF1_YEAST)
Lysines
91
167
166
626
PFAM Domains
PIF1
PIF1
Herpes_Helicase
Secondary Structure
Globular Domains
Disorder Score
Coiled-Coil
Transmembrane
0
100
200
300
400
500
600
700
800

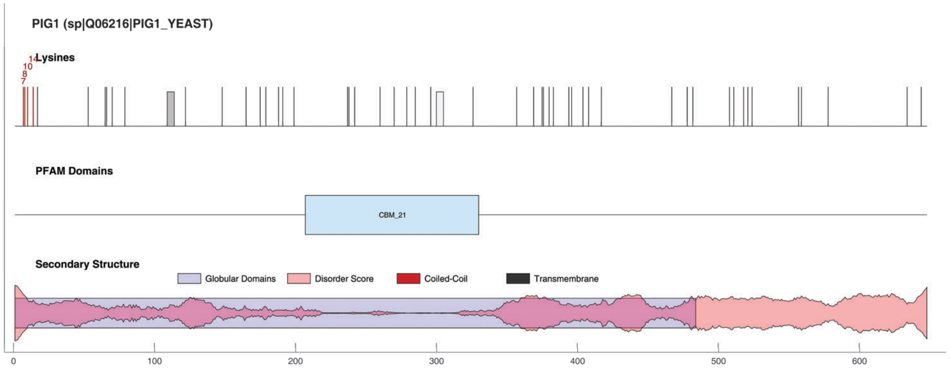
PIG1 (sp|Q06216|PIG1_YEAST)
Lysines
10
8
7
PFAM Domains
CBM_21
Secondary Structure
Globular Domains
Disorder Score
Coiled-Coil
Transmembrane
0
100
200
300
400
500
600

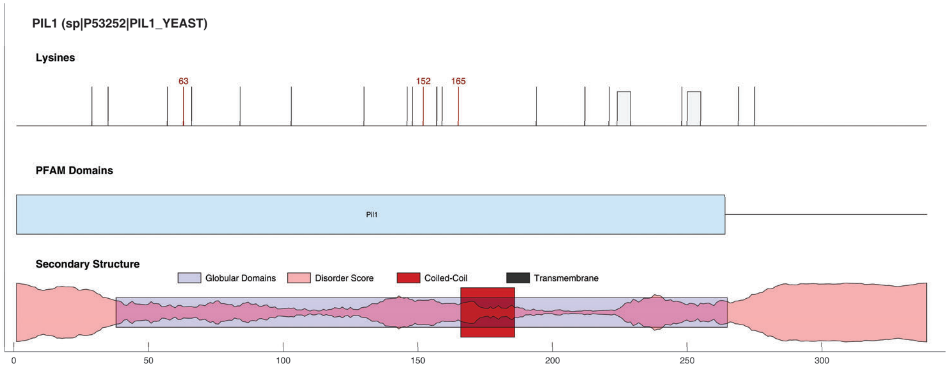
PIL1 (sp|P53252|PIL1_YEAST)
Lysines
63
152
165
PFAM Domains
Pil1
Secondary Structure
Globular Domains
Disorder Score
Coiled-Coil
Transmembrane
0
50
100
150
200
250
300

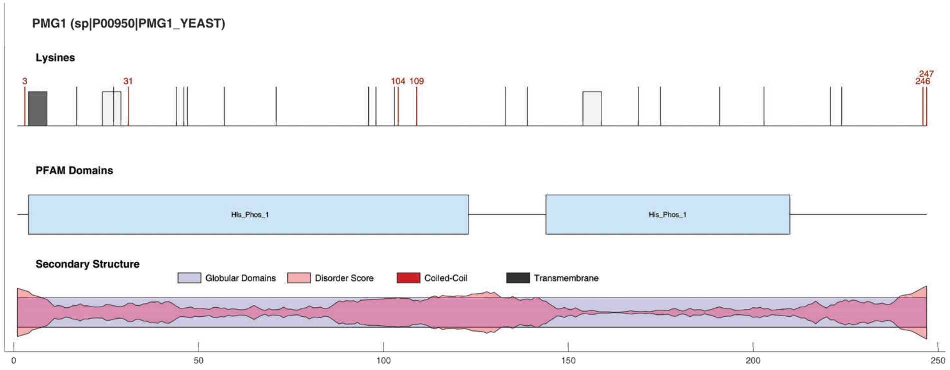
PMG1 (sp|P00950|PMG1_YEAST)
Lysines
3
31
104
109
247
246
PFAM Domains
His_Phos_1
His_Phos_1
Secondary Structure
Globular Domains
Disorder Score
Coiled-Coil
Transmembrane
0
50
100
150
200
250

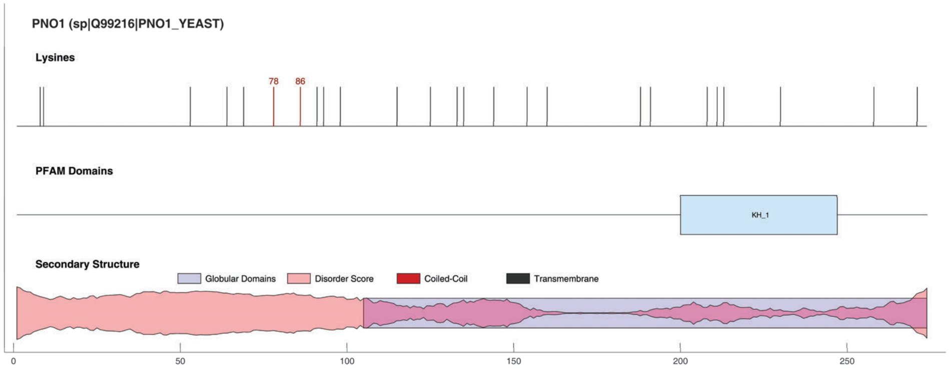
PNO1 (sp|Q99216|PNO1_YEAST)
Lysines
78
86
PFAM Domains
KH_1
Secondary Structure
Globular Domains
Disorder Score
Coiled-Coil
Transmembrane
0
50
100
150
200
250

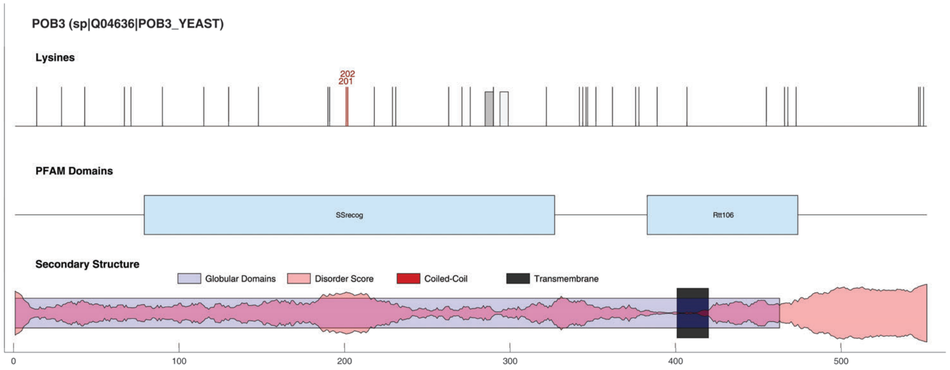
POB3 (sp|Q04636|POB3_YEAST)
Lysines
202
201
PFAM Domains
SSrecog
Rtt106
Secondary Structure
Globular Domains
Disorder Score
Coiled-Coil
Transmembrane
0
100
200
300
400
500

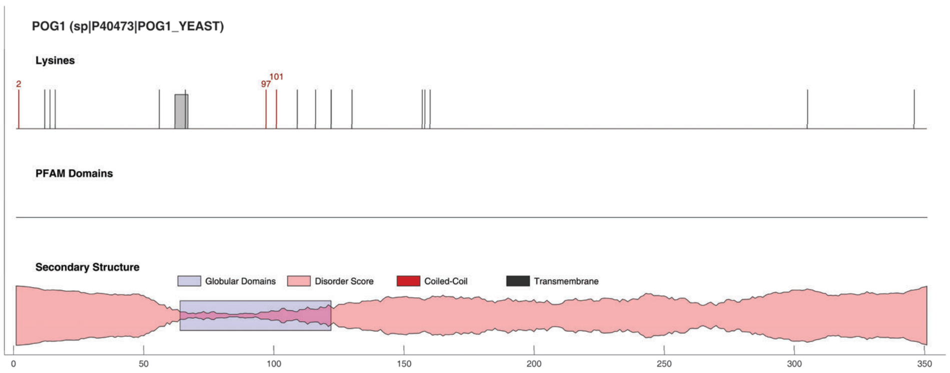
POG1 (sp|P40473|POG1_YEAST)
Lysines
2
97
101
PFAM Domains
Secondary Structure
Globular Domains
Disorder Score
Coiled-Coil
Transmembrane
0
50
100
150
200
250
300
350

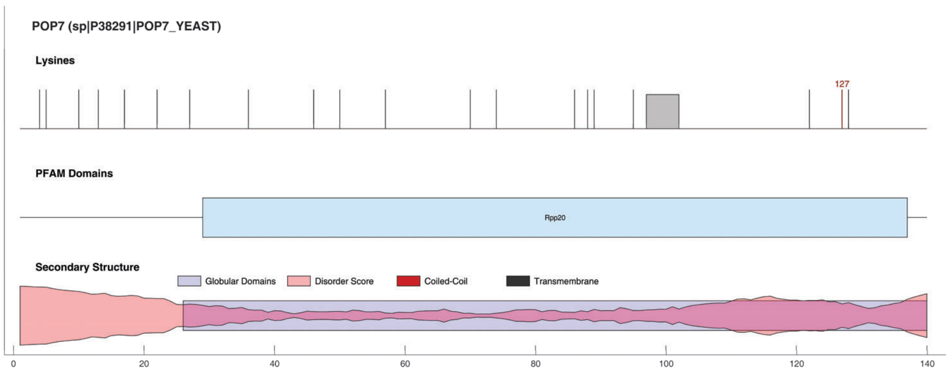
POP7 (sp|P38291|POP7_YEAST)
Lysines
127
PFAM Domains
Rpp20
Secondary Structure
Globular Domains
Disorder Score
Coiled-Coil
Transmembrane
0
20
40
60
80
100
120
140

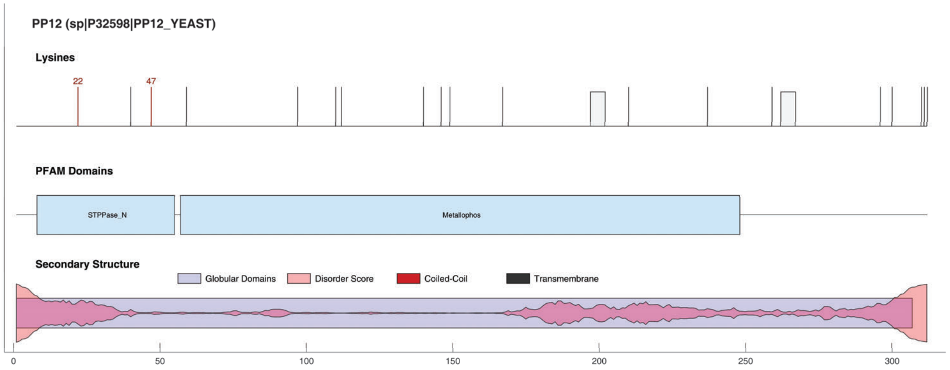
PP12 (sp|P32598|PP12_YEAST)
Lysines
22
47
PFAM Domains
STPPase_N
Metallophos
Secondary Structure
Globular Domains
Disorder Score
Coiled-Coil
Transmembrane
0
50
100
150
200
250
300

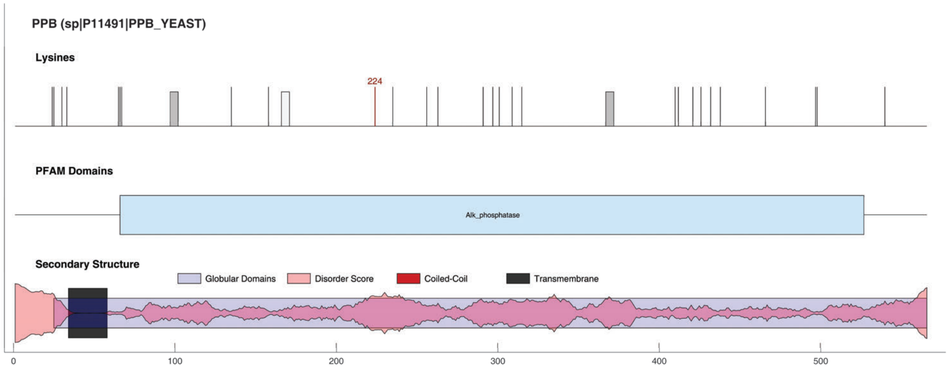
PPB (sp|P11491|PPB_YEAST)
Lysines
224
PFAM Domains
Alk_phosphatase
Secondary Structure
Globular Domains
Disorder Score
Coiled-Coil
Transmembrane
0
100
200
300
400
500

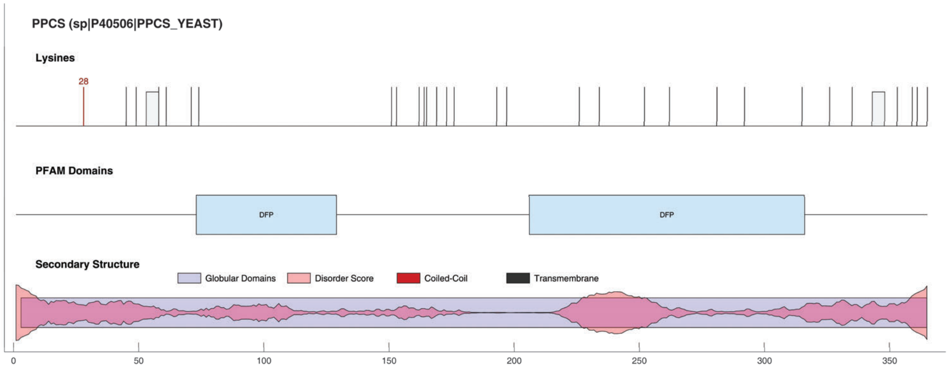
PPCS (sp|P40506|PPCS_YEAST)
Lysines
28
PFAM Domains
DFP
DFP
Secondary Structure
Globular Domains
Disorder Score
Coiled-Coil
Transmembrane
0
50
100
150
200
250
300
350

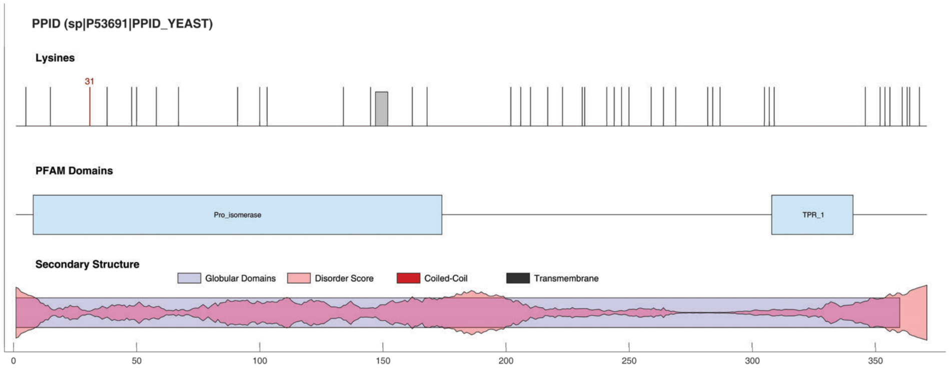
PPID (sp|P53691|PPID_YEAST)
Lysines
31
PFAM Domains
Pro_isomerase
TPR_1
Secondary Structure
Globular Domains
Disorder Score
Coiled-Coil
Transmembrane
0
50
100
150
200
250
300
350

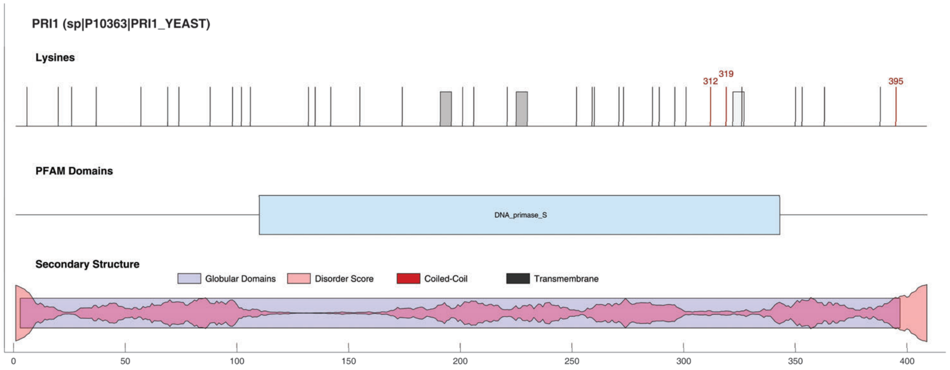
PRI1 (sp|P10363|PRI1_YEAST)
Lysines
312
319
395
PFAM Domains
DNA_primase_S
Secondary Structure
Globular Domains
Disorder Score
Coiled-Coil
Transmembrane
0
50
100
150
200
250
300
350
400

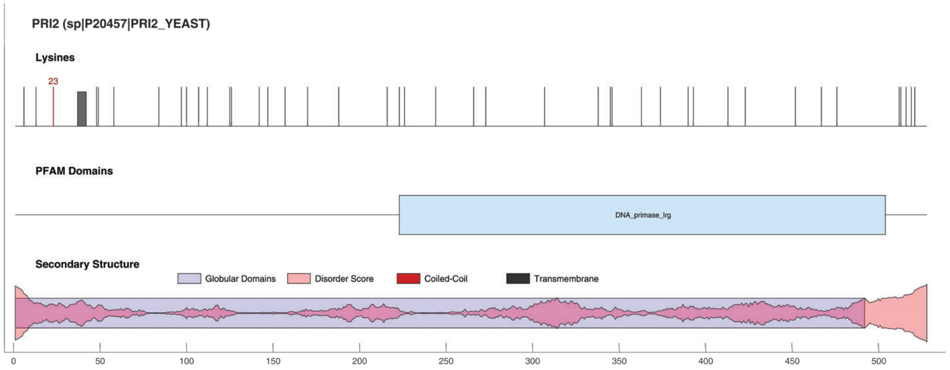
PRI2 (sp|P20457|PRI2_YEAST)
Lysines
23
PFAM Domains
DNA_primase_lrg
Secondary Structure
Globular Domains
Disorder Score
Coiled-Coil
Transmembrane
0
50
100
150
200
250
300
350
400
450
500

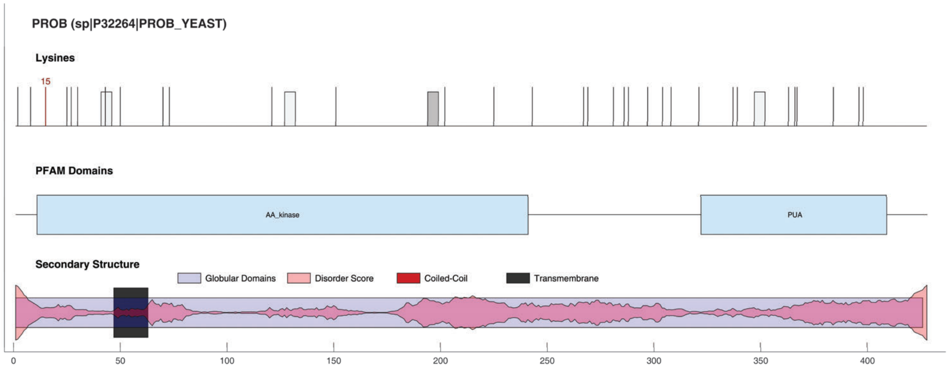
PROB (sp|P32264|PROB_YEAST)
Lysines
15
PFAM Domains
AA_kinase
PUA
Secondary Structure
Globular Domains
Disorder Score
Coiled-Coil
Transmembrane

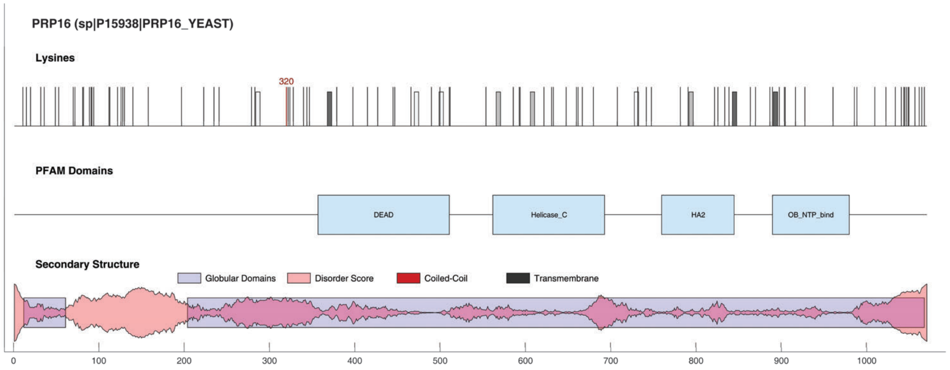
PRP16 (sp|P15938|PRP16_YEAST)
Lysines
320
PFAM Domains
DEAD
Helicase_C
HA2
OB_NTP_bind
Secondary Structure
Globular Domains
Disorder Score
Coiled-Coil
Transmembrane

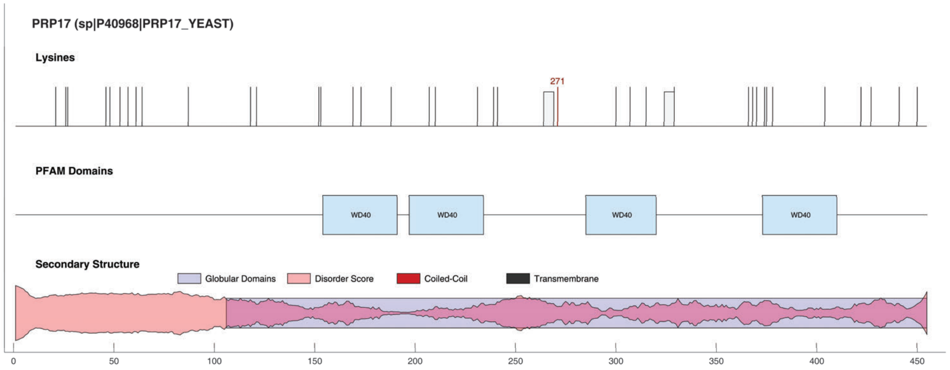
PRP17 (sp|P40968|PRP17_YEAST)
Lysines
271
PFAM Domains
WD40
WD40
WD40
WD40
Secondary Structure
Globular Domains
Disorder Score
Coiled-Coil
Transmembrane

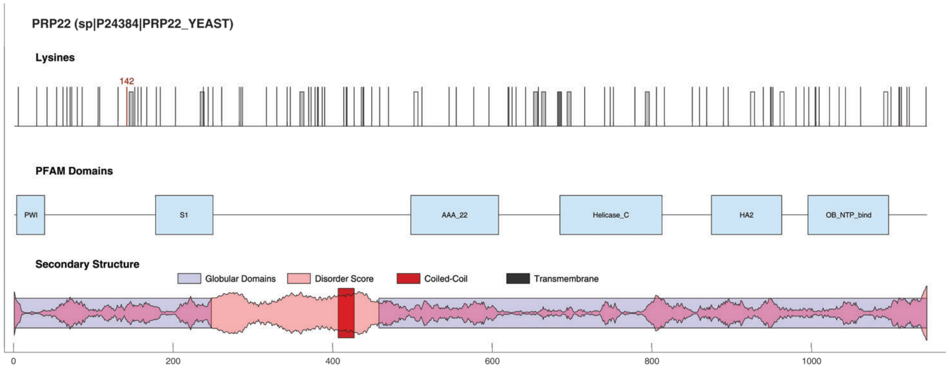
PRP22 (sp|P24384|PRP22_YEAST)
Lysines
142
PFAM Domains
PWI
S1
AAA_22
Helicase_C
HA2
OB_NTP_bind
Secondary Structure
Globular Domains
Disorder Score
Coiled-Coil
Transmembrane

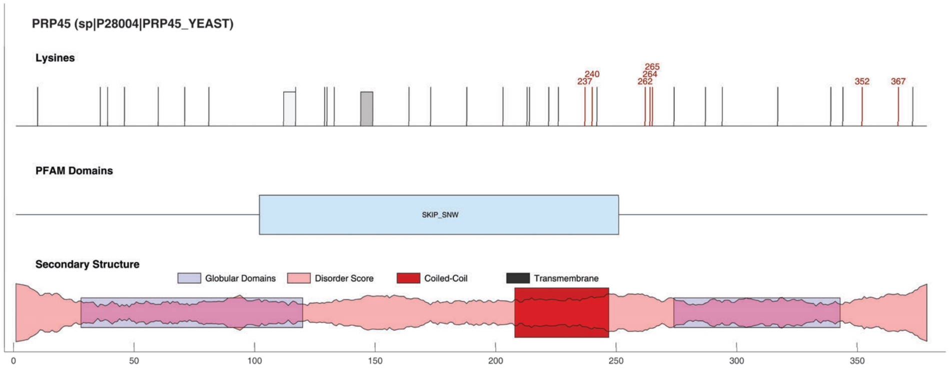
PRP45 (sp|P28004|PRP45_YEAST)
Lysines
240
237
265
264
262
352
367
PFAM Domains
SKIP_SNW
Secondary Structure
Globular Domains
Disorder Score
Coiled-Coil
Transmembrane
0
50
100
150
200
250
300
350

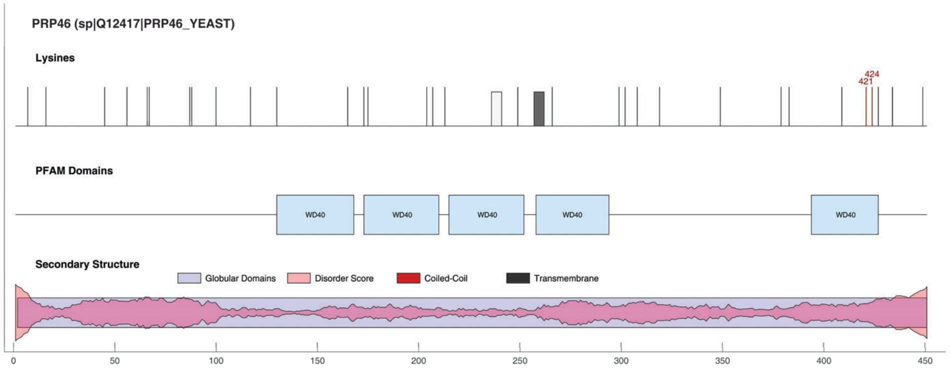
PRP46 (sp|Q12417|PRP46_YEAST)
Lysines
424
421
PFAM Domains
WD40
WD40
WD40
WD40
WD40
Secondary Structure
Globular Domains
Disorder Score
Coiled-Coil
Transmembrane
0
50
100
150
200
250
300
350
400
450

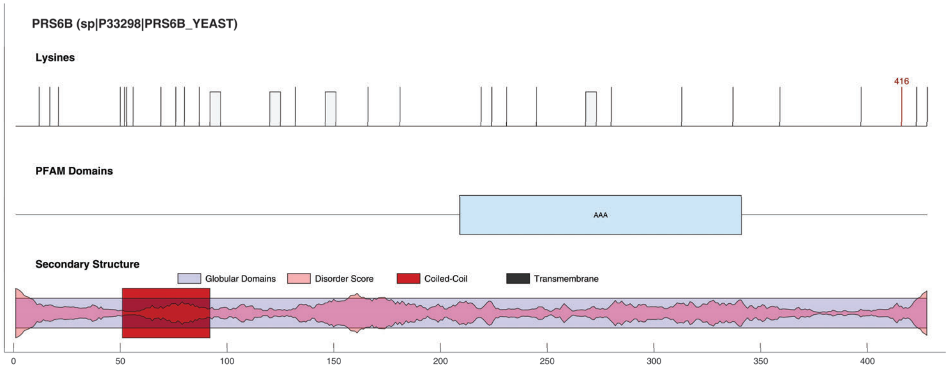
PRS6B (sp|P33298|PRS6B_YEAST)
Lysines
416
PFAM Domains
AAA
Secondary Structure
Globular Domains
Disorder Score
Coiled-Coil
Transmembrane
0
50
100
150
200
250
300
350
400

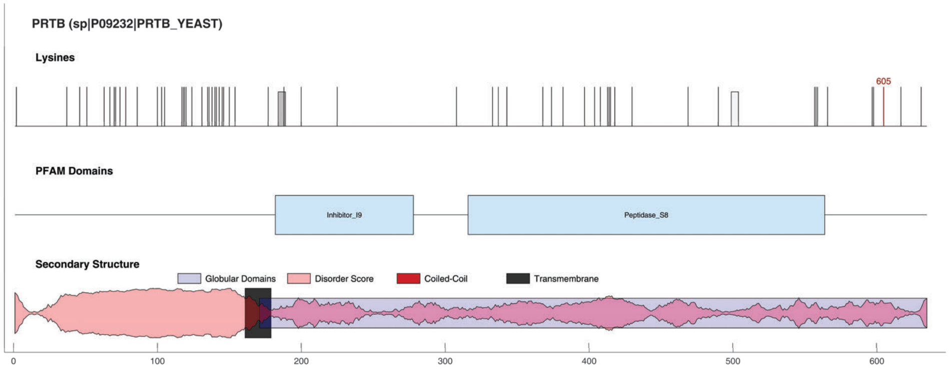
PRTB (sp|P09232|PRTB_YEAST)
Lysines
605
PFAM Domains
Inhibitor_I9
Peptidase_S8
Secondary Structure
Globular Domains
Disorder Score
Coiled-Coil
Transmembrane
0
100
200
300
400
500
600

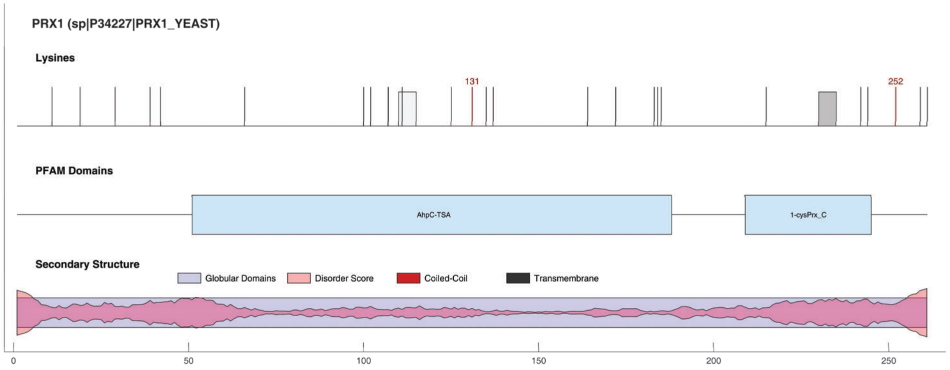
PRX1 (sp|P34227|PRX1_YEAST)
Lysines
131
252
PFAM Domains
AhpC-TSA
1-cysPrx_C
Secondary Structure
Globular Domains
Disorder Score
Coiled-Coil
Transmembrane
0
50
100
150
200
250

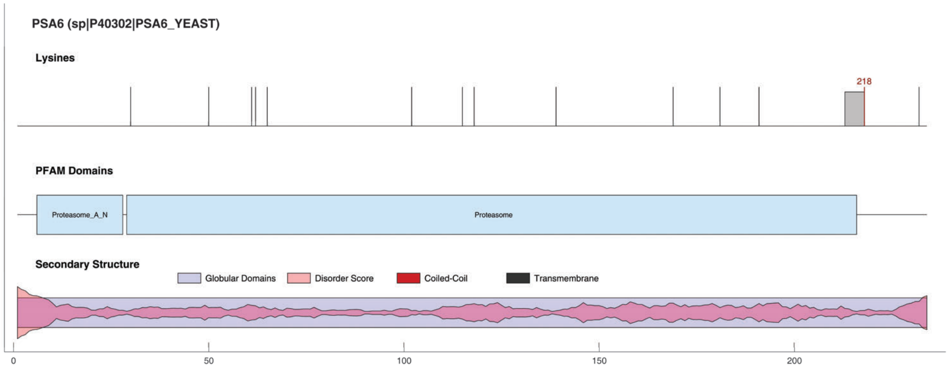
PSA6 (sp|P40302|PSA6_YEAST)
Lysines
218
PFAM Domains
Proteasome_A_N
Proteasome
Secondary Structure
Globular Domains
Disorder Score
Coiled-Coil
Transmembrane
0
50
100
150
200

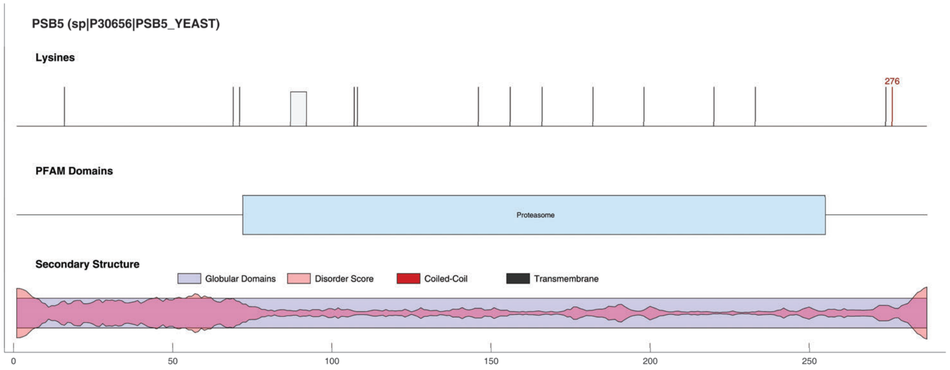
PSB5 (sp|P30656|PSB5_YEAST)
Lysines
276
PFAM Domains
Proteasome
Secondary Structure
Globular Domains
Disorder Score
Coiled-Coil
Transmembrane
0
50
100
150
200
250

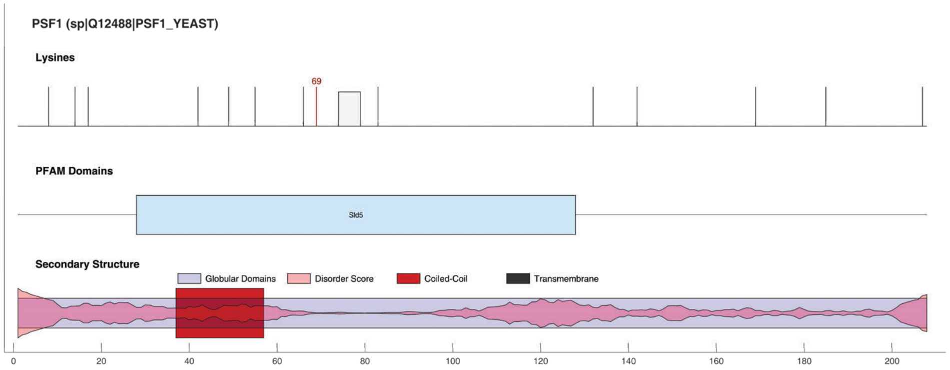
PSF1 (sp|Q12488|PSF1_YEAST)
Lysines
69
PFAM Domains
Sld5
Secondary Structure
Globular Domains
Disorder Score
Coiled-Coil
Transmembrane
0
20
40
60
80
100
120
140
160
180
200

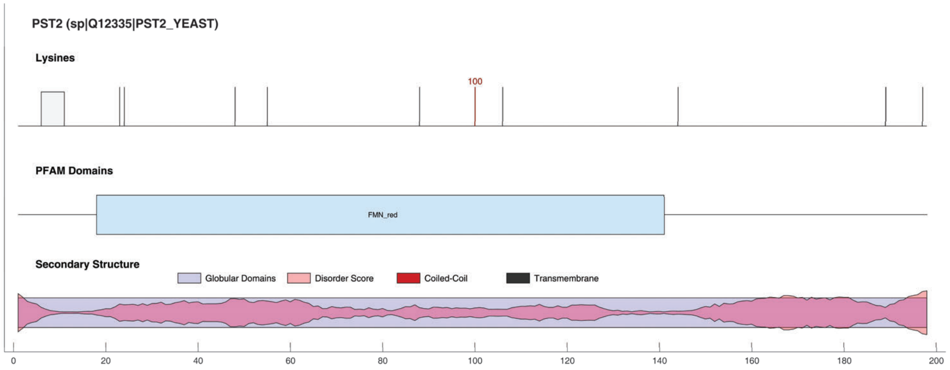
PST2 (sp|Q12335|PST2_YEAST)
Lysines
100
PFAM Domains
FMN_red
Secondary Structure
Globular Domains
Disorder Score
Coiled-Coil
Transmembrane
0
20
40
60
80
100
120
140
160
180
200

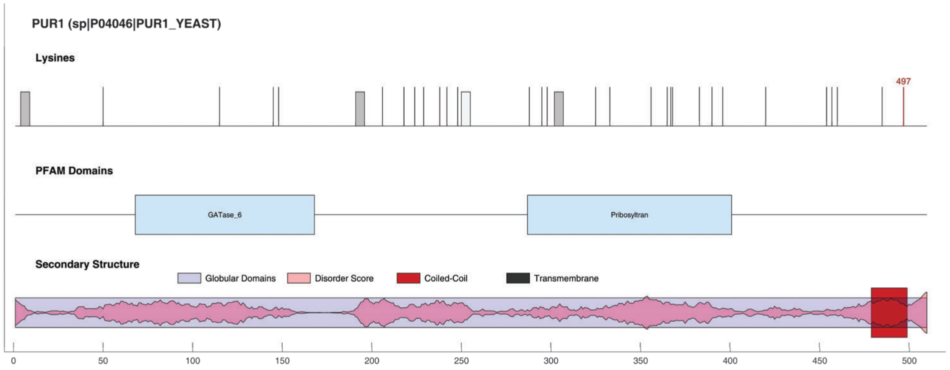
PUR1 (sp|P04046|PUR1_YEAST)
Lysines
497
PFAM Domains
GATase_6
Pribosyltran
Secondary Structure
Globular Domains
Disorder Score
Coiled-Coil
Transmembrane
0
50
100
150
200
250
300
350
400
450
500

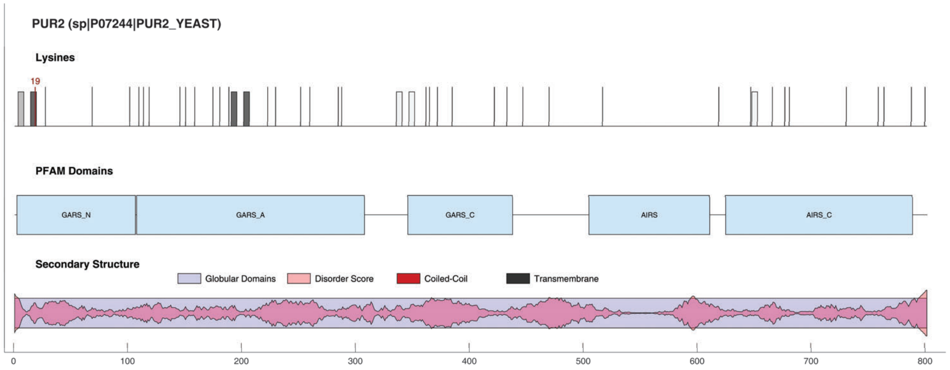
PUR2 (sp|P07244|PUR2_YEAST)
Lysines
19
PFAM Domains
GARS_N
GARS_A
GARS_C
AIRS
AIRS_C
Secondary Structure
Globular Domains
Disorder Score
Coiled-Coil
Transmembrane
0
100
200
300
400
500
600
700
800

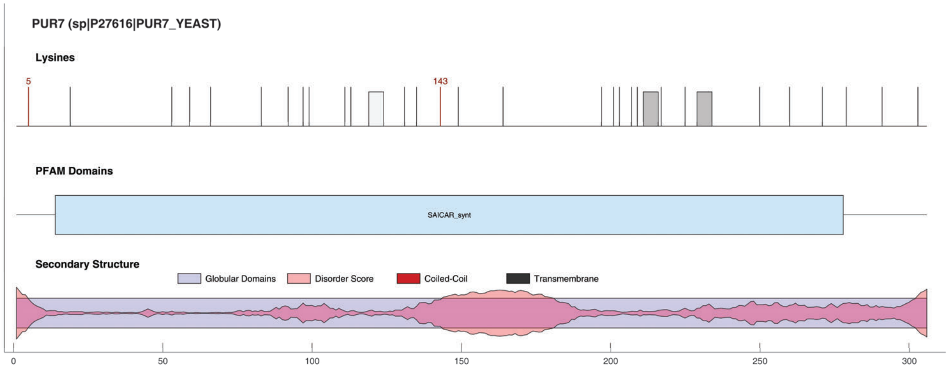
PUR7 (sp|P27616|PUR7_YEAST)
Lysines
5
143
PFAM Domains
SAICAR_synt
Secondary Structure
Globular Domains
Disorder Score
Coiled-Coil
Transmembrane
0
50
100
150
200
250
300

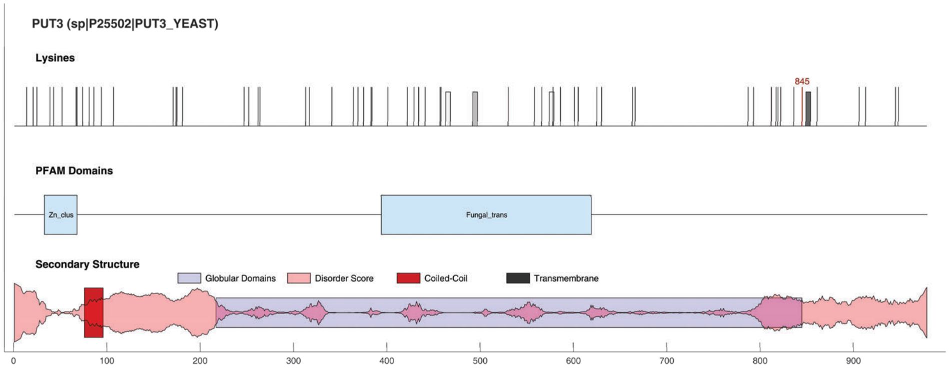
PUT3 (sp|P25502|PUT3_YEAST)
Lysines
845
PFAM Domains
Zn_clus
Fungal_trans
Secondary Structure
Globular Domains
Disorder Score
Coiled-Coil
Transmembrane
0
100
200
300
400
500
600
700
800
900

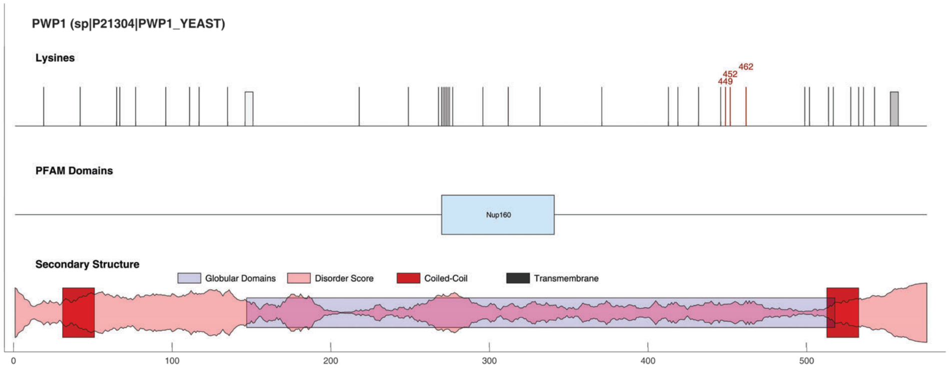
PWP1 (sp|P21304|PWP1_YEAST)
Lysines
449
452
462
PFAM Domains
Nup160
Secondary Structure
Globular Domains
Disorder Score
Coiled-Coil
Transmembrane
0
100
200
300
400
500

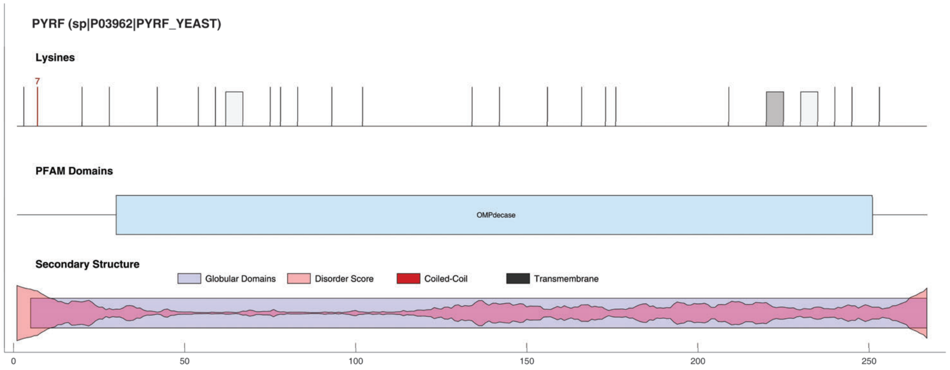
PYRF (sp|P03962|PYRF_YEAST)
Lysines
7
PFAM Domains
OMPdecase
Secondary Structure
Globular Domains
Disorder Score
Coiled-Coil
Transmembrane
0
50
100
150
200
250

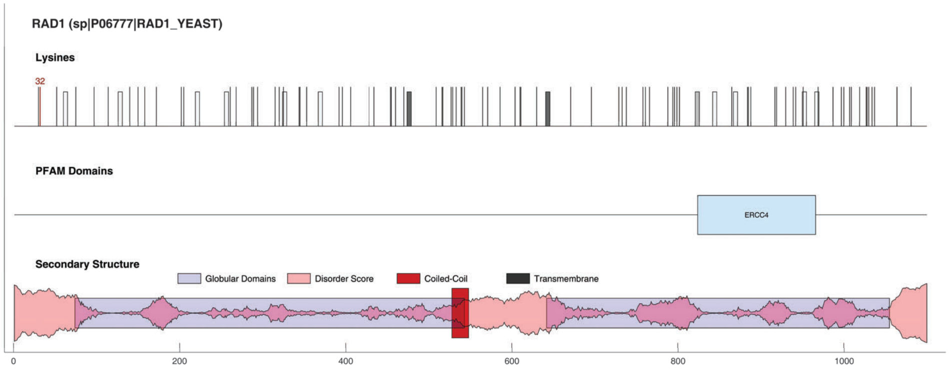
RAD1 (sp|P06777|RAD1_YEAST)
Lysines
32
PFAM Domains
ERCC4
Secondary Structure
Globular Domains
Disorder Score
Coiled-Coil
Transmembrane
0
200
400
600
800
1000

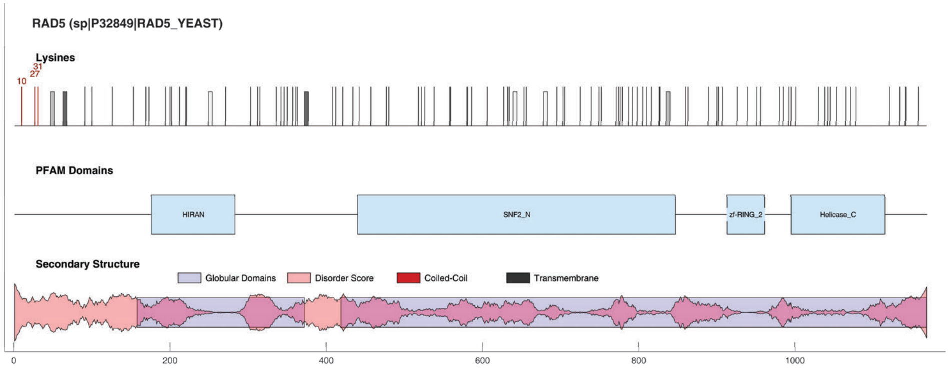
RAD5 (sp|P32849|RAD5_YEAST)
Lysines
10
27
31
PFAM Domains
HIRAN
SNF2_N
zf-RING_2
Helicase_C
Secondary Structure
Globular Domains
Disorder Score
Coiled-Coil
Transmembrane
0
200
400
600
800
1000

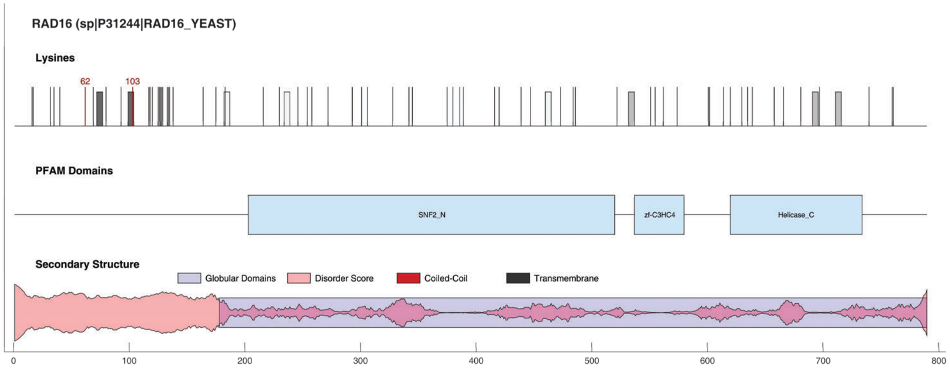
RAD16 (sp|P31244|RAD16_YEAST)
Lysines
62
103
PFAM Domains
SNF2_N
zf-C3HC4
Helicase_C
Secondary Structure
Globular Domains
Disorder Score
Coiled-Coil
Transmembrane
0
100
200
300
400
500
600
700
800

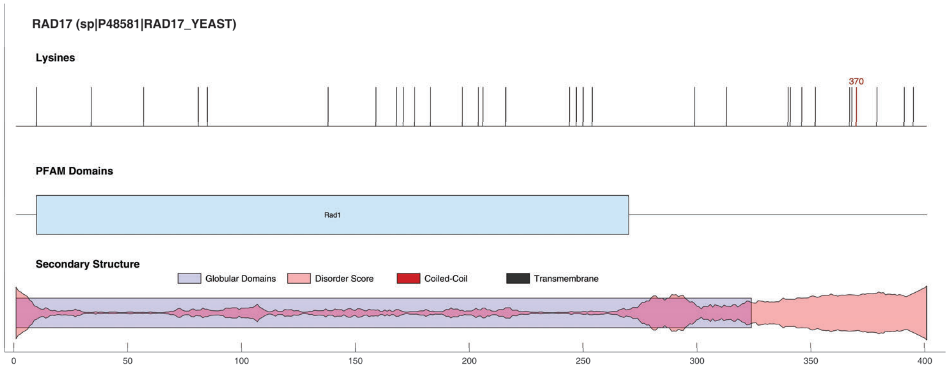
RAD17 (sp|P48581|RAD17_YEAST)
Lysines
370
PFAM Domains
Rad1
Secondary Structure
Globular Domains
Disorder Score
Coiled-Coil
Transmembrane
0
50
100
150
200
250
300
350
400

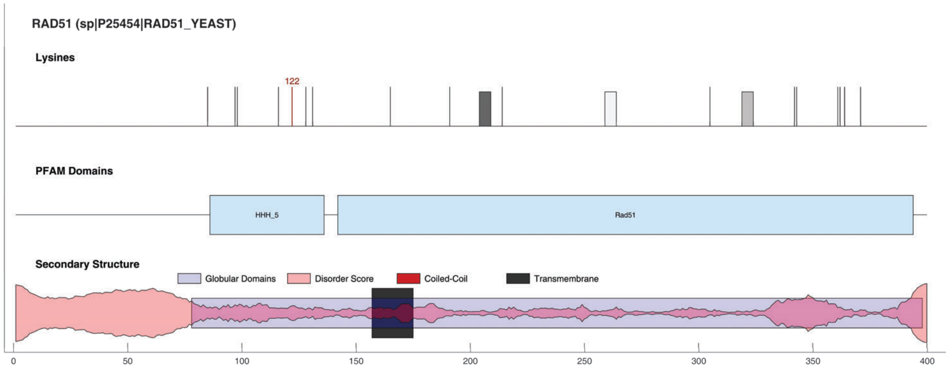
RAD51 (sp|P25454|RAD51_YEAST)
Lysines
122
PFAM Domains
HHH_5
Rad51
Secondary Structure
Globular Domains
Disorder Score
Coiled-Coil
Transmembrane
0
50
100
150
200
250
300
350
400

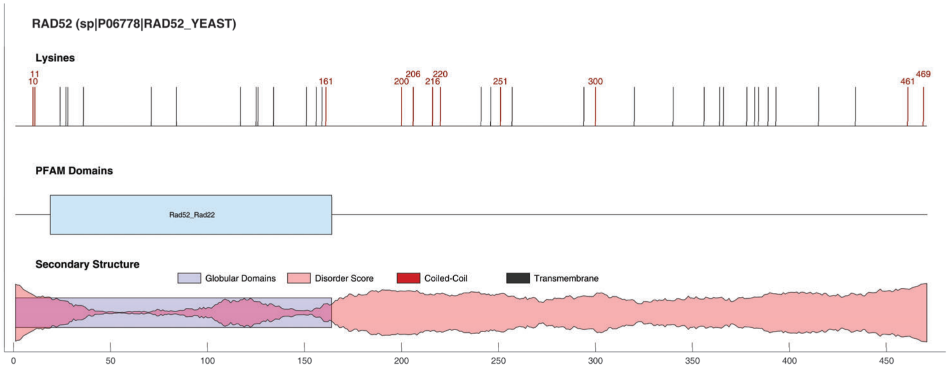
RAD52 (sp|P06778|RAD52_YEAST)
Lysines
11
10
161
200
206
216
220
251
300
461
469
PFAM Domains
Rad52_Rad22
Secondary Structure
Globular Domains
Disorder Score
Coiled-Coil
Transmembrane
0
50
100
150
200
250
300
350
400
450

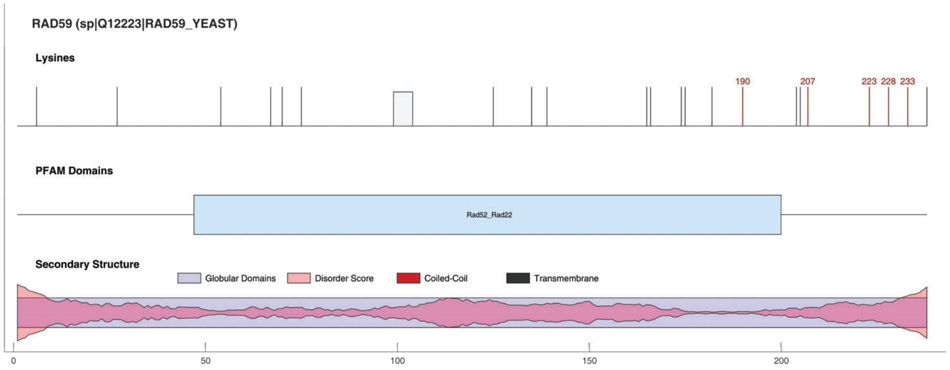
RAD59 (sp|Q12223|RAD59_YEAST)
Lysines
190
207
223
228
233
PFAM Domains
Rad52_Rad22
Secondary Structure
Globular Domains
Disorder Score
Coiled-Coil
Transmembrane
0
50
100
150
200

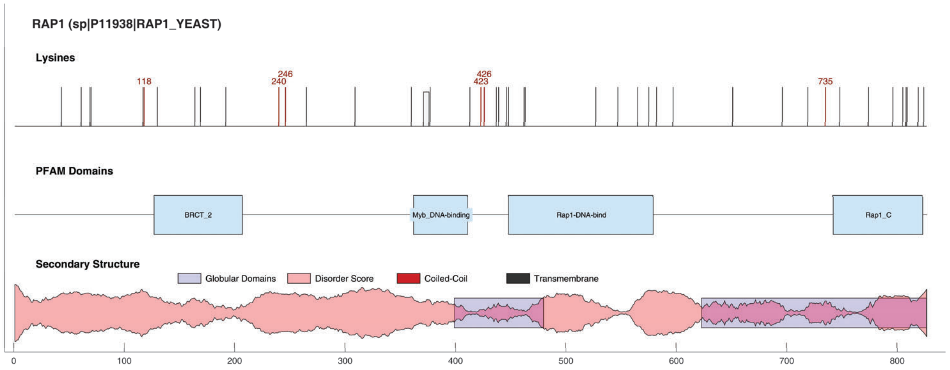
RAP1 (sp|P11938|RAP1_YEAST)
Lysines
118
240
246
423
426
735
PFAM Domains
BRCT_2
Myb_DNA-binding
Rap1-DNA-bind
Rap1_C
Secondary Structure
Globular Domains
Disorder Score
Coiled-Coil
Transmembrane
0
100
200
300
400
500
600
700
800

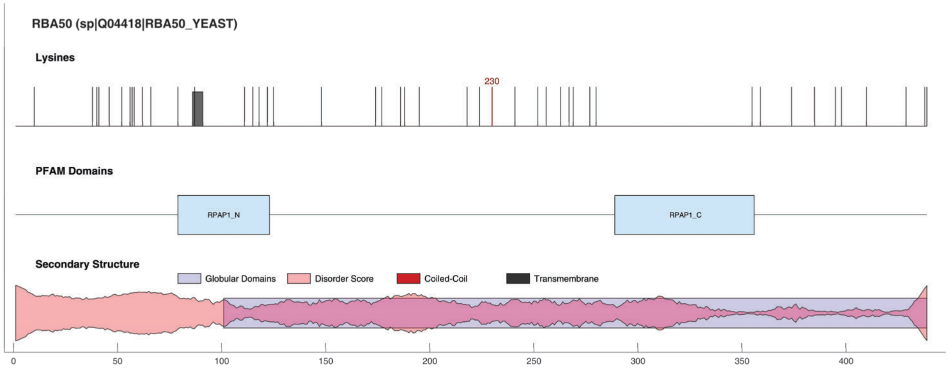
RBA50 (sp|Q04418|RBA50_YEAST)
Lysines
230
PFAM Domains
RPAP1_N
RPAP1_C
Secondary Structure
Globular Domains
Disorder Score
Coiled-Coil
Transmembrane
0
50
100
150
200
250
300
350
400

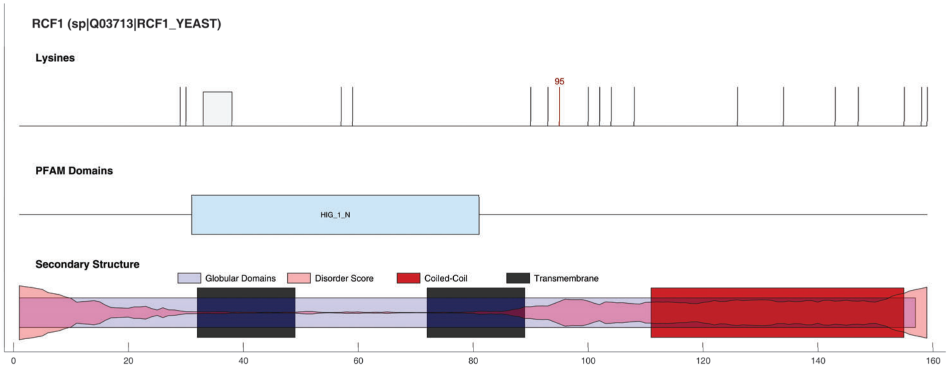
RCF1 (sp|Q03713|RCF1_YEAST)
Lysines
95
PFAM Domains
HIG_1_N
Secondary Structure
Globular Domains
Disorder Score
Coiled-Coil
Transmembrane
0
20
40
60
80
100
120
140
160

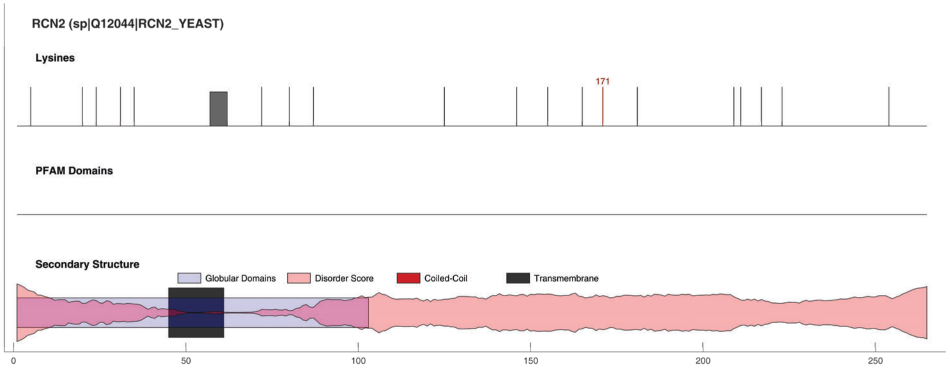
RCN2 (sp|Q12044|RCN2_YEAST)
Lysines
171
PFAM Domains
Secondary Structure
Globular Domains
Disorder Score
Coiled-Coil
Transmembrane
0
50
100
150
200
250

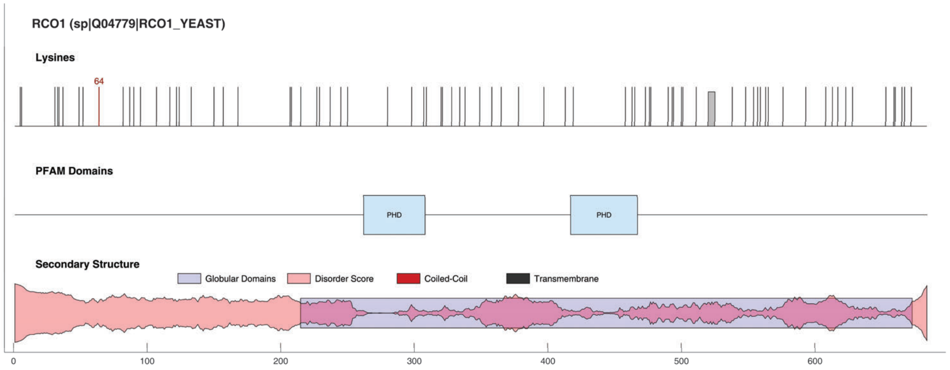
RCO1 (sp|Q04779|RCO1_YEAST)
Lysines
64
PFAM Domains
PHD
PHD
Secondary Structure
Globular Domains
Disorder Score
Coiled-Coil
Transmembrane
0
100
200
300
400
500
600

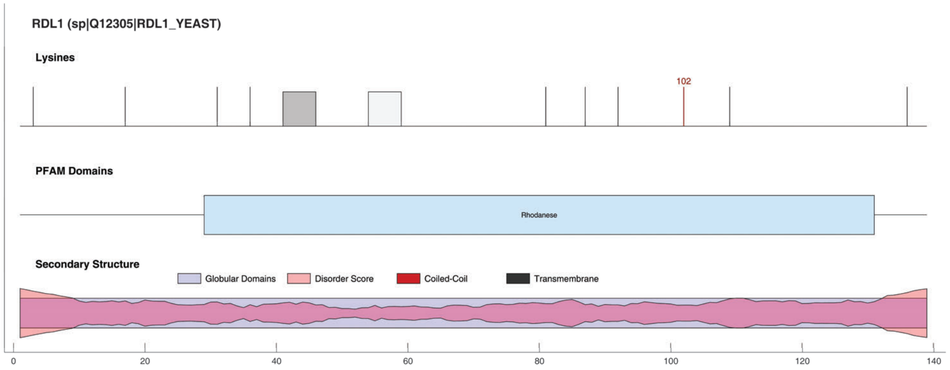
RDL1 (sp|Q12305|RDL1_YEAST)
Lysines
102
PFAM Domains
Rhodanese
Secondary Structure
Globular Domains
Disorder Score
Coiled-Coil
Transmembrane
0
20
40
60
80
100
120
140

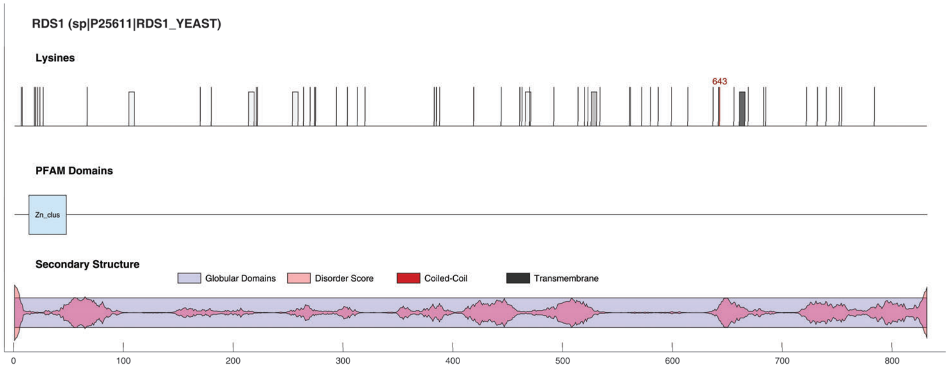
RDS1 (sp|P25611|RDS1_YEAST)
Lysines
643
PFAM Domains
Zn_clus
Secondary Structure
Globular Domains
Disorder Score
Coiled-Coil
Transmembrane
0
100
200
300
400
500
600
700
800

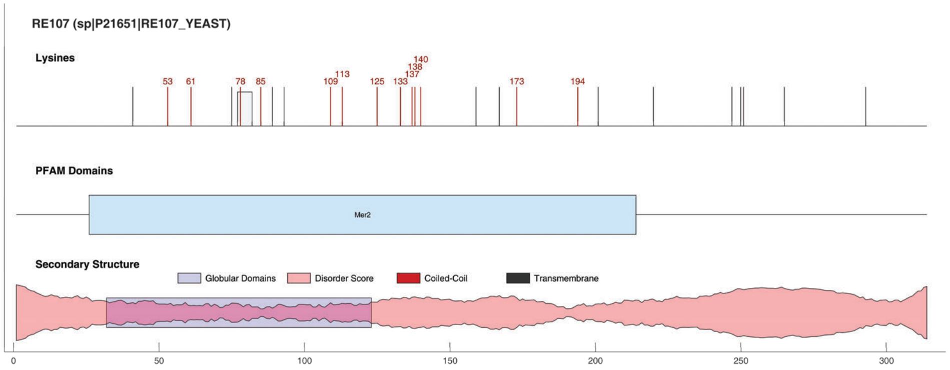
RE107 (sp|P21651|RE107_YEAST)
Lysines
53
61
78
85
109
113
125
133
137
138
140
173
194
PFAM Domains
Mer2
Secondary Structure
Globular Domains
Disorder Score
Coiled-Coil
Transmembrane
0
50
100
150
200
250
300

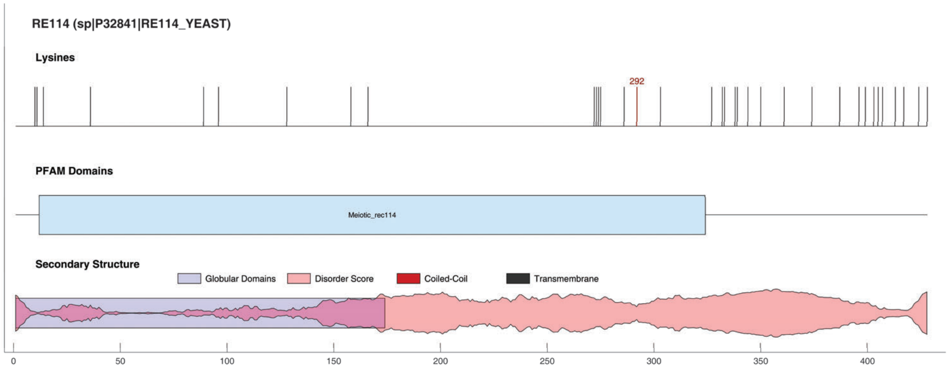
RE114 (sp|P32841|RE114_YEAST)
Lysines
292
PFAM Domains
Meiotic_rec114
Secondary Structure
Globular Domains
Disorder Score
Coiled-Coil
Transmembrane
0
50
100
150
200
250
300
350
400

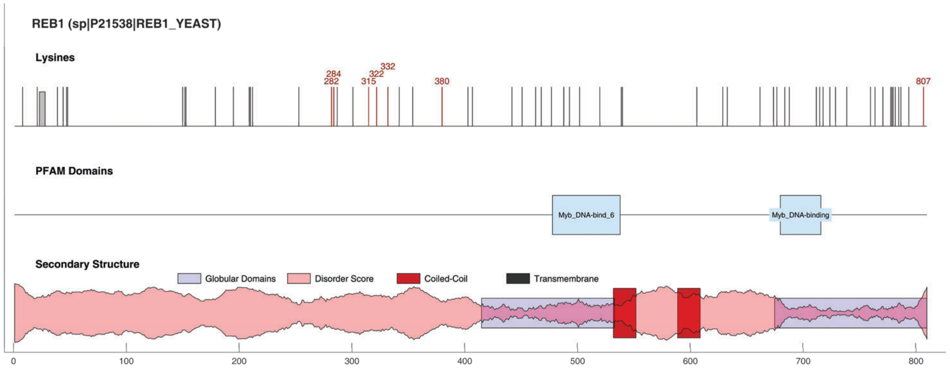
REB1 (sp|P21538|REB1_YEAST)
Lysines
282
284
315
322
332
380
807
PFAM Domains
Myb_DNA-bind_6
Myb_DNA-binding
Secondary Structure
Globular Domains
Disorder Score
Coiled-Coil
Transmembrane
0
100
200
300
400
500
600
700
800

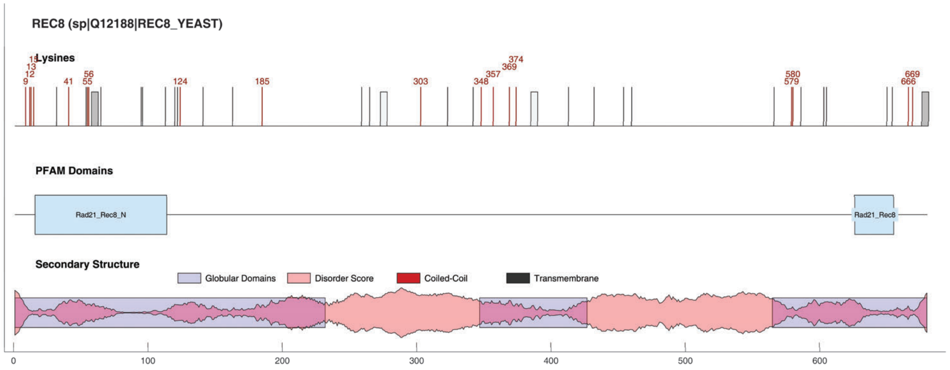
REC8 (sp|Q12188|REC8_YEAST)
Lysines
PFAM Domains
Rad21_Rec8_N
Rad21_Rec8
Secondary Structure
Globular Domains
Disorder Score
Coiled-Coil
Transmembrane

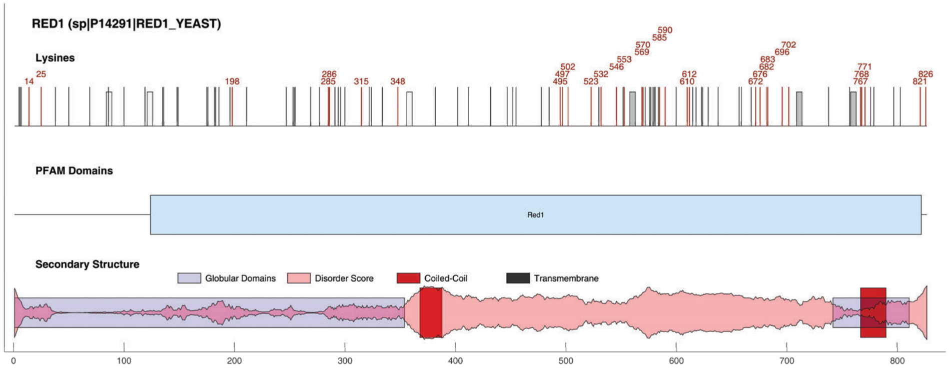
RED1 (sp|P14291|RED1_YEAST)
Lysines
PFAM Domains
Red1
Secondary Structure
Globular Domains
Disorder Score
Coiled-Coil
Transmembrane

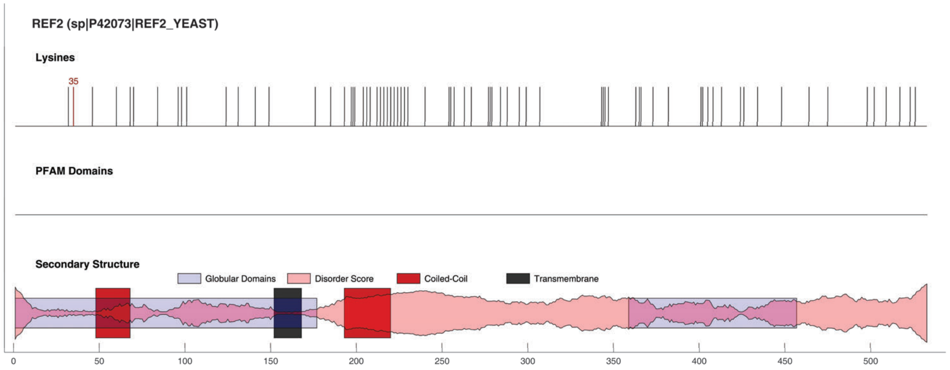
REF2 (sp|P42073|REF2_YEAST)
Lysines
PFAM Domains
Secondary Structure
Globular Domains
Disorder Score
Coiled-Coil
Transmembrane

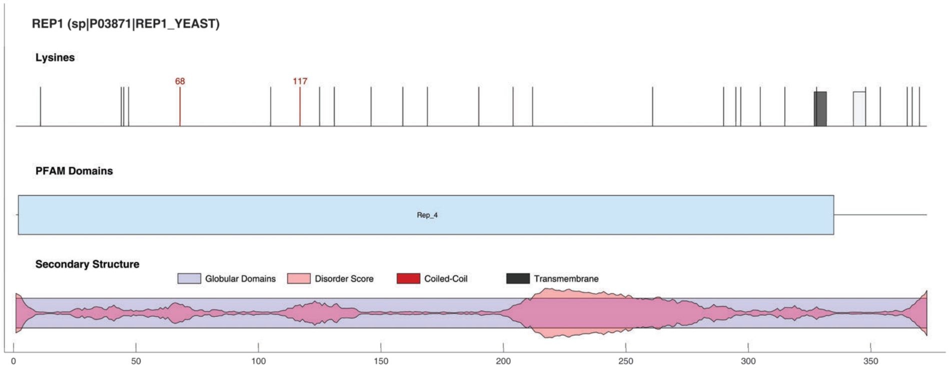
REP1 (sp|P03871|REP1_YEAST)
Lysines
PFAM Domains
Rep_4
Secondary Structure
Globular Domains
Disorder Score
Coiled-Coil
Transmembrane

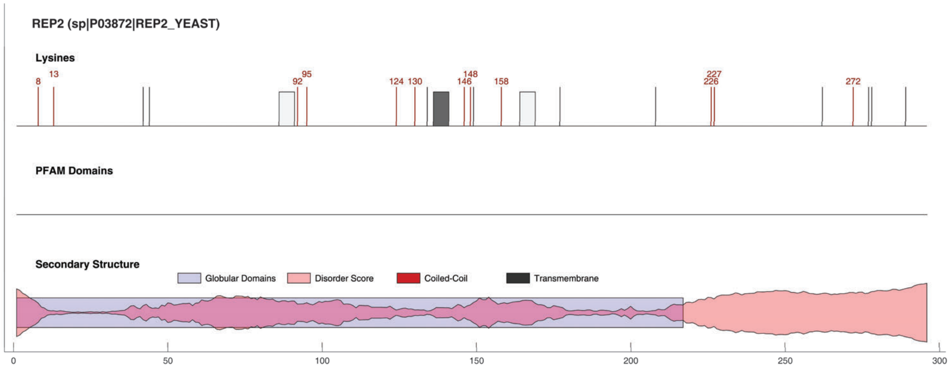
REP2 (sp|P03872|REP2_YEAST)
Lysines
8
13
92
95
124
130
146
148
158
226
227
272
PFAM Domains
Secondary Structure
Globular Domains
Disorder Score
Coiled-Coil
Transmembrane
0
50
100
150
200
250
300

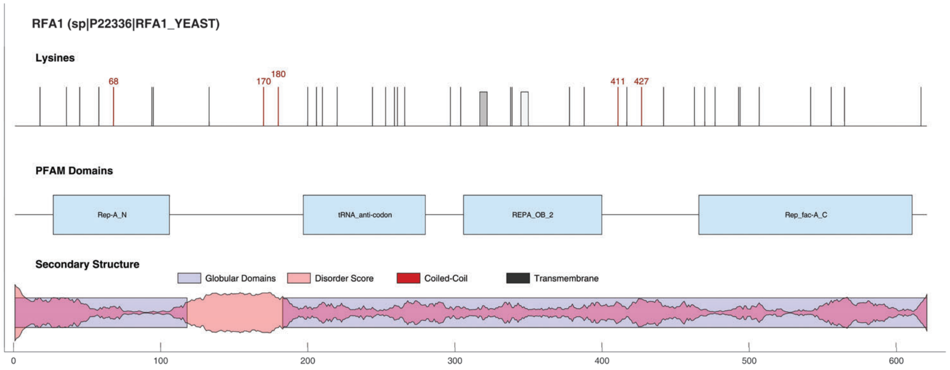
RFA1 (sp|P22336|RFA1_YEAST)
Lysines
68
170
180
411
427
PFAM Domains
Rep-A_N
tRNA_anti-codon
REPA_OB_2
Rep_fac-A_C
Secondary Structure
Globular Domains
Disorder Score
Coiled-Coil
Transmembrane
0
100
200
300
400
500
600

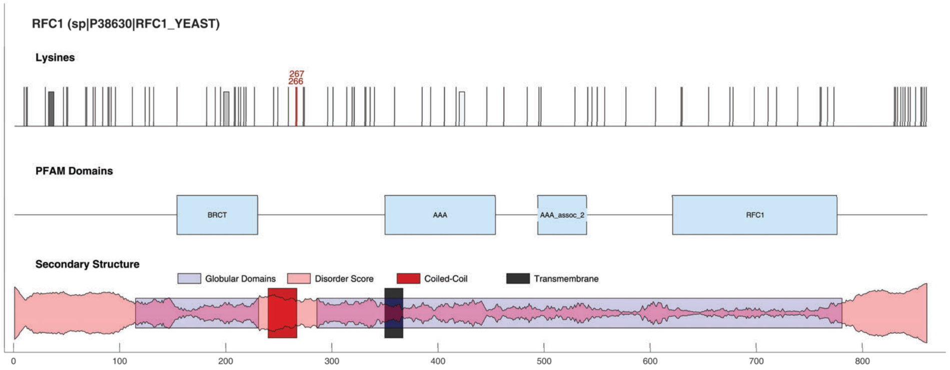
RFC1 (sp|P38630|RFC1_YEAST)
Lysines
266
267
PFAM Domains
BRCT
AAA
AAA_assoc_2
RFC1
Secondary Structure
Globular Domains
Disorder Score
Coiled-Coil
Transmembrane
0
100
200
300
400
500
600
700
800

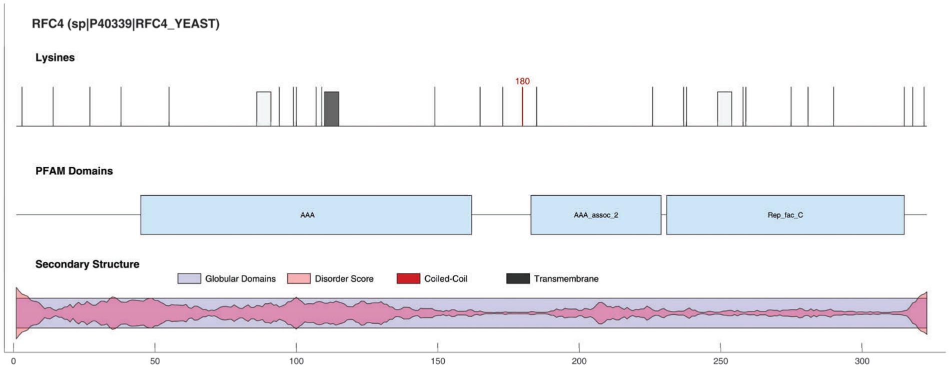
RFC4 (sp|P40339|RFC4_YEAST)
Lysines
180
PFAM Domains
AAA
AAA_assoc_2
Rep_fac_C
Secondary Structure
Globular Domains
Disorder Score
Coiled-Coil
Transmembrane
0
50
100
150
200
250
300

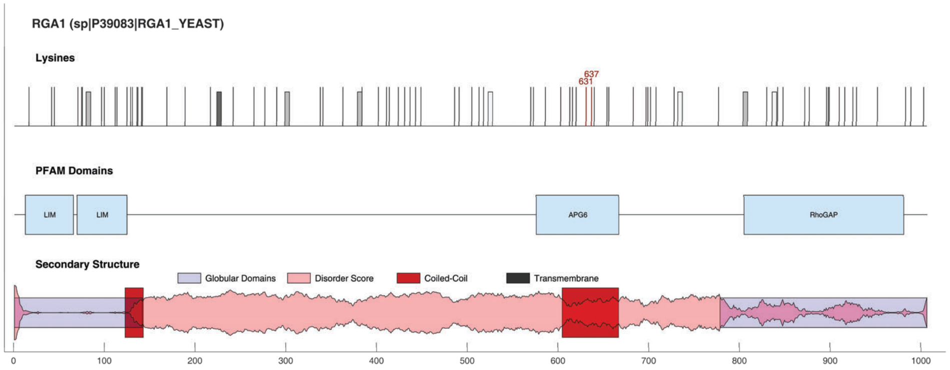

RGA1 (sp|P39083|RGA1_YEAST)
Lysines
637
631
PFAM Domains
LIM
LIM
APG6
RhoGAP
Secondary Structure
Globular Domains
Disorder Score
Coiled-Coil
Transmembrane
0
100
200
300
400
500
600
700
800
900
1000

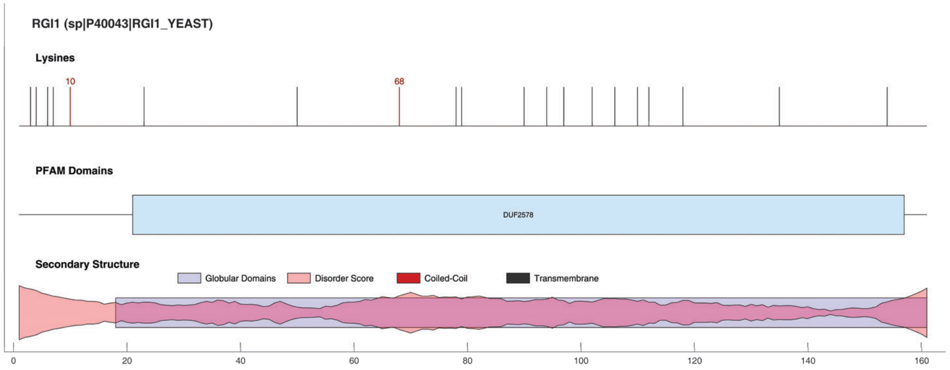

RGI1 (sp|P40043|RGI1_YEAST)
Lysines
10
68
PFAM Domains
DUF2578
Secondary Structure
Globular Domains
Disorder Score
Coiled-Coil
Transmembrane
0
20
40
60
80
100
120
140
160

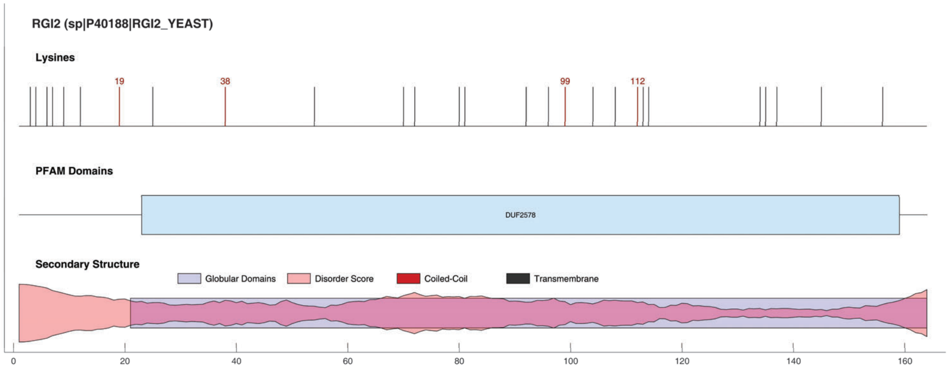

RGI2 (sp|P40188|RGI2_YEAST)
Lysines
19
38
99
112
PFAM Domains
DUF2578
Secondary Structure
Globular Domains
Disorder Score
Coiled-Coil
Transmembrane
0
20
40
60
80
100
120
140
160

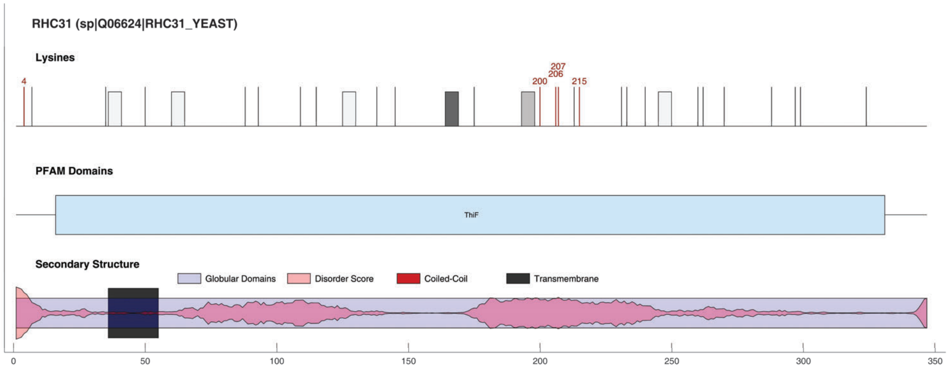

RHC31 (sp|Q06624|RHC31_YEAST)
Lysines
4
200
207
206
215
PFAM Domains
ThiF
Secondary Structure
Globular Domains
Disorder Score
Coiled-Coil
Transmembrane
0
50
100
150
200
250
300
350

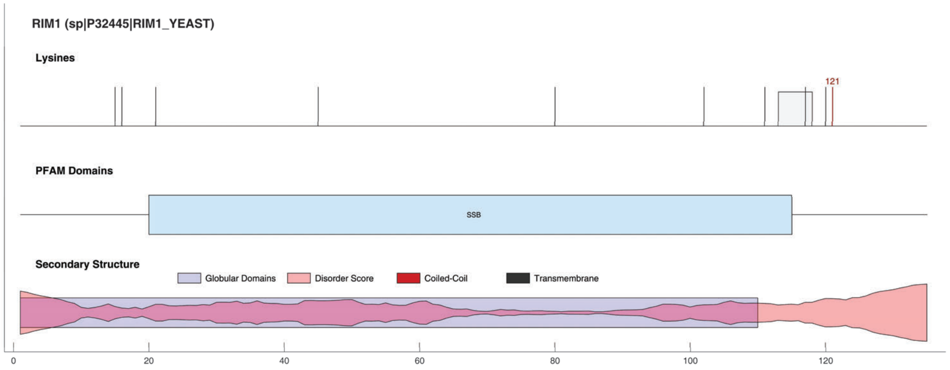
RIM1 (sp|P32445|RIM1_YEAST)
Lysines
121
PFAM Domains
SSB
Secondary Structure
Globular Domains
Disorder Score
Coiled-Coil
Transmembrane
0
20
40
60
80
100
120

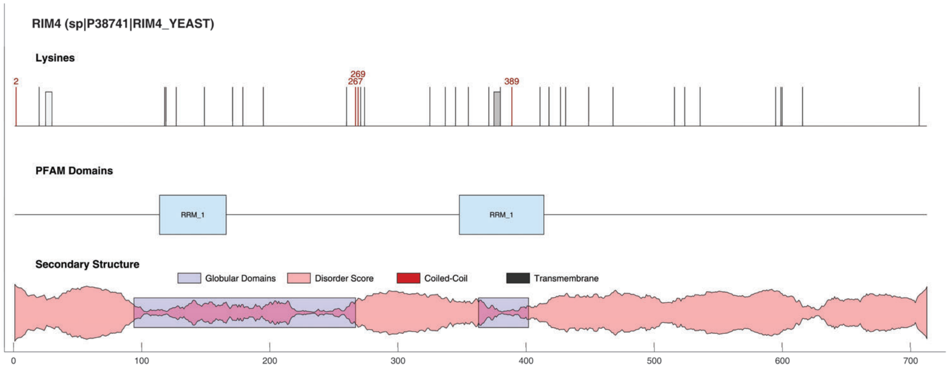
RIM4 (sp|P38741|RIM4_YEAST)
Lysines
2
269
267
389
PFAM Domains
RRM_1
RRM_1
Secondary Structure
Globular Domains
Disorder Score
Coiled-Coil
Transmembrane
0
100
200
300
400
500
600
700

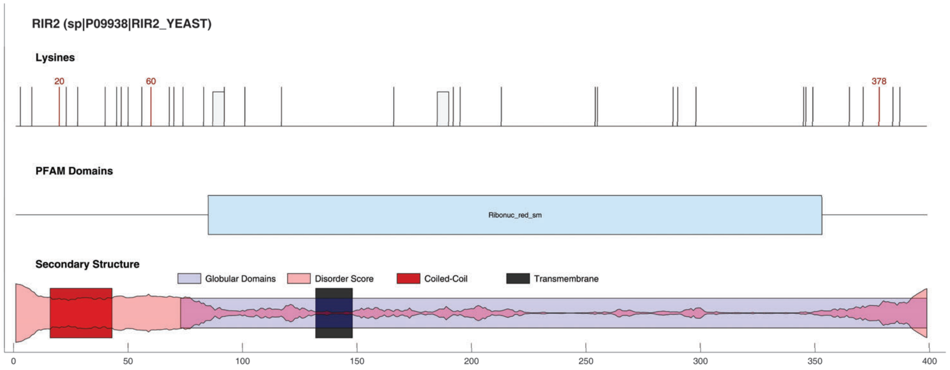
RIR2 (sp|P09938|RIR2_YEAST)
Lysines
20
60
378
PFAM Domains
Ribonuc_red_sm
Secondary Structure
Globular Domains
Disorder Score
Coiled-Coil
Transmembrane
0
50
100
150
200
250
300
350
400

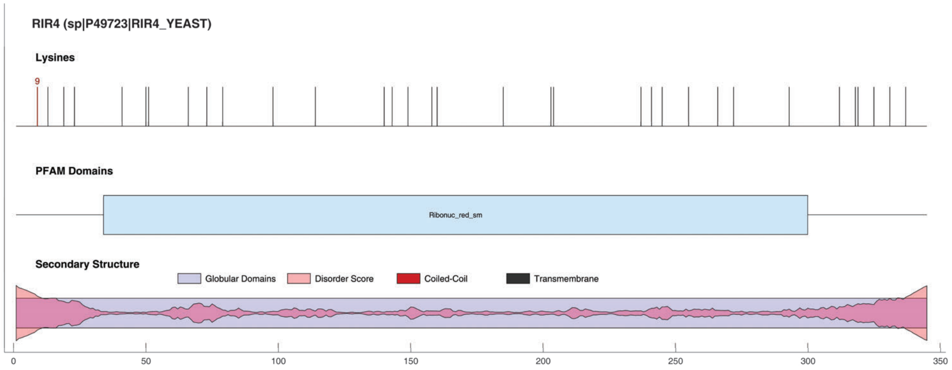
RIR4 (sp|P49723|RIR4_YEAST)
Lysines
9
PFAM Domains
Ribonuc_red_sm
Secondary Structure
Globular Domains
Disorder Score
Coiled-Coil
Transmembrane
0
50
100
150
200
250
300
350

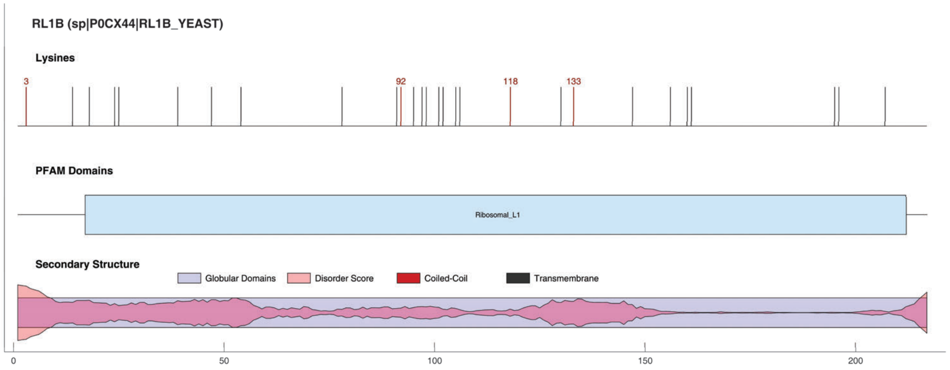
RL1B (sp|P0CX44|RL1B_YEAST)
Lysines
3
92
118
133
PFAM Domains
Ribosomal_L1
Secondary Structure
Globular Domains
Disorder Score
Coiled-Coil
Transmembrane
0
50
100
150
200

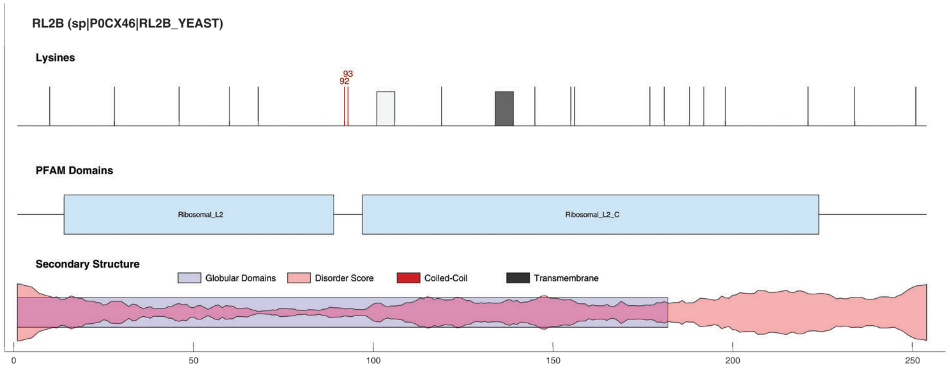
RL2B (sp|P0CX46|RL2B_YEAST)
Lysines
93
92
PFAM Domains
Ribosomal_L2
Ribosomal_L2_C
Secondary Structure
Globular Domains
Disorder Score
Coiled-Coil
Transmembrane
0
50
100
150
200
250

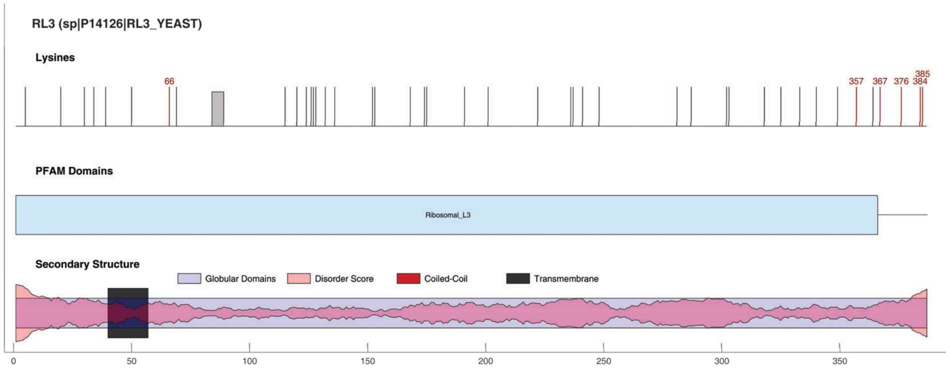
RL3 (sp|P14126|RL3_YEAST)
Lysines
66
357
367
376
384
385
PFAM Domains
Ribosomal_L3
Secondary Structure
Globular Domains
Disorder Score
Coiled-Coil
Transmembrane
0
50
100
150
200
250
300
350

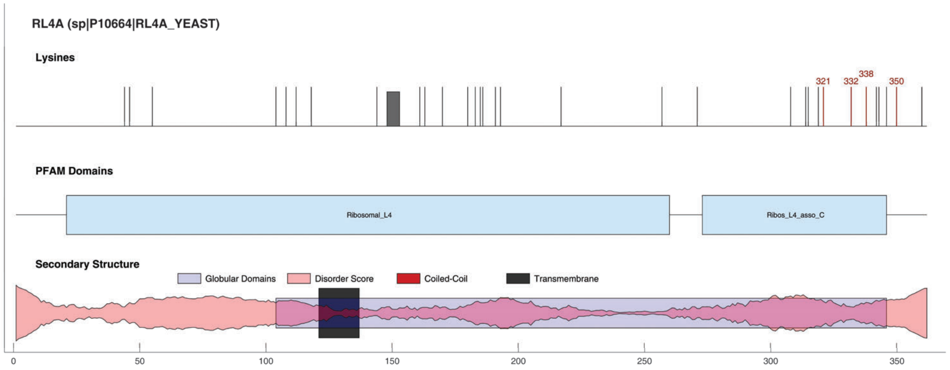
RL4A (sp|P10664|RL4A_YEAST)
Lysines
321
332
338
350
PFAM Domains
Ribosomal_L4
Ribos_L4_asso_C
Secondary Structure
Globular Domains
Disorder Score
Coiled-Coil
Transmembrane
0
50
100
150
200
250
300
350

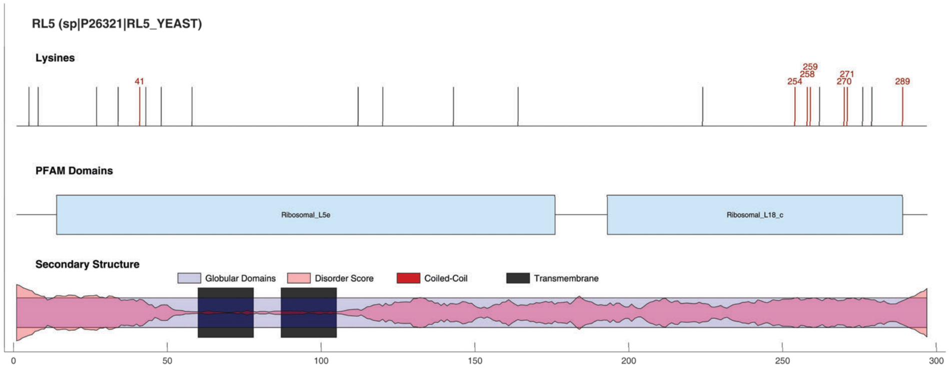
RL5 (sp|P26321|RL5_YEAST)
Lysines
41
254
258
259
270
271
289
PFAM Domains
Ribosomal_L5e
Ribosomal_L18_c
Secondary Structure
Globular Domains
Disorder Score
Coiled-Coil
Transmembrane
0
50
100
150
200
250
300

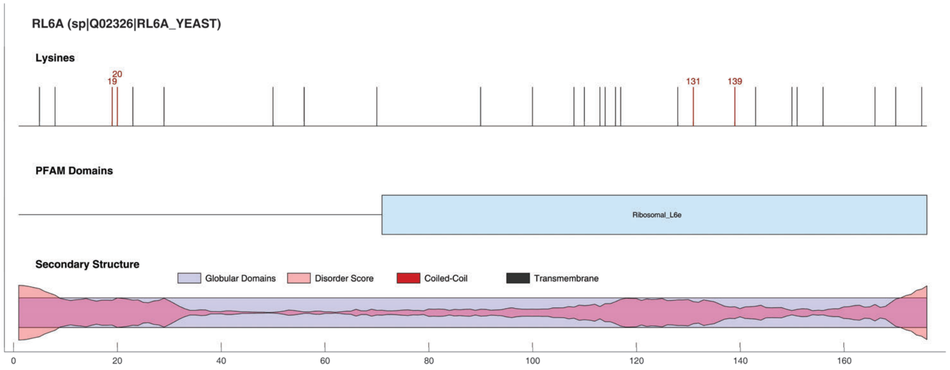
RL6A (sp|Q02326|RL6A_YEAST)
Lysines
19
20
131
139
PFAM Domains
Ribosomal_L6e
Secondary Structure
Globular Domains
Disorder Score
Coiled-Coil
Transmembrane
0
20
40
60
80
100
120
140
160

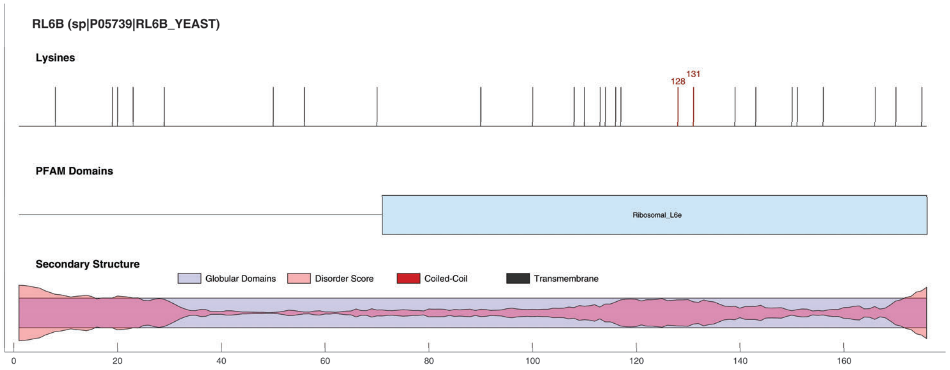
RL6B (sp|P05739|RL6B_YEAST)
Lysines
128
131
PFAM Domains
Ribosomal_L6e
Secondary Structure
Globular Domains
Disorder Score
Coiled-Coil
Transmembrane
0
20
40
60
80
100
120
140
160

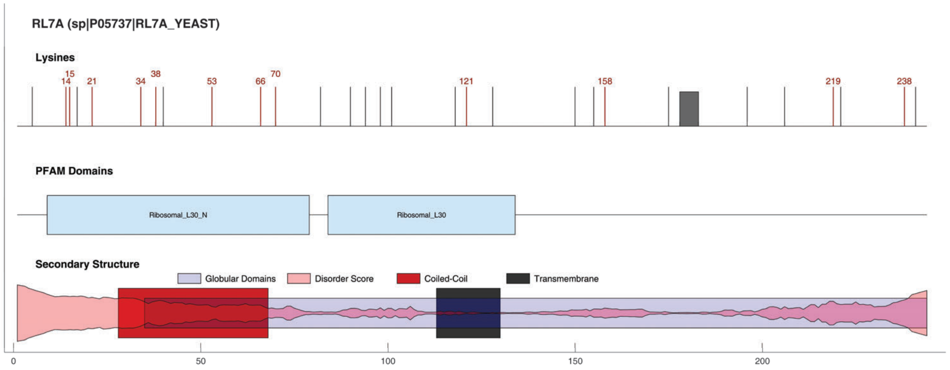
RL7A (sp|P05737|RL7A_YEAST)
Lysines
14
15
21
34
38
53
66
70
121
158
219
238
PFAM Domains
Ribosomal_L30_N
Ribosomal_L30
Secondary Structure
Globular Domains
Disorder Score
Coiled-Coil
Transmembrane
0
50
100
150
200

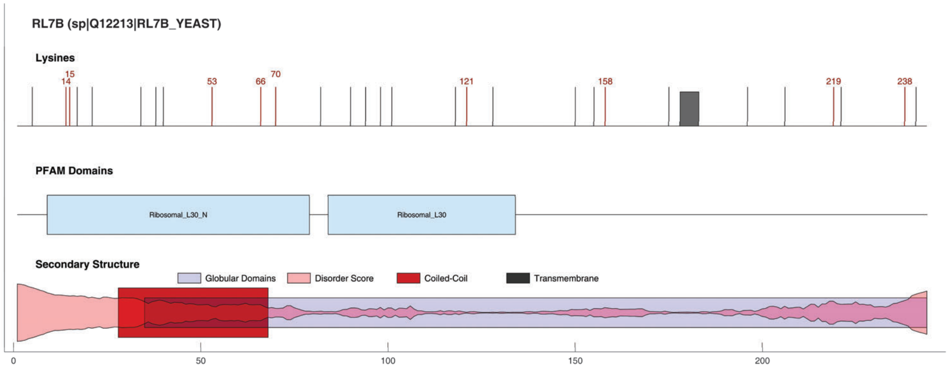
RL7B (sp|Q12213|RL7B_YEAST)
Lysines
14
15
53
66
70
121
158
219
238
PFAM Domains
Ribosomal_L30_N
Ribosomal_L30
Secondary Structure
Globular Domains
Disorder Score
Coiled-Coil
Transmembrane
0
50
100
150
200

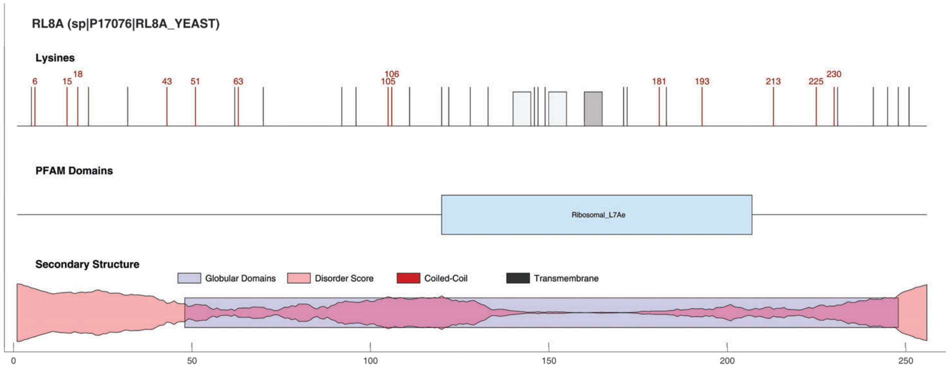
RL8A (sp|P17076|RL8A_YEAST)
Lysines
6
15
18
43
51
63
105
106
181
193
213
225
230
PFAM Domains
Ribosomal_L7Ae
Secondary Structure
Globular Domains
Disorder Score
Coiled-Coil
Transmembrane
0
50
100
150
200
250

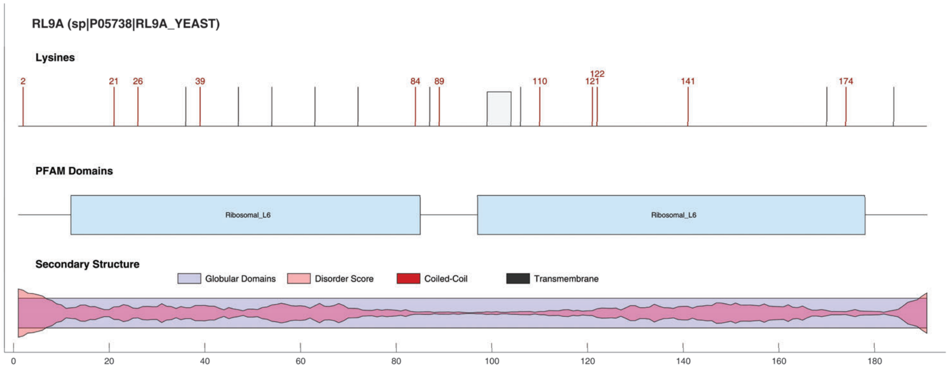
RL9A (sp|P05738|RL9A_YEAST)
Lysines
2
21
26
39
84
89
110
121
122
141
174
PFAM Domains
Ribosomal_L6
Ribosomal_L6
Secondary Structure
Globular Domains
Disorder Score
Coiled-Coil
Transmembrane
0
20
40
60
80
100
120
140
160
180

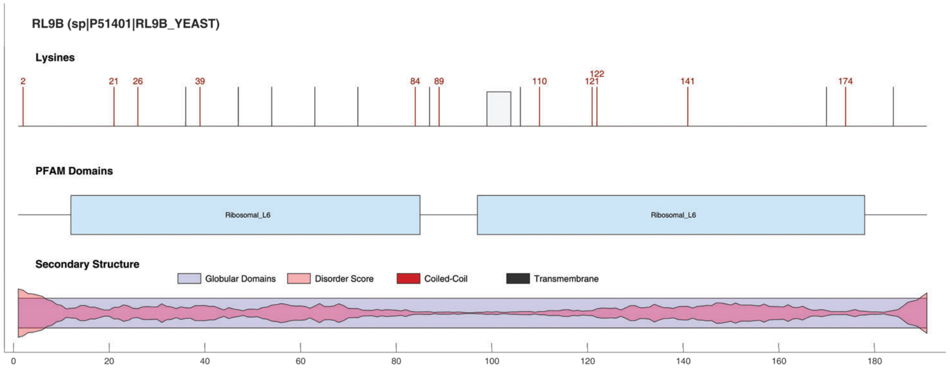
RL9B (sp|P51401|RL9B_YEAST)
Lysines
2
21
26
39
84
89
110
121
122
141
174
PFAM Domains
Ribosomal_L6
Ribosomal_L6
Secondary Structure
Globular Domains
Disorder Score
Coiled-Coil
Transmembrane
0
20
40
60
80
100
120
140
160
180

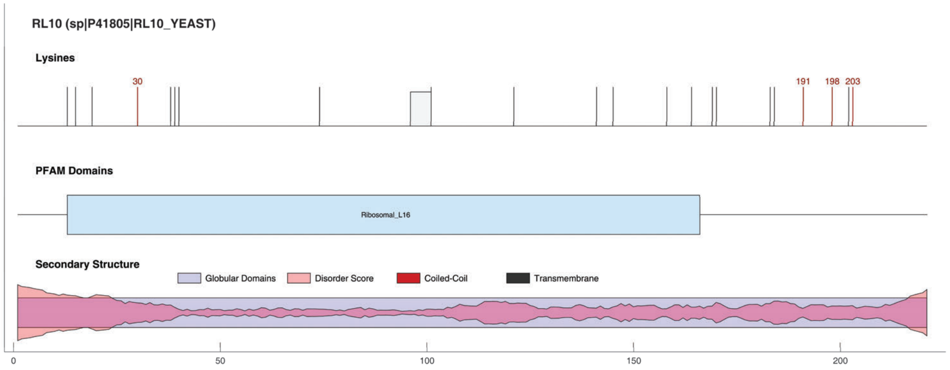
RL10 (sp|P41805|RL10_YEAST)
Lysines
30
191
198
203
PFAM Domains
Ribosomal_L16
Secondary Structure
Globular Domains
Disorder Score
Coiled-Coil
Transmembrane
0
50
100
150
200

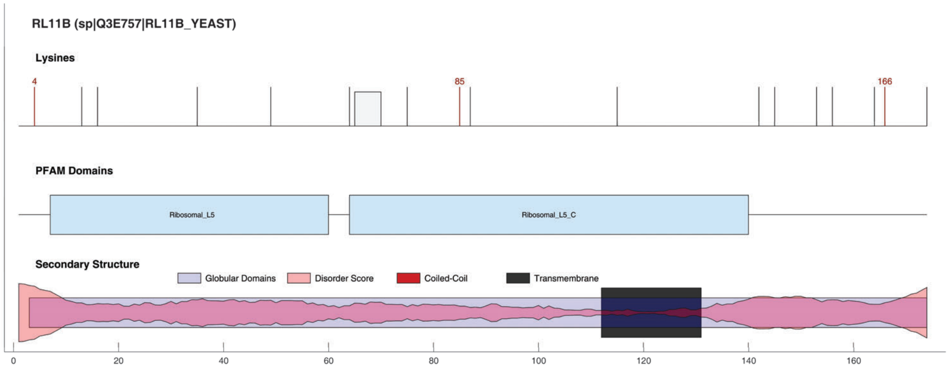
RL11B (sp|Q3E757|RL11B_YEAST)
Lysines
4
85
166
PFAM Domains
Ribosomal_L5
Ribosomal_L5_C
Secondary Structure
Globular Domains
Disorder Score
Coiled-Coil
Transmembrane
0
20
40
60
80
100
120
140
160

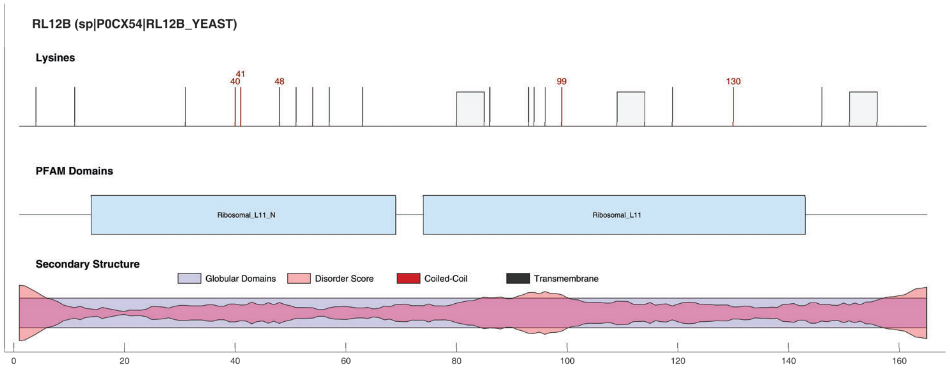
RL12B (sp|P0CX54|RL12B_YEAST)
Lysines
40
41
48
99
130
PFAM Domains
Ribosomal_L11_N
Ribosomal_L11
Secondary Structure
Globular Domains
Disorder Score
Coiled-Coil
Transmembrane
0
20
40
60
80
100
120
140
160

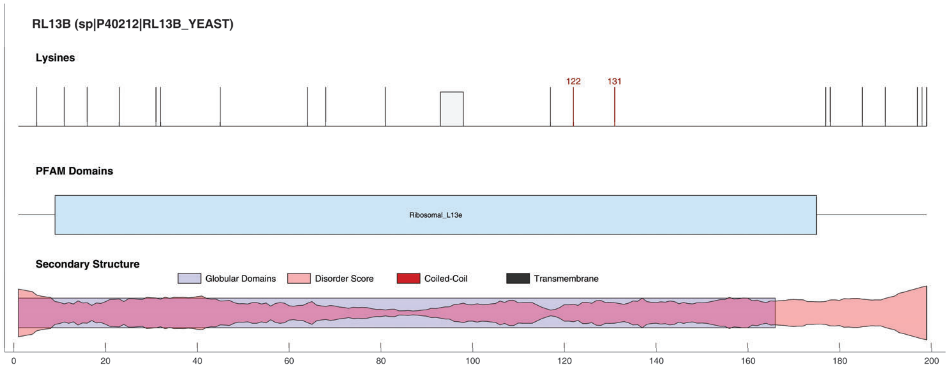
RL13B (sp|P40212|RL13B_YEAST)
Lysines
122
131
PFAM Domains
Ribosomal_L13e
Secondary Structure
Globular Domains
Disorder Score
Coiled-Coil
Transmembrane
0
20
40
60
80
100
120
140
160
180
200

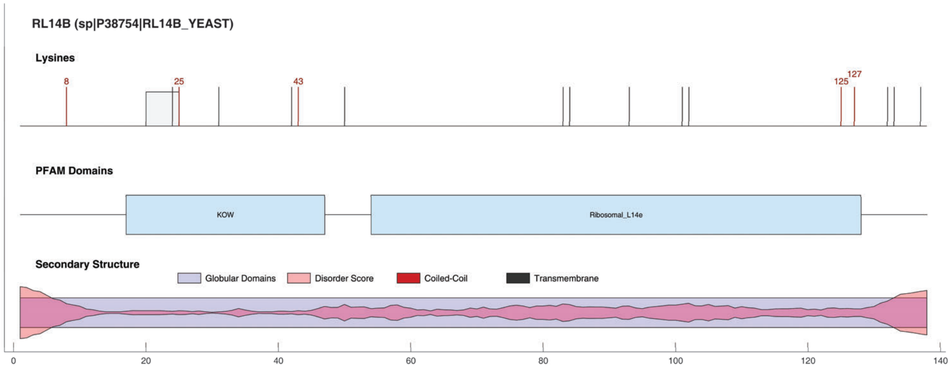
RL14B (sp|P38754|RL14B_YEAST)
Lysines
8
25
43
125
127
PFAM Domains
KOW
Ribosomal_L14e
Secondary Structure
Globular Domains
Disorder Score
Coiled-Coil
Transmembrane
0
20
40
60
80
100
120
140

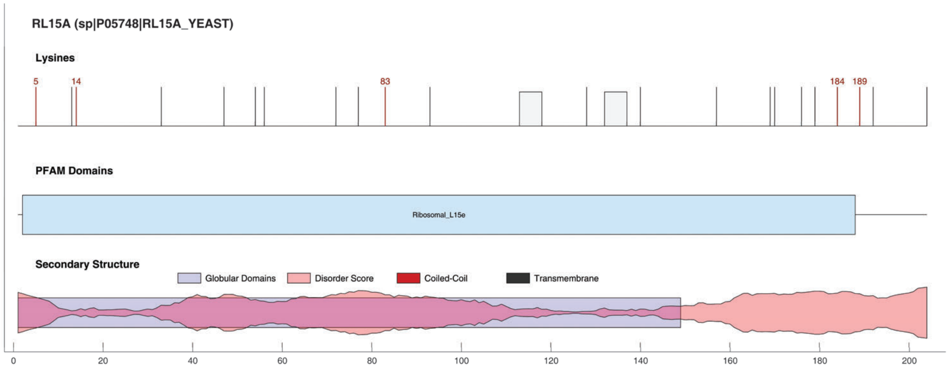
RL15A (sp|P05748|RL15A_YEAST)
Lysines
5
14
83
184
189
PFAM Domains
Ribosomal_L15e
Secondary Structure
Globular Domains
Disorder Score
Coiled-Coil
Transmembrane
0
20
40
60
80
100
120
140
160
180
200

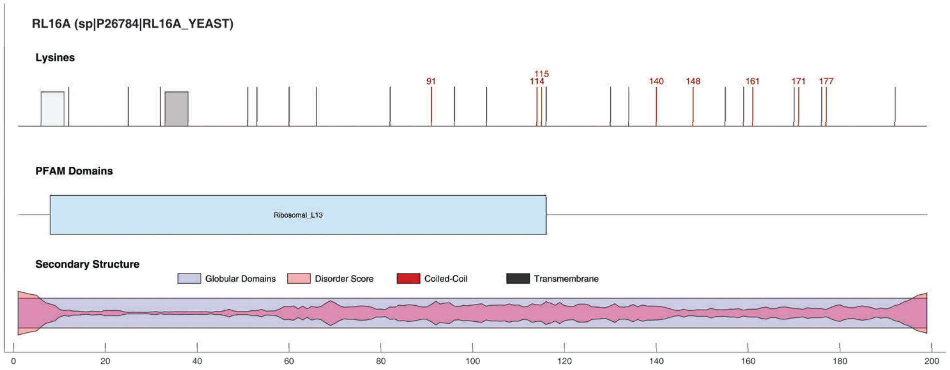
RL16A (sp|P26784|RL16A_YEAST)
Lysines
91
114
115
140
148
161
171
177
PFAM Domains
Ribosomal_L13
Secondary Structure
Globular Domains
Disorder Score
Coiled-Coil
Transmembrane
0
20
40
60
80
100
120
140
160
180
200

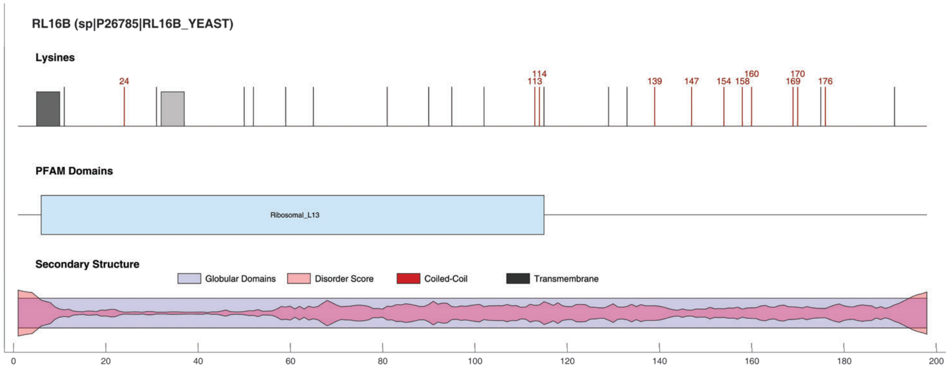
RL16B (sp|P26785|RL16B_YEAST)
Lysines
24
113
114
139
147
154
158
160
169
170
176
PFAM Domains
Ribosomal_L13
Secondary Structure
Globular Domains
Disorder Score
Coiled-Coil
Transmembrane
0
20
40
60
80
100
120
140
160
180
200

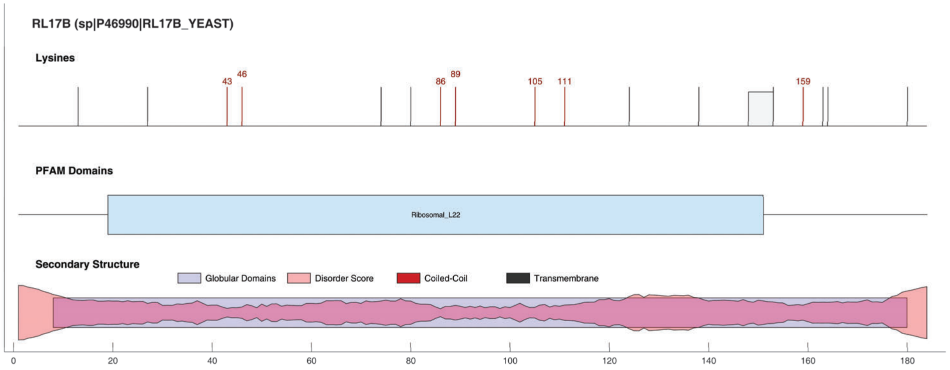
RL17B (sp|P46990|RL17B_YEAST)
Lysines
43
46
86
89
105
111
159
PFAM Domains
Ribosomal_L22
Secondary Structure
Globular Domains
Disorder Score
Coiled-Coil
Transmembrane
0
20
40
60
80
100
120
140
160
180

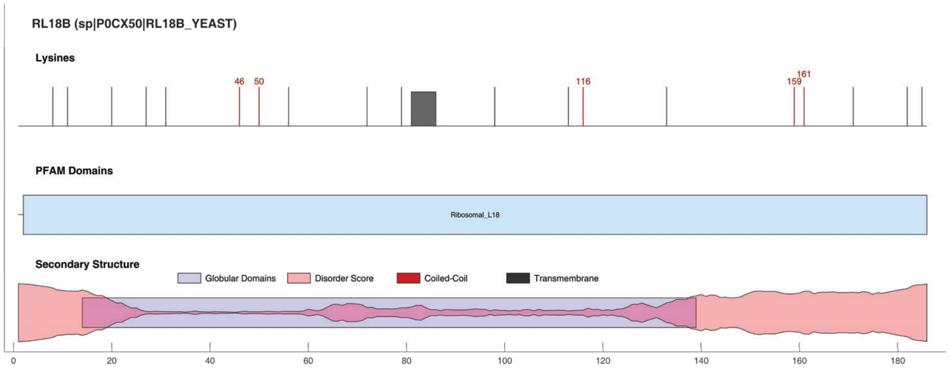
RL18B (sp|P0CX50|RL18B_YEAST)
Lysines
46
50
116
159
161
PFAM Domains
Ribosomal_L18
Secondary Structure
Globular Domains
Disorder Score
Coiled-Coil
Transmembrane
0
20
40
60
80
100
120
140
160
180

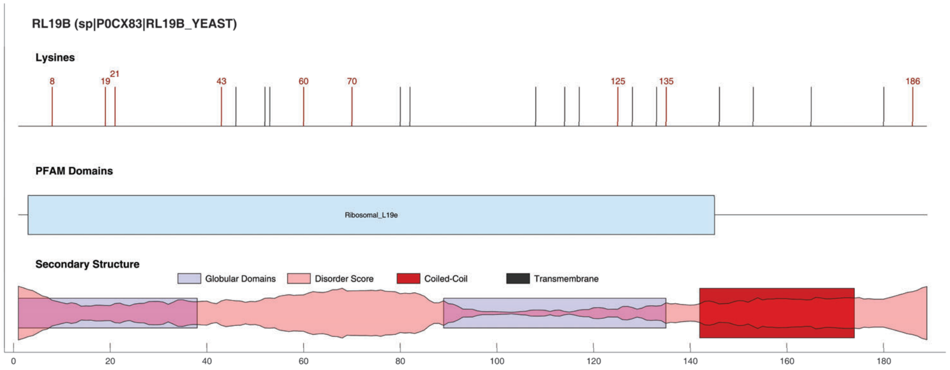
RL19B (sp|P0CX83|RL19B_YEAST)
Lysines
8
19
21
43
60
70
125
135
186
PFAM Domains
Ribosomal_L19e
Secondary Structure
Globular Domains
Disorder Score
Coiled-Coil
Transmembrane
0
20
40
60
80
100
120
140
160
180

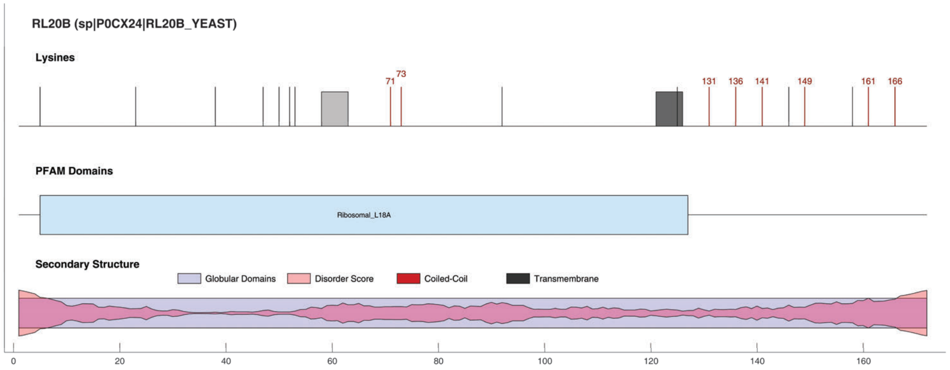
RL20B (sp|P0CX24|RL20B_YEAST)
Lysines
71
73
131
136
141
149
161
166
PFAM Domains
Ribosomal_L18A
Secondary Structure
Globular Domains
Disorder Score
Coiled-Coil
Transmembrane
0
20
40
60
80
100
120
140
160

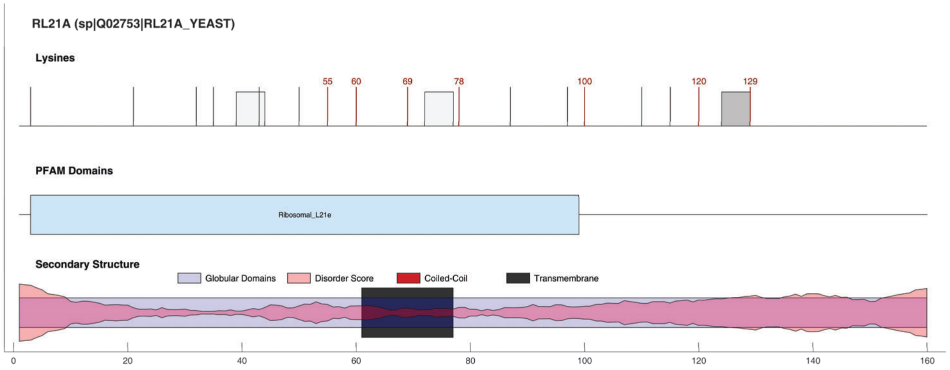
RL21A (sp|Q02753|RL21A_YEAST)
Lysines
55
60
69
78
100
120
129
PFAM Domains
Ribosomal_L21e
Secondary Structure
Globular Domains
Disorder Score
Coiled-Coil
Transmembrane
0
20
40
60
80
100
120
140
160

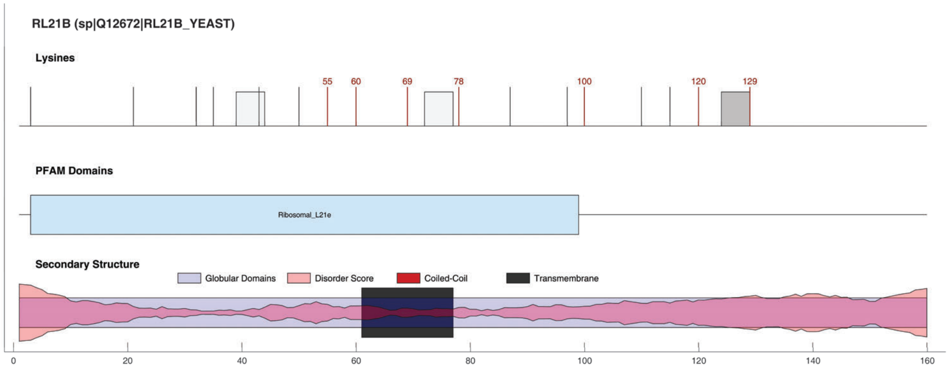
RL21B (sp|Q12672|RL21B_YEAST)
Lysines
55
60
69
78
100
120
129
PFAM Domains
Ribosomal_L21e
Secondary Structure
Globular Domains
Disorder Score
Coiled-Coil
Transmembrane
0
20
40
60
80
100
120
140
160

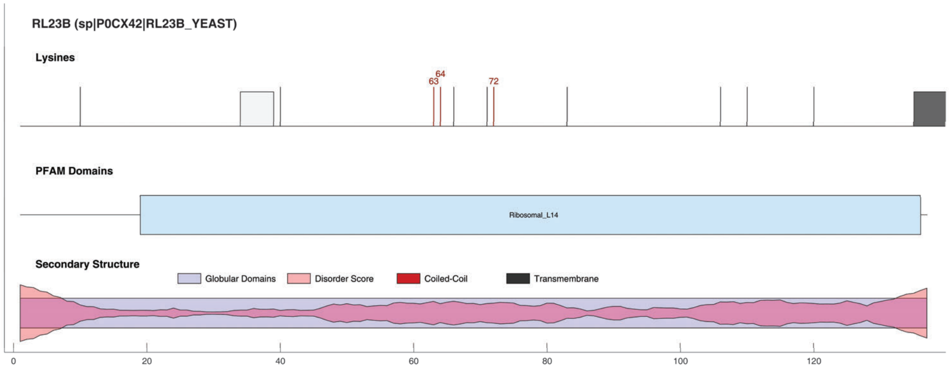
RL23B (sp|P0CX42|RL23B_YEAST)
Lysines
63
64
72
PFAM Domains
Ribosomal_L14
Secondary Structure
Globular Domains
Disorder Score
Coiled-Coil
Transmembrane
0
20
40
60
80
100
120

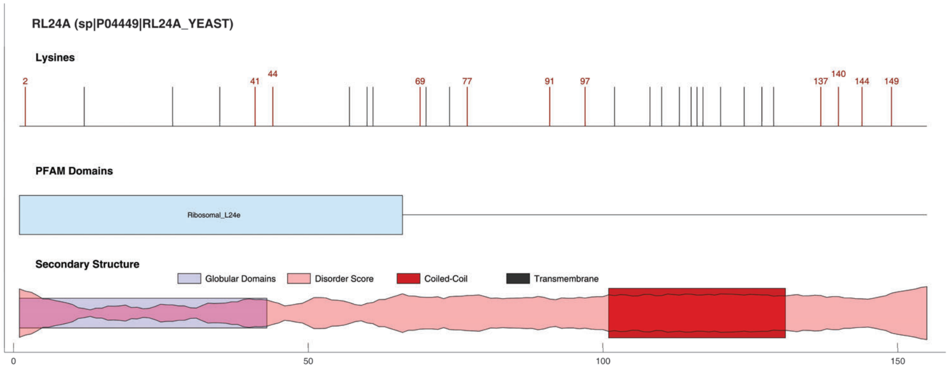
RL24A (sp|P04449|RL24A_YEAST)
Lysines
2
41
44
69
77
91
97
137
140
144
149
PFAM Domains
Ribosomal_L24e
Secondary Structure
Globular Domains
Disorder Score
Coiled-Coil
Transmembrane
0
50
100
150

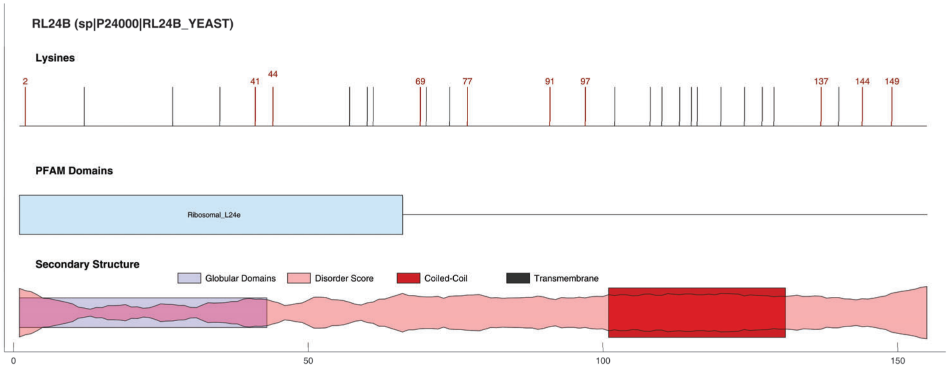
RL24B (sp|P24000|RL24B_YEAST)
Lysines
2 41 44 69 77 91 97 137 144 149
PFAM Domains
Ribosomal_L24e
Secondary Structure
Globular Domains
Disorder Score
Coiled-Coil
Transmembrane
0 50 100 150

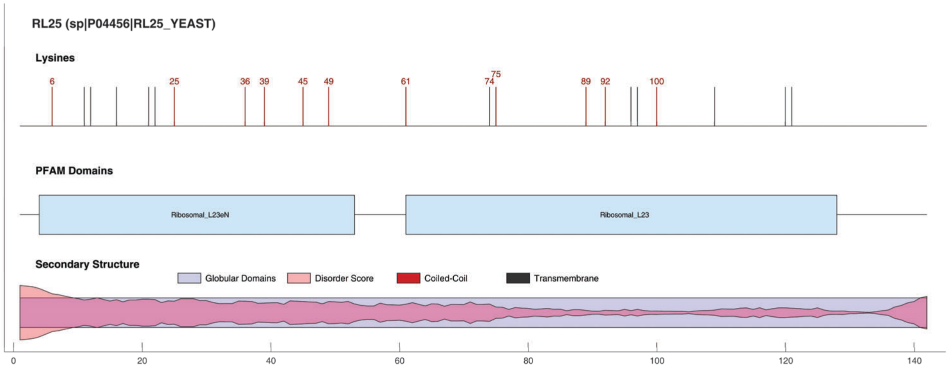
RL25 (sp|P04456|RL25_YEAST)
Lysines
6 25 36 39 45 49 61 74 75 89 92 100
PFAM Domains
Ribosomal_L23eN
Ribosomal_L23
Secondary Structure
Globular Domains
Disorder Score
Coiled-Coil
Transmembrane
0 20 40 60 80 100 120 140

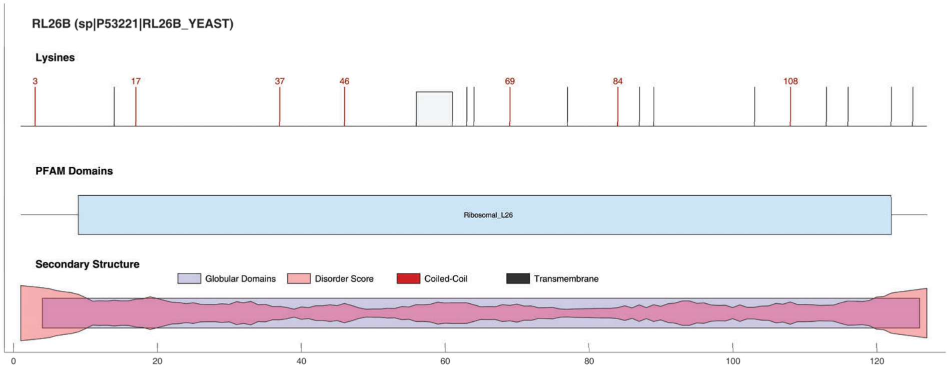
RL26B (sp|P53221|RL26B_YEAST)
Lysines
3 17 37 46 69 84 108
PFAM Domains
Ribosomal_L26
Secondary Structure
Globular Domains
Disorder Score
Coiled-Coil
Transmembrane
0 20 40 60 80 100 120

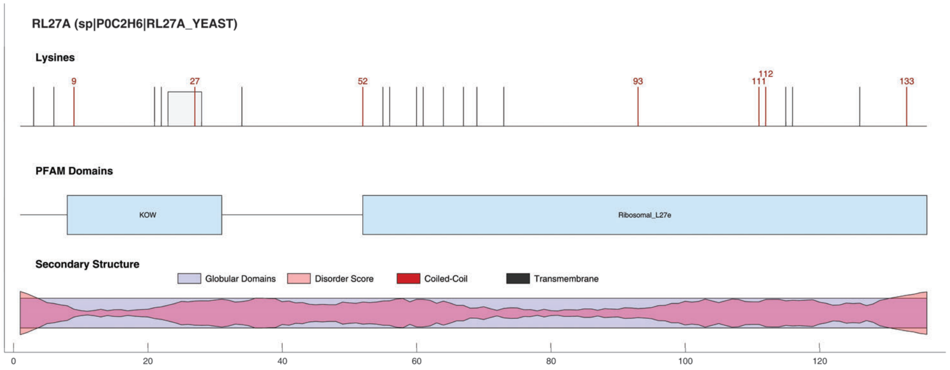
RL27A (sp|P0C2H6|RL27A_YEAST)
Lysines
9 27 52 93 111 112 133
PFAM Domains
KOW
Ribosomal_L27e
Secondary Structure
Globular Domains
Disorder Score
Coiled-Coil
Transmembrane
0 20 40 60 80 100 120

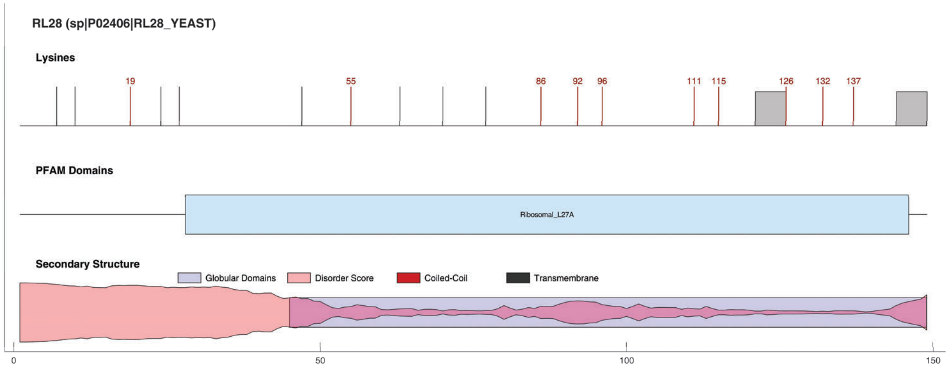
RL28 (sp|P02406|RL28_YEAST)
Lysines
19
55
86
92
96
111
115
126
132
137
PFAM Domains
Ribosomal_L27A
Secondary Structure
Globular Domains
Disorder Score
Coiled-Coil
Transmembrane
0
50
100
150

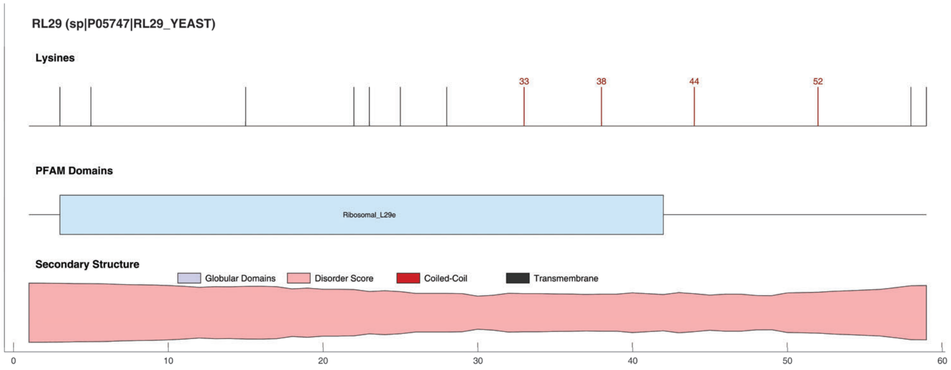
RL29 (sp|P05747|RL29_YEAST)
Lysines
33
38
44
52
PFAM Domains
Ribosomal_L29e
Secondary Structure
Globular Domains
Disorder Score
Coiled-Coil
Transmembrane
0
10
20
30
40
50
60

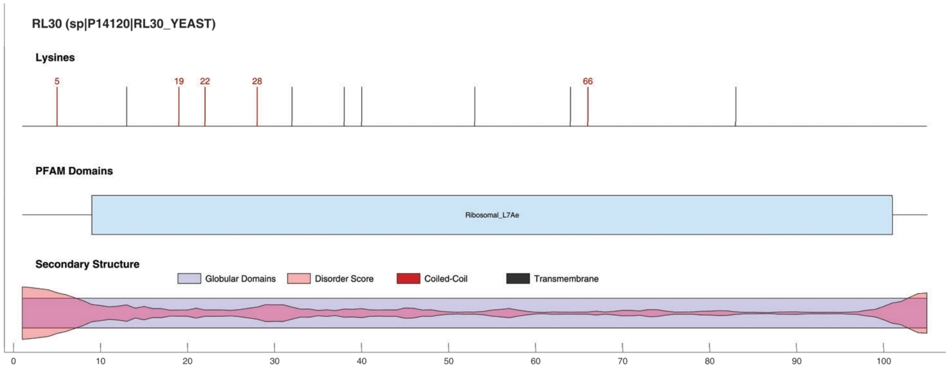
RL30 (sp|P14120|RL30_YEAST)
Lysines
5
19
22
28
66
PFAM Domains
Ribosomal_L7Ae
Secondary Structure
Globular Domains
Disorder Score
Coiled-Coil
Transmembrane
0
10
20
30
40
50
60
70
80
90
100

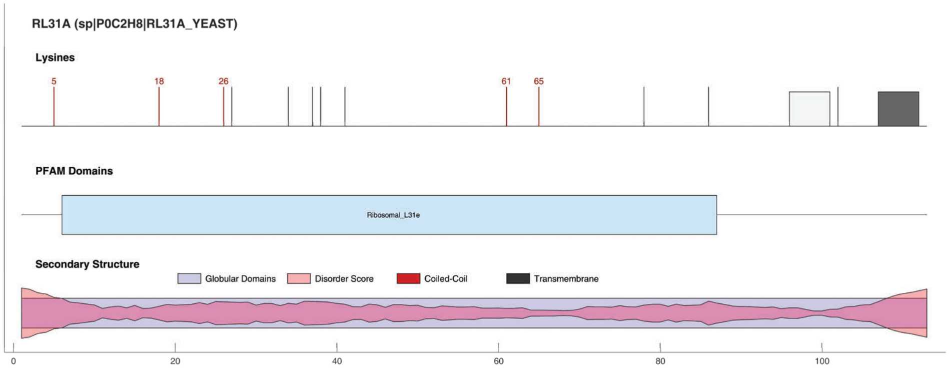
RL31A (sp|P0C2H8|RL31A_YEAST)
Lysines
5
18
26
61
65
PFAM Domains
Ribosomal_L31e
Secondary Structure
Globular Domains
Disorder Score
Coiled-Coil
Transmembrane
0
20
40
60
80
100

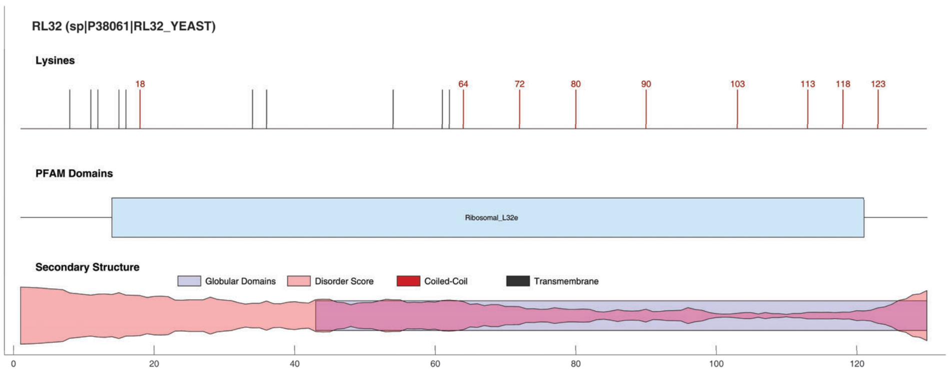
RL32 (sp|P38061|RL32_YEAST)
Lysines
18
64
72
80
90
103
113
118
123
PFAM Domains
Ribosomal_L32e
Secondary Structure
Globular Domains
Disorder Score
Coiled-Coil
Transmembrane
0
20
40
60
80
100
120

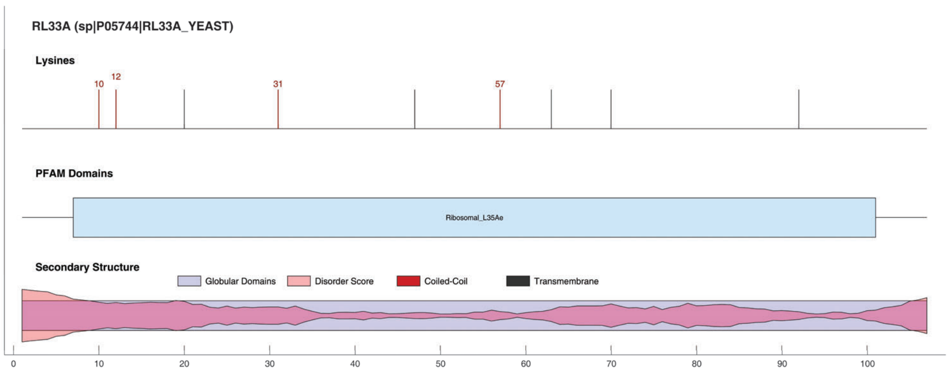
RL33A (sp|P05744|RL33A_YEAST)
Lysines
10
12
31
57
PFAM Domains
Ribosomal_L35Ae
Secondary Structure
Globular Domains
Disorder Score
Coiled-Coil
Transmembrane
0
10
20
30
40
50
60
70
80
90
100

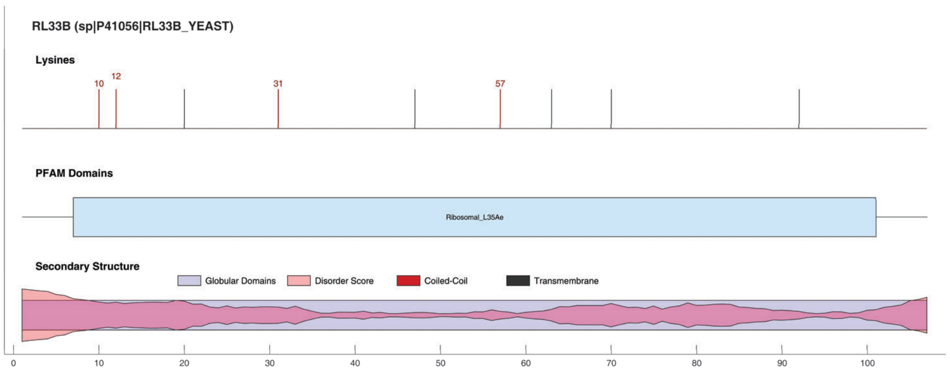
RL33B (sp|P41056|RL33B_YEAST)
Lysines
10
12
31
57
PFAM Domains
Ribosomal_L35Ae
Secondary Structure
Globular Domains
Disorder Score
Coiled-Coil
Transmembrane
0
10
20
30
40
50
60
70
80
90
100

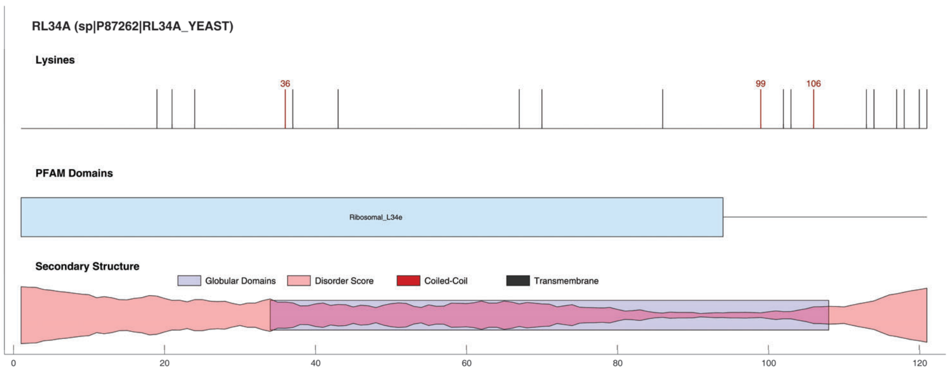
RL34A (sp|P87262|RL34A_YEAST)
Lysines
36
99
106
PFAM Domains
Ribosomal_L34e
Secondary Structure
Globular Domains
Disorder Score
Coiled-Coil
Transmembrane
0
20
40
60
80
100
120

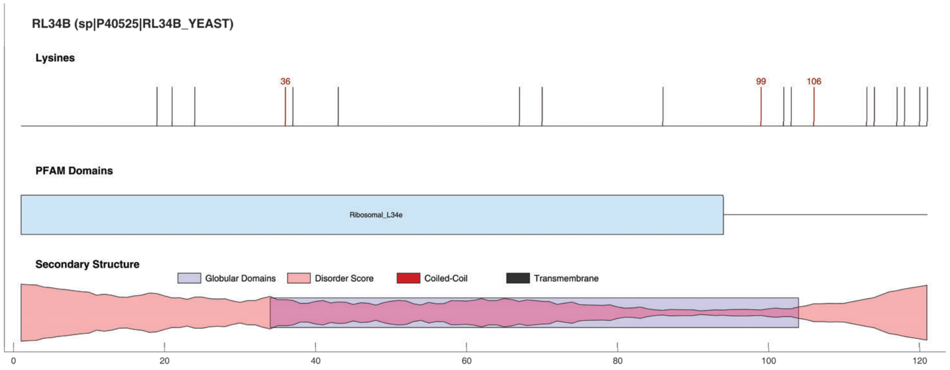
RL34B (sp|P40525|RL34B_YEAST)
Lysines
36
99
106
PFAM Domains
Ribosomal_L34e
Secondary Structure
Globular Domains
Disorder Score
Coiled-Coil
Transmembrane
0
20
40
60
80
100
120

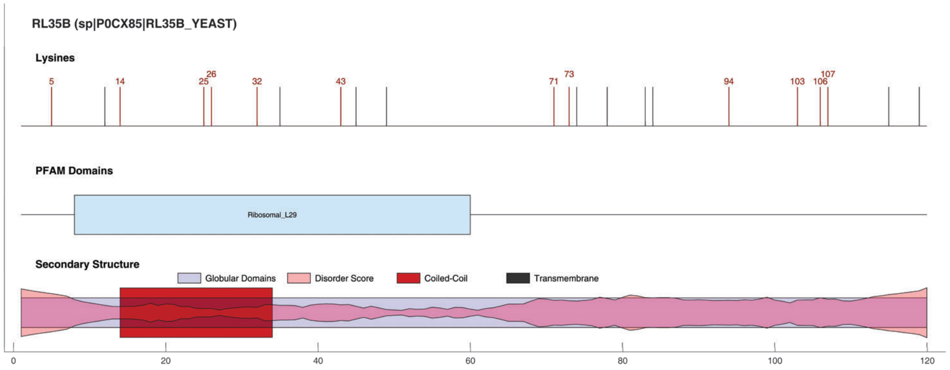
RL35B (sp|P0CX85|RL35B_YEAST)
Lysines
5
14
25
26
32
43
71
73
94
103
106
107
PFAM Domains
Ribosomal_L29
Secondary Structure
Globular Domains
Disorder Score
Coiled-Coil
Transmembrane
0
20
40
60
80
100
120

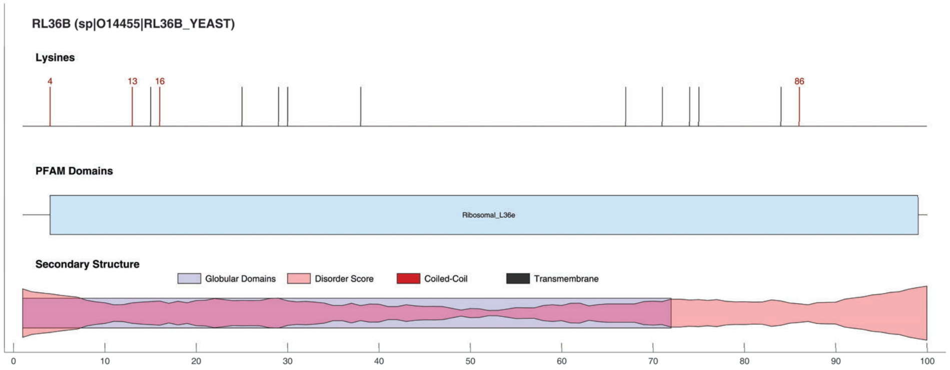
RL36B (sp|O14455|RL36B_YEAST)
Lysines
4
13
16
86
PFAM Domains
Ribosomal_L36e
Secondary Structure
Globular Domains
Disorder Score
Coiled-Coil
Transmembrane
0
10
20
30
40
50
60
70
80
90
100

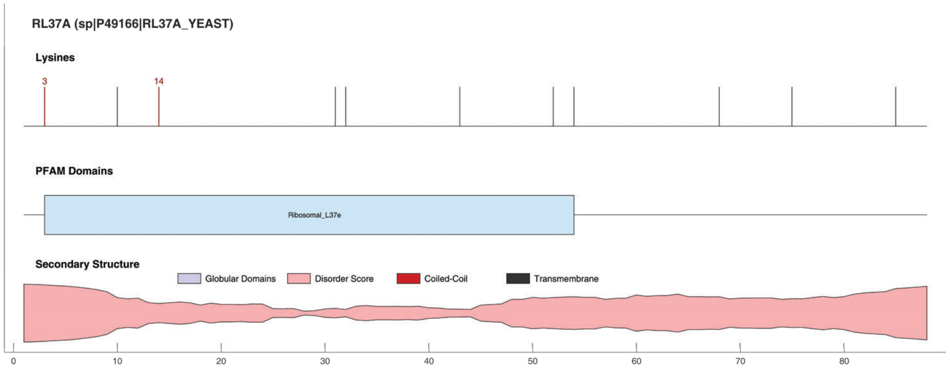
RL37A (sp|P49166|RL37A_YEAST)
Lysines
3
14
PFAM Domains
Ribosomal_L37e
Secondary Structure
Globular Domains
Disorder Score
Coiled-Coil
Transmembrane
0
10
20
30
40
50
60
70
80

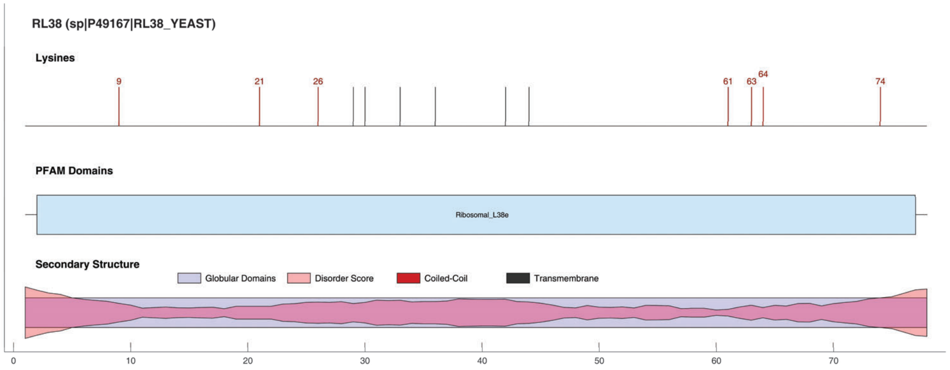

RL38 (sp|P49167|RL38_YEAST)
Lysines
9
21
26
61
63
64
74
PFAM Domains
Ribosomal_L38e
Secondary Structure
Globular Domains
Disorder Score
Coiled-Coil
Transmembrane
0
10
20
30
40
50
60
70

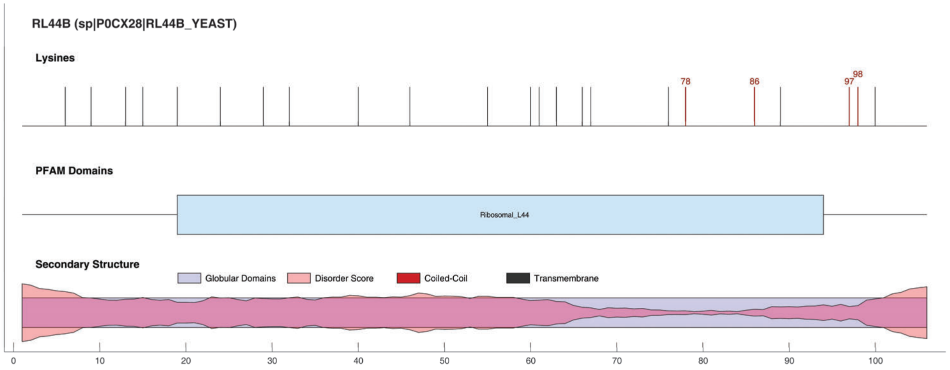

RL44B (sp|P0CX28|RL44B_YEAST)
Lysines
78
86
97
98
PFAM Domains
Ribosomal_L44
Secondary Structure
Globular Domains
Disorder Score
Coiled-Coil
Transmembrane
0
10
20
30
40
50
60
70
80
90
100

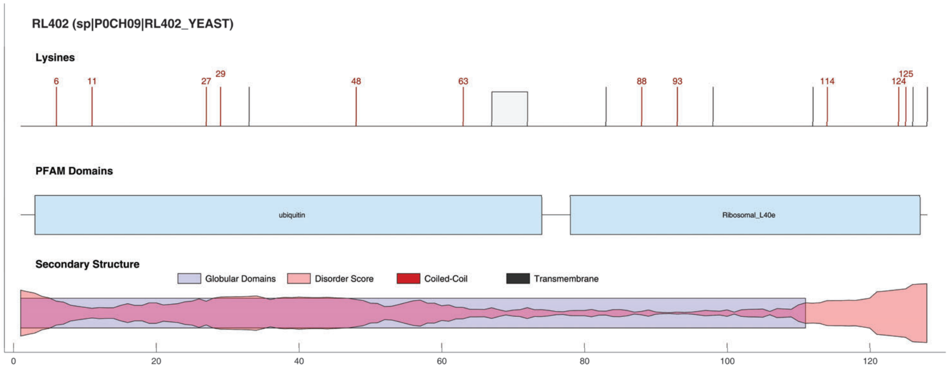

RL402 (sp|P0CH09|RL402_YEAST)
Lysines
6
11
27
29
48
63
88
93
114
124
125
PFAM Domains
ubiquitin
Ribosomal_L40e
Secondary Structure
Globular Domains
Disorder Score
Coiled-Coil
Transmembrane
0
20
40
60
80
100
120

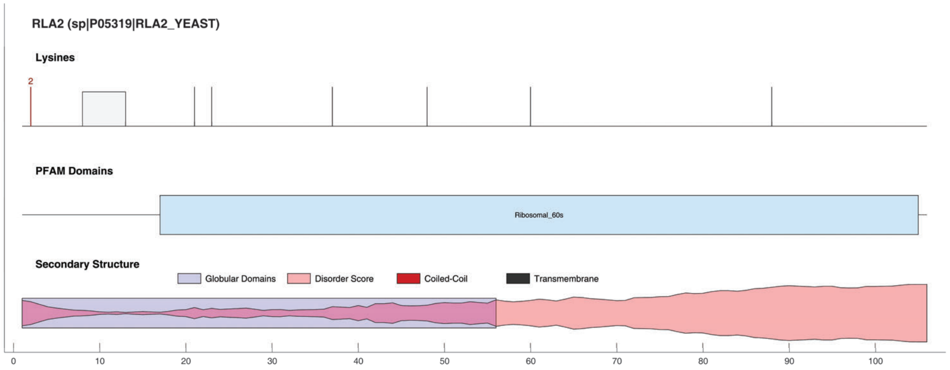

RLA2 (sp|P05319|RLA2_YEAST)
Lysines
2
PFAM Domains
Ribosomal_60s
Secondary Structure
Globular Domains
Disorder Score
Coiled-Coil
Transmembrane
0
10
20
30
40
50
60
70
80
90
100

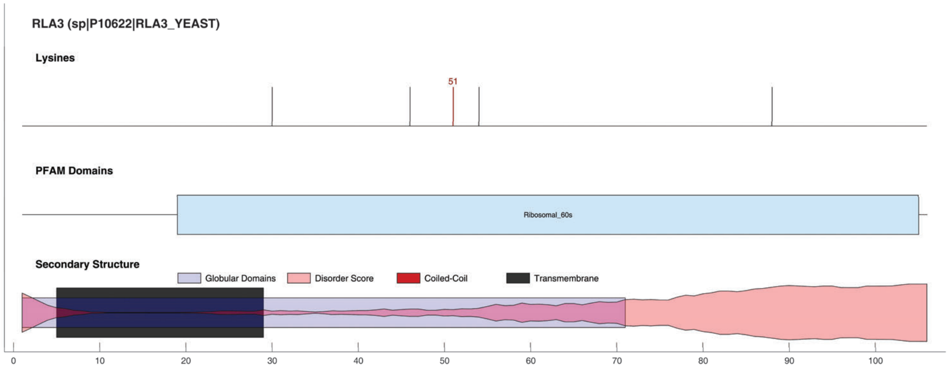
RLA3 (sp|P10622|RLA3_YEAST)
Lysines
51
PFAM Domains
Ribosomal_60s
Secondary Structure
Globular Domains
Disorder Score
Coiled-Coil
Transmembrane
0
10
20
30
40
50
60
70
80
90
100

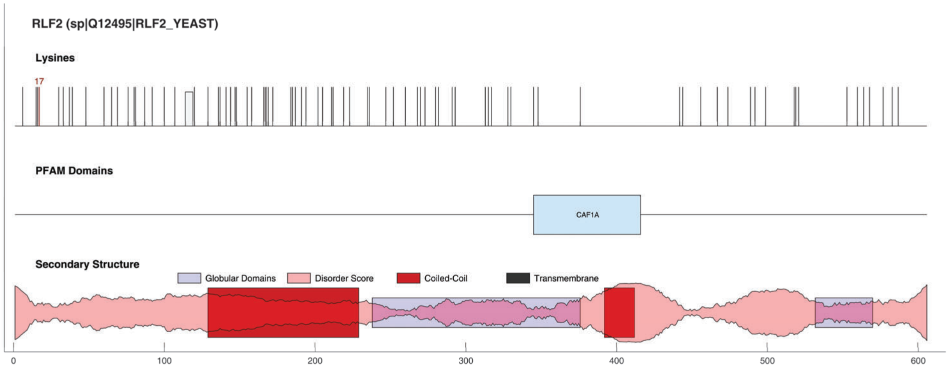
RLF2 (sp|Q12495|RLF2_YEAST)
Lysines
17
PFAM Domains
CAF1A
Secondary Structure
Globular Domains
Disorder Score
Coiled-Coil
Transmembrane
0
100
200
300
400
500
600

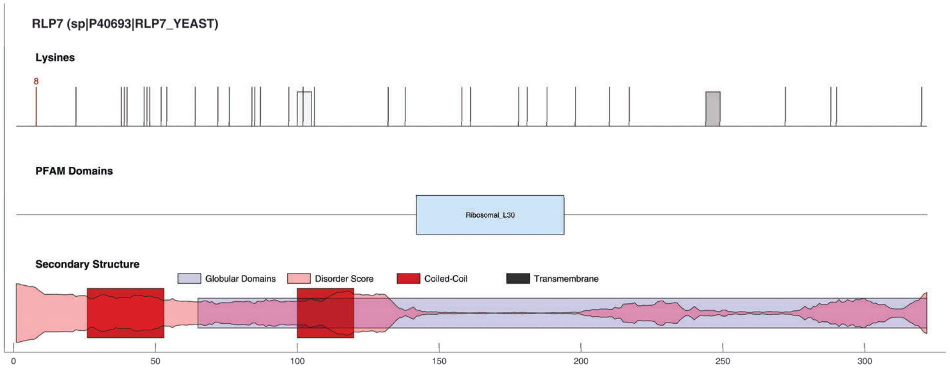
RLP7 (sp|P40693|RLP7_YEAST)
Lysines
8
PFAM Domains
Ribosomal_L30
Secondary Structure
Globular Domains
Disorder Score
Coiled-Coil
Transmembrane
0
50
100
150
200
250
300

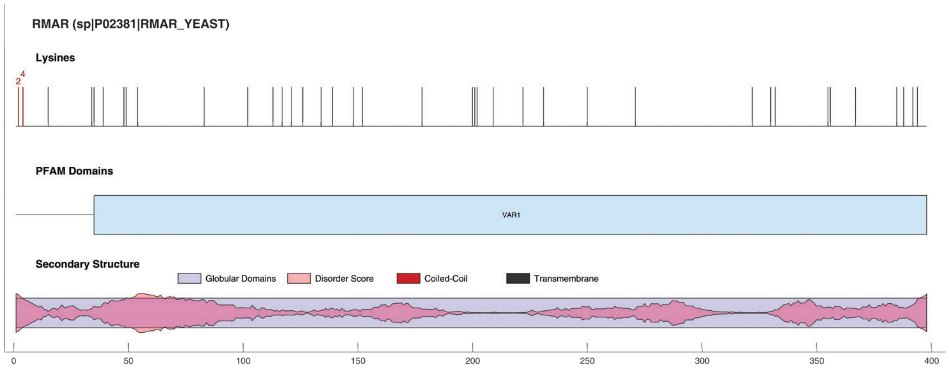
RMAR (sp|P02381|RMAR_YEAST)
Lysines
2
4
PFAM Domains
VAR1
Secondary Structure
Globular Domains
Disorder Score
Coiled-Coil
Transmembrane
0
50
100
150
200
250
300
350
400

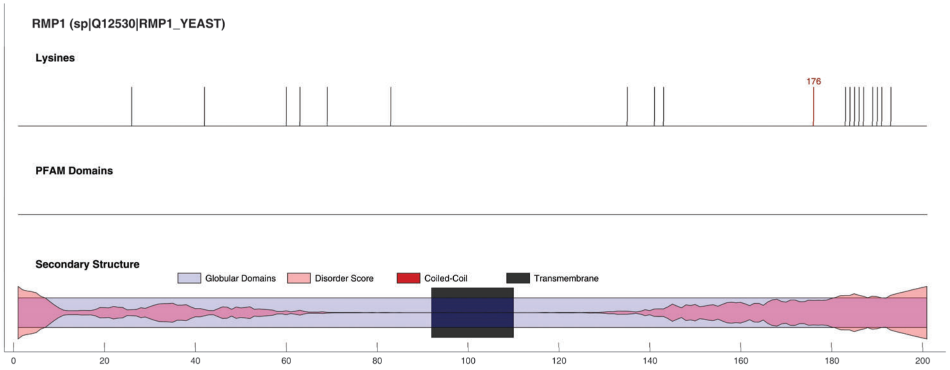

RMP1 (sp|Q12530|RMP1_YEAST)
Lysines
176
PFAM Domains
Secondary Structure
Globular Domains
Disorder Score
Coiled-Coil
Transmembrane
0
20
40
60
80
100
120
140
160
180
200

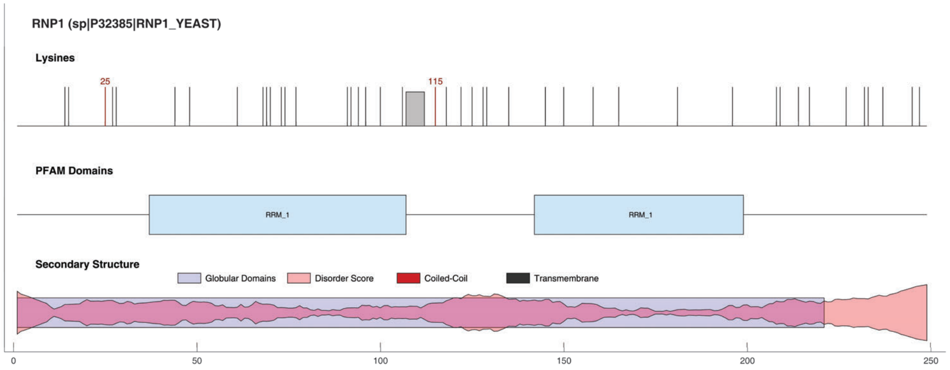

RNP1 (sp|P32385|RNP1_YEAST)
Lysines
25
115
PFAM Domains
RRM_1
RRM_1
Secondary Structure
Globular Domains
Disorder Score
Coiled-Coil
Transmembrane
0
50
100
150
200
250

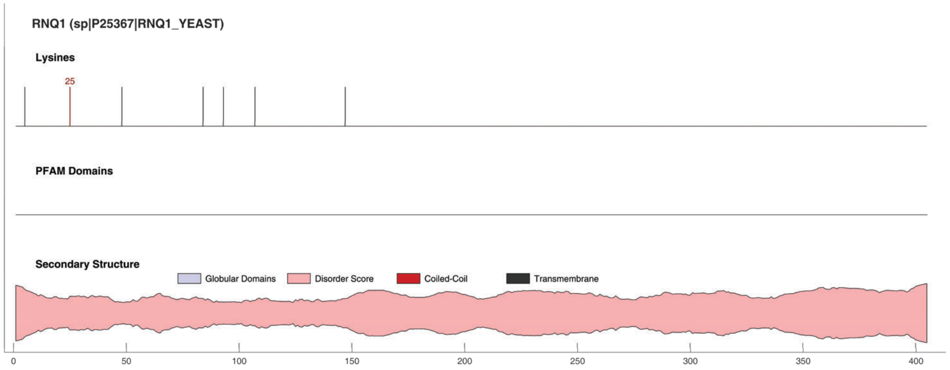

RNQ1 (sp|P25367|RNQ1_YEAST)
Lysines
25
PFAM Domains
Secondary Structure
Globular Domains
Disorder Score
Coiled-Coil
Transmembrane
0
50
100
150
200
250
300
350
400

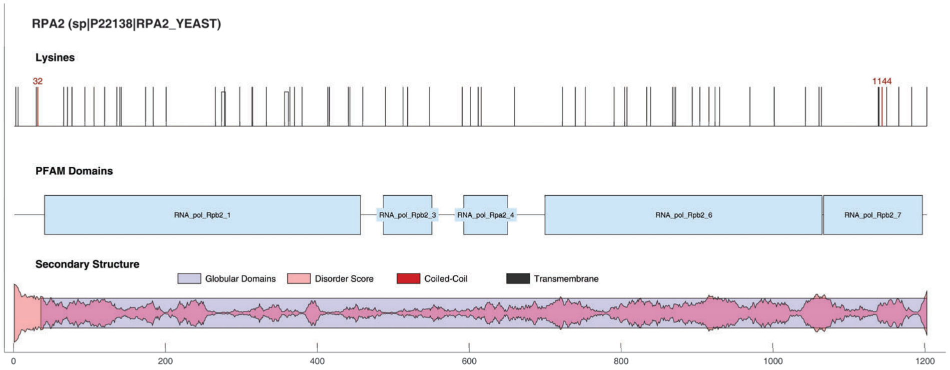

RPA2 (sp|P22138|RPA2_YEAST)
Lysines
32
1144
PFAM Domains
RNA_pol_Rpb2_1
RNA_pol_Rpb2_3
RNA_pol_Rpa2_4
RNA_pol_Rpb2_6
RNA_pol_Rpb2_7
Secondary Structure
Globular Domains
Disorder Score
Coiled-Coil
Transmembrane
0
200
400
600
800
1000
1200

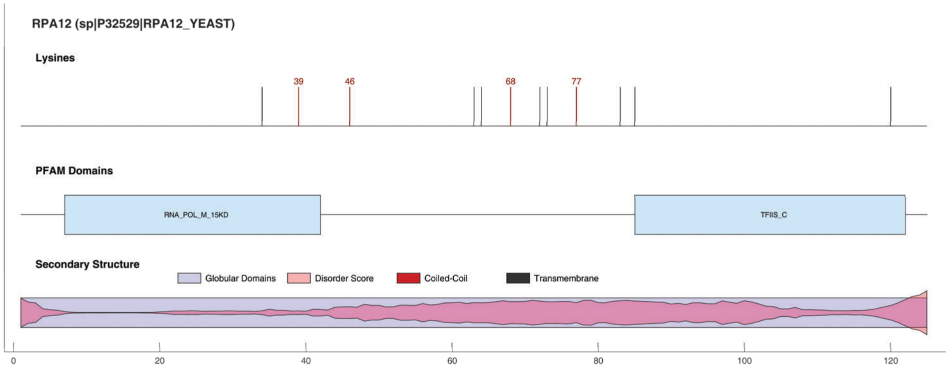
RPA12 (sp|P32529|RPA12_YEAST)
Lysines
39
46
68
77
PFAM Domains
RNA_POL_M_15KD
TFIIS_C
Secondary Structure
Globular Domains
Disorder Score
Coiled-Coil
Transmembrane
0
20
40
60
80
100
120

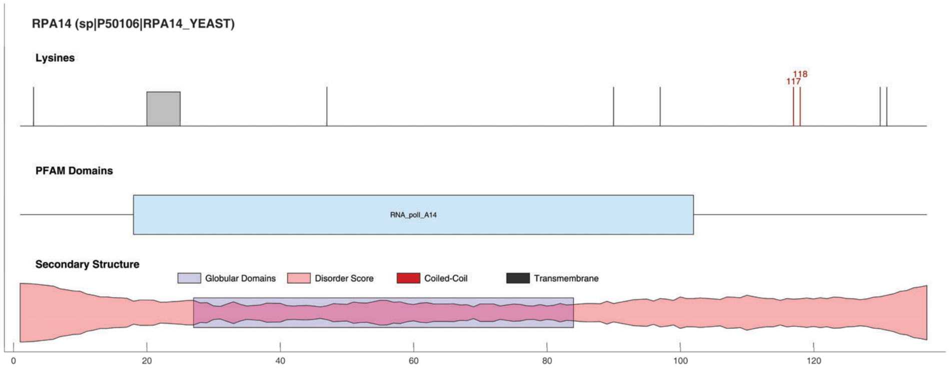
RPA14 (sp|P50106|RPA14_YEAST)
Lysines
117
118
PFAM Domains
RNA_pol_A14
Secondary Structure
Globular Domains
Disorder Score
Coiled-Coil
Transmembrane
0
20
40
60
80
100
120

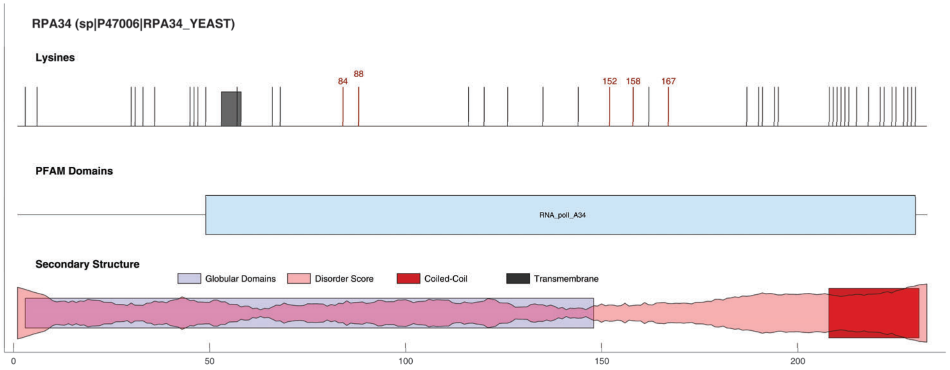
RPA34 (sp|P47006|RPA34_YEAST)
Lysines
84
88
152
158
167
PFAM Domains
RNA_pol_A34
Secondary Structure
Globular Domains
Disorder Score
Coiled-Coil
Transmembrane
0
50
100
150
200

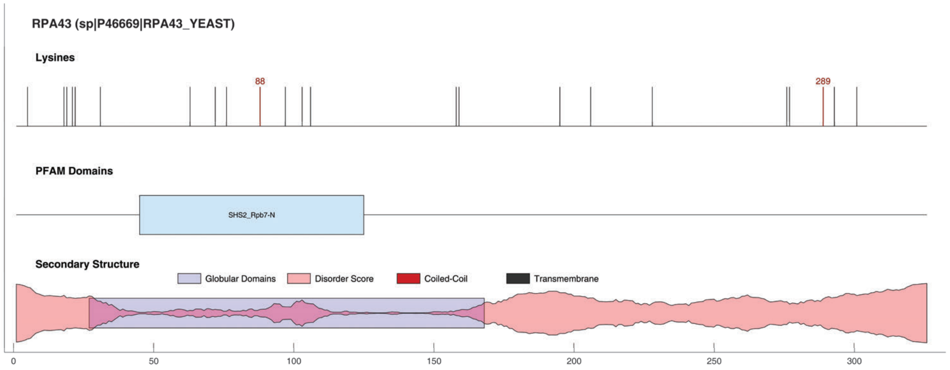
RPA43 (sp|P46669|RPA43_YEAST)
Lysines
88
289
PFAM Domains
SHS2_Rpb7-N
Secondary Structure
Globular Domains
Disorder Score
Coiled-Coil
Transmembrane
0
50
100
150
200
250
300

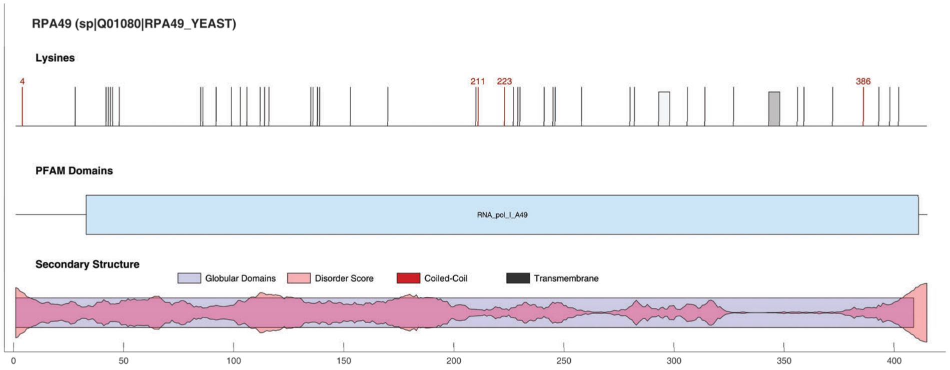

RPA49 (sp|Q01080|RPA49_YEAST)
Lysines
4
211
223
386
PFAM Domains
RNA_pol_I_A49
Secondary Structure
Globular Domains
Disorder Score
Coiled-Coil
Transmembrane
0
50
100
150
200
250
300
350
400

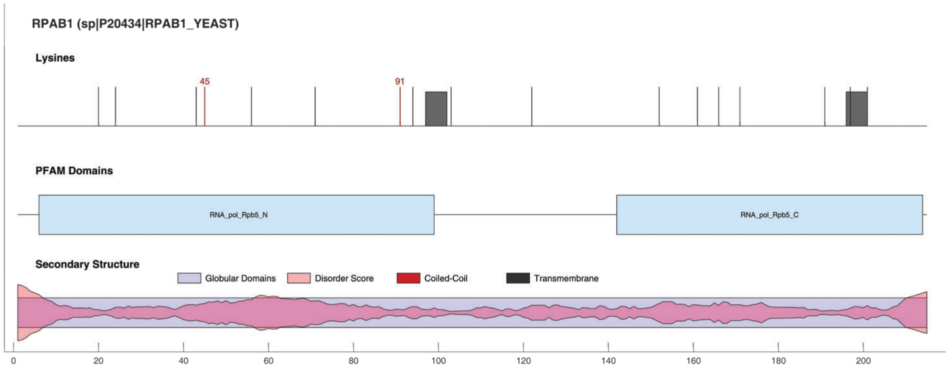

RPAB1 (sp|P20434|RPAB1_YEAST)
Lysines
45
91
PFAM Domains
RNA_pol_Rpb5_N
RNA_pol_Rpb5_C
Secondary Structure
Globular Domains
Disorder Score
Coiled-Coil
Transmembrane
0
20
40
60
80
100
120
140
160
180
200

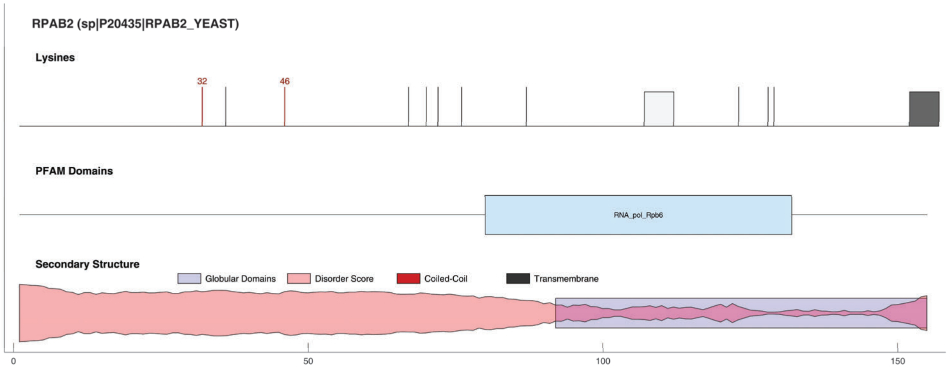

RPAB2 (sp|P20435|RPAB2_YEAST)
Lysines
32
46
PFAM Domains
RNA_pol_Rpb6
Secondary Structure
Globular Domains
Disorder Score
Coiled-Coil
Transmembrane
0
50
100
150

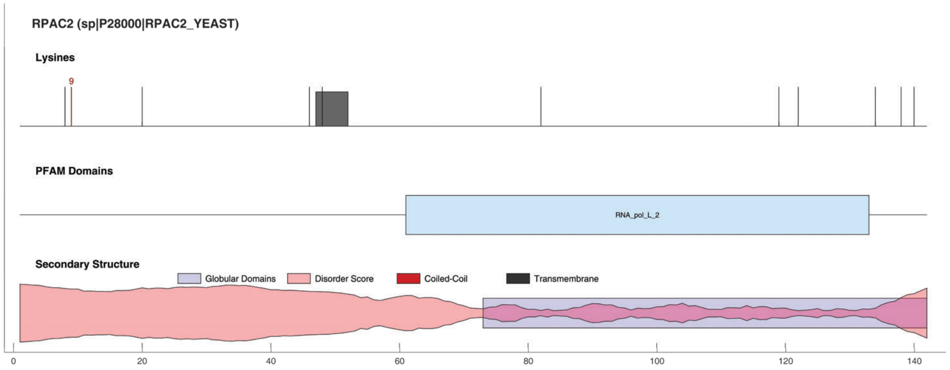

RPAC2 (sp|P28000|RPAC2_YEAST)
Lysines
9
PFAM Domains
RNA_pol_L_2
Secondary Structure
Globular Domains
Disorder Score
Coiled-Coil
Transmembrane
0
20
40
60
80
100
120
140

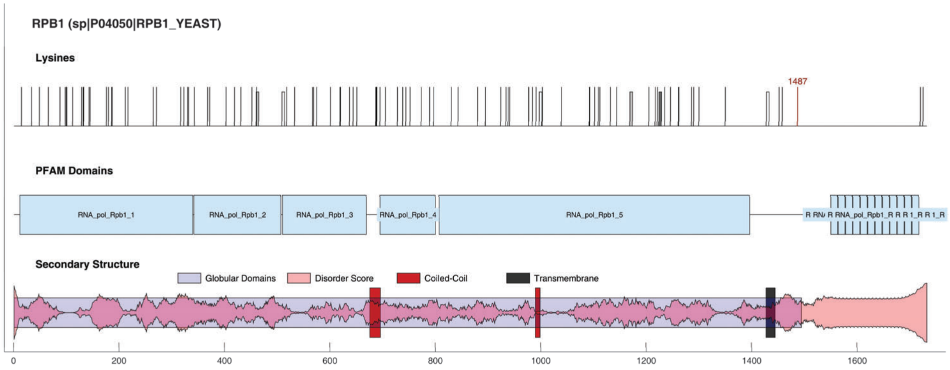
RPB1 (sp|P04050|RPB1_YEAST)
Lysines
1487
PFAM Domains
RNA_pol_Rpb1_1
RNA_pol_Rpb1_2
RNA_pol_Rpb1_3
RNA_pol_Rpb1_4
RNA_pol_Rpb1_5
Secondary Structure
Globular Domains
Disorder Score
Coiled-Coil
Transmembrane
0 200 400 600 800 1000 1200 1400 1600

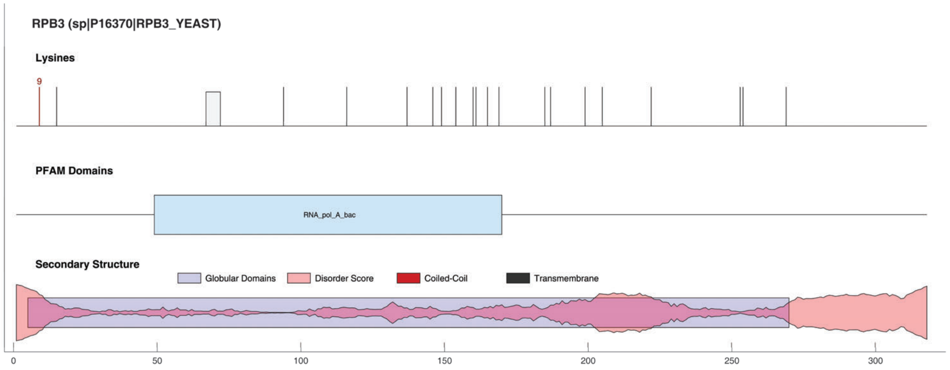
RPB3 (sp|P16370|RPB3_YEAST)
Lysines
9
PFAM Domains
RNA_pol_A_bac
Secondary Structure
Globular Domains
Disorder Score
Coiled-Coil
Transmembrane
0 50 100 150 200 250 300

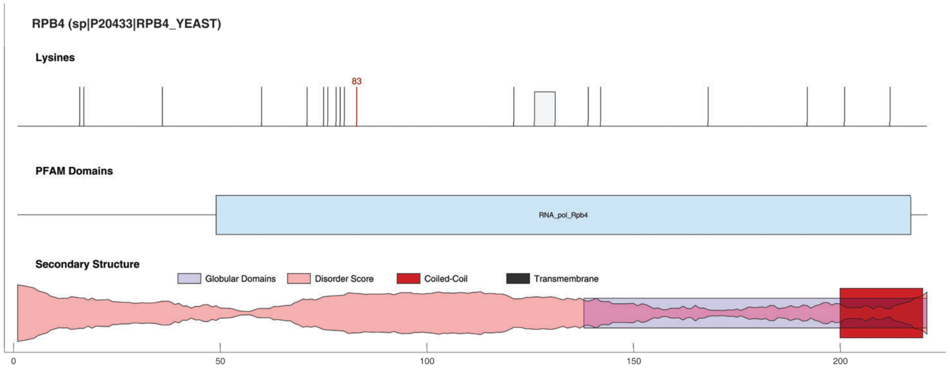
RPB4 (sp|P20433|RPB4_YEAST)
Lysines
83
PFAM Domains
RNA_pol_Rpb4
Secondary Structure
Globular Domains
Disorder Score
Coiled-Coil
Transmembrane
0 50 100 150 200

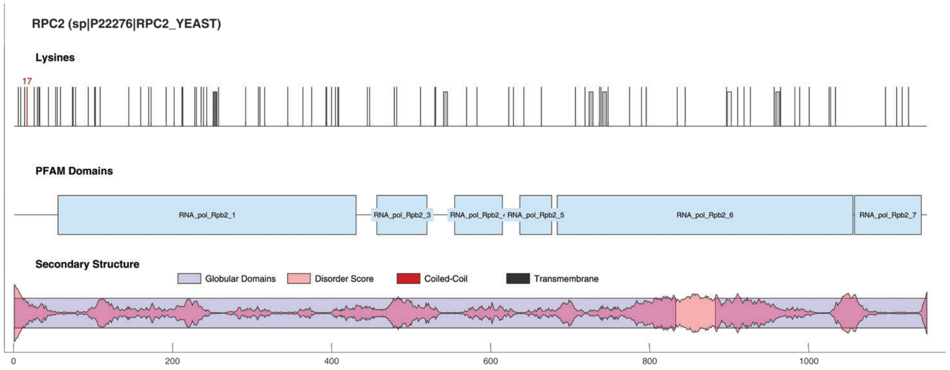
RPC2 (sp|P22276|RPC2_YEAST)
Lysines
17
PFAM Domains
RNA_pol_Rpb2_1
RNA_pol_Rpb2_3
RNA_pol_Rpb2_5
RNA_pol_Rpb2_6
RNA_pol_Rpb2_7
Secondary Structure
Globular Domains
Disorder Score
Coiled-Coil
Transmembrane
0 200 400 600 800 1000

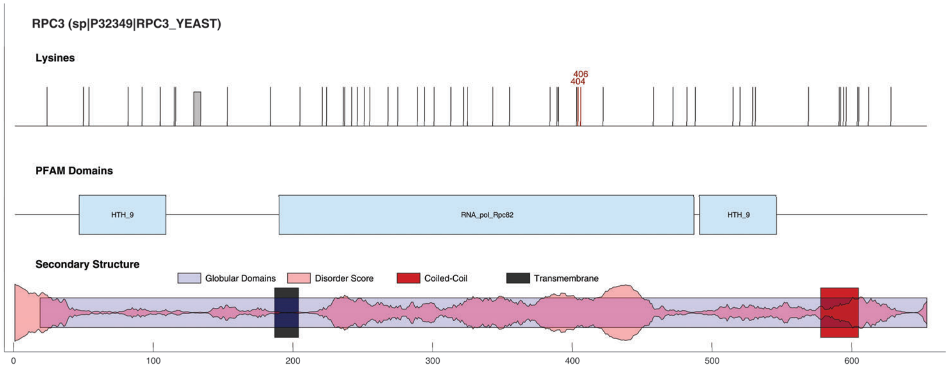
RPC3 (sp|P32349|RPC3_YEAST)
Lysines
406
404
PFAM Domains
HTH_9
RNA_pol_Rpc82
HTH_9
Secondary Structure
Globular Domains
Disorder Score
Coiled-Coil
Transmembrane
0
100
200
300
400
500
600

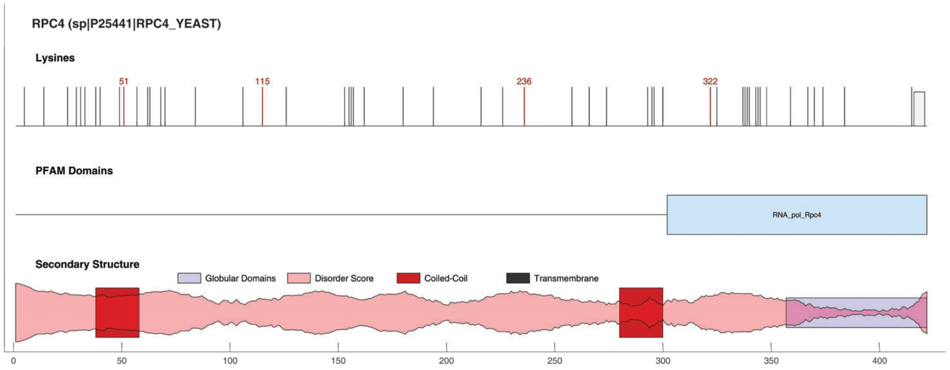
RPC4 (sp|P25441|RPC4_YEAST)
Lysines
51
115
236
322
PFAM Domains
RNA_pol_Rpc4
Secondary Structure
Globular Domains
Disorder Score
Coiled-Coil
Transmembrane
0
50
100
150
200
250
300
350
400

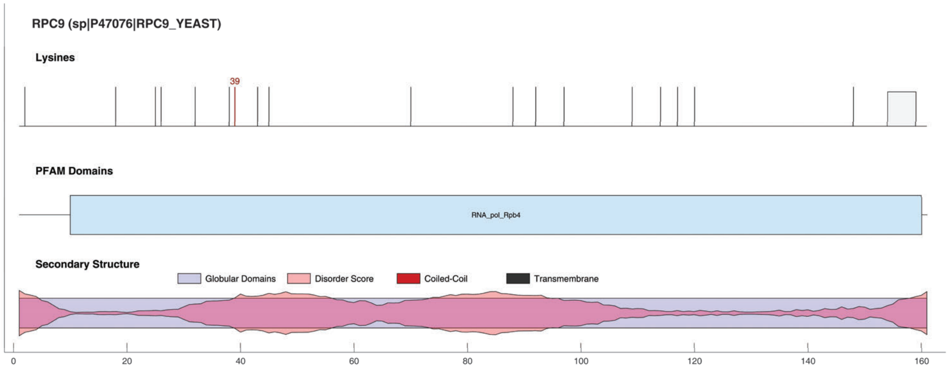
RPC9 (sp|P47076|RPC9_YEAST)
Lysines
39
PFAM Domains
RNA_pol_Rpb4
Secondary Structure
Globular Domains
Disorder Score
Coiled-Coil
Transmembrane
0
20
40
60
80
100
120
140
160

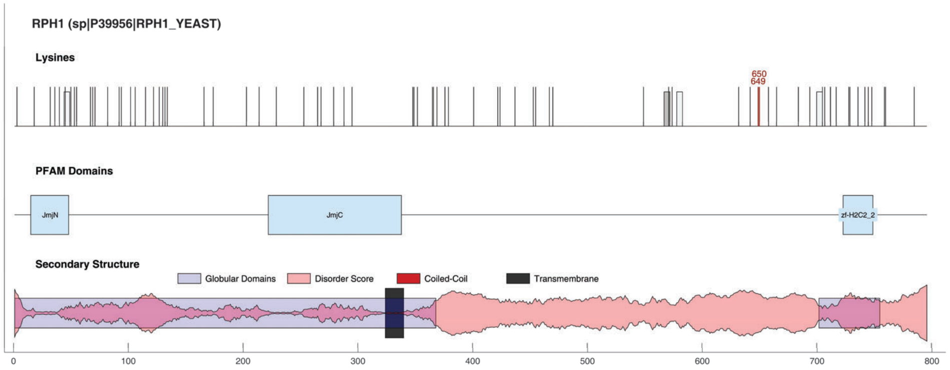
RPH1 (sp|P39956|RPH1_YEAST)
Lysines
650
649
PFAM Domains
JmjN
JmjC
zf-H2C2_2
Secondary Structure
Globular Domains
Disorder Score
Coiled-Coil
Transmembrane
0
100
200
300
400
500
600
700
800

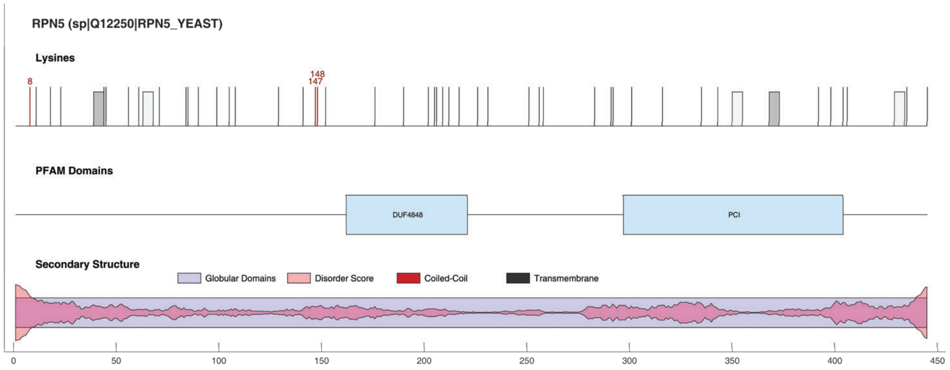
RPN5 (sp|Q12250|RPN5_YEAST)
Lysines
8
148
147
PFAM Domains
DUF4848
PCI
Secondary Structure
Globular Domains
Disorder Score
Coiled-Coil
Transmembrane
0
50
100
150
200
250
300
350
400
450

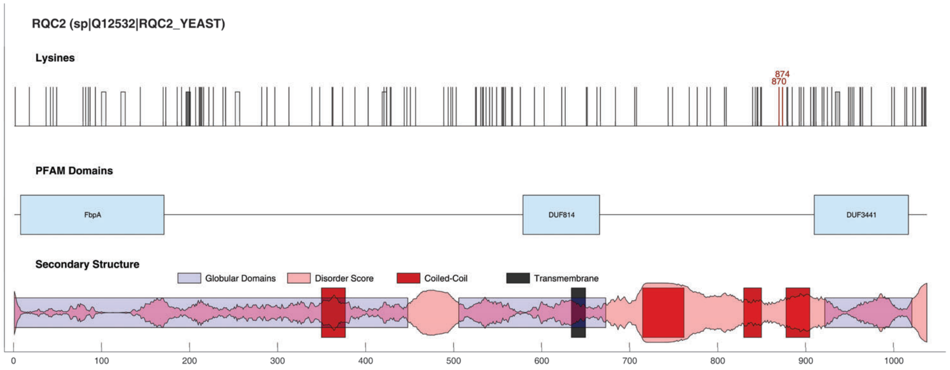
RQC2 (sp|Q12532|RQC2_YEAST)
Lysines
874
870
PFAM Domains
FbpA
DUF814
DUF3441
Secondary Structure
Globular Domains
Disorder Score
Coiled-Coil
Transmembrane
0
100
200
300
400
500
600
700
800
900
1000

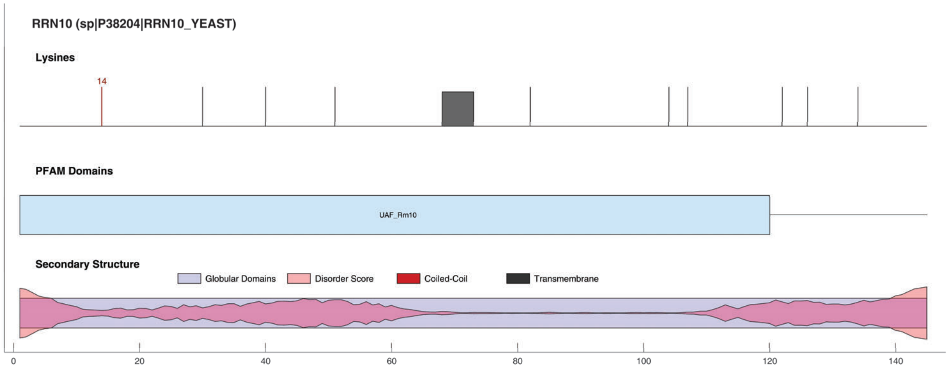
RRN10 (sp|P38204|RRN10_YEAST)
Lysines
14
PFAM Domains
UAF_Rrn10
Secondary Structure
Globular Domains
Disorder Score
Coiled-Coil
Transmembrane
0
20
40
60
80
100
120
140

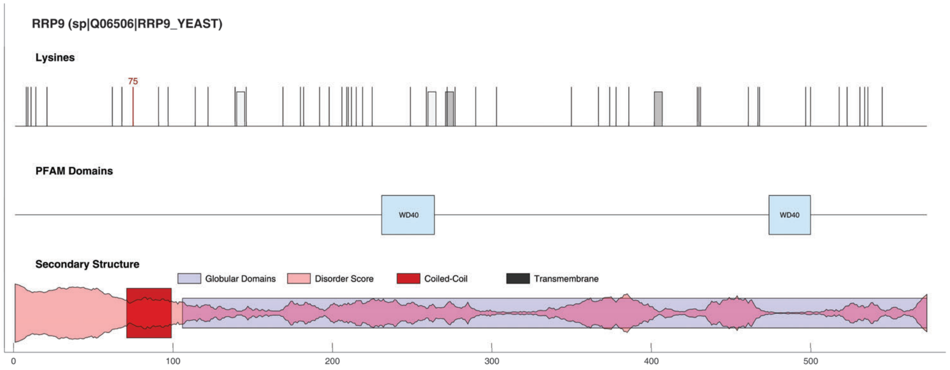
RRP9 (sp|Q06506|RRP9_YEAST)
Lysines
75
PFAM Domains
WD40
WD40
Secondary Structure
Globular Domains
Disorder Score
Coiled-Coil
Transmembrane
0
100
200
300
400
500

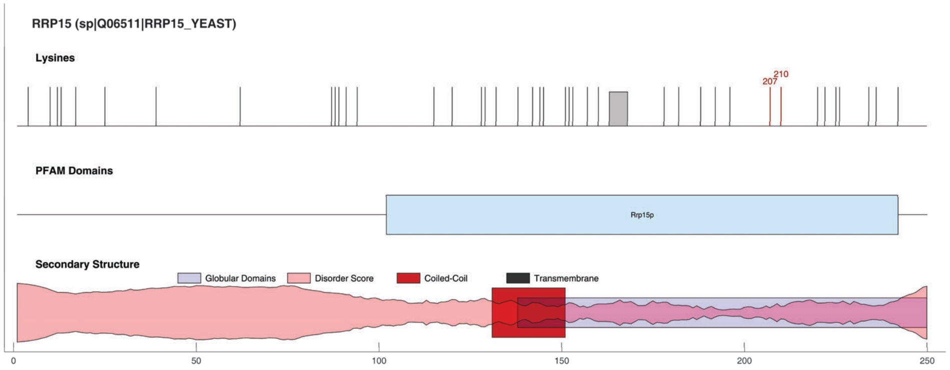
RRP15 (sp|Q06511|RRP15_YEAST)
Lysines
207
210
PFAM Domains
Rrp15p
Secondary Structure
Globular Domains
Disorder Score
Coiled-Coil
Transmembrane
0
50
100
150
200
250

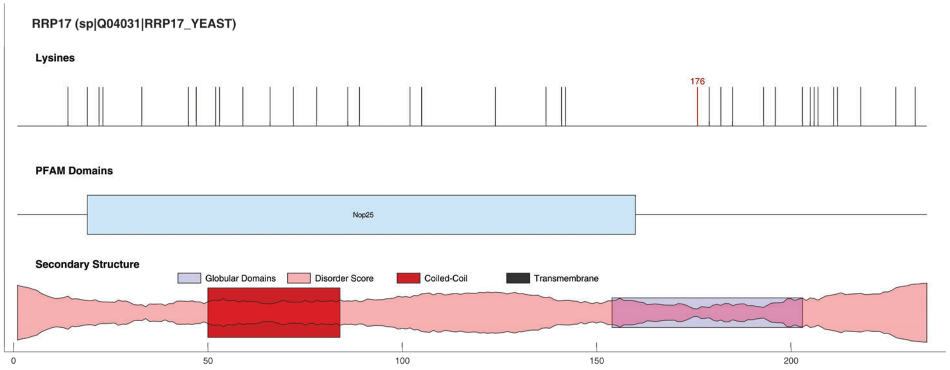
RRP17 (sp|Q04031|RRP17_YEAST)
Lysines
176
PFAM Domains
Nop25
Secondary Structure
Globular Domains
Disorder Score
Coiled-Coil
Transmembrane
0
50
100
150
200

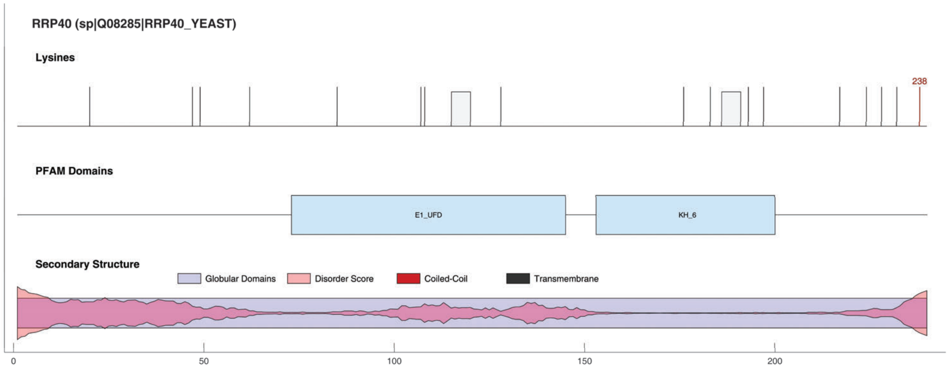
RRP40 (sp|Q08285|RRP40_YEAST)
Lysines
238
PFAM Domains
E1_UFD
KH_6
Secondary Structure
Globular Domains
Disorder Score
Coiled-Coil
Transmembrane
0
50
100
150
200

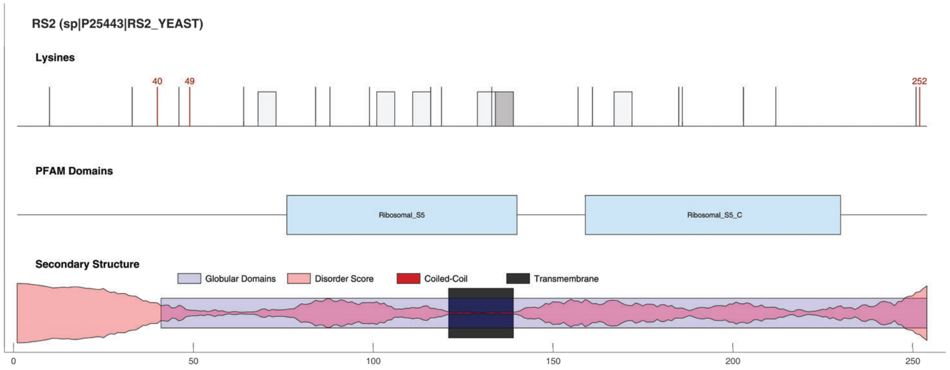
RS2 (sp|P25443|RS2_YEAST)
Lysines
40
49
252
PFAM Domains
Ribosomal_S5
Ribosomal_S5_C
Secondary Structure
Globular Domains
Disorder Score
Coiled-Coil
Transmembrane
0
50
100
150
200
250

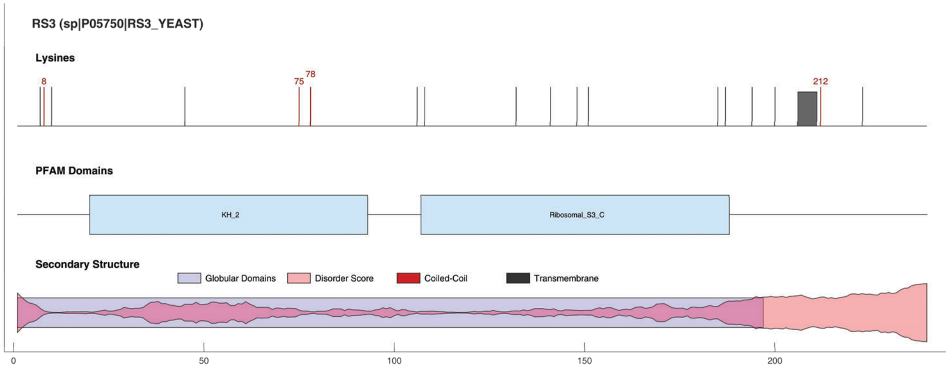
RS3 (sp|P05750|RS3_YEAST)
Lysines
8
75
78
212
PFAM Domains
KH_2
Ribosomal_S3_C
Secondary Structure
Globular Domains
Disorder Score
Coiled-Coil
Transmembrane
0
50
100
150
200

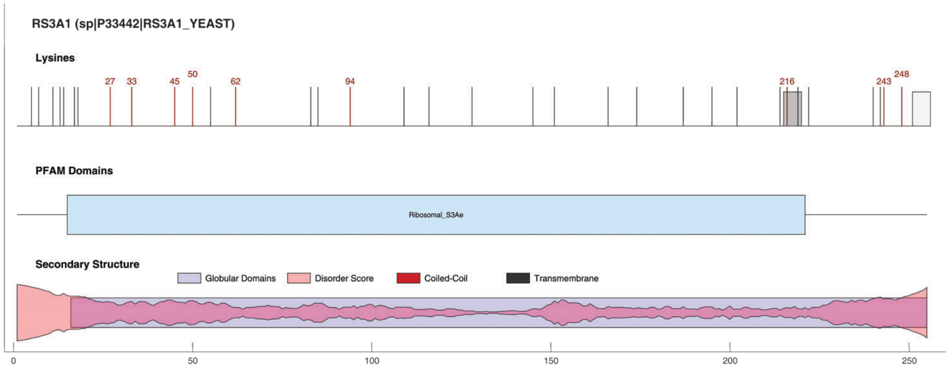
RS3A1 (sp|P33442|RS3A1_YEAST)
Lysines
27
33
45
50
62
94
216
243
248
PFAM Domains
Ribosomal_S3Ae
Secondary Structure
Globular Domains
Disorder Score
Coiled-Coil
Transmembrane
0
50
100
150
200
250

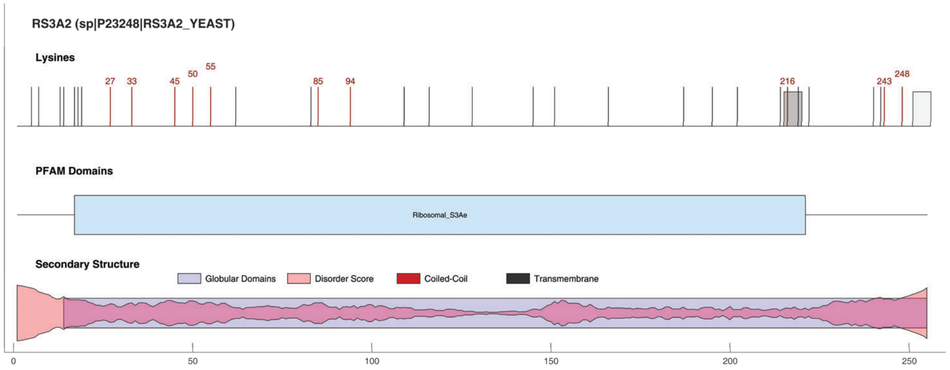
RS3A2 (sp|P23248|RS3A2_YEAST)
Lysines
27
33
45
50
55
85
94
216
243
248
PFAM Domains
Ribosomal_S3Ae
Secondary Structure
Globular Domains
Disorder Score
Coiled-Coil
Transmembrane
0
50
100
150
200
250

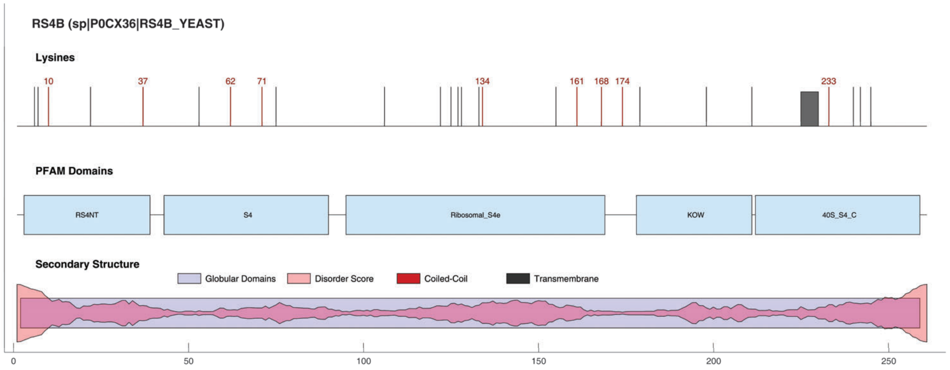
RS4B (sp|P0CX36|RS4B_YEAST)
Lysines
10
37
62
71
134
161
168
174
233
PFAM Domains
RS4NT
S4
Ribosomal_S4e
KOW
40S_S4_C
Secondary Structure
Globular Domains
Disorder Score
Coiled-Coil
Transmembrane
0
50
100
150
200
250

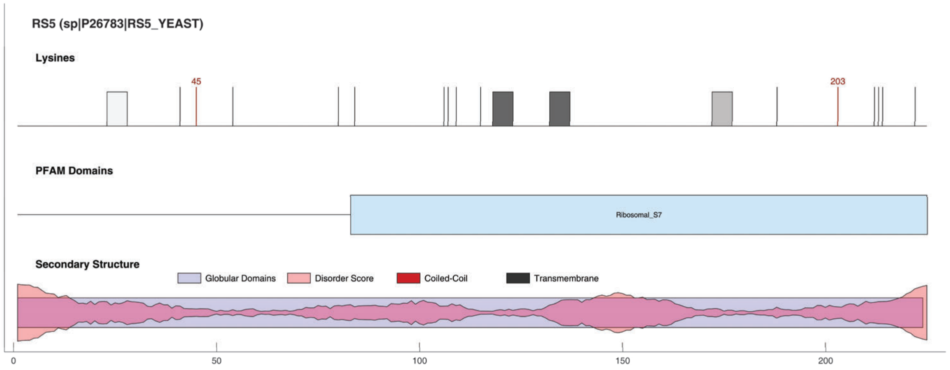
RS5 (sp|P26783|RS5_YEAST)
Lysines
45
203
PFAM Domains
Ribosomal_S7
Secondary Structure
Globular Domains
Disorder Score
Coiled-Coil
Transmembrane
0
50
100
150
200

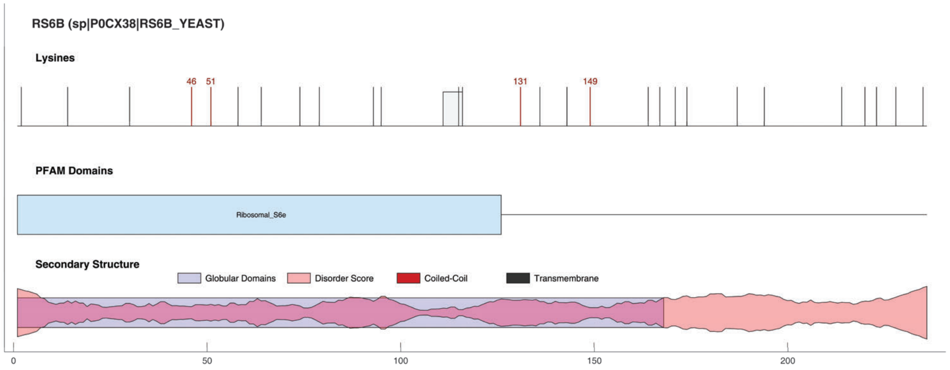
RS6B (sp|P0CX38|RS6B_YEAST)
Lysines
46
51
131
149
PFAM Domains
Ribosomal_S6e
Secondary Structure
Globular Domains
Disorder Score
Coiled-Coil
Transmembrane
0
50
100
150
200

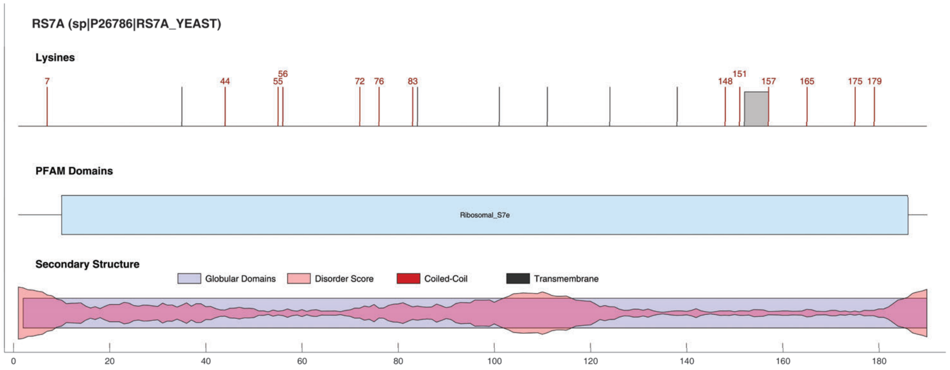
RS7A (sp|P26786|RS7A_YEAST)
Lysines
7
44
55
56
72
76
83
148
151
157
165
175
179
PFAM Domains
Ribosomal_S7e
Secondary Structure
Globular Domains
Disorder Score
Coiled-Coil
Transmembrane
0
20
40
60
80
100
120
140
160
180

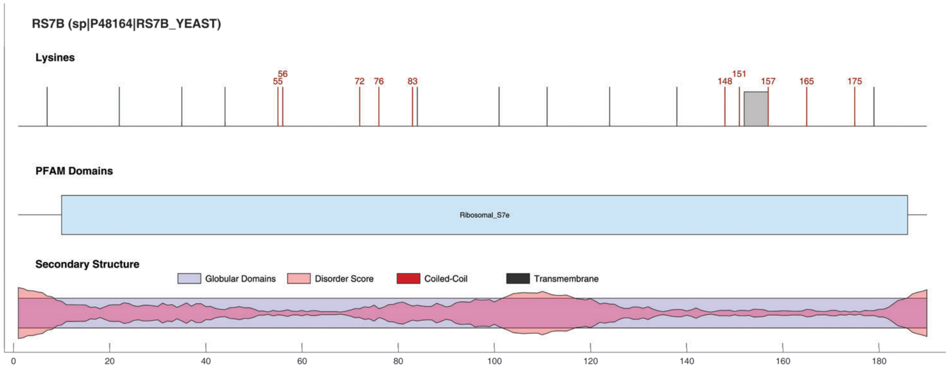
RS7B (sp|P48164|RS7B_YEAST)
Lysines
55
56
72
76
83
148
151
157
165
175
PFAM Domains
Ribosomal_S7e
Secondary Structure
Globular Domains
Disorder Score
Coiled-Coil
Transmembrane
0
20
40
60
80
100
120
140
160
180

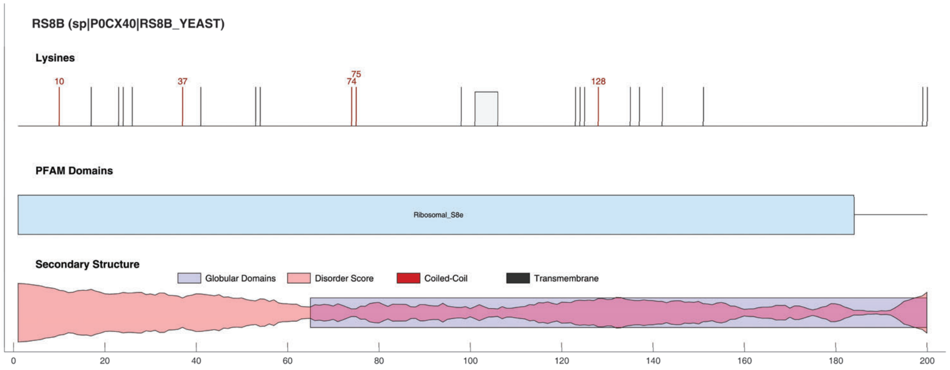
RS8B (sp|P0CX40|RS8B_YEAST)
Lysines
10
37
75
74
128
PFAM Domains
Ribosomal_S8e
Secondary Structure
Globular Domains
Disorder Score
Coiled-Coil
Transmembrane
0
20
40
60
80
100
120
140
160
180
200

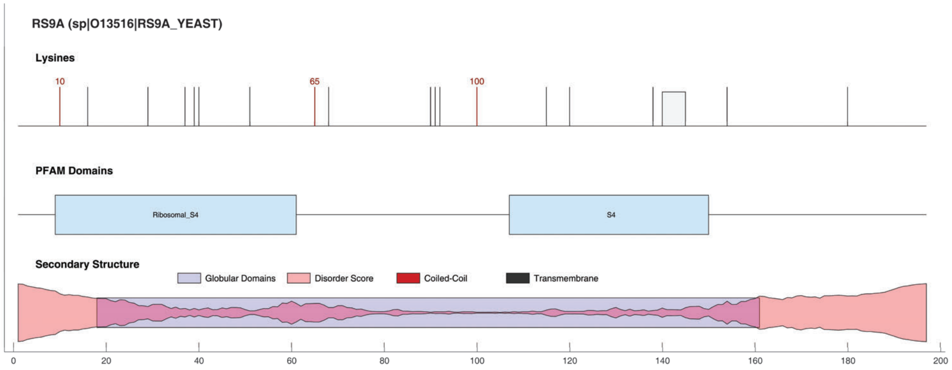
RS9A (sp|O13516|RS9A_YEAST)
Lysines
10
65
100
PFAM Domains
Ribosomal_S4
S4
Secondary Structure
Globular Domains
Disorder Score
Coiled-Coil
Transmembrane
0
20
40
60
80
100
120
140
160
180
200

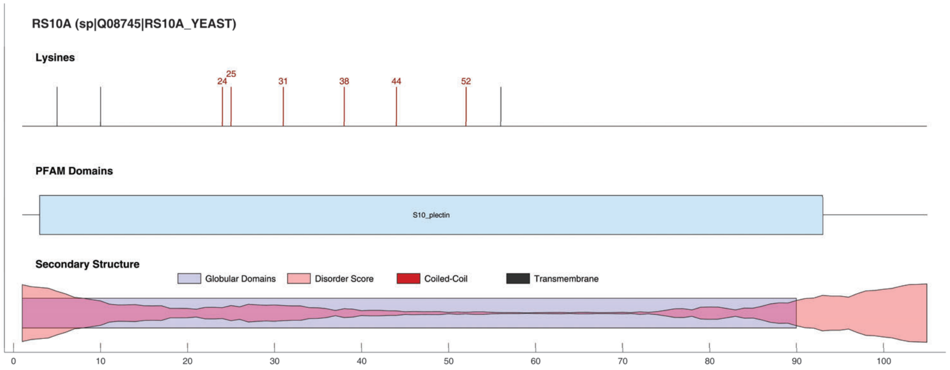
RS10A (sp|Q08745|RS10A_YEAST)
Lysines
24
25
31
38
44
52
PFAM Domains
S10_plectin
Secondary Structure
Globular Domains
Disorder Score
Coiled-Coil
Transmembrane
0
10
20
30
40
50
60
70
80
90
100

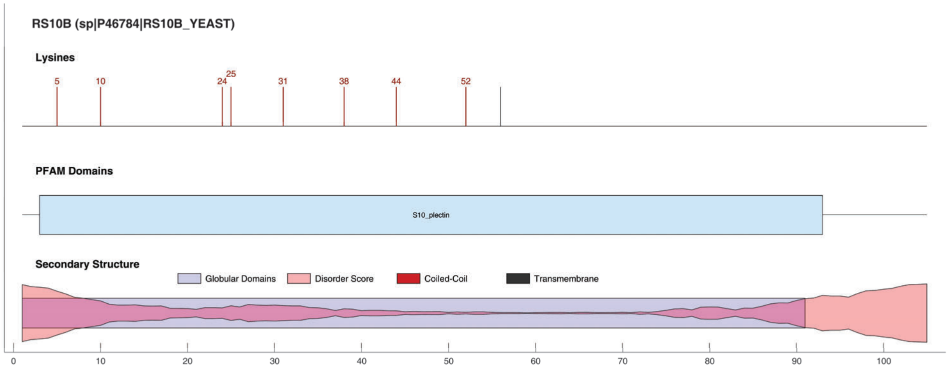
RS10B (sp|P46784|RS10B_YEAST)
Lysines
5
10
24
25
31
38
44
52
PFAM Domains
S10_plectin
Secondary Structure
Globular Domains
Disorder Score
Coiled-Coil
Transmembrane
0
10
20
30
40
50
60
70
80
90
100

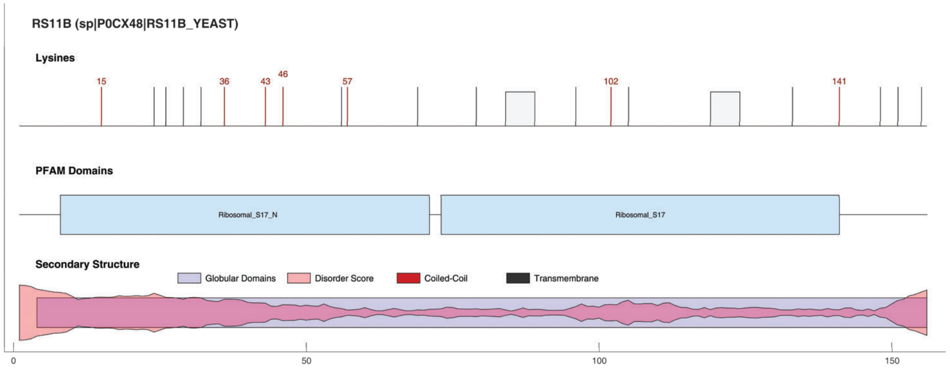
RS11B (sp|P0CX48|RS11B_YEAST)
Lysines
15
36
43
46
57
102
141
PFAM Domains
Ribosomal_S17_N
Ribosomal_S17
Secondary Structure
Globular Domains
Disorder Score
Coiled-Coil
Transmembrane
0
50
100
150

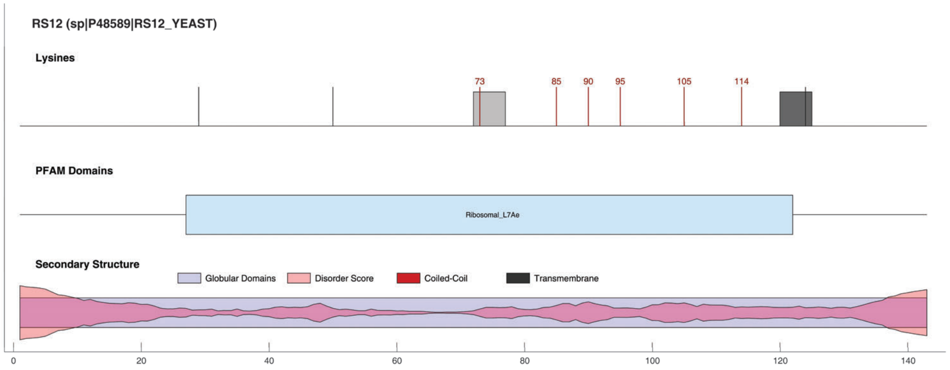
RS12 (sp|P48589|RS12_YEAST)
Lysines
73
85
90
95
105
114
PFAM Domains
Ribosomal_L7Ae
Secondary Structure
Globular Domains
Disorder Score
Coiled-Coil
Transmembrane
0
20
40
60
80
100
120
140

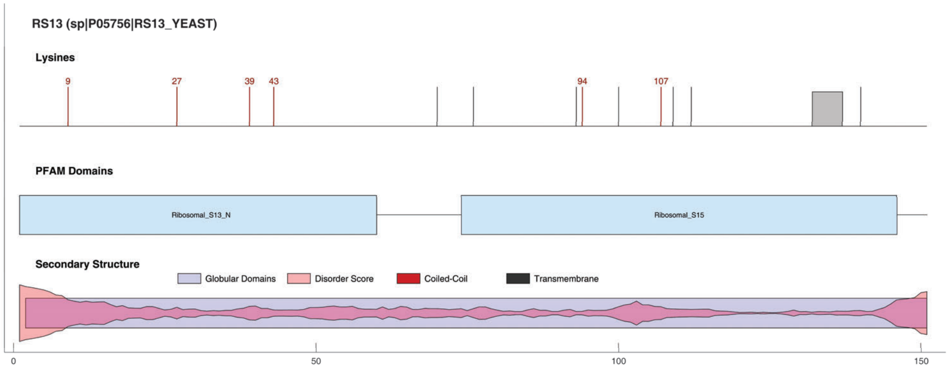
RS13 (sp|P05756|RS13_YEAST)
Lysines
9
27
39
43
94
107
PFAM Domains
Ribosomal_S13_N
Ribosomal_S15
Secondary Structure
Globular Domains
Disorder Score
Coiled-Coil
Transmembrane
0
50
100
150

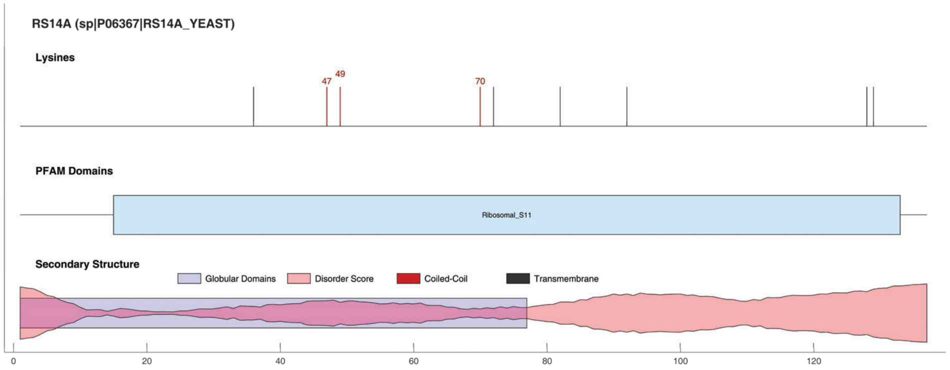
RS14A (sp|P06367|RS14A_YEAST)
Lysines
47
49
70
PFAM Domains
Ribosomal_S11
Secondary Structure
Globular Domains
Disorder Score
Coiled-Coil
Transmembrane
0
20
40
60
80
100
120

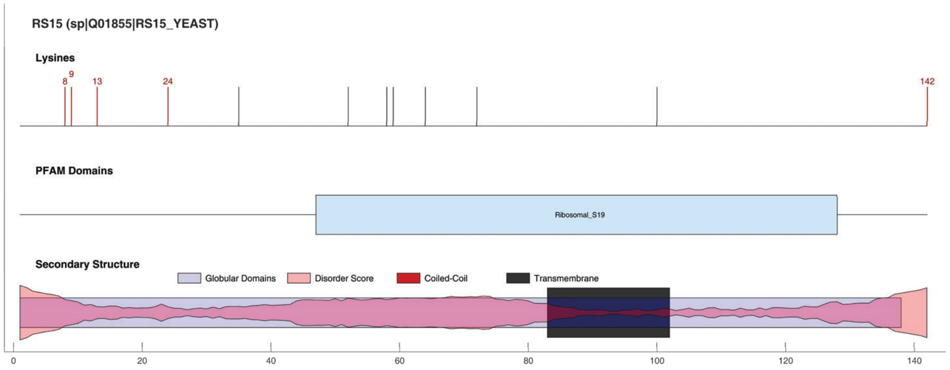
RS15 (sp|Q01855|RS15_YEAST)
Lysines
8
9
13
24
142
PFAM Domains
Ribosomal_S19
Secondary Structure
Globular Domains
Disorder Score
Coiled-Coil
Transmembrane
0
20
40
60
80
100
120
140

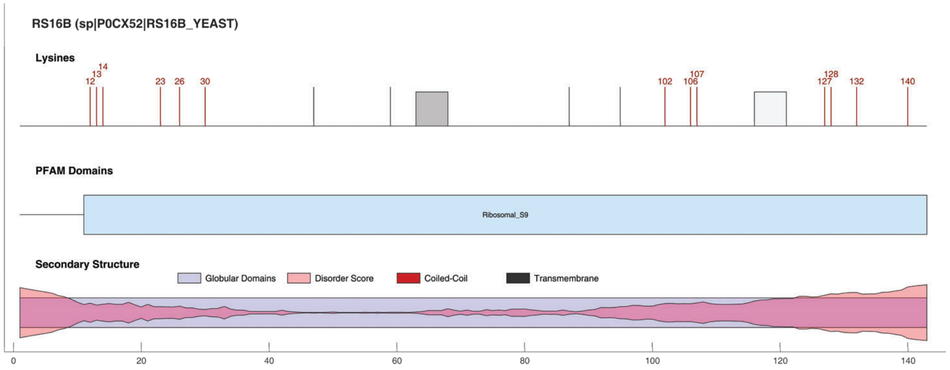
RS16B (sp|P0CX52|RS16B_YEAST)
Lysines
12
13
14
23
26
30
102
106
107
127
128
132
140
PFAM Domains
Ribosomal_S9
Secondary Structure
Globular Domains
Disorder Score
Coiled-Coil
Transmembrane
0
20
40
60
80
100
120
140

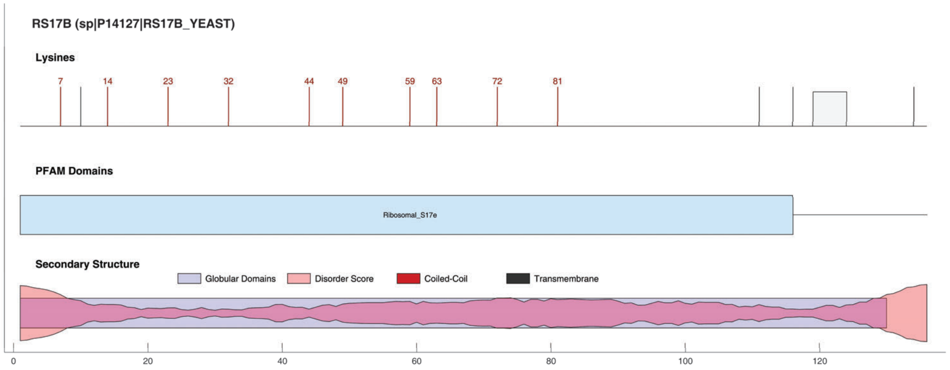
RS17B (sp|P14127|RS17B_YEAST)
Lysines
7
14
23
32
44
49
59
63
72
81
PFAM Domains
Ribosomal_S17e
Secondary Structure
Globular Domains
Disorder Score
Coiled-Coil
Transmembrane
0
20
40
60
80
100
120

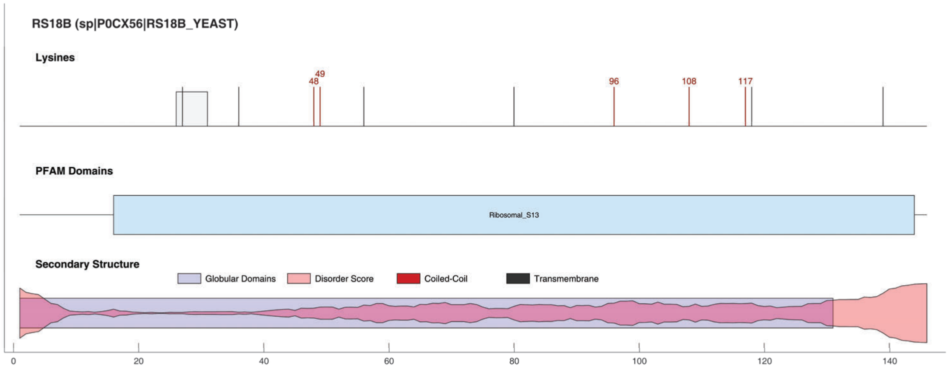
RS18B (sp|P0CX56|RS18B_YEAST)
Lysines
48
49
96
108
117
PFAM Domains
Ribosomal_S13
Secondary Structure
Globular Domains
Disorder Score
Coiled-Coil
Transmembrane
0
20
40
60
80
100
120
140

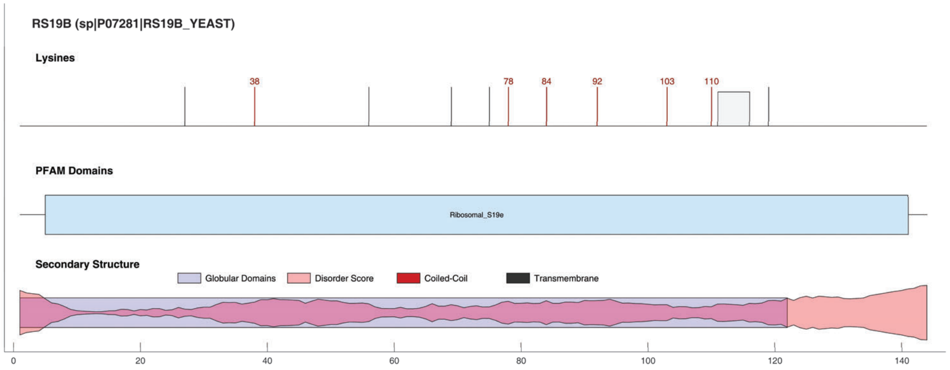
RS19B (sp|P07281|RS19B_YEAST)
Lysines
38
78
84
92
103
110
PFAM Domains
Ribosomal_S19e
Secondary Structure
Globular Domains
Disorder Score
Coiled-Coil
Transmembrane
0
20
40
60
80
100
120
140

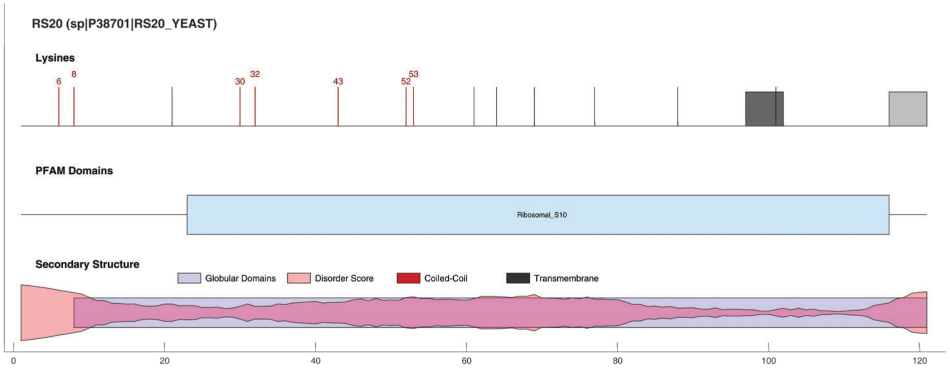
RS20 (sp|P38701|RS20_YEAST)
Lysines
6
8
30
32
43
52
53
PFAM Domains
Ribosomal_S10
Secondary Structure
Globular Domains
Disorder Score
Coiled-Coil
Transmembrane
0
20
40
60
80
100
120

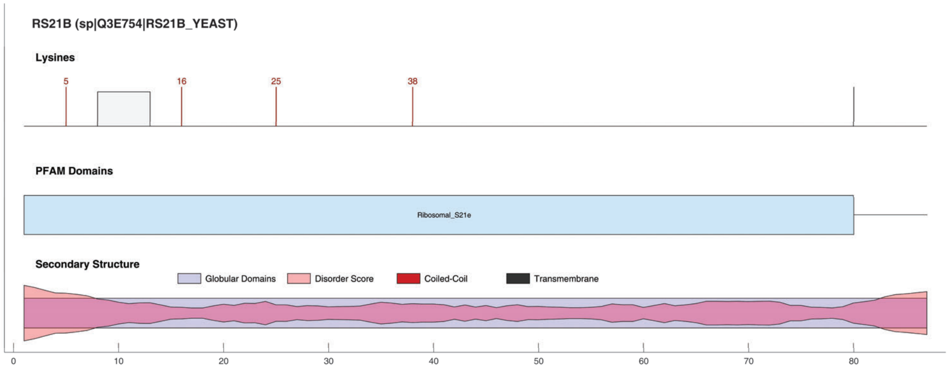
RS21B (sp|Q3E754|RS21B_YEAST)
Lysines
5
16
25
38
PFAM Domains
Ribosomal_S21e
Secondary Structure
Globular Domains
Disorder Score
Coiled-Coil
Transmembrane
0
10
20
30
40
50
60
70
80

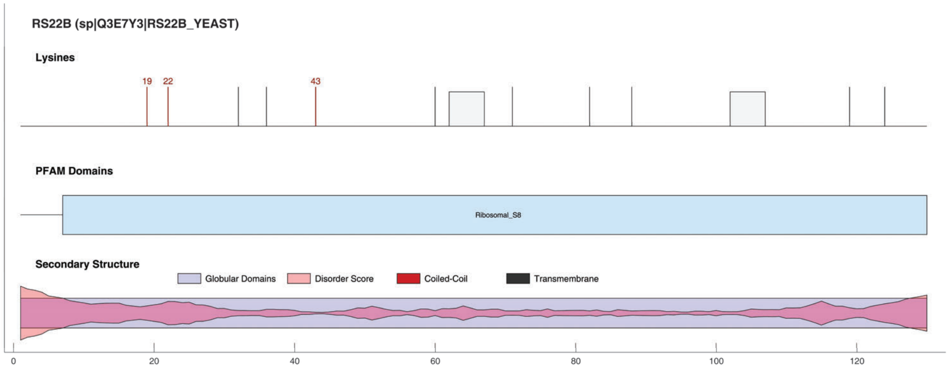
RS22B (sp|Q3E7Y3|RS22B_YEAST)
Lysines
19
22
43
PFAM Domains
Ribosomal_S8
Secondary Structure
Globular Domains
Disorder Score
Coiled-Coil
Transmembrane
0
20
40
60
80
100
120

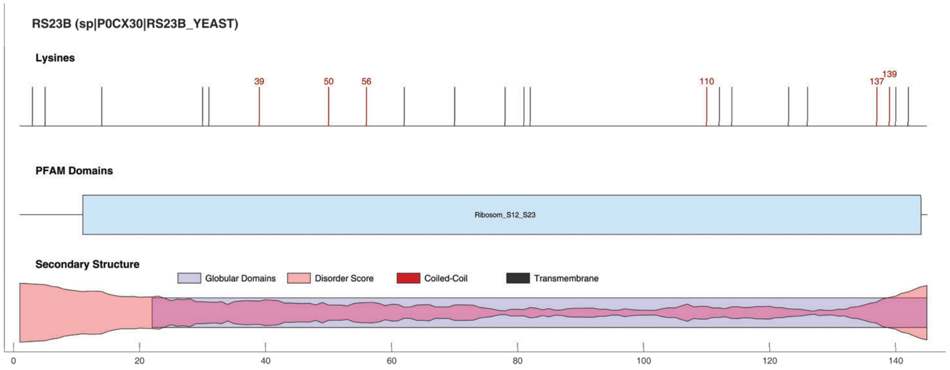
RS23B (sp|P0CX30|RS23B_YEAST)
Lysines
39 50 56 110 137 139
PFAM Domains
Ribosom_S12_S23
Secondary Structure
Globular Domains
Disorder Score
Coiled-Coil
Transmembrane
0 20 40 60 80 100 120 140

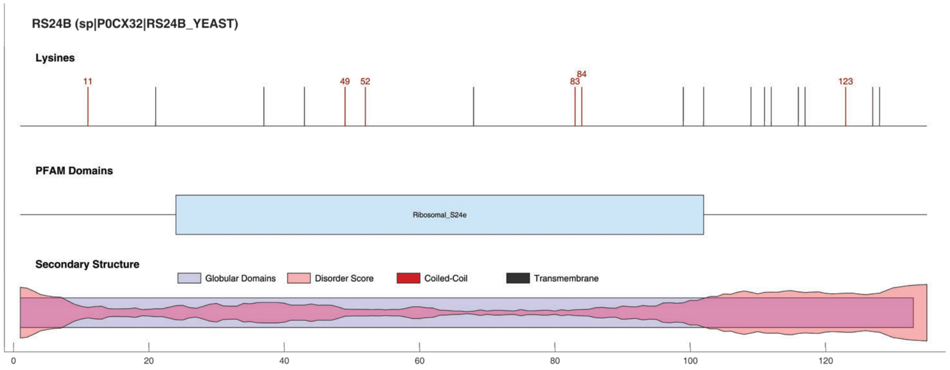
RS24B (sp|P0CX32|RS24B_YEAST)
Lysines
11 49 52 83 84 123
PFAM Domains
Ribosomal_S24e
Secondary Structure
Globular Domains
Disorder Score
Coiled-Coil
Transmembrane
0 20 40 60 80 100 120

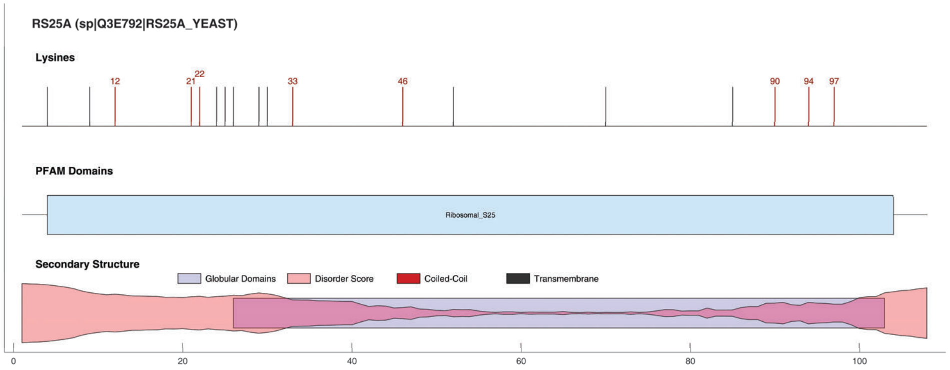
RS25A (sp|Q3E792|RS25A_YEAST)
Lysines
12 21 22 33 46 90 94 97
PFAM Domains
Ribosomal_S25
Secondary Structure
Globular Domains
Disorder Score
Coiled-Coil
Transmembrane
0 20 40 60 80 100

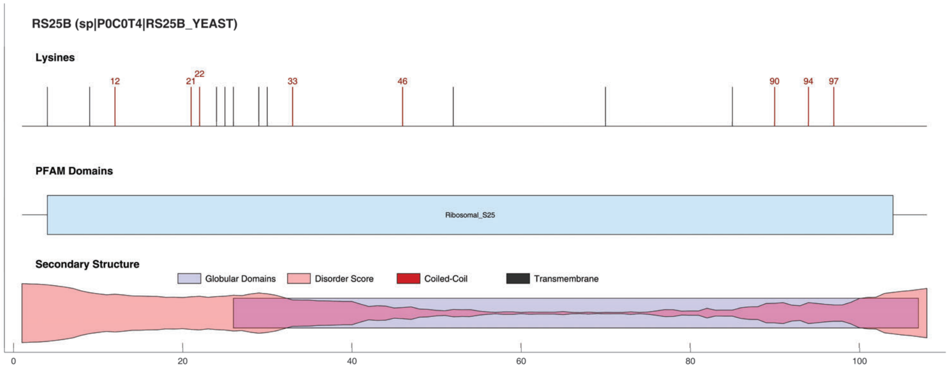
RS25B (sp|P0C0T4|RS25B_YEAST)
Lysines
12 21 22 33 46 90 94 97
PFAM Domains
Ribosomal_S25
Secondary Structure
Globular Domains
Disorder Score
Coiled-Coil
Transmembrane
0 20 40 60 80 100

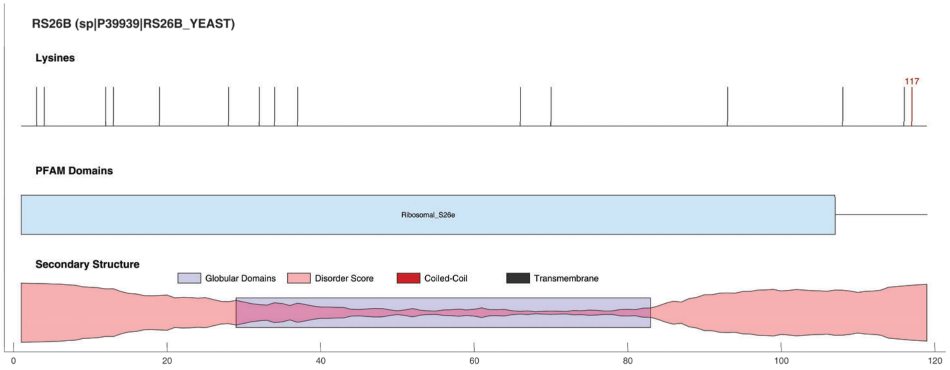
RS26B (sp|P39939|RS26B_YEAST)
Lysines
117
PFAM Domains
Ribosomal_S26e
Secondary Structure
Globular Domains
Disorder Score
Coiled-Coil
Transmembrane
0
20
40
60
80
100
120

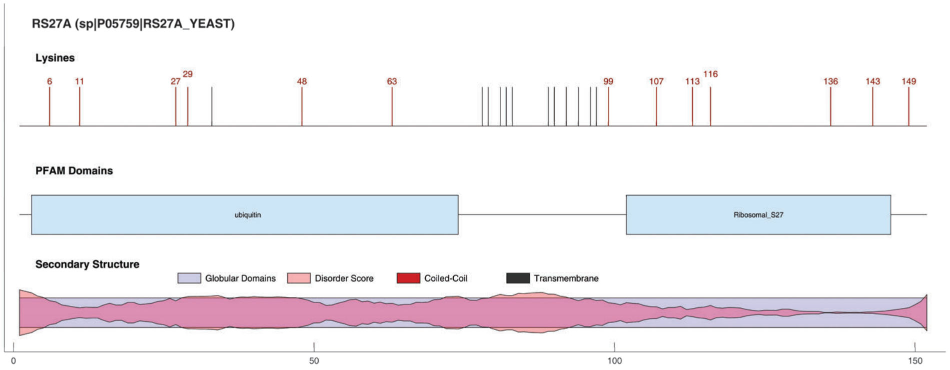
RS27A (sp|P05759|RS27A_YEAST)
Lysines
6
11
27
29
48
63
99
107
113
116
136
143
149
PFAM Domains
ubiquitin
Ribosomal_S27
Secondary Structure
Globular Domains
Disorder Score
Coiled-Coil
Transmembrane
0
50
100
150

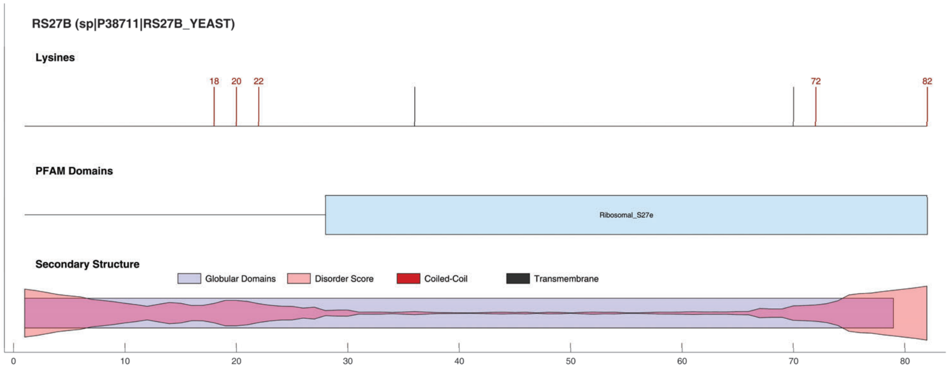
RS27B (sp|P38711|RS27B_YEAST)
Lysines
18
20
22
72
82
PFAM Domains
Ribosomal_S27e
Secondary Structure
Globular Domains
Disorder Score
Coiled-Coil
Transmembrane
0
10
20
30
40
50
60
70
80

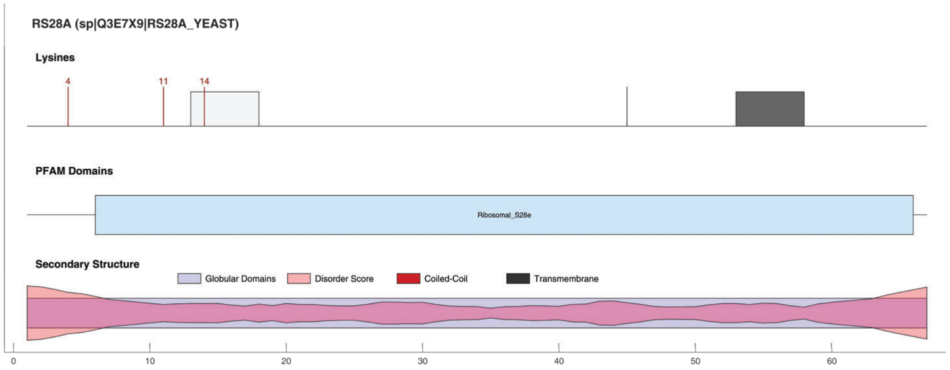
RS28A (sp|Q3E7X9|RS28A_YEAST)
Lysines
4
11
14
PFAM Domains
Ribosomal_S28e
Secondary Structure
Globular Domains
Disorder Score
Coiled-Coil
Transmembrane
0
10
20
30
40
50
60

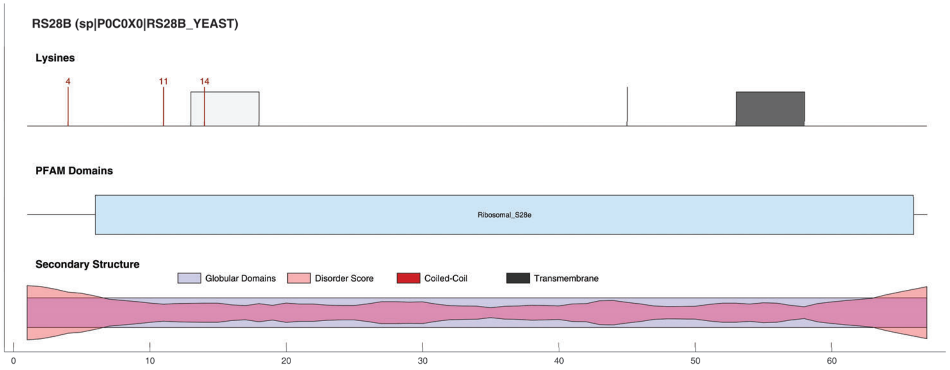
RS28B (sp|P0C0X0|RS28B_YEAST)
Lysines
4
11
14
PFAM Domains
Ribosomal_S28e
Secondary Structure
Globular Domains
Disorder Score
Coiled-Coil
Transmembrane
0
10
20
30
40
50
60

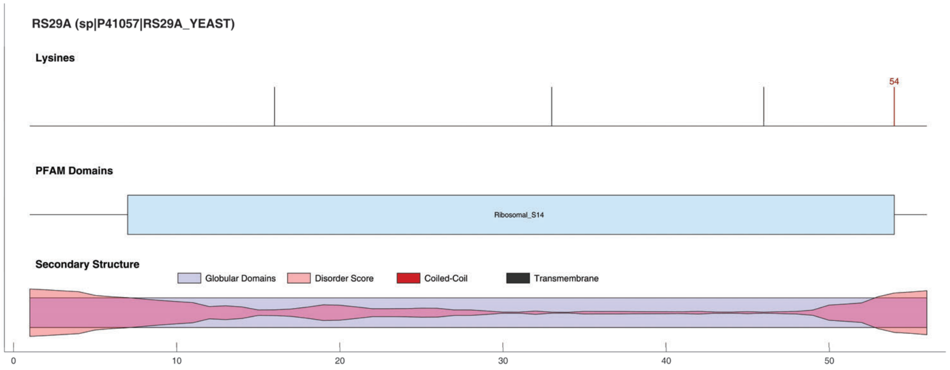
RS29A (sp|P41057|RS29A_YEAST)
Lysines
54
PFAM Domains
Ribosomal_S14
Secondary Structure
Globular Domains
Disorder Score
Coiled-Coil
Transmembrane
0
10
20
30
40
50

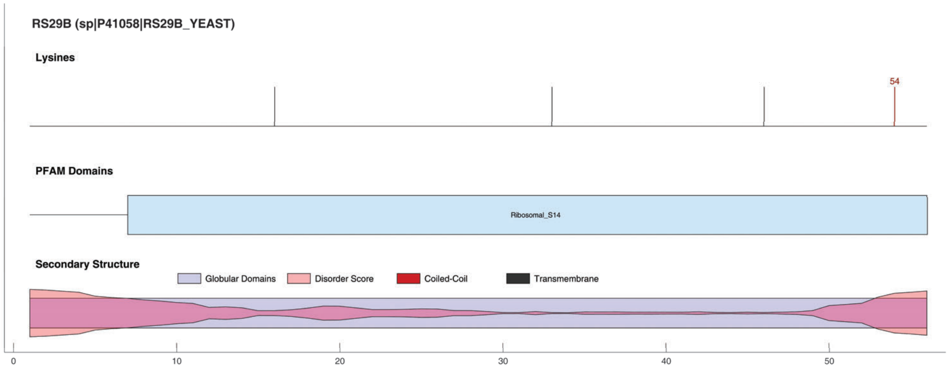
RS29B (sp|P41058|RS29B_YEAST)
Lysines
54
PFAM Domains
Ribosomal_S14
Secondary Structure
Globular Domains
Disorder Score
Coiled-Coil
Transmembrane
0
10
20
30
40
50

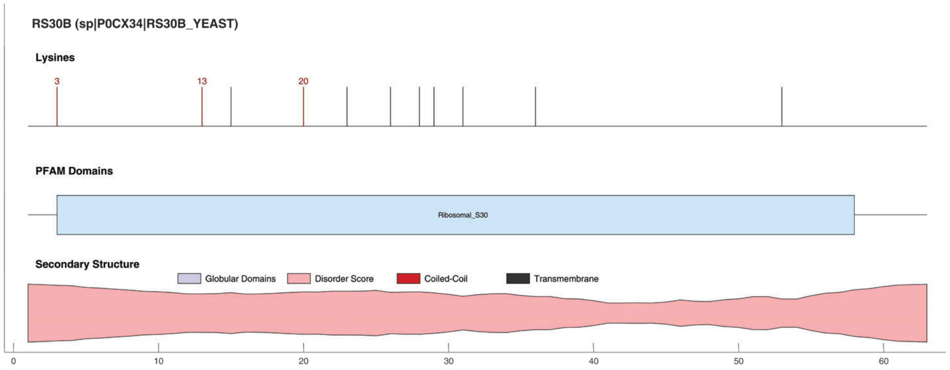
RS30B (sp|P0CX34|RS30B_YEAST)
Lysines
3
13
20
PFAM Domains
Ribosomal_S30
Secondary Structure
Globular Domains
Disorder Score
Coiled-Coil
Transmembrane
0
10
20
30
40
50
60

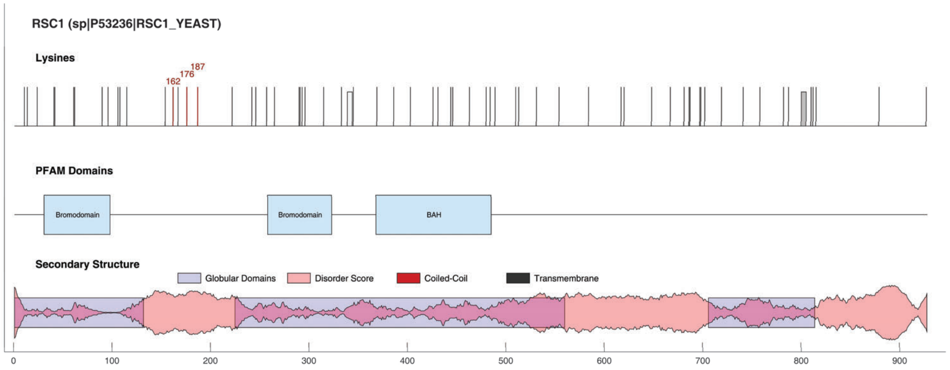
RSC1 (sp|P53236|RSC1_YEAST)
Lysines
162
176
187
PFAM Domains
Bromodomain
Bromodomain
BAH
Secondary Structure
Globular Domains
Disorder Score
Coiled-Coil
Transmembrane
0
100
200
300
400
500
600
700
800
900

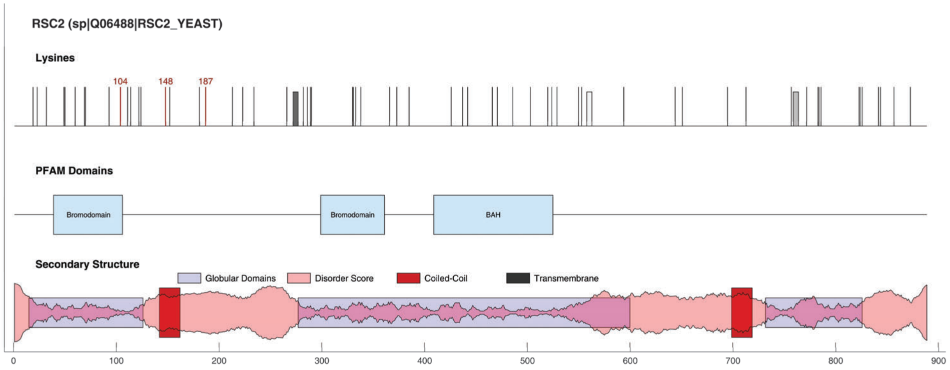
RSC2 (sp|Q06488|RSC2_YEAST)
Lysines
104
148
187
PFAM Domains
Bromodomain
Bromodomain
BAH
Secondary Structure
Globular Domains
Disorder Score
Coiled-Coil
Transmembrane
0
100
200
300
400
500
600
700
800
900

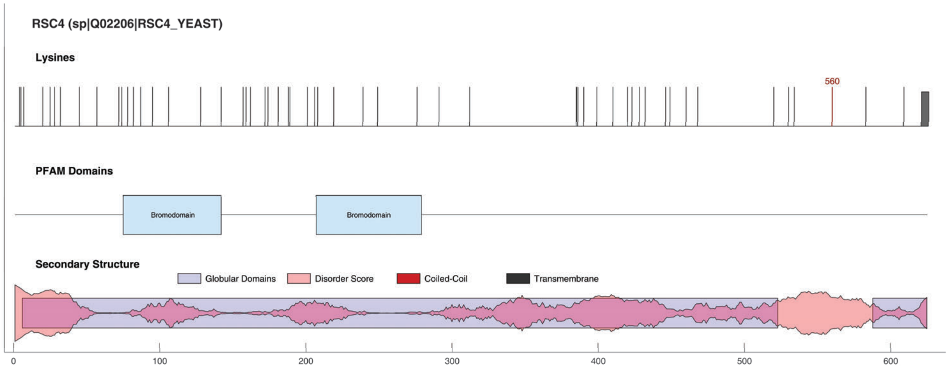
RSC4 (sp|Q02206|RSC4_YEAST)
Lysines
560
PFAM Domains
Bromodomain
Bromodomain
Secondary Structure
Globular Domains
Disorder Score
Coiled-Coil
Transmembrane
0
100
200
300
400
500
600

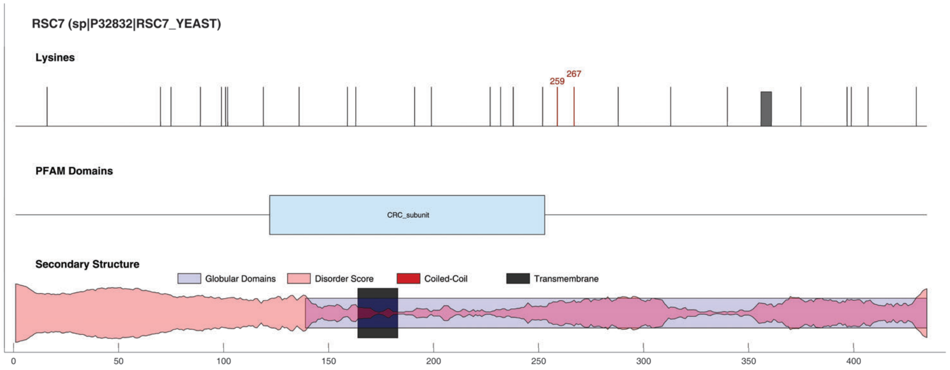
RSC7 (sp|P32832|RSC7_YEAST)
Lysines
259
267
PFAM Domains
CRC_subunit
Secondary Structure
Globular Domains
Disorder Score
Coiled-Coil
Transmembrane
0
50
100
150
200
250
300
350
400

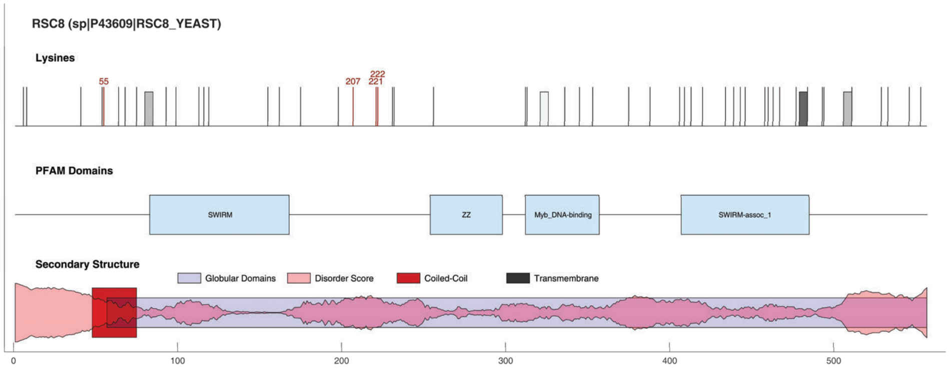
RSC8 (sp|P43609|RSC8_YEAST)
Lysines
55
207
221
222
PFAM Domains
SWIRM
ZZ
Myb_DNA-binding
SWIRM-assoc_1
Secondary Structure
Globular Domains
Disorder Score
Coiled-Coil
Transmembrane
0
100
200
300
400
500

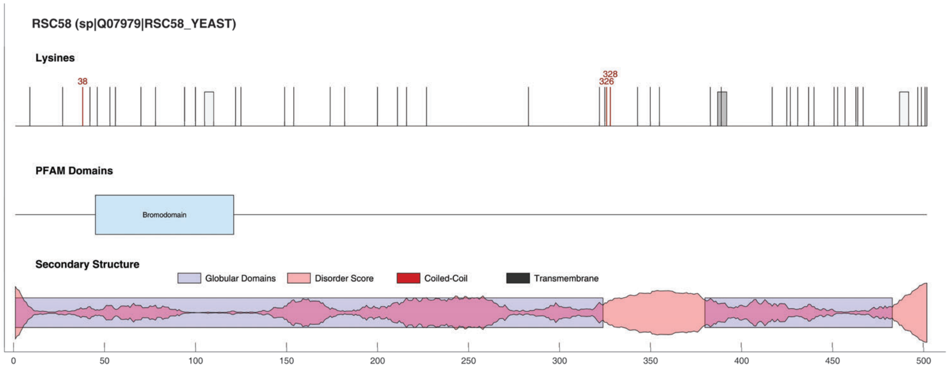
RSC58 (sp|Q07979|RSC58_YEAST)
Lysines
38
326
328
PFAM Domains
Bromodomain
Secondary Structure
Globular Domains
Disorder Score
Coiled-Coil
Transmembrane
0
50
100
150
200
250
300
350
400
450
500

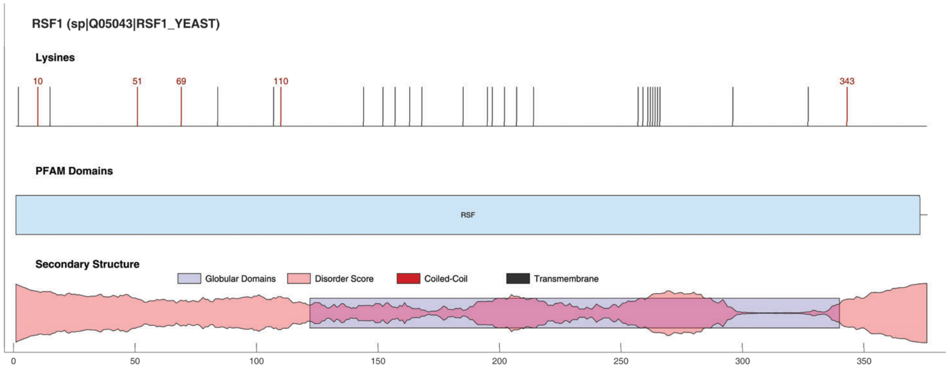
RSF1 (sp|Q05043|RSF1_YEAST)
Lysines
10
51
69
110
343
PFAM Domains
RSF
Secondary Structure
Globular Domains
Disorder Score
Coiled-Coil
Transmembrane
0
50
100
150
200
250
300
350

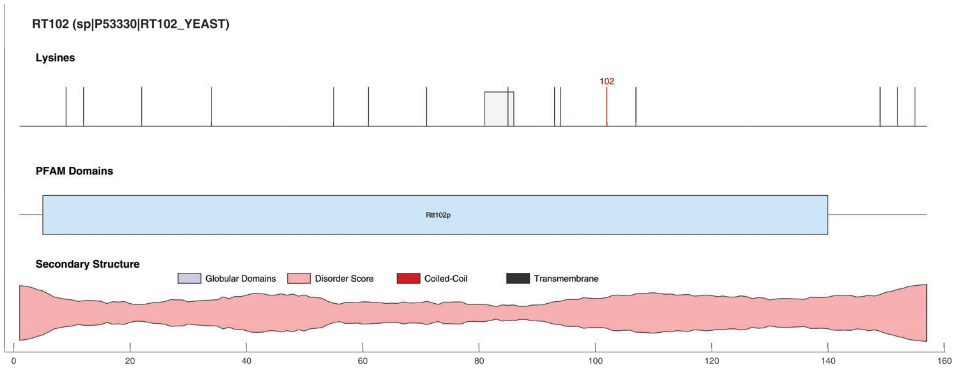
RT102 (sp|P53330|RT102_YEAST)
Lysines
102
PFAM Domains
Rtt102p
Secondary Structure
Globular Domains
Disorder Score
Coiled-Coil
Transmembrane
0
20
40
60
80
100
120
140
160

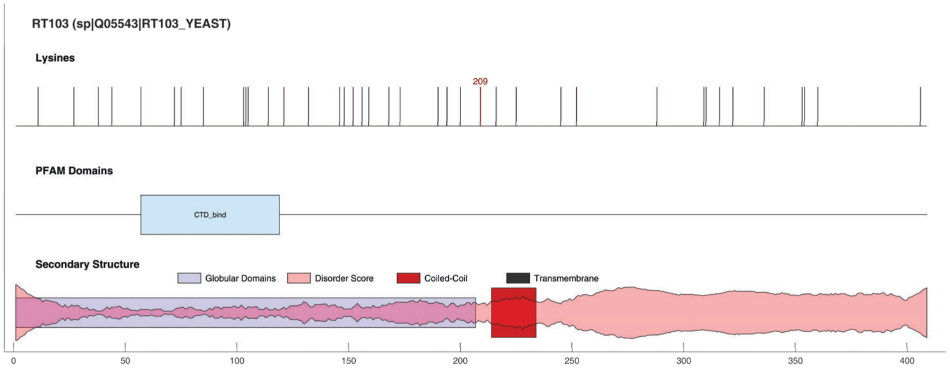
RT103 (sp|Q05543|RT103_YEAST)
Lysines
209
PFAM Domains
CTD_bind
Secondary Structure
Globular Domains
Disorder Score
Coiled-Coil
Transmembrane
0
50
100
150
200
250
300
350
400

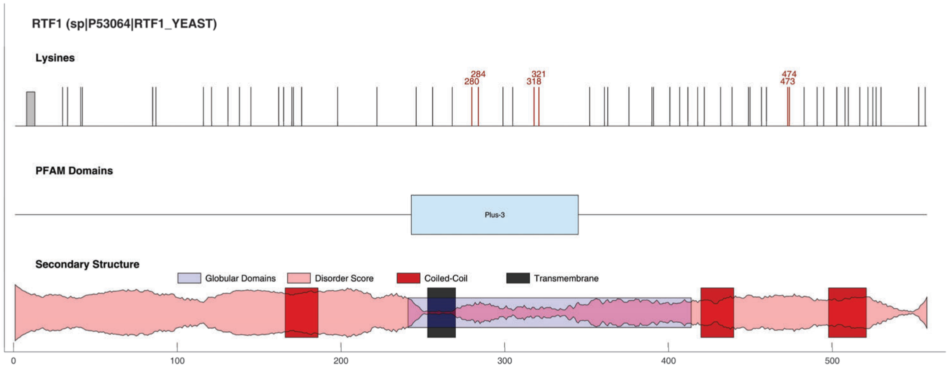
RTF1 (sp|P53064|RTF1_YEAST)
Lysines
284
280
321
318
474
473
PFAM Domains
Plus-3
Secondary Structure
Globular Domains
Disorder Score
Coiled-Coil
Transmembrane
0
100
200
300
400
500

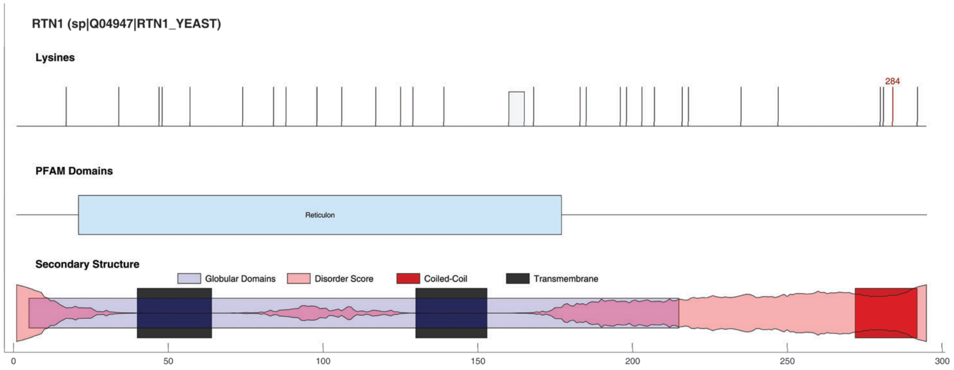
RTN1 (sp|Q04947|RTN1_YEAST)
Lysines
284
PFAM Domains
Reticulon
Secondary Structure
Globular Domains
Disorder Score
Coiled-Coil
Transmembrane
0
50
100
150
200
250
300

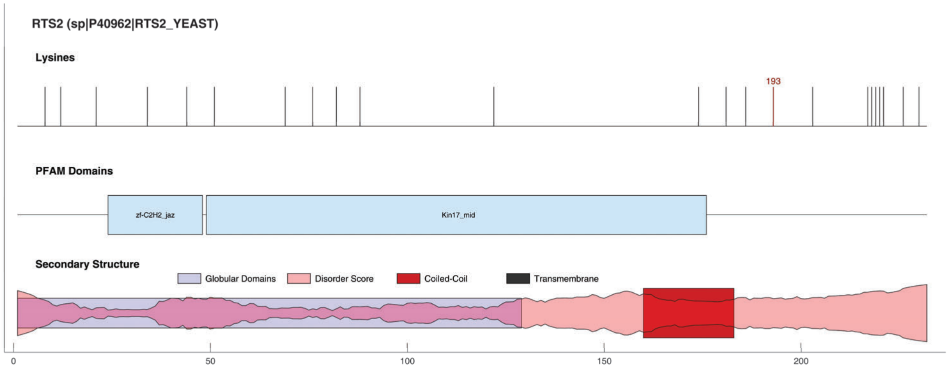
RTS2 (sp|P40962|RTS2_YEAST)
Lysines
193
PFAM Domains
zf-C2H2_jaz
Kin17_mid
Secondary Structure
Globular Domains
Disorder Score
Coiled-Coil
Transmembrane
0
50
100
150
200

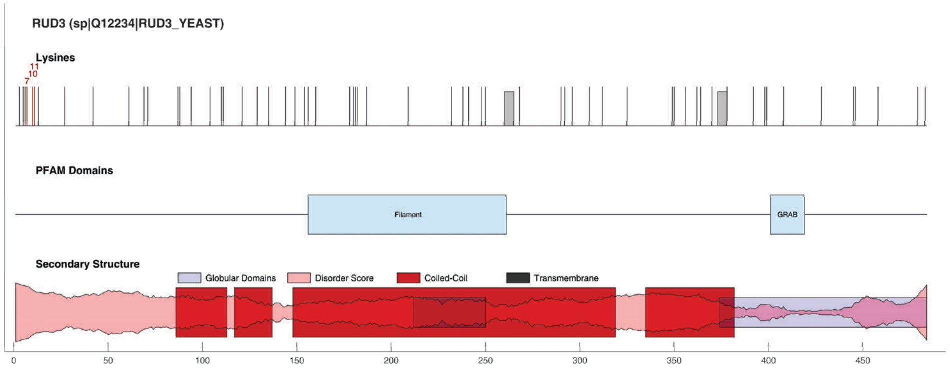

RUD3 (sp|Q12234|RUD3_YEAST)
Lysines
7
10
11
PFAM Domains
Filament
GRAB
Secondary Structure
Globular Domains
Disorder Score
Coiled-Coil
Transmembrane
0
50
100
150
200
250
300
350
400
450

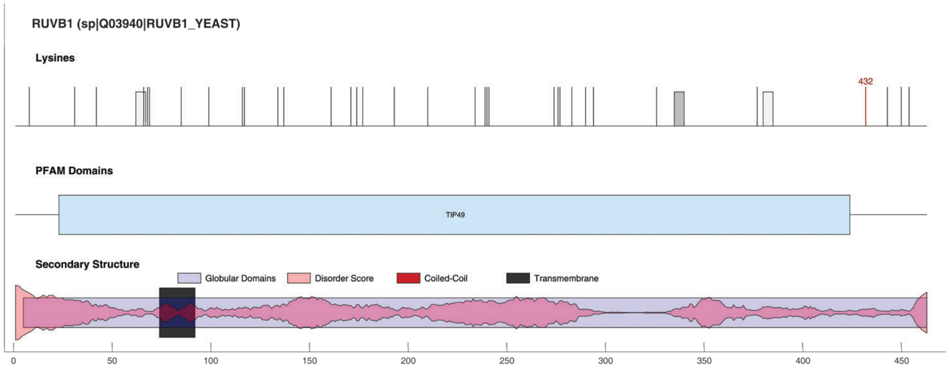

RUVB1 (sp|Q03940|RUVB1_YEAST)
Lysines
432
PFAM Domains
TIP49
Secondary Structure
Globular Domains
Disorder Score
Coiled-Coil
Transmembrane
0
50
100
150
200
250
300
350
400
450

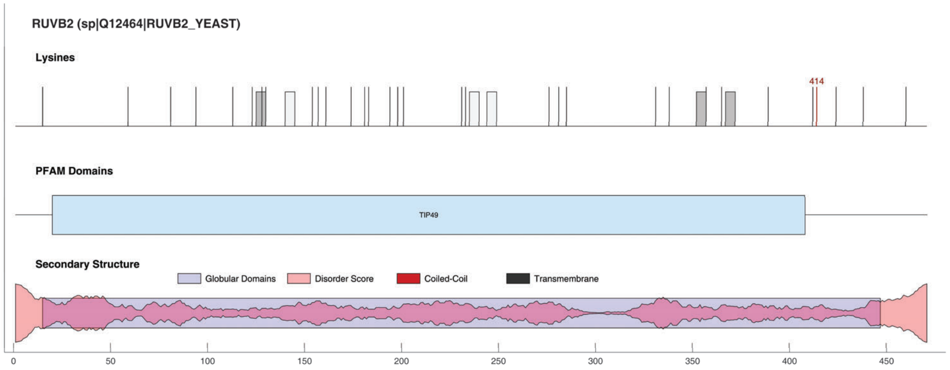

RUVB2 (sp|Q12464|RUVB2_YEAST)
Lysines
414
PFAM Domains
TIP49
Secondary Structure
Globular Domains
Disorder Score
Coiled-Coil
Transmembrane
0
50
100
150
200
250
300
350
400
450

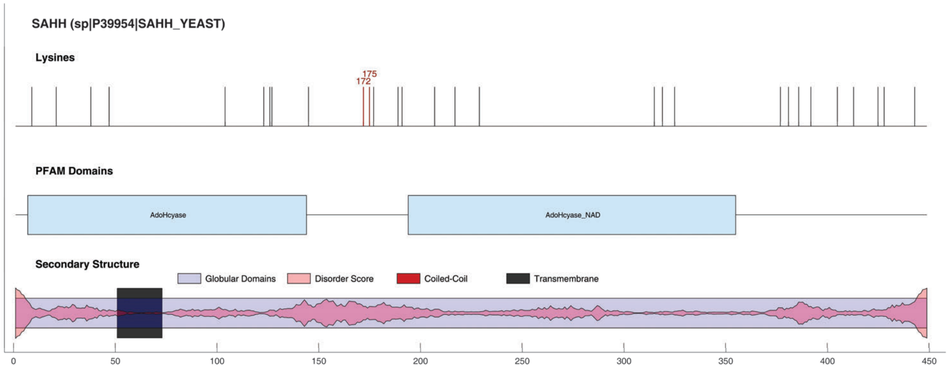

SAHH (sp|P39954|SAHH_YEAST)
Lysines
172
175
PFAM Domains
AdoHcyase
AdoHcyase_NAD
Secondary Structure
Globular Domains
Disorder Score
Coiled-Coil
Transmembrane
0
50
100
150
200
250
300
350
400
450

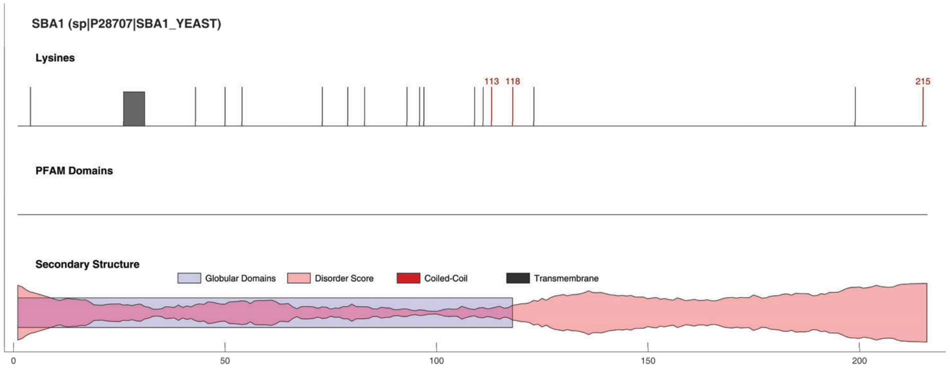
SBA1 (sp|P28707|SBA1_YEAST)
Lysines
113 118
215
PFAM Domains
Secondary Structure
Globular Domains
Disorder Score
Coiled-Coil
Transmembrane
0
50
100
150
200

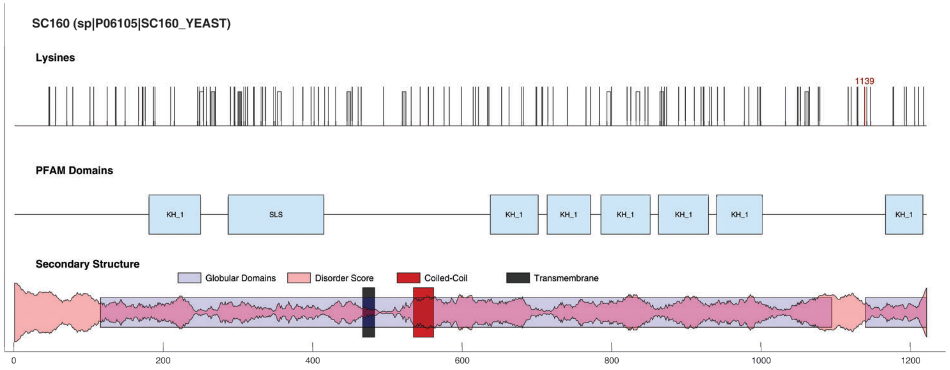
SC160 (sp|P06105|SC160_YEAST)
Lysines
1139
PFAM Domains
KH_1
SLS
KH_1
KH_1
KH_1
KH_1
KH_1
KH_1
Secondary Structure
Globular Domains
Disorder Score
Coiled-Coil
Transmembrane
0
200
400
600
800
1000
1200

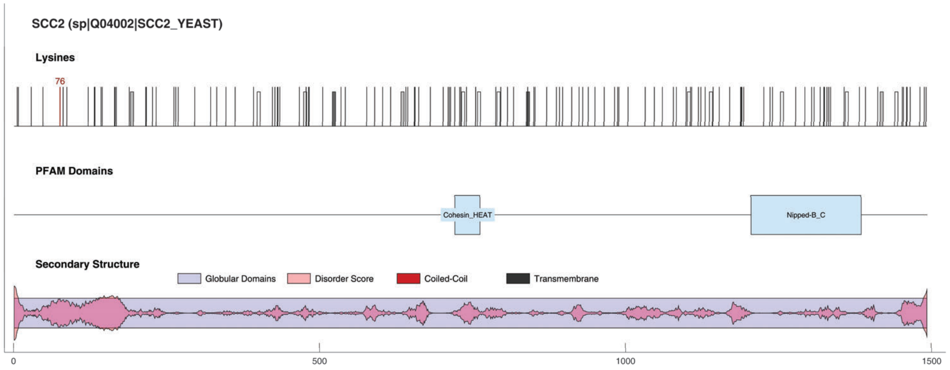
SCC2 (sp|Q04002|SCC2_YEAST)
Lysines
76
PFAM Domains
Cohesin_HEAT
Nipped-B_C
Secondary Structure
Globular Domains
Disorder Score
Coiled-Coil
Transmembrane
0
500
1000
1500

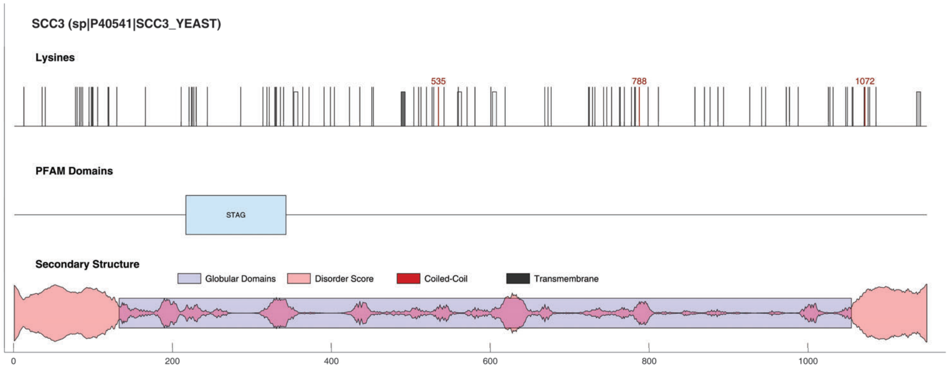
SCC3 (sp|P40541|SCC3_YEAST)
Lysines
535
788
1072
PFAM Domains
STAG
Secondary Structure
Globular Domains
Disorder Score
Coiled-Coil
Transmembrane
0
200
400
600
800
1000

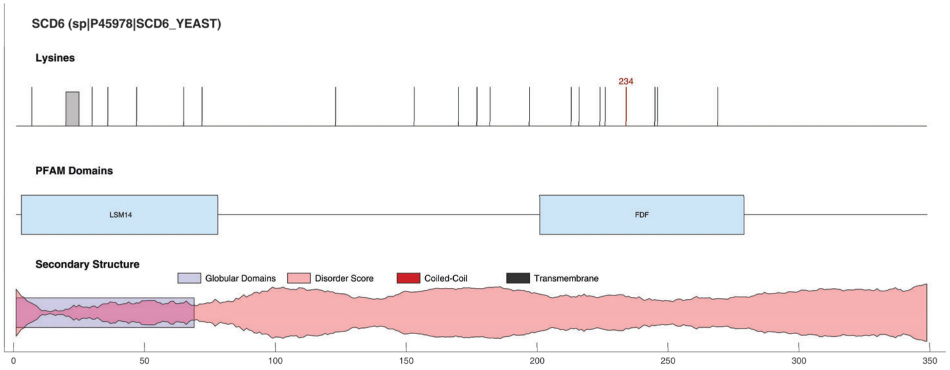
SCD6 (sp|P45978|SCD6_YEAST)
Lysines
234
PFAM Domains
LSM14
FDF
Secondary Structure
Globular Domains
Disorder Score
Coiled-Coil
Transmembrane
0
50
100
150
200
250
300
350

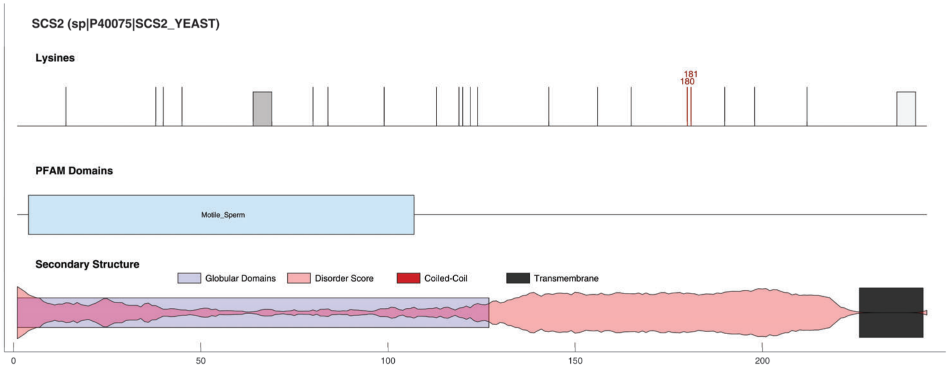
SCS2 (sp|P40075|SCS2_YEAST)
Lysines
181
180
PFAM Domains
Motile_Sperm
Secondary Structure
Globular Domains
Disorder Score
Coiled-Coil
Transmembrane
0
50
100
150
200

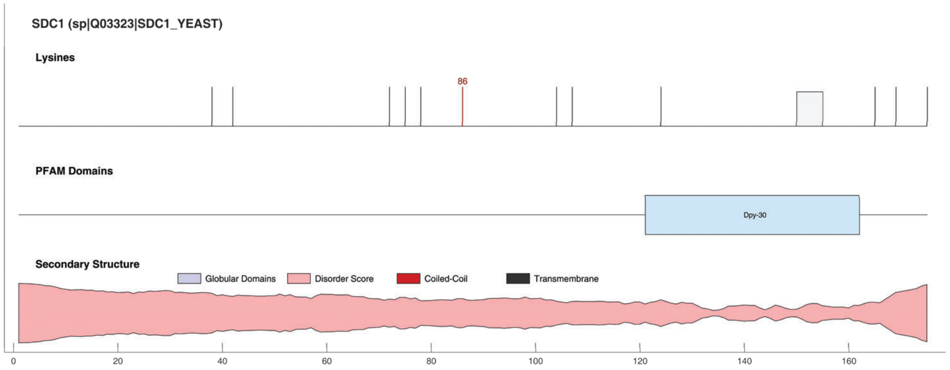
SDC1 (sp|Q03323|SDC1_YEAST)
Lysines
86
PFAM Domains
Dpy-30
Secondary Structure
Globular Domains
Disorder Score
Coiled-Coil
Transmembrane
0
20
40
60
80
100
120
140
160

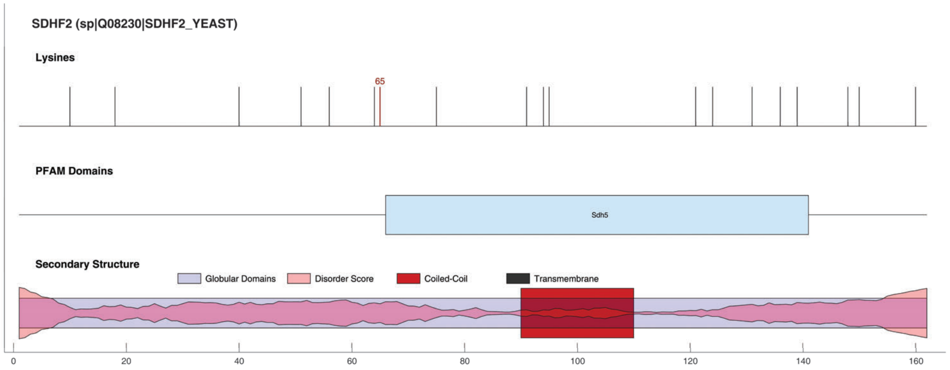
SDHF2 (sp|Q08230|SDHF2_YEAST)
Lysines
65
PFAM Domains
Sdh5
Secondary Structure
Globular Domains
Disorder Score
Coiled-Coil
Transmembrane
0
20
40
60
80
100
120
140
160

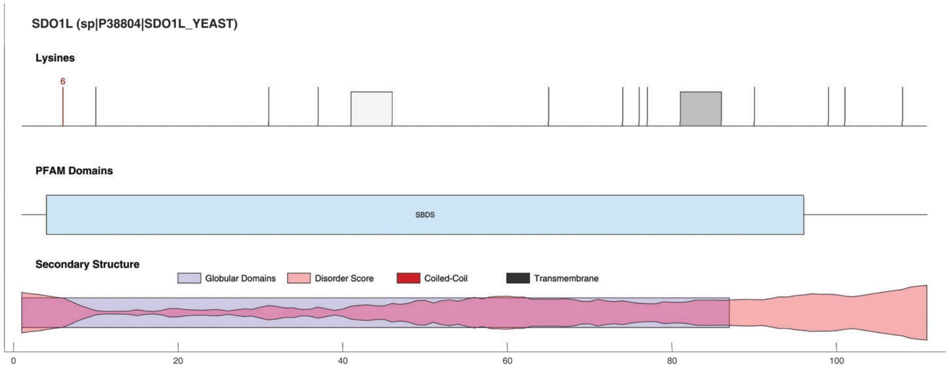

SDO1L (sp|P38804|SDO1L_YEAST)
Lysines
6
PFAM Domains
SBDS
Secondary Structure
Globular Domains
Disorder Score
Coiled-Coil
Transmembrane
0
20
40
60
80
100

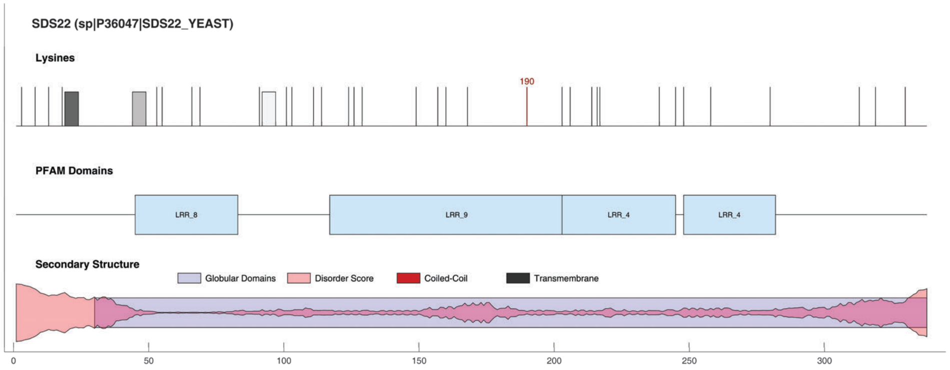

SDS22 (sp|P36047|SDS22_YEAST)
Lysines
190
PFAM Domains
LRR_8
LRR_9
LRR_4
LRR_4
Secondary Structure
Globular Domains
Disorder Score
Coiled-Coil
Transmembrane
0
50
100
150
200
250
300

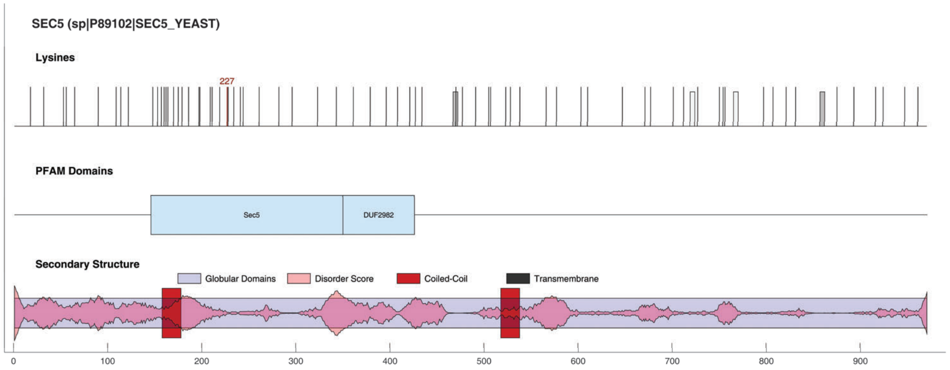

SEC5 (sp|P89102|SEC5_YEAST)
Lysines
227
PFAM Domains
Sec5
DUF2982
Secondary Structure
Globular Domains
Disorder Score
Coiled-Coil
Transmembrane
0
100
200
300
400
500
600
700
800
900

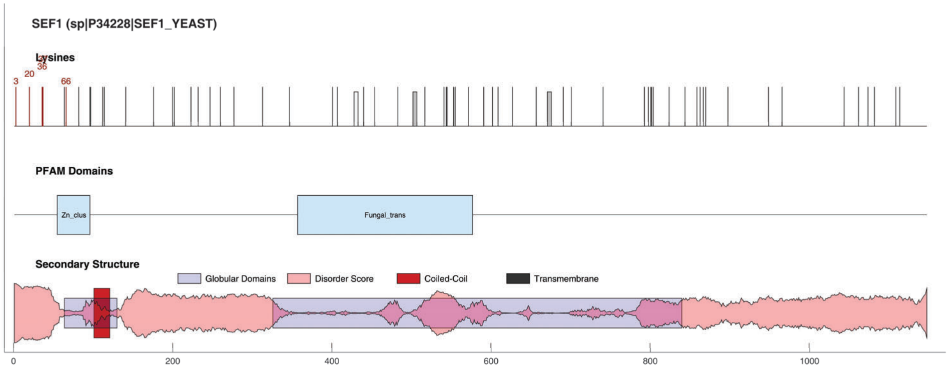

SEF1 (sp|P34228|SEF1_YEAST)
Lysines
3
20
36
66
PFAM Domains
Zn_clus
Fungal_trans
Secondary Structure
Globular Domains
Disorder Score
Coiled-Coil
Transmembrane
0
200
400
600
800
1000

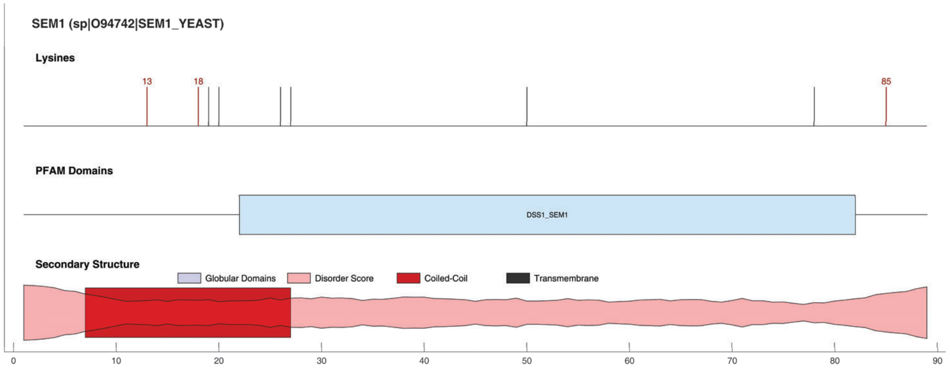
SEM1 (sp|O94742|SEM1_YEAST)
Lysines
13
18
85
PFAM Domains
DSS1_SEM1
Secondary Structure
Globular Domains
Disorder Score
Coiled-Coil
Transmembrane
0
10
20
30
40
50
60
70
80
90

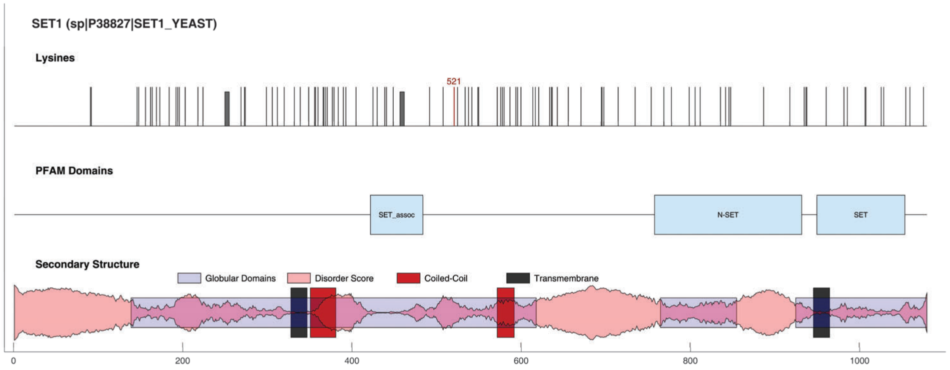
SET1 (sp|P38827|SET1_YEAST)
Lysines
521
PFAM Domains
SET_assoc
N-SET
SET
Secondary Structure
Globular Domains
Disorder Score
Coiled-Coil
Transmembrane
0
200
400
600
800
1000

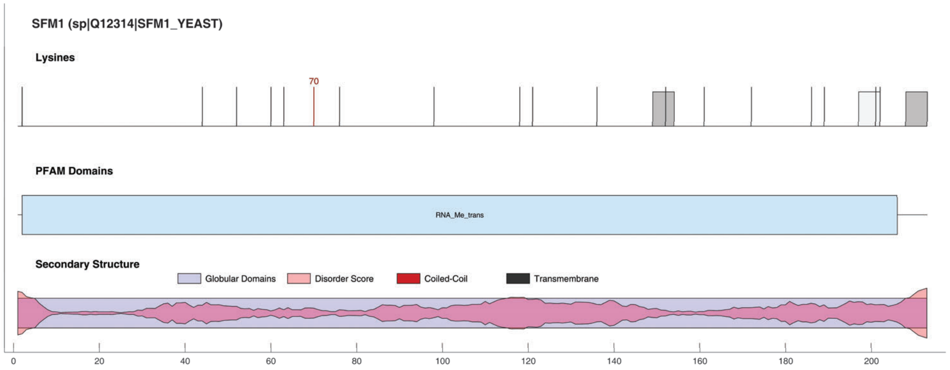
SFM1 (sp|Q12314|SFM1_YEAST)
Lysines
70
PFAM Domains
RNA_Me_trans
Secondary Structure
Globular Domains
Disorder Score
Coiled-Coil
Transmembrane
0
20
40
60
80
100
120
140
160
180
200

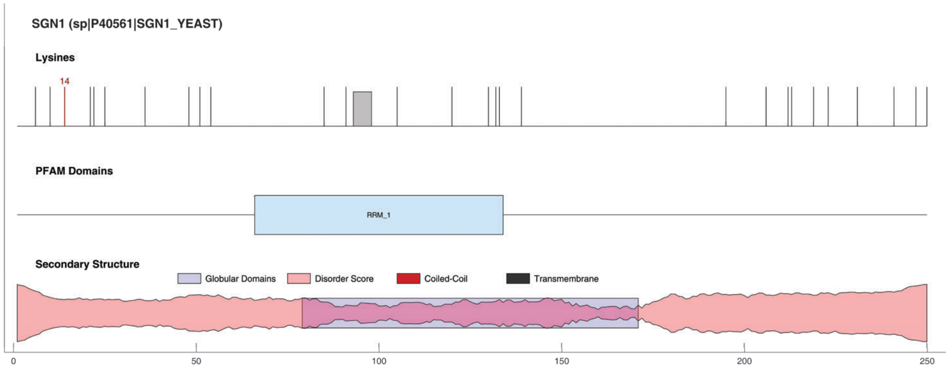
SGN1 (sp|P40561|SGN1_YEAST)
Lysines
14
PFAM Domains
RRM_1
Secondary Structure
Globular Domains
Disorder Score
Coiled-Coil
Transmembrane
0
50
100
150
200
250

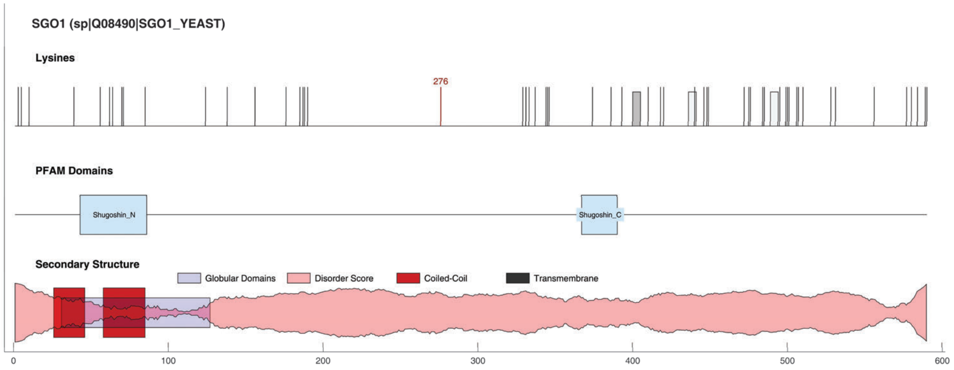
SGO1 (sp|Q08490|SGO1_YEAST)
Lysines
276
PFAM Domains
Shugoshin_N
Shugoshin_C
Secondary Structure
Globular Domains
Disorder Score
Coiled-Coil
Transmembrane
0
100
200
300
400
500
600

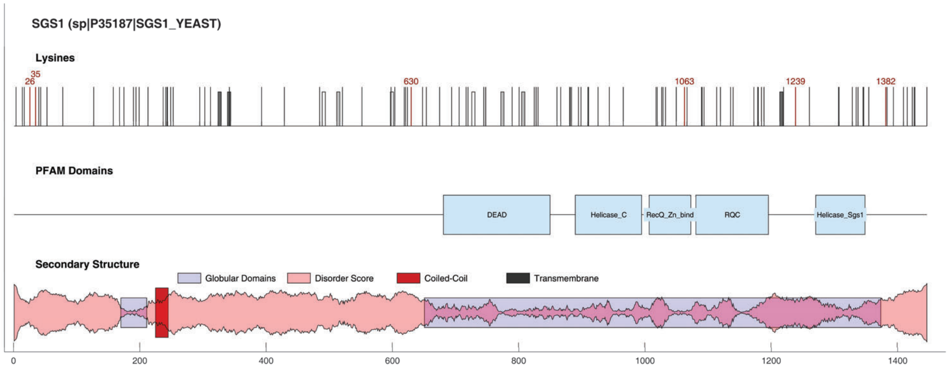
SGS1 (sp|P35187|SGS1_YEAST)
Lysines
26
35
630
1063
1239
1382
PFAM Domains
DEAD
Helicase_C
RecQ_Zn_bind
RQC
Helicase_Sgs1
Secondary Structure
Globular Domains
Disorder Score
Coiled-Coil
Transmembrane
0
200
400
600
800
1000
1200
1400

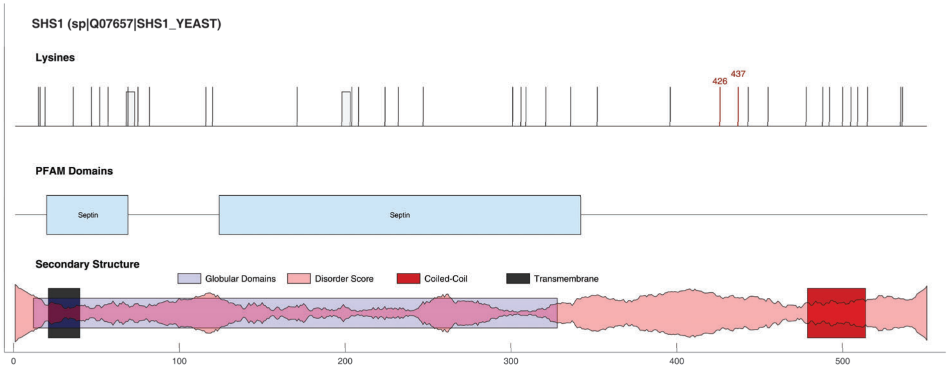
SHS1 (sp|Q07657|SHS1_YEAST)
Lysines
426
437
PFAM Domains
Septin
Septin
Secondary Structure
Globular Domains
Disorder Score
Coiled-Coil
Transmembrane
0
100
200
300
400
500

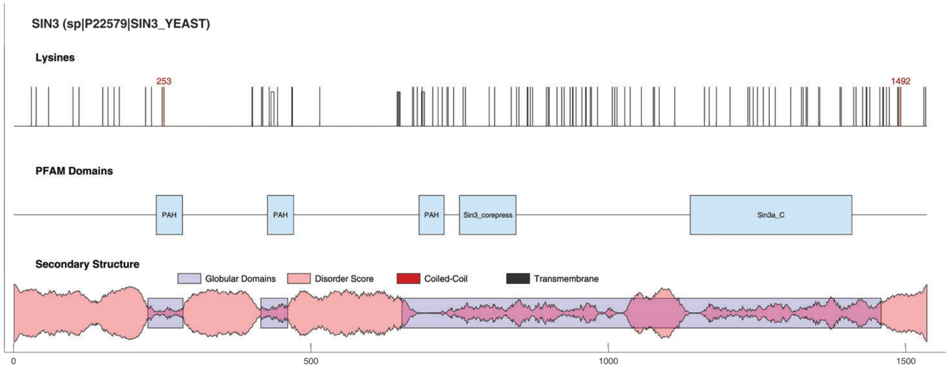
SIN3 (sp|P22579|SIN3_YEAST)
Lysines
253
1492
PFAM Domains
PAH
PAH
PAH
Sin3_corepress
Sin3a_C
Secondary Structure
Globular Domains
Disorder Score
Coiled-Coil
Transmembrane
0
500
1000
1500

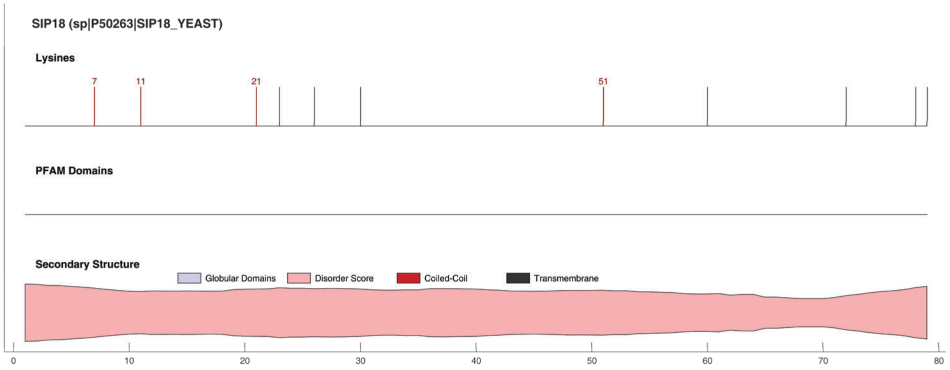
SIP18 (sp|P50263|SIP18_YEAST)
Lysines
7
11
21
51
PFAM Domains
Secondary Structure
Globular Domains
Disorder Score
Coiled-Coil
Transmembrane
0
10
20
30
40
50
60
70
80

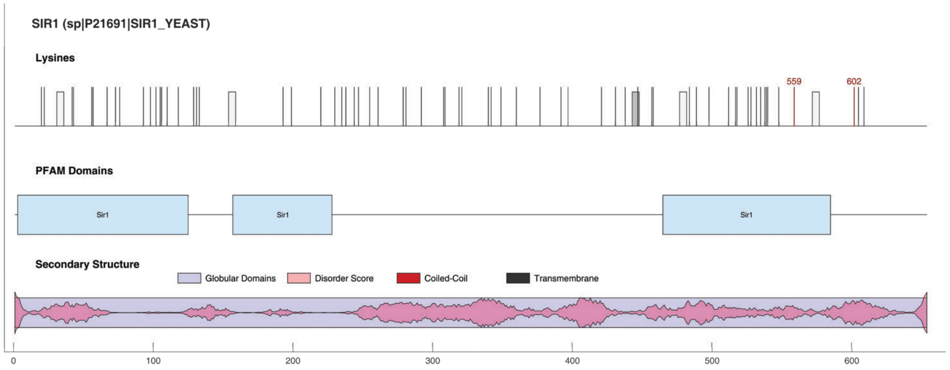
SIR1 (sp|P21691|SIR1_YEAST)
Lysines
559
602
PFAM Domains
Sir1
Sir1
Sir1
Secondary Structure
Globular Domains
Disorder Score
Coiled-Coil
Transmembrane
0
100
200
300
400
500
600

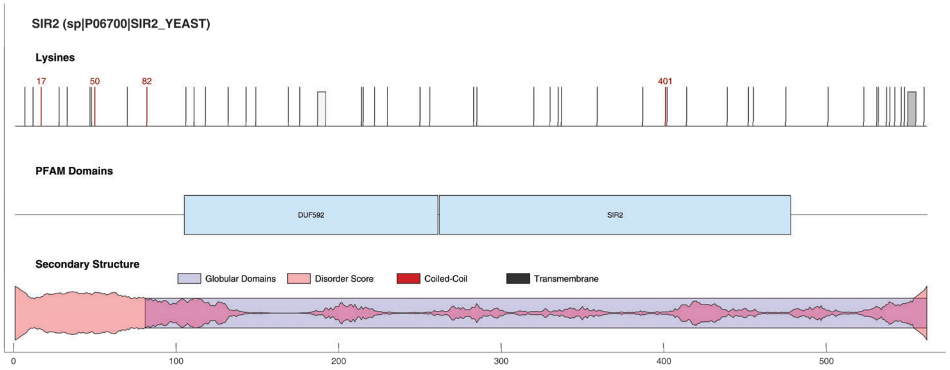
SIR2 (sp|P06700|SIR2_YEAST)
Lysines
17
50
82
401
PFAM Domains
DUF592
SIR2
Secondary Structure
Globular Domains
Disorder Score
Coiled-Coil
Transmembrane
0
100
200
300
400
500

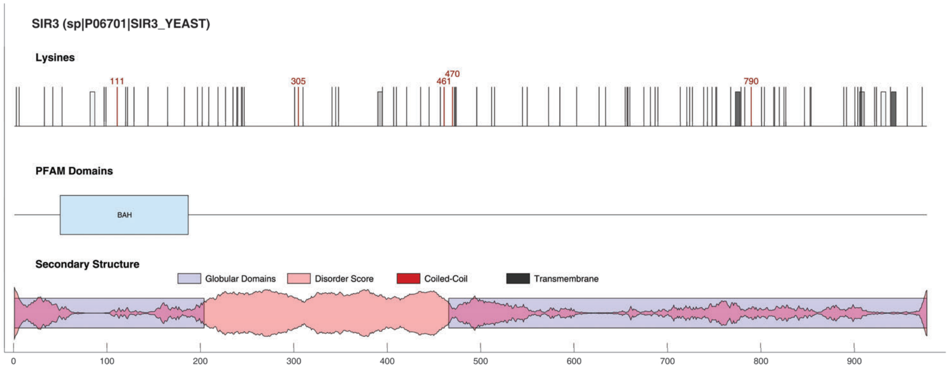
SIR3 (sp|P06701|SIR3_YEAST)
Lysines
111
305
470
461
790
PFAM Domains
BAH
Secondary Structure
Globular Domains
Disorder Score
Coiled-Coil
Transmembrane
0
100
200
300
400
500
600
700
800
900

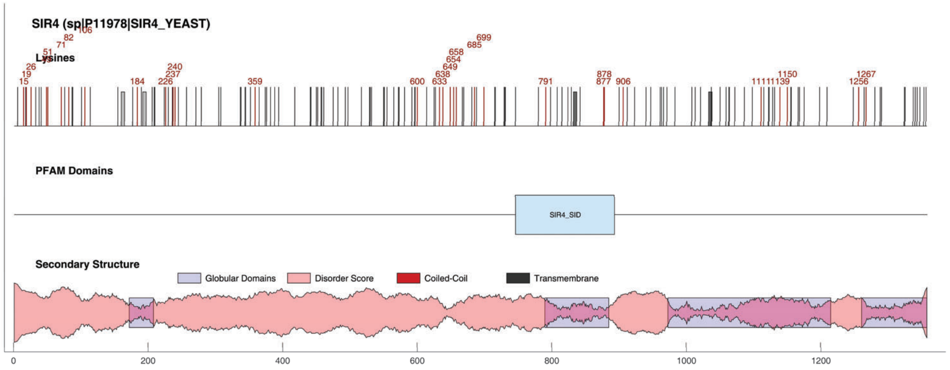
SIR4 (sp|P11978|SIR4_YEAST)
Lysines
15
19
26
51
71
82
106
184
226
237
240
359
600
633
638
649
654
658
685
699
791
877
878
906
1111139
1150
1256
1267
PFAM Domains
SIR4_SID
Secondary Structure
Globular Domains
Disorder Score
Coiled-Coil
Transmembrane
0
200
400
600
800
1000
1200

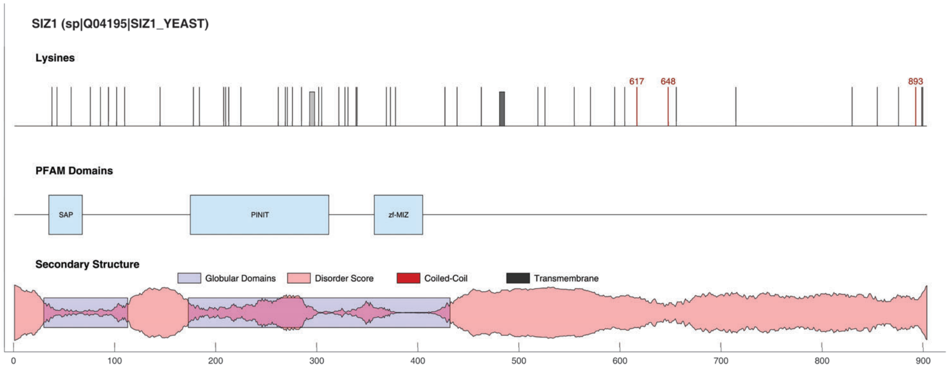
SIZ1 (sp|Q04195|SIZ1_YEAST)
Lysines
617
648
893
PFAM Domains
SAP
PINIT
zf-MIZ
Secondary Structure
Globular Domains
Disorder Score
Coiled-Coil
Transmembrane
0
100
200
300
400
500
600
700
800
900

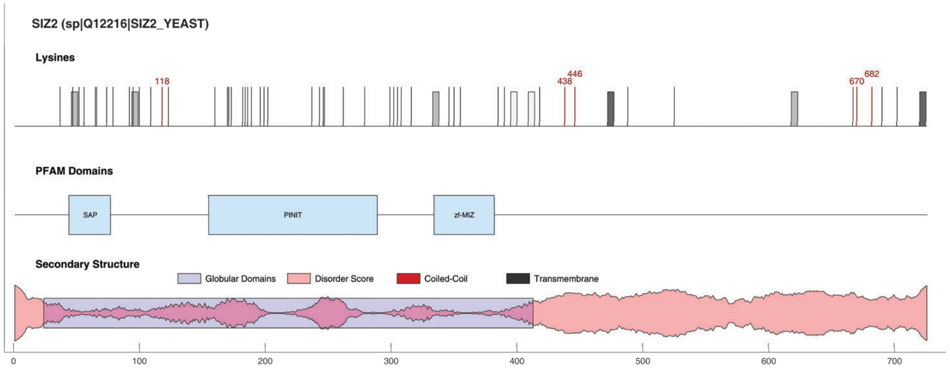
SIZ2 (sp|Q12216|SIZ2_YEAST)
Lysines
118
438
446
670
682
PFAM Domains
SAP
PINIT
zf-MIZ
Secondary Structure
Globular Domains
Disorder Score
Coiled-Coil
Transmembrane
0
100
200
300
400
500
600
700

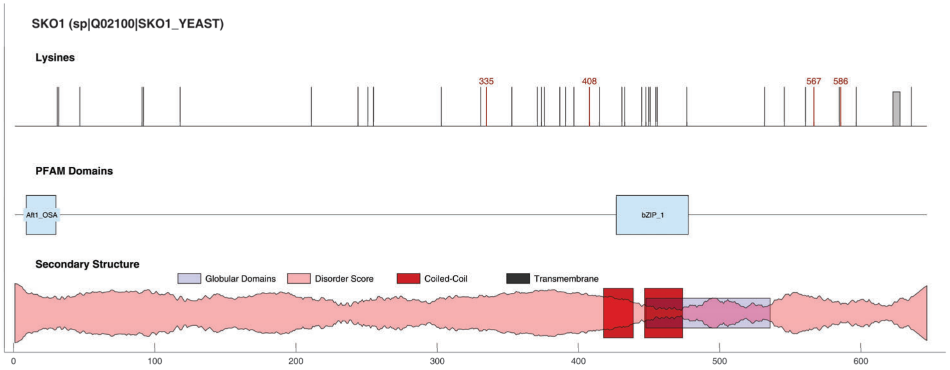
SKO1 (sp|Q02100|SKO1_YEAST)
Lysines
335
408
567
586
PFAM Domains
Aft1_OSA
bZIP_1
Secondary Structure
Globular Domains
Disorder Score
Coiled-Coil
Transmembrane
0
100
200
300
400
500
600

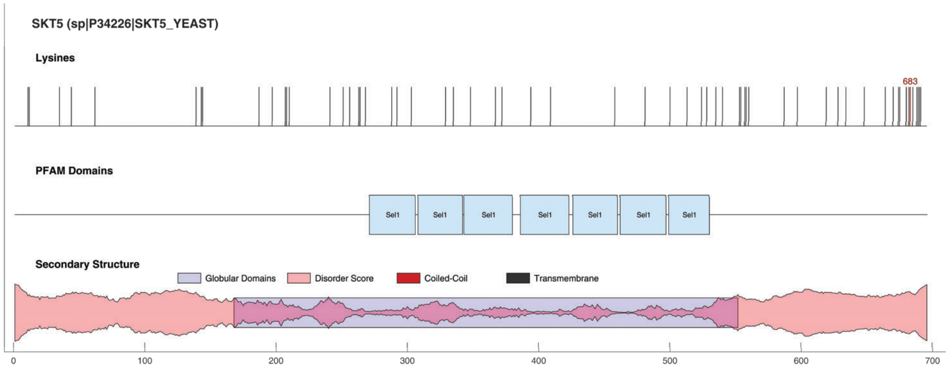
SKT5 (sp|P34226|SKT5_YEAST)
Lysines
683
PFAM Domains
Sel1
Sel1
Sel1
Sel1
Sel1
Sel1
Sel1
Secondary Structure
Globular Domains
Disorder Score
Coiled-Coil
Transmembrane
0
100
200
300
400
500
600
700

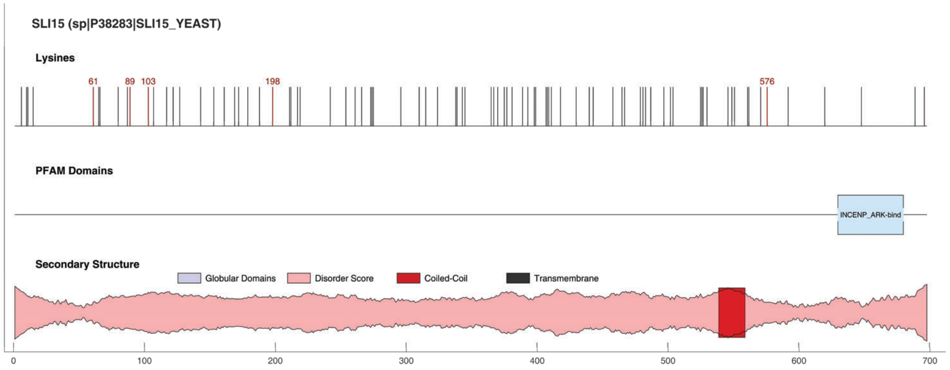
SLI15 (sp|P38283|SLI15_YEAST)
Lysines
61
89
103
198
576
PFAM Domains
INCENP_ARK-bind
Secondary Structure
Globular Domains
Disorder Score
Coiled-Coil
Transmembrane
0
100
200
300
400
500
600
700

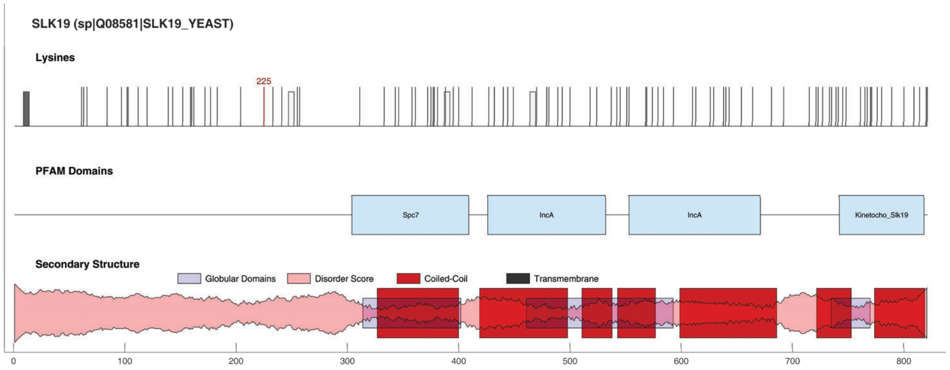
SLK19 (sp|Q08581|SLK19_YEAST)
Lysines
225
PFAM Domains
Spc7
IncA
IncA
Kinetocho_Slk19
Secondary Structure
Globular Domains
Disorder Score
Coiled-Coil
Transmembrane
0
100
200
300
400
500
600
700
800

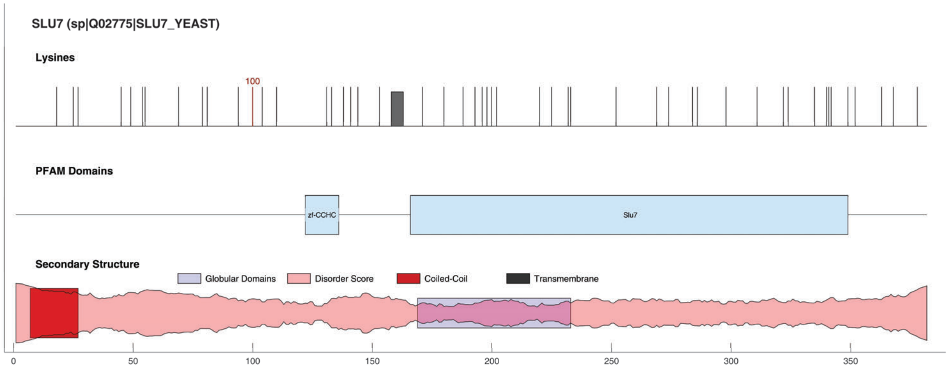
SLU7 (sp|Q02775|SLU7_YEAST)
Lysines
100
PFAM Domains
zf-CCHC
Slu7
Secondary Structure
Globular Domains
Disorder Score
Coiled-Coil
Transmembrane
0
50
100
150
200
250
300
350

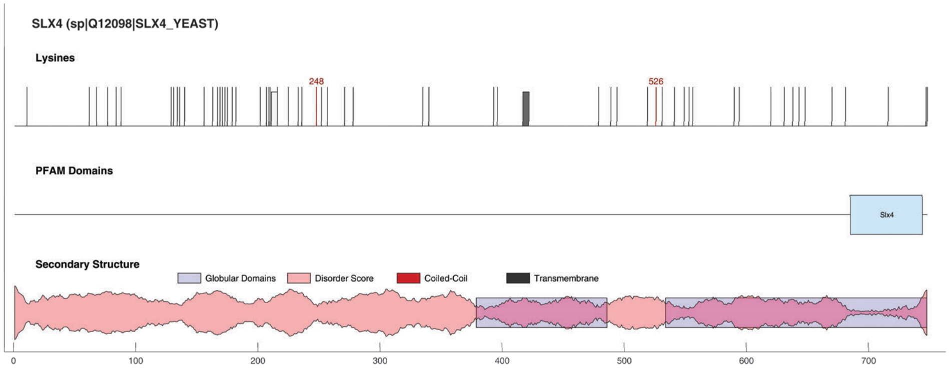
SLX4 (sp|Q12098|SLX4_YEAST)
Lysines
248
526
PFAM Domains
Slx4
Secondary Structure
Globular Domains
Disorder Score
Coiled-Coil
Transmembrane
0
100
200
300
400
500
600
700

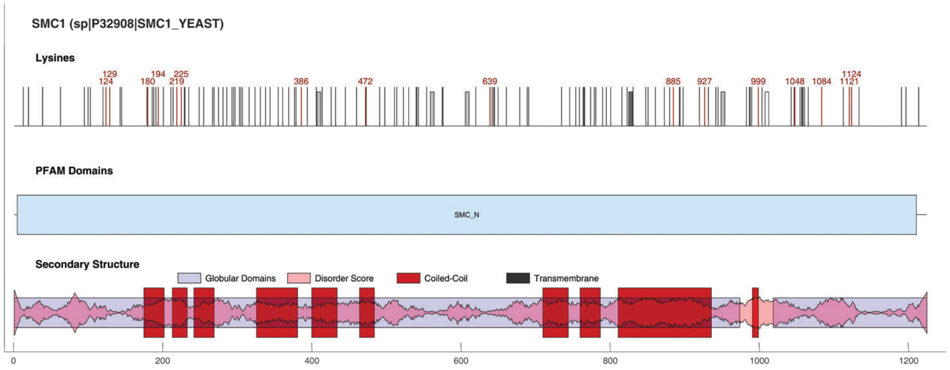
SMC1 (sp|P32908|SMC1_YEAST)
Lysines
124 129 180 194 219 225 386 472 639 885 927 999 1048 1084 1121 1124
PFAM Domains
SMC_N
Secondary Structure
Globular Domains
Disorder Score
Coiled-Coil
Transmembrane
0
200
400
600
800
1000
1200

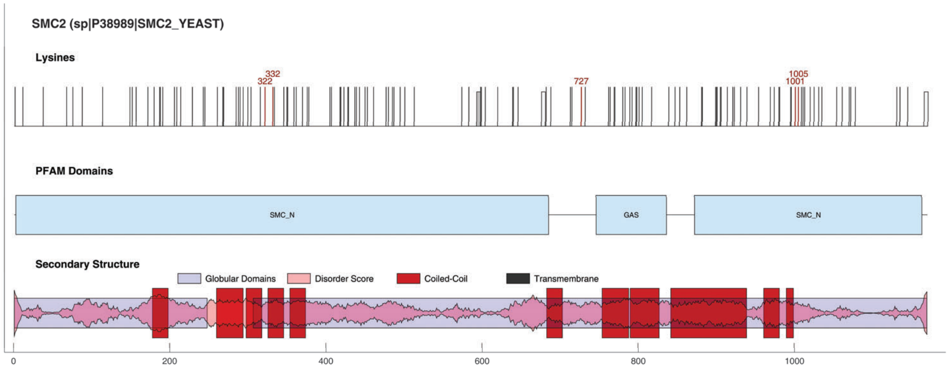
SMC2 (sp|P38989|SMC2_YEAST)
Lysines
322 332 727 1001 1005
PFAM Domains
SMC_N
GAS
SMC_N
Secondary Structure
Globular Domains
Disorder Score
Coiled-Coil
Transmembrane
0
200
400
600
800
1000

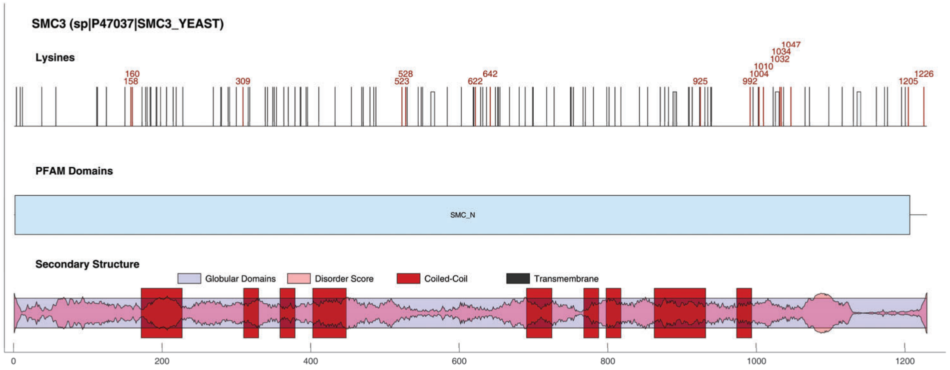
SMC3 (sp|P47037|SMC3_YEAST)
Lysines
158 160 309 523 528 622 642 925 992 1004 1010 1032 1034 1047 1205 1226
PFAM Domains
SMC_N
Secondary Structure
Globular Domains
Disorder Score
Coiled-Coil
Transmembrane
0
200
400
600
800
1000
1200

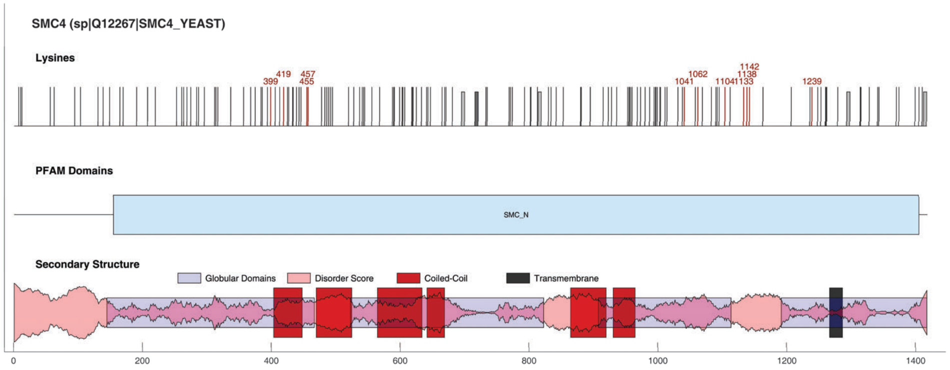
SMC4 (sp|Q12267|SMC4_YEAST)
Lysines
399
419
455
457
1041
1062
1104
1133
1138
1142
1239
PFAM Domains
SMC_N
Secondary Structure
Globular Domains
Disorder Score
Coiled-Coil
Transmembrane
0
200
400
600
800
1000
1200
1400

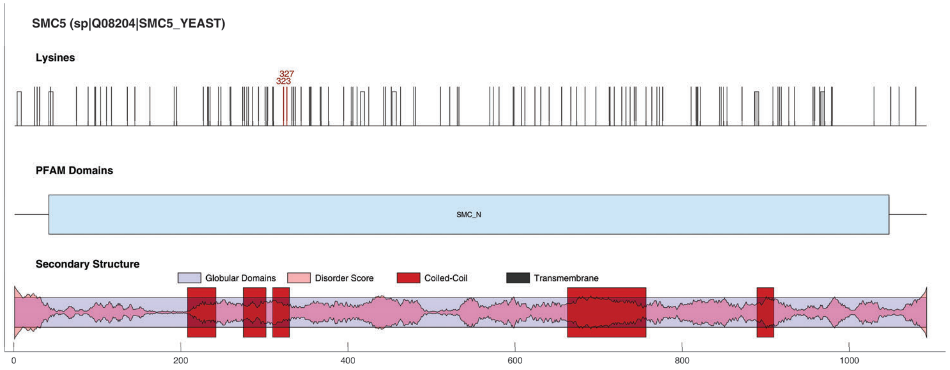
SMC5 (sp|Q08204|SMC5_YEAST)
Lysines
323
327
PFAM Domains
SMC_N
Secondary Structure
Globular Domains
Disorder Score
Coiled-Coil
Transmembrane
0
200
400
600
800
1000

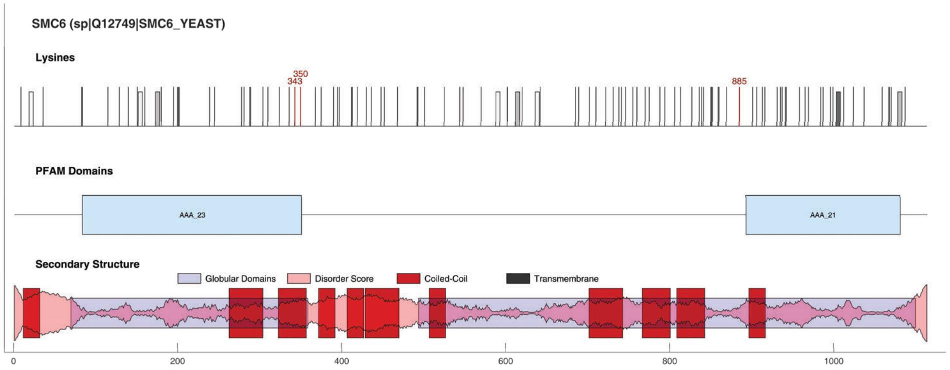
SMC6 (sp|Q12749|SMC6_YEAST)
Lysines
343
350
885
PFAM Domains
AAA_23
AAA_21
Secondary Structure
Globular Domains
Disorder Score
Coiled-Coil
Transmembrane
0
200
400
600
800
1000

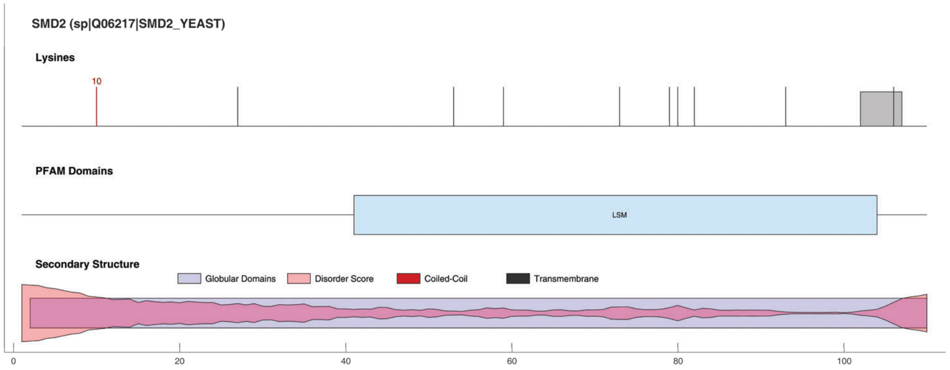
SMD2 (sp|Q06217|SMD2_YEAST)
Lysines
10
PFAM Domains
LSM
Secondary Structure
Globular Domains
Disorder Score
Coiled-Coil
Transmembrane
0
20
40
60
80
100

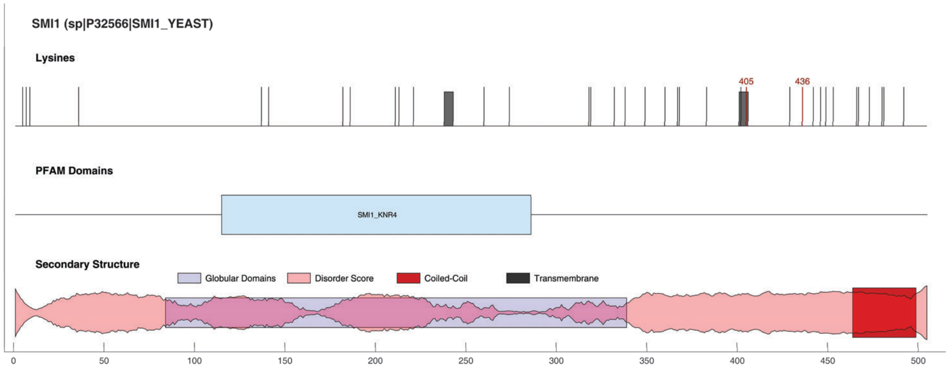
SMI1 (sp|P32566|SMI1_YEAST)
Lysines
405
436
PFAM Domains
SMI1_KNR4
Secondary Structure
Globular Domains
Disorder Score
Coiled-Coil
Transmembrane
0 50 100 150 200 250 300 350 400 450 500

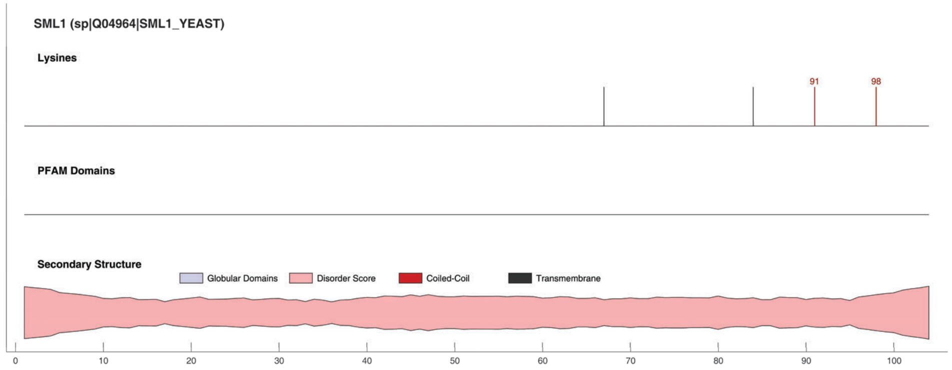
SML1 (sp|Q04964|SML1_YEAST)
Lysines
91
98
PFAM Domains
Secondary Structure
Globular Domains
Disorder Score
Coiled-Coil
Transmembrane
0 10 20 30 40 50 60 70 80 90 100

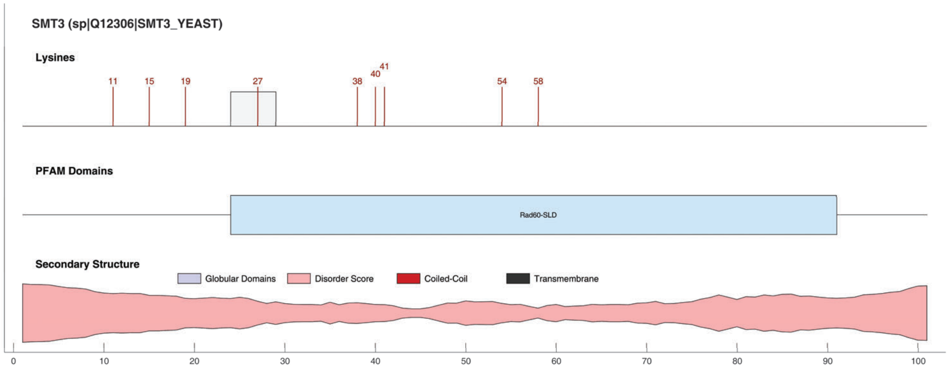
SMT3 (sp|Q12306|SMT3_YEAST)
Lysines
11
15
19
27
38
40
41
54
58
PFAM Domains
Rad60-SLD
Secondary Structure
Globular Domains
Disorder Score
Coiled-Coil
Transmembrane
0 10 20 30 40 50 60 70 80 90 100

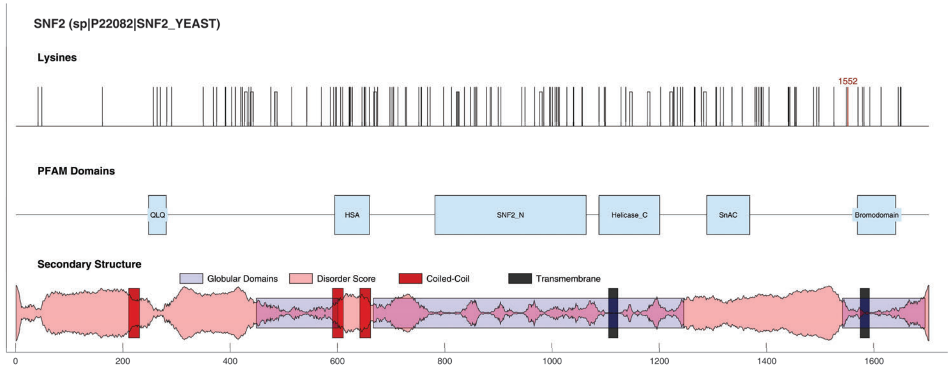
SNF2 (sp|P22082|SNF2_YEAST)
Lysines
1552
PFAM Domains
QLQ
HSA
SNF2_N
Helicase_C
SnAC
Bromodomain
Secondary Structure
Globular Domains
Disorder Score
Coiled-Coil
Transmembrane
0 200 400 600 800 1000 1200 1400 1600

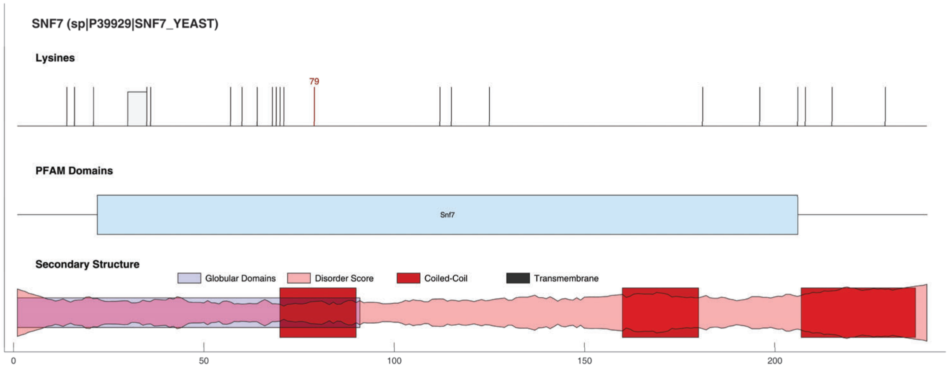
SNF7 (sp|P39929|SNF7_YEAST)
Lysines
79
PFAM Domains
Snf7
Secondary Structure
Globular Domains
Disorder Score
Coiled-Coil
Transmembrane
0
50
100
150
200

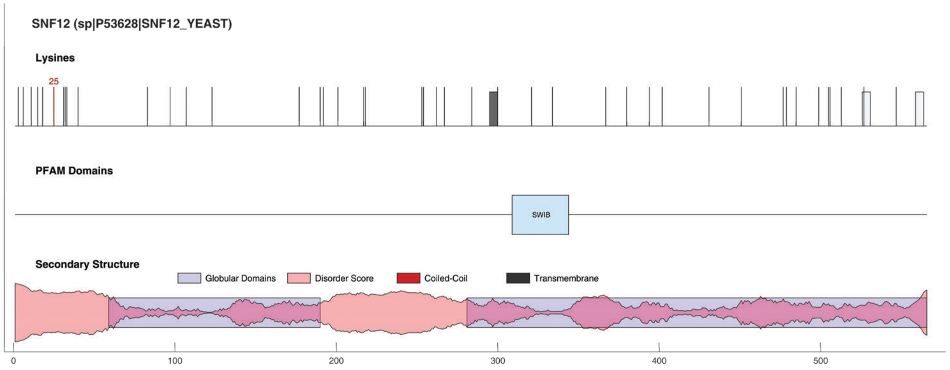
SNF12 (sp|P53628|SNF12_YEAST)
Lysines
25
PFAM Domains
SWIB
Secondary Structure
Globular Domains
Disorder Score
Coiled-Coil
Transmembrane
0
100
200
300
400
500

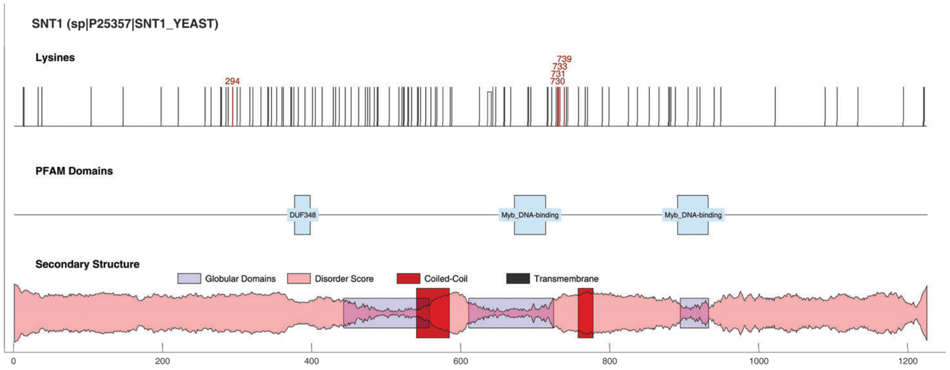
SNT1 (sp|P25357|SNT1_YEAST)
Lysines
294
739
733
731
730
PFAM Domains
DUF348
Myb_DNA-binding
Myb_DNA-binding
Secondary Structure
Globular Domains
Disorder Score
Coiled-Coil
Transmembrane
0
200
400
600
800
1000
1200

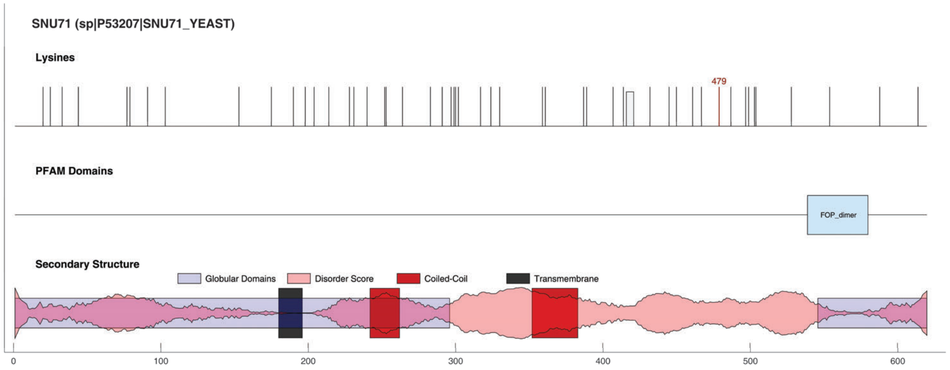
SNU71 (sp|P53207|SNU71_YEAST)
Lysines
479
PFAM Domains
FOP_dimer
Secondary Structure
Globular Domains
Disorder Score
Coiled-Coil
Transmembrane
0
100
200
300
400
500
600

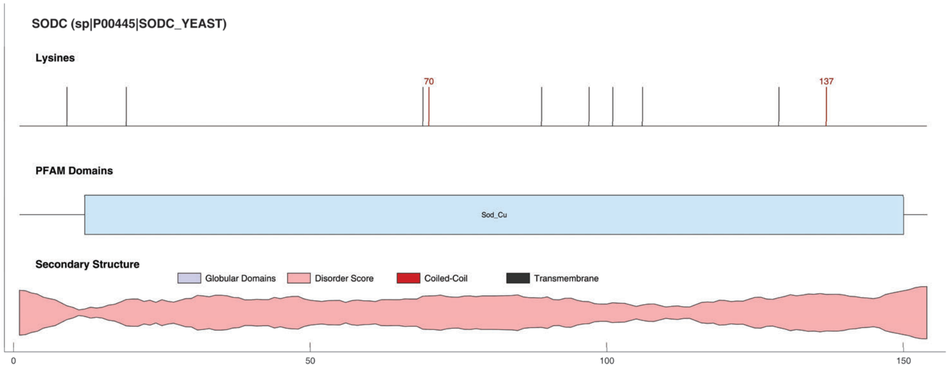
SODC (sp|P00445|SODC_YEAST)
Lysines
70
137
PFAM Domains
Sod_Cu
Secondary Structure
Globular Domains
Disorder Score
Coiled-Coil
Transmembrane
0
50
100
150

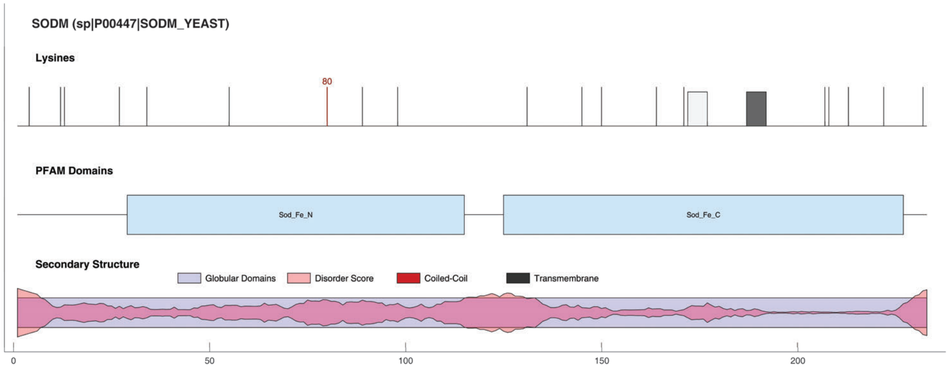
SODM (sp|P00447|SODM_YEAST)
Lysines
80
PFAM Domains
Sod_Fe_N
Sod_Fe_C
Secondary Structure
Globular Domains
Disorder Score
Coiled-Coil
Transmembrane
0
50
100
150
200

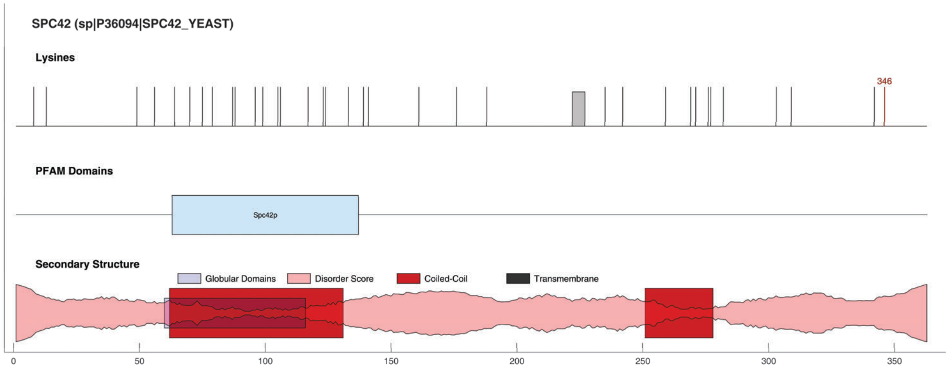
SPC42 (sp|P36094|SPC42_YEAST)
Lysines
346
PFAM Domains
Spc42p
Secondary Structure
Globular Domains
Disorder Score
Coiled-Coil
Transmembrane
0
50
100
150
200
250
300
350

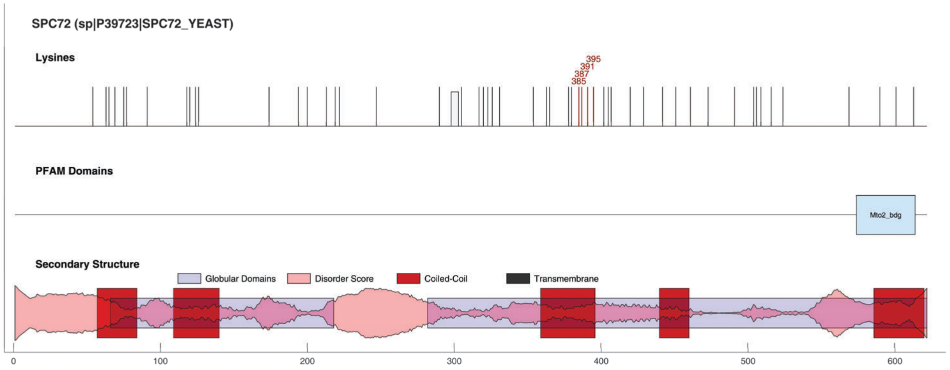
SPC72 (sp|P39723|SPC72_YEAST)
Lysines
395
391
387
385
PFAM Domains
Mto2_bdg
Secondary Structure
Globular Domains
Disorder Score
Coiled-Coil
Transmembrane
0
100
200
300
400
500
600

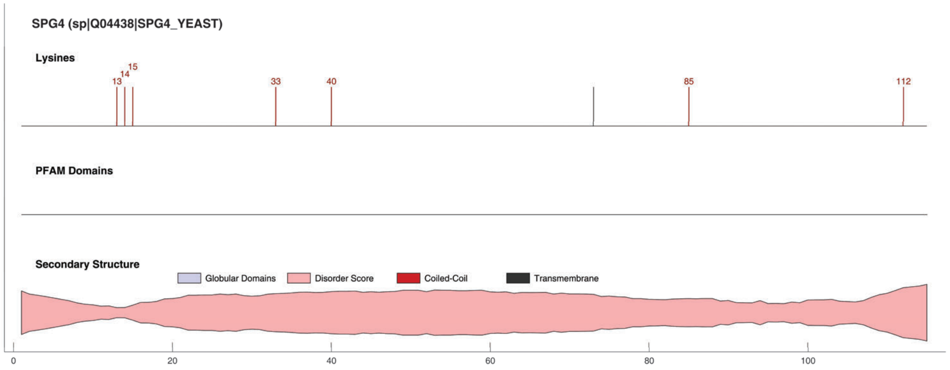
SPG4 (sp|Q04438|SPG4_YEAST)
Lysines
13
14
15
33
40
85
112
PFAM Domains
Secondary Structure
Globular Domains
Disorder Score
Coiled-Coil
Transmembrane
0
20
40
60
80
100

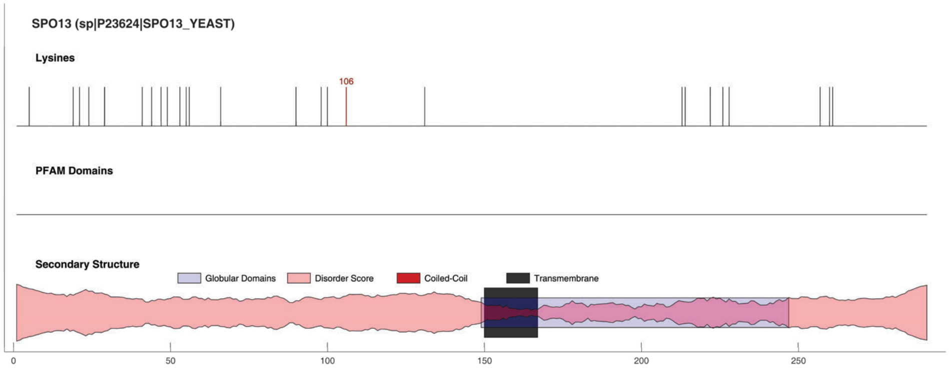
SPO13 (sp|P23624|SPO13_YEAST)
Lysines
106
PFAM Domains
Secondary Structure
Globular Domains
Disorder Score
Coiled-Coil
Transmembrane
0
50
100
150
200
250

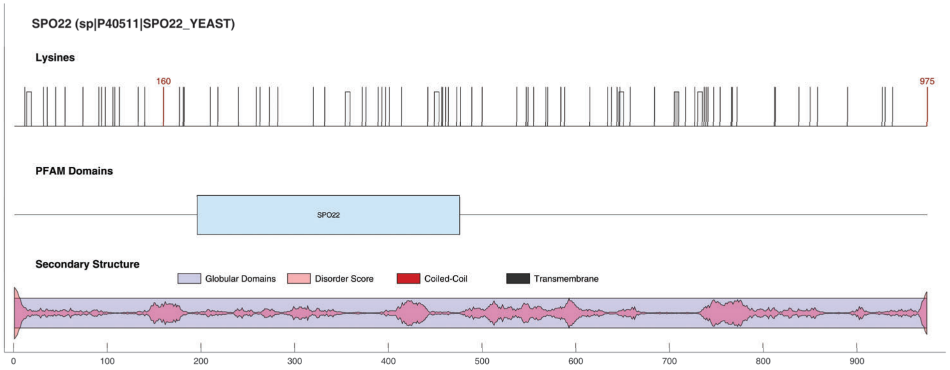
SPO22 (sp|P40511|SPO22_YEAST)
Lysines
160
975
PFAM Domains
SPO22
Secondary Structure
Globular Domains
Disorder Score
Coiled-Coil
Transmembrane
0
100
200
300
400
500
600
700
800
900

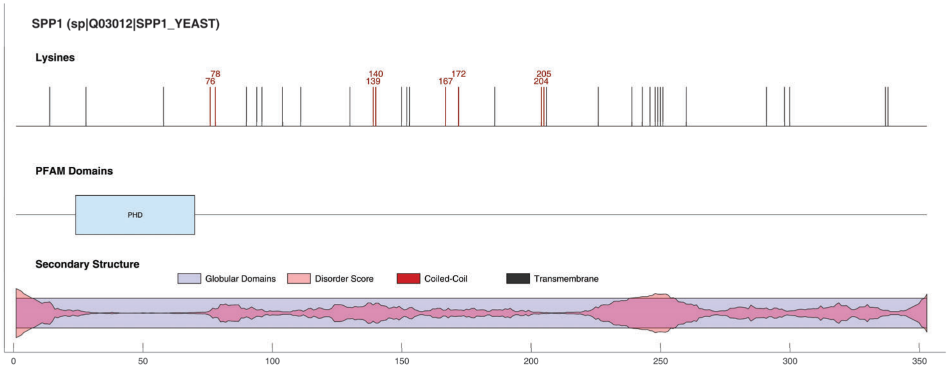
SPP1 (sp|Q03012|SPP1_YEAST)
Lysines
76
78
139
140
167
172
204
205
PFAM Domains
PHD
Secondary Structure
Globular Domains
Disorder Score
Coiled-Coil
Transmembrane
0
50
100
150
200
250
300
350

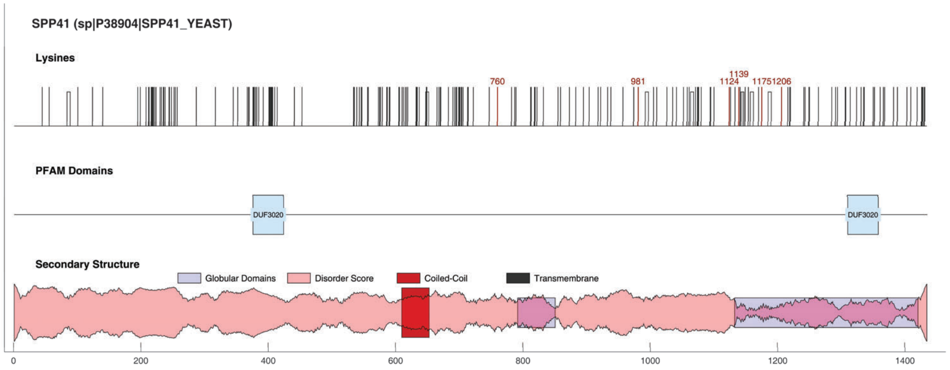
SPP41 (sp|P38904|SPP41_YEAST)
Lysines
760
981
1124
1139
11751206
PFAM Domains
DUF3020
DUF3020
Secondary Structure
Globular Domains
Disorder Score
Coiled-Coil
Transmembrane
0
200
400
600
800
1000
1200
1400

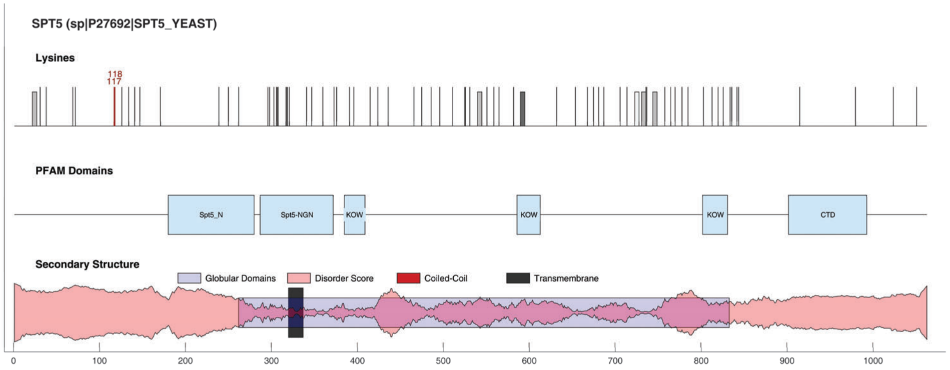
SPT5 (sp|P27692|SPT5_YEAST)
Lysines
118
117
PFAM Domains
Spt5_N
Spt5-NGN
KOW
KOW
KOW
CTD
Secondary Structure
Globular Domains
Disorder Score
Coiled-Coil
Transmembrane
0
100
200
300
400
500
600
700
800
900
1000

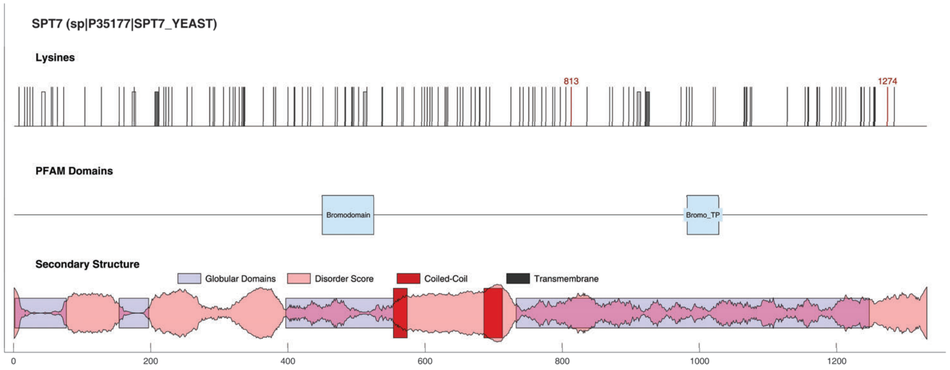
SPT7 (sp|P35177|SPT7_YEAST)
Lysines
813
1274
PFAM Domains
Bromodomain
Bromo_TP
Secondary Structure
Globular Domains
Disorder Score
Coiled-Coil
Transmembrane
0
200
400
600
800
1000
1200

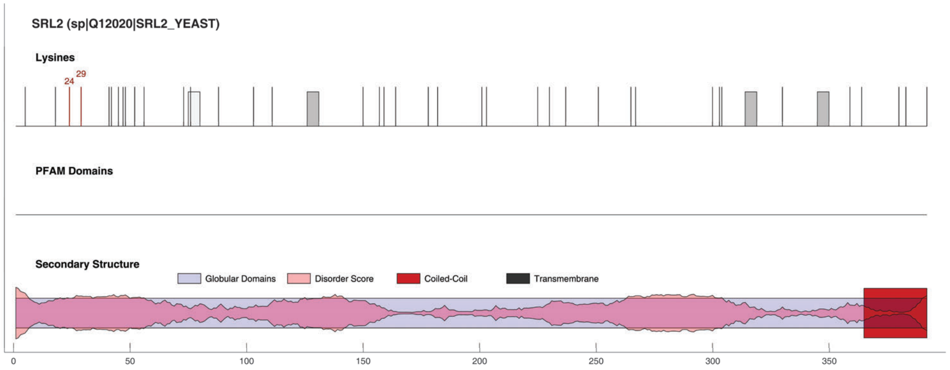
SRL2 (sp|Q12020|SRL2_YEAST)
Lysines
24
29
PFAM Domains
Secondary Structure
Globular Domains
Disorder Score
Coiled-Coil
Transmembrane
0
50
100
150
200
250
300
350

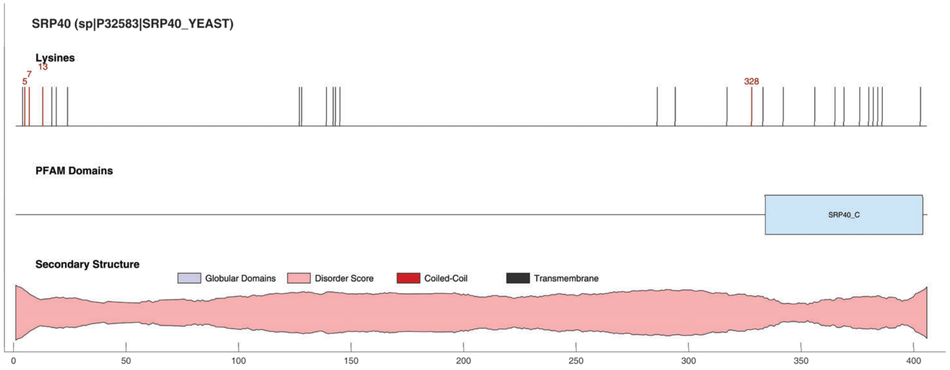
SRP40 (sp|P32583|SRP40_YEAST)
Lysines
5
7
13
328
PFAM Domains
SRP40_C
Secondary Structure
Globular Domains
Disorder Score
Coiled-Coil
Transmembrane
0
50
100
150
200
250
300
350
400

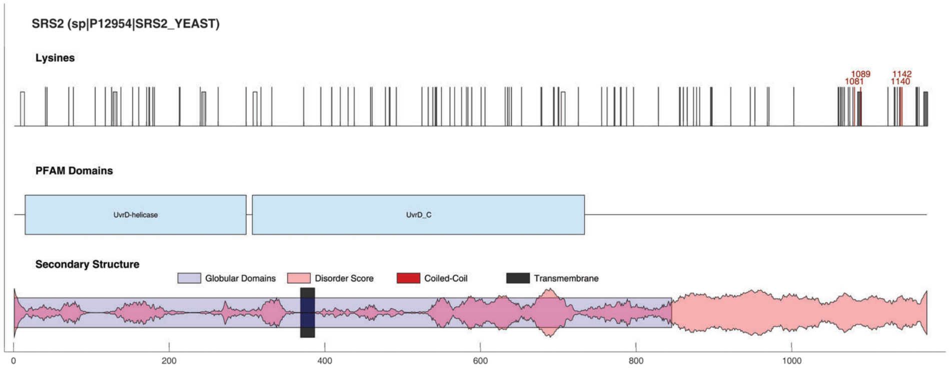
SRS2 (sp|P12954|SRS2_YEAST)
Lysines
1081
1089
1140
1142
PFAM Domains
UvrD-helicase
UvrD_C
Secondary Structure
Globular Domains
Disorder Score
Coiled-Coil
Transmembrane
0
200
400
600
800
1000

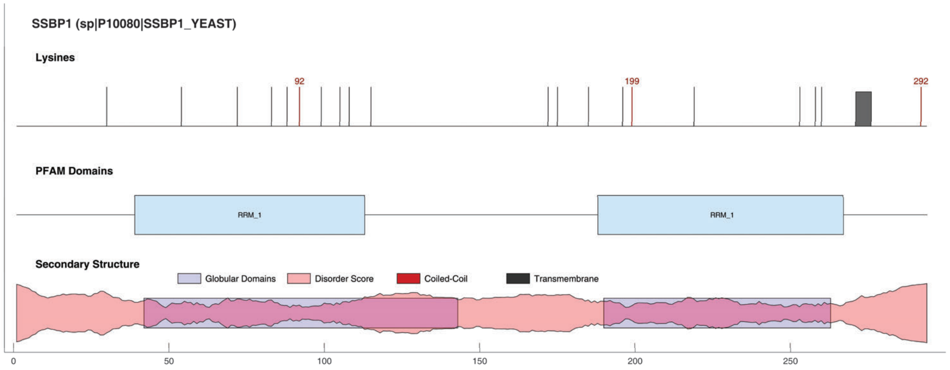
SSBP1 (sp|P10080|SSBP1_YEAST)
Lysines
92
199
292
PFAM Domains
RRM_1
RRM_1
Secondary Structure
Globular Domains
Disorder Score
Coiled-Coil
Transmembrane
0
50
100
150
200
250

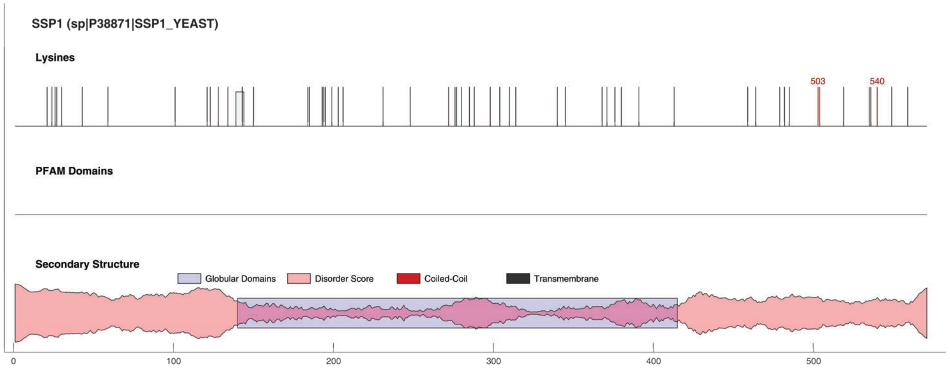
SSP1 (sp|P38871|SSP1_YEAST)
Lysines
503
540
PFAM Domains
Secondary Structure
Globular Domains
Disorder Score
Coiled-Coil
Transmembrane
0
100
200
300
400
500

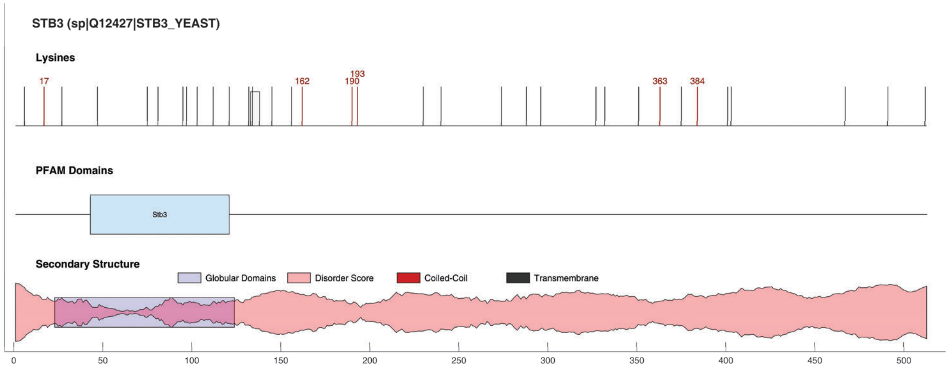
STB3 (sp|Q12427|STB3_YEAST)
Lysines
17
162
190
193
363
384
PFAM Domains
Stb3
Secondary Structure
Globular Domains
Disorder Score
Coiled-Coil
Transmembrane
0
50
100
150
200
250
300
350
400
450
500

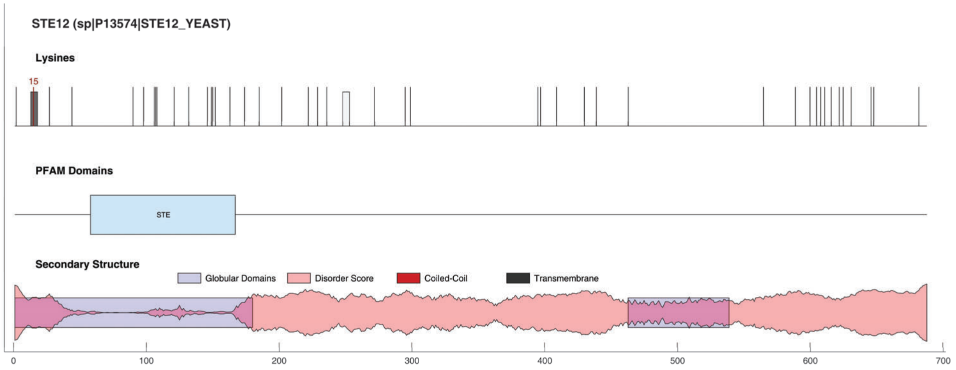
STE12 (sp|P13574|STE12_YEAST)
Lysines
15
PFAM Domains
STE
Secondary Structure
Globular Domains
Disorder Score
Coiled-Coil
Transmembrane
0
100
200
300
400
500
600
700

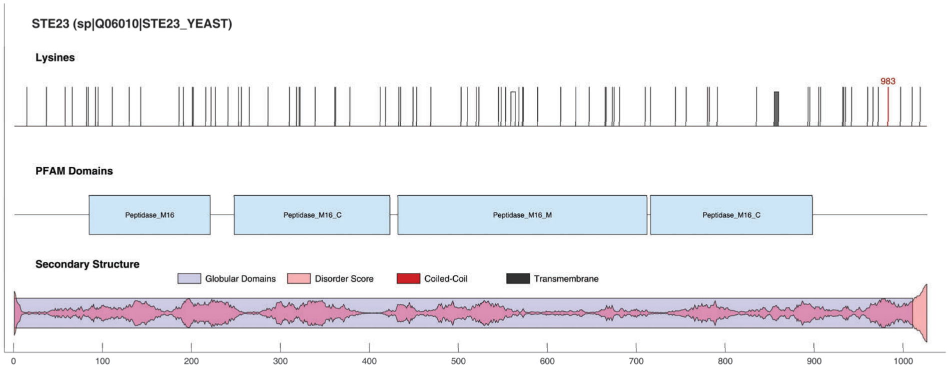
STE23 (sp|Q06010|STE23_YEAST)
Lysines
983
PFAM Domains
Peptidase_M16
Peptidase_M16_C
Peptidase_M16_M
Peptidase_M16_C
Secondary Structure
Globular Domains
Disorder Score
Coiled-Coil
Transmembrane
0
100
200
300
400
500
600
700
800
900
1000

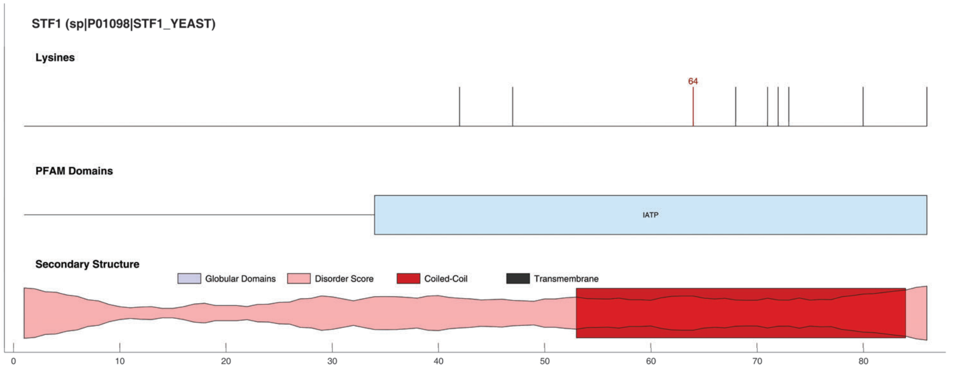
STF1 (sp|P01098|STF1_YEAST)
Lysines
64
PFAM Domains
IATP
Secondary Structure
Globular Domains
Disorder Score
Coiled-Coil
Transmembrane
0
10
20
30
40
50
60
70
80

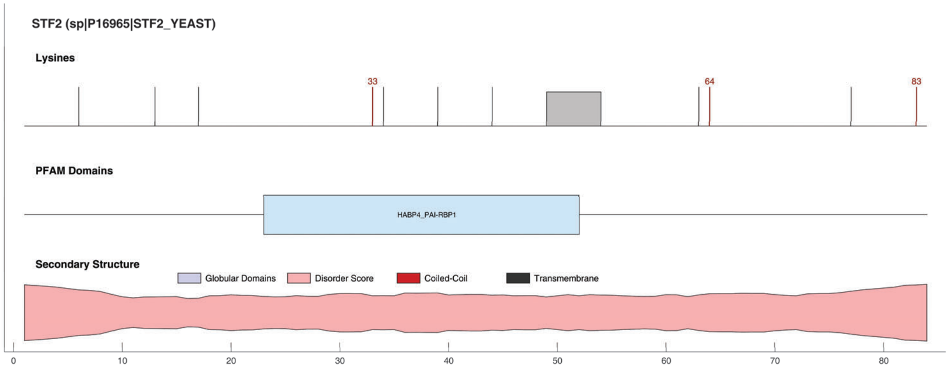
STF2 (sp|P16965|STF2_YEAST)
Lysines
33
64
83
PFAM Domains
HABP4_PAI-RBP1
Secondary Structure
Globular Domains
Disorder Score
Coiled-Coil
Transmembrane
0
10
20
30
40
50
60
70
80

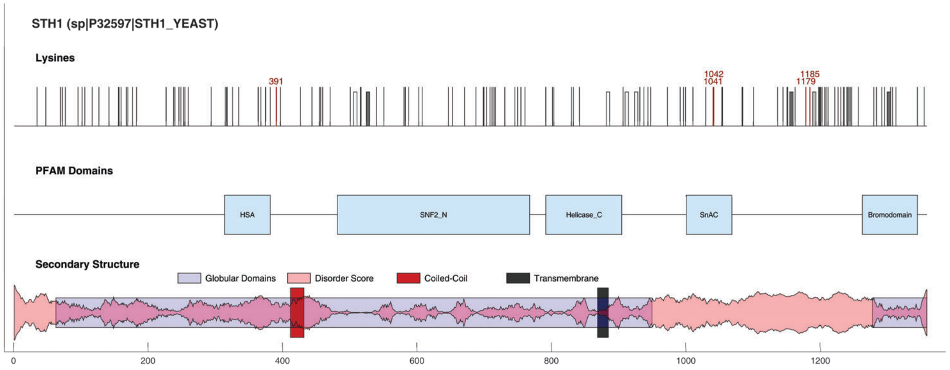
STH1 (sp|P32597|STH1_YEAST)
Lysines
391
1042
1041
1185
1179
PFAM Domains
HSA
SNF2_N
Helicase_C
SnAC
Bromodomain
Secondary Structure
Globular Domains
Disorder Score
Coiled-Coil
Transmembrane
0
200
400
600
800
1000
1200

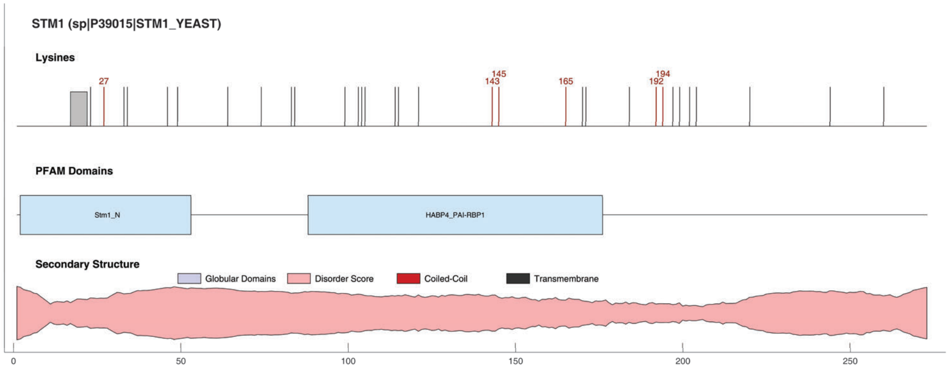
STM1 (sp|P39015|STM1_YEAST)
Lysines
27
145
143
165
194
192
PFAM Domains
Stm1_N
HABP4_PAI-RBP1
Secondary Structure
Globular Domains
Disorder Score
Coiled-Coil
Transmembrane
0
50
100
150
200
250

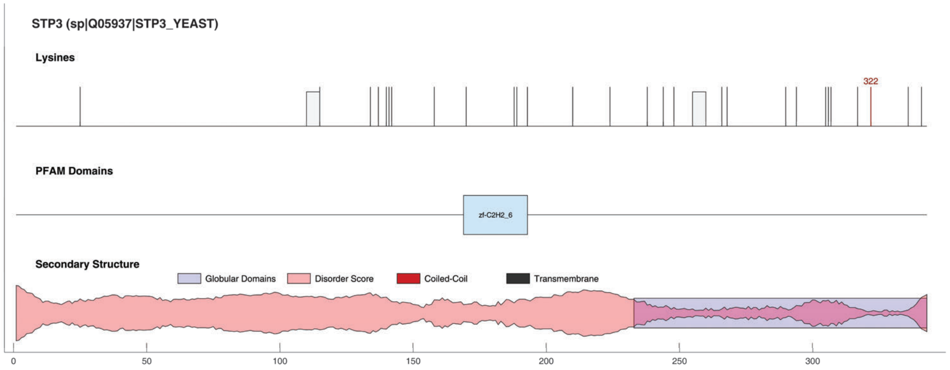
STP3 (sp|Q05937|STP3_YEAST)
Lysines
322
PFAM Domains
zf-C2H2_6
Secondary Structure
Globular Domains
Disorder Score
Coiled-Coil
Transmembrane
0
50
100
150
200
250
300

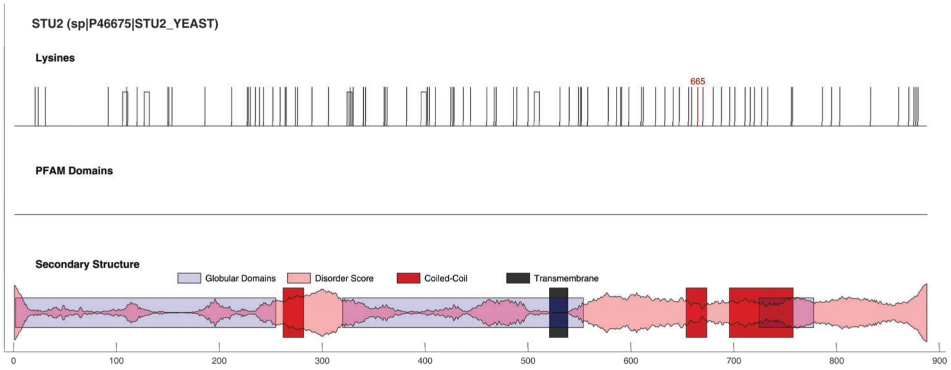

STU2 (sp|P46675|STU2_YEAST)
Lysines
665
PFAM Domains
Secondary Structure
Globular Domains
Disorder Score
Coiled-Coil
Transmembrane
0
100
200
300
400
500
600
700
800
900

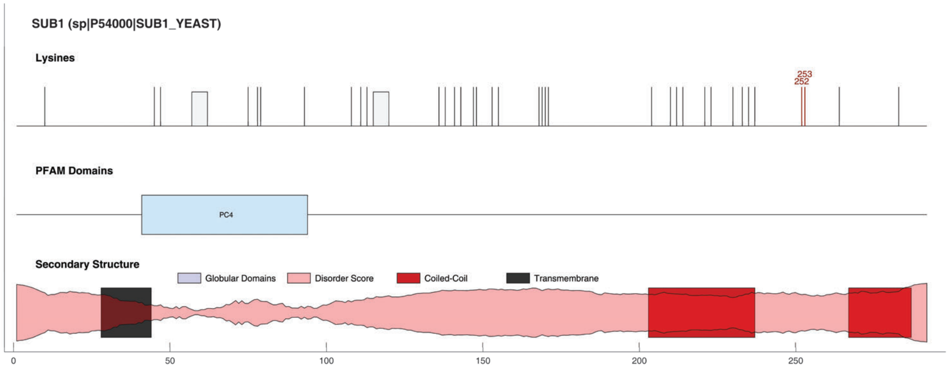

SUB1 (sp|P54000|SUB1_YEAST)
Lysines
253
252
PFAM Domains
PC4
Secondary Structure
Globular Domains
Disorder Score
Coiled-Coil
Transmembrane
0
50
100
150
200
250

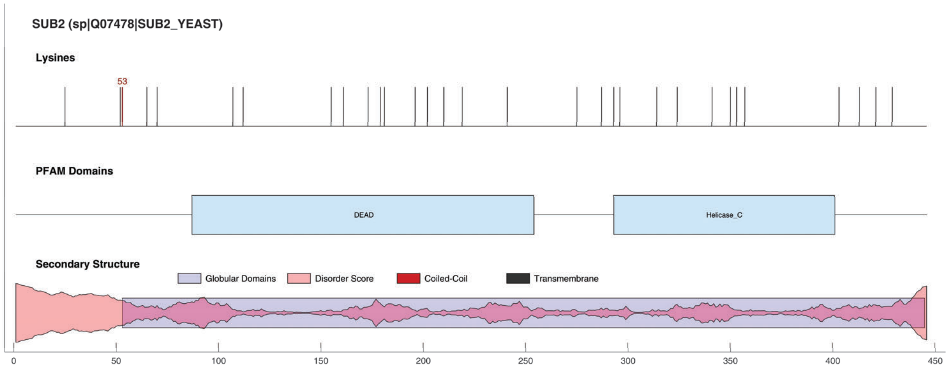

SUB2 (sp|Q07478|SUB2_YEAST)
Lysines
53
PFAM Domains
DEAD
Helicase_C
Secondary Structure
Globular Domains
Disorder Score
Coiled-Coil
Transmembrane
0
50
100
150
200
250
300
350
400
450

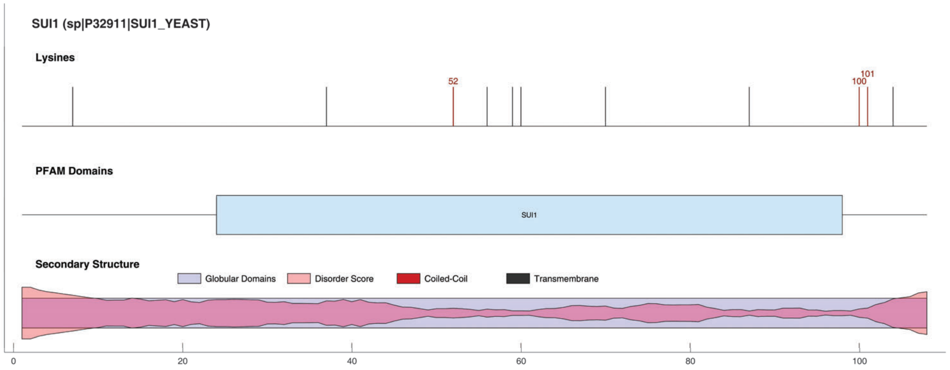

SUI1 (sp|P32911|SUI1_YEAST)
Lysines
52
100
101
PFAM Domains
SUI1
Secondary Structure
Globular Domains
Disorder Score
Coiled-Coil
Transmembrane
0
20
40
60
80
100

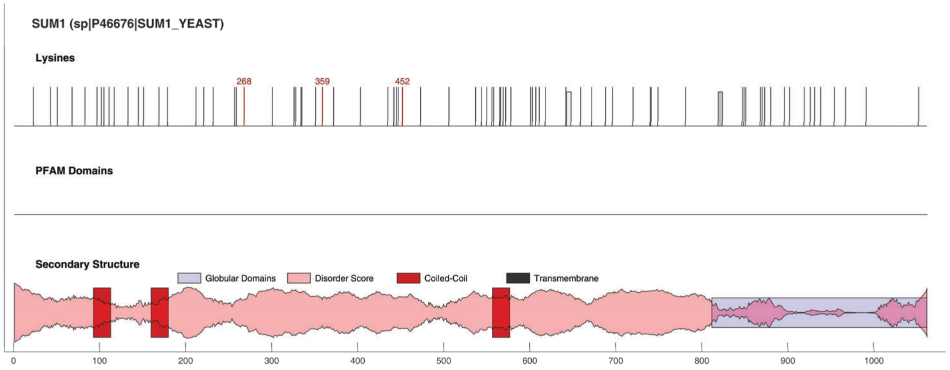
SUM1 (sp|P46676|SUM1_YEAST)
Lysines
268
359
452
PFAM Domains
Secondary Structure
Globular Domains
Disorder Score
Coiled-Coil
Transmembrane
0
100
200
300
400
500
600
700
800
900
1000

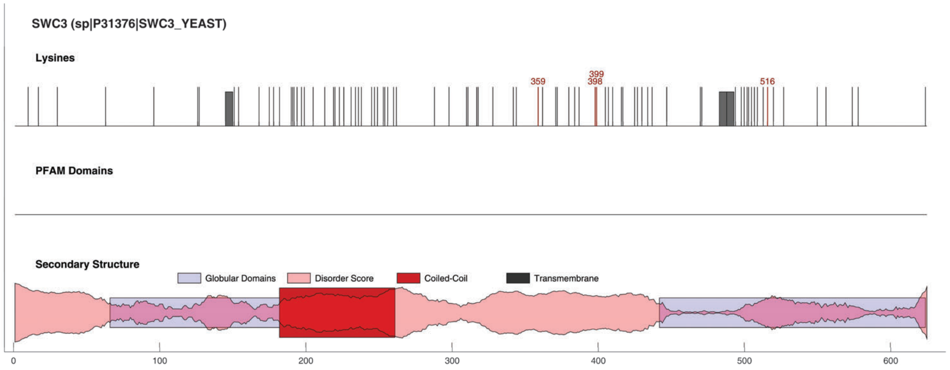
SWC3 (sp|P31376|SWC3_YEAST)
Lysines
359
399
398
516
PFAM Domains
Secondary Structure
Globular Domains
Disorder Score
Coiled-Coil
Transmembrane
0
100
200
300
400
500
600

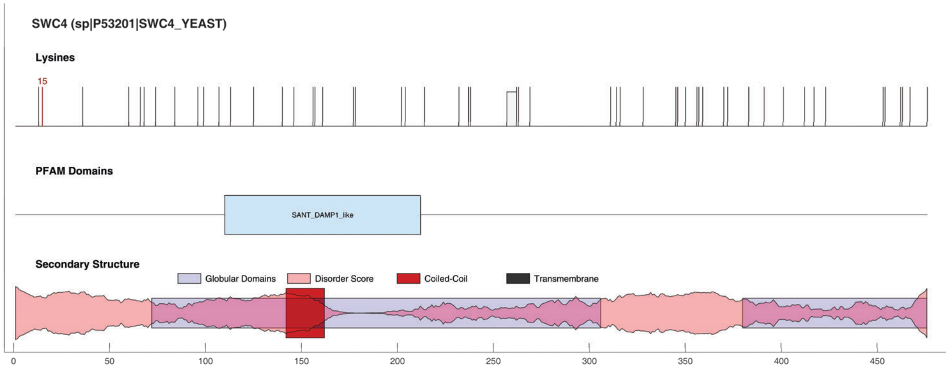
SWC4 (sp|P53201|SWC4_YEAST)
Lysines
15
PFAM Domains
SANT_DAMP1_like
Secondary Structure
Globular Domains
Disorder Score
Coiled-Coil
Transmembrane
0
50
100
150
200
250
300
350
400
450

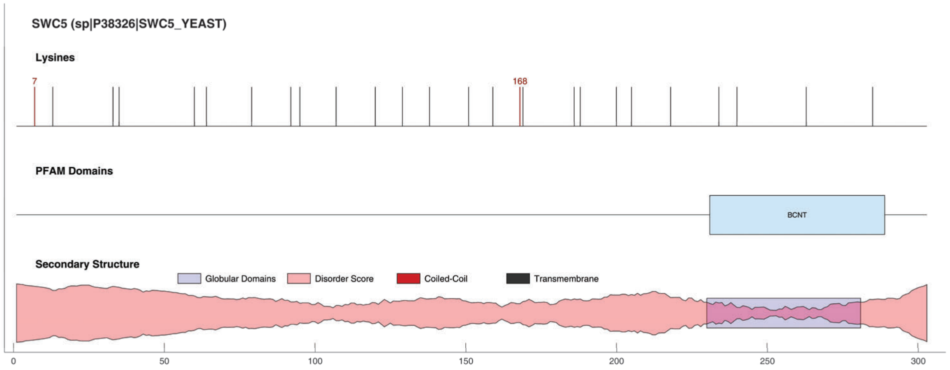
SWC5 (sp|P38326|SWC5_YEAST)
Lysines
7
168
PFAM Domains
BCNT
Secondary Structure
Globular Domains
Disorder Score
Coiled-Coil
Transmembrane
0
50
100
150
200
250
300

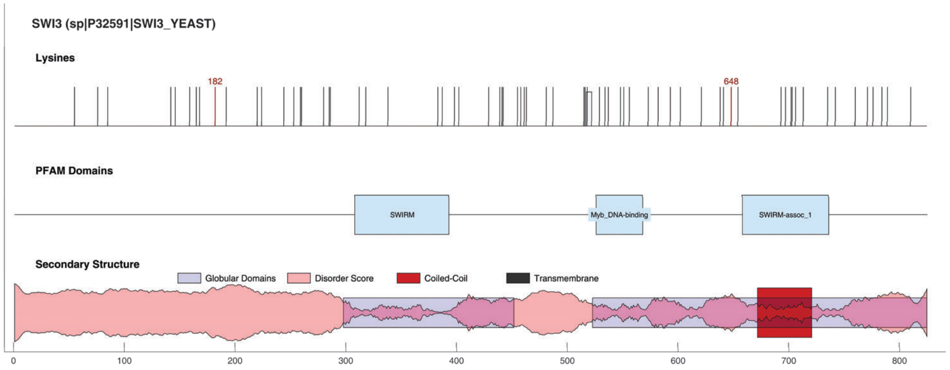
SWI3 (sp|P32591|SWI3_YEAST)
Lysines
182
648
PFAM Domains
SWIRM
Myb_DNA-binding
SWIRM-assoc_1
Secondary Structure
Globular Domains
Disorder Score
Coiled-Coil
Transmembrane
0
100
200
300
400
500
600
700
800

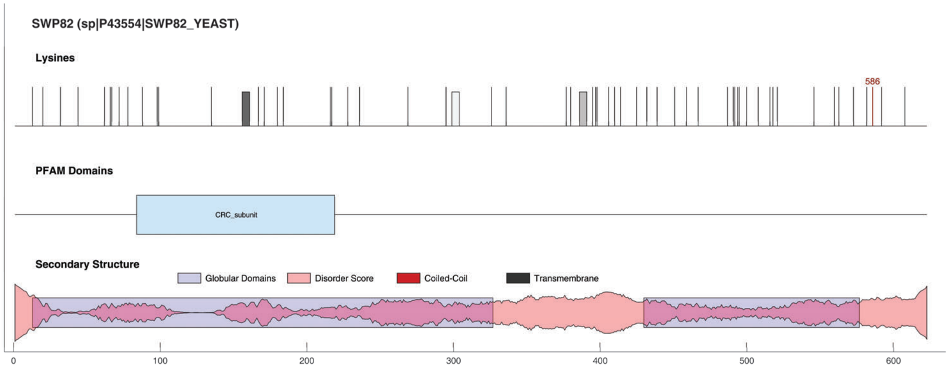
SWP82 (sp|P43554|SWP82_YEAST)
Lysines
586
PFAM Domains
CRC_subunit
Secondary Structure
Globular Domains
Disorder Score
Coiled-Coil
Transmembrane
0
100
200
300
400
500
600

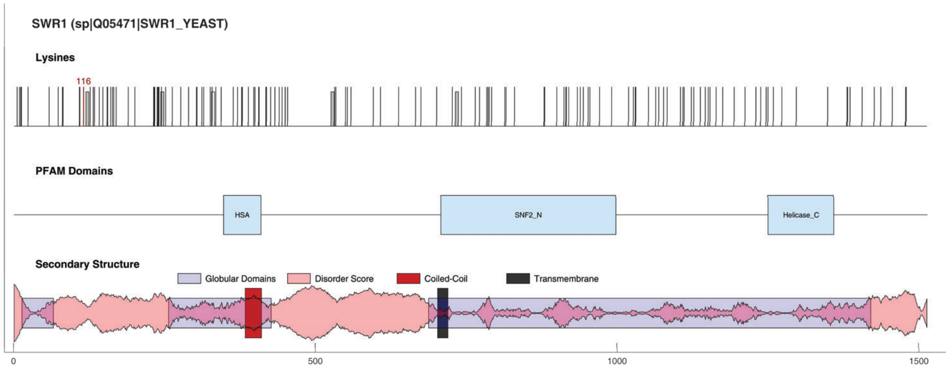
SWR1 (sp|Q05471|SWR1_YEAST)
Lysines
116
PFAM Domains
HSA
SNF2_N
Helicase_C
Secondary Structure
Globular Domains
Disorder Score
Coiled-Coil
Transmembrane
0
500
1000
1500

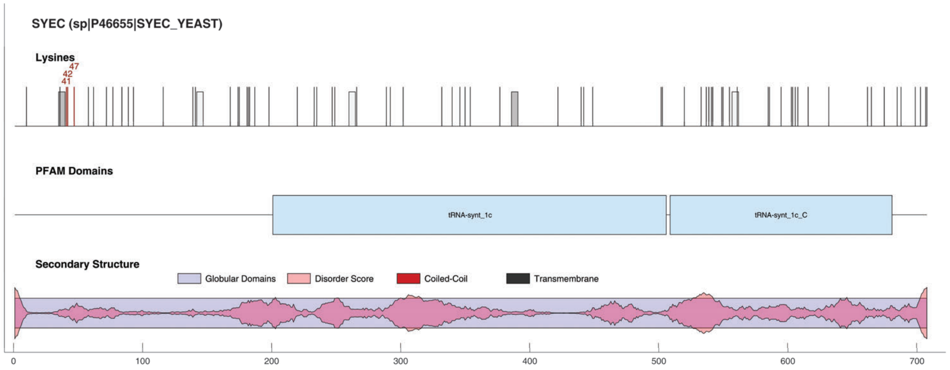
SYEC (sp|P46655|SYEC_YEAST)
Lysines
47
42
41
PFAM Domains
tRNA-synt_1c
tRNA-synt_1c_C
Secondary Structure
Globular Domains
Disorder Score
Coiled-Coil
Transmembrane
0
100
200
300
400
500
600
700

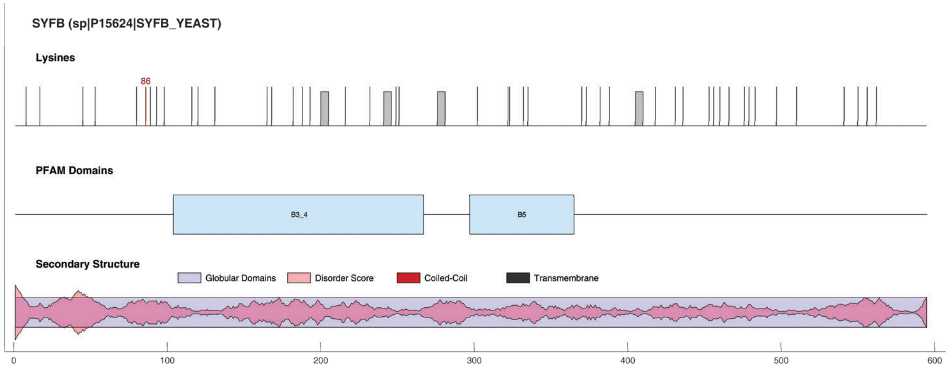
SYFB (sp|P15624|SYFB_YEAST)
Lysines
86
PFAM Domains
B3_4
B5
Secondary Structure
Globular Domains
Disorder Score
Coiled-Coil
Transmembrane
0
100
200
300
400
500
600

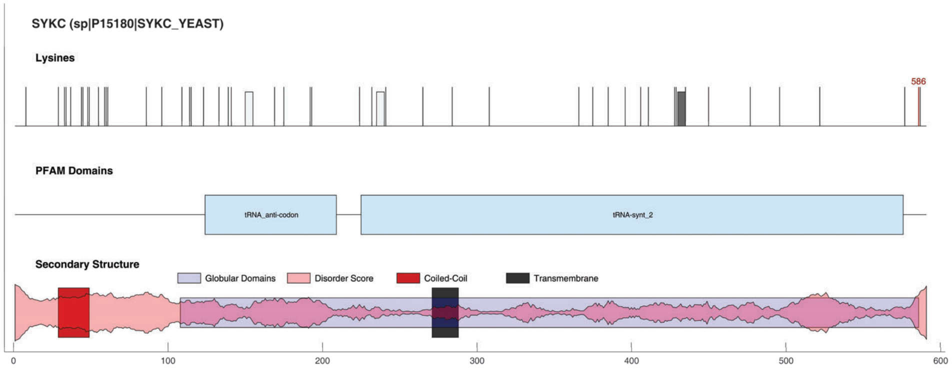
SYKC (sp|P15180|SYKC_YEAST)
Lysines
586
PFAM Domains
tRNA_anti-codon
tRNA-synt_2
Secondary Structure
Globular Domains
Disorder Score
Coiled-Coil
Transmembrane
0
100
200
300
400
500
600

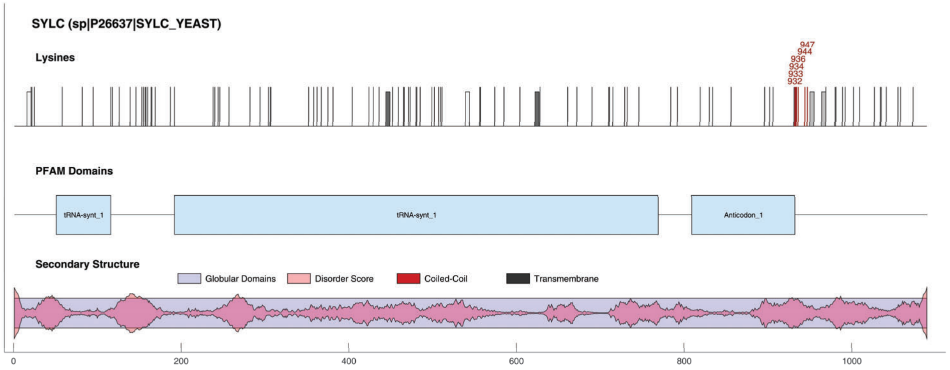
SYLC (sp|P26637|SYLC_YEAST)
Lysines
947
944
936
934
933
932
PFAM Domains
tRNA-synt_1
tRNA-synt_1
Anticodon_1
Secondary Structure
Globular Domains
Disorder Score
Coiled-Coil
Transmembrane
0
200
400
600
800
1000

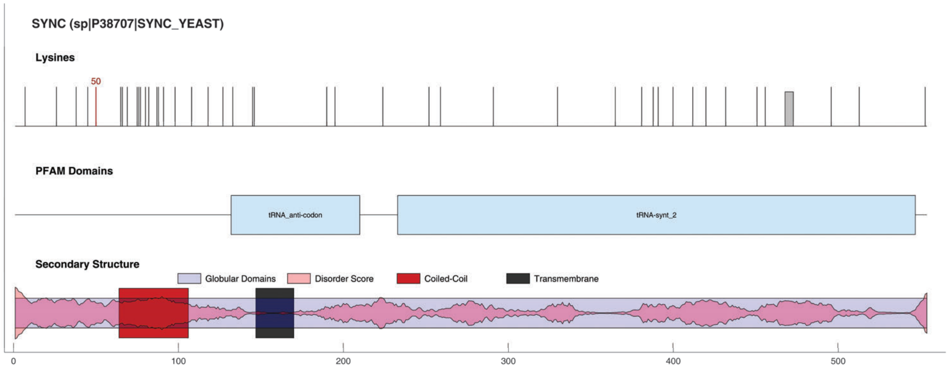
SYNC (sp|P38707|SYNC_YEAST)
Lysines
50
PFAM Domains
tRNA_anti-codon
tRNA-synt_2
Secondary Structure
Globular Domains
Disorder Score
Coiled-Coil
Transmembrane
0
100
200
300
400
500

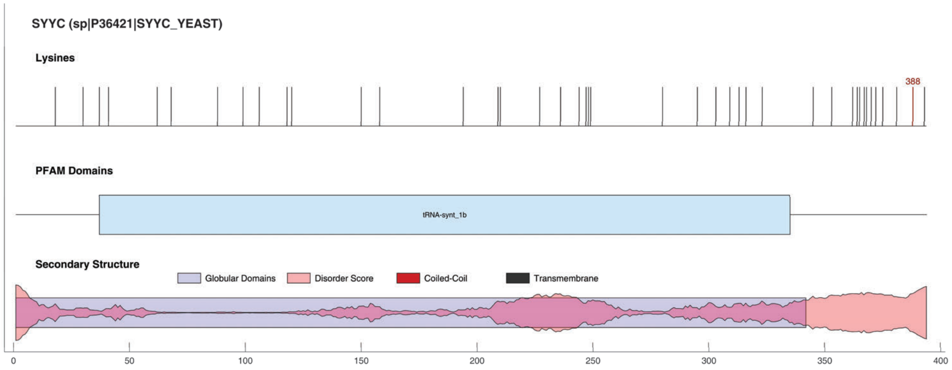
SYYC (sp|P36421|SYYC_YEAST)
Lysines
388
PFAM Domains
tRNA-synt_1b
Secondary Structure
Globular Domains
Disorder Score
Coiled-Coil
Transmembrane
0
50
100
150
200
250
300
350
400

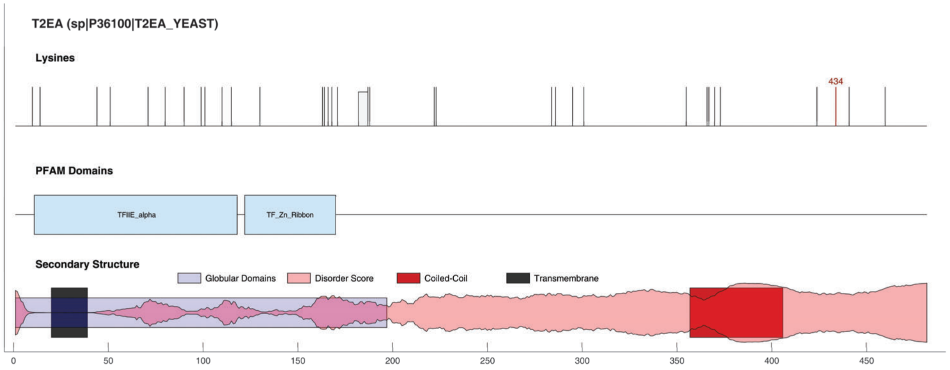
T2EA (sp|P36100|T2EA_YEAST)
Lysines
434
PFAM Domains
TFIIE_alpha
TF_Zn_Ribbon
Secondary Structure
Globular Domains
Disorder Score
Coiled-Coil
Transmembrane
0
50
100
150
200
250
300
350
400
450

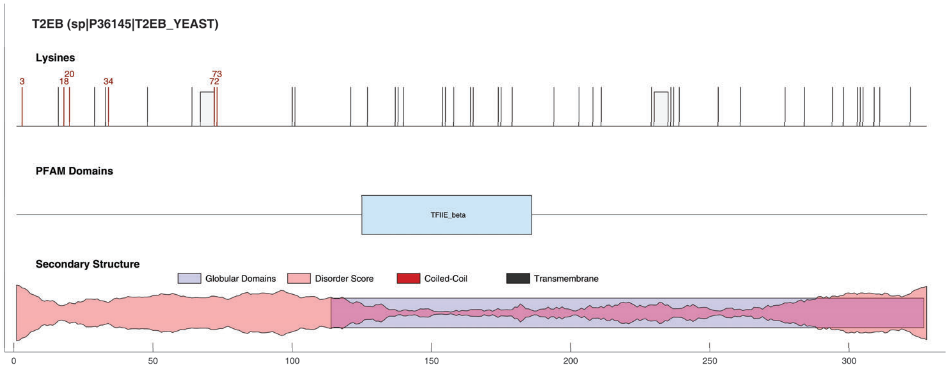
T2EB (sp|P36145|T2EB_YEAST)
Lysines
3
18
20
34
72
73
PFAM Domains
TFIIE_beta
Secondary Structure
Globular Domains
Disorder Score
Coiled-Coil
Transmembrane
0
50
100
150
200
250
300

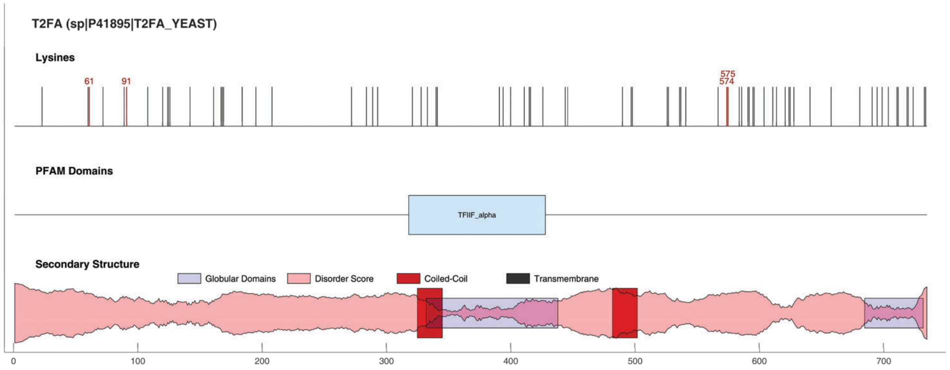
T2FA (sp|P41895|T2FA_YEAST)
Lysines
61
91
574
575
PFAM Domains
TFIIF_alpha
Secondary Structure
Globular Domains
Disorder Score
Coiled-Coil
Transmembrane
0
100
200
300
400
500
600
700

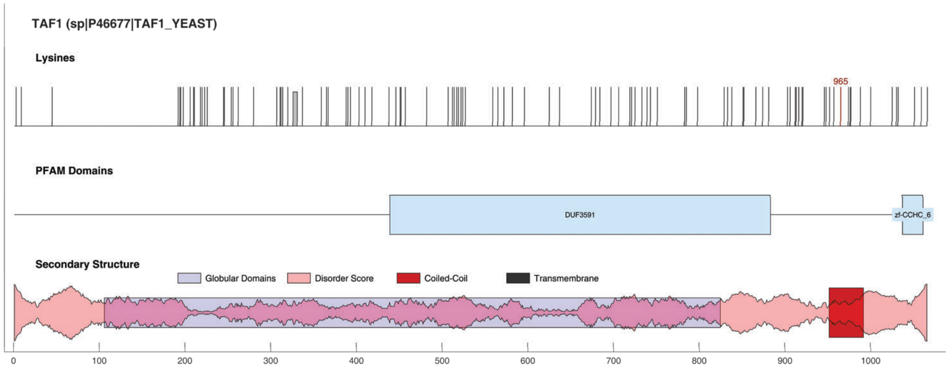
TAF1 (sp|P46677|TAF1_YEAST)
Lysines
965
PFAM Domains
DUF3591
zf-CCHC_6
Secondary Structure
Globular Domains
Disorder Score
Coiled-Coil
Transmembrane
0
100
200
300
400
500
600
700
800
900
1000

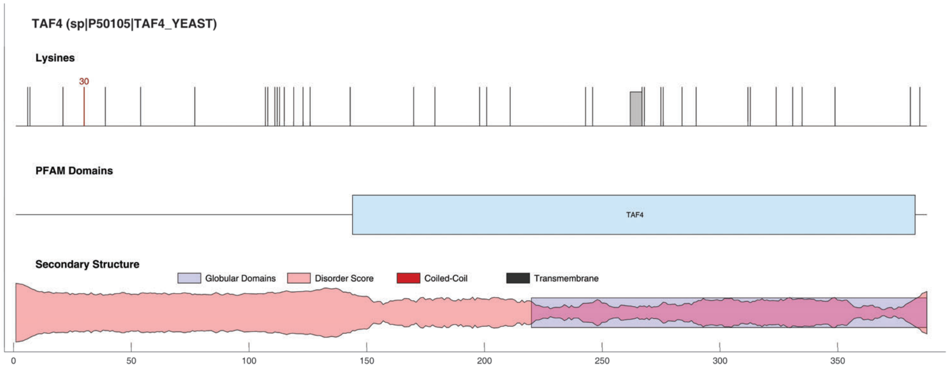
TAF4 (sp|P50105|TAF4_YEAST)
Lysines
30
PFAM Domains
TAF4
Secondary Structure
Globular Domains
Disorder Score
Coiled-Coil
Transmembrane
0
50
100
150
200
250
300
350

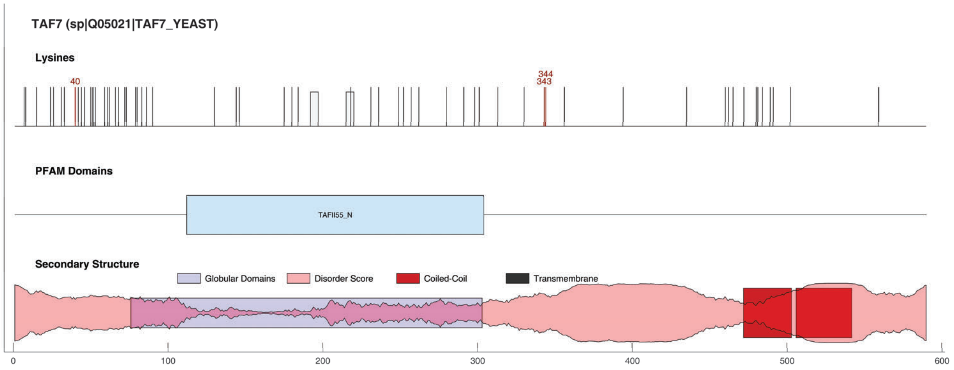
TAF7 (sp|Q05021|TAF7_YEAST)
Lysines
40
344
343
PFAM Domains
TAFII55_N
Secondary Structure
Globular Domains
Disorder Score
Coiled-Coil
Transmembrane
0
100
200
300
400
500
600

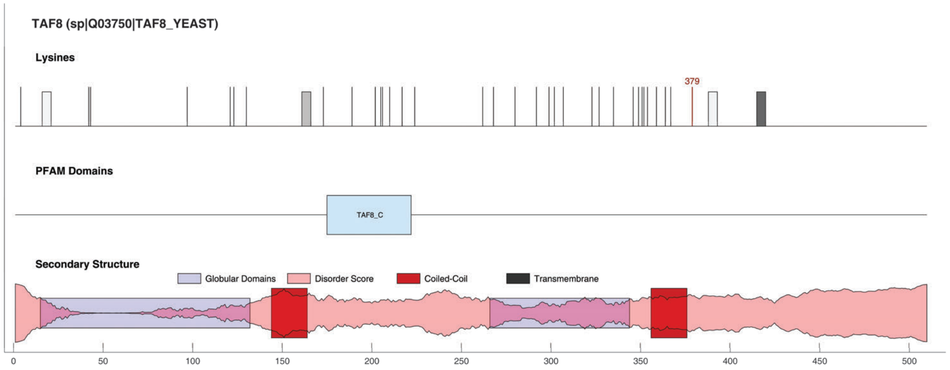
TAF8 (sp|Q03750|TAF8_YEAST)
Lysines
379
PFAM Domains
TAF8_C
Secondary Structure
Globular Domains
Disorder Score
Coiled-Coil
Transmembrane
0
50
100
150
200
250
300
350
400
450
500

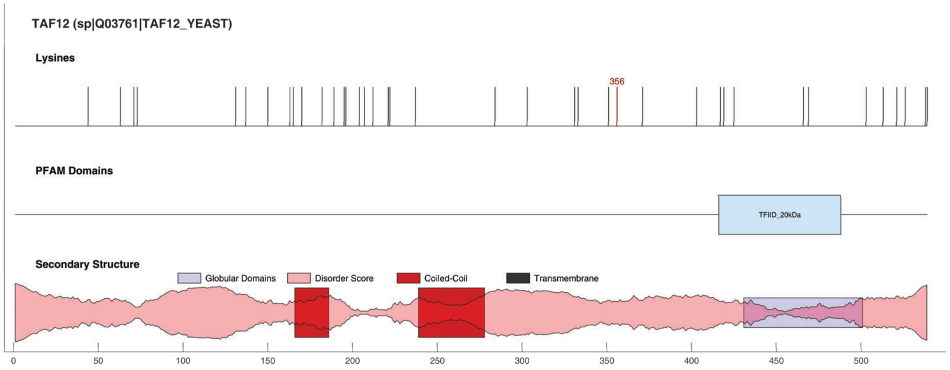
TAF12 (sp|Q03761|TAF12_YEAST)
Lysines
356
PFAM Domains
TFIID_20kDa
Secondary Structure
Globular Domains
Disorder Score
Coiled-Coil
Transmembrane
0
50
100
150
200
250
300
350
400
450
500

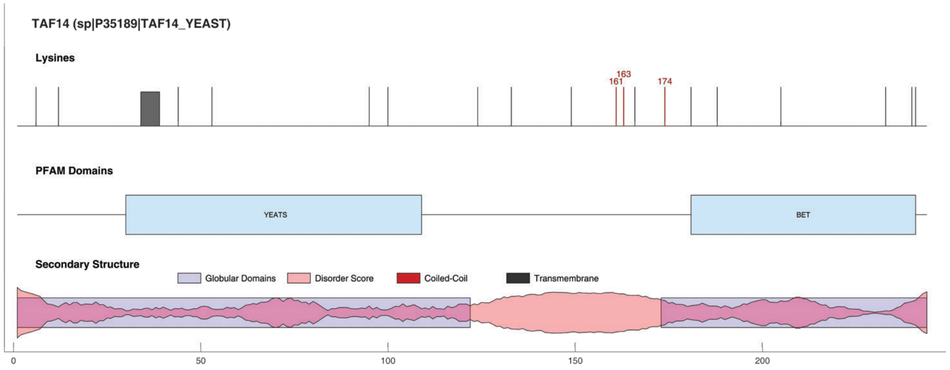
TAF14 (sp|P35189|TAF14_YEAST)
Lysines
163
161
174
PFAM Domains
YEATS
BET
Secondary Structure
Globular Domains
Disorder Score
Coiled-Coil
Transmembrane
0
50
100
150
200

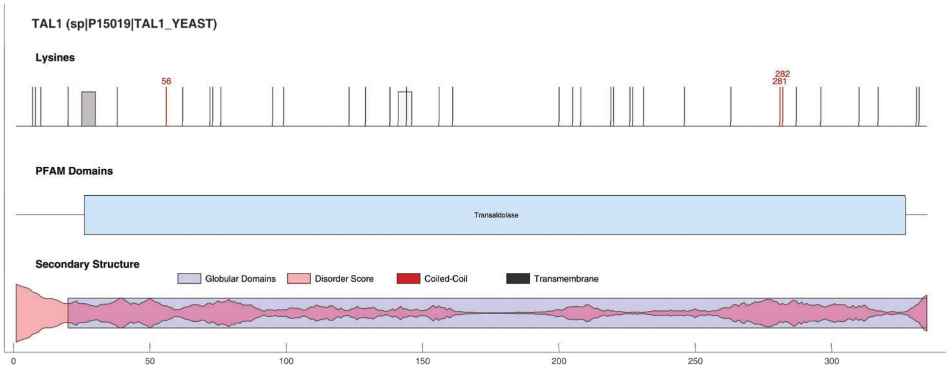
TAL1 (sp|P15019|TAL1_YEAST)
Lysines
56
282
281
PFAM Domains
Transaldolase
Secondary Structure
Globular Domains
Disorder Score
Coiled-Coil
Transmembrane
0
50
100
150
200
250
300

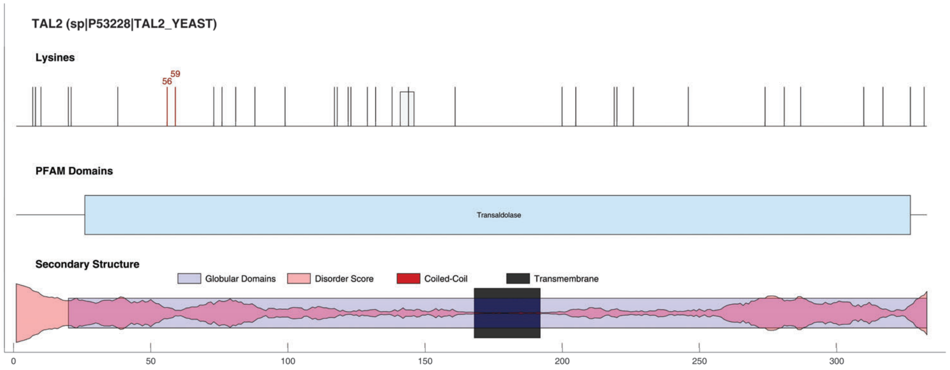
TAL2 (sp|P53228|TAL2_YEAST)
Lysines
59
56
PFAM Domains
Transaldolase
Secondary Structure
Globular Domains
Disorder Score
Coiled-Coil
Transmembrane
0
50
100
150
200
250
300

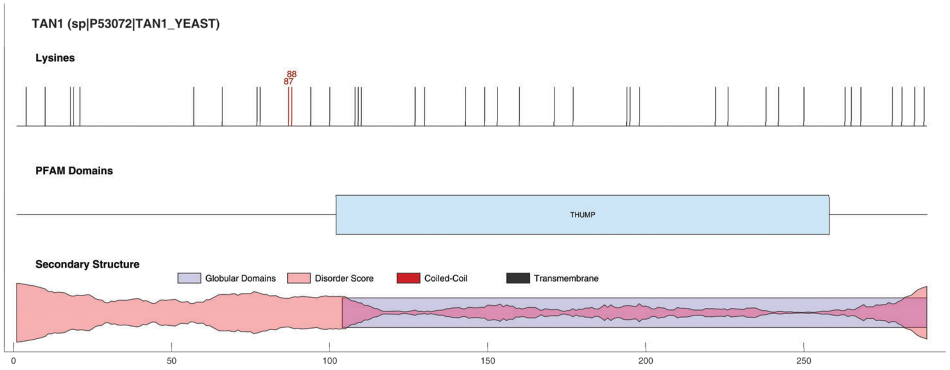
TAN1 (sp|P53072|TAN1_YEAST)
Lysines
88
87
PFAM Domains
THUMP
Secondary Structure
Globular Domains
Disorder Score
Coiled-Coil
Transmembrane
0
50
100
150
200
250

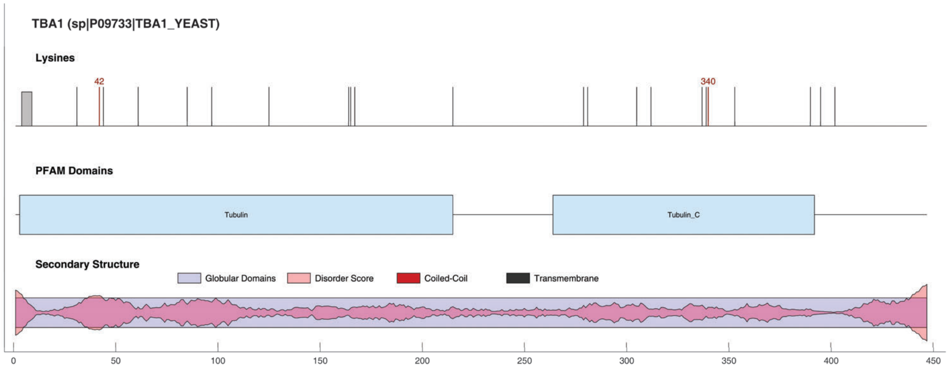
TBA1 (sp|P09733|TBA1_YEAST)
Lysines
42
340
PFAM Domains
Tubulin
Tubulin_C
Secondary Structure
Globular Domains
Disorder Score
Coiled-Coil
Transmembrane
0
50
100
150
200
250
300
350
400
450

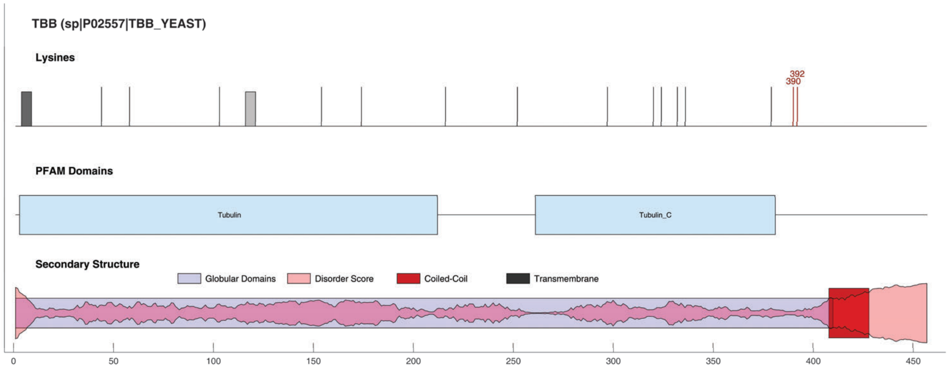
TBB (sp|P02557|TBB_YEAST)
Lysines
392
390
PFAM Domains
Tubulin
Tubulin_C
Secondary Structure
Globular Domains
Disorder Score
Coiled-Coil
Transmembrane
0
50
100
150
200
250
300
350
400
450

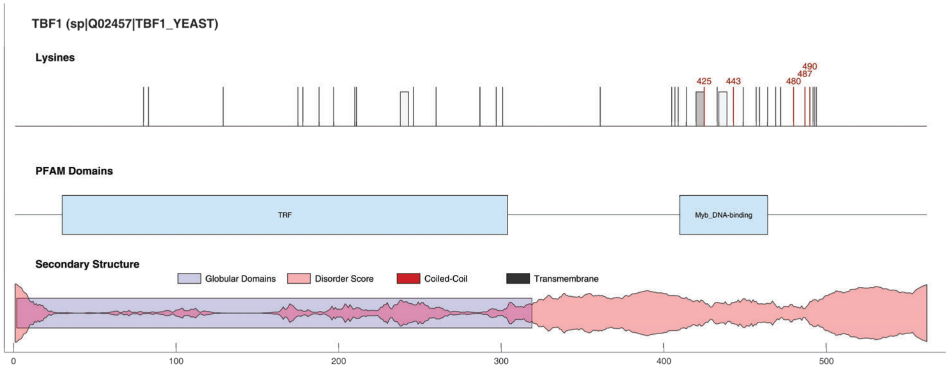
TBF1 (sp|Q02457|TBF1_YEAST)
Lysines
425
443
480
487
490
PFAM Domains
TRF
Myb_DNA-binding
Secondary Structure
Globular Domains
Disorder Score
Coiled-Coil
Transmembrane
0
100
200
300
400
500

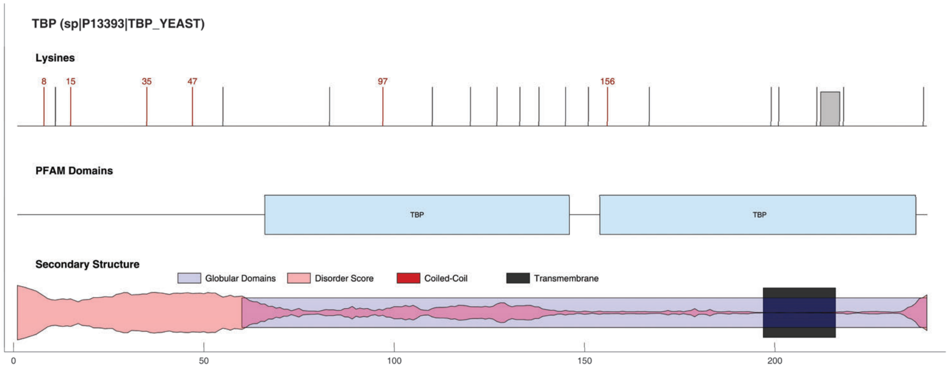

TBP (sp|P13393|TBP_YEAST)
Lysines
8
15
35
47
97
156
PFAM Domains
TBP
TBP
Secondary Structure
Globular Domains
Disorder Score
Coiled-Coil
Transmembrane
0
50
100
150
200

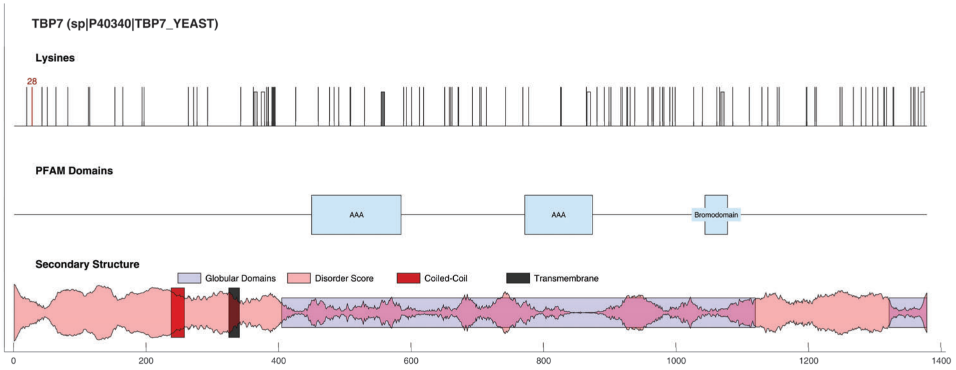

TBP7 (sp|P40340|TBP7_YEAST)
Lysines
28
PFAM Domains
AAA
AAA
Bromodomain
Secondary Structure
Globular Domains
Disorder Score
Coiled-Coil
Transmembrane
0
200
400
600
800
1000
1200
1400

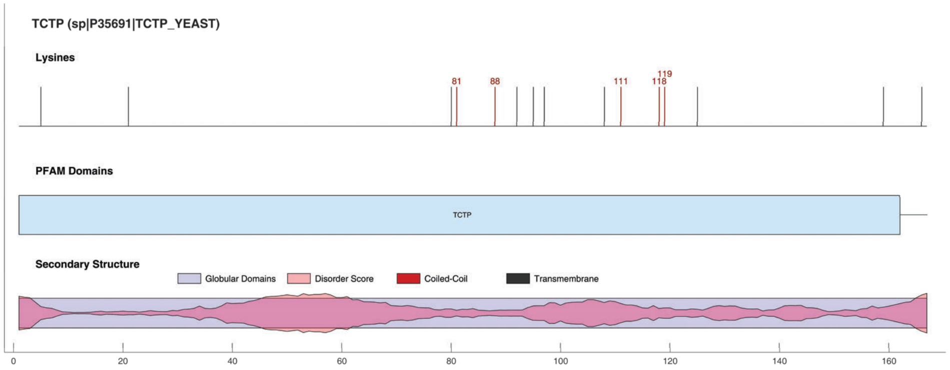

TCTP (sp|P35691|TCTP_YEAST)
Lysines
81
88
111
118
119
PFAM Domains
TCTP
Secondary Structure
Globular Domains
Disorder Score
Coiled-Coil
Transmembrane
0
20
40
60
80
100
120
140
160

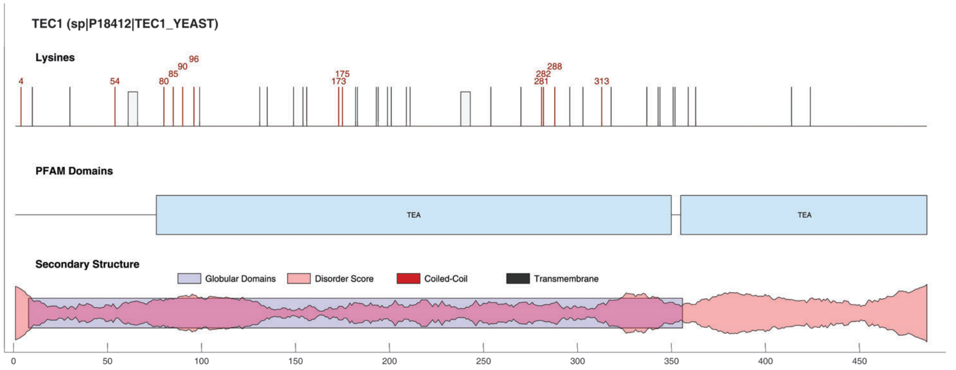

TEC1 (sp|P18412|TEC1_YEAST)
Lysines
4
54
80
85
90
96
173
175
281
282
288
313
PFAM Domains
TEA
TEA
Secondary Structure
Globular Domains
Disorder Score
Coiled-Coil
Transmembrane
0
50
100
150
200
250
300
350
400
450

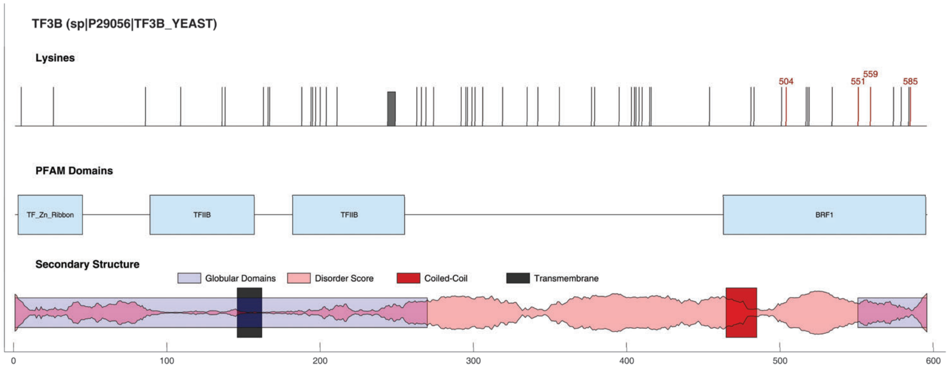
TF3B (sp|P29056|TF3B_YEAST)
Lysines
504
551
559
585
PFAM Domains
TF_Zn_Ribbon
TFIIB
TFIIB
BRF1
Secondary Structure
Globular Domains
Disorder Score
Coiled-Coil
Transmembrane
0
100
200
300
400
500
600

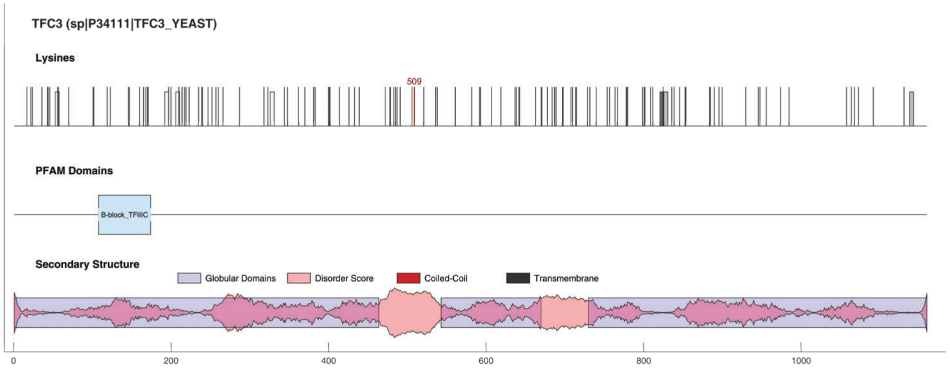
TFC3 (sp|P34111|TFC3_YEAST)
Lysines
509
PFAM Domains
B-block_TFIIIC
Secondary Structure
Globular Domains
Disorder Score
Coiled-Coil
Transmembrane
0
200
400
600
800
1000

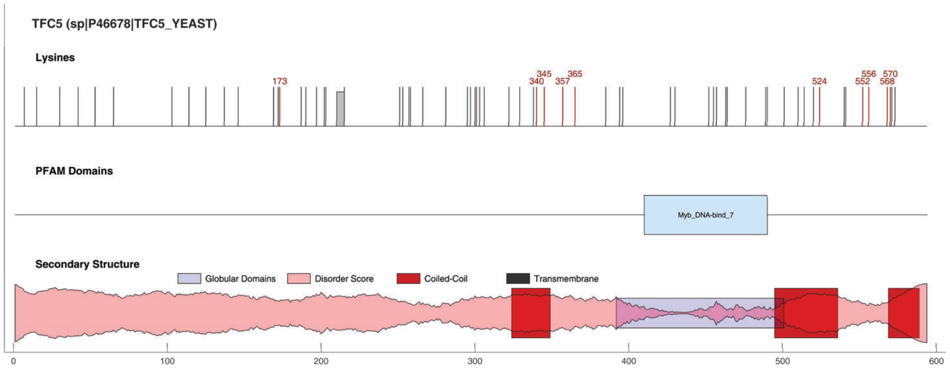
TFC5 (sp|P46678|TFC5_YEAST)
Lysines
173
340
345
357
365
524
552
556
568
570
PFAM Domains
Myb_DNA-bind_7
Secondary Structure
Globular Domains
Disorder Score
Coiled-Coil
Transmembrane
0
100
200
300
400
500
600

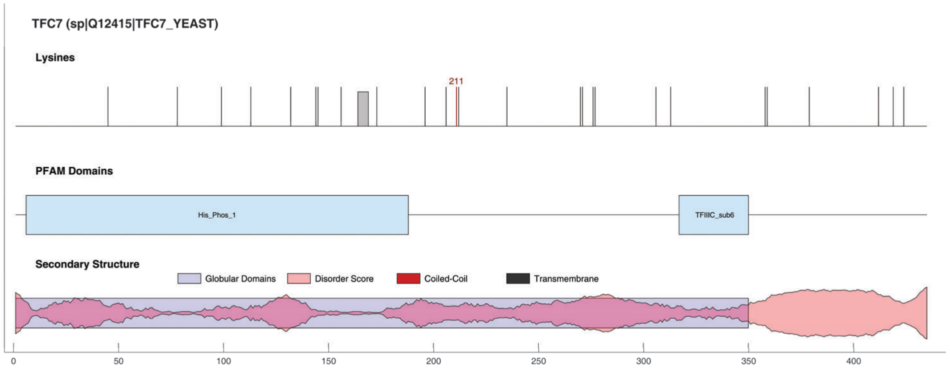
TFC7 (sp|Q12415|TFC7_YEAST)
Lysines
211
PFAM Domains
His_Phos_1
TFIIIC_sub6
Secondary Structure
Globular Domains
Disorder Score
Coiled-Coil
Transmembrane
0
50
100
150
200
250
300
350
400

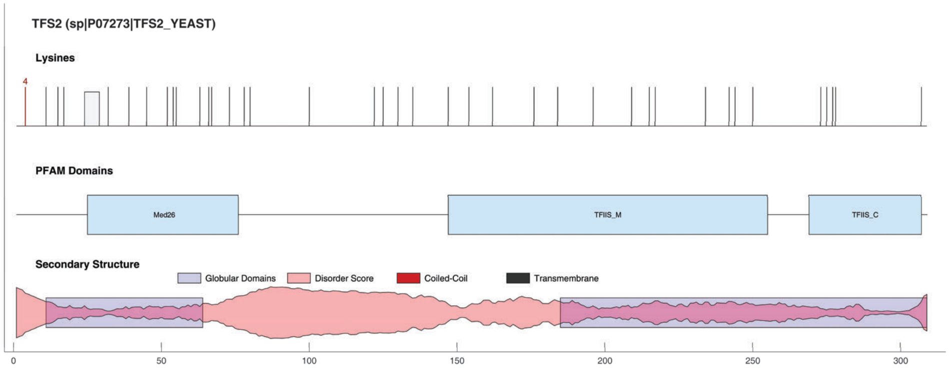
TFS2 (sp|P07273|TFS2_YEAST)
Lysines
4
PFAM Domains
Med26
TFIIS_M
TFIIS_C
Secondary Structure
Globular Domains
Disorder Score
Coiled-Coil
Transmembrane
0
50
100
150
200
250
300

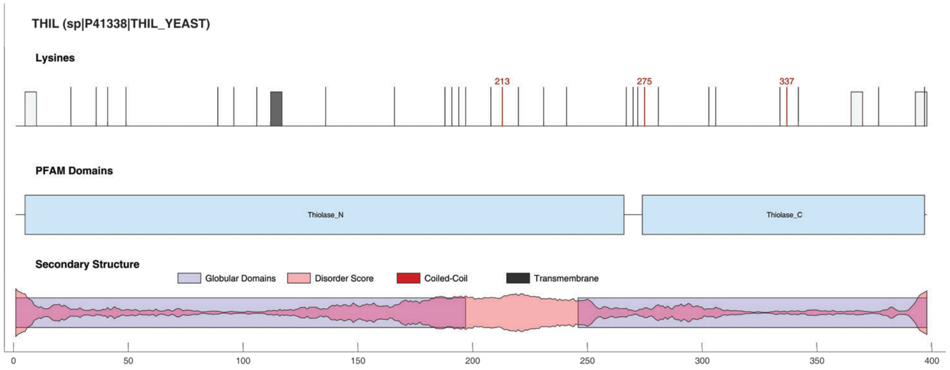
THIL (sp|P41338|THIL_YEAST)
Lysines
213
275
337
PFAM Domains
Thiolase_N
Thiolase_C
Secondary Structure
Globular Domains
Disorder Score
Coiled-Coil
Transmembrane
0
50
100
150
200
250
300
350
400

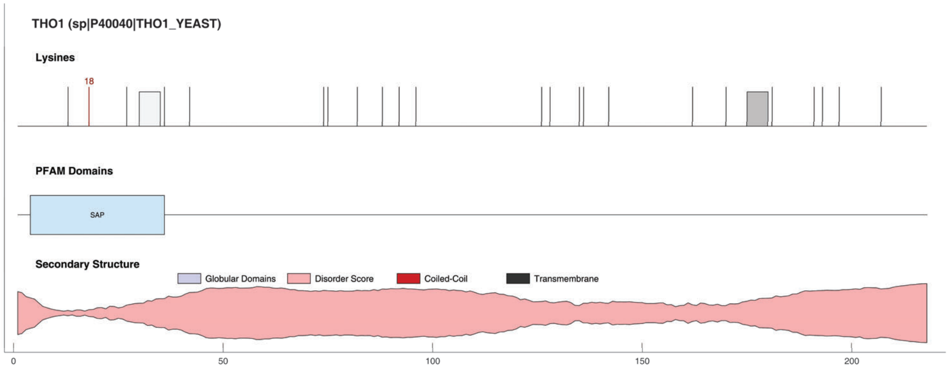
THO1 (sp|P40040|THO1_YEAST)
Lysines
18
PFAM Domains
SAP
Secondary Structure
Globular Domains
Disorder Score
Coiled-Coil
Transmembrane
0
50
100
150
200

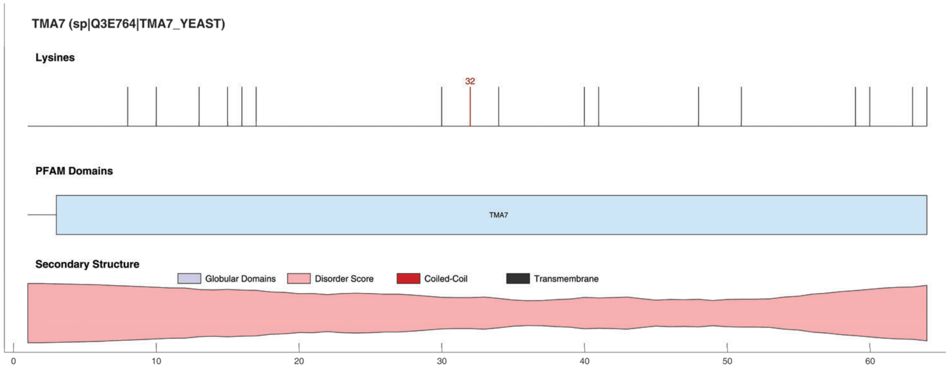
TMA7 (sp|Q3E764|TMA7_YEAST)
Lysines
32
PFAM Domains
TMA7
Secondary Structure
Globular Domains
Disorder Score
Coiled-Coil
Transmembrane
0
10
20
30
40
50
60

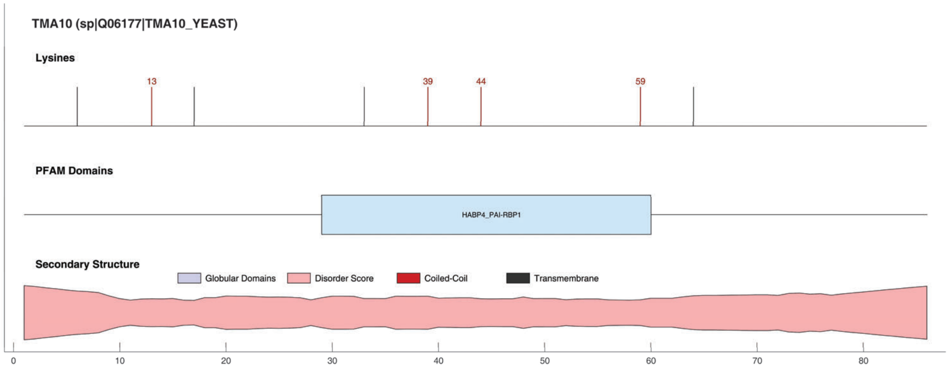
TMA10 (sp|Q06177|TMA10_YEAST)
Lysines
13
39
44
59
PFAM Domains
HABP4_PAI-RBP1
Secondary Structure
Globular Domains
Disorder Score
Coiled-Coil
Transmembrane
0
10
20
30
40
50
60
70
80

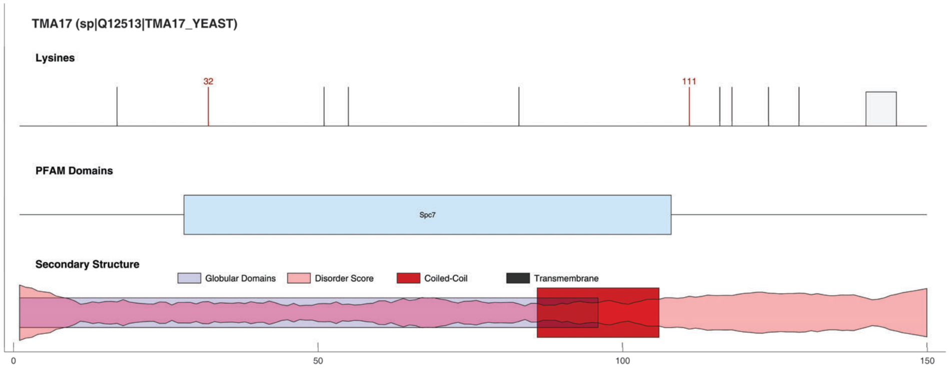
TMA17 (sp|Q12513|TMA17_YEAST)
Lysines
32
111
PFAM Domains
Spc7
Secondary Structure
Globular Domains
Disorder Score
Coiled-Coil
Transmembrane
0
50
100
150

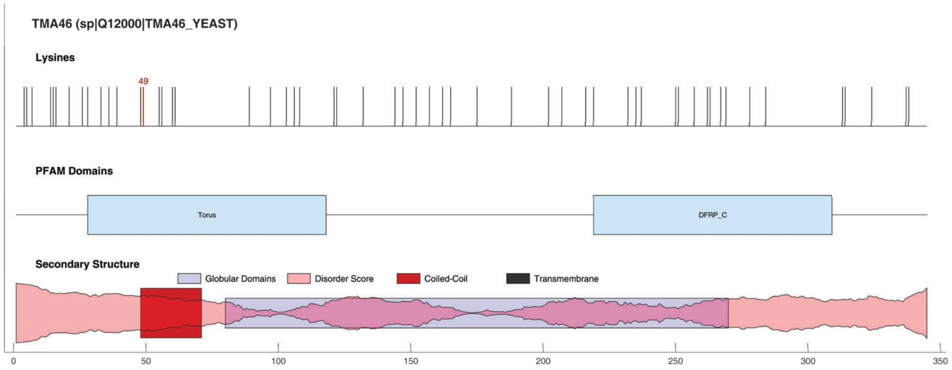
TMA46 (sp|Q12000|TMA46_YEAST)
Lysines
49
PFAM Domains
Torus
DFRP_C
Secondary Structure
Globular Domains
Disorder Score
Coiled-Coil
Transmembrane
0
50
100
150
200
250
300
350

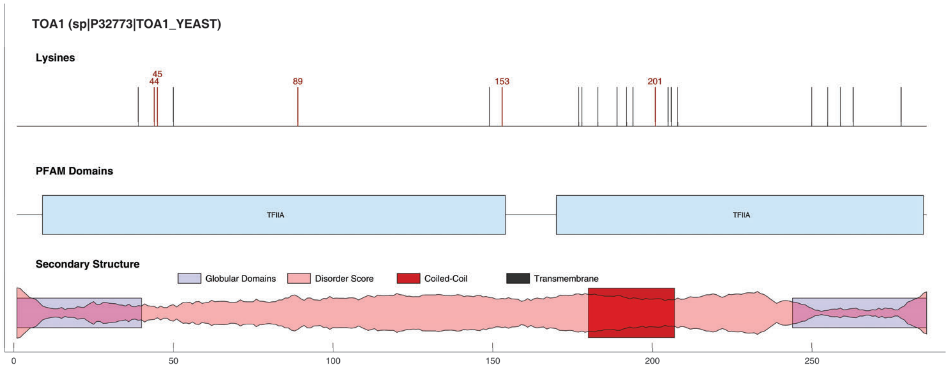
TOA1 (sp|P32773|TOA1_YEAST)
Lysines
45
44
89
153
201
PFAM Domains
TFIIA
TFIIA
Secondary Structure
Globular Domains
Disorder Score
Coiled-Coil
Transmembrane
0
50
100
150
200
250

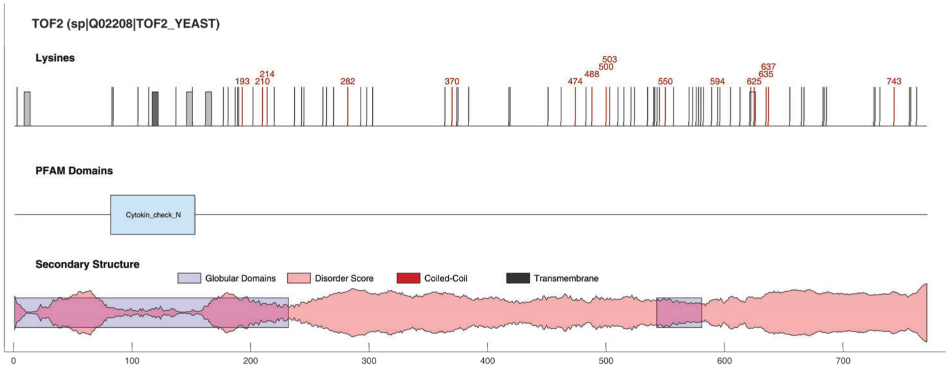
TOF2 (sp|Q02208|TOF2_YEAST)
Lysines
193 210 214 282 370 474 488 500 503 550 594 625 635 637 743
PFAM Domains
Cytokin_check_N
Secondary Structure
Globular Domains
Disorder Score
Coiled-Coil
Transmembrane
0 100 200 300 400 500 600 700

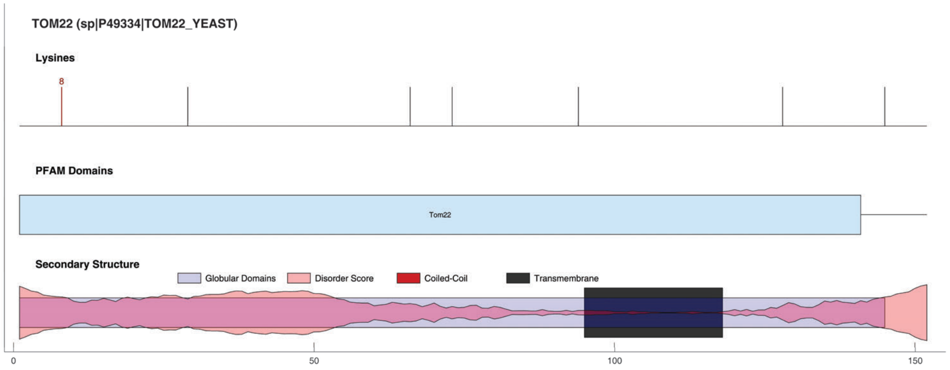
TOM22 (sp|P49334|TOM22_YEAST)
Lysines
8
PFAM Domains
Tom22
Secondary Structure
Globular Domains
Disorder Score
Coiled-Coil
Transmembrane
0 50 100 150

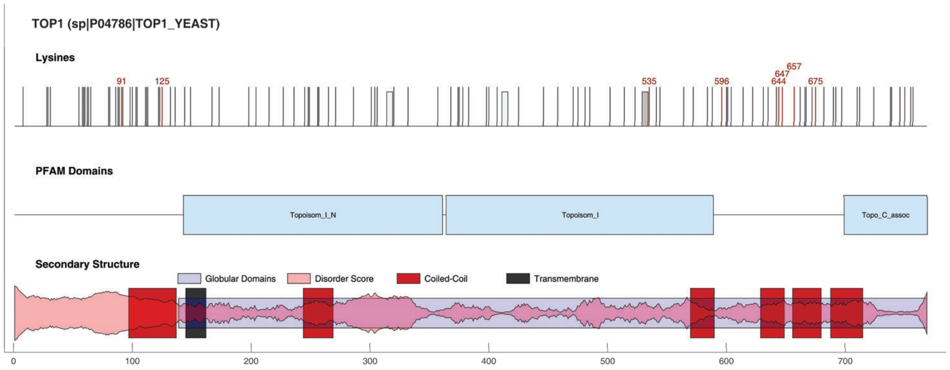
TOP1 (sp|P04786|TOP1_YEAST)
Lysines
91 125 535 596 644 647 657 675
PFAM Domains
Topoisom_I_N
Topoisom_I
Topo_C_assoc
Secondary Structure
Globular Domains
Disorder Score
Coiled-Coil
Transmembrane
0 100 200 300 400 500 600 700

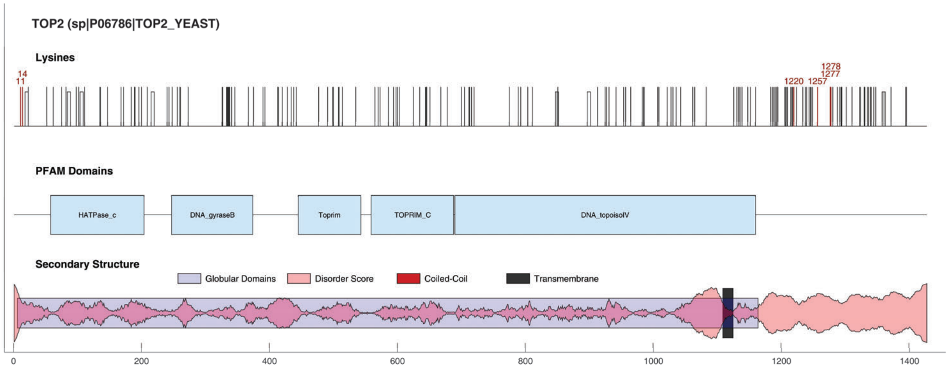
TOP2 (sp|P06786|TOP2_YEAST)
Lysines
11 14 1220 1257 1277 1278
PFAM Domains
HATPase_c
DNA_gyraseB
Toprim
TOPRIM_C
DNA_topoisoIV
Secondary Structure
Globular Domains
Disorder Score
Coiled-Coil
Transmembrane
0 200 400 600 800 1000 1200 1400

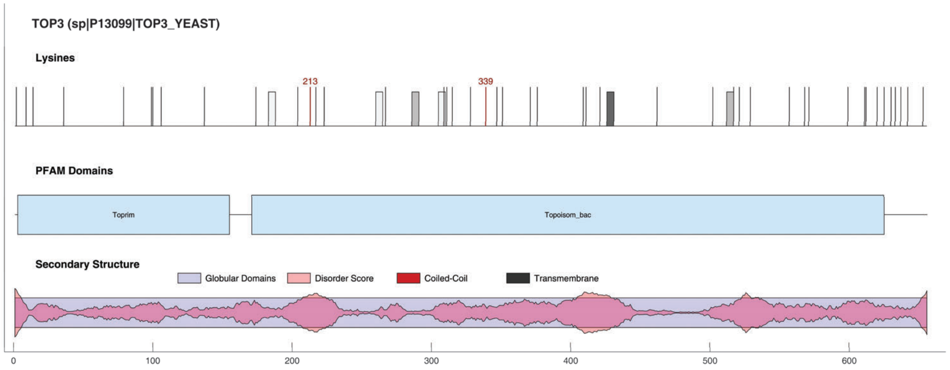
TOP3 (sp|P13099|TOP3_YEAST)
Lysines
213
339
PFAM Domains
Toprim
Topoisom_bac
Secondary Structure
Globular Domains
Disorder Score
Coiled-Coil
Transmembrane
0
100
200
300
400
500
600

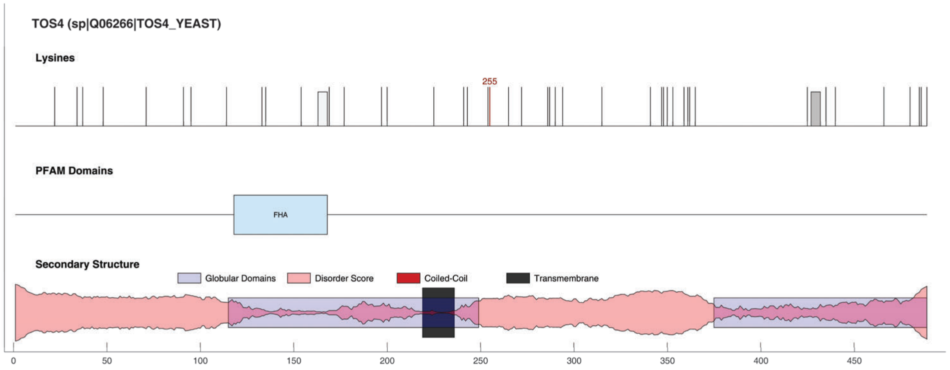
TOS4 (sp|Q06266|TOS4_YEAST)
Lysines
255
PFAM Domains
FHA
Secondary Structure
Globular Domains
Disorder Score
Coiled-Coil
Transmembrane
0
50
100
150
200
250
300
350
400
450

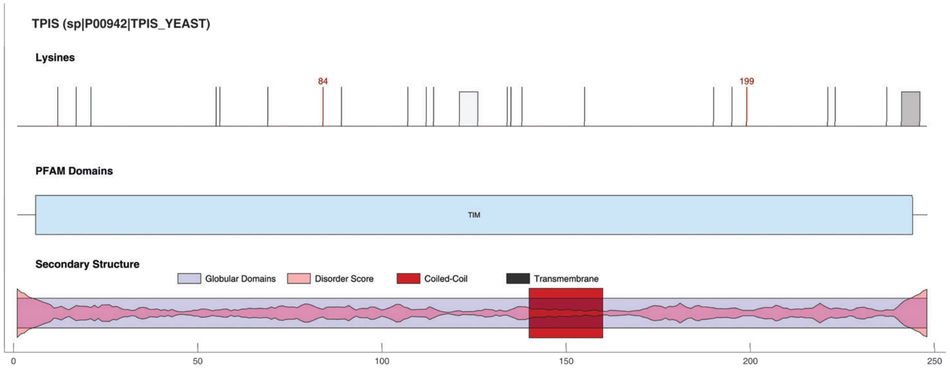
TPIS (sp|P00942|TPIS_YEAST)
Lysines
84
199
PFAM Domains
TIM
Secondary Structure
Globular Domains
Disorder Score
Coiled-Coil
Transmembrane
0
50
100
150
200
250

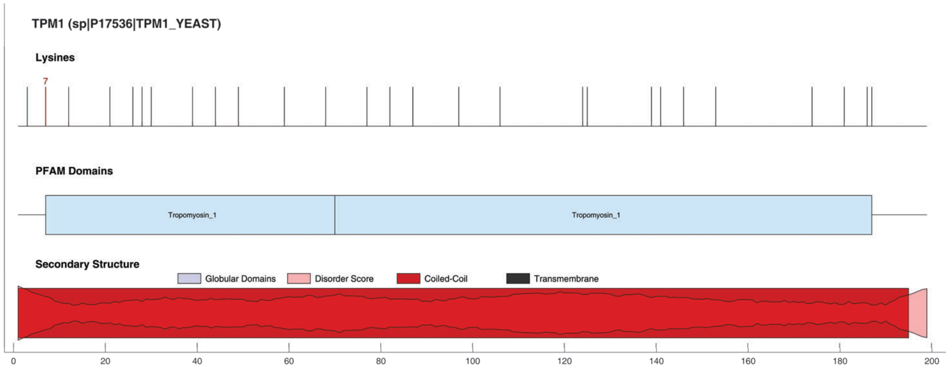
TPM1 (sp|P17536|TPM1_YEAST)
Lysines
7
PFAM Domains
Tropomyosin_1
Tropomyosin_1
Secondary Structure
Globular Domains
Disorder Score
Coiled-Coil
Transmembrane
0
20
40
60
80
100
120
140
160
180
200

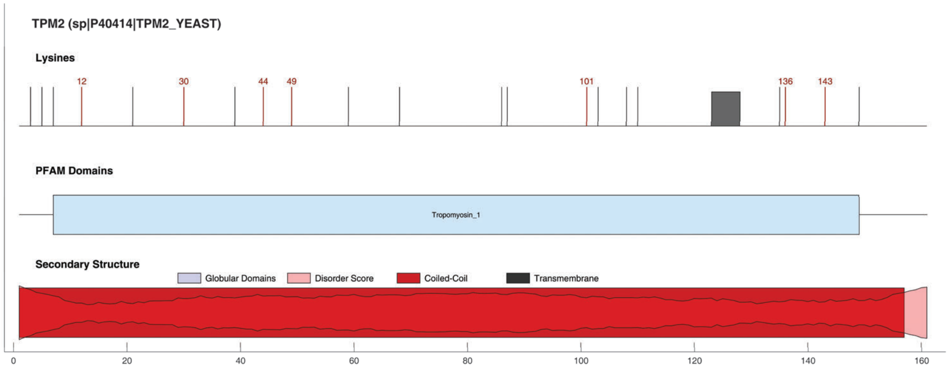
TPM2 (sp|P40414|TPM2_YEAST)
Lysines
12
30
44
49
101
136
143
PFAM Domains
Tropomyosin_1
Secondary Structure
Globular Domains
Disorder Score
Coiled-Coil
Transmembrane
0 20 40 60 80 100 120 140 160

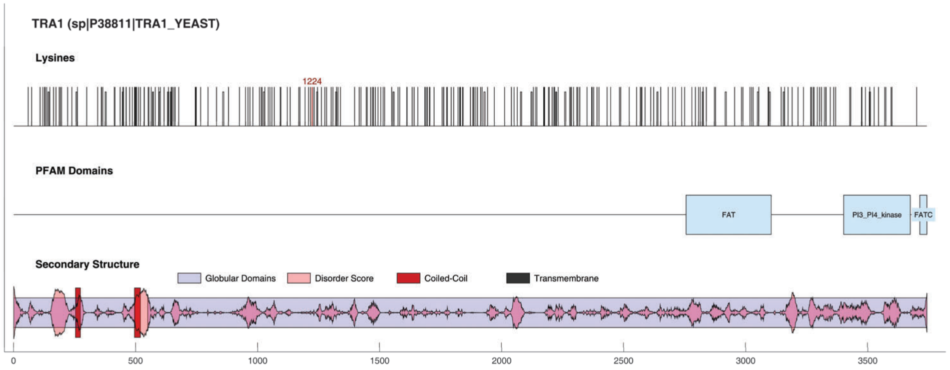
TRA1 (sp|P38811|TRA1_YEAST)
Lysines
1224
PFAM Domains
FAT
PI3_PI4_kinase
FATC
Secondary Structure
Globular Domains
Disorder Score
Coiled-Coil
Transmembrane
0 500 1000 1500 2000 2500 3000 3500

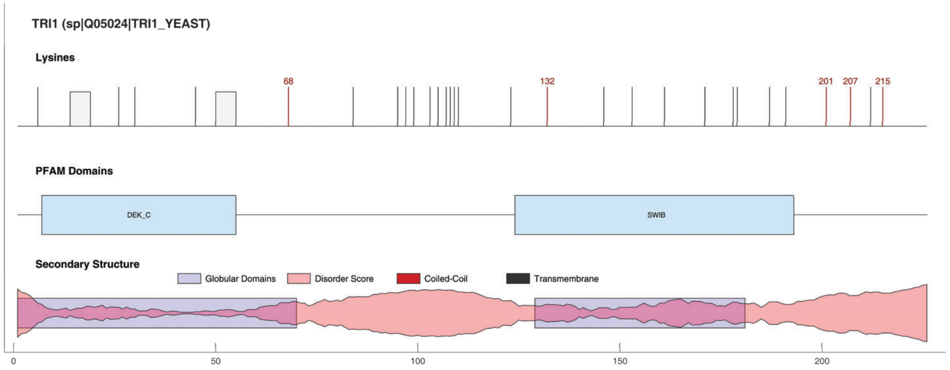
TRI1 (sp|Q05024|TRI1_YEAST)
Lysines
68
132
201
207
215
PFAM Domains
DEK_C
SWIB
Secondary Structure
Globular Domains
Disorder Score
Coiled-Coil
Transmembrane
0 50 100 150 200

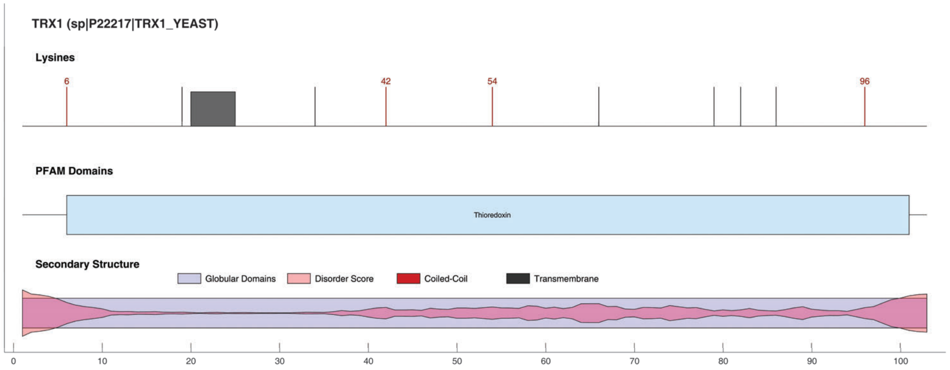
TRX1 (sp|P22217|TRX1_YEAST)
Lysines
6
42
54
96
PFAM Domains
Thioredoxin
Secondary Structure
Globular Domains
Disorder Score
Coiled-Coil
Transmembrane
0 10 20 30 40 50 60 70 80 90 100

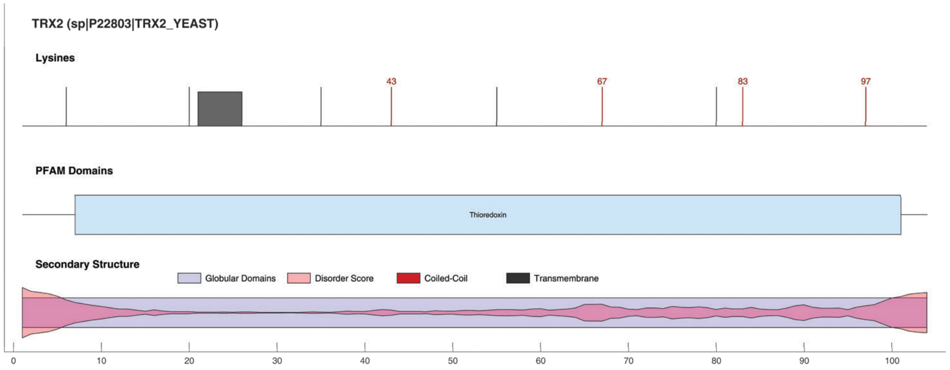
TRX2 (sp|P22803|TRX2_YEAST)
Lysines
43
67
83
97
PFAM Domains
Thioredoxin
Secondary Structure
Globular Domains
Disorder Score
Coiled-Coil
Transmembrane
0
10
20
30
40
50
60
70
80
90
100

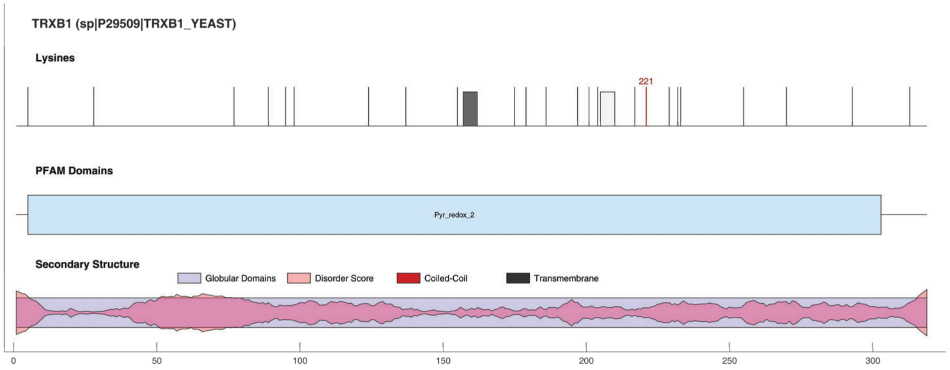
TRXB1 (sp|P29509|TRXB1_YEAST)
Lysines
221
PFAM Domains
Pyr_redox_2
Secondary Structure
Globular Domains
Disorder Score
Coiled-Coil
Transmembrane
0
50
100
150
200
250
300

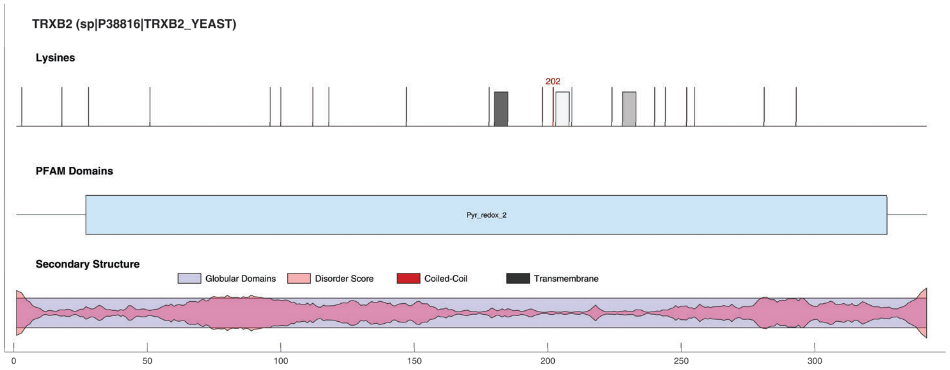
TRXB2 (sp|P38816|TRXB2_YEAST)
Lysines
202
PFAM Domains
Pyr_redox_2
Secondary Structure
Globular Domains
Disorder Score
Coiled-Coil
Transmembrane
0
50
100
150
200
250
300

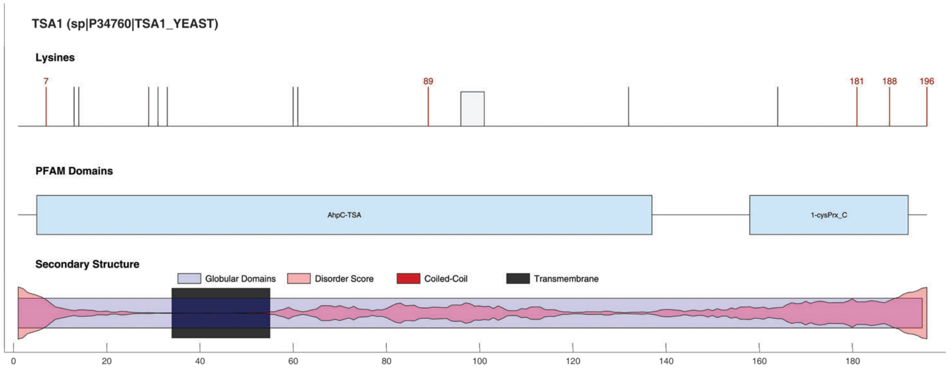
TSA1 (sp|P34760|TSA1_YEAST)
Lysines
7
89
181
188
196
PFAM Domains
AhpC-TSA
1-cysPrx_C
Secondary Structure
Globular Domains
Disorder Score
Coiled-Coil
Transmembrane
0
20
40
60
80
100
120
140
160
180

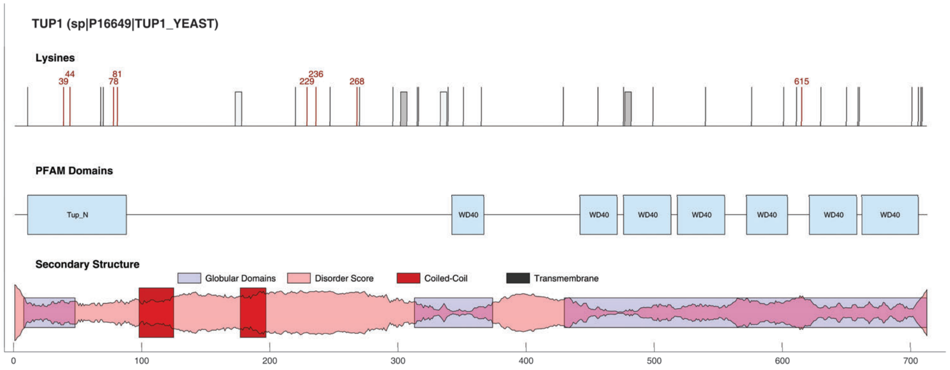
TUP1 (sp|P16649|TUP1_YEAST)
Lysines
39 44 78 81 229 236 268 615
PFAM Domains
Tup_N
WD40
Secondary Structure
Globular Domains
Disorder Score
Coiled-Coil
Transmembrane
0 100 200 300 400 500 600 700

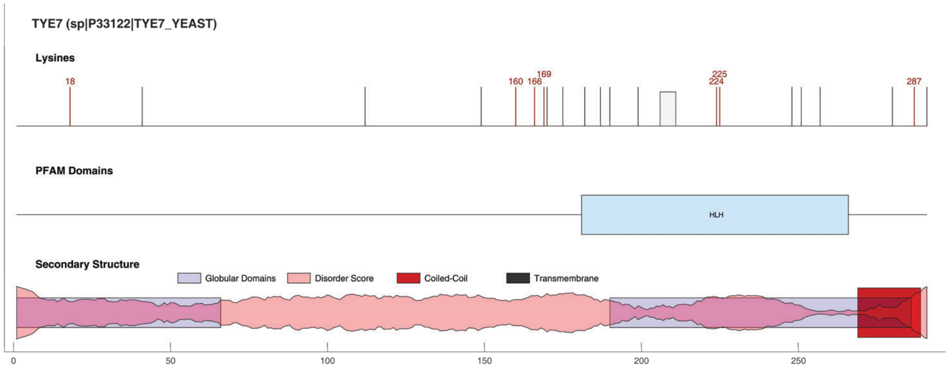
TYE7 (sp|P33122|TYE7_YEAST)
Lysines
18 160 166 169 224 225 287
PFAM Domains
HLH
Secondary Structure
Globular Domains
Disorder Score
Coiled-Coil
Transmembrane
0 50 100 150 200 250

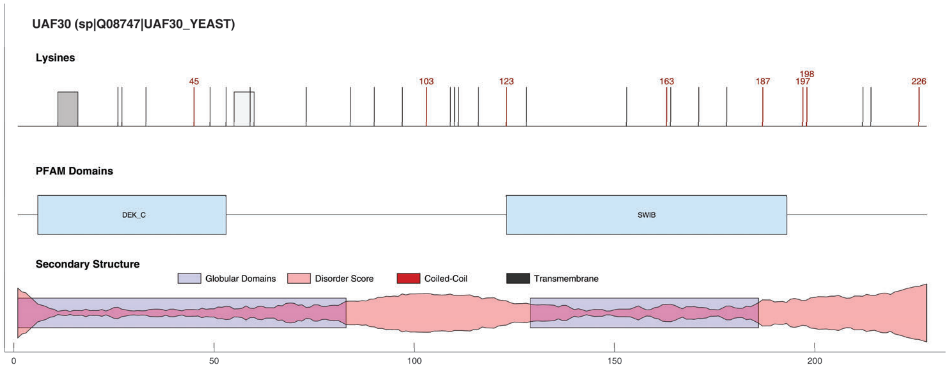
UAF30 (sp|Q08747|UAF30_YEAST)
Lysines
45 103 123 163 187 197 198 226
PFAM Domains
DEK_C
SWIB
Secondary Structure
Globular Domains
Disorder Score
Coiled-Coil
Transmembrane
0 50 100 150 200

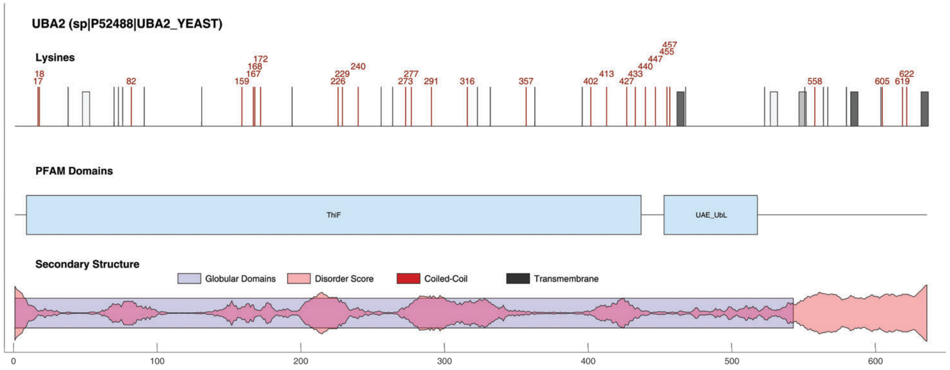
UBA2 (sp|P52488|UBA2_YEAST)
Lysines
17 18 82 159 167 168 172 226 229 240 273 277 291 316 357 402 413 427 433 440 447 455 457 558 605 619 622
PFAM Domains
ThiF
UAE_UbL
Secondary Structure
Globular Domains
Disorder Score
Coiled-Coil
Transmembrane
0 100 200 300 400 500 600

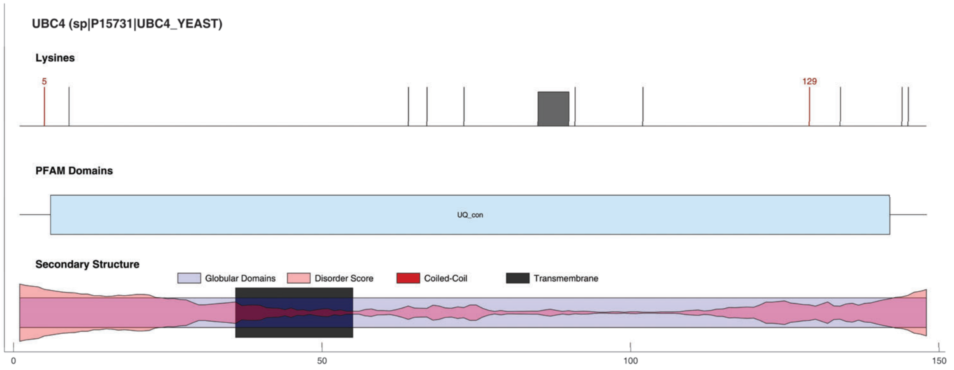
UBC4 (sp|P15731|UBC4_YEAST)
Lysines
5
129
PFAM Domains
UQ_con
Secondary Structure
Globular Domains
Disorder Score
Coiled-Coil
Transmembrane
0
50
100
150

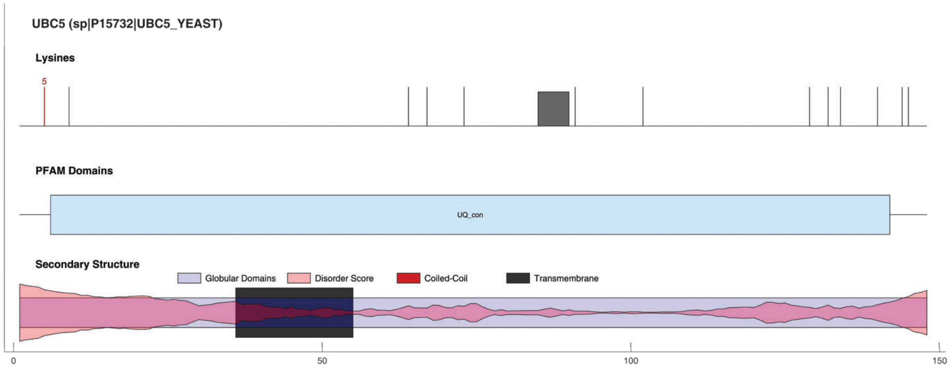
UBC5 (sp|P15732|UBC5_YEAST)
Lysines
5
PFAM Domains
UQ_con
Secondary Structure
Globular Domains
Disorder Score
Coiled-Coil
Transmembrane
0
50
100
150

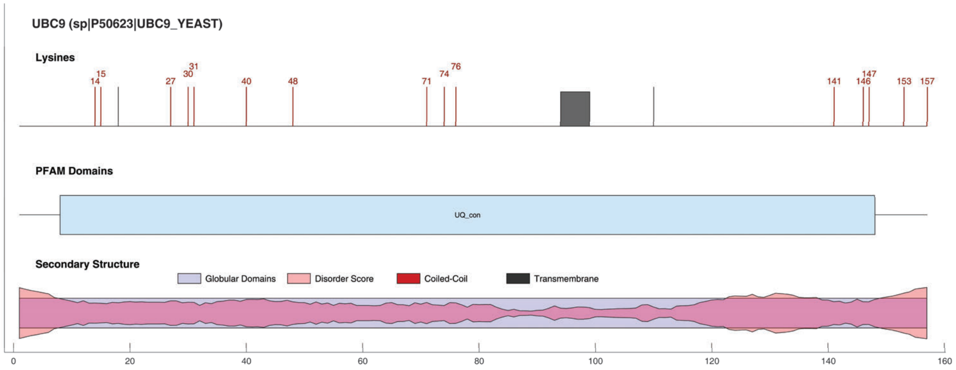
UBC9 (sp|P50623|UBC9_YEAST)
Lysines
14
15
27
30
31
40
48
71
74
76
141
146
147
153
157
PFAM Domains
UQ_con
Secondary Structure
Globular Domains
Disorder Score
Coiled-Coil
Transmembrane
0
20
40
60
80
100
120
140
160

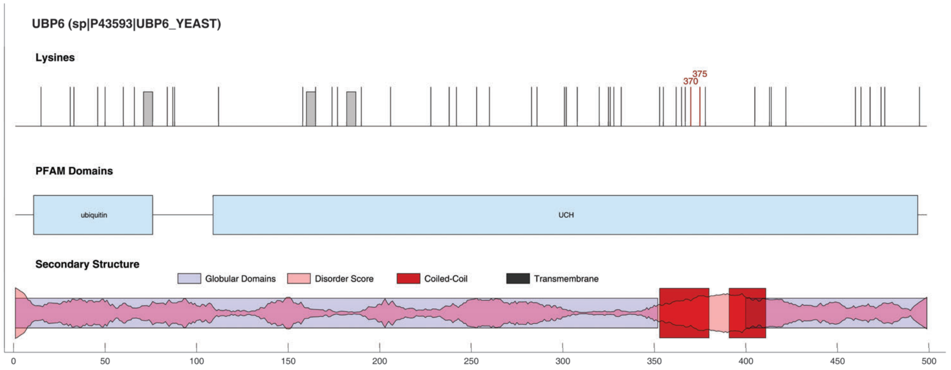
UBP6 (sp|P43593|UBP6_YEAST)
Lysines
370
375
PFAM Domains
ubiquitin
UCH
Secondary Structure
Globular Domains
Disorder Score
Coiled-Coil
Transmembrane
0
50
100
150
200
250
300
350
400
450
500

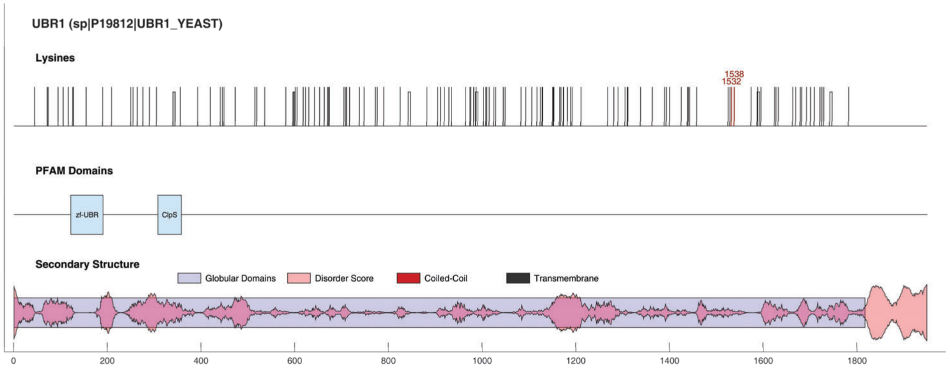
UBR1 (sp|P19812|UBR1_YEAST)
Lysines
1538
1532
PFAM Domains
zf-UBR
ClpS
Secondary Structure
Globular Domains
Disorder Score
Coiled-Coil
Transmembrane
0
200
400
600
800
1000
1200
1400
1600
1800

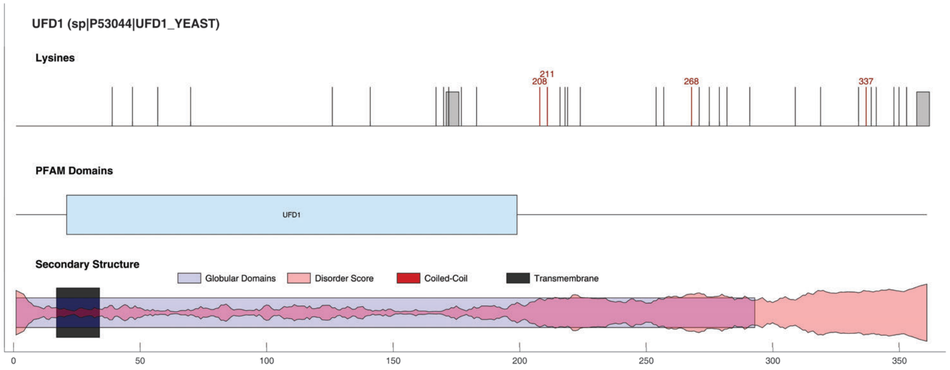
UFD1 (sp|P53044|UFD1_YEAST)
Lysines
211
208
268
337
PFAM Domains
UFD1
Secondary Structure
Globular Domains
Disorder Score
Coiled-Coil
Transmembrane
0
50
100
150
200
250
300
350

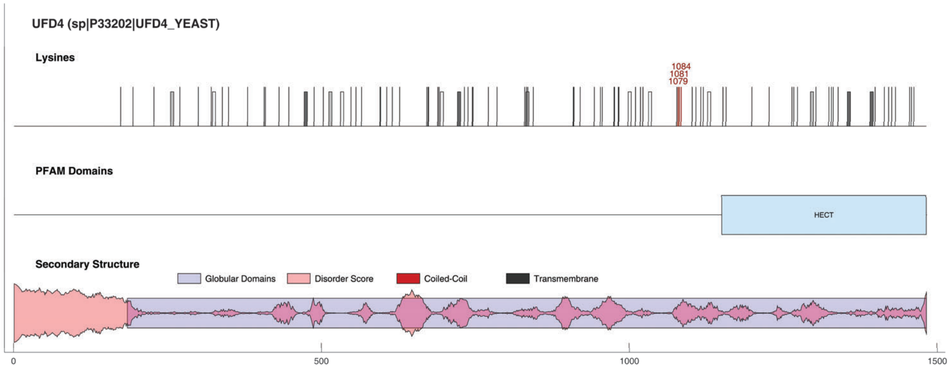
UFD4 (sp|P33202|UFD4_YEAST)
Lysines
1084
1081
1079
PFAM Domains
HECT
Secondary Structure
Globular Domains
Disorder Score
Coiled-Coil
Transmembrane
0
500
1000
1500

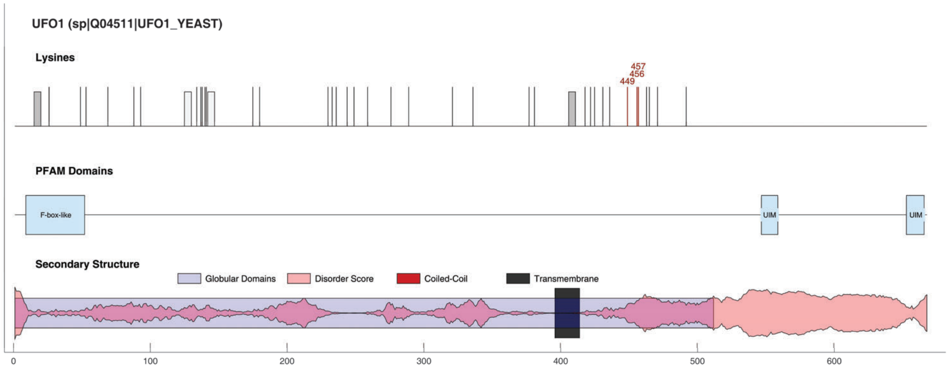
UFO1 (sp|Q04511|UFO1_YEAST)
Lysines
457
456
449
PFAM Domains
F-box-like
UIM
UIM
Secondary Structure
Globular Domains
Disorder Score
Coiled-Coil
Transmembrane
0
100
200
300
400
500
600

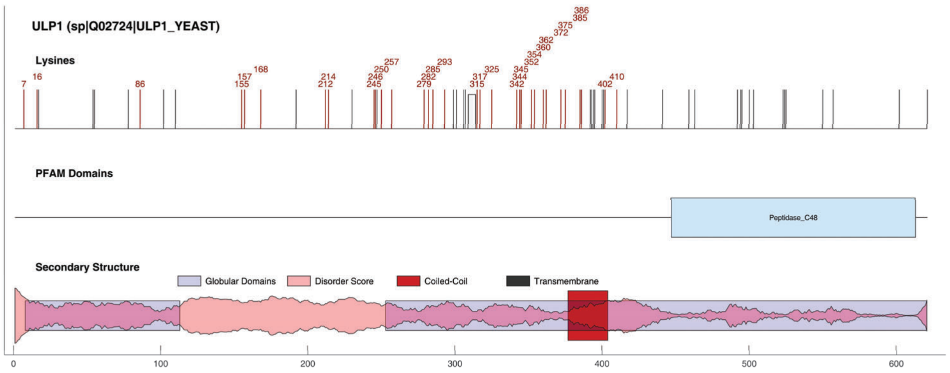
ULP1 (sp|Q02724|ULP1_YEAST)
Lysines
7 16 86 155 157 168 212 214 245 246 250 257 279 282 285 293 315 317 325 342 344 345 352 354 360 362 372 375 385 386 402 410
PFAM Domains
Peptidase_C48
Secondary Structure
Globular Domains
Disorder Score
Coiled-Coil
Transmembrane
0 100 200 300 400 500 600

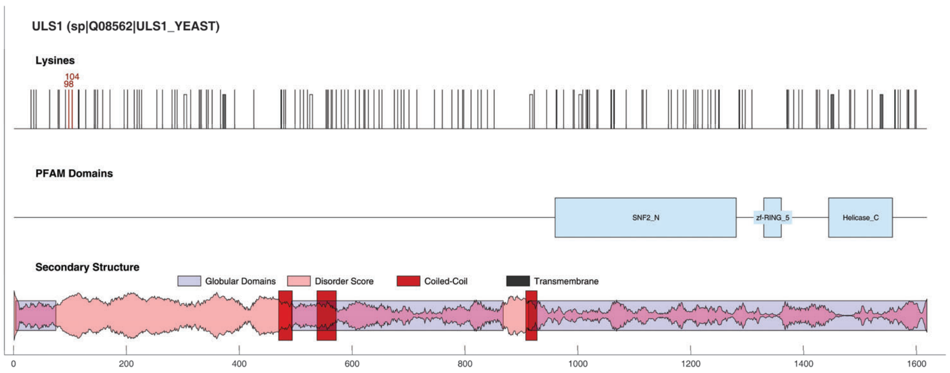
ULS1 (sp|Q08562|ULS1_YEAST)
Lysines
98 104
PFAM Domains
SNF2_N
zf-RING_5
Helicase_C
Secondary Structure
Globular Domains
Disorder Score
Coiled-Coil
Transmembrane
0 200 400 600 800 1000 1200 1400 1600

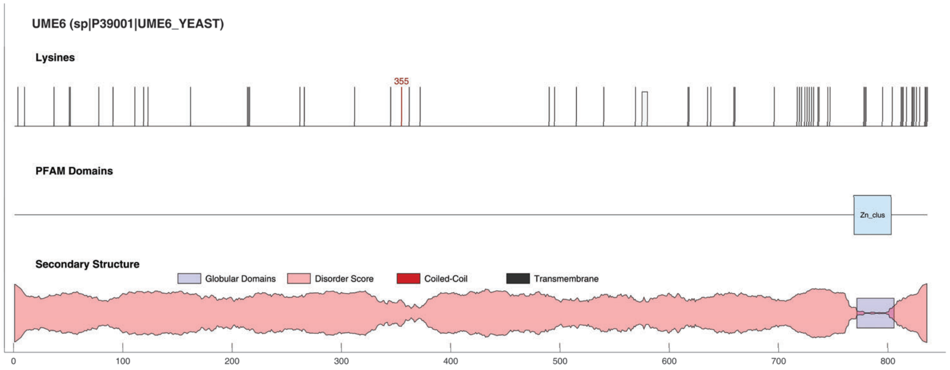
UME6 (sp|P39001|UME6_YEAST)
Lysines
355
PFAM Domains
Zn_clus
Secondary Structure
Globular Domains
Disorder Score
Coiled-Coil
Transmembrane
0 100 200 300 400 500 600 700 800

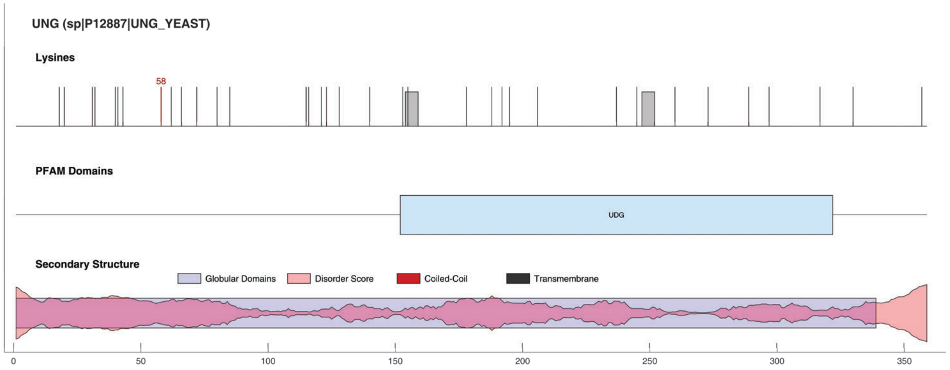
UNG (sp|P12887|UNG_YEAST)
Lysines
58
PFAM Domains
UDG
Secondary Structure
Globular Domains
Disorder Score
Coiled-Coil
Transmembrane
0 50 100 150 200 250 300 350

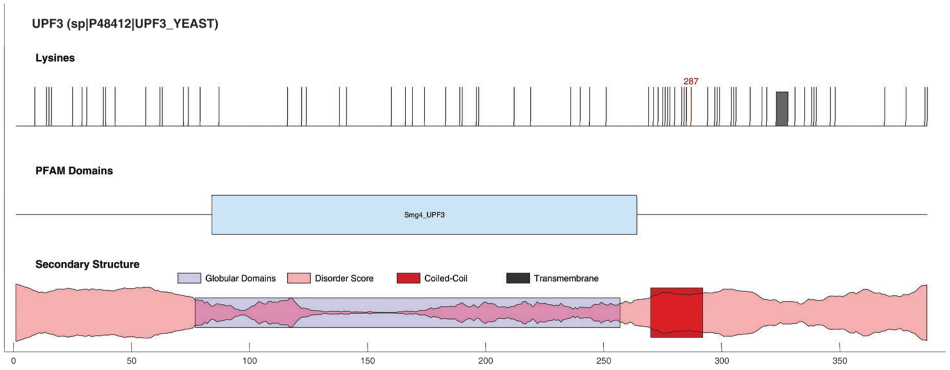
UPF3 (sp|P48412|UPF3_YEAST)
Lysines
287
PFAM Domains
Smg4_UPF3
Secondary Structure
Globular Domains
Disorder Score
Coiled-Coil
Transmembrane
0
50
100
150
200
250
300
350

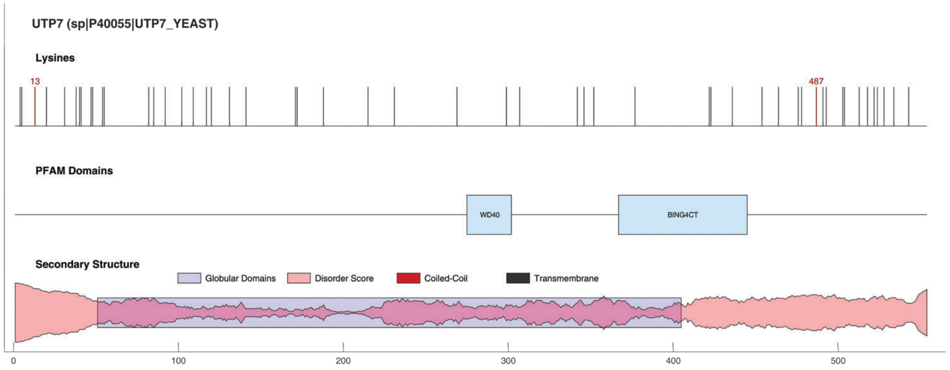
UTP7 (sp|P40055|UTP7_YEAST)
Lysines
13
487
PFAM Domains
WD40
BING4CT
Secondary Structure
Globular Domains
Disorder Score
Coiled-Coil
Transmembrane
0
100
200
300
400
500

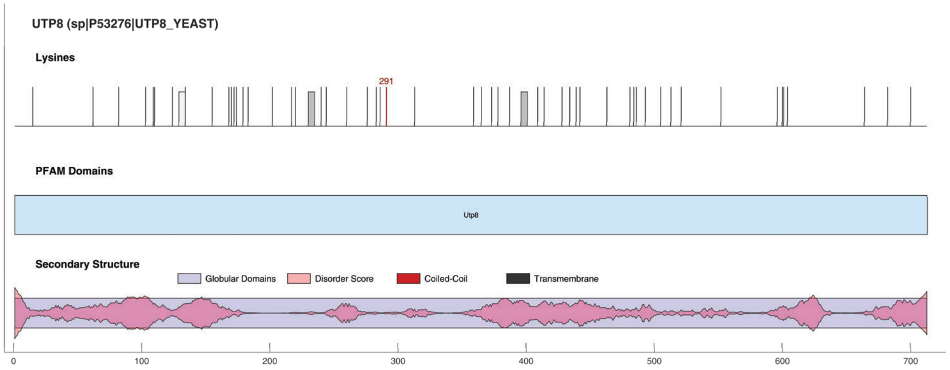
UTP8 (sp|P53276|UTP8_YEAST)
Lysines
291
PFAM Domains
Utp8
Secondary Structure
Globular Domains
Disorder Score
Coiled-Coil
Transmembrane
0
100
200
300
400
500
600
700

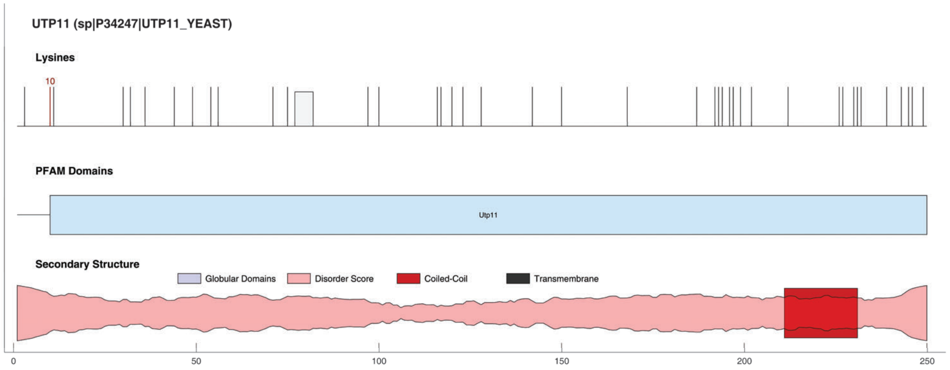
UTP11 (sp|P34247|UTP11_YEAST)
Lysines
10
PFAM Domains
Utp11
Secondary Structure
Globular Domains
Disorder Score
Coiled-Coil
Transmembrane
0
50
100
150
200
250

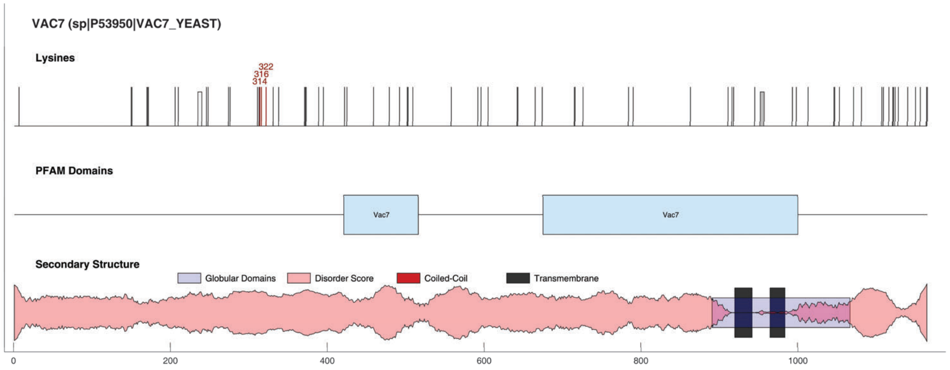

VAC7 (sp|P53950|VAC7_YEAST)
Lysines
322
316
314
PFAM Domains
Vac7
Vac7
Secondary Structure
Globular Domains
Disorder Score
Coiled-Coil
Transmembrane
0
200
400
600
800
1000

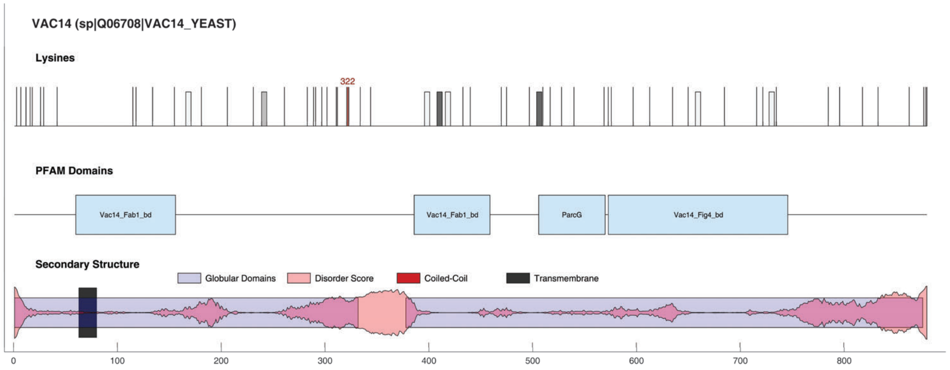

VAC14 (sp|Q06708|VAC14_YEAST)
Lysines
322
PFAM Domains
Vac14_Fab1_bd
Vac14_Fab1_bd
ParcG
Vac14_Fig4_bd
Secondary Structure
Globular Domains
Disorder Score
Coiled-Coil
Transmembrane
0
100
200
300
400
500
600
700
800

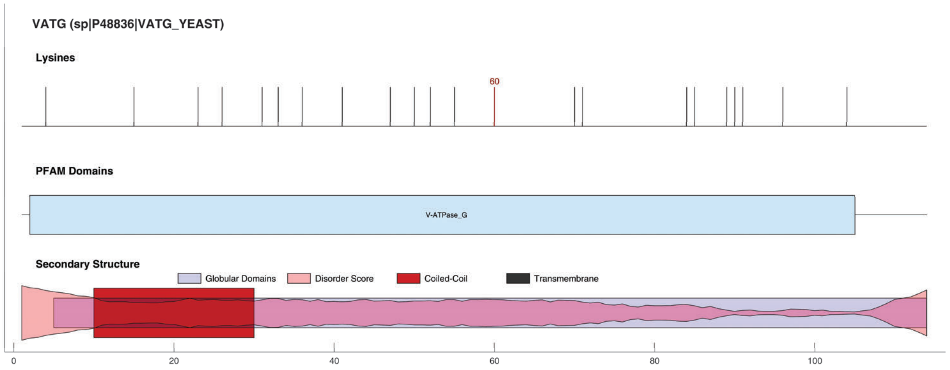

VATG (sp|P48836|VATG_YEAST)
Lysines
60
PFAM Domains
V-ATPase_G
Secondary Structure
Globular Domains
Disorder Score
Coiled-Coil
Transmembrane
0
20
40
60
80
100

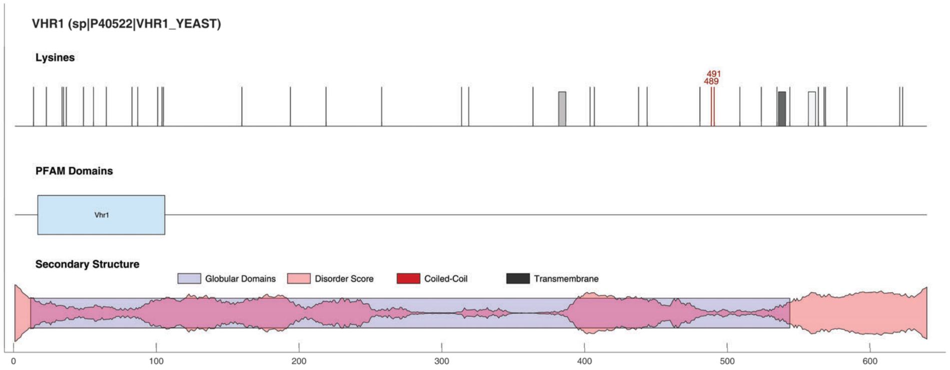

VHR1 (sp|P40522|VHR1_YEAST)
Lysines
491
489
PFAM Domains
Vhr1
Secondary Structure
Globular Domains
Disorder Score
Coiled-Coil
Transmembrane
0
100
200
300
400
500
600

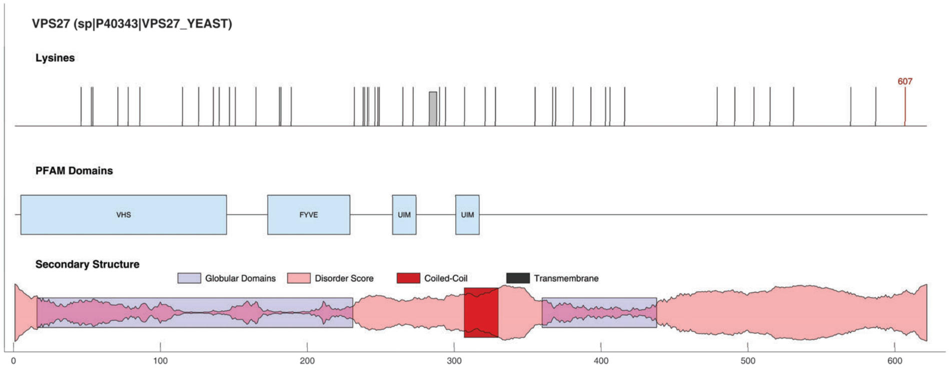
VPS27 (sp|P40343|VPS27_YEAST)
Lysines
607
PFAM Domains
VHS
FYVE
UIM
UIM
Secondary Structure
Globular Domains
Disorder Score
Coiled-Coil
Transmembrane
0
100
200
300
400
500
600

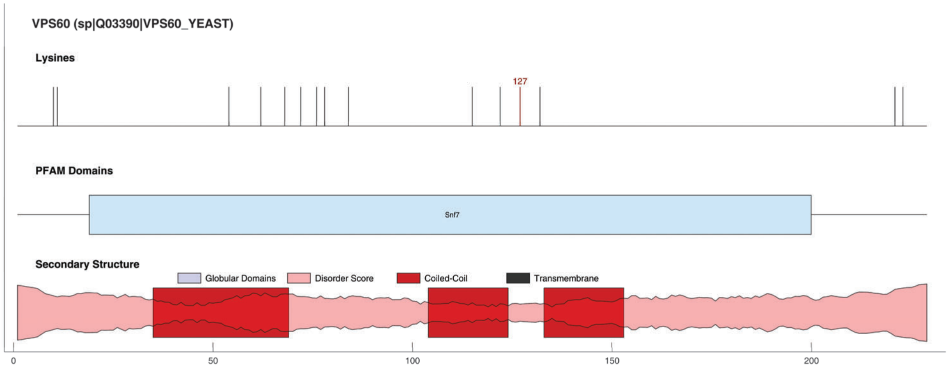
VPS60 (sp|Q03390|VPS60_YEAST)
Lysines
127
PFAM Domains
Snf7
Secondary Structure
Globular Domains
Disorder Score
Coiled-Coil
Transmembrane
0
50
100
150
200

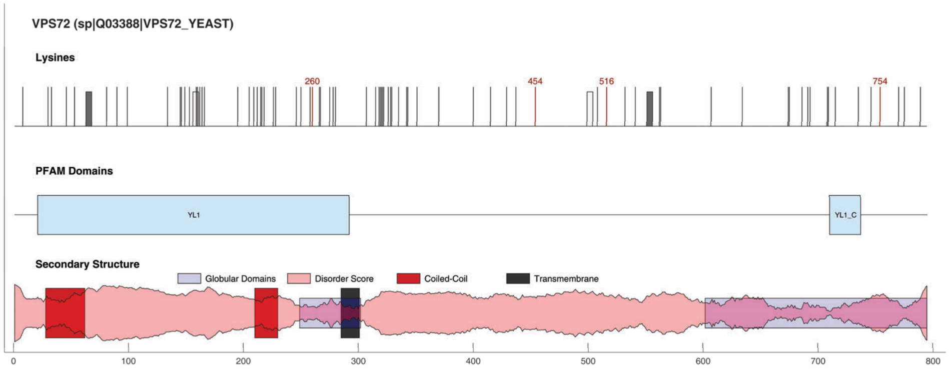
VPS72 (sp|Q03388|VPS72_YEAST)
Lysines
260
454
516
754
PFAM Domains
YL1
YL1_C
Secondary Structure
Globular Domains
Disorder Score
Coiled-Coil
Transmembrane
0
100
200
300
400
500
600
700
800

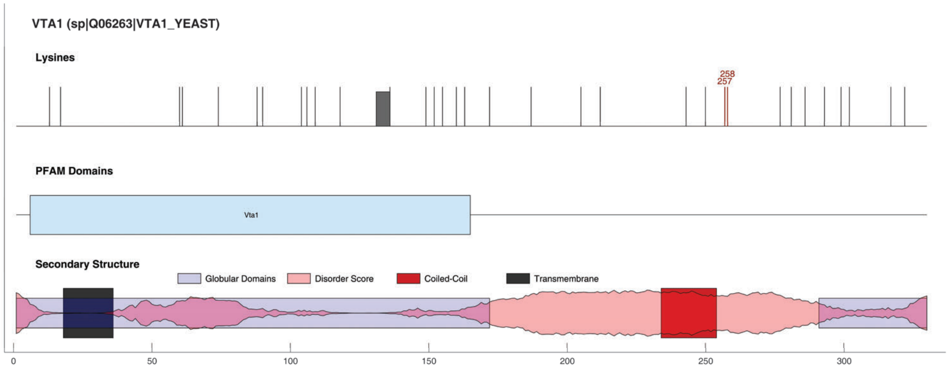
VTA1 (sp|Q06263|VTA1_YEAST)
Lysines
258
257
PFAM Domains
Vta1
Secondary Structure
Globular Domains
Disorder Score
Coiled-Coil
Transmembrane
0
50
100
150
200
250
300

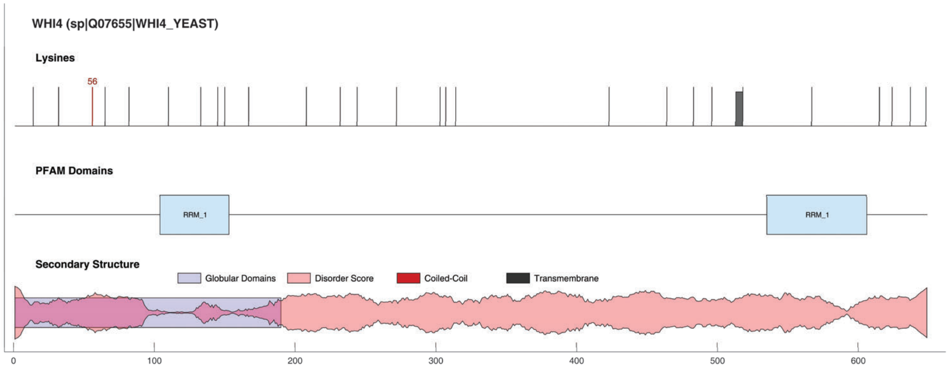
WHI4 (sp|Q07655|WHI4_YEAST)
Lysines
56
PFAM Domains
RRM_1
RRM_1
Secondary Structure
Globular Domains
Disorder Score
Coiled-Coil
Transmembrane
0
100
200
300
400
500
600

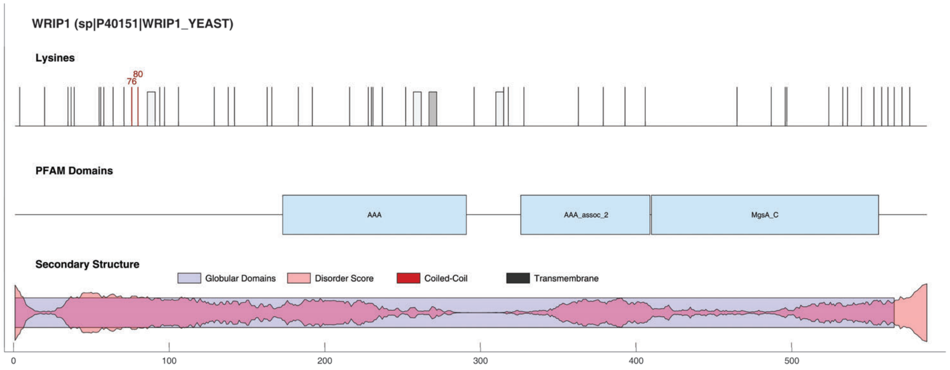
WRIP1 (sp|P40151|WRIP1_YEAST)
Lysines
80
76
PFAM Domains
AAA
AAA_assoc_2
MgsA_C
Secondary Structure
Globular Domains
Disorder Score
Coiled-Coil
Transmembrane
0
100
200
300
400
500

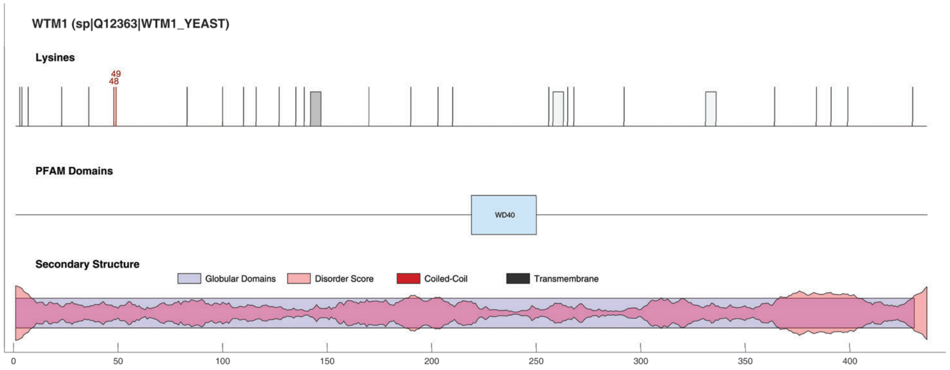
WTM1 (sp|Q12363|WTM1_YEAST)
Lysines
49
48
PFAM Domains
WD40
Secondary Structure
Globular Domains
Disorder Score
Coiled-Coil
Transmembrane
0
50
100
150
200
250
300
350
400

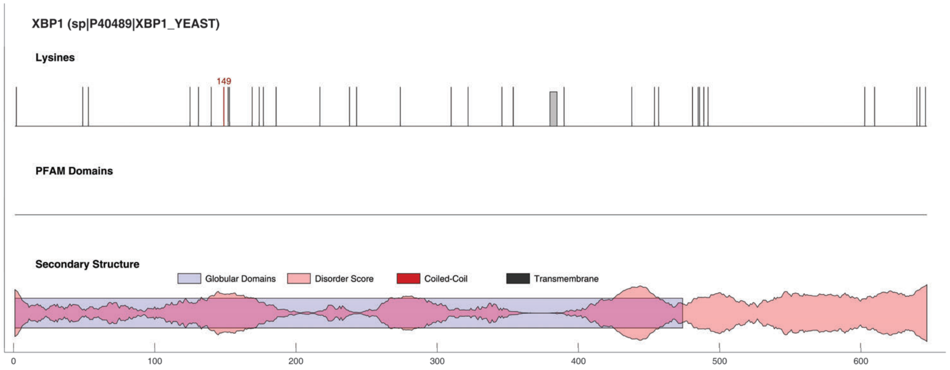
XBP1 (sp|P40489|XBP1_YEAST)
Lysines
149
PFAM Domains
Secondary Structure
Globular Domains
Disorder Score
Coiled-Coil
Transmembrane
0
100
200
300
400
500
600

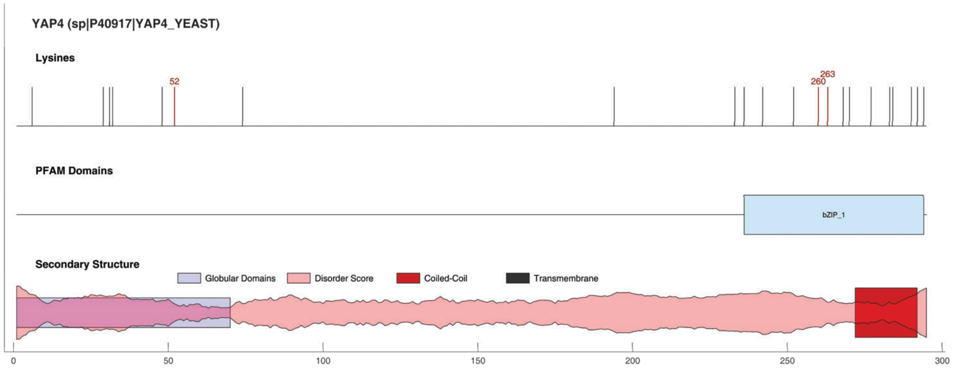
YAP4 (sp|P40917|YAP4_YEAST)
Lysines
52
260
263
PFAM Domains
bZIP_1
Secondary Structure
Globular Domains
Disorder Score
Coiled-Coil
Transmembrane
0
50
100
150
200
250
300

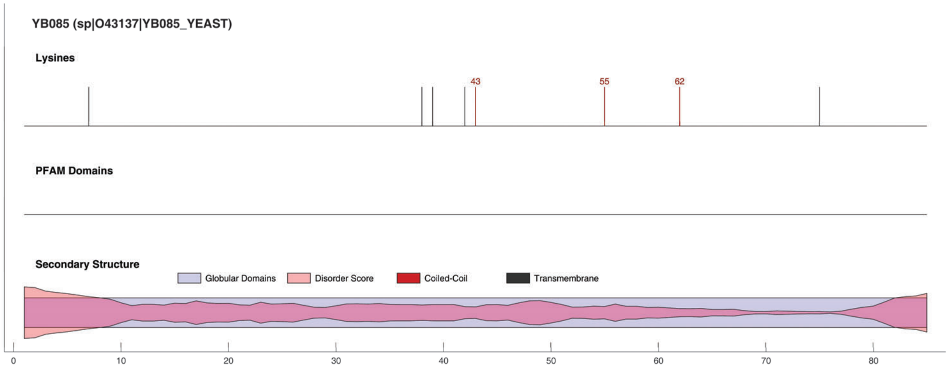
YB085 (sp|O43137|YB085_YEAST)
Lysines
43
55
62
PFAM Domains
Secondary Structure
Globular Domains
Disorder Score
Coiled-Coil
Transmembrane
0
10
20
30
40
50
60
70
80

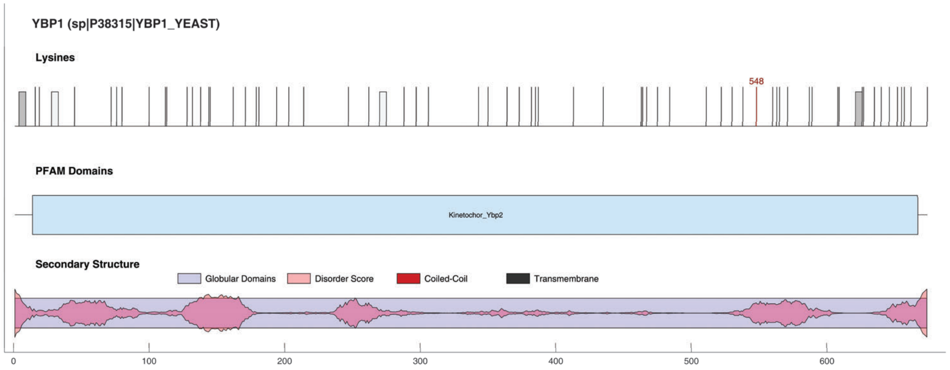
YBP1 (sp|P38315|YBP1_YEAST)
Lysines
548
PFAM Domains
Kinetochor_Ybp2
Secondary Structure
Globular Domains
Disorder Score
Coiled-Coil
Transmembrane
0
100
200
300
400
500
600

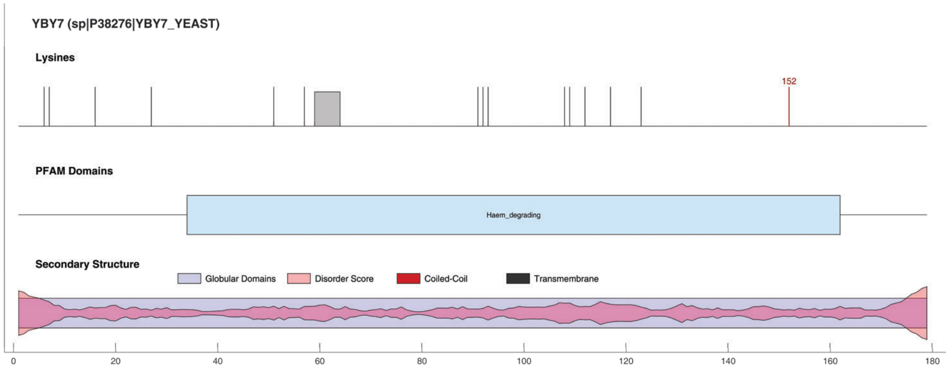
YBY7 (sp|P38276|YBY7_YEAST)
Lysines
152
PFAM Domains
Haem_degrading
Secondary Structure
Globular Domains
Disorder Score
Coiled-Coil
Transmembrane
0
20
40
60
80
100
120
140
160
180

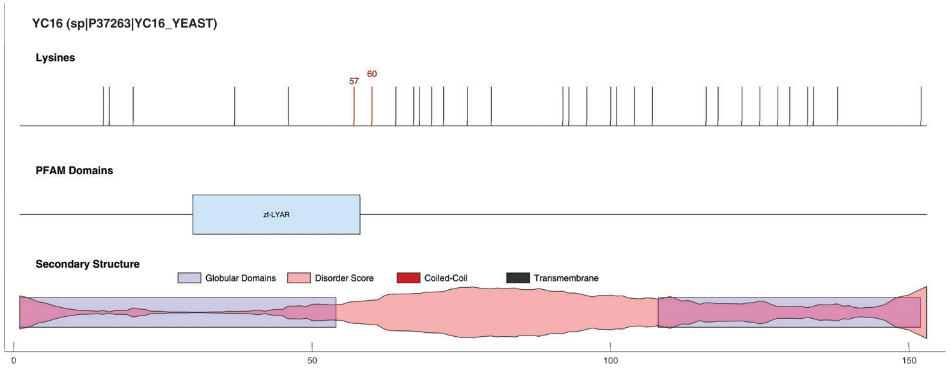
YC16 (sp|P37263|YC16_YEAST)
Lysines
57
60
PFAM Domains
zf-LYAR
Secondary Structure
Globular Domains
Disorder Score
Coiled-Coil
Transmembrane
0
50
100
150

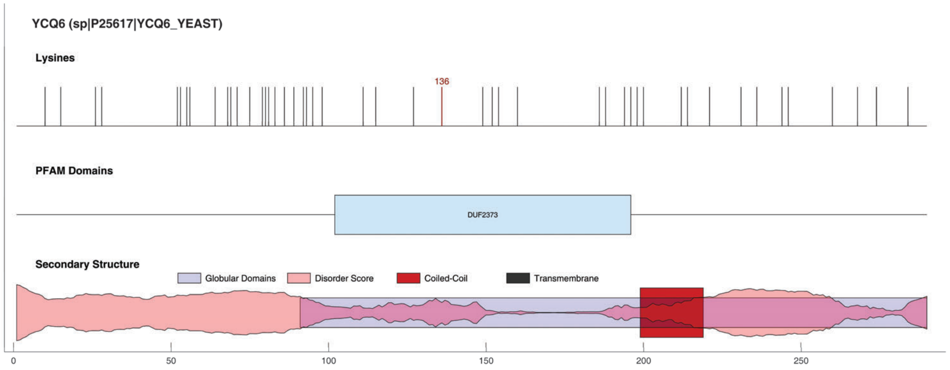
YCQ6 (sp|P25617|YCQ6_YEAST)
Lysines
136
PFAM Domains
DUF2373
Secondary Structure
Globular Domains
Disorder Score
Coiled-Coil
Transmembrane
0
50
100
150
200
250

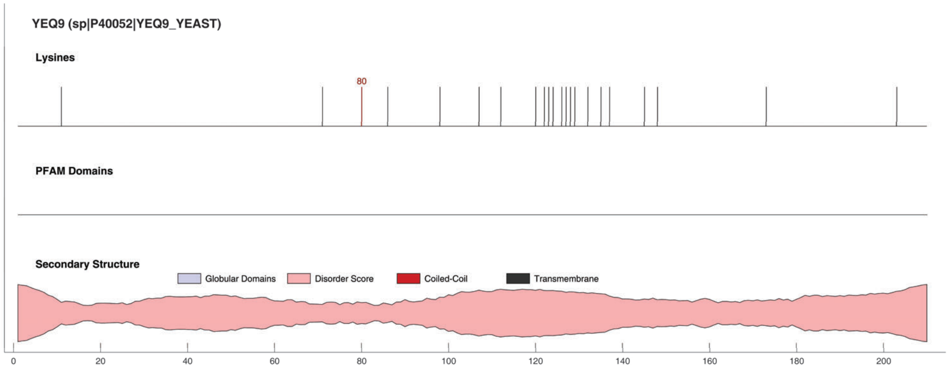
YEQ9 (sp|P40052|YEQ9_YEAST)
Lysines
80
PFAM Domains
Secondary Structure
Globular Domains
Disorder Score
Coiled-Coil
Transmembrane
0
20
40
60
80
100
120
140
160
180
200

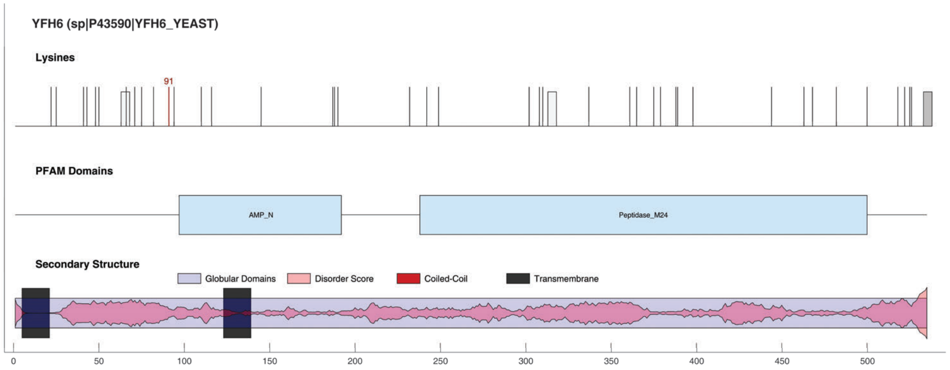
YFH6 (sp|P43590|YFH6_YEAST)
Lysines
91
PFAM Domains
AMP_N
Peptidase_M24
Secondary Structure
Globular Domains
Disorder Score
Coiled-Coil
Transmembrane
0
50
100
150
200
250
300
350
400
450
500

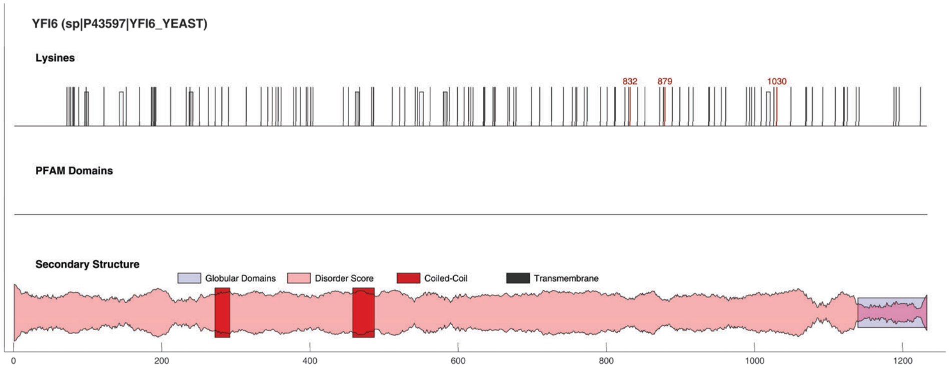

YFI6 (sp|P43597|YFI6_YEAST)
Lysines
832
879
1030
PFAM Domains
Secondary Structure
Globular Domains
Disorder Score
Coiled-Coil
Transmembrane
0
200
400
600
800
1000
1200

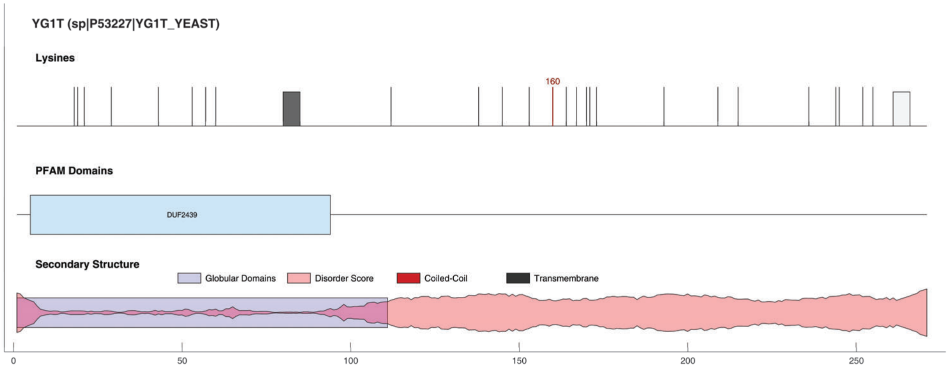

YG1T (sp|P53227|YG1T_YEAST)
Lysines
160
PFAM Domains
DUF2439
Secondary Structure
Globular Domains
Disorder Score
Coiled-Coil
Transmembrane
0
50
100
150
200
250

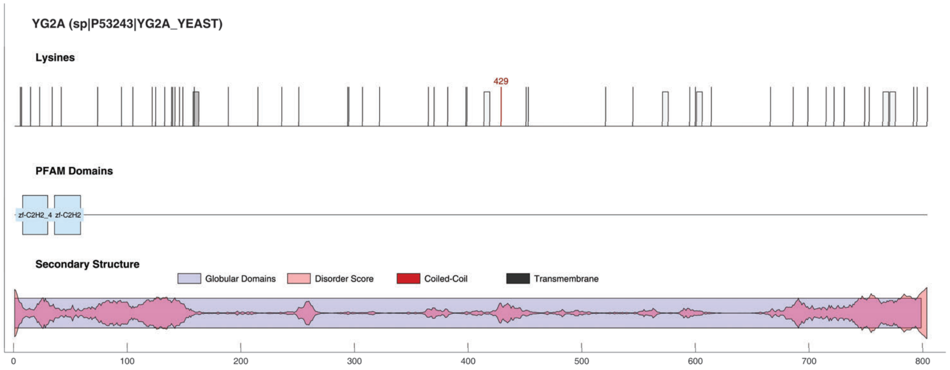

YG2A (sp|P53243|YG2A_YEAST)
Lysines
429
PFAM Domains
zf-C2H2_4
zf-C2H2
Secondary Structure
Globular Domains
Disorder Score
Coiled-Coil
Transmembrane
0
100
200
300
400
500
600
700
800

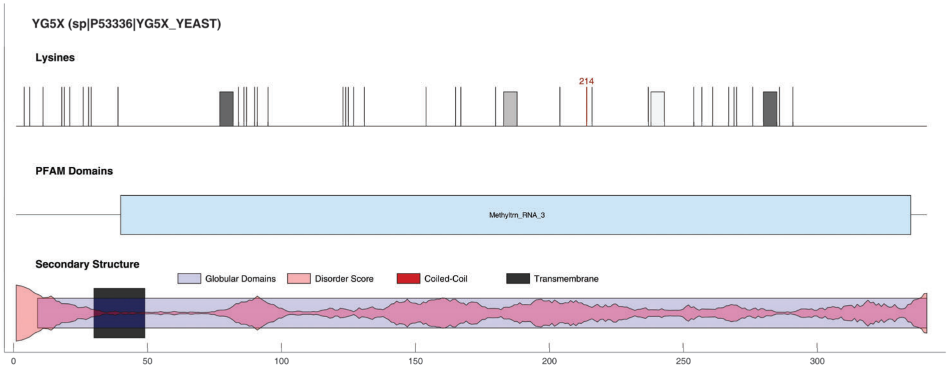

YG5X (sp|P53336|YG5X_YEAST)
Lysines
214
PFAM Domains
Methyltrn_RNA_3
Secondary Structure
Globular Domains
Disorder Score
Coiled-Coil
Transmembrane
0
50
100
150
200
250
300

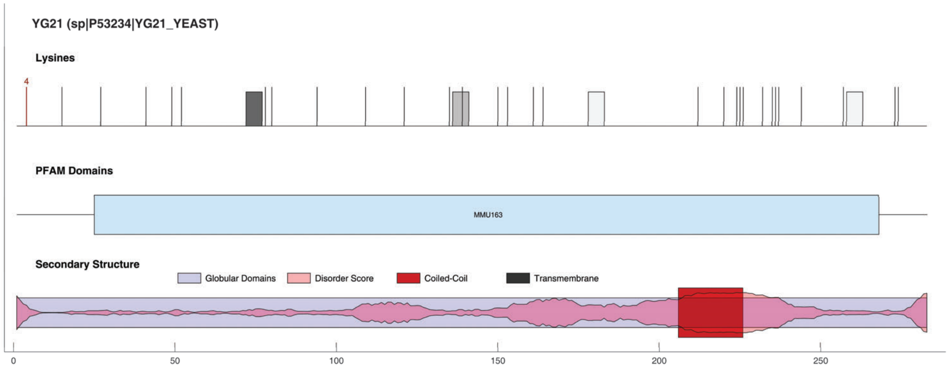
YG21 (sp|P53234|YG21_YEAST)
Lysines
4
PFAM Domains
MMU163
Secondary Structure
Globular Domains
Disorder Score
Coiled-Coil
Transmembrane
0
50
100
150
200
250

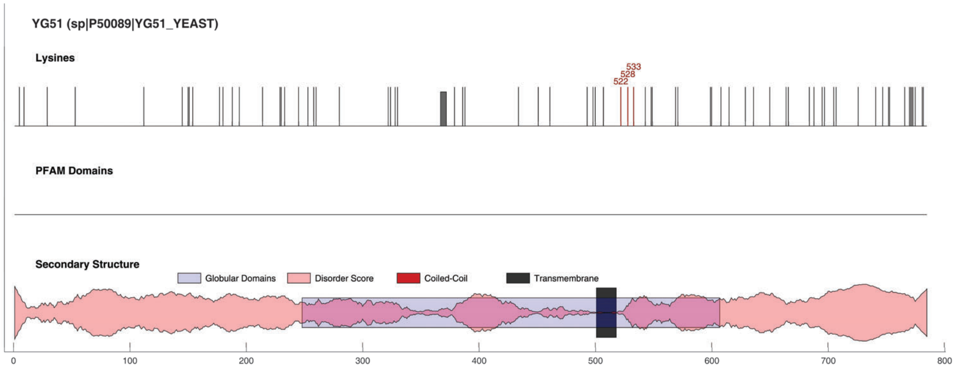
YG51 (sp|P50089|YG51_YEAST)
Lysines
533
528
522
PFAM Domains
Secondary Structure
Globular Domains
Disorder Score
Coiled-Coil
Transmembrane
0
100
200
300
400
500
600
700
800

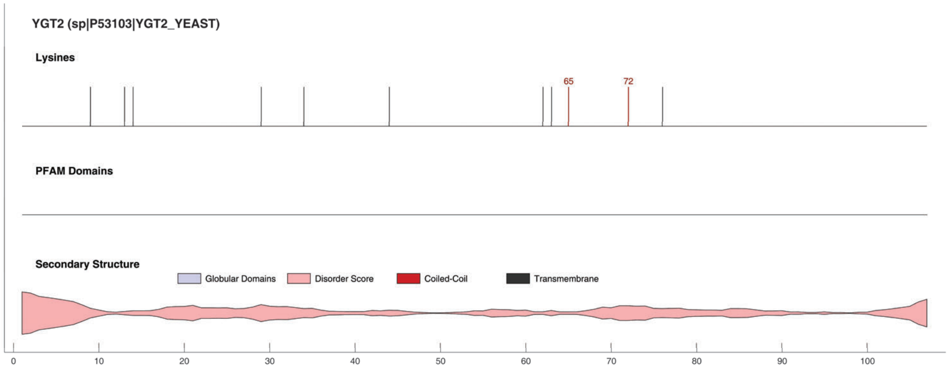
YGT2 (sp|P53103|YGT2_YEAST)
Lysines
65
72
PFAM Domains
Secondary Structure
Globular Domains
Disorder Score
Coiled-Coil
Transmembrane
0
10
20
30
40
50
60
70
80
90
100

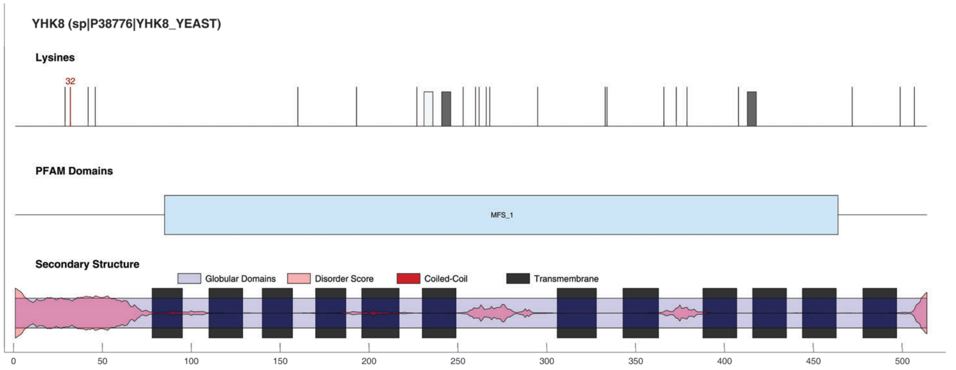
YHK8 (sp|P38776|YHK8_YEAST)
Lysines
32
PFAM Domains
MFS_1
Secondary Structure
Globular Domains
Disorder Score
Coiled-Coil
Transmembrane
0
50
100
150
200
250
300
350
400
450
500

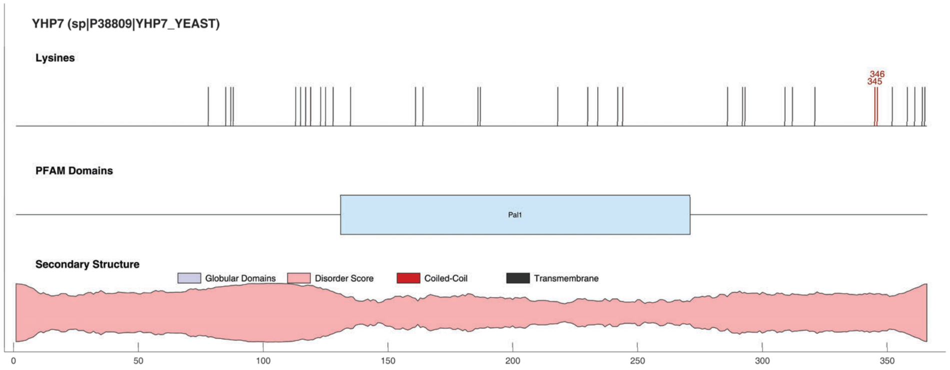
YHP7 (sp|P38809|YHP7_YEAST)
Lysines
346
345
PFAM Domains
Pal1
Secondary Structure
Globular Domains
Disorder Score
Coiled-Coil
Transmembrane
0
50
100
150
200
250
300
350

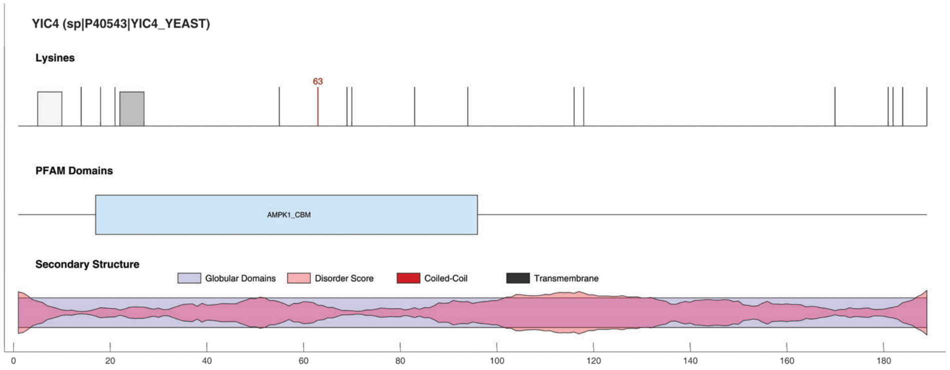
YIC4 (sp|P40543|YIC4_YEAST)
Lysines
63
PFAM Domains
AMPK1_CBM
Secondary Structure
Globular Domains
Disorder Score
Coiled-Coil
Transmembrane
0
20
40
60
80
100
120
140
160
180

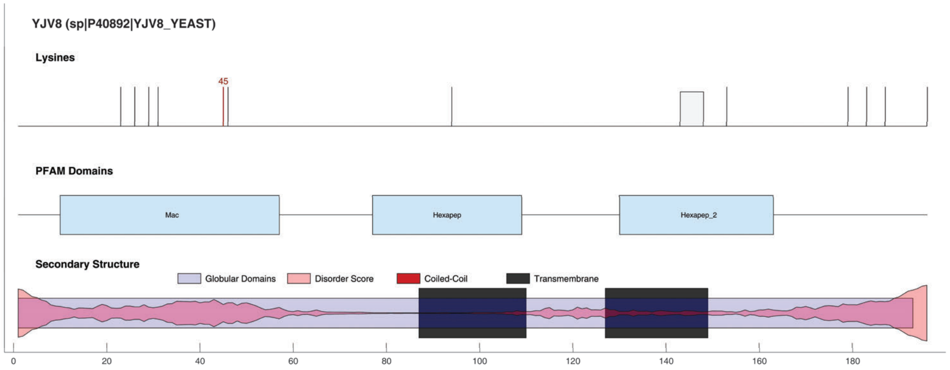
YJV8 (sp|P40892|YJV8_YEAST)
Lysines
45
PFAM Domains
Mac
Hexapep
Hexapep_2
Secondary Structure
Globular Domains
Disorder Score
Coiled-Coil
Transmembrane
0
20
40
60
80
100
120
140
160
180

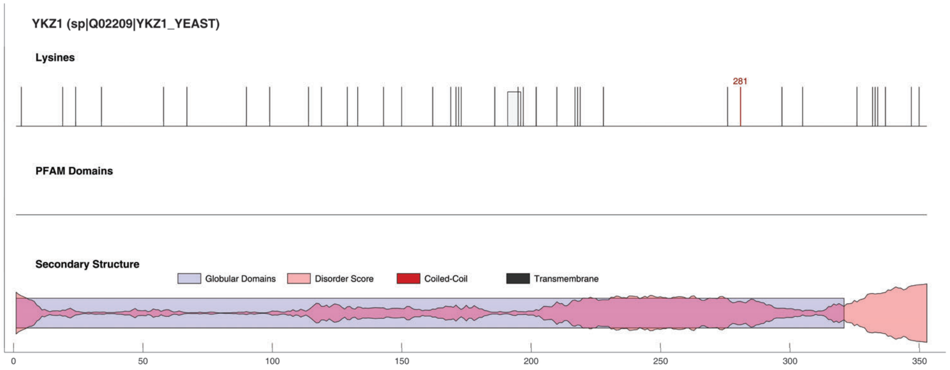
YKZ1 (sp|Q02209|YKZ1_YEAST)
Lysines
281
PFAM Domains
Secondary Structure
Globular Domains
Disorder Score
Coiled-Coil
Transmembrane
0
50
100
150
200
250
300
350

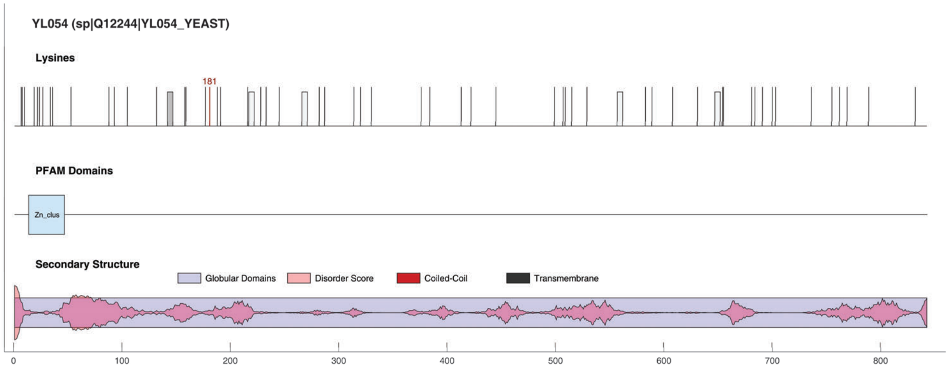
YL054 (sp|Q12244|YL054_YEAST)
Lysines
181
PFAM Domains
Zn_clus
Secondary Structure
Globular Domains
Disorder Score
Coiled-Coil
Transmembrane
0
100
200
300
400
500
600
700
800

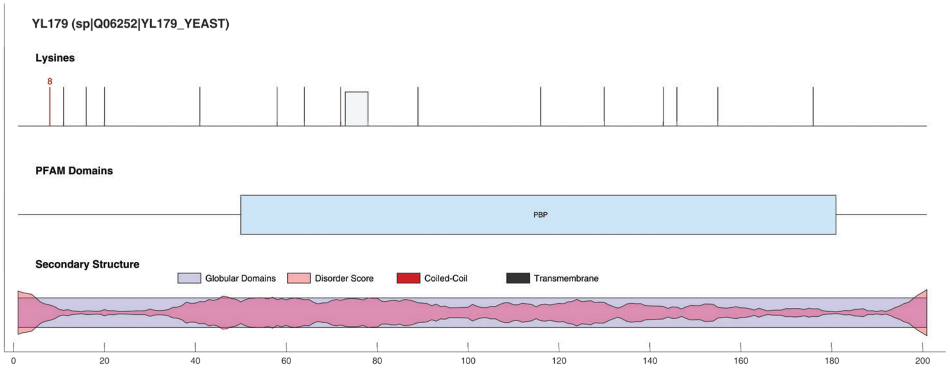
YL179 (sp|Q06252|YL179_YEAST)
Lysines
8
PFAM Domains
PBP
Secondary Structure
Globular Domains
Disorder Score
Coiled-Coil
Transmembrane
0
20
40
60
80
100
120
140
160
180
200

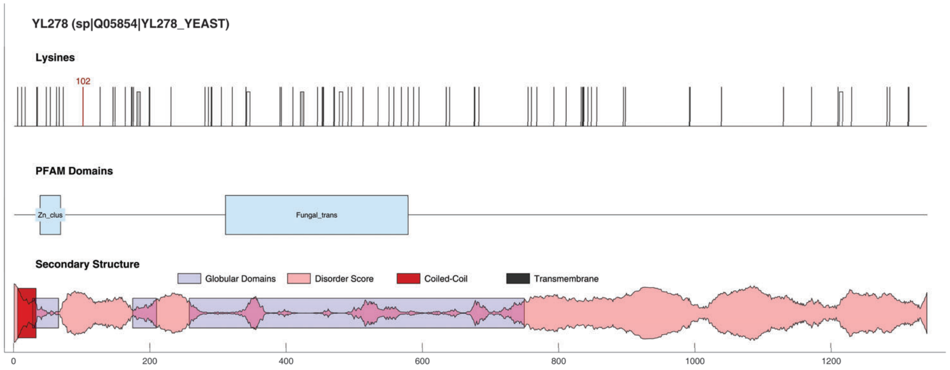
YL278 (sp|Q05854|YL278_YEAST)
Lysines
102
PFAM Domains
Zn_clus
Fungal_trans
Secondary Structure
Globular Domains
Disorder Score
Coiled-Coil
Transmembrane
0
200
400
600
800
1000
1200

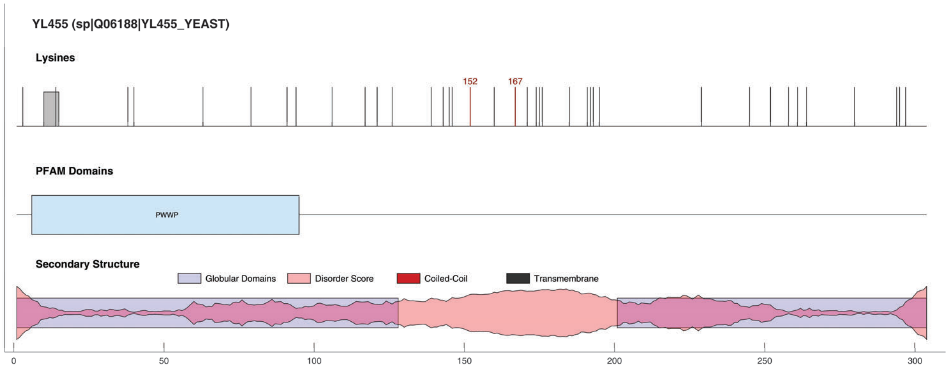
YL455 (sp|Q06188|YL455_YEAST)
Lysines
152
167
PFAM Domains
PWWP
Secondary Structure
Globular Domains
Disorder Score
Coiled-Coil
Transmembrane
0
50
100
150
200
250
300

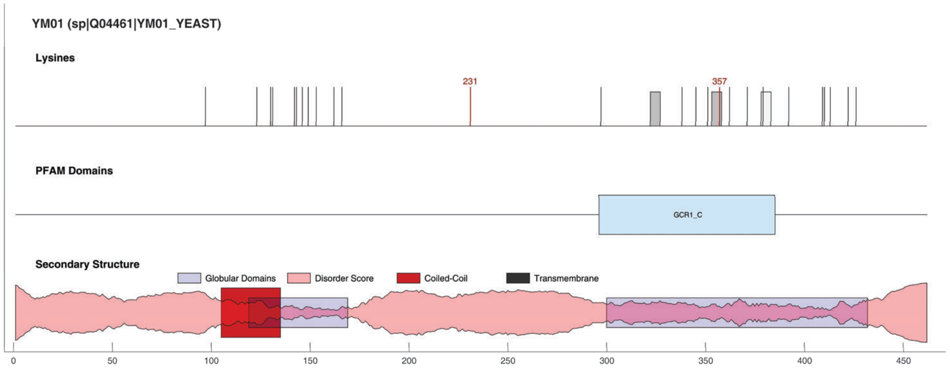
YM01 (sp|Q04461|YM01_YEAST)
Lysines
231
357
PFAM Domains
GCR1_C
Secondary Structure
Globular Domains
Disorder Score
Coiled-Coil
Transmembrane
0
50
100
150
200
250
300
350
400
450

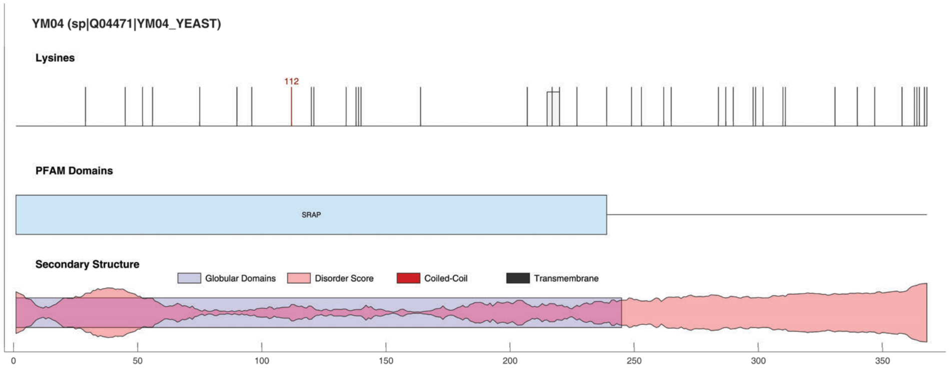
YM04 (sp|Q04471|YM04_YEAST)
Lysines
112
PFAM Domains
SRAP
Secondary Structure
Globular Domains
Disorder Score
Coiled-Coil
Transmembrane
0
50
100
150
200
250
300
350

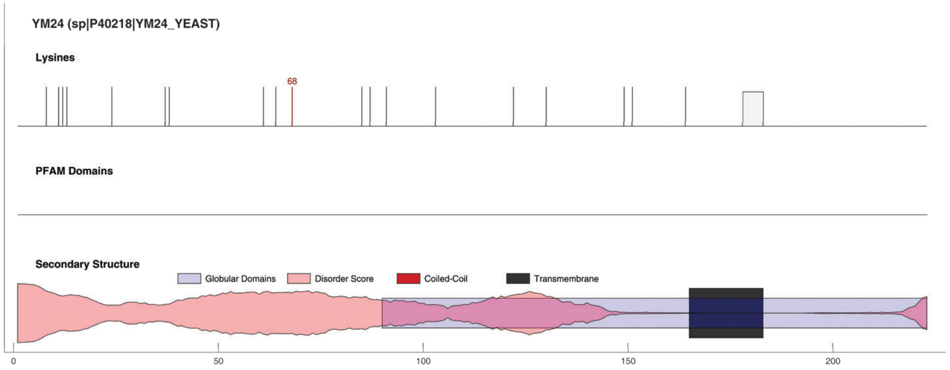
YM24 (sp|P40218|YM24_YEAST)
Lysines
68
PFAM Domains
Secondary Structure
Globular Domains
Disorder Score
Coiled-Coil
Transmembrane
0
50
100
150
200

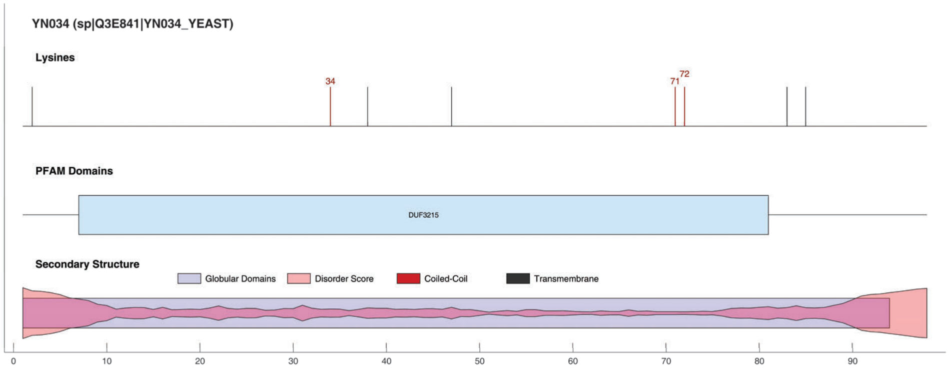
YN034 (sp|Q3E841|YN034_YEAST)
Lysines
34
71
72
PFAM Domains
DUF3215
Secondary Structure
Globular Domains
Disorder Score
Coiled-Coil
Transmembrane
0
10
20
30
40
50
60
70
80
90

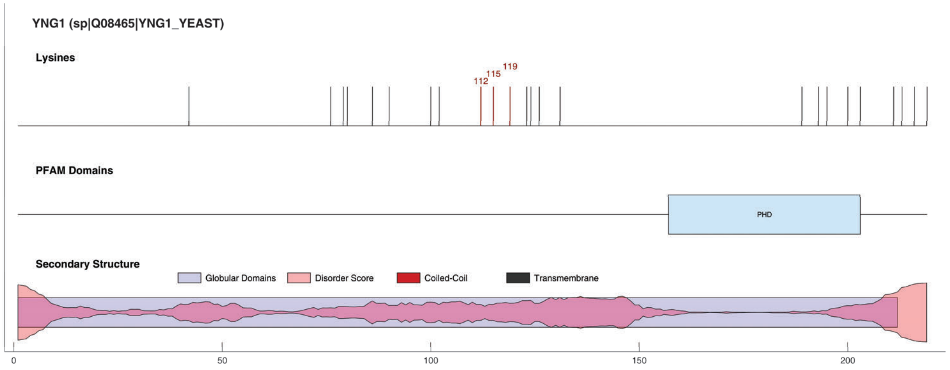
YNG1 (sp|Q08465|YNG1_YEAST)
Lysines
112
115
119
PFAM Domains
PHD
Secondary Structure
Globular Domains
Disorder Score
Coiled-Coil
Transmembrane
0
50
100
150
200

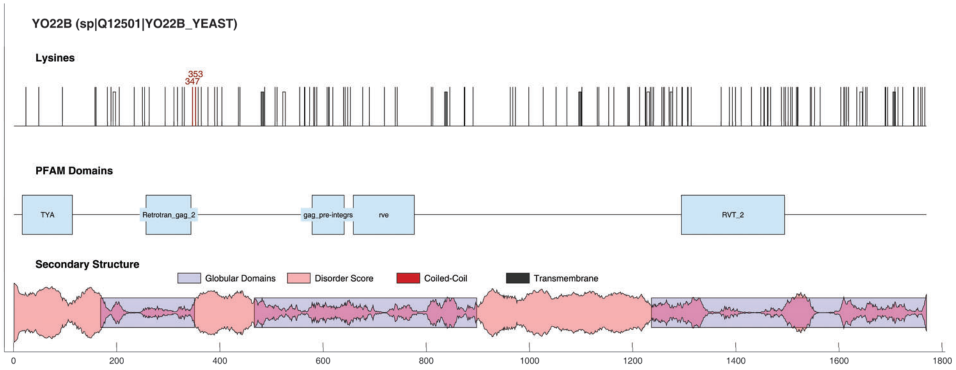
YO22B (sp|Q12501|YO22B_YEAST)
Lysines
353
347
PFAM Domains
TYA
Retrotran_gag_2
gag_pre-integrs
rve
RVT_2
Secondary Structure
Globular Domains
Disorder Score
Coiled-Coil
Transmembrane
0
200
400
600
800
1000
1200
1400
1600
1800

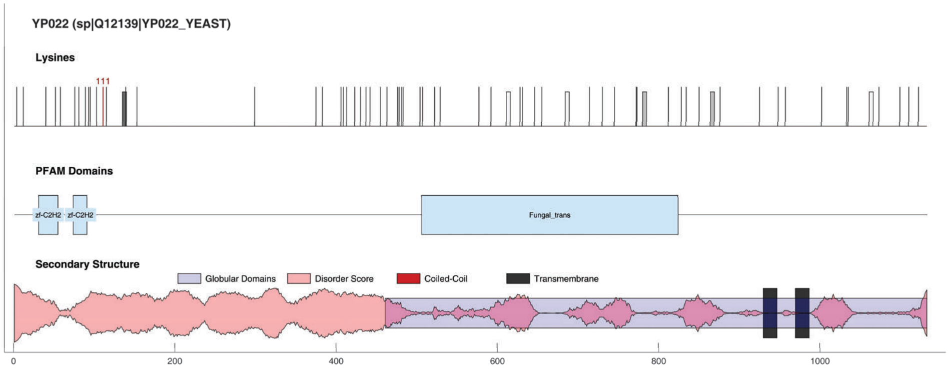
YP022 (sp|Q12139|YP022_YEAST)
Lysines
111
PFAM Domains
zf-C2H2
zf-C2H2
Fungal_trans
Secondary Structure
Globular Domains
Disorder Score
Coiled-Coil
Transmembrane
0
200
400
600
800
1000

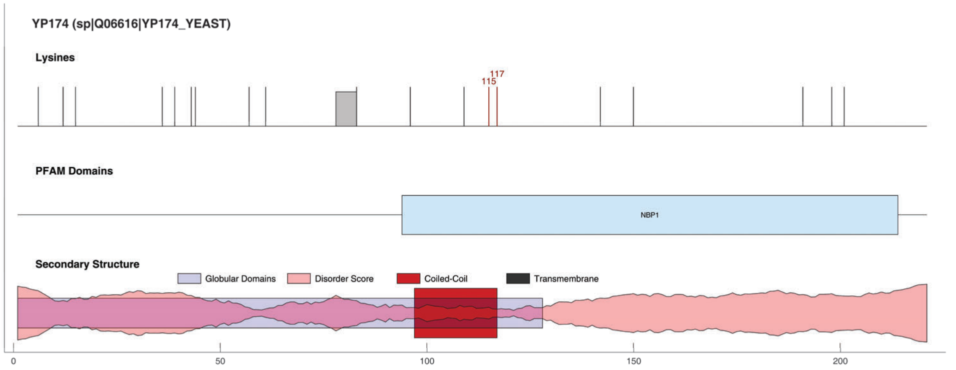
YP174 (sp|Q06616|YP174_YEAST)
Lysines
117
115
PFAM Domains
NBP1
Secondary Structure
Globular Domains
Disorder Score
Coiled-Coil
Transmembrane
0
50
100
150
200

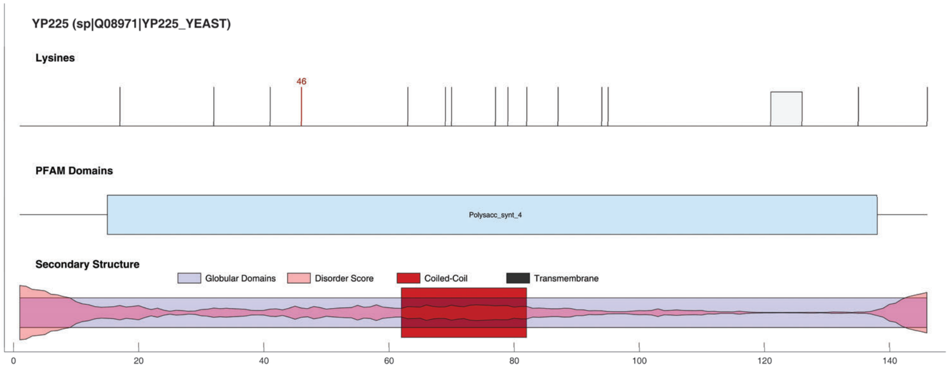
YP225 (sp|Q08971|YP225_YEAST)
Lysines
46
PFAM Domains
Polysacc_synt_4
Secondary Structure
Globular Domains
Disorder Score
Coiled-Coil
Transmembrane
0
20
40
60
80
100
120
140

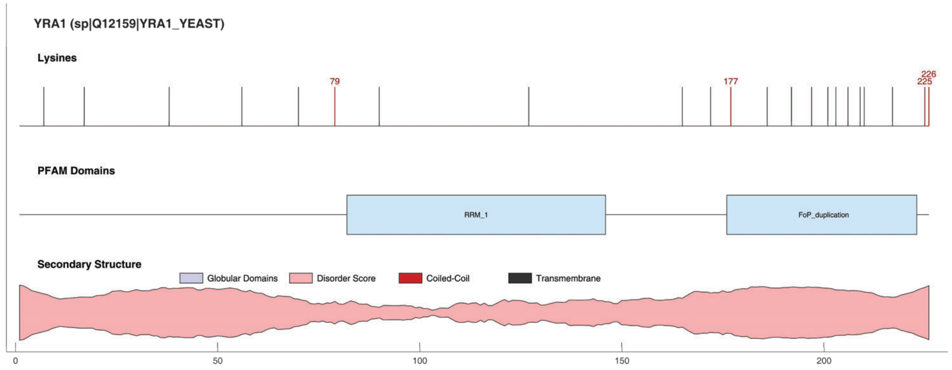
YRA1 (sp|Q12159|YRA1_YEAST)
Lysines
79
177
225
226
PFAM Domains
RRM_1
FoP_duplication
Secondary Structure
Globular Domains
Disorder Score
Coiled-Coil
Transmembrane
0
50
100
150
200

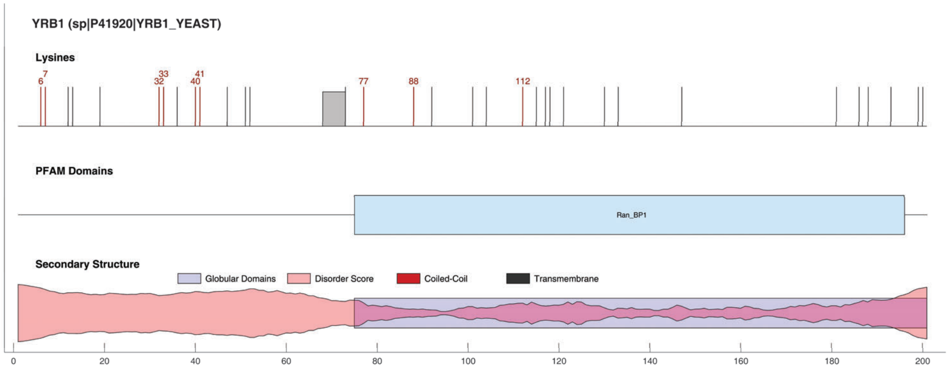
YRB1 (sp|P41920|YRB1_YEAST)
Lysines
6
7
32
33
40
41
77
88
112
PFAM Domains
Ran_BP1
Secondary Structure
Globular Domains
Disorder Score
Coiled-Coil
Transmembrane
0
20
40
60
80
100
120
140
160
180
200

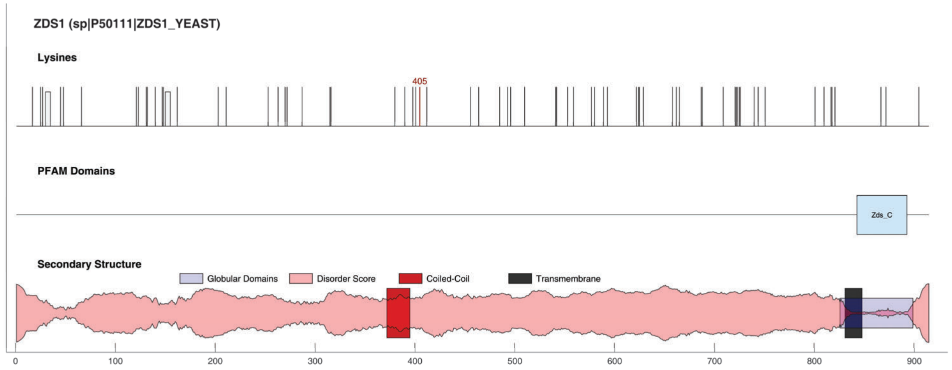
ZDS1 (sp|P50111|ZDS1_YEAST)
Lysines
405
PFAM Domains
Zds_C
Secondary Structure
Globular Domains
Disorder Score
Coiled-Coil
Transmembrane
0
100
200
300
400
500
600
700
800
900

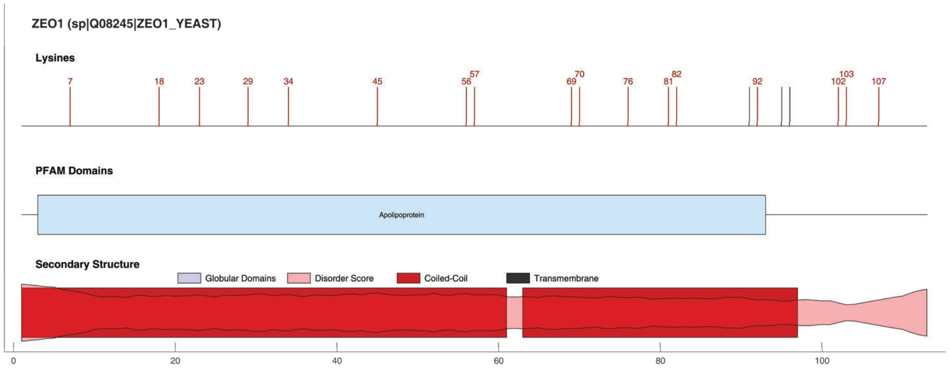
ZEO1 (sp|Q08245|ZEO1_YEAST)
Lysines
7 18 23 29 34 45 56 57 69 70 76 81 82 92 102 103 107
PFAM Domains
Apolipoprotein
Secondary Structure
Globular Domains
Disorder Score
Coiled-Coil
Transmembrane
0 20 40 60 80 100

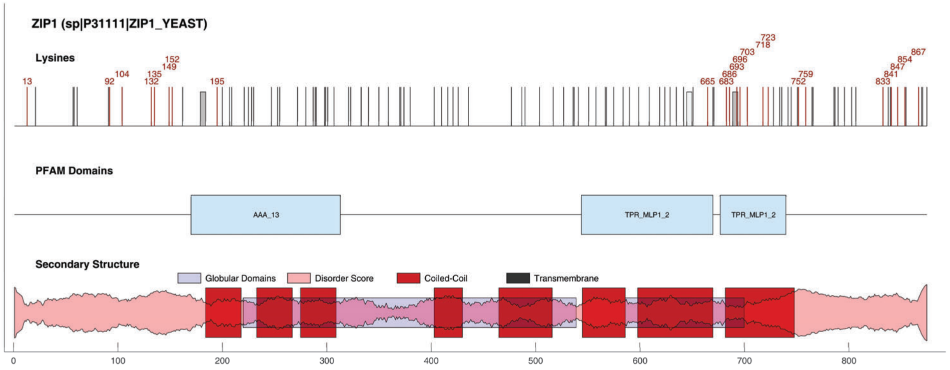
ZIP1 (sp|P31111|ZIP1_YEAST)
Lysines
13 92 104 132 135 149 152 195 665 683 686 693 696 703 718 723 752 759 833 841 847 854 867
PFAM Domains
AAA_13
TPR_MLP1_2
TPR_MLP1_2
Secondary Structure
Globular Domains
Disorder Score
Coiled-Coil
Transmembrane
0 100 200 300 400 500 600 700 800

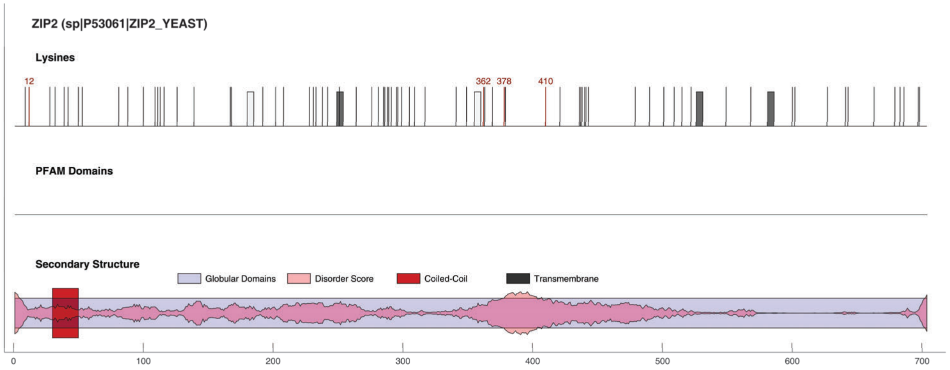
ZIP2 (sp|P53061|ZIP2_YEAST)
Lysines
12 362 378 410
PFAM Domains
Secondary Structure
Globular Domains
Disorder Score
Coiled-Coil
Transmembrane
0 100 200 300 400 500 600 700

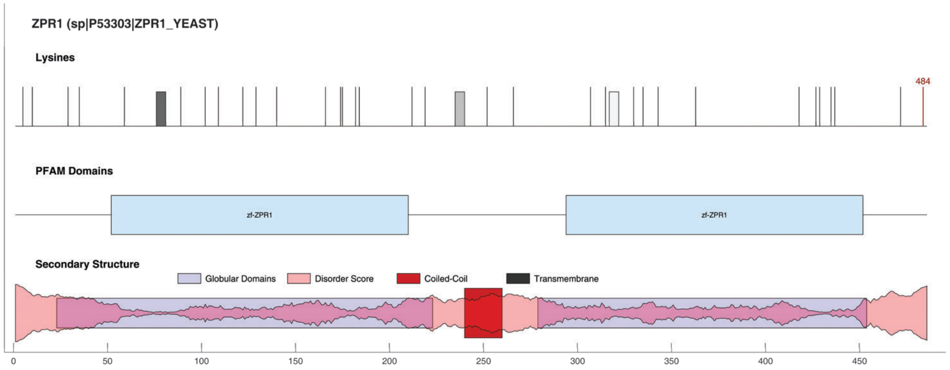
ZPR1 (sp|P53303|ZPR1_YEAST)
Lysines
484
PFAM Domains
zf-ZPR1
zf-ZPR1
Secondary Structure
Globular Domains
Disorder Score
Coiled-Coil
Transmembrane
0 50 100 150 200 250 300 350 400 450

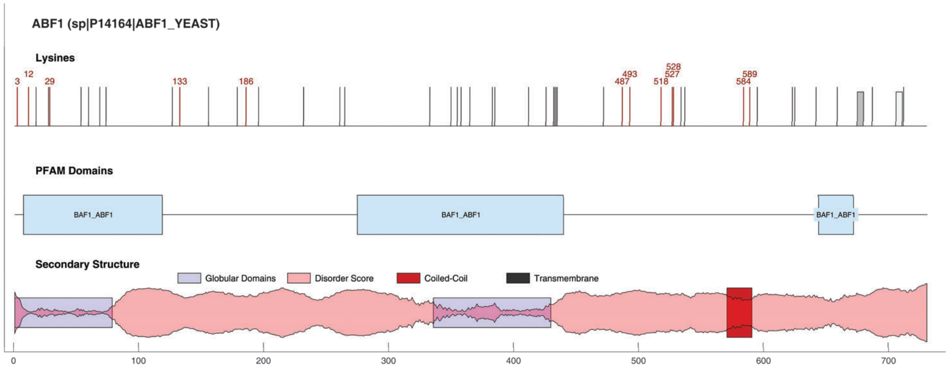

ABF1 (sp|P14164|ABF1_YEAST)
Lysines
3 12 29 133 186 487 493 518 527 528 584 589
PFAM Domains
BAF1_ABF1
BAF1_ABF1
BAF1_ABF1
Secondary Structure
Globular Domains
Disorder Score
Coiled-Coil
Transmembrane
0 100 200 300 400 500 600 700

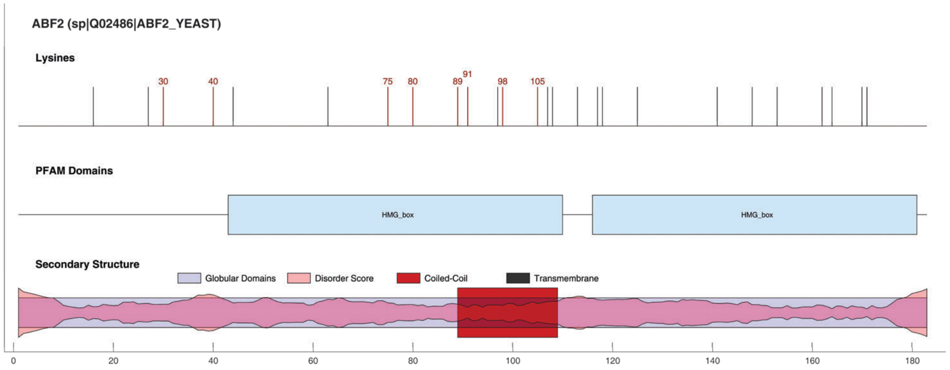

ABF2 (sp|Q02486|ABF2_YEAST)
Lysines
30 40 75 80 89 91 98 105
PFAM Domains
HMG_box
HMG_box
Secondary Structure
Globular Domains
Disorder Score
Coiled-Coil
Transmembrane
0 20 40 60 80 100 120 140 160 180

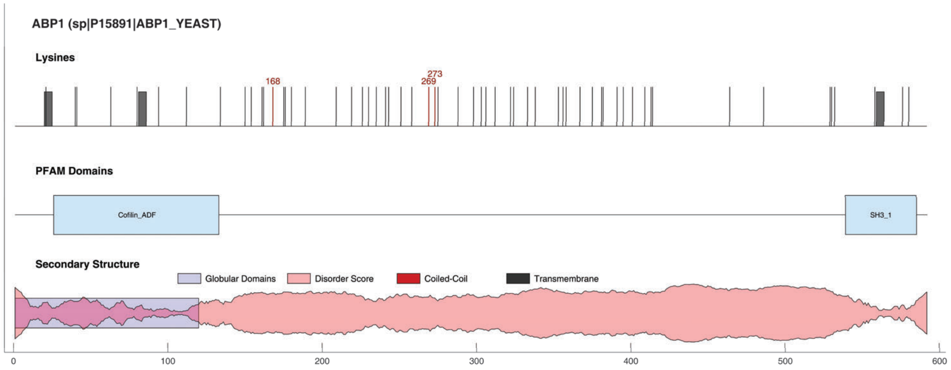

ABP1 (sp|P15891|ABP1_YEAST)
Lysines
168 269 273
PFAM Domains
Cofilin_ADF
SH3_1
Secondary Structure
Globular Domains
Disorder Score
Coiled-Coil
Transmembrane
0 100 200 300 400 500 600

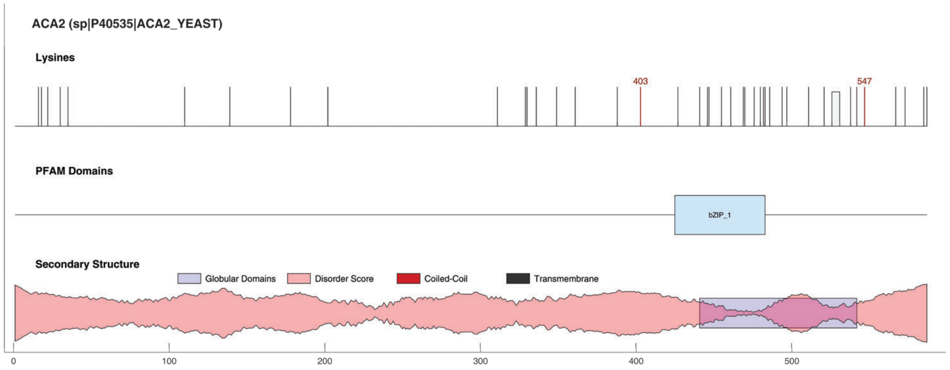

ACA2 (sp|P40535|ACA2_YEAST)
Lysines
403 547
PFAM Domains
bZIP_1
Secondary Structure
Globular Domains
Disorder Score
Coiled-Coil
Transmembrane
0 100 200 300 400 500

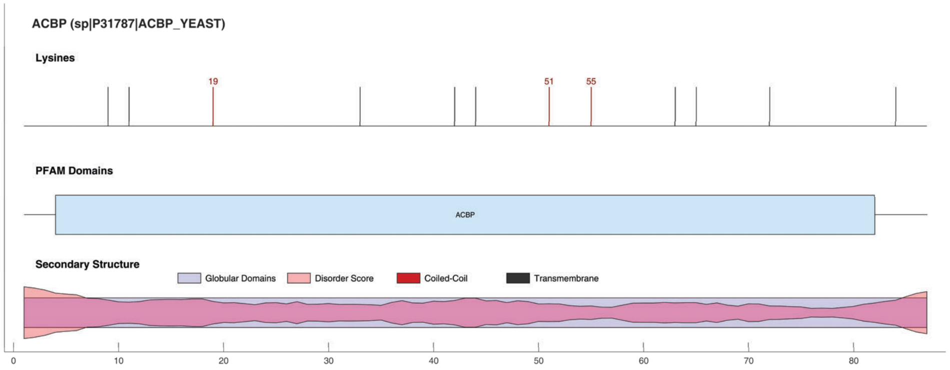
ACBP (sp|P31787|ACBP_YEAST)
Lysines
19
51
55
PFAM Domains
ACBP
Secondary Structure
Globular Domains
Disorder Score
Coiled-Coil
Transmembrane
0
10
20
30
40
50
60
70
80

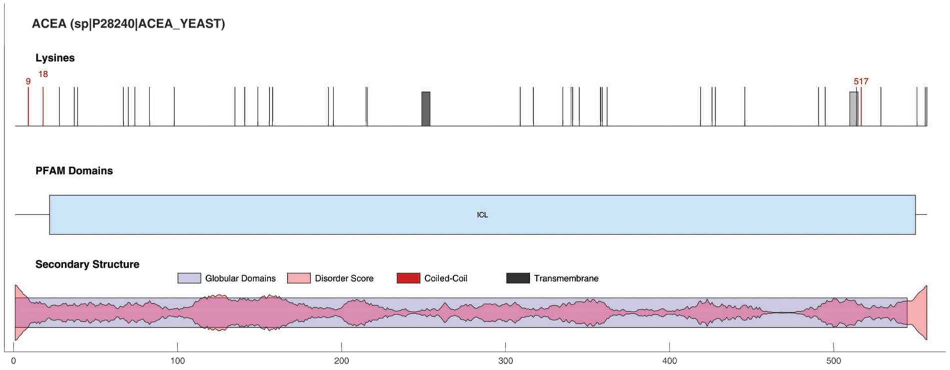
ACEA (sp|P28240|ACEA_YEAST)
Lysines
9
18
517
PFAM Domains
ICL
Secondary Structure
Globular Domains
Disorder Score
Coiled-Coil
Transmembrane
0
100
200
300
400
500

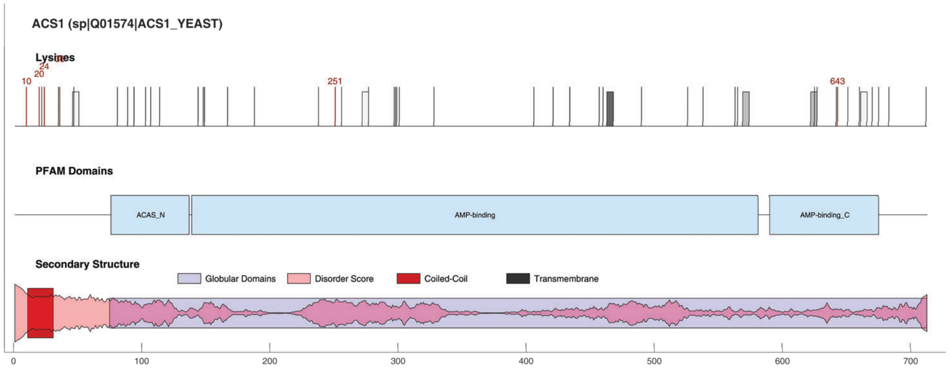
ACS1 (sp|Q01574|ACS1_YEAST)
Lysines
10
20
24
251
643
PFAM Domains
ACAS_N
AMP-binding
AMP-binding_C
Secondary Structure
Globular Domains
Disorder Score
Coiled-Coil
Transmembrane
0
100
200
300
400
500
600
700

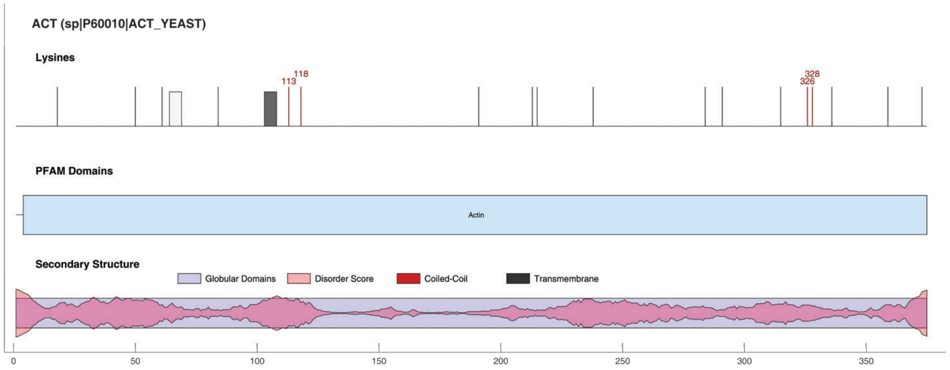
ACT (sp|P60010|ACT_YEAST)
Lysines
113
118
326
328
PFAM Domains
Actin
Secondary Structure
Globular Domains
Disorder Score
Coiled-Coil
Transmembrane
0
50
100
150
200
250
300
350
